# GRHL3 binding and enhancers rearrange as epidermal keratinocytes transition between functional states

**DOI:** 10.1371/journal.pgen.1006745

**Published:** 2017-04-26

**Authors:** Rachel Herndon Klein, Ziguang Lin, Amelia Soto Hopkin, William Gordon, Lam C. Tsoi, Yun Liang, Johann E. Gudjonsson, Bogi Andersen

**Affiliations:** 1 Department of Biological Chemistry, School of Medicine, University of California Irvine, Irvine, California, United States of America; 2 Department of Dermatology, University of Michigan, Ann Arbor, Michigan, United States of America; 3 Department of Computational Medicine & Bioinformatics, University of Michigan, Ann Arbor, Michigan, United States of America; 4 Department of Biostatistics, University of Michigan, Ann Arbor, Michigan, United States of America; 5 Center for Complex Biological Systems, University of California Irvine, Irvine, California, United States of America; 6 Department of Medicine, School of Medicine, University of California Irvine, Irvine, California, United States of America; University of Bradford, UNITED KINGDOM

## Abstract

Transcription factor binding, chromatin modifications and large scale chromatin re-organization underlie progressive, irreversible cell lineage commitments and differentiation. We know little, however, about chromatin changes as cells enter transient, reversible states such as migration. Here we demonstrate that when human progenitor keratinocytes either differentiate or migrate they form complements of typical enhancers and super-enhancers that are unique for each state. Unique super-enhancers for each cellular state link to gene expression that confers functions associated with the respective cell state. These super-enhancers are also enriched for skin disease sequence variants. GRHL3, a transcription factor that promotes both differentiation and migration, binds preferentially to super-enhancers in differentiating keratinocytes, while during migration, it binds preferentially to promoters along with REST, repressing the expression of migration inhibitors. Key epidermal differentiation transcription factor genes, including *GRHL3*, are located within super-enhancers, and many of these transcription factors in turn bind to and regulate super-enhancers. Furthermore, GRHL3 represses the formation of a number of progenitor and non-keratinocyte super-enhancers in differentiating keratinocytes. Hence, chromatin relocates GRHL3 binding and enhancers to regulate both the irreversible commitment of progenitor keratinocytes to differentiation and their reversible transition to migration.

## Author summary

The epidermis, a continuously renewing epithelium, balances proliferation and differentiation during development and homeostasis. During wound healing epidermal keratinocytes become migratory to close the wound. The transition of keratinocytes between these three different states—progenitor, differentiation and migration-requires the activation of distinct gene expression programs whose regulation is poorly understood. Our study demonstrates how regulatory chromatin domains like typical enhancers and super enhancers interact with transcription factors, resulting in complex layers of regulation with specific transcription factors regulating distinct stages of the epidermal differentiation process. Specifically, the epidermal transcription factor GRHL3 regulates both possible transitions of progenitor epidermal cell: differentiation and migration. We found that both GRHL3 chromatin binding and enhancers relocate as keratinocytes move between functional states. In addition, we show reciprocal regulation between GRHL3 and enhancers: chromatin domains, including a super enhancer, regulate *GRHL3* expression, while GRHL3 regulates the formation of a subset of epidermal super enhancers and typical enhancers.

## Introduction

Gene expression changes in stem cells committing to cellular lineages correspond to large scale reorganization of epigenetic regulatory structures, including super-enhancers (SEs) [1–4], which are thought to be more important than typical enhancers (TEs) in controlling cell identity [1, 5–7]. Less is known about the chromatin regulatory changes corresponding to transitions of committed cell types to reversible functional states, including migration. Here we used primary human epidermal keratinocytes [8–12] to investigate the genomic regulatory structures underpinning different functional states of a committed cell type.

As epidermal progenitors in the basal layer exit the cell cycle, they move towards the surface of the skin, progressively differentiating by activating gene expression programs required for epidermal barrier formation [13]. During early wound healing, however, these progenitors migrate to cover the wound, activating a gene expression program distinct from that of differentiation [14]. While both migration and differentiation require distinct gene expression changes, chromatin changes in transient functional states such as migration remain uncharacterized.

Essential for embryonic epidermal differentiation and barrier formation, and adult epidermal repair, the transcription factor Grainyhead-like 3 (GRHL3; also referred to as GET1) activates gene expression programs required for cell adhesion, lipid production, cornified envelope formation and protein crosslinking [15–17]. Intriguingly, GRHL3 is also essential for normal keratinocyte migration during eyelid closure and wound healing [18–20] where it modulates gene expression programs that promote the movement of keratinocytes and suppress the progenitor and differentiation states. How a single transcription factor GRHL3 can promote both differentiation and migration of a single cell type remains poorly understood.

The formation of unique complements of enhancers, genomic regulatory regions residing at a distance from their target promoters, is critical for cell type specifications. Initially discovered as short regions that activate transcription independent of orientation or location relative to target promoters [21], enhancers are now known to be bound by active transcription factors [22]. More recently, advances in DNA sequencing enabled the identification of gene regulatory regions based on histone modifications. High levels of H3K4me1 and low levels of H3K4me3 mark enhancers; during enhancer activation, high levels of H3K27ac are also found [23, 24]. Poised enhancers, so named because they are repressed while primed for rapid activation, have both H3K4me1 and the repressive mark H3K27me3 [23].

Recent work classified enhancers into TEs (usually about 1–2 kb long) and SEs that are longer (greater than 12.5kb) with higher intensity of cooperatively binding transcription factors and higher H3K27Ac activation marks [1]. By driving the expression of cell identity genes, SEs are more important for cell type specifications than TEs [1, 4, 6]. Whereas recent studies mapped SEs in human epidermal keratinocytes during differentiation and showed that transcription factor p63 [2] and DNA methylation enzymes DNMT3A and DNMT3B [25] bind and regulate their activity, the regulatory role of SEs for reversible functional states like migration remains unexplored. Also, we don’t know if transcription factors such as GRHL3 regulate the formation of TEs and SEs in epidermal keratinocytes.

To address the aforementioned knowledge gaps, we defined the TE and SE complements in progenitor, migrating, and differentiating keratinocytes. Combining this data with gene expression data after siRNA knockdowns of 50 epidermal differentiation-associated transcription factors, we gained a high-level view of the transcriptional regulation of transitions between different epidermal functional states. A focus on one of these transcription factors, GRHL3, provides mechanistic insight into how a single transcription factor can control distinct gene expression programs under different physiological states within the same cell type. Our studies suggest that GRHL3 regulates divergent gene expression programs in differentiating and migrating keratinocytes by switching locations of chromatin binding in the context of enhancer landscapes that are distinct for each state.

## Results

### The active enhancer landscape is highly dynamic as progenitor keratinocytes transition to differentiation or migration

Transitions between different functional states can be modeled with primary human epidermal keratinocytes (NHEK); we used this system in our studies. From a proliferative, progenitor-like state, NHEKs are differentiated by raising the calcium concentration, and induced to migrate by scratching out parts of the monolayer surface, triggering cells to migrate to close the “wound” [8–12].

**Fig 1:**
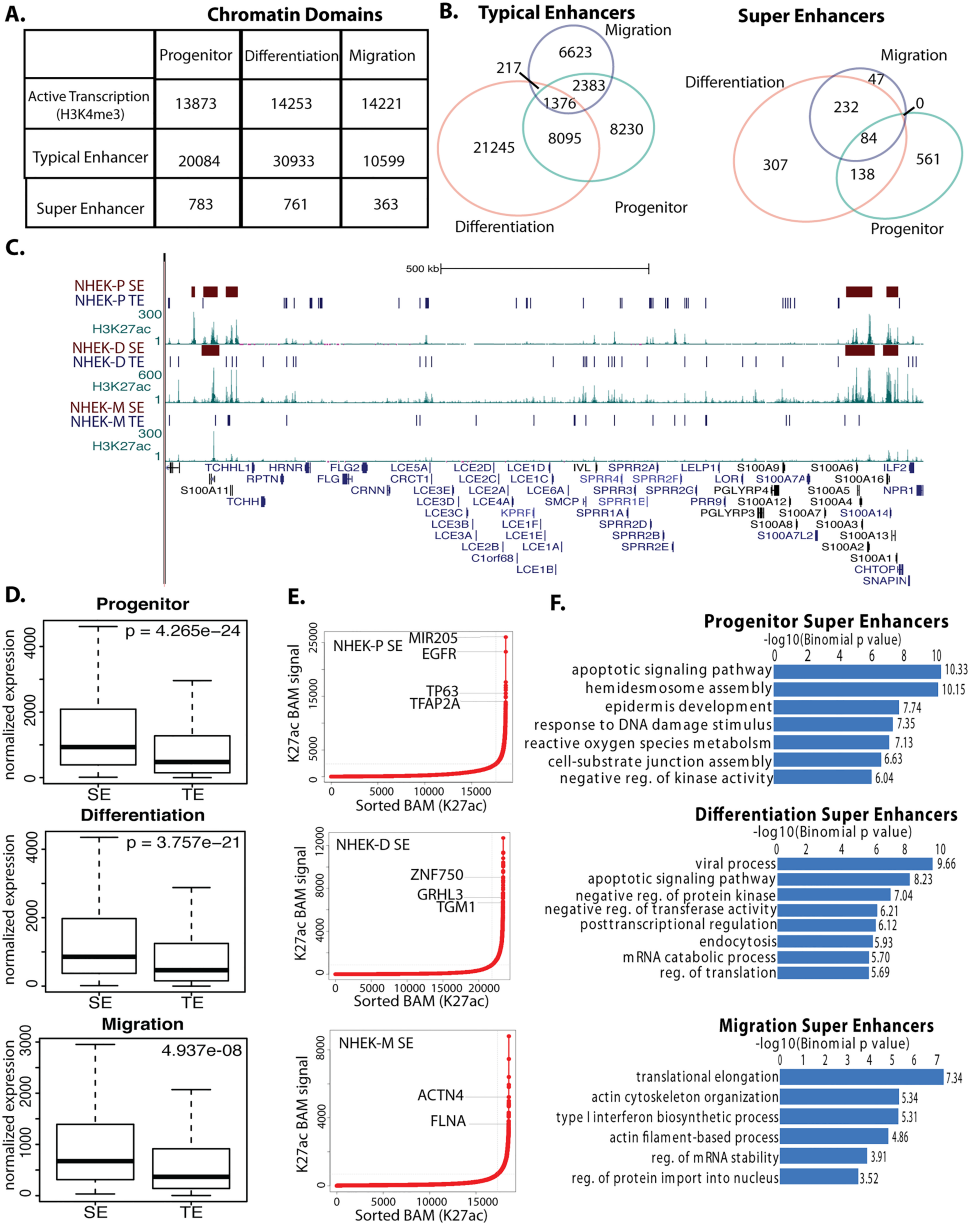
Identification and characterization of typical enhancers (TE) and super enhancers (SE) in different keratinocyte states. (A) Identification of chromatin regulatory domains in progenitor, differentiating, and migrating keratinocytes through ChIP-Seq profiling of histone modifications. Indicated are regions with active transcription (marked by H3K4me3), typical enhancers (H3K4me1 and H3K27ac with low levels of H3K4me3), and super enhancers (regions with highest H3K27ac, at least 12.5kb). (B) Overlap of TEs (left Venn diagram) and SEs (right Venn diagram) among three different keratinocyte states. (C) Plot of the H3K27ac signal and called SE and TE peaks at the Epidermal Differentiation Complex (EDC) in NHEK-D, NHEK-P, and NHEK-M. (D) Comparison of the expression levels of nearest gene to TEs and SEs in NHEK-P, NHEK-D, and NHEK-M. Significance determined with T-test. (E) Plot of the H3K27ac signal at all enhancers highlighting selective genes linked to top SEs. (F) Gene ontology categories for nearest gene to all SEs in NHEK-P, NHEK-D, or NHEK-M.

As GRHL3 binds to distal regulatory regions during keratinocyte differentiation [26], we first defined the enhancer complement in NHEKs. We employed ChIP-Seq to define active regulatory regions based on histone modifications in migrating (NHEK-M) and differentiating (NHEK-D) keratinocytes, and used comparable data on progenitor state keratinocytes (NHEK-P) from the ENCODE project [27]. Defining active TEs based on the presence of H3K4me1 and H3K27ac, and the absence of high levels of H3K4me3, we identified approximately 20,000 TEs in NHEK-P, 31,000 in NHEK-D, and 11,000 in NHEK-M (Fig 1A). We also defined SEs using the H3K27ac mark [4, 6], identifying 783, 761, and 363 SEs in NHEK-P, NHEK-D, and NHEK-M, respectively (Fig 1A). The typical sizes of TEs and SEs were approximately 2kb and 50–100 kb, respectively (S1A Fig). As previously shown [1], SEs are on average closer to the nearest gene than TEs (S1B Fig), localize more frequently to tissue specific genes than housekeeping genes (S1C Fig), and are enriched in Mediator 1 (MED1) binding (S1D Fig).

During the transition from the progenitor state to differentiation, 9,471 TEs persist while 21,462 are activated and 10,613 are inactivated (Fig 1B). In the progenitor to migration transition, 3,759 TEs persist while 6,840 are activated and 16,235 are inactivated (Fig 1B). Despite the different number of enhancers detected in NHEK-D and NHEK-M, perhaps related to different sequencing depth, a similar percentage of TEs are activated in the transition from NHEK-P to either NHEK-D (69%) or NHEK-M (65%). Thus, the majority of TEs are active only in one functional state, and only 1,376 TEs overlap between all three conditions (Fig 1B); a list of the nearest gene to the overlapping TEs showed significant enrichment in functional categories related to kinase and Notch signaling, keratinocyte development, and wound healing (S2A Fig).

We found analogous dynamic patterns with SEs with only 84 SEs persisting in all 3 cell states (Fig 1B). The SEs are near epidermal differentiation genes, including intermediate filament organization genes, and link to mouse phenotypes that suggest a role in regulating cell size (S2B Fig). Normalizing to the total number of TEs or SEs in NHEK-P and NHEK-M, about 2-fold more TEs than SEs persist when progenitor cells migrate (20% of all possible TEs in NHEK-P or NHEK-M persist while 8% of all possible SEs between NHEK-P and NHEK-M persist), indicating that SEs are more specific for functional states than TEs.

### SEs locate to edges of important epidermal gene clusters, are close to highly expressed genes that confer functional keratinocyte states, and are the preferred location of skin disease-associated SNPs

Whereas TEs are scattered at multiple locations throughout the epidermal differentiation complex (EDC) on chromosome 1, SEs are positioned at the edges of the EDC; these EDC-flanking SEs are already established in NHEK-P and persist in NHEK-D (Fig 1C, S3 Fig). We observed similar flanking SEs in the keratin gene clusters on chromosomes 12 and 17 (S4A and S4B Fig), perhaps indicative of higher order chromatin structure at these important gene cluster loci. In contrast to a prominent border location in the EDC and keratin gene clusters, SEs span the majority of the *HOXA* and *HOXC* loci (S4C Fig).

SE-associated genes are more highly expressed than TE-associated genes under each of the three functional states (Fig 1D), underscoring SEs’ role as more powerful enhancers than TEs. The SEs with the highest intensity of H3K27Ac link to key regulators of each functional state. The top SEs in NHEK-P overlap progenitor-promoting genes *Mir205*, *EGFR*, and *TP63*; the top SEs in NHEK-D overlap pro-differentiation genes *ZNF750* and *GRHL3*; while the top SEs in NHEK-M overlap pro-migration genes *ACTN4* and *FLNA* (Fig 1E). Consistently, the genes associated with SEs in each of the cell states have functions characteristic of the particular cell state (Fig 1F). For example, in NHEK-P, which normally adhere to the basal lamina, SE-associated genes are important for hemidesmosome assembly and epidermal identity. SE-associated genes in NHEK-D are important for the regulation of apoptosis and differentiation (Fig 1F). SE-associated genes in NHEK-M are important for cell migration, including actin filament regulation (Fig 1F). The top enriched functional categories for genes linked to unique SEs in each cell state are different than for TE linked genes (S5 Fig).

**Fig 2:**
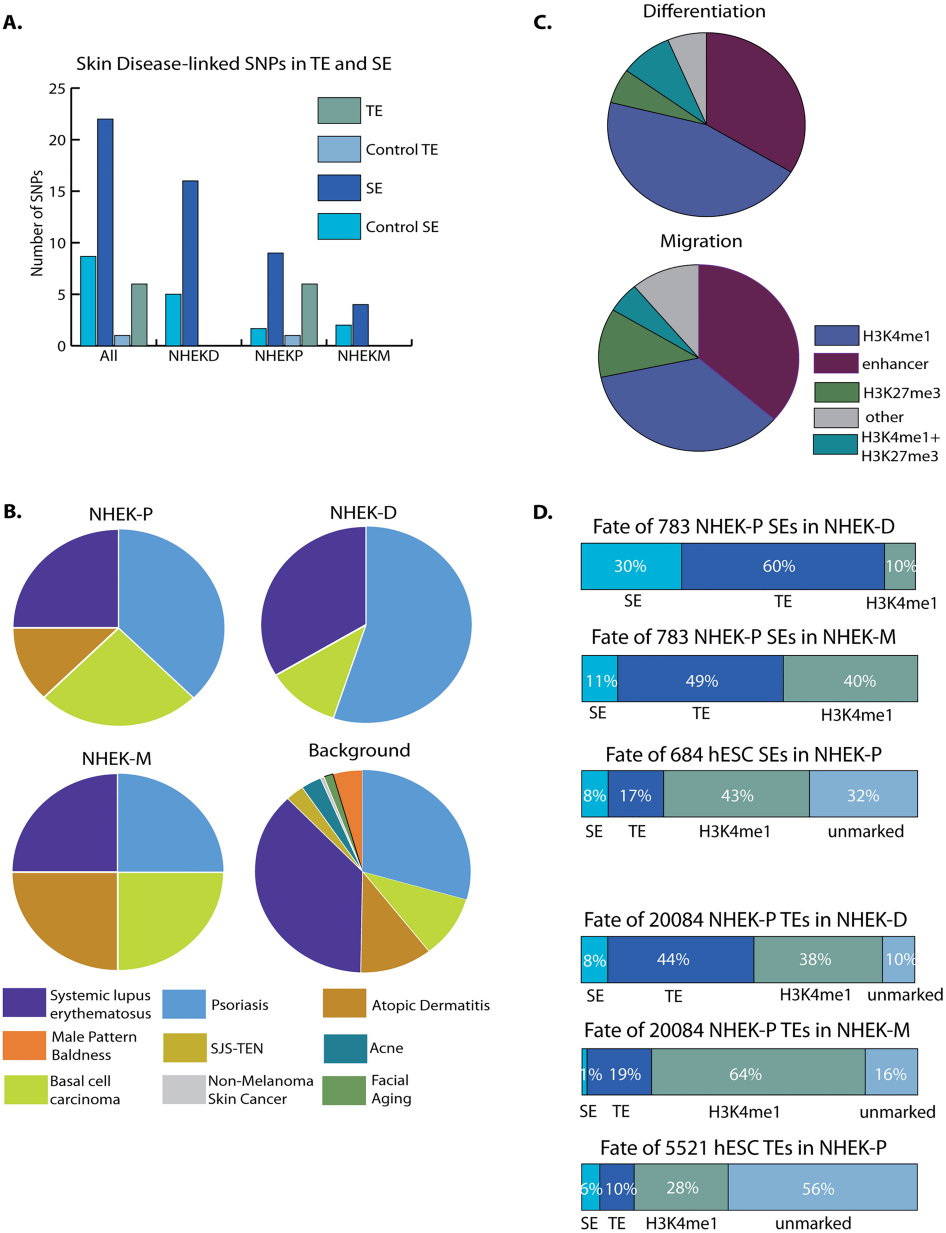
Association of SNPs with SEs and comparison of chromatin domains across keratinocyte cell states. (A) Overlap of SEs and TEs in three NHEK states with skin disease SNPs. (B) Distribution of skin disease SNPs in SEs of NHEK-P, NHEK-D, or NHEK-M, compared with background distribution of SNPs. (C) Comparison of the chromatin landscape of NHEK-D (top pie chart) and NHEK-M (lower) at regions labeled as active enhancers (both TEs and SEs) in NHEK-P cells. (D) Comparison of changes in SEs and TEs as cells differentiate from embryonic stem cells (ESC) to keratinocyte progenitors (P) with changes that occur in the transition between keratinocyte cell states: P to M and P to D.

SEs in other cell types are enriched for SNPs associated with corresponding organ-specific diseases [6]. We found that SNPs associated (i.e. p < 5e-08) with complex skin diseases (psoriasis, atopic dermatitis, alopecia, basal cell carcinoma, severe acne, androgenic alopecia, facial aging, and Stevens-Johnson syndrome) are significantly enriched in SEs, particularly in NHEK-D SEs, compared to random regions of similar size (Chi-square test, p<3.2E-6) (Fig 2A and 2B, Table 1**)**. There is a small but noticeable dip in H3K27ac signal adjacent to these SNPs (S6 Fig), indicating these SNPs might be located at the edge of transcription factor binding domains. These SNPs are also more highly enriched in SEs than TEs, pointing to the importance of SEs in maintaining normal function of the epidermis and the likelihood that common skin disease gene variants disrupt the function of SEs.

**Table 1:** SNPs linked to epidermal diseases overlapping SE in NHEK-D, NHEK-P, and NHEK-M.

**NHEK-D SE**				
**SNP**	**Location**	**Disease**	**Nearby genes**	**Location**
chr5	150467189	psoriasis	Tnip1, Anxa6	Promoter
chr10	81032532	psoriasis	Zmiz1, Pp1f, Zcchc24	Intron
chr11	64135298	psoriasis	Rps6ka4, Mir1237	Intron
chr22	21979289	psoriasis	Vdjc, Ccdc116, Ube2l3	Intergenic
chr19	10818092	psoriasis	Qtrt1, Ilf3, Dnm2	Intron
chr10	75599127	psoriasis	Camk2g, Plau, Ndst2	Intron
chr5	1322087	Basal cell carcinoma	Clptm1l, Mir4457, Tert	Intron
chr7	130585553	Basal cell carcinoma	Mir29a, Mir29b1	Intergenic
chr1	17722363	Basal cell carcinoma	Padl6, Padl4, Rcc2	Intron
chr16	89986117	Basal cell carcinoma	Tubb3, Tcf25, Def8	Promoter
chr20	62328742	Atopic dermatitis	Arfrp1, Tnfrsf6b, Zgpat	Promoter/Exon
chr2	74208362	Systemic lupus erythematosus	Dgouk, Tet3, Mir598	Intergenic
chr6	32158319	Atopic dermatitis	Notch4, Gpsm3, Pbx2, Ager	Promoter
chr5	150458146	Systemic lupus erythematosus	Tnip1, Anxa6, Gpx3	Intron
chr11	589564	Systemic lupus erythematosus	Phrf1, Mir210hg, Irf7	Intron
chr17	4712617	Systemic lupus erythematosus	Pld2, Psmb6, Gltpd2	Exon
**NHEK-P SE**				
**SNP**	**Location**	**Disease**	**Nearby genes**	**Location**
chr5	150467189	psoriasis	Tnip1, Anxa6	Promoter
chr6	111913262	psoriasis	Traf3ip2, Fvn, Rev3l	Exon
chr9	110817020	psoriasis	Klf4, Actl7b	Intergenic
chr11	109962432	psoriasis	Zc3h12c, Rdx	Intergenic
chr3	189615475	psoriasis	Tp63, Leprel1, Mir944	Intergenic
chr7	130585553	Basal cell carcinoma	Mir29a, Mir29b1	Intergenic
chr10	63805617	Systemic lupus erythematosus	Arid5b, Rtkn2	Intron
chr13	41558110	Systemic lupus erythematosus	Elf1, Sugt1p, Wbp4	Intron
chr5	150458146	Systemic lupus erythematosus	Tnip1, Anxa6, Gpx3	Intron
**NHEK-M SE**				
**SNP**	**Location**	**Disease**	**Nearby genes**	**Location**
chr11	64135298	psoriasis	Rps6ka4, Mir1237	Intron
chr16	89986117	Basal cell carcinoma	Tubb3, Tcf25, Def8	Promoter
chr20	62328742	Atopic dermatitis	Arfrp1, Tnfrsf6b, Zgpat	Exon
chr11	589564	Systemic lupus erythematosus	Phrf1, Mir210hg, Irf7	Intron

### Basic enhancer structures are established in epidermal progenitors

Next we compared enhancers (TEs and SEs combined) in migrating and differentiating keratinocytes with the chromatin state at the same regions in the progenitors from which they derive. Approximately one third of active enhancers in NHEK-D or NHEK-M are also active enhancers in NHEK-P (Fig 2C). H3K4me1 alone marks another third, suggesting these regions, while not active in NHEK-P, are already marked for future activation during functional transitions. Less than 8 percent of enhancers derive from the poised state in progenitor cells, and less than 10 percent do not show any histone marks associated with enhancers in the progenitor state **(**Fig 2C). These results suggest that the underlying enhancer landscape is already established in progenitor keratinocytes.

We then compared the enhancer landscape between different cellular functional states with the changes that occur in lineage specification from embryonic stem cell (ESC) to NHEK-P. Only 8% of ESC SEs persist in NHEK-P; 17% are converted to TEs; 43% lose active enhancer marks, only retaining H3K4me1; and 32% lose all enhancer chromatin marks in NHEK-P (Fig 2D). In contrast, all SEs in NHEK-P retain some regulatory chromatin marks in NHEK-D or NHEK-M—the majority is converted to TEs in each case (Fig 2D)—indicating less changes in SE landscape than during keratinocytes lineage specification from ESCs. Surprisingly, TEs in ESC show even greater changes than SEs during the lineage specification from ESC to NHEK-P: more than 50% of TEs in ESCs lose all enhancer marks in NHEK-P (Fig 2D). In contrast, the majority of TEs in NHEK-P persist or are marked by some enhancer mark in NHEK-D or NHEK-M (Fig 2D). In addition, across SEs and TEs, we find greater overlap between NHEK-P and NHEK-D than between NHEK-P and NHEK-M (Fig 2D), suggesting that progenitors may be more epigenetically primed to differentiate than migrate. Together, these results show greater chromatin changes in the transition from ESC to progenitor keratinocyte than in the functional transitions within the keratinocyte lineage, and greater changes in TEs than SEs in the transition from ESC to progenitor keratinocytes.

### Fifty transcription factors regulate distinct epidermal differentiation stages

A large number of SEs in NHEK-D and NHEK-M overlap genes encoding transcription factors with important roles in promotion of epidermal differentiation, including *GRHL3*, *TP63*, *RUNX1*, *NOTCH3* and *FOS*. To test the role of these SE-associated transcription factors in a systematic manner, and to place GRHL3 in the context of other keratinocyte differentiation regulators, we used siRNAs to individually knock down *GRHL3* and 50 other transcriptional regulators in differentiating keratinocytes (S2 Table, S7 Fig). To assess the effect of the knockdowns on keratinocyte differentiation, we used custom-made Agilent microarrays to monitor the expression of approximately 14,000 genes, including all genes expressed in human keratinocytes and all transcriptional regulators (S1 Table). This 51 × 14,000 gene expression matrix provided a rich dataset to explore gene regulatory networks in epidermal differentiation.

**Fig 3:**
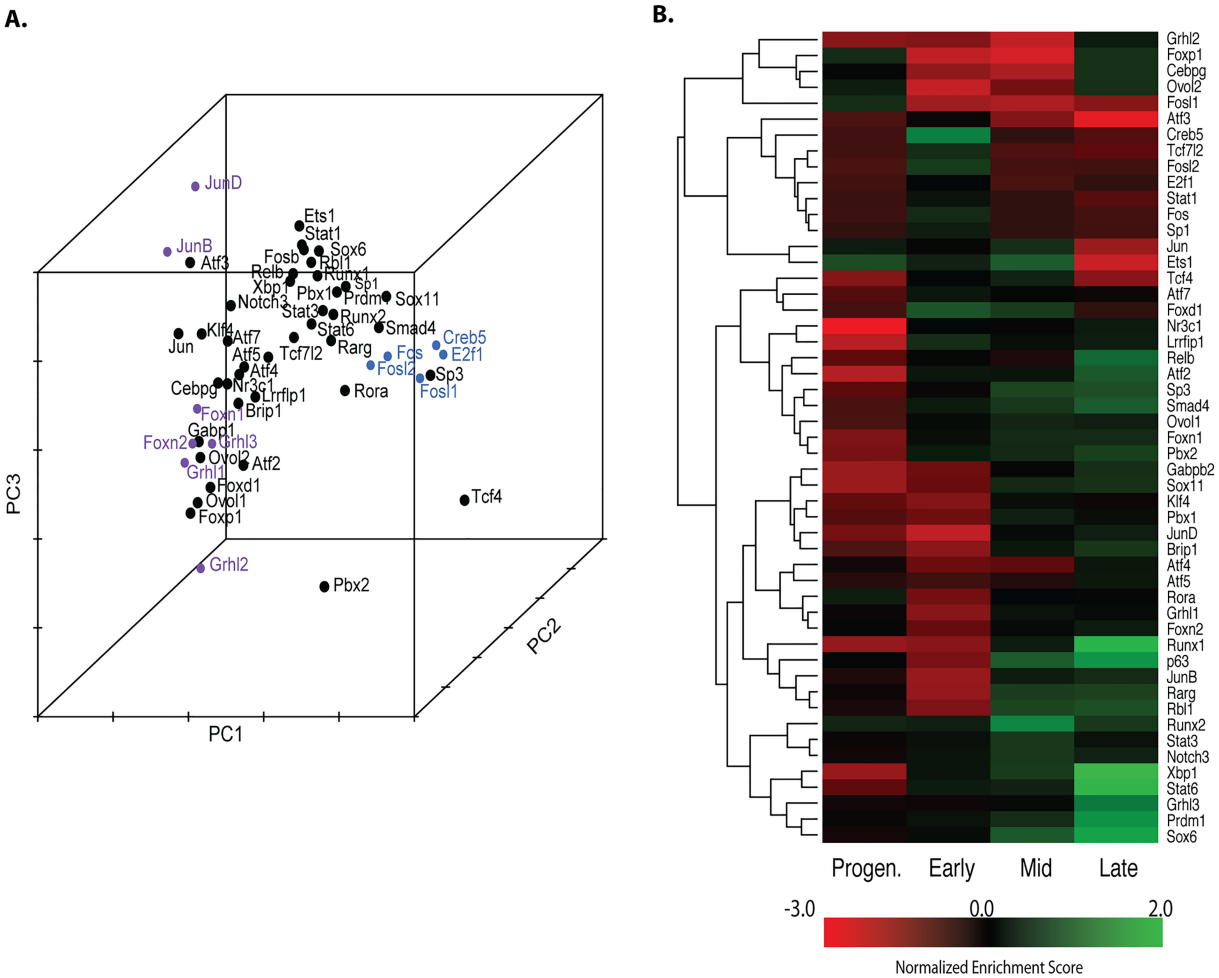
Many transcription factors regulate epidermal differentiation. (A) PCA analysis of siRNA screen, each dot represents gene expression in a single siRNA experiment, labeled with the name of the factor that was knocked down. Clusters of genes mentioned in the text are in color. (B) Clustering of 50 transcription factors based on GSEA enrichment scores for 4 distinct epidermal differentiation gene signatures (progenitor, early, mid, and late) after knockdowns.

Principal component (PC) analyses identified transcription factors that have similar effects on global gene expression (Fig 3A, S8 Fig). Gene expression profiles after knockdowns of *E2F1*, *SP1*, *CREB5* and *FOSL2* cluster together and away from other profiles, suggesting these factors regulate similar genes during epidermal differentiation. Gene expression profiles after the knockdowns of another group of transcription factors (including *JUNB*, *JUND*, *GRHL1*, *GRHL2*, *GRHL3*, *FOXN1* and *FOXN2*) cluster together at the other end of the primary axis (Fig 3A, S8 Fig), suggesting these factors have overlapping gene-regulatory functions distinct from the aforementioned group.

We used Gene Set Enrichment Analysis (GSEA) [28, 29] to more directly assess the role of each transcription factor in the regulation of distinct stages of epidermal differentiation; we calculated the enrichment of our 4 previously defined gene signatures characteristic of progenitor, early-, mid- or late-differentiation [26] among the genes affected by the knockdown of each transcriptional regulator, displaying the (inverse) enrichment score of each of the 4 signatures for each knockdown as a heat map (Fig 3B). The inverse enrichment score was used so that genes that are downregulated upon knockdown of the factor show positive enrichment scores, and those upregulated upon knockdown show negative enrichment scores. The majority of the transcriptional regulators most strongly affect mid- and late-differentiation genes. In contrast one factor, FOSL1, exclusively upregulates progenitor genes while downregulating genes in all three differentiation signatures. Combined with the finding that FOSL1 is upregulated in psoriatic epidermis [30], these results suggest that FOSL1 promotes keratinocyte proliferation. Also, a significant subset of factors, including CREB5, STAT1 and FOS, is associated with activation of early differentiation genes, suggesting these factors are early initiators of epidermal differentiation.

Similar to PRDM1 [31], GRHL3 is a selective activator of late-differentiation genes without significantly affecting the progenitor and earlier differentiation signatures. Other factors, however, show prominent regulatory duality during differentiation, repressing some signatures while activating others; the well-known pro-differentiation factor KLF4 [32, 33] represses progenitor and early-differentiation genes while activating mid-differentiation genes. Yet other factors that promote distinct stages of differentiation, including NR3C1 and RUNX1, repress progenitor genes, suggesting that they also have a dual role in repressing the progenitor state and promoting differentiation. Weighted correlation network analysis with R program WGCNA suggested coordinated regulation of modules of genes with distinct functions in epidermal differentiation, including a “transcription” module, and an “epidermal structure” module (S9 Fig).

### An SE-based transcriptional network regulates epidermal differentiation

**Fig 4:**
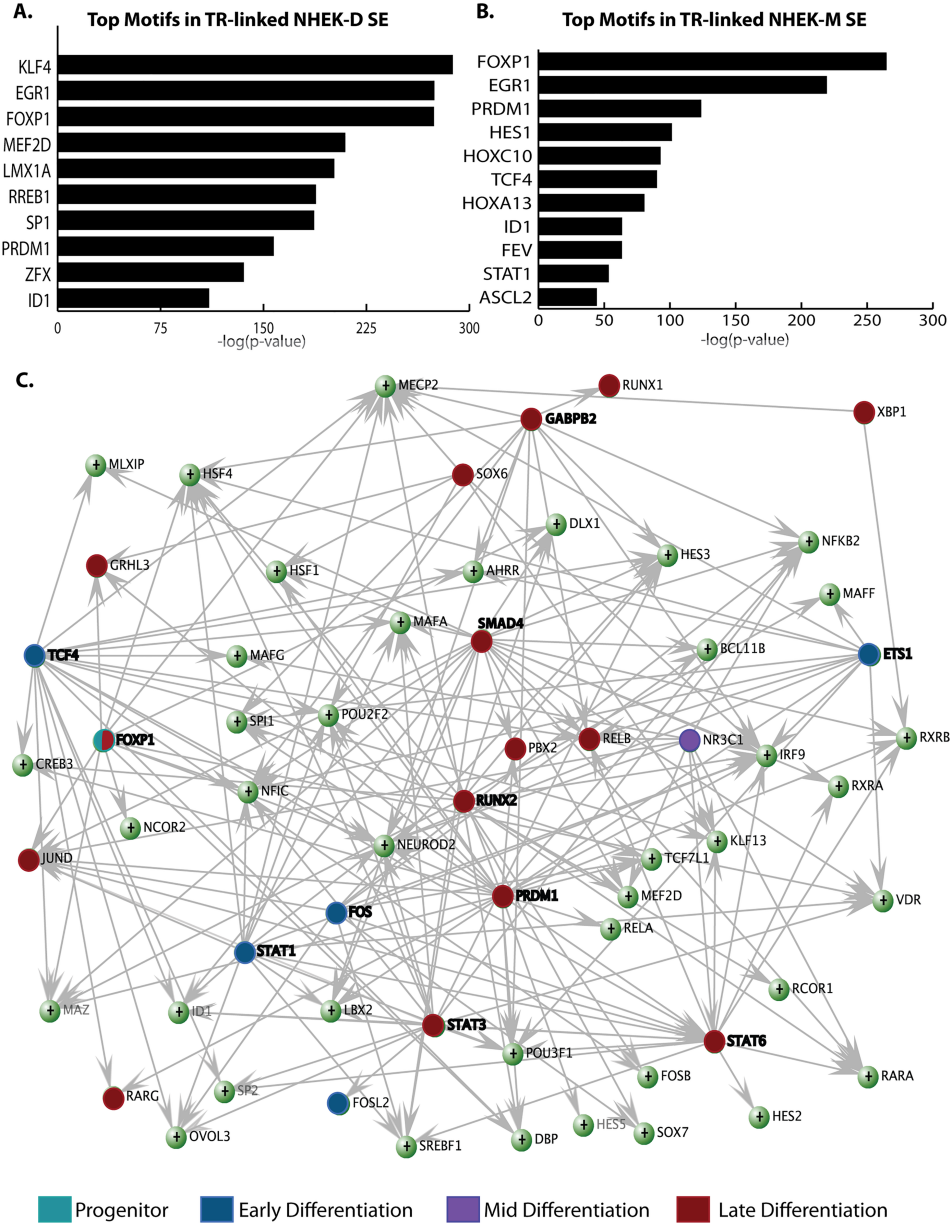
A reciprocal regulatory relationship between transcription factors and SEs. (A) Top enriched motifs for transcription factors differentially expressed during differentiation in SEs linked to transcriptional regulators (TR). (B) Top enriched motifs for transcription factors differentially expressed during migration in SEs linked to TRs. (C) Network level view of enriched motifs in the SEs linked to TRs; the transcription factor at base of arrows bind to the motif, the transcription factor gene at the point of the arrow contains the motif in its SE. Factors knocked down in the siRNA screen are in bold. Color coding refers to whether the factor is associated with a specific epidermal differentiation gene expression signature (progenitor, early, mid, or late differentiation) based on Fig 3B.

In addition to the 22 factors in the siRNA screen that are close to SEs, 208 other transcriptional regulators, including *ZNF750*, *KLF5*, *TCF3* and *ETS2* (S3 Table), are near SEs in NHEK-D. To understand which of these transcription factors bind NHEK-D and NHEK-M SEs, we searched for enrichment of the known motifs of all transcription factors differentially expressed during keratinocyte differentiation and migration in the nucleosome-free regions of SEs. In NHEK-D SEs there was significant enrichment of the motifs for a number of important epidermal transcription factors, including KLF4, PRDM1, ETS1, and XBP1 (Fig 4A, S4 Table), suggesting these factors are important regulators of SEs and thereby of key transcriptional programs activated in epidermal differentiation. NHEK-M SEs were enriched for similar motifs, but additionally featured motifs for HES1 and ASCL2; these factors may regulate migration-specific functions (Fig 4B, S4 Table).

To better understand the regulatory relationships between transcription factors and SEs, we focused on SEs overlapping genes encoding transcriptional regulators that are differentially expressed during epidermal differentiation [26]. Based on the transcription factor motifs enriched within these SE, we constructed a network describing SE regulation of transcription factors during epidermal differentiation. Consistent with the motif analysis (Fig 4A and 4B), the motifs for PRDM1, FOXP1, ETS1, and SMAD4 are enriched in the SEs linked to differentially expressed transcription factors; these factors form “hubs” in the network, acting on several epidermal differentiation transcriptional regulators (Fig 4C). Interestingly, two SNPs linked to skin diseases (rs3802826, psoriasis; and rs1128334, systemic lupus erythematosus) overlap “hub” gene *ETS1*, although both fall outside of the ETS1-linked SE domain. Examination of the regulatory directionality in the network revealed two classes of SE-linked transcription factors: those that act on other transcription factor genes, and those that receive regulation by other transcription factors. For example, PRDM1, ETS1, RUNX2, and SMAD4 bind to SEs to regulate the expression of other transcription factors, while the genes encoding *JUND*, *RELB*, and *IRF9* are recipients of regulation by other members of the network. We also noticed interesting regulatory differences within families of transcription factors: whereas STAT1 and STAT3 appeared to act primarily on other SEs, STAT6 both received input from other transcription factors and acted on other epidermal regulator genes.

We used the siRNA data (Fig 3) to validate the predicted network connections between transcription factors and their targets. Hence, a significant fraction of the transcription factors in SEs that are predicted to be regulated by PRDM1 based on motif analysis are indeed affected by the knockdown of *PRDM1* (S10 Fig). Other factors like FOXP1 and STAT6 also affect the expression of transcription factors whose SEs are enriched for their respective motifs.

We could also add a temporal layer to the network, color coding factors based on their regulatory characteristics in the siRNA screen (Fig 3B). With the exception of FOSL2, transcriptional regulators linked to early differentiation all regulate other SEs in the network. In contrast, many of the late differentiation transcriptional regulators primarily receive regulation from other members of the network (Fig 4C). Together, these results suggest that upon receiving the signal for differentiation, the early differentiation factors not only regulate early differentiation genes, but also bind to and activate SEs that control the mid and late differentiation transcriptional regulators.

### GRHL3 binds preferentially to SEs and shifts its chromatin binding locations between differentiation and migration

**Fig 5:**
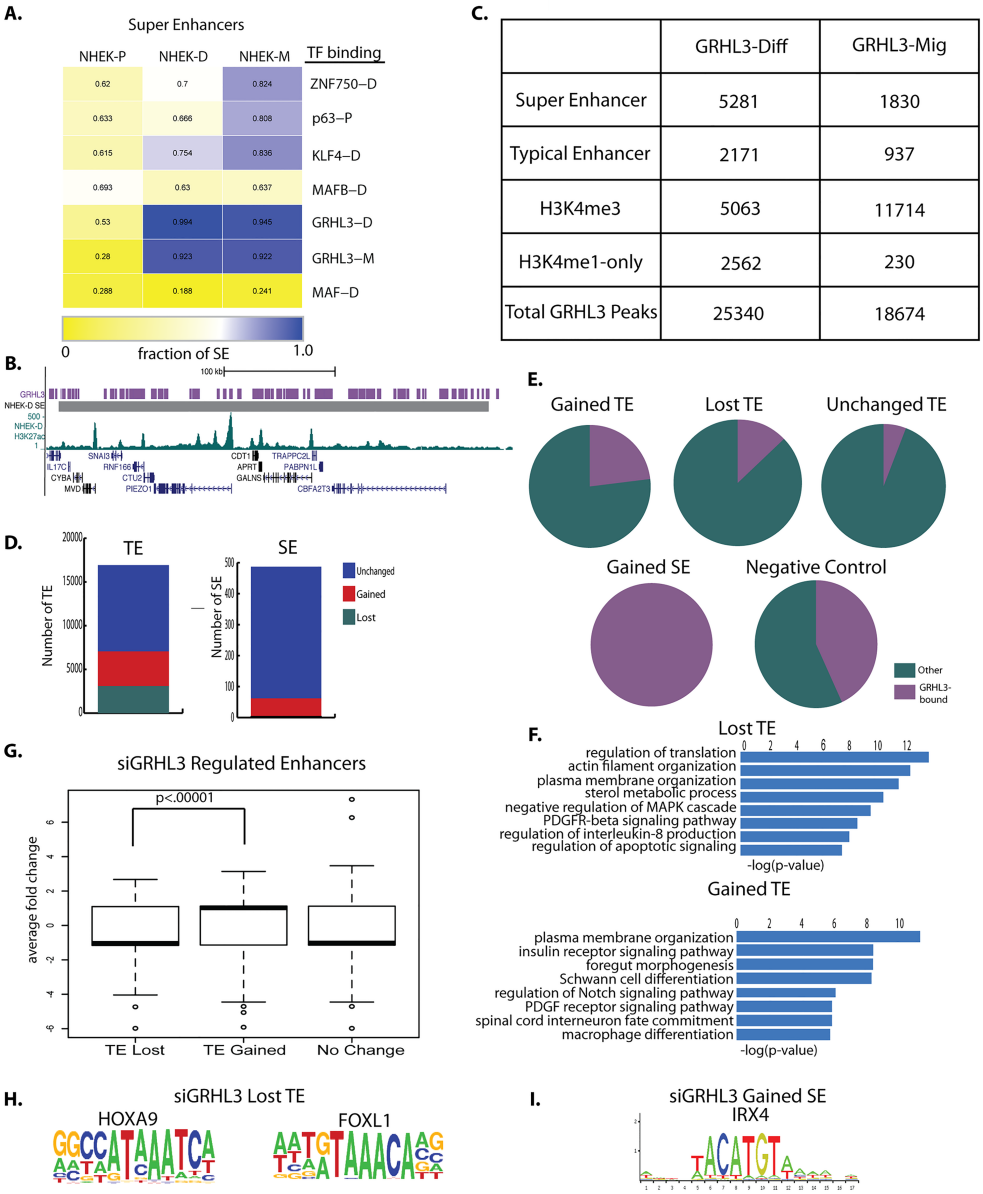
GRHL3 binds to super enhancers. (A) Fraction of SEs bound by epidermal transcription factors in progenitor (NHEK-P), differentiating (NHEK-D), and migrating (NHEK-M) cells. (B) Genomic view of high enrichment of GRHL3 binding in a NHEK-D SE. (C) Distribution of GRHL3 peaks in chromatin domains. (D) Fraction of NHEK-D TEs and SEs affected by siGRHL3. (E) Fraction of siGRHL3-affected TEs and SEs that are directly bound by GRHL3. (F) Gene ontology categories for TEs lost with GRHL3 knockdown, and TEs gained with GRHL3 knockdown. (G) Average expression fold-change of nearest genes to TEs lost, TEs gained, and TEs unchanged by siGRHL3. (H) Motifs enriched in TEs lost with GRHL3 knockdown. (I) Motif enriched in SEs gained with GRHL3 knockdown.

To examine further the transcription factors that regulate SEs in keratinocytes, we overlapped published NHEK ChIP-Seq data for epidermal regulators GRHL3, KLF4, MAFB, p63, and ZNF750 [2, 26, 34, 35] with NHEK SEs. Because GRHL3 is also known to regulate keratinocyte migration, we also generated new GRHL3 ChIP-Seq data in NHEK-M. Intriguingly, while KLF4, ZNF750, and MAFB bound to a significant fraction of SEs, GRHL3 bound to a larger proportion (98%) of SEs in NHEK-D than any other factor tested (Fig 5A). On average, there are 3 GRHL3 peaks in each of its target SEs, and approximately one third of SEs had greater than 5 GRHL3 peaks, further underscoring the important role of GRHL3 in keratinocyte gene regulation. GRHL3 binding also appears to be stronger in SEs than TEs; the average number of tags for GRHL3 peaks in SEs is 1.5 fold higher than the average number of tags for GRHL3 peaks in TEs. Consistent with binding specificity, GRHL3 chromatin binding sites in NHEK-D and NHEK-M showed significantly less overlap with regions corresponding to SEs in NHEK-P. A number of the SEs with the highest number of GRHL3 peaks overlap genes linked to psoriasis, like *IL17C* [36], providing further insight into the role of GRHL3 in psoriasis (Fig 5B, S5 Table**)** [17].

Consistent with its critical role in epidermal differentiation, the *GRHL3* gene is embedded within an SE in NHEK-D **(**S11A Fig); this fact and the fact that GRHL3 binds to the majority of SEs in NHEK-D and NHEK-M, makes GRHL3 suitable for studying the interaction between SEs and transcription factors in keratinocyte cell state transitions. The number of GRHL3 peaks is comparable in NHEK-D (approx. 25,000, one replicate) and NHEK-M (approx. 19,000, the overlap of two replicates) (Fig 5C). GRHL3 binding was more highly enriched in SEs than TEs for both functional states (Fig 5C). GRHL3 binding, however, was more highly enriched in regions marked by H3K4me3 in NHEK-M than NHEK-D, suggesting more prominent promoter binding in migrating than differentiating keratinocytes -- in contrast, GRHL3 binding is more highly enriched in enhancers in differentiating than migrating-keratinocytes (Fig 5C).

### GRHL3 suppresses the formation of non-keratinocyte SEs in differentiating keratinocytes

To determine whether GRHL3 acts to promote or repress enhancer formation, we knocked it down in differentiating keratinocytes, and then performed ChIP-seq for H3K4me1, H3K4me3, and H3K27ac in duplicate for both *GRHL3* knockdown cells and scramble control cells. Approximately 70% of H3K27ac peaks, 84% of H3K4me1 peaks, and 81% of H3K4me3 peaks overlapped between the two replicates for each condition; the overlapping enhancers between replicates were used for comparisons between siGRHL3 and scramble control. While over half of TEs are insensitive to GRHL3 levels, approximately 3,000 TEs are lost upon GRHL3 knockdown (Fig 5D). Intriguingly, 4,000 active TEs appear after GRHL3 knockdown (Fig 5D); only 10% of these new TEs are active enhancers in NHEK-P. The majority of TEs that are lost or gained upon GRHL3 knockdown contain the H3K4me1 mark in NHEK-P and NHEK-D, suggesting that GRHL3 activates or inhibits pre-defined regulatory regions (S11B Fig). Approximately 25% and 10% of TEs gained and lost, respectively, upon GRHL3 knockdown correspond to regions that are normally directly bound by GRHL3 in NHEK-D (Fig 5E). In contrast, only about 5% of TEs unchanged after GRHL3 knockdown are bound by GRHL3. Knockdown of *GRHL3* resulted in the loss of only 4 SEs, but 58 new SEs emerged (Fig 5D). All of the regions corresponding to these newly formed SEs after GRHL3 knockdown are normally bound by GRHL3, compared to approximately 40 percent of random regions of similar size, suggesting that binding of GRHL3 directly suppresses the formation of a subset of SEs (Fig 5E). While these newly formed SEs do not overlap SEs in either NHEK-P or NHEK-M, 89% overlap TEs in NHEK-D, and approximately half of them overlap TEs in NHEK-P, and the rest are marked with H3K4me1 signal. Of the four SEs lost upon siGRHL3, two are converted to TEs. It is intriguing that more TEs than SEs are affected by siGRHL3 even though GRHL3 binds to more SEs than TEs. One possible explanation is that GRHL3 primarily binds to SEs after they have been created by other factors earlier in the differentiation process, therefore, its binding and regulation at these sites may be secondary to their creation, and loss of GRHL3 may not interfere with the creation of the majority of SEs.

The TEs lost with siGRHL3 are near genes enriched for categories that include regulation of MAP kinase signaling and E-cadherin stabilization, while the TEs that are formed upon *GRHL3* knockdown link to genes with roles in neuronal commitment, axon projection, and regulation of foregut morphogenesis (Fig 5F). Many of the SEs formed upon GRHL3 knockdown also link to genes with roles in neuronal migration and axon guidance, including *UNC5A* and *NTNG2* (S6 Table). Fitting with the role of enhancers in promoting gene expression, there is a significant difference in the effect of siGRHL3 on genes near TEs gained compared to TEs lost: genes near TEs lost and unchanged in siGRHL3 cells are generally downregulated, whereas genes near TEs gained in siGRHL3 are upregulated by siGRHL3 (Fig 5G). There was no significant difference in expression levels of genes near SEs gained and SEs lost by siGRHL3, but this may be due to the small number of siGRHL3-affected SEs. Together, these results indicate that GRHL3 is required for the formation of a subset of SEs and TEs in differentiating keratinocytes. Intriguingly, GRHL3 also represses the formation of TEs and SEs near genes involved in the regulation of neuronal migration and the differentiation of non-epidermal cell lineages. Evidence of GRHL3 mediated regulation of genes with neuronal functions also comes from previous gene expression studies of developing epidermis in *Grhl3* knockout mice [15], where we find misregulation of a number of genes with roles in neuronal cells. It is interesting to note that all newly-formed SEs upon GRHL3 knockdown are marked with H3K4me3 and H3K27ac in some neuronal cell types [37], further supporting the idea that these are regulatory regions in non-epidermal cell types.

Motif analysis revealed that the TEs lost upon GRHL3 knockdown are enriched for HOXA9 and FOXL1 motifs (Fig 5H). SEs gained upon GRHL3 knockdown are strikingly enriched for the homeodomain factor IRX4 motif (Fig 5I), which is only weakly enriched above background in the full set of NHEK-D SEs.

### In keratinocyte migration, GRHL3 binds preferentially to promoters and regulates a gene set distinct from terminal differentiation

**Fig 6:**
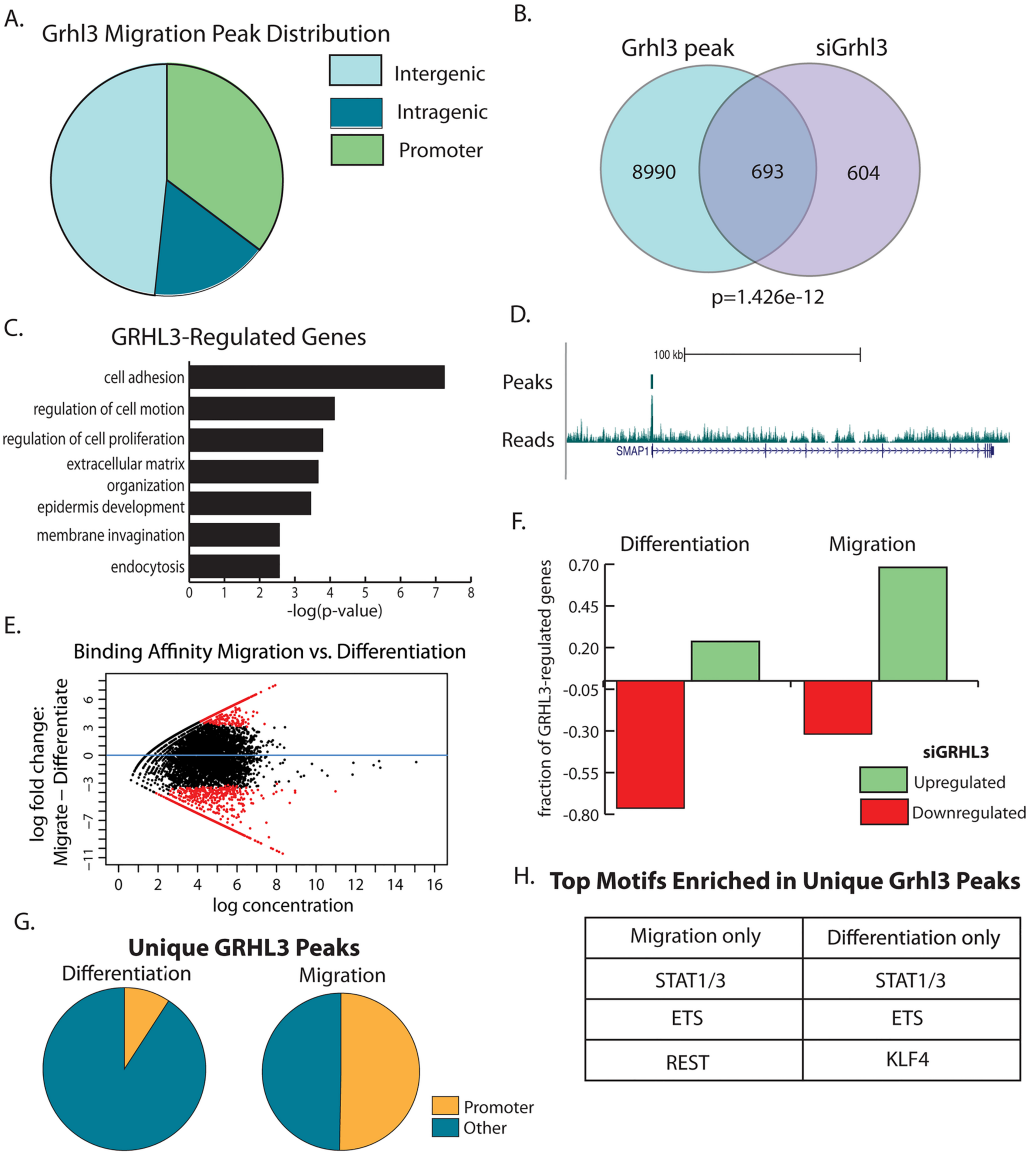
GRHL3 regulates genes through unique mechanisms in migrating and differentiating keratinocytes. (A) Distribution of GRHL3 peaks in NHEK-M across genomic features. (B) Overlap of genes with GRHL3 peaks and genes affected by siGRHL3. (C) Enriched gene ontology categories for overlapping genes in (B). (D) Genomic view of GRHL3 peak at the *Smap1* promoter. (E) DiffBind identification of significantly differentially bound GRHL3 sites in migration and differentiation. (F) Proportion of GRHL3 bound genes upregulated or downregulated by siGRHL3 in differentiation and migration. (G) Fraction of GRHL3-unique peaks in promoters in NHEK-D and NHEK-M. (H) Enriched motifs in unique migration or differentiation GRHL3 peaks.

Having characterized GRHL3-enhancer interactions in the progenitor to differentiation transition, we next examined GRHL3 binding in the progenitor to migration transition in more detail. Consistent with the high overlap with H3K4me3 regions (Fig 5C), further analysis of the GRHL3 migration ChIP-Seq data revealed that about 30 percent of peaks were in proximal promoters of annotated genes (Fig 6A). To identify direct targets of GRHL3 in NHEK-M, we used the GRHL3 ChIP-Seq peaks and global gene expression data after GRHL3 siRNA knockdown in NHEK-M, finding that about half of the genes whose expression was affected by the GRHL3 siRNA knockdown had a GRHL3 peak in their promoter (Fig 6B). These direct targets, which are enriched in gene ontology categories cell adhesion, epidermis development, and cell motion (Fig 6C), include *SMAP1* (Fig 6D), a small GTPase-activating protein that inhibits E-cadherin endocytosis. These target genes are distinct from those in differentiation.

### During keratinocyte migration, GRHL3 is primarily a repressor, acting on inhibitors of migration

Using the R program DiffBind [38], we found that GRHL3 binds 1,800 and 2,700 distinct genomic regions in NHEK-D and NHEK-M, respectively (Fig 6E). Consistent with the previous observation, the majority of direct GRHL3 targets in NHEK-D are downregulated when *GRHL3* is knocked down, and the majority of direct GRHL3 targets in NHEK-M are upregulated when *GRHL3* is knocked down (Fig 6F), supporting the idea that GRHL3 primarily acts as a transcriptional activator during differentiation and as a transcriptional repressor during migration. We also found that the nearest gene to each GRHL3-bound TE and SE in NHEK-M was more often upregulated by siGRHL3 rather than downregulated (S11D Fig).

As was suggested by the overlap of GRHL3 binding in NHEK-M and the histone modification H3K4me3, the majority of GRHL3 peaks unique to migration are found in the proximal 3kb promoter (2kb upstream through 1kb downstream), while the majority of peaks unique to differentiation are found outside the promoter (Fig 6G). Further analysis of the genes repressed by GRHL3 in migration identified *SEMA5A*, *TGFBR3*, *SMAP1* and a number of other genes that have been previously shown to act as inhibitors of migration in various different systems [39–44], suggesting GRHL3 may promote migration through direct inhibition of these genes.

### GRHL3 cooperates with REST to repress inhibitors of keratinocyte migration

We next performed *de novo* motif searches on the GRHL3 peaks unique to migration and differentiation, identifying enrichment of STAT and ETS motifs in both sets of unique peaks (Fig 6H). Additionally, the KLF4 motif was uniquely enriched in the differentiation peaks, consistent with KLF4’s known role in epidermal differentiation [34]. In the unique migration peaks, a motif matching the transcriptional repressor REST was significantly enriched (Fig 6H, S12 Fig). While a role for REST in epidermal keratinocyte migration has not been conclusively identified, the enrichment of the REST motif fits with the predicted role of GRHL3 in repressing gene expression during migration as REST functions as a repressive factor, recruiting HDACs to inhibit gene expression. Semaphorin 3a, an inhibitor of keratinocyte migration, is a known REST target in keratinocytes [45].

**Fig 7:**
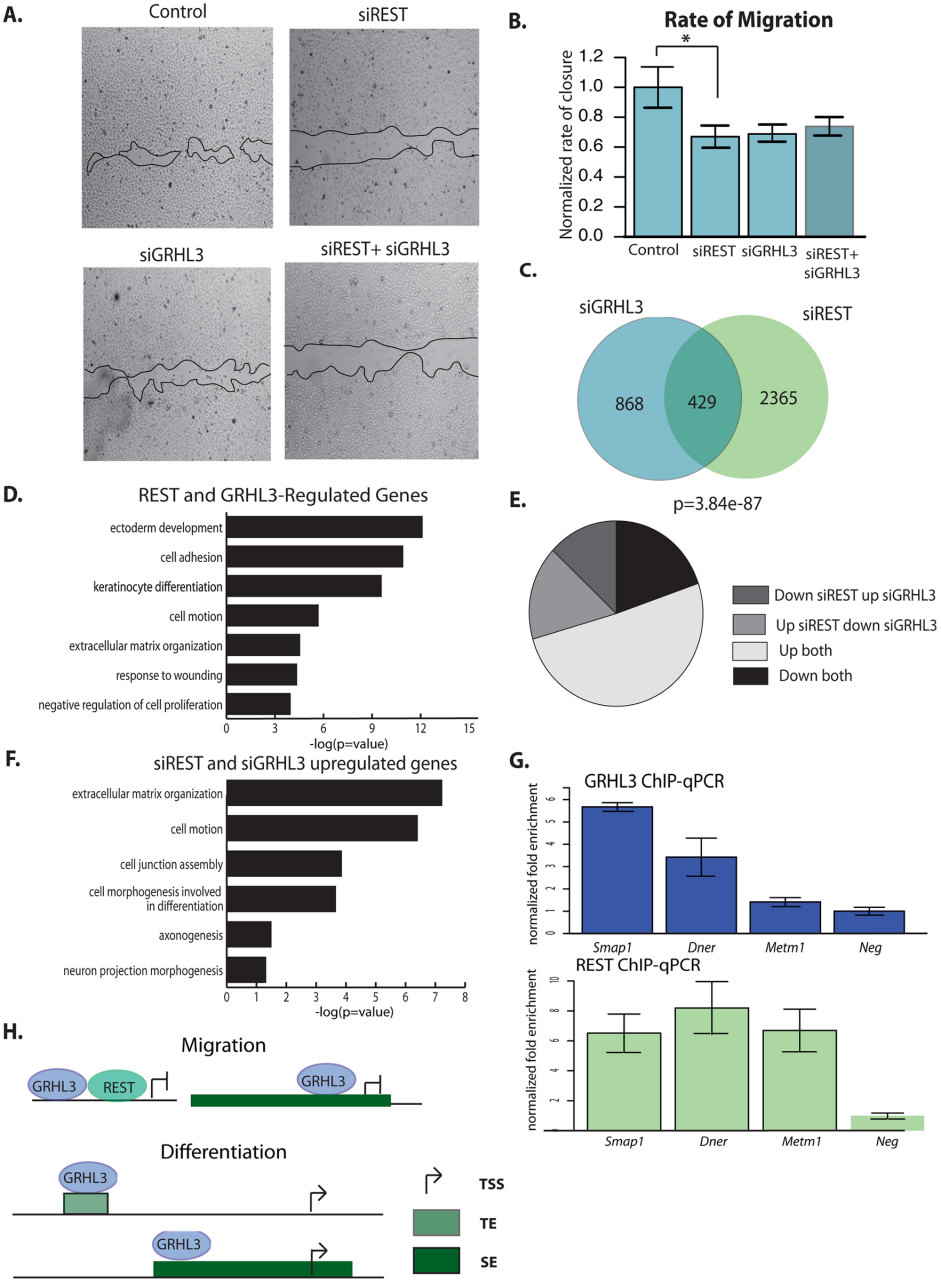
GRHL3 and REST regulate shared targets during keratinocyte migration. (A) Images of migration assays in siControl, siREST, siGRHL3, and combined siREST and siGRHL3 treated cells. (B) Quantification of the rate of migration in siControl, siREST, siGRHL3, and combined siREST+siGRHL3; n = 12 control, siREST; n = 10 siGRHL3, siGRHL3+siREST. (C) Overlap of genes affected (p<.05) by siGRHL3 and siREST in NHEK migration. (D) Enriched gene ontology terms for overlapping genes in (C). (E) Percentage of genes upregulated by both siGRHL3 and siREST (up both), downregulated by both siGRHL3 and siREST (down both), upregulated by siREST and downregulated by siGRHL3, or downregulated by siREST and upregulated by siGRHL3. (F) Enriched gene ontology terms for genes upregulated by both siREST and siGRHL3. (G) qPCR of GRHL3 or REST ChIP at several predicted shared targets. (H) Model for GRHL3-mediated gene regulation during epidermal keratinocyte migration and keratinocyte differentiation.

To test the effect of REST on keratinocyte migration we performed scratch assays after siRNA knockdown of REST. REST depleted cells closed the scratch at a slower rate than scramble control cells, indicating REST promotes keratinocyte migration (Fig 7A and 7B). We also tested the effect of depleting both REST and GRHL3 and found no additional decrease in rate of migration compared to either siRNA alone, suggesting REST and GRHL3 act on a similar set of targets to promote keratinocyte migration (Fig 7A and 7B).

Consistent with this idea, we observed a significant overlap of genes affected by REST and GRHL3 knockdowns (Fig 7C); these genes are involved in processes related to ectoderm development, cell adhesion, and cell motion (Fig 7D). The majority of these overlapping genes are upregulated by knockdown of *REST* or *GRHL3* (Fig 7E), further supporting the repressive co-regulatory role for these two factors suggested by the motif analysis of GRHL3 peaks. Many of the commonly regulated genes fall into cell migration and neuron projection categories, suggesting genes characterized as crucial components of neuronal migration during development also function in cell projection and movement during keratinocyte migration (Fig 7F). While we could not detect a direct interaction between GRHL3 and REST in Co-IP experiments, ChIP-qPCR experiments in NHEK-M validated that REST binds to GRHL3 target genes that contain a REST motif (Fig 7G).

In sum, our data highlights the reciprocal regulatory relationship between transcription factor GRHL3 and chromatin domains, including SEs—a regulation that applies not only to cell type specifications but also to migration, a reversible cell state. A transcription factor that regulates multiple transitions between keratinocyte states, like GRHL3, can do so through distinct mechanisms. In the transition from progenitor to differentiating keratinocyte, GRHL3 binds preferentially to SEs, and contributes to the formation of a small number of enhancers and the suppression of a larger number of non-keratinocyte and progenitor enhancers (Fig 7H). In contrast, in the transition between progenitor and migrating keratinocyte, GRHL3 primarily associates with promoters, where it represses transcription of genes inhibitory for migration in collaboration with the transcriptional repressor REST (Fig 7H).

## Discussion

### SEs regulate distinct functional states of committed cells

Compared to TEs, SEs are more directly linked to gene expression that confers cell type specification [1, 6]. In ESCs, where SEs have been most extensively studied, the genes encoding pluripotency factors are linked to SEs and binding sites for the pluripotency factors themselves are also enriched in SEs [1]. Analogous observations were made for the relationship between SEs and cell type specifying transcription factors in differentiated cells [4, 46, 47], and between SEs and oncogenes in transformed cells [48]. These findings, and the fact that common disease SNPs are overrepresented in SEs [6], point to SEs’ important regulatory functions. Our findings suggest that in addition to controlling cell type specification, SEs control migration, a transient functional state of lineage committed cells -- while a core group of SEs remains unchanged, keratinocytes activate unique subsets of SEs as they transition from progenitors to either differentiation or migration.

The SEs identified here have features previously described in other cell types, including the enrichment of disease-associated SNPs and higher expression of SE-associated genes than TE-associated genes. We have also identified new features of SEs in keratinocytes. First, in contrast to TEs, which frequently arise in the keratinocyte lineage from unmarked chromatin regions in ESCs, the majority of SEs in progenitor keratinocytes derive from chromatin regions already marked by H3K4me1 in ESCs, suggesting that SEs arise in more stable regulatory regions already marked for regulatory potential early in development. Second, GRHL3 knockdown leads to the appearance of novel SEs in differentiated keratinocytes, suggesting that GRHL3 represses non-keratinocyte SEs. A number of the new SEs formed after *GRHL3* knockdown are linked to neuronal gene expression, indicating that cell-type specific transcription factors can act to repress the formation of spurious SEs normally active in related cellular lineages. Interestingly, Polycomb factor CBX4 also represses neuronal cell gene expression in the epidermis [49], raising the possibility that GRHL3 could recruit the chromatin-modifying CBX4 to repress the formation of non-keratinocyte enhancers. Third, we found that keratinocyte SEs have propensity to locate at the edges of important epidermal gene clusters, including that of the EDC and keratin clusters, suggesting that SEs may promote loops between boundary enhancers and the promoters of genes within the cluster. In fact, locus control regions were some of the earliest identified enhancers and more recently have been shown to overlap SEs [6].

### Distinct networks of SE-linked transcription factors regulate progressive stages of epidermal differentiation

Our siRNA dataset provides a wealth of information about transcriptional regulation during the transition from progenitor to differentiated keratinocytes. By combining chromatin landscape features with this functional gene expression data, we identified a set of “hub” transcription factors, including ETS1, SMAD4, RUNX2, and PRDM1, that are close to SEs and whose motifs are enriched within many of the SEs linked to other epidermal transcription factor genes. Each of these factors shows a distinct pattern of association with the temporal gene signatures of epidermal differentiation. For example, PRDM1 shows little correlation with the progenitor signature, but an increasingly positive correlation across early, mid, and late differentiation. Other factors are primarily linked to progenitor and early gene expression, including FOSL1, CREB5, and STAT1. Our findings suggest that “hub” transcription factors may bind to and regulate SEs at specific time points during differentiation, ensuring the correct sequence of events for a successful transition from a progenitor to a differentiating cell. Interestingly, we find that two SNPs linked to skin diseases overlap the ETS1 gene, although both fall outside of the ETS1-linked SE. Since ETS1 is one of the “hub” transcription factors identified by the network analysis as acting early in differentiation to regulate other epidermal transcriptional regulators, even mild disruption of ETS1 expression or function could have pathological downstream effects on epidermal differentiation in diseases like psoriasis [17].

The data also show several examples where different members of transcription factor families, including the AP1, GRHL, OVOL, and SOX families, affect distinct stages of epidermal differentiation, which is consistent with the distinct epidermal phenotypes when the genes encoding different family members are deleted in mice [15, 26, 50–52].

### Large scale shifts in chromatin binding and co-factor associations underlie GRHL3’s ability to regulate both keratinocyte differentiation and migration

Our data on dual roles of GRHL3 in the transitions from progenitors to migration or differentiation increase our understanding of how a single transcription factor can perform unique regulatory functions in two cell states. The GRHL3-REST [53] co-regulation in migrating keratinocytes is a novel finding, and while targets of REST have been shown to have a role in keratinocyte migration [45], our study is the first to identify a direct regulatory role for REST in keratinocyte migration and to identify shared REST and GRHL3 gene targets. We were unable to detect a direct interaction between REST and GRHL3, suggesting that the GRHL3-REST interaction is chromatin dependent. Many of the shared targets of REST and GRHL3, the majority of which are repressed by the two factors, are inhibitors of cell migration and of axon projection in neurons. Consistent with our finding for GRHL3 and REST in repressing inhibitors of migration, including genes regulating cell projections, GRHL3 has been shown to act in leading edge cells during migration, promoting actin polymerization and filopodia projections [19]. In sum, GRHL3 and REST regulate a group of shared genes involved in neuronal and keratinocyte migration and development.

## Conclusions

While previous studies have linked SEs to cell fate and differentiation-associated gene expression [1, 4, 6], we characterized the gene-regulatory landscape in a reversible functional state, migration, demonstrating extensive rearrangements of SEs as progenitor keratinocytes either differentiate or migrate. We also uncovered a novel role for GRHL3 in regulating the formation of enhancers, most strikingly in the suppression of non-keratinocyte SEs in differentiating keratinocytes. SNPs associated with skin diseases are overrepresented in SEs and the genes encoding many key transcriptional regulators of epidermal differentiation, including that of *GRHL3*, are located within SEs. In turn these transcription factors act on other SEs, suggesting the existence of SE-based feed-forward loops for driving high gene expression that stabilizes functional states. Genome-wide binding and gene-regulatory activity of GRHL3, is distinct in differentiating keratinocytes, where it primarily binds SEs and activates transcription, and in migrating keratinocytes, where it primarily binds proximal promoters and represses transcription (Fig 7H). Hence, the unique binding profiles of transcription factors like GRHL3 under different keratinocyte functional states allow for multiple transcriptional outputs from the same factor to meet the unique physiological needs of each functional state. Together, our data indicate that regulation of keratinocyte function involves complex and reciprocal interaction of the chromatin landscape with cohorts of transcription factors acting at specific time points during transition processes.

## Materials and methods

### Cell culture

Normal Human Epidermal Keratinocytes (NHEK) were purchased from LifeLine Technologies and grown according to the manufacturer’s instructions in DermaLife medium (LifeLine Tech) supplemented with DermaLife growth factors (LifeLine Tech). High calcium medium (1.8mM Ca2+) was used to induce differentiation. To induce migration, cells were grown to a confluent monolayer and scratched with a p200 pipette tip. Scratches were made approximately every 1 cm horizontally and vertically, resulting in a grid of scratches. While only the proportion of cells at the edges of the scratch are induced to migrate, significant and substantial gene expression and chromatin changes are observed in the mixed population.

### Transfection

Lipofectamine RNAi Max (Life Technologies) in OptiMEM medium was used for transfections of GRHL3 and REST siRNA: 30nM pooled siRNA was used for knockdown each of *GRHL3* (Dharmacon L-014017-02), *REST* (Qiagen hs_REST_5: S104153765, hs_REST_1: s100701407), and scramble control (Dharmacon D-001810-10-05). Experiments were performed 72 hours after transfection.

### Migration assays

72 hours after transfection, cells were incubated for 2 hours with 3.5ug mitomyosin C. Scrapes were made with a pipette tip and medium was changed. Images were taken immediately (0 hour), and 12, 14, 16, 20, 24 and 36 hours after scratching. The area of the scratches was measured by Image J.

### Chromatin immunoprecipitation assays

ChIP assays were performed as previously described [54] with the following changes: 28ug of sonicated chromatin was used for each IP, magnetic Dynabeads (Invitrogen) were used for immunoprecipitation and, for ChIP-qPCR analysis, enrichment was calculated over IgG and normalized to an intergenic negative control region. The following antibodies were used: IgG (Sigma), H3K4me1 (AbCam), H3K27ac (Milipore), H3K4me3 (Milipore), GRHL3 (Andersen Lab), and REST (Millipore 17–641). Primers for ChIP-qPCR are listed in S8 Table.

### ChIP-Seq library preparation

Sequencing libraries were generated for the GRHL3, H3K4me1, H3K4me3, H3K27ac, and Input samples using the NEB Next reagents, and Illumina adaptors and oligos, according to the Illumina protocol for ChIP-Seq library prep, with the following modifications. Following the protocol by Schmidt et. al., after adaptor ligation, PCR amplification was performed prior to size selection of the library [55]. Clustering and 50-cycle single end sequencing were performed on the Illumina Hi-Seq 2000 Genome Analyzer.

### siRNA screen

Using a previously published keratinocyte differentiation timecourse microarray [26], we identified a list of significantly differentially expressed transcriptional regulators during early differentiation (0–24 hours). Each transcriptional regulator selected was required to have a well-defined DNA-binding motif either in the JASPAR database, or in the literature. Literature searches further helped to narrow the list to transcriptional regulators predicted to be important in epidermal differentiation. 50 transcription factors with known or suspected roles in epidermal differentiation were selected for siRNA knockdown; the genes encoding 22 of them are associated with SEs (S2 Table). While many of the selected factors overlapped SEs in keratinocytes, we also included a number of well-characterized epidermal regulators that do not overlap SEs to allow the incorporation of network information about the relation of these well studied factors to other transcriptional regulators and SEs during keratinocyte differentiation. Pooled siRNAs for each factor (Dharmacon, S7 Table) were used for knockdown in NHEK-P. 24 hours after knockdown, cells were induced to differentiate by the addition of high calcium media, and collected 24 hours later. RNA was extracted and run on custom designed arrays (Agilent, S1 Table). Each factor was knocked down as a single replicate, and four samples of scramble control siRNA were also included for comparison. Differential gene expression was determined by CyberT [56].

## Bioinformatics analysis

### siRNA screen

R was used for PCA analysis and hierarchical clustering of the data. Gene Enrichment Analysis was performed using GSEA [28, 29]. Weighted Gene Correlation Analysis (WGCNA) was used to develop network level descriptions of the siRNA data, and to link factors to the genes they are predicted to regulate. Analysis was limited to genes that showed differential expression across epidermal differentiation. To determine the potential regulatory links between transcription factors, a second, separate WGCNA analysis was performed on all transcriptional regulators that are expressed in epidermal keratinocytes and present on the array.

#### ChIP-Seq

Sequencing reads were aligned using Bowtie [57], and only uniquely aligned reads were retained. For GRHL3, peaks were called using MACS. The two NHEK-M replicates clustered together, and away from GRHL3 peaks in NHEK-D. For histone modifications, peaks were called using SICER [58]; typical enhancers were defined as regions with overlapping H3K27ac and H3K4me1 peaks, with low levels of H3K4me3 (no peak, or peaks scoring less than 5 fold enrichment above input control). The similarity of replicates was assessed by calculating Pearson correlation coefficients for two NHEK-D replicates generated in this experiment, and three NHEK-P replicates from ENCODE (S13 Fig). There are stronger correlations between replicates, suggesting the data is reproducible, and that comparisons between the NHEK-P and NHEK-D reveal true differences between cells in each condition. Super enhancers were called using ROSE [6, 48] with the H3K27ac ChIP data; SEs were subtracted from the previously identified TEs. BedTools (closestBed, intersectBed, subtract) were used to analyze overlapping regulatory regions between NHEK-P, NHEK-M and NHEK-D, and to identify the nearest genes. GREAT [59] and DAVID [60] were used for gene ontology analysis. Galaxy [61, 62] software was used for further analysis.

#### Motif analysis

We identified nucleosome free regions in SEs and TEs with Homer’s –nfr option [63]. We then used Homer and MEME [64] to identify enriched motifs *de novo* within these nfr regions. To identify epidermal regulators of SE, the motifs for all differentially expressed transcription factors during epidermal differentiation were tested for enrichment in NHEK-D SEs using AME, a package within the MEME suite of programs. This analysis was repeated with NHEK-M SEs for all differentially expressed transcription factors during keratinocyte migration. NHEK-D SEs were then scanned with the motifs that showed significant enrichment above background, and the results were used to build a network describing SE regulation of transcription factors during epidermal differentiation. visANT [65] was used to visualize the network. The predictions of this motif-based SE network were tested with the siRNA screen data for applicable factors.

#### Epidermal disease SNP analysis

Association results for complex skin diseases were retrieved from the NHGRI catalog and Immunobase. Only SNPs achieving genome-wide association (*p* ≤ 5x10-8) were included. When there were multiple SNPs for a disease within a locus (within +-500kb), only the most significant variant was used for the analysis. The resulting list of 160 SNPs was overlapped with SEs and TEs in NHEK-P, NHEK-D, and NHEK-M. As a control, for each condition, random regions of the same size were overlapped with the SNP list, showing significantly lower levels of overlap.

### Quantitative real time PCR

For mRNA expression analysis, cDNA was prepared using iScript cDNA kit and RT-PCR was performed using SsoFast for Probes and SsoFast EvaGreen (Biorad Laboratories) master mixes in CFX384 Real-Time PCR Detection System (Biorad Laboratories). GAPDH or RPLPO were used as endogenous controls.

### RNA extractions

Cells were collected and lysed in Trizol, followed purification with Zymo RNA extraction kit. RNA concentration and quality were quantified on a NanoDrop.

### Affymetrix microarray analysis

Gene expression analysis for NHEK cells was performed with biological duplicates, Affymetrix Human Gene 1.0 ST arrays (26,869 probe sets) were used and washed according to manufacturer's recommendations (Affymetrix, Santa Clara, CA). Plier analysis was performed, and the data were then filtered for expression levels. Probes with raw expression values below 200 were considered not expressed for subsequent analysis.

#### Ethics approval and consent to participate

Not applicable.

#### Availability of data and materials

The datasets generated and analyzed during the current study are available in the GEO repository: GSE68257, GSE68075, GSE76691, GSE86193, GSE94465, GSE94466, GSE94467, GSE94471

## Supporting information

S1 Fig (A) Plot of H3K27ac signal intensity and length for SEs and TEs in NHEK-P. (B) Comparison of the distance from TE or SE to the nearest gene. (C) Overlap of SEs with housekeeping genes in each cell state. (D) ChIP-qPCR of MED1 binding to SE (2 primer sets for each of 4 SE tested) and TE (one primer set for each of 3 TE tested). Neg = negative control, EDC = epidermal differentiation complex, TE = typical enhancer, n = 3.
(TIF)

S2 Fig (A) Gene ontology analysis for genes near TE shared between NHEK-P, NHEK-M, and NHEK-D. (B) Gene ontology analysis for genes near SEs.
(TIF)

S3 Fig Plot of the H3K27ac, H3K4me3, and H3K4me1 signal and called SE and TE peaks at the Epidermal Differentiation Complex (EDC) in NHEK-D, NHEK-P, and NHEK-M. (TIF)

S4 Fig SEs are located at the edges of key epidermal gene clusters. (A) SEs at the keratin gene cluster on chromosome 17. (B) SEs at the keratin gene cluster on chromosome 12. C) SEs at the HOXA gene locus.
(TIF)

S5 Fig Gene ontology analysis for genes near TEs unique to each cell state. (TIF)

S6 Fig H3K27ac signal at skin disease-linked SNPs. (TIF)

S7 Fig Validation of siRNA knockdown of 50 transcription factors prior to array hybridization. (TIF)

S8 Fig PCA analysis of siRNA screen, each dot represents gene expression after a single siRNA experiment, labeled with the factor name. (TIF)

S9 Fig Epidermal differentiation genes cluster into distinct modules based on correlated expression patterns and functional roles. Gene ontology of top identified co-expression modules and list of selected transcription factors.
(TIF)

S10 Fig Effect of knockdown of PRDM1 (A), FOXP1 (B), or STAT6 (C) on their predicted targets based on motif-based network analysis. Predicted gene targets that did not validate based on the siRNA experiment are presented in grey.
(TIF)

S11 Fig GRHL3 interacts with TEs and SEs. (A) An SE overlapping the *Grhl3* gene body in NHEK-D and NHEK-P. (B) Overlap of H3K4me1 histone modification with TEs and SEs gained or lost by siGRHL3. Overlap of GRHL3 binding with SEs gained or lost by siGRHL3. (C) Effect of GRHL3 knockdown on expression of nearest gene to SEs lost, SEs gained, and SEs unchanged. (D) Effect of GRHL3 knockdown on expression of nearest gene to GRHL3 bound TE, and to GRHL3 bound, non-promoter SE.
(TIF)

S12 Fig REST and GRHL3 bind to shared targets. (A) REST motif and enrichment score for GRHL3 peaks unique to migration.
(TIF)

S13 Fig Pearson correlation coefficients of comparisons between two NHEK-D replicates (scramble control samples) and 3 NHEK-P replicates (ENCODE data) for H3K4me1 and H2K27ac. (TIF)

S1 Table List of probe names, gene symbols, and accession numbers for custom array. (PDF)

S2 Table List of transcription factors selected for siRNA and their overlap with SEs in NHEK-D or NHEK-M (indicated by “*”). (PDF)

S3 Table List of all transcriptional regulators that overlap NHEK-D SEs. (PDF)

S4 Table Motif enrichment in NHEK-D or NHEK-M SEs for transcription factors differentially expressed in keratinocyte differentiation or migration, respectively. (PDF)

S5 Table Top SEs with greatest number of GRHL3 binding peaks and the psoriasis related genes they overlap. (PDF)

S6 Table Nearest gene to SEs gained when GRHL3 is knocked down in NHEK-D. (PDF)

S7 Table Dharmacon catalog numbers for siRNA used in screen. (PDF)

S8 Table Primers for ChIP-qPCR of REST in Fig 7 and MED1 in S1D Fig. (PDF)

## References

[1] Whyte WA, Orlando DA, Hnisz D, Abraham BJ, Lin CY, Kagey MH, et al Master Transcription Factors and Mediator Establish Super-Enhancers at Key Cell Identity Genes. Cell. 2013;153(2):307–19. 10.1016/j.cell.2013.03.035 23582322 PMC3653129

[2] Kouwenhoven EN, Oti M, Niehues H, van Heeringen SJ, Schalkwijk J, Stunnenberg HG, et al Transcription factor p63 bookmarks and regulates dynamic enhancers during epidermal differentiation. EMBO reports. 2015;16(7):863–78. Epub 06/03. Epub 2015 Jun 1. 10.15252/embr.201439941 26034101 PMC4515125

[3] Cavazza A, Miccio A, Romano O, Petiti L, Malagoli Tagliazucchi G, Peano C, et al Dynamic Transcriptional and Epigenetic Regulation of Human Epidermal Keratinocyte Differentiation. Stem cell reports. 2016;6(4):618–32. Epub 04/07. Epub 2016 Mar 31. 10.1016/j.stemcr.2016.03.003 27050947 PMC4834057

[4] Adam RC, Yang H, Rockowitz S, Larsen SB, Nikolova M, Oristian DS, et al Pioneer factors govern super-enhancer dynamics in stem cell plasticity and lineage choice. Nature. 2015;521(7552):366–70. Epub 03/25. Epub 2015 Mar 18. 10.1038/nature14289 25799994 PMC4482136

[5] Sha K, Boyer LA. The chromatin signature of pluripotent cells StemBook. Cambridge (MA): Harvard Stem Cell Institute Copyright: (c) 2009 Ky Sha and Laurie Boyer; 2008. 20614601

[6] Hnisz D, Abraham BJ, Lee TI, Lau A, Saint-Andre V, Sigova AA, et al Super-enhancers in the control of cell identity and disease. Cell. 2013;155(4):934–47. Epub 10/15. Epub 2013 Oct 10. 10.1016/j.cell.2013.09.053 24119843 PMC3841062

[7] Pott S, Lieb JD. What are super-enhancers? Nat Genet. 2015;47(1):8–12. Epub 2014/12/31. 10.1038/ng.3167 25547603

[8] Tao H, Berno AJ, Cox DR, Frazer KA. In Vitro Human Keratinocyte Migration Rates Are Associated with SNPs in the KRT1 Interval. PLoS ONE. 2007;2(8):e697 10.1371/journal.pone.0000697 17668073 PMC1933256

[9] Liu M, Saeki K, Matsunobu T, Okuno T, Koga T, Sugimoto Y, et al 12-hydroxyheptadecatrienoic acid promotes epidermal wound healing by accelerating keratinocyte migration via the BLT2 receptor. The Journal of Experimental Medicine. 2014;211(6):1063–78. 10.1084/jem.20132063 24821912 PMC4042643

[10] Meyer M, Muller AK, Yang J, Moik D, Ponzio G, Ornitz DM, et al FGF receptors 1 and 2 are key regulators of keratinocyte migration in vitro and in wounded skin. Journal of cell science. 2012;125(Pt 23):5690–701. Epub 09/21. Epub 2012 Sep 19. 10.1242/jcs.108167 22992463 PMC3575704

[11] Boyce ST, Ham RG. Calcium-regulated differentiation of normal human epidermal keratinocytes in chemically defined clonal culture and serum-free serial culture. J Invest Dermatol. 1983;81(1 Suppl):33s–40s. Epub 07/01. 6345690 10.1111/1523-1747.ep12540422

[12] Borowiec AS, Delcourt P, Dewailly E, Bidaux G. Optimal differentiation of in vitro keratinocytes requires multifactorial external control. PLoS One. 2013;8(10):e77507 Epub 10/12. 10.1371/journal.pone.0077507 24116231 PMC3792032

[13] Fuchs E, Raghavan S. Getting under the skin of epidermal morphogenesis. Nature reviews Genetics. 3 England 2002 p. 199–209. 10.1038/nrg758 11972157

[14] Shaw TJ, Martin P. Wound repair at a glance. Journal of cell science. 122 England 2009 p. 3209–13. 10.1242/jcs.031187 19726630 PMC2736861

[15] Yu Z, Lin KK, Bhandari A, Spencer JA, Xu X, Wang N, et al The Grainyhead-like epithelial transactivator Get-1/Grhl3 regulates epidermal terminal differentiation and interacts functionally with LMO4. Developmental biology. 299 United States 2006 p. 122–36. 10.1016/j.ydbio.2006.07.015 16949565

[16] Ting SB, Caddy J, Hislop N, Wilanowski T, Auden A, Zhao LL, et al A homolog of Drosophila grainy head is essential for epidermal integrity in mice. Science (New York, NY). 308 United States 2005 p. 411–3. 10.1126/science.1107511 15831758

[17] Gordon WM, Zeller MD, Klein RH, Swindell WR, Ho H, Espetia F, et al A GRHL3-regulated repair pathway suppresses immune-mediated epidermal hyperplasia. The Journal of clinical investigation. 2014;124(12):5205–18. Epub 10/28. Epub 2014 Oct 27. 10.1172/JCI77138 25347468 PMC4348962

[18] Caddy J, Wilanowski T, Darido C, Dworkin S, Ting SB, Zhao Q, et al Epidermal wound repair is regulated by the planar cell polarity signaling pathway. Developmental cell. 19 United States: 2010 Elsevier Inc; 2010 p. 138–47. 10.1016/j.devcel.2010.06.008 20643356 PMC2965174

[19] Yu Z, Bhandari A, Mannik J, Pham T, Xu X, Andersen B. Grainyhead-like factor Get1/Grhl3 regulates formation of the epidermal leading edge during eyelid closure. Developmental biology. 319 United States 2008 p. 56–67. 10.1016/j.ydbio.2008.04.001 18485343 PMC2494567

[20] Hislop NR, Caddy J, Ting SB, Auden A, Vasudevan S, King SL, et al Grhl3 and Lmo4 play coordinate roles in epidermal migration. Developmental biology. 2008;321(1):263–72. Epub 07/16. Epub 2008 Jun 26. 10.1016/j.ydbio.2008.06.026 18619436

[21] Erokhin M, Vassetzky Y, Georgiev P, Chetverina D. Eukaryotic enhancers: common features, regulation, and participation in diseases. Cellular and molecular life sciences: CMLS. 2015;72(12):2361–75. Epub 02/27. Epub 2015 Feb 26. 10.1007/s00018-015-1871-9 25715743 PMC11114076

[22] Pombo A, Dillon N. Three-dimensional genome architecture: players and mechanisms. Nature reviews Molecular cell biology. 2015;16(4):245–57. Epub 03/12. Epub 2015 Mar 11. 10.1038/nrm3965 25757416

[23] Rada-Iglesias A, Bajpai R, Swigut T, Brugmann SA, Flynn RA, Wysocka J. A unique chromatin signature uncovers early developmental enhancers in humans. Nature. 470 England 2011 p. 279–83. 10.1038/nature09692 21160473 PMC4445674

[24] Heintzman ND, Stuart RK, Hon G, Fu Y, Ching CW, Hawkins RD, et al Distinct and predictive chromatin signatures of transcriptional promoters and enhancers in the human genome. Nature genetics. 2007;39(3):311–8. Epub 02/06. 10.1038/ng1966 17277777

[25] Rinaldi L, Datta D, Serrat J, Morey L, Solanas G, Avgustinova A, et al Dnmt3a and Dnmt3b Associate with Enhancers to Regulate Human Epidermal Stem Cell Homeostasis. Cell stem cell. 2016;19(4):491–501. Epub 2016/08/02. 10.1016/j.stem.2016.06.020 27476967

[26] Hopkin AS, Gordon W, Klein RH, Espitia F, Daily K, Zeller M, et al GRHL3/GET1 and Trithorax Group Members Collaborate to Activate the Epidermal Progenitor Differentiation Program. PLoS genetics. 8 United States 2012 p. e1002829 10.1371/journal.pgen.1002829 22829784 PMC3400561

[27] An integrated encyclopedia of DNA elements in the human genome. Nature. 2012;489(7414):57–74. Epub 09/08. 10.1038/nature11247 22955616 PMC3439153

[28] Subramanian A, Tamayo P, Mootha VK, Mukherjee S, Ebert BL, Gillette MA, et al Gene set enrichment analysis: a knowledge-based approach for interpreting genome-wide expression profiles. Proceedings of the National Academy of Sciences of the United States of America. 2005;102(43):15545–50. Epub 10/04. 10.1073/pnas.0506580102 16199517 PMC1239896

[29] Mootha VK, Lindgren CM, Eriksson KF, Subramanian A, Sihag S, Lehar J, et al PGC-1alpha-responsive genes involved in oxidative phosphorylation are coordinately downregulated in human diabetes. Nature genetics. 2003;34(3):267–73. Epub 06/17. 10.1038/ng1180 12808457

[30] Sobolev VV, Zolotorenko AD, Soboleva AG, Elkin AM, Il'ina SA, Serov DN, et al Effects of expression of transcriptional factor AP-1 FOSL1 gene on psoriatic process. Bull Exp Biol Med. 2011;150(5):632–4. Epub 2012/01/12. 22235402 10.1007/s10517-011-1208-0

[31] Magnusdottir E, Kalachikov S, Mizukoshi K, Savitsky D, Ishida-Yamamoto A, Panteleyev AA, et al Epidermal terminal differentiation depends on B lymphocyte-induced maturation protein-1. Proceedings of the National Academy of Sciences of the United States of America. 2007;104(38):14988–93. Epub 09/12. 10.1073/pnas.0707323104 17846422 PMC1986600

[32] Segre JA, Bauer C, Fuchs E. Klf4 is a transcription factor required for establishing the barrier function of the skin. Nature genetics. 1999;22(4):356–60. Epub 08/04. 10.1038/11926 10431239

[33] Sen GL, Boxer LD, Webster DE, Bussat RT, Qu K, Zarnegar BJ, et al ZNF750 is a p63 target gene that induces KLF4 to drive terminal epidermal differentiation. Developmental cell. 2012;22(3):669–77. Epub 03/01. Epub 2012 Feb 23. 10.1016/j.devcel.2011.12.001 22364861 PMC3306457

[34] Boxer LD, Barajas B, Tao S, Zhang J, Khavari PA. ZNF750 interacts with KLF4 and RCOR1, KDM1A, and CTBP1/2 chromatin regulators to repress epidermal progenitor genes and induce differentiation genes. Genes & development. 28 United States: 2014 Boxer et al.; Published by Cold Spring Harbor Laboratory Press.; 2014 p. 2013–26. 25228645 10.1101/gad.246579.114 PMC4173152

[35] Lopez-Pajares V, Qu K, Zhang J, Webster DE, Barajas BC, Siprashvili Z, et al A LncRNA-MAF:MAFB transcription factor network regulates epidermal differentiation. Developmental cell. 2015;32(6):693–706. Epub 03/26. 10.1016/j.devcel.2015.01.028 25805135 PMC4456036

[36] Johnston A, Fritz Y, Dawes SM, Diaconu D, Al-Attar PM, Guzman AM, et al Keratinocyte overexpression of IL-17C promotes psoriasiform skin inflammation. Journal of immunology (Baltimore, Md: 1950). 2013;190(5):2252–62. Epub 01/30. Epub 2013 Jan 28. 10.4049/jimmunol.1201505 PMC3577967 23359500

[37] Ziller MJ, Edri R, Yaffe Y, Donaghey J, Pop R, Mallard W, et al Dissecting neural differentiation regulatory networks through epigenetic footprinting. Nature. 2015;518(7539):355–9. Epub 2014/12/24. 10.1038/nature13990 25533951 PMC4336237

[38] Ross-Innes CS, Stark R, Teschendorff AE, Holmes KA, Ali HR, Dunning MJ, et al Differential oestrogen receptor binding is associated with clinical outcome in breast cancer. Nature. 2012;481(7381):389–93. Epub 01/06. 10.1038/nature10730 22217937 PMC3272464

[39] Kon S, Tanabe K, Watanabe T, Sabe H, Satake M. Clathrin dependent endocytosis of E-cadherin is regulated by the Arf6GAP isoform SMAP1. Experimental cell research. 2008;314(7):1415–28. Epub 03/12. Epub 2007 Nov 17. 10.1016/j.yexcr.2007.11.006 18331728

[40] Li X, Lee AY. Semaphorin 5A and plexin-B3 inhibit human glioma cell motility through RhoGDIalpha-mediated inactivation of Rac1 GTPase. The Journal of biological chemistry. 2010;285(42):32436–45. Epub 08/11. Epub 2010 Aug 9. 10.1074/jbc.M110.120451 20696765 PMC2952245

[41] Oh SY, Knelson EH, Blobe GC, Mythreye K. The type III TGFbeta receptor regulates filopodia formation via a Cdc42-mediated IRSp53-N-WASP interaction in epithelial cells. The Biochemical journal. 2013;454(1):79–89. Epub 06/12. 10.1042/BJ20121701 23750457 PMC4082962

[42] Lim S, Yoo BK, Kim HS, Gilmore HL, Lee Y, Lee HP, et al Amyloid-beta precursor protein promotes cell proliferation and motility of advanced breast cancer. BMC cancer. 2014;14:928 Epub 12/11. 10.1186/1471-2407-14-928 25491510 PMC4295427

[43] Du C, Huang T, Sun D, Mo Y, Feng H, Zhou X, et al CDH4 as a novel putative tumor suppressor gene epigenetically silenced by promoter hypermethylation in nasopharyngeal carcinoma. Cancer letters. 2011;309(1):54–61. Epub 06/15. Epub 2011 Jun 12. 10.1016/j.canlet.2011.05.016 21665361

[44] Andreeva AV, Kutuzov MA. Cadherin 13 in cancer. Genes, chromosomes & cancer. 2010;49(9):775–90. Epub 07/08. 20607704 10.1002/gcc.20787

[45] Kurschat P, Bielenberg D, Rossignol-Tallandier M, Stahl A, Klagsbrun M. Neuron restrictive silencer factor NRSF/REST is a transcriptional repressor of neuropilin-1 and diminishes the ability of semaphorin 3A to inhibit keratinocyte migration. J Biol Chem. 2006;281(5):2721–9. Epub 2005/12/07. 10.1074/jbc.M507860200 16330548

[46] Heinz S, Romanoski CE, Benner C, Glass CK. The selection and function of cell type-specific enhancers. Nature reviews Molecular cell biology. 2015;16(3):144–54. Epub 02/05. Epub 2015 Feb 4. 10.1038/nrm3949 25650801 PMC4517609

[47] Brown JD, Lin CY, Duan Q, Griffin G, Federation AJ, Paranal RM, et al NF-kappaB directs dynamic super enhancer formation in inflammation and atherogenesis. Molecular cell. 2014;56(2):219–31. Epub 09/30. Epub 2014 Sep 25. 10.1016/j.molcel.2014.08.024 25263595 PMC4224636

[48] Loven J, Hoke HA, Lin CY, Lau A, Orlando DA, Vakoc CR, et al Selective inhibition of tumor oncogenes by disruption of super-enhancers. Cell. 2013;153(2):320–34. Epub 04/16. 10.1016/j.cell.2013.03.036 23582323 PMC3760967

[49] Mardaryev AN, Liu B, Rapisarda V, Poterlowicz K, Malashchuk I, Rudolf J, et al Cbx4 maintains the epithelial lineage identity and cell proliferation in the developing stratified epithelium. The Journal of cell biology. 2016;212(1):77–89. Epub 2015/12/30. 10.1083/jcb.201506065 26711500 PMC4700479

[50] Wells J, Lee B, Cai AQ, Karapetyan A, Lee WJ, Rugg E, et al Ovol2 suppresses cell cycling and terminal differentiation of keratinocytes by directly repressing c-Myc and Notch1. The Journal of biological chemistry. 2009;284(42):29125–35. Epub 08/25. Epub 2009 Aug 21. 10.1074/jbc.M109.008847 19700410 PMC2781457

[51] Lee B, Villarreal-Ponce A, Fallahi M, Ovadia J, Sun P, Yu QC, et al Transcriptional mechanisms link epithelial plasticity to adhesion and differentiation of epidermal progenitor cells. Developmental cell. 2014;29(1):47–58. Epub 04/17. 10.1016/j.devcel.2014.03.005 24735878 PMC4153751

[52] Chen W, Xiao Liu Z, Oh JE, Shin KH, Kim RH, Jiang M, et al Grainyhead-like 2 (GRHL2) inhibits keratinocyte differentiation through epigenetic mechanism. Cell death & disease. 2012;3:e450. Epub 12/21. 23254293 10.1038/cddis.2012.190 PMC3542624

[53] Rockowitz S, Lien WH, Pedrosa E, Wei G, Lin M, Zhao K, et al Comparison of REST cistromes across human cell types reveals common and context-specific functions. PLoS computational biology. 2014;10(6):e1003671 Epub 06/13. 10.1371/journal.pcbi.1003671 24922058 PMC4055426

[54] Yu Z, Mannik J, Soto A, Lin KK, Andersen B. The epidermal differentiation-associated Grainyhead gene Get1/Grhl3 also regulates urothelial differentiation. The EMBO journal. 2009;28(13):1890–903. Epub 06/06. Epub 2009 Jun 4. 10.1038/emboj.2009.142 19494835 PMC2711180

[55] Schmidt D, Wilson MD, Spyrou C, Brown GD, Hadfield J, Odom DT. ChIP-seq: using high-throughput sequencing to discover protein-DNA interactions. Methods (San Diego, Calif). 2009;48(3):240–8. Epub 03/12. Epub 2009 Mar 9. 10.1016/j.ymeth.2009.03.001 PMC4052679 19275939

[56] Kayala MA, Baldi P. Cyber-T web server: differential analysis of high-throughput data. Nucleic acids research. 2012;40(Web Server issue):W553–9. Epub 05/19. Epub 2012 May 16. 10.1093/nar/gks420 22600740 PMC3394347

[57] Langmead B, Trapnell C, Pop M, Salzberg SL. Ultrafast and memory-efficient alignment of short DNA sequences to the human genome. Genome biology. 2009;10(3):R25 Epub 03/06. Epub 2009 Mar 4. 10.1186/gb-2009-10-3-r25 19261174 PMC2690996

[58] Zang C, Schones DE, Zeng C, Cui K, Zhao K, Peng W. A clustering approach for identification of enriched domains from histone modification ChIP-Seq data. Bioinformatics (Oxford, England). 2009;25(15):1952–8. Epub 06/10. Epub 2009 Jun 8 10.1093/bioinformatics/btp340 PMC2732366 19505939

[59] McLean CY, Bristor D, Hiller M, Clarke SL, Schaar BT, Lowe CB, et al GREAT improves functional interpretation of cis-regulatory regions. Nature biotechnology. 2010;28(5):495–501. Epub 05/04. Epub 2010 May 2. 10.1038/nbt.1630 20436461 PMC4840234

[60] Daily K, Patel VR, Rigor P, Xie X, Baldi P. MotifMap: integrative genome-wide maps of regulatory motif sites for model species. BMC Bioinformatics. 2011;12:495 Epub 2012/01/03. 10.1186/1471-2105-12-495 22208852 PMC3293935

[61] Blankenberg D, Von Kuster G, Coraor N, Ananda G, Lazarus R, Mangan M, et al. Galaxy: a web-based genome analysis tool for experimentalists. Current protocols in molecular biology / edited by Ausubel Frederick M [et al]. 2010;Chapter 19:Unit 19.0.1–21. Epub 01/14. 10.1002/0471142727.mb1910s89 PMC4264107 20069535

[62] Goecks J, Nekrutenko A, Taylor J. Galaxy: a comprehensive approach for supporting accessible, reproducible, and transparent computational research in the life sciences. Genome biology. 2010;11(8):R86 Epub 08/27. Epub 2010 Aug 25. 10.1186/gb-2010-11-8-r86 20738864 PMC2945788

[63] Heinz S, Benner C, Spann N, Bertolino E, Lin YC, Laslo P, et al Simple combinations of lineage-determining transcription factors prime cis-regulatory elements required for macrophage and B cell identities. Molecular cell. 2010;38(4):576–89. Epub 06/02. 10.1016/j.molcel.2010.05.004 20513432 PMC2898526

[64] Bailey TL, Boden M, Buske FA, Frith M, Grant CE, Clementi L, et al MEME SUITE: tools for motif discovery and searching. Nucleic acids research. 2009;37(Web Server issue):W202–8. Epub 05/22. Epub 2009 May 20 10.1093/nar/gkp335 19458158 PMC2703892

[65] Hu Z, Chang YC, Wang Y, Huang CL, Liu Y, Tian F, et al VisANT 4.0: Integrative network platform to connect genes, drugs, diseases and therapies. Nucleic acids research. 2013;41(Web Server issue):W225–31. Epub 2013/05/30. 10.1093/nar/gkt401 23716640 PMC3692070

