# Supplementary figures and images for: GRHL3 binding and enhancers rearrange as epidermal keratinocytes transition between functional states
Source: PLoS Genet. 2017 Apr 26;13(4):e1006745. doi: 10.1371/journal.pgen.1006745 (PMC5425218; doi:10.1371/journal.pgen.1006745)

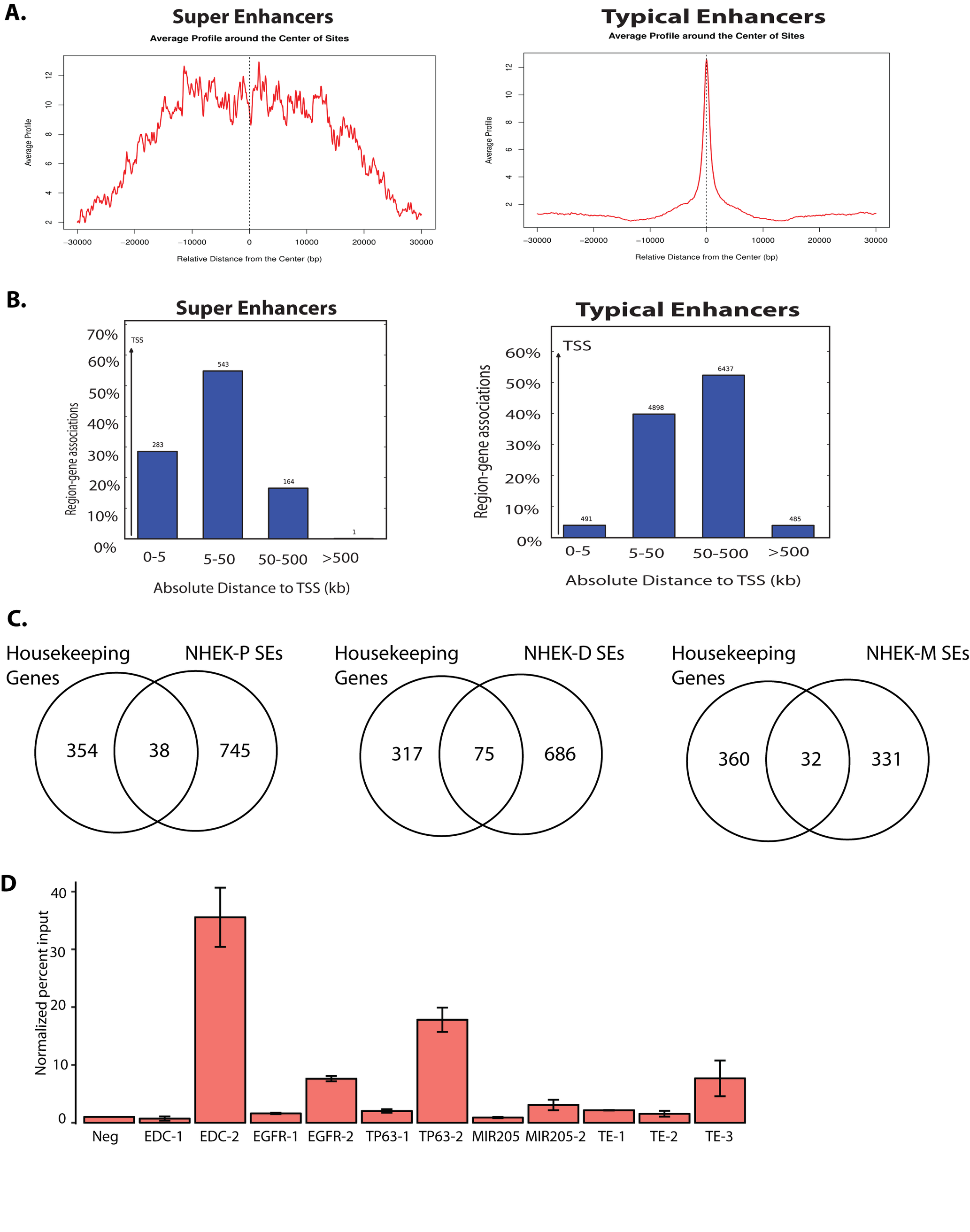

Supplement: S1 Fig — (A) Plot of H3K27ac signal intensity and length for SEs and TEs in NHEK-P. (B) Comparison of the distance from TE or SE to the nearest gene. (C) Overlap of SEs with housekeeping genes in each cell state. (D) ChIP-qPCR of MED1 binding to SE (2 primer sets for each of 4 SE tested) and TE (one primer set for each of 3 TE tested). Neg = negative control, EDC = epidermal differentiation complex, TE = typical enhancer, n = 3. (TIF) [file pgen.1006745.s001.tif]

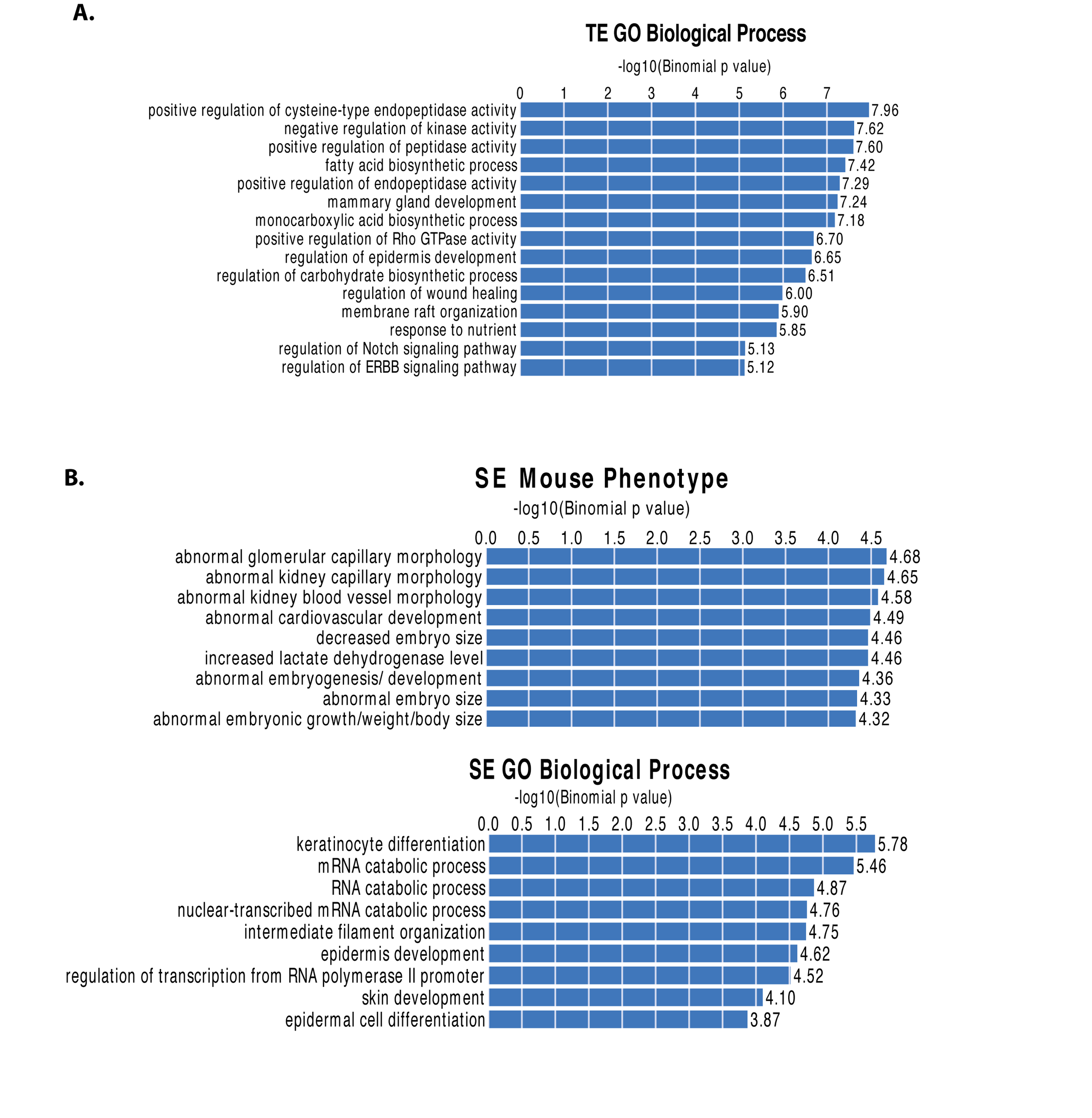

Supplement: S2 Fig — (A) Gene ontology analysis for genes near TE shared between NHEK-P, NHEK-M, and NHEK-D. (B) Gene ontology analysis for genes near SEs. (TIF) [file pgen.1006745.s002.tif]

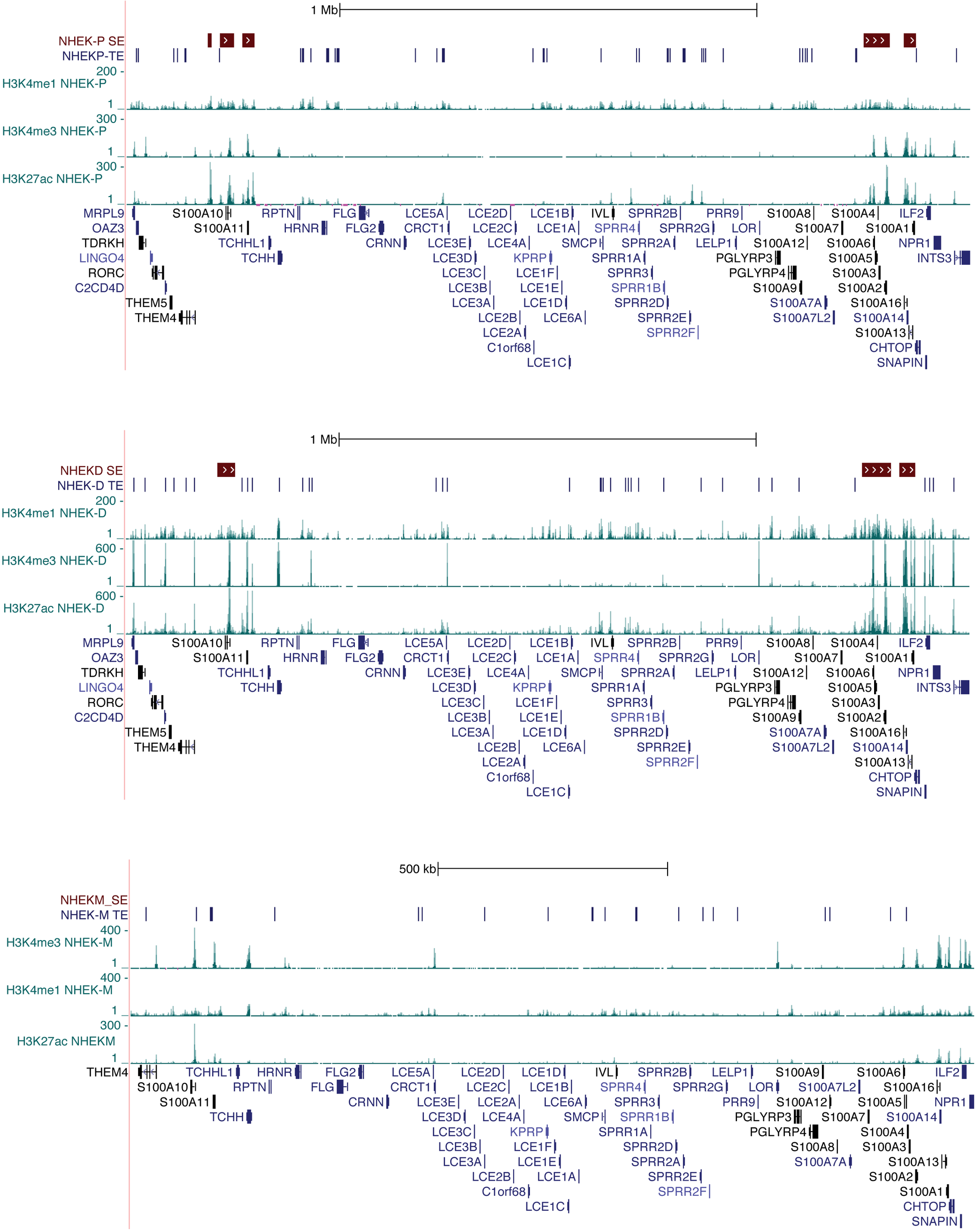

Supplement: S3 Fig — (TIF) [file pgen.1006745.s003.tif]

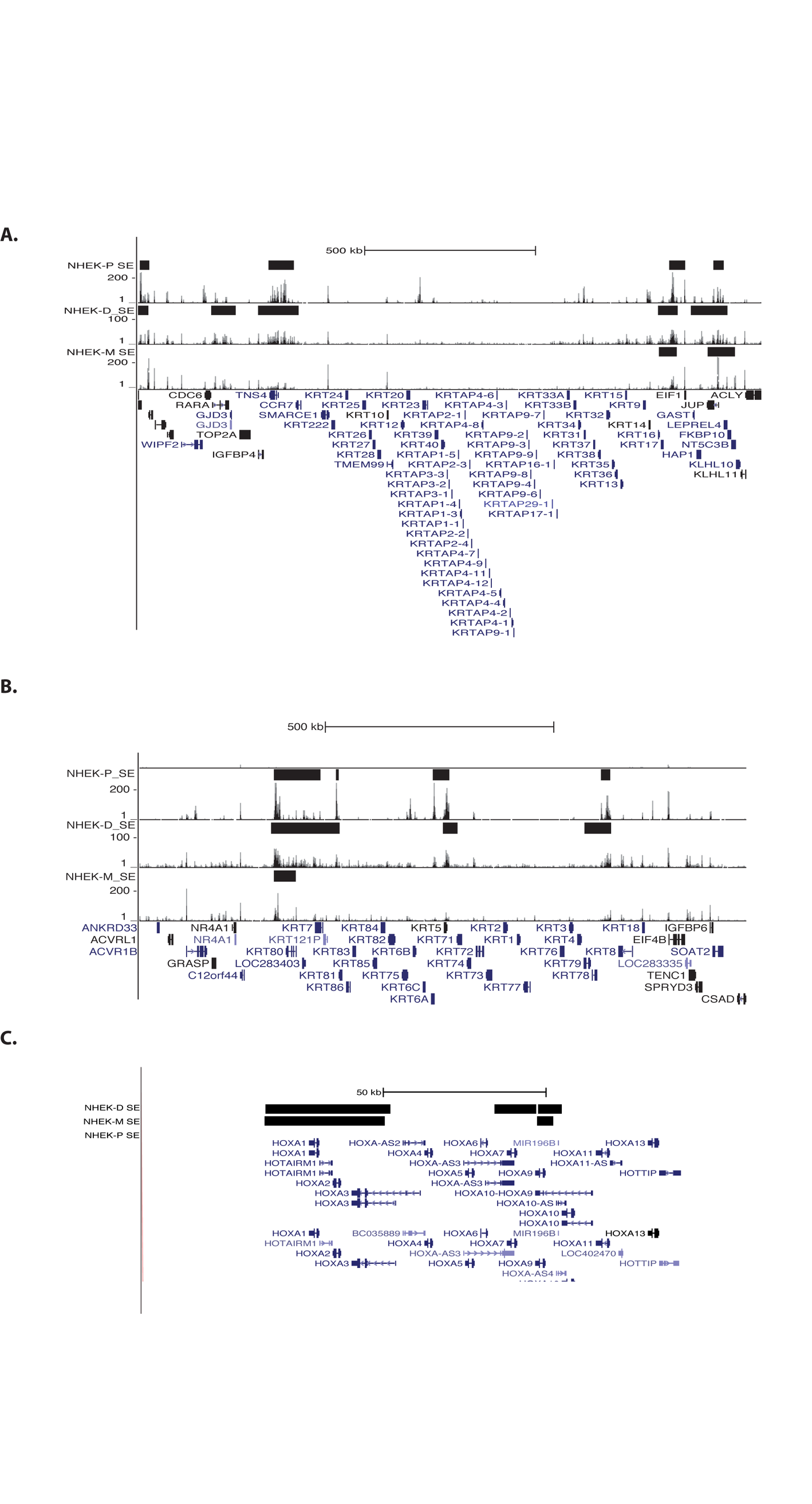

Supplement: S4 Fig — (A) SEs at the keratin gene cluster on chromosome 17. (B) SEs at the keratin gene cluster on chromosome 12. C) SEs at the HOXA gene locus. (TIF) [file pgen.1006745.s004.tif]

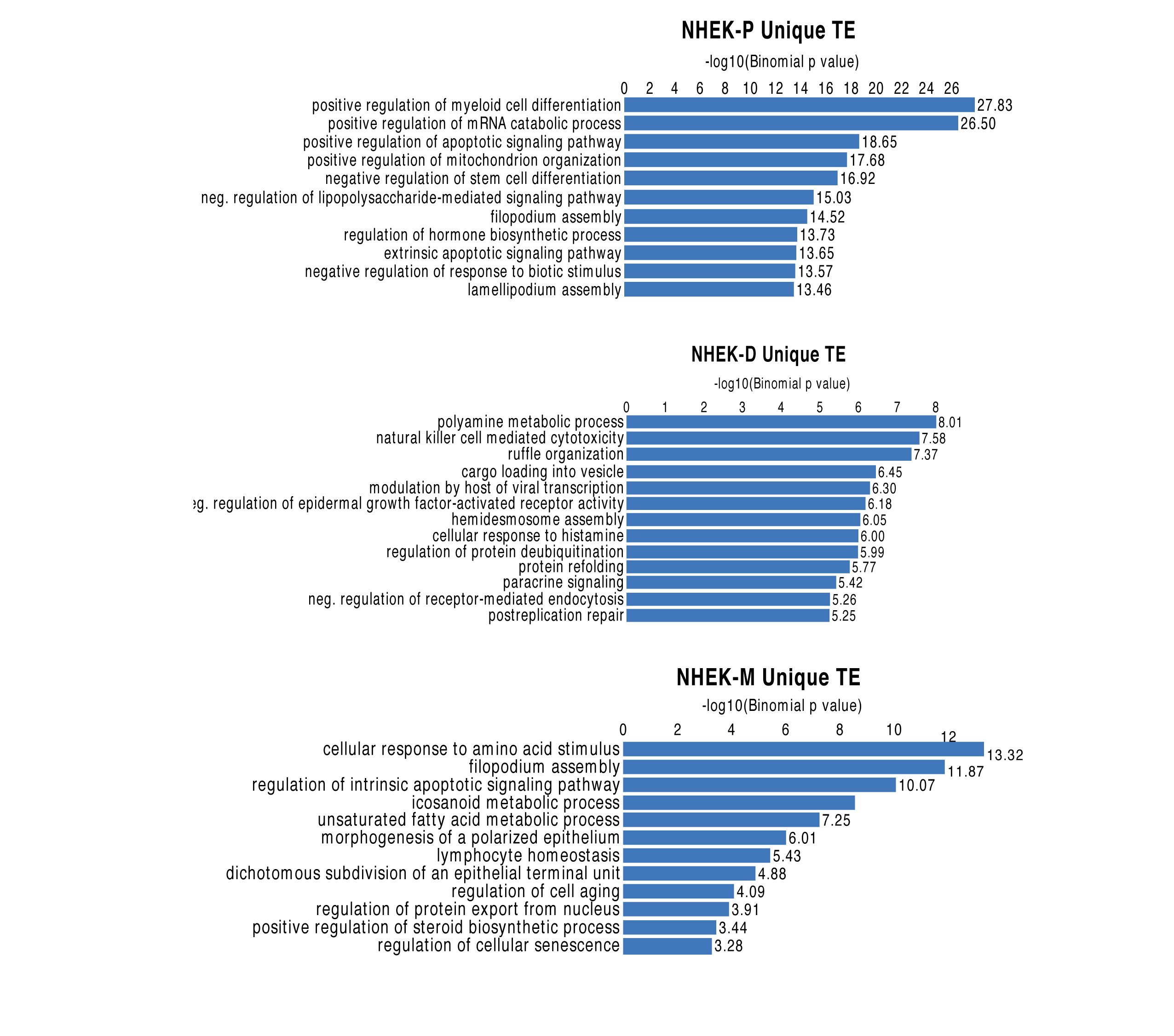

Supplement: S5 Fig — (TIF) [file pgen.1006745.s005.tif]

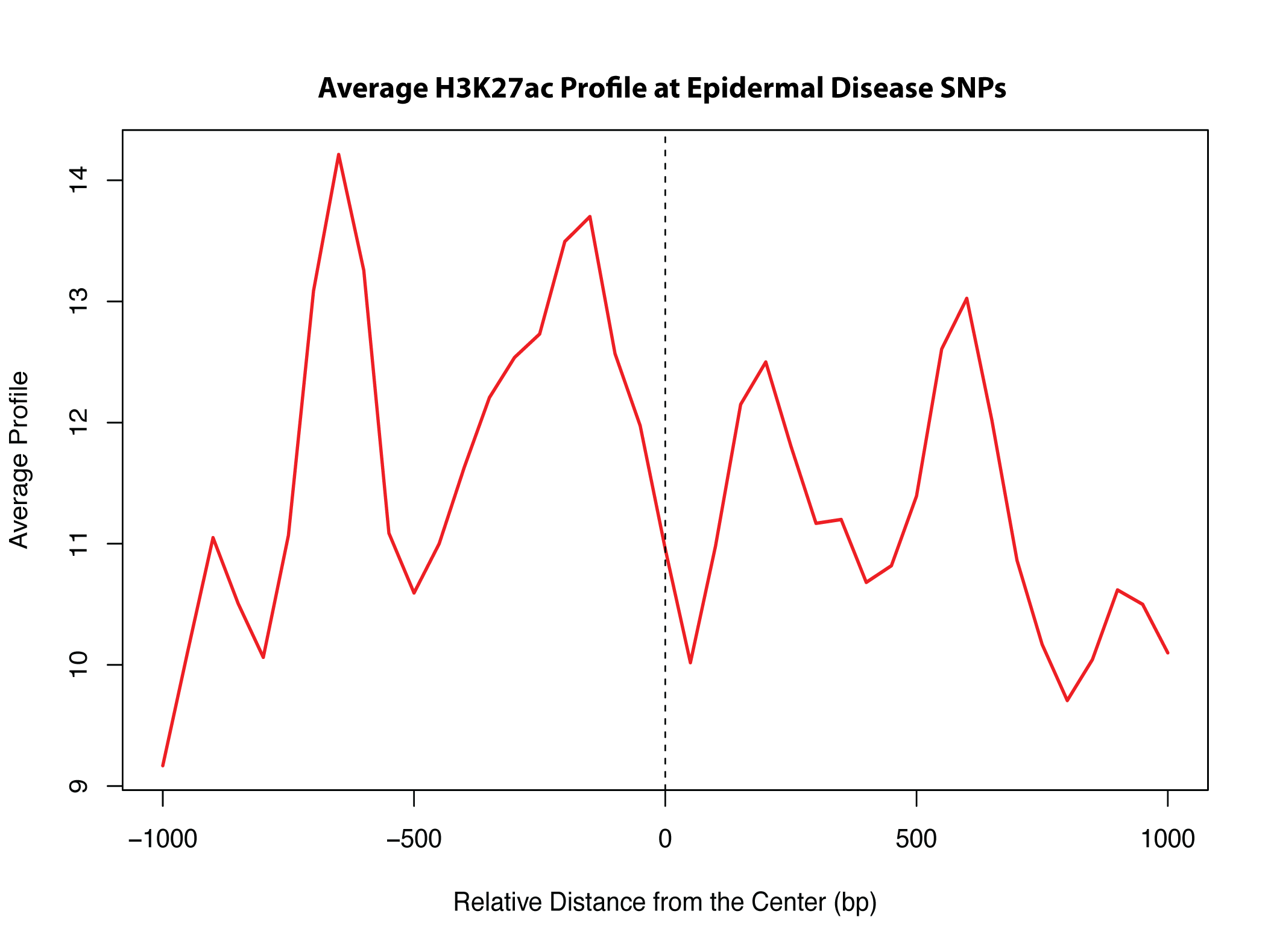

Supplement: S6 Fig — (TIF) [file pgen.1006745.s006.tif]

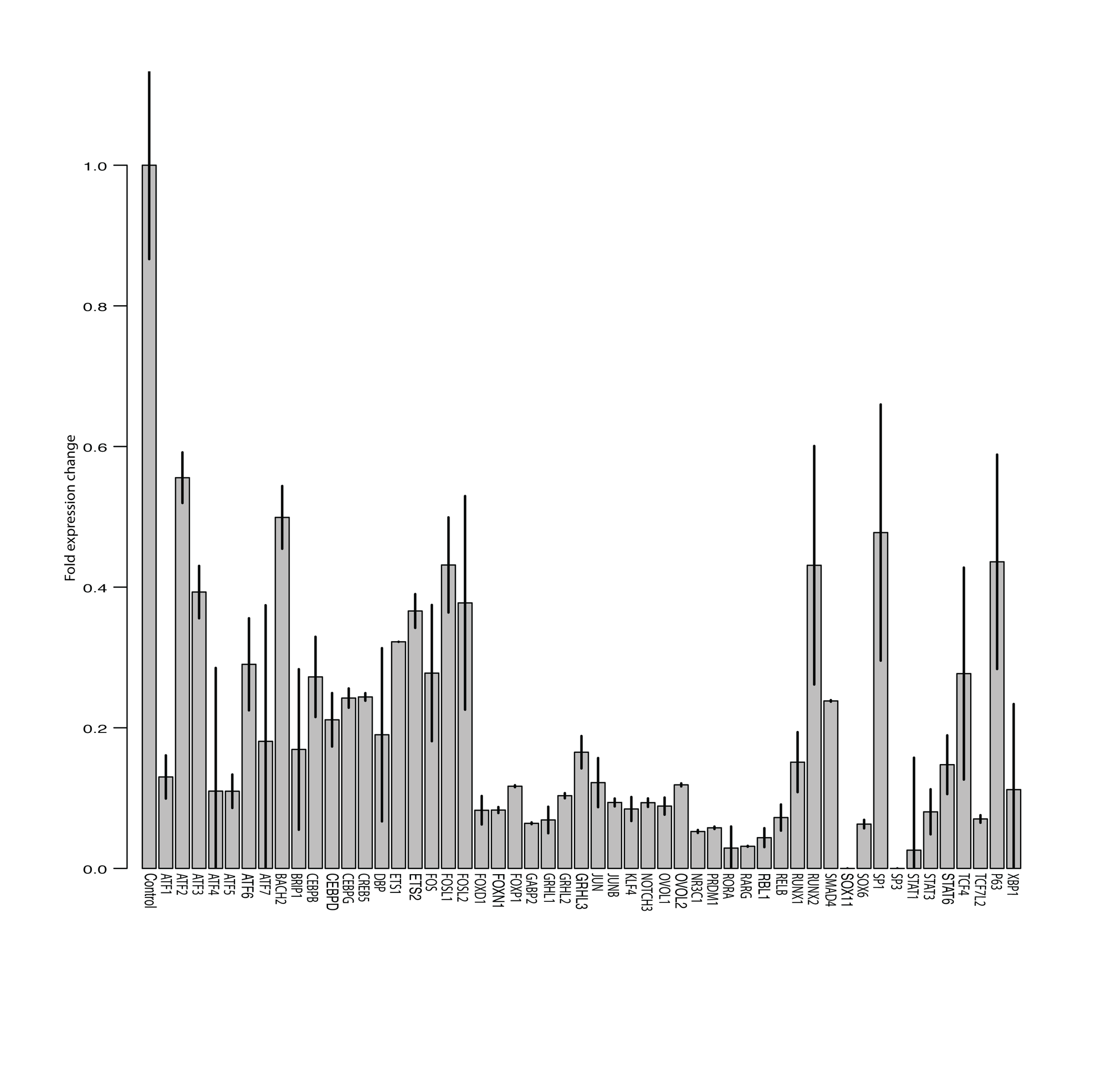

Supplement: S7 Fig — (TIF) [file pgen.1006745.s007.tif]

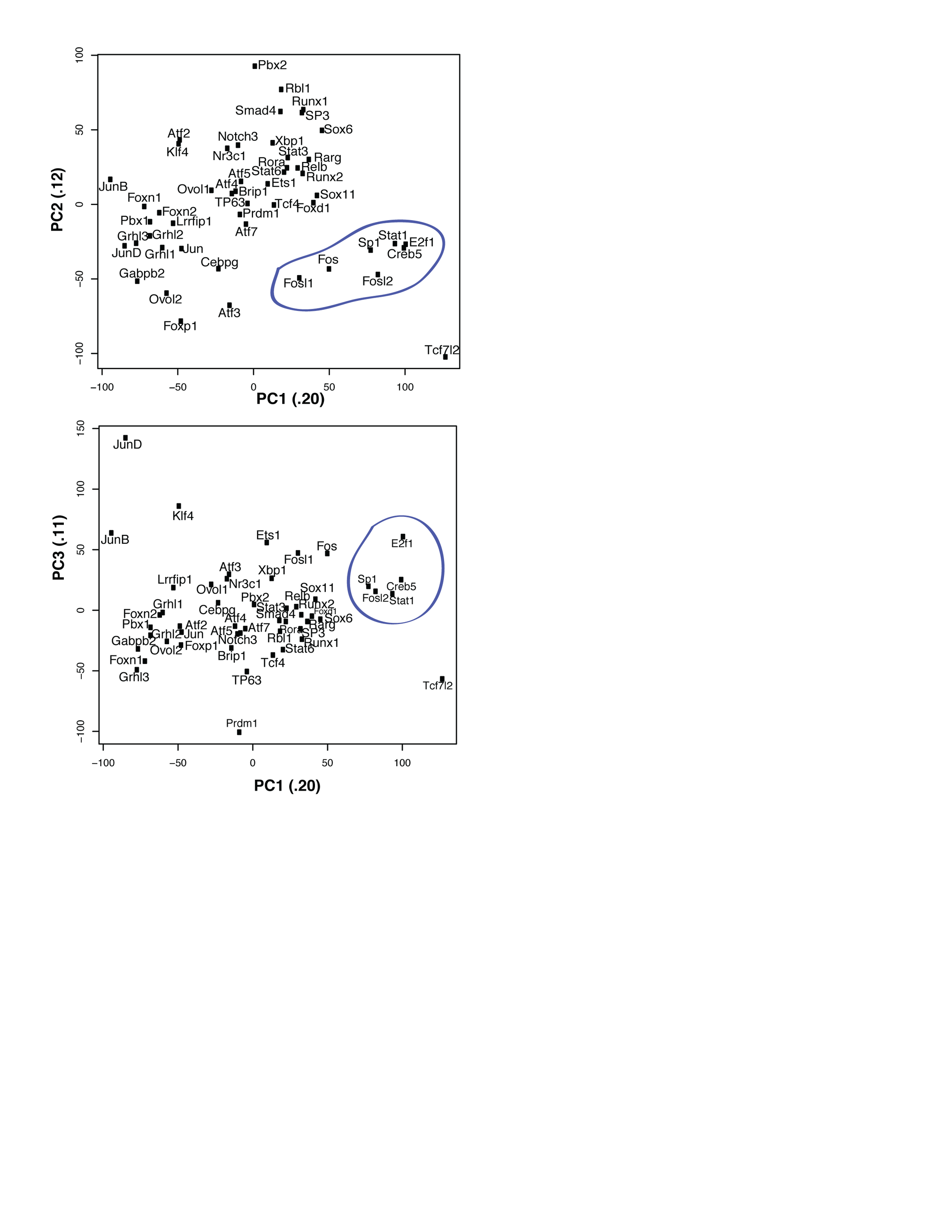

Supplement: S8 Fig — (TIF) [file pgen.1006745.s008.tif]

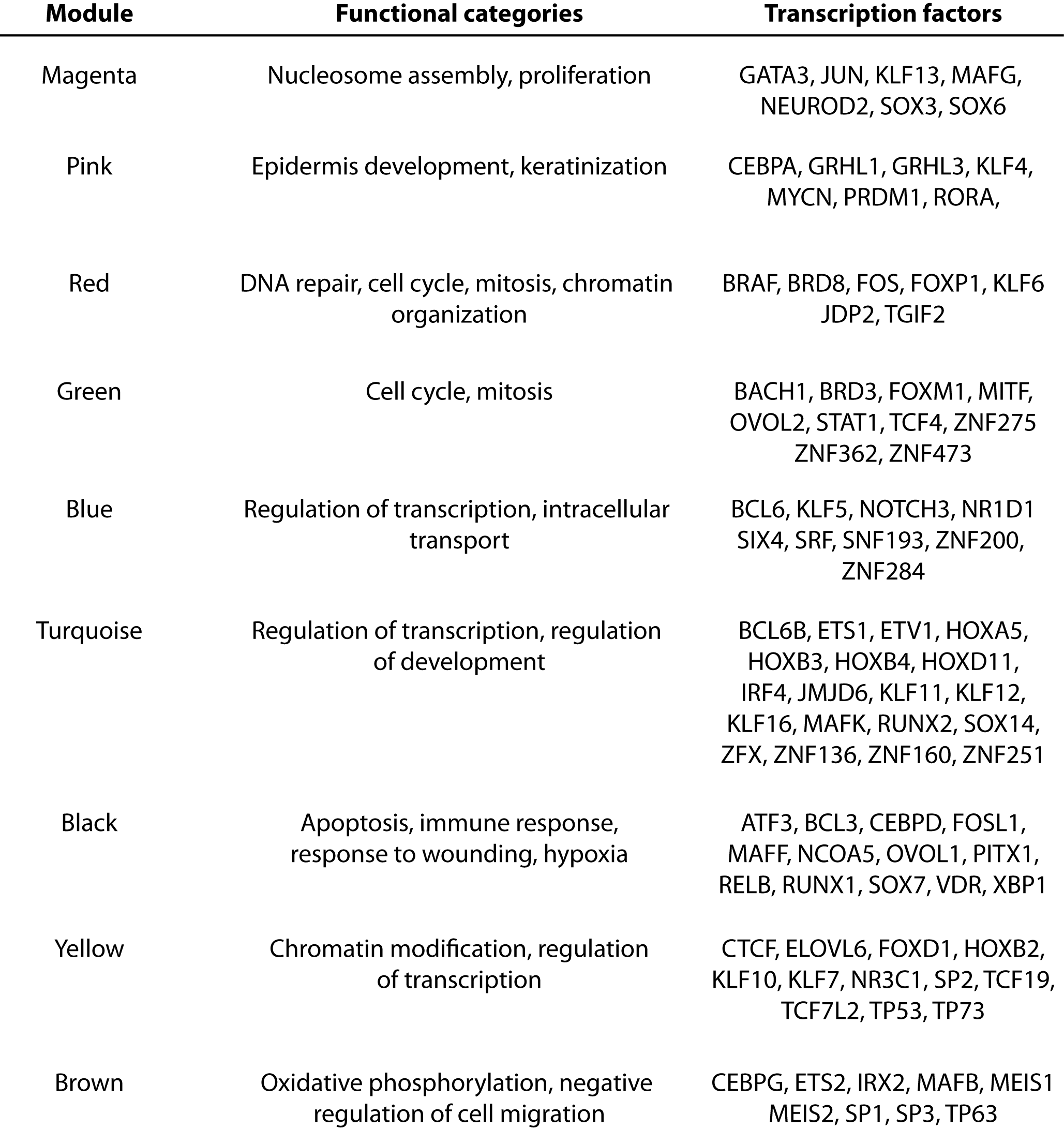

Supplement: S9 Fig — Gene ontology of top identified co-expression modules and list of selected transcription factors. (TIF) [file pgen.1006745.s009.tif]

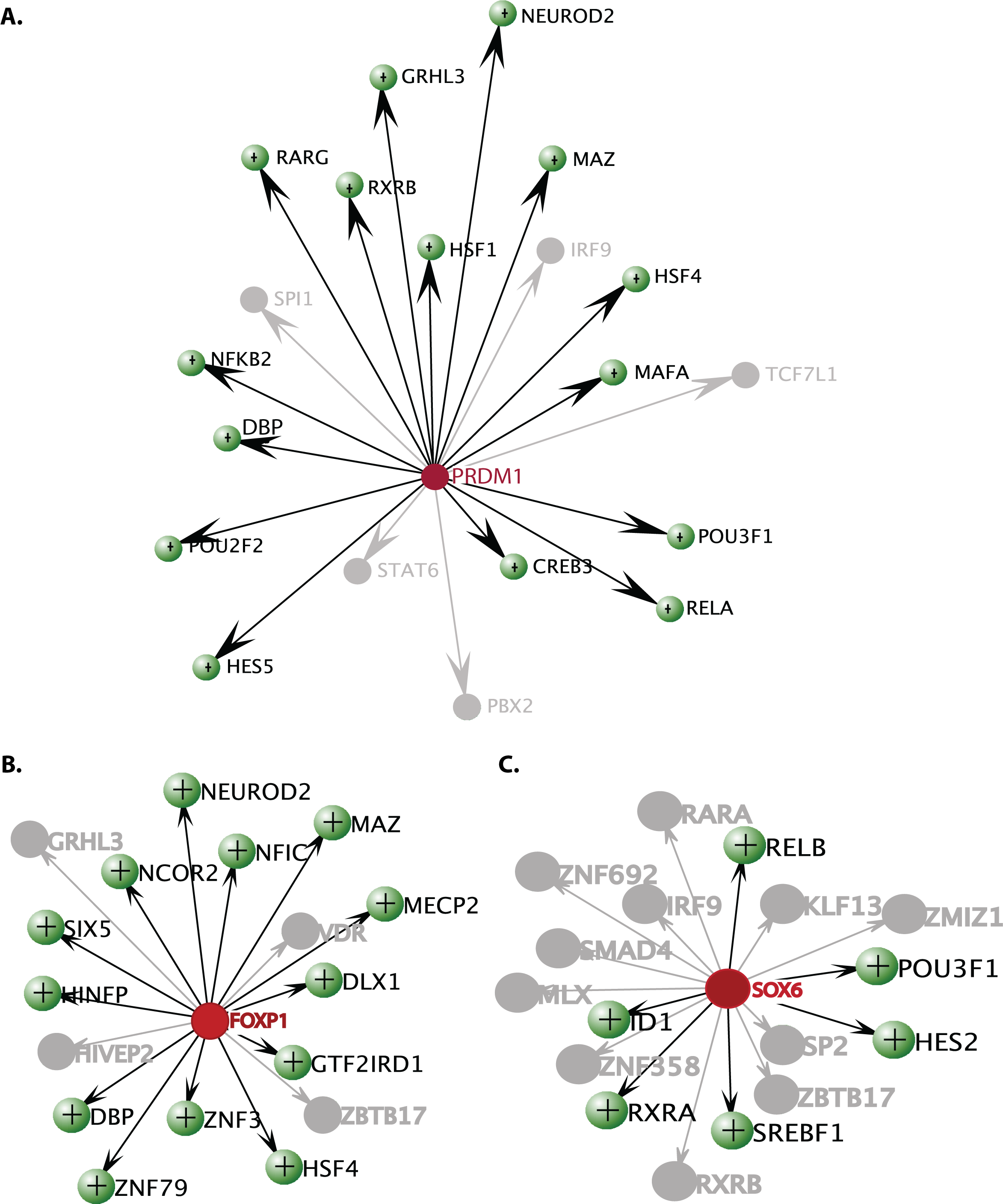

Supplement: S10 Fig — Predicted gene targets that did not validate based on the siRNA experiment are presented in grey. (TIF) [file pgen.1006745.s010.tif]

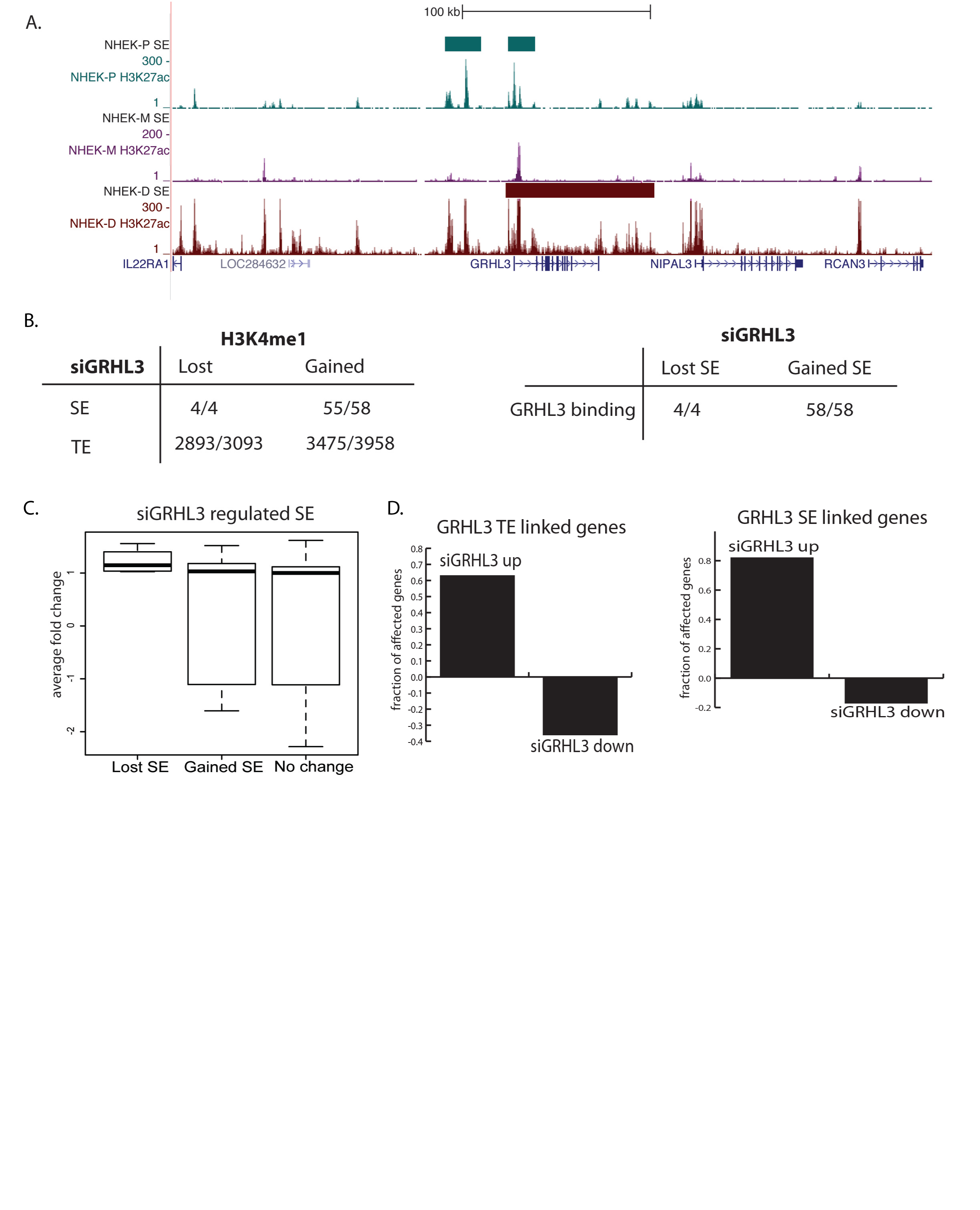

Supplement: S11 Fig — (A) An SE overlapping the Grhl3 gene body in NHEK-D and NHEK-P. (B) Overlap of H3K4me1 histone modification with TEs and SEs gained or lost by siGRHL3. Overlap of GRHL3 binding with SEs gained or lost by siGRHL3. (C) Effect of GRHL3 knockdown on expression of nearest gene to SEs lost, SEs gained, and SEs unchanged. (D) Effect of GRHL3 knockdown on expression of nearest gene to GRHL3 bound TE, and to GRHL3 bound, non-promoter SE. (TIF) [file pgen.1006745.s011.tif]

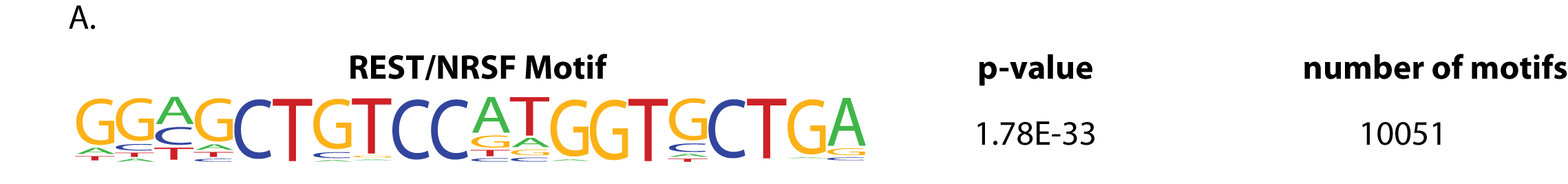

Supplement: S12 Fig — (A) REST motif and enrichment score for GRHL3 peaks unique to migration. (TIF) [file pgen.1006745.s012.tif]

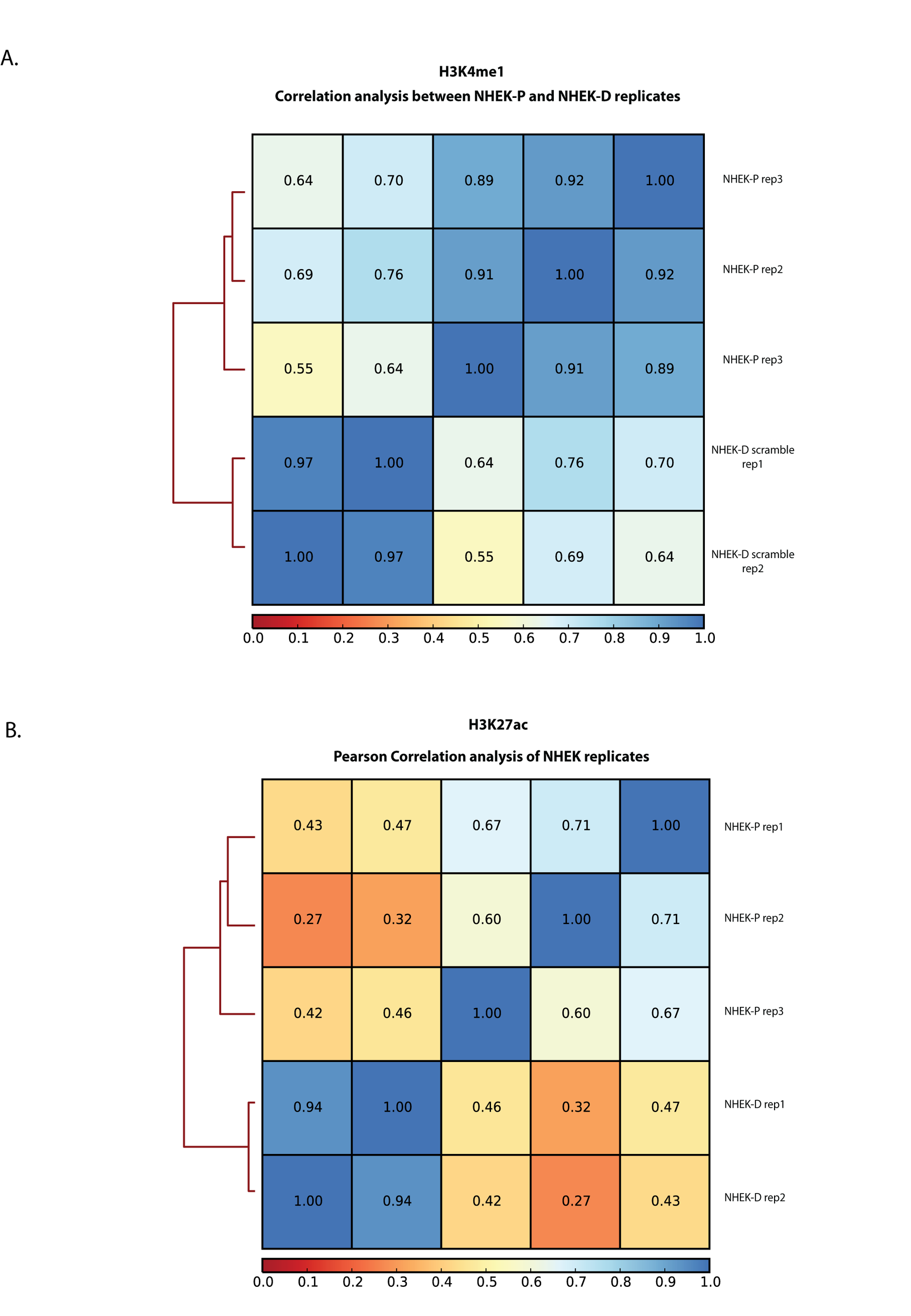

Supplement: S13 Fig — (TIF) [file pgen.1006745.s013.tif]
