# Supplementary material for: GRHL3 binding and enhancers rearrange as epidermal keratinocytes transition between functional states
Source: PLoS Genet. 2017 Apr 26;13(4):e1006745. doi: 10.1371/journal.pgen.1006745 (PMC5425218; doi:10.1371/journal.pgen.1006745)
Supplement: S1 Table — (PDF) [file pgen.1006745.s014.pdf]

Table S1. List of probe names, gene symbols, and accession numbers for custom array.

ProbeName	Symbol	Accesion
A_23_P31536	CXCL2	NM_002089
A_33_P32942	MTF1	NM_005955
A_23_P71103	TFPI2	ENST00000222543
A_33_P33114	LOC283398	XM_208658
A_32_P55273	GPR157	ENST00000377411
A_33_P32478	ZNF844	NM_001136501
A_23_P37126	DNM3	NM_015569
A_23_P32919	OBFC2A	NM_001031716
A_23_P1461	OPTN	NM_001008211
A_23_P79398	IL1R2	NM_004633
A_23_P21594	CTSB	NM_147780
A_24_P21665	SOAT1	NM_003101
A_33_P32468	IL1RN	NM_173843
A_24_P72788	C11orf41	NM_012194
A_23_P15749	PPP3CC	NM_005605
A_23_P97621	RRAGC	NM_022157
A_23_P13753	NFE2	NM_006163
A_33_P33333	OPTN	NM_001008211
A_33_P33697	GLIPR2	NM_022343
A_32_P14803	BACH1	NM_206866
A_23_P7144	CXCL1	NM_001511
A_23_P32702	MDFIC	NM_199072
A_24_P26729	SARM1	NM_015077
A_24_P37262	RNF141	NM_016422
A_23_P14139	WIPI1	NM_017983
A_23_P12490	NPTX1	NM_002522
A_32_P20454	COPA	NM_001098398
A_23_P21328	SCD5	NM_001037582
A_23_P20383	GLIPR1	NM_006851
A_33_P33632	PGM2L1	NM_173582
A_23_P36704	DCUN1D3	NM_173475
A_23_P79658	CCDC93	NM_019044
A_23_P52161	NUAK2	NM_030952
A_23_P20923	CLIP4	NM_024692
A_23_P20999	IL1RN	NM_173842
A_33_P33185	PLOD2	NM_182943
A_24_P55631	SAA3P	NR_026576
A_23_P51126	IL1RL1	NM_016232
A_23_P42602	SEL1L3	NM_015187
A_32_P42056	RNF215	NM_001017981
A_33_P32850	ZNF75A	NM_153028
A_23_P26314	ZNF319	NM_020807
A_23_P17073	ANTXR2	NM_058172
A_23_P24104	PLAU	NM_002658
A_32_P48976	S1PR3	NM_005226
A_33_P34105	FAM46A	NM_017633
A_33_P32812	S1PR3	NM_005226

A_32_P12270	PGM2L1	NM_173582
A_24_P36597	COL8A2	NM_005202
A_23_P12158	HERC3	NM_014606
A_23_P21666	FOXE1	NM_004473
A_33_P33555	FOXL1	NM_005250
A_23_P86959	BIRC2	NM_001166
A_24_P36406	HIPK3	ENST00000303296
A_23_P30417	KIAA0226	NM_001145642
A_33_P32129	PLEKHM1	NM_014798
A_33_P34037	ZNF569	NM_152484
A_23_P21046	PI3	NM_002638
A_24_P8371	SPNS2	NM_001124758
A_24_P38160	ITM2B	NM_021999
A_23_P76450	PHLDA1	NM_007350
A_33_P32441	NDEL1	NM_001025579
A_23_P39000	PCSK6	NM_138325
A_23_P11806	HSD17B2	NM_002153
A_24_P30415	AMPD3	NM_001025390
A_23_P15197	FEM1B	NM_015322
A_32_P46571	RHBDL2	NM_017821
A_32_P10543	MAP1LC3B	NM_022818
A_23_P42353	ETV7	NM_016135
A_33_P33314	TGFBR1	NM_004612
A_23_P51346	KIAA2013	ENST00000376572
A_23_P83579	ARNT2	NM_014862
A_24_P37975	MXD1	NM_002357
A_23_P83931	NET1	NM_001047160
A_23_P25451	EFNA1	NM_004428
A_33_P33635	TMEM51	NM_001136216
A_23_P6216	APP	NM_000484
A_23_P43138	SPOCD1	NM_144569
A_33_P33817	C17orf39	NM_024052
A_33_P32474	SPRR2B	NM_001017418
A_32_P15933	RECQL	NM_002907
A_32_P10400	DCUN1D3	NM_173475
A_23_P20388	MMP19	NM_002429
A_23_P81770	PTP4A1	NM_003463
A_24_P18137	NEFL	NM_006158
A_32_P22620	ZFHX2	NM_033400
A_33_P33883	SKAP2	NM_003930
A_33_P33125	KPRP	NM_001025231
A_23_P14160	BLMH	NM_000386
A_23_P12158	HERC3	NM_014606
A_23_P12358	IPPK	NM_022755
A_33_P32603	NR6A1	NM_033334
A_23_P58266	S100P	NM_005980
A_23_P16794	TREM2	NM_018965
A_33_P32159	MPZL2	NM_005797
A_23_P34511	PIM1	NM_002648

A_23_P50304:	F11R	NM_016946
A_33_P34134(	EDA2R	NM_021783
A_23_P90311	TICAM1	NM_182919
A_23_P35005!	C1orf152	NR_003242
A_23_P95930	HMGA2	NM_003483
A_24_P10830:	RTTN	NM_173630
A_24_P26931!	SERPINB1	ENST00000380739
A_23_P72097	CNKSR2	ENST00000379510
A_23_P10140(	C3	NM_000064
A_33_P32696:	SBSN	NM_001166034
A_23_P79518	IL1B	NM_000576
A_23_P39294	PLAC2	NR_027064
A_23_P99693	ZBTB1	NM_014950
A_33_P33529:	IRAK2	NM_001570
A_33_P35995!	PAPPA	NM_002581
A_23_P36032(	AIM1L	NM_001039775
A_24_P31936(	F11R	NM_016946
A_24_P37356:	ADAP2	NM_018404
A_32_P33567	LOC10000967(	NR_024407
A_23_P40154:	PVRL3	NM_015480
A_33_P33844:	TFDP2	NM_006286
A_33_P34145!	TWIST1	NM_000474
A_23_P78518	CEACAM19	NM_020219
A_23_P33847!	CD274	NM_014143
A_23_P71570	OSR2	NM_053001
A_23_P12292(	INHBA	NM_002192
A_23_P55666	ZNF702P	AK023047
A_23_P40613:	TMEM159	NM_020422
A_23_P39134(	RASGEF1A	BC022548
A_33_P33381(	NUAK2	NM_030952
A_23_P21254:	RAB5A	NM_004162
A_24_P11576:	CTSC	NM_148170
A_23_P16344(	MEX3B	NM_032246
A_24_P17556:	ACBD5	NM_145698
A_24_P56388	HIF1A	NM_181054
A_24_P19635:	PLXNC1	NM_005761
A_23_P13554:	GPSM1	NM_001145638
A_23_P10140:	C3	NM_000064
A_32_P11655(	ZNF469	NM_001127464
A_23_P1691	MMP1	NM_002421
A_23_P15230!	CDH11	NM_001797
A_23_P25648:	CD274	ENST00000381577
A_33_P33169:	PELI1	NM_020651
A_23_P25597!	FOXD1	NM_004472
A_32_P21593!	GPSM1	NM_001145638
A_23_P79836	SERINC3	NM_006811
A_23_P46588	IRF2BP2	NM_182972
A_24_P84296:	ZXDA	NM_007156
A_23_P98350	BIRC3	NM_001165

A_24_P32247[illegible]	PDE4A	NM_006202
A_23_P35242[illegible]	CDRT1	NM_006382
A_33_P32144[illegible]	MESP1	NM_018670
A_33_P33719[illegible]	TPPP	NM_007030
A_33_P32820[illegible]	SP6	NM_199262
A_23_P42207[illegible]	B3GALT4	NM_003782
A_23_P13988[illegible]	CCND2	NM_001759
A_23_P16423	ZBTB7A	AL390151
A_23_P33733	INPP5D	NM_001017915
A_23_P86012	LAMB3	NM_001017402
A_33_P33061[illegible]	KIAA1841	NM_032506
A_24_P15341[illegible]	PNMA2	NM_007257
A_32_P21362[illegible]	RTCD1	NM_001130841
A_23_P50198[illegible]	CSF2RA	NM_172249
A_23_P20223	RRM2B	NM_015713
A_23_P78458	ZNF350	NM_021632
A_23_P21369[illegible]	NRG2	NM_013982
A_32_P75284	KATNAL1	NM_032116
A_33_P33496[illegible]	PCDH1	NM_002587
A_24_P13422[illegible]	TNFAIP8L1	NM_001167942
A_23_P12690[illegible]	TNFRSF14	NM_003820
A_24_P66603[illegible]	KRT77	NM_175078
A_23_P16428[illegible]	CLDN7	NM_001307
A_23_P25595[illegible]	MYO6	NM_004999
A_23_P66203	PRSS22	NM_022119
A_23_P87238	SAA4	NM_006512
A_33_P34234[illegible]	ZNF770	NM_014106
A_33_P34003[illegible]	PRIC285	NM_001037335
A_23_P35039[illegible]	CDSN	NM_001264
A_23_P10338[illegible]	PBXIP1	NM_020524
A_23_P92909	SPINK6	NM_205841
A_24_P29650[illegible]	SLC43A2	NM_152346
A_33_P32427[illegible]	CPS1	NM_001875
A_24_P39009[illegible]	GLIPR1	NM_006851
A_23_P53288	CNPY2	NM_014255
A_32_P89310	PLEKHM3	NM_001080475
A_23_P67708	TCF3	NM_003200
A_23_P42627[illegible]	KIF19	NM_153209
A_23_P25962[illegible]	LAT2	NM_032464
A_23_P79758	LOC151009	NR_027244
A_23_P58636	PITX1	NM_002653
A_24_P19104[illegible]	CRCT1	NM_019060
A_23_P32616[illegible]	ZC3H12A	NM_025079
A_23_P83298	PRRX2	NM_016307
A_32_P76479[illegible]	CNKSR2	ENST00000379510
A_23_P16469	PLAUR	NM_001005377
A_23_P21269[illegible]	FSTL1	NM_007085
A_23_P16231[illegible]	WNT10B	NM_003394
A_23_P30363	P4HA2	NM_004199

A_33_P34226	FAM149B1	NM_173348
A_23_P40711	SPATA18	NM_145263
A_23_P97332	SLC16A4	NM_004696
A_24_P32948	FAM84B	NM_174911
A_23_P42273	WDR63	NM_145172
A_23_P74001	S100A12	NM_005621
A_33_P33819	WTIP	NM_001080436
A_32_P36692	JAZF1	NM_175061
A_23_P36825	EID2B	NM_152361
A_33_P34074	CDC42EP1	NM_152243
A_32_P85999	CDH13	NM_001257
A_23_P21749	GDPD2	NM_017711
A_23_P30267	DDIT4L	BC013592
A_23_P11253	FAM102A	NM_001035254
A_23_P16465	APOE	NM_000041
A_33_P32189	KDM6A	NM_021140
A_24_P19047	SLPI	NM_003064
A_24_P35562	ABCG4	NM_022169
A_23_P72989	CCR4	NM_005508
A_23_P11644	SPRR2D	NM_006945
A_23_P20354	EHF	NM_012153
A_23_P21835	FBXW10	NM_031456
A_24_P75947	ITGB8	NM_002214
A_23_P10105	KRT34	NM_021013
A_24_P34880	PLEKHA7	NM_175058
A_23_P10447	DUSP13	NM_001007271
A_23_P80759	PVRL3	ENST00000485303
A_23_P49816	ADAP2	NM_018404
A_23_P10600	NFKBIA	NM_020529
A_32_P34980	TAP1	NM_000593
A_23_P58658	IL6ST	NM_002184
A_33_P33821	TIMP2	NM_003255
A_32_P45925	SP6	NM_199262
A_33_P32190	INSIG1	NM_005542
A_33_P32398	FBXL18	NM_024963
A_23_P11117	B3GALT4	NM_003782
A_24_P53525	INHBA	ENST00000242208
A_24_P41060	ROR1	NM_001083592
A_23_P11551	LCE3D	NM_032563
A_33_P32448	BEST4	NM_153274
A_23_P38569	WNT3A	NM_033131
A_33_P32675	VAX2	NM_012476
A_33_P33583	SIAH2	NM_005067
A_33_P33096	ZFP3	NM_153018
A_24_P11363	C6orf35	NM_018452
A_24_P41437	KLF3	NM_016531
A_23_P34209	TTTY5	NR_001541
A_23_P11592	EIF4EBP2	NM_004096
A_23_P25559	ZNF268	NM_152943

A_33_P32199[illegible]	EML1	NM_001008707
A_23_P21455[illegible]	TRIM15	NM_033229
A_33_P33857[illegible]	S100A12	NM_005621
A_23_P16341[illegible]	NIPA2	NM_030922
A_23_P16888[illegible]	TP53INP1	NM_033285
A_32_P47745[illegible]	TRIM24	ENST00000343526
A_23_P85509	IVL	NM_005547
A_23_P10740[illegible]	TIMP2	NM_003255
A_23_P52207	BAMBI	NM_012342
A_24_P33342[illegible]	ZNF862	NM_001099220
A_33_P34085[illegible]	SCNN1D	NM_001130413
A_23_P42142[illegible]	TNFAIP2	NM_006291
A_23_P50817	TTYH1	NM_020659
A_33_P32694[illegible]	FOXI2	NM_207426
A_24_P35891	ZNF219	NM_016423
A_23_P57779	CLDN1	NM_021101
A_23_P12044	TMEM51	NM_018022
A_24_P80204	MALL	NM_005434
A_23_P87709	PLBD1	NM_024829
A_23_P21336[illegible]	KIAA0141	NM_014773
A_23_P14502[illegible]	ADRB2	NM_000024
A_23_P11628[illegible]	AMPD3	NM_001025390
A_24_P91566	BMP7	NM_001719
A_33_P33345[illegible]	NDRG2	NM_201535
A_24_P36310[illegible]	RGMB	NM_001012761
A_23_P96383	SRPX	NM_006307
A_24_P18479[illegible]	COCH	NM_004086
A_23_P16913[illegible]	NINJ1	NM_004148
A_24_P18480[illegible]	COCH	NM_004086
A_33_P33420[illegible]	PRDM1	NM_001198
A_23_P16668[illegible]	AMOTL2	NM_016201
A_33_P32143[illegible]	FOXP1	NM_032682
A_23_P14734[illegible]	SLC16A3	U81800
A_33_P33962[illegible]	LRCH4	NM_002319
A_24_P17680[illegible]	ZNF264	NM_003417
A_23_P13276[illegible]	VGLL3	NM_016206
A_32_P73001	IL6R	ENST00000368485
A_23_P12976[illegible]	NOG	NM_005450
A_23_P47614	PHLDA2	NM_003311
A_23_P42330[illegible]	PCDH12	NM_016580
A_23_P17481	SIGLEC1	NM_023068
A_23_P65918	ITPKA	NM_002220
A_23_P94921	SLC20A2	NM_006749
A_23_P41021	NISCH	NM_007184
A_32_P78826[illegible]	ZNF571	ENST00000328550
A_23_P23924	CAPN2	NM_001748
A_24_P70383[illegible]	NANOS3	NM_001098622
A_23_P21647[illegible]	ZBTB5	NM_014872
A_33_P32885[illegible]	KRTAP10-11	NM_198692

A_33_P32985	SBNO2	NM_014963
A_23_P13798	S100A10	NM_002966
A_33_P32790	RIPK1	NM_003804
A_24_P30257	OSGEPL1	NM_022353
A_33_P32209	PCID2	NM_001127202
A_24_P37315	CFL2	NM_021914
A_23_P21482	EDN1	NM_001955
A_33_P33468	IL32	NM_001012633
A_23_P12686	GFRA1	NM_145793
A_33_P33381	LAMB3	NM_001017402
A_23_P13647	IRF2	NM_002199
A_33_P32814	YPEL5	NM_001127401
A_33_P32259	AJAP1	NM_001042478
A_23_P34567	ZNF71	NM_021216
A_23_P33463	JRK	NM_003724
A_23_P10196	ZFP36L2	NM_006887
A_23_P89780	LAMA3	NM_198129
A_33_P33111	TLX3	NM_021025
A_23_P40503	ZNF295	NM_020727
A_33_P33660	ADPRH	NM_001125
A_23_P14195	SERTAD3	NM_203344
A_23_P42838	COX17	NM_005694
A_23_P11920	CD70	NM_001252
A_23_P20210	PPIF	NM_005729
A_23_P91230	SLPI	NM_003064
A_33_P33821	PICALM	NM_007166
A_33_P32970	ALG2	NM_033087
A_33_P33102	PKIG	NM_181805
A_33_P33827	KDM5A	NM_001042603
A_32_P15860	MAF	NM_001031804
A_23_P94501	ANXA1	NM_000700
A_33_P34163	CHAF1A	NM_005483
A_33_P33942	GRIN3B	NM_138690
A_24_P34232	ZNF710	NM_198526
A_23_P16465	APOE	NM_000041
A_24_P11562	EIF4EBP2	NM_004096
A_23_P15392	MAP2	NM_002374
A_24_P44596	MON1B	NM_014940
A_23_P67169	IL11	NM_000641
A_24_P11762	CAMK2N1	NM_018584
A_32_P10700	RUNDC2A	NM_032167
A_23_P12823	KRT1	NM_006121
A_23_P14944	HES4	NM_021170
A_32_P40272	RORA	BC035094
A_24_P14209	AKAP13	NM_006738
A_23_P50275	MDM2	NM_002392
A_24_P24797	ZNF589	NM_016089
A_32_P17530	DENND3	NM_014957
A_23_P42514	C6orf62	NM_030939

A_33_P32926	TMPRSS11F	NM_207407
A_23_P16254	KCTD10	NM_031954
A_24_P13325	KITLG	NM_000899
A_23_P25392	ZNF167	NM_018651
A_33_P34027	ASH1L	NM_018489
A_23_P33823	BCDIN3D	NM_181708
A_33_P32881	RHOBTB3	NM_014899
A_23_P72737	IFITM1	NM_003641
A_33_P33171	TFAP2B	NM_003221
A_23_P99349	BTBD11	NM_001018072
A_24_P41312	PMEPA1	NM_020182
A_23_P21666	FOXE1	NM_004473
A_23_P15332	ICAM1	NM_000201
A_33_P32686	LY6D	NM_003695
A_24_P79808	PBXIP1	NM_020524
A_23_P31407	AGR2	NM_006408
A_23_P11858	ASB16	NM_080863
A_33_P32530	CRYAA	X14789
A_23_P30077	UBR1	NM_174916
A_23_P20397	FZD10	NM_007197
A_23_P51646	PLK3	NM_004073
A_32_P40617	FLG2	NM_001014342
A_23_P66732	GSG2	NM_031965
A_23_P25298	ACE2	NM_021804
A_23_P10380	FCRL3	NM_052939
A_23_P20225	RRM2B	NM_015713
A_23_P15872	SLC16A3	NM_001042422
A_24_P59403	ATP11A	NM_015205
A_24_P59786	GFAP	NM_001131019
A_32_P49199	PGR	NM_000926
A_23_P97339	SLC16A4	NM_004696
A_33_P33034	C19orf39	NM_175871
A_23_P23102	ZSCAN20	NM_145238
A_23_P15241	KIAA0182	NM_014615
A_33_P32759	SEL1L	NM_005065
A_23_P82868	PLAT	NM_000930
A_32_P22452	SLC25A23	NM_024103
A_24_P38122	SYVN1	NM_172230
A_23_P98930	C12orf35	NM_018169
A_23_P15628	OSMR	NM_003999
A_24_P97374	EOMES	NM_005442
A_33_P33062	PABPC1L2A	NM_001012977
A_23_P15283	CCL5	NM_002985
A_23_P20635	CDH1	NM_004360
A_33_P34044	RNF170	NM_001160224
A_23_P65617	TGM1	NM_000359
A_23_P35295	USP36	AK022913
A_23_P40809	MXD1	NM_002357
A_33_P33923	CDC16	NM_001078645

A_23_P51856	DUSP10	NM_007207
A_33_P33975	MEMO1	NM_015955
A_23_P25862	RTCD1	NM_003729
A_24_P12435	NCOA7	NM_181782
A_23_P51986	MTMR11	NM_181873
A_33_P32766	PGF	NM_002632
A_23_P58642	PITX1	NM_002653
A_23_P12513	SGSH	NM_000199
A_23_P30647	LOC10000967	NR_024407
A_23_P31810	CEBPD	NM_005195
A_23_P11914	ICAM5	NM_003259
A_24_P79631	EIF2C4	ENST00000373210
A_33_P32567	C9orf100	NM_032818
A_23_P42718	NFE2L3	NM_004289
A_24_P38863	PCGF3	NM_006315
A_23_P32253	NFIL3	NM_005384
A_32_P80413	LPPR5	NM_001010861
A_23_P35351	MSL3	NM_078628
A_24_P29658	DLX3	NM_005220
A_23_P21462	MGAT4C	NM_013244
A_23_P27649	ZNF433	NM_001080411
A_24_P41037	FTSJD1	NM_018348
A_23_P21487	JARID2	NM_004973
A_23_P25492	PTPRK	NM_002844
A_32_P36982	PGM5P2	NR_002836
A_23_P15367	TLE2	NM_003260
A_23_P42347	ETV7	NM_016135
A_33_P32130	STAT2	NM_005419
A_24_P30557	TBX5	NM_000192
A_33_P32335	RTN4	NM_020532
A_23_P23171	EIF2C4	NM_017629
A_33_P33703	TMEM106A	NM_145041
A_32_P99171	CHST11	NM_018413
A_24_P25847	SMOC1	NM_001034852
A_23_P74737	EYA3	NM_001990
A_23_P93348	LTB	NM_002341
A_32_P86400	LYSMD3	NM_198273
A_24_P20140	C11orf54	NM_014039
A_23_P10135	ZNF426	NM_024106
A_23_P33774	NRL	NM_006177
A_23_P14694	CST6	NM_001323
A_23_P14438	GALNT7	NM_017423
A_33_P32748	SPRR2F	NM_001014450
A_32_P47629	B3GALT4	NM_003782
A_33_P33045	CDX2	NM_001265
A_32_P45866	TNF	NM_000594
A_33_P32995	OVCH2	NM_198185
A_23_P43480	S100A8	NM_002964
A_23_P14171	C18orf10	NM_015476

A_23_P21010(	CYP26B1	NM_019885
A_23_P39856	IL1F5	NM_012275
A_24_P48627	PIAS4	ENST00000262971
A_33_P34035:	FCGR2A	NM_001136219
A_23_P85503	PGLYRP4	NM_020393
A_23_P37205	NDRG2	NM_201535
A_24_P36580:	EFNB1	NM_004429
A_33_P33330:	SUFU	NM_001178133
A_23_P49499	ST6GALNAC2	NM_006456
A_24_P80135	PTPN18	NM_014369
A_32_P23144(	HIPK1	NM_198268
A_24_P34132:	ZKSCAN2	NM_001012981
A_23_P30633	BACH2	NM_021813
A_24_P88004:	PCGF5	NM_032373
A_23_P15005:	ACTA2	NM_001613
A_33_P33785:	PDE5A	NM_001083
A_23_P41920:	ZNF658	NM_033160
A_23_P31348:	ABRA	NM_139166
A_23_P12608!	SPRR2C	NR_003062
A_24_P34537:	GOLPH3L	NM_018178
A_24_P10062:	ATRNL1	NM_207303
A_23_P11275(	GPSM1	NM_001145638
A_32_P16567:	ZNF135	AL157426
A_23_P35240:	C10orf47	NM_153256
A_24_P94121:	SGPP2	ENST00000321276
A_24_P22746	ZADH2	NM_175907
A_23_P25403	HCFC2	NM_013320
A_23_P36060!	KIAA0802	NM_015210
A_23_P7882	SLC22A23	NM_015482
A_23_P5901	SLCO4A1	NM_016354
A_23_P38269	HIST1H2BG	NM_003518
A_24_P29686	ZMIZ2	NM_031449
A_32_P18968(	KLHDC10	NM_014997
A_23_P10181:	ZNF14	NM_021030
A_23_P30023!	GAS2L3	NM_174942
A_23_P76654	CDX2	NM_001265
A_23_P11224:	DNAJB5	NM_012266
A_23_P23114	PTP4A2	NM_080391
A_24_P14478	HILS1	NR_024193
A_33_P34054	WASL	NM_003941
A_33_P34114:	NCCRP1	NM_001001414
A_23_P38106	SPHK1	NM_021972
A_23_P85619	GLUL	NM_002065
A_23_P75532	PKNOX2	NM_022062
A_33_P36355:	KRTAP24-1	NM_001085455
A_32_P10582!	MPPED2	NM_001584
A_33_P34239	IFITM1	NM_003641
A_24_P27385:	ZFPM2	NM_012082
A_23_P13809!	ABL2	NM_007314

A_23_P15545	LRRC2	NM_024512
A_23_P11201	DLC1	NM_024767
A_23_P45999	FBXO2	NM_012168
A_33_P33793	KRT1	NM_006121
A_24_P18999	PCSK6	NM_138322
A_23_P20829	PVRL2	NM_001042724
A_33_P32413	UCN3	NM_053049
A_23_P50252	IL4I1	NM_172374
A_23_P31393	PPIF	AY358854
A_32_P61654	ZNF33B	NM_006955
A_33_P33696	EIF3J	NM_003758
A_23_P11867	HOXB1	NM_002144
A_23_P40094	ETV3	NM_005240
A_33_P34199	SLC4A8	NM_001039960
A_23_P10361	ANXA9	NM_003568
A_23_P82169	SOX4	NM_003107
A_23_P7483	BDP1	NM_018429
A_23_P85783	PHGDH	NM_006623
A_32_P70811	PAX9	NM_006194
A_32_P20761	RUNDC2A	NM_032167
A_23_P43136	ZNF219	NM_016423
A_23_P43484	CDKN2A	NM_058197
A_23_P46837	TRIM8	NM_030912
A_33_P32927	NFAM1	NM_145912
A_33_P33094	PTPRU	NM_005704
A_23_P14320	UBE2C	NM_007019
A_33_P32900	HPS4	NM_022081
A_23_P20845	SLC25A23	NM_024103
A_24_P18502	SH2B1	NM_015503
A_23_P30015	NFATC1	NM_172387
A_23_P13584	PROP1	NM_006261
A_23_P32475	KIAA1199	NM_018689
A_23_P87941	ALDH1L2	NM_001034173
A_24_P41501	LRP12	NM_013437
A_23_P15275	PLEKHH3	NM_024927
A_23_P37391	CCDC85C	NM_001144995
A_33_P32711	NINJ1	NM_004148
A_23_P13094	MMP10	NM_002425
A_24_P36546	B4GALT5	NM_004776
A_32_P24965	ZFYVE26	NM_015346
A_32_P18226	PION	NM_017439
A_23_P20554	MAX	NM_145113
A_23_P46936	EGR2	NM_000399
A_23_P21394	HBEGF	NM_001945
A_33_P32604	SPRR2A	NM_005988
A_32_P34032	CSK	NM_004383
A_23_P15863	CEND1	NM_016564
A_23_P11959	EPHX3	NM_024794
A_23_P20993	TGOLN2	NM_006464

A_32_P53049	EIF1	NM_005801
A_23_P20516	POU4F1	NM_006237
A_33_P32402	CREBBP	NM_004380
A_24_P41630	FOXK2	NM_004514
A_23_P16975	HIPK2	NM_022740
A_32_P87013	IL8	NM_000584
A_32_P36378	ELK4	BX104627
A_23_P54140	BMP4	NM_001202
A_23_P85067	GPM6B	NM_001001995
A_23_P77668	ZNF821	NM_017530
A_23_P59397	RSPH3	NM_031924
A_23_P12823	NR4A1	NM_002135
A_23_P11269	PNMA2	NM_007257
A_32_P14699	POM121L1P	NR_024591
A_32_P16185	KIAA1199	NM_018689
A_23_P14332	BMP2	NM_001200
A_23_P21191	PLOD2	NM_182943
A_24_P13789	IFRD1	NM_001007245
A_33_P33655	SETD3	NM_199123
A_23_P11950	TNFAIP8L1	NM_001167942
A_23_P10721	HAP1	NM_177977
A_23_P21730	KDM6A	NM_021140
A_23_P14589	TP53TG1	NR_015381
A_24_P73075	TTC12	NM_017868
A_23_P21076	JAG1	NM_000214
A_24_P29501	SERPINB9	NM_004155
A_23_P32432	GPRC5B	NM_016235
A_23_P25150	HOXC9	NM_006897
A_33_P33850	ELK1	NM_001114123
A_23_P25352	CENPE	NM_001813
A_32_P3375	UBXN7	NM_015562
A_23_P10431	DDIT4	NM_019058
A_23_P16053	C1orf135	NM_024037
A_24_P20256	ITPKC	NM_025194
A_23_P13585	EIF2AK3	NM_004836
A_24_P37801	IRF7	NM_004031
A_33_P32875	BCAP29	NM_018844
A_23_P32560	ESX1	NM_153448
A_23_P35524	SAMD9	NM_017654
A_23_P91019	PRKRA	NM_003690
A_33_P33720	DDIT4L	NM_145244
A_23_P11264	ABO	NM_020469
A_24_P11920	MBD2	NM_015832
A_23_P62967	DISC1	NM_018662
A_23_P10892	MOBKL1B	ENST00000396049
A_33_P32795	HIPK3	NM_005734
A_23_P10688	FUS	NM_004960
A_24_P28322	MYO6	NM_004999
A_32_P92488	PKD1L2	NM_052892

A_23_P20201	ZEB1	NM_030751
A_23_P11494	RGS2	NM_002923
A_23_P21588	NCALD	NM_001040630
A_33_P33595	MYSM1	NM_001085487
A_32_P20237	NAV3	NM_014903
A_23_P42224	TRIM27	NM_006510
A_23_P10859	ANKLE2	NM_015114
A_23_P14074	NDRG4	NM_022910
A_23_P37424	SPATA5L1	NM_024063
A_24_P6889	PIAS3	NM_006099
A_24_P60523	KDM4C	NM_001146695
A_23_P39134	RASGEF1A	NM_145313
A_23_P42884	TMEM44	NM_138399
A_23_P25707	C7orf25	NM_024054
A_23_P16947	CDK9	NM_001261
A_33_P33257	SPRR2E	NM_001024209
A_23_P20880	CEBPG	NM_001806
A_23_P43255	FAM49B	NM_016623
A_24_P15149	PRMT2	NM_206962
A_33_P33578	SGCB	NM_000232
A_23_P26674	KIAA0430	NM_014647
A_33_P32184	CDC6	NM_001254
A_24_P23727	ADORA2A	NM_000675
A_33_P33357	ENST0000037	ENST00000377300
A_23_P93260	PRSS16	NM_005865
A_24_P11518	CLDN4	NM_001305
A_23_P15914	C1orf55	NM_152608
A_23_P45945	EIF1	NM_005801
A_23_P13468	HMBOX1	NM_024567
A_33_P32774	MLL5	NM_182931
A_23_P10842	GALNT5	ENST00000259056
A_23_P11279	ALG13	NM_018466
A_32_P22655	EXT1	ENST00000378204
A_32_P14049	GDF6	NM_001001557
A_24_P84340	MAST4	NM_198828
A_32_P15316	ISL2	NM_145805
A_23_P41800	LCE2A	NM_178428
A_23_P31292	POU2AF1	NM_006235
A_33_P32116	WDR3	NM_006784
A_23_P20055	USP48	NM_032236
A_23_P11405	SEMA4C	NM_017789
A_23_P12199	FAM46B	NM_052943
A_23_P85236	FAM127A	NM_001078171
A_32_P79036	ZNF90	NM_007138
A_23_P12117	EIF5A2	NM_020390
A_23_P31037	IFNK	NM_020124
A_23_P11162	GTF2IRD1	NM_005685
A_33_P33103	SLC7A8	NM_182728
A_23_P20666	NQO1	NM_000903

A_23_P7562	ACSL6	NM_001009185
A_23_P13253[illegible]	TRAK1	NM_001042646
A_23_P35859[illegible]	POPDC3	NM_022361
A_33_P33877[illegible]	USP9X	NM_001039590
A_23_P59637	DOCK4	NM_014705
A_32_P86163[illegible]	ELF3	NM_001114309
A_32_P52877[illegible]	DYNC1I1	NM_004411
A_23_P13186[illegible]	AURKA	NM_198433
A_23_P41143[illegible]	MTA3	AK127245
A_23_P21697[illegible]	TRAF1	NM_005658
A_23_P98763	LRTOMT	NM_145309
A_23_P12458[illegible]	ZNF93	NM_031218
A_23_P20281[illegible]	OVOL1	NM_004561
A_23_P50990	CENPO	NM_024322
A_32_P27917	KIF26A	NM_015656
A_23_P11733[illegible]	TNFAIP2	NM_006291
A_23_P13302[illegible]	TMPRSS11F	NM_207407
A_24_P11913[illegible]	TMEM120B	NM_001080825
A_32_P22073[illegible]	ZNF658	NM_033160
A_33_P33762[illegible]	PHTF1	NM_006608
A_23_P34570[illegible]	C15orf42	NM_152259
A_24_P96961	SPSB1	NM_025106
A_23_P44132	FASN	NM_004104
A_33_P33223[illegible]	SPRR2D	NM_006945
A_23_P66798	KRT19	NM_002276
A_23_P31092[illegible]	PCDH7	NM_002589
A_23_P16473[illegible]	MED26	NM_004831
A_23_P41986[illegible]	ENST0000031[illegible]	ENST00000318245
A_23_P33700	NET1	NM_001047160
A_32_P10474[illegible]	ZFYVE28	NM_020972
A_23_P30445[illegible]	GATA6	NM_005257
A_23_P25791[illegible]	USP16	NM_001032410
A_24_P64888[illegible]	MEIS3P1	NR_002211
A_23_P90211	IRF2BP1	NM_015649
A_23_P8221	SSR1	NM_003144
A_23_P13417[illegible]	SOD2	NM_001024465
A_23_P12918[illegible]	CALML4	NM_033429
A_23_P81248	TAF7	NM_005642
A_23_P15562[illegible]	CD80	ENST00000478182
A_23_P15272[illegible]	PLEKHM1	NM_014798
A_24_P27128[illegible]	NACC1	NM_052876
A_23_P17107[illegible]	ITM2A	NM_004867
A_32_P41848[illegible]	RPS6KA1	NM_002953
A_23_P25907[illegible]	AREG	NM_001657
A_24_P17299[illegible]	AARS	NM_001605
A_23_P11262	F8A1	NM_012151
A_23_P11046[illegible]	PCDHB1	NM_013340
A_23_P19944	CLDN4	NM_001305
A_24_P21826[illegible]	TNFRSF10B	NM_003842

A_33_P32151	ZNF777	NM_015694
A_24_P94028	PGS1	AL359590
A_24_P69476	ERC1	NM_178040
A_32_P64093	ZKSCAN2	NM_001012981
A_23_P20647	TRAF7	NM_032271
A_23_P21000	PAX8	NM_003466
A_23_P16674	EAF1	NM_033083
A_33_P34066	ZSCAN5B	NM_001080456
A_24_P29541	VCP	NM_007126
A_23_P47073	WDR37	NM_014023
A_23_P12607	KCNK1	NM_002245
A_23_P78358	PIGN	NM_176787
A_23_P36446	GPBP1	NM_022913
A_23_P20104	THBS3	NM_007112
A_23_P21009	SUPT7L	NM_014860
A_23_P31174	CUL9	NM_015089
A_23_P39265	LYPD3	NM_014400
A_24_P38006	ARHGAP24	NM_001025616
A_24_P39337	PACS2	NM_001100913
A_23_P13642	EPS8L2	NM_022772
A_32_P16846	CASK	NM_003688
A_24_P35526	SLC25A25	NM_001006641
A_23_P21740	USP9X	NM_001039590
A_23_P10692	CHST6	NM_021615
A_23_P12647	SSR2	NM_003145
A_24_P23748	MECP2	NM_004992
A_23_P12867	KCTD12	NM_138444
A_23_P84060	NTM	NM_016522
A_23_P15223	IRX3	NM_024336
A_24_P27461	ARRDC3	NM_020801
A_32_P83839	NFATC2	ENST00000371564
A_32_P13239	LOC10012555	NR_024251
A_23_P47991	MED13L	NM_015335
A_32_P36694	JAZF1	NM_175061
A_23_P38881	CKAP2L	NM_152515
A_24_P36407	HIPK3	ENST00000303296
A_23_P31091	BLMH	NM_000386
A_33_P33073	LPHN2	NM_012302
A_23_P25533	C4orf49	NM_032623
A_23_P12402	MED10	NM_032286
A_33_P34145	NR1I3	NM_001077474
A_23_P14587	SAMD9L	NM_152703
A_23_P63798	KLF6	NM_001300
A_23_P21651	C9orf100	NM_032818
A_33_P34054	IL4I1	NM_172374
A_23_P21795	HK1	NM_033500
A_23_P10250	SLC5A6	NM_021095
A_23_P38814	ZNF587	BC011243
A_23_P48835	KIF23	NM_138555

A_23_P13476	LY6D	NM_003695
A_33_P32666	ZBTB46	NM_025224
A_23_P24843	MICAL2	NM_014632
A_23_P12983	PPP1R1B	NM_032192
A_24_P38751	LRP5L	NM_182492
A_23_P21696	PTGS1	NM_000962
A_33_P33223	LOC654780	XR_111602
A_32_P18886	IL17RD	NM_017563
A_24_P18150	ZNF646	NM_014699
A_23_P75239	OPALIN	NM_001040103
A_23_P59005	TAP1	NM_000593
A_23_P10313	SMCR7L	NM_019008
A_33_P33444	NCR3	NM_001145467
A_23_P30223	SRD5A1	NM_001047
A_23_P21320	WHSC1	NM_133334
A_23_P94008	ADCK2	NM_052853
A_23_P12491	NPEPPS	NM_006310
A_23_P36032	AIM1L	NM_001039775
A_23_P13747	SPRR2F	NM_001014450
A_23_P60627	ALOX15B	NM_001141
A_23_P38585	MBNL3	NM_133486
A_33_P33426	ADAM33	NM_153202
A_23_P35506	TMCO1	NM_019026
A_23_P13431	C7orf11	NM_138701
A_32_P14821	TRAIP	NM_005879
A_23_P73517	CITED1	NM_004143
A_32_P45189	ZSCAN22	NM_181846
A_24_P50405	TMEM170A	ENST00000357613
A_33_P33816	ABLIM2	NM_001130088
A_23_P27315	EMILIN2	NM_032048
A_23_P10241	PLEKHA3	NM_019091
A_24_P30555	MCM8	NM_182802
A_23_P16463	ZNF419	NM_024691
A_33_P32549	PLEKHH3	NM_024927
A_24_P15296	AKR1C1	NM_001353
A_23_P88179	BTBD7	NM_018167
A_23_P25974	SATB1	NM_002971
A_23_P17065	CCL20	NM_004591
A_32_P15001	CHD7	NM_017780
A_33_P32277	PANK1	NM_148977
A_32_P32794	ITIH5L	NM_198510
A_23_P59470	ZNF467	BC038972
A_33_P32916	ZNF74	NM_003426
A_33_P33341	ZNF740	NM_001004304
A_23_P38235	ACE	NM_000789
A_33_P33480	CABP7	NM_182527
A_32_P39745	FAM111B	NM_198947
A_33_P33603	GATA3	NM_001002295
A_24_P18269	MYO3B	NM_138995

A_24_P12163:	ZNF764	NM_033410
A_32_P49468!	ZNF280C	NM_017666
A_23_P21536(	TAX1BP1	NM_006024
A_33_P32580-	TAC4	NM_170685
A_23_P30071(	SORBS3	AF037261
A_23_P21879:	XPNPEP3	NM_022098
A_32_P44446(	HMGN5	NM_030763
A_23_P16169{	MMP3	NM_002422
A_23_P20682:	XPO6	AK123846
A_33_P32497-	C8orf55	NM_016647
A_32_P33833(	TPRG1	ENST00000345063
A_24_P32175:	TTLL5	NM_015072
A_23_P36871{	C13orf1	NM_020456
A_23_P13168:	MAPRE3	NM_012326
A_23_P42812!	CDKN1C	NM_000076
A_23_P35415-	GALE	NM_000403
A_23_P43490(	HN1L	NM_144570
A_32_P52950{	C22orf39	NM_001166242
A_23_P41737:	ZSCAN2	NM_017894
A_24_P54178	TMED5	NM_016040
A_23_P14894:	GJA5	NM_005266
A_32_P18672:	ISM1	NM_080826
A_23_P10791:	KLK10	NM_002776
A_24_P92652!	MPHOSPH6	BC029395
A_23_P21339-	FLT4	NM_182925
A_24_P66648:	LRRC37A2	NM_001006607
A_23_P40771{	TRIM59	NM_173084
A_23_P82499	PEG10	NM_001040152
A_23_P11825-	FOXF1	NM_001451
A_23_P39766	GLS	NM_014905
A_23_P14524-	ZNF187	NM_001023560
A_23_P13164(	HOXD10	NM_002148
A_24_P40585(	PGRMC2	NM_006320
A_24_P26585(	SENP7	NM_020654
A_33_P33359:	HN1L	NM_144570
A_23_P25027-	LRRC8A	NM_019594
A_23_P37673!	ZNF524	NM_153219
A_23_P20973!	ARMC9	NM_025139
A_23_P15302(	GAA	NM_000152
A_23_P30521(	ZNF680	NM_178558
A_24_P13986:	TRIM62	NM_018207
A_23_P31094!	KIF1A	NM_004321
A_24_P68585	SHC1	NM_183001
A_23_P21433(	SERPINB1	NM_030666
A_33_P33722!	ABR	NM_001159746
A_23_P11698(	TULP3	AF045583
A_33_P33233{	RRM2B	NM_015713
A_23_P16847!	RALA	NM_005402
A_24_P17260(	ANKRD52	NM_173595

A_23_P52239	ADRB1	NM_000684
A_32_P37907	ZFP57	NM_001109809
A_32_P78381	LHX8	NM_001001933
A_24_P33231	FAM111B	NM_198947
A_23_P11667	PA2G4	NM_006191
A_24_P92534	MAN1C1	ENST00000374332
A_23_P63789	ZWINT	NM_032997
A_24_P59201	ZBTB46	ENST00000245663
A_23_P21731	FGF13	NM_004114
A_32_P44185	ZBTB7C	NM_001039360
A_24_P11424	GALNT3	NM_004482
A_33_P34053	KRTAP5-4	NM_001012709
A_33_P32195	NCRNA00207	NR_028409
A_23_P21027	MOBKL3	NM_015387
A_33_P32295	RASA4	NM_001079877
A_23_P30387	APOBEC3A	NM_145699
A_23_P30009	SLC10A7	NM_032128
A_23_P26843	SOX9	NM_000346
A_24_P6467	DENND2C	NM_198459
A_32_P18723	TMEM64	NM_001008495
A_23_P20833	PDE4A	NM_006202
A_23_P12527	CXCL11	NM_005409
A_33_P33914	ZNF672	NM_024836
A_24_P33349	FBXO42	NM_018994
A_23_P4662	BCL3	NM_005178
A_24_P18735	CNOT4	NM_013316
A_23_P32615	MNAT1	NM_002431
A_23_P72285	YWHAE	NM_006761
A_23_P14263	FKBP1B	NM_054033
A_33_P33567	NUDCD3	NM_015332
A_23_P25293	NCAPD2	NM_014865
A_23_P48755	SPATA7	NM_018418
A_32_P15827	MACC1	NM_182762
A_23_P10613	KTN1	NM_004986
A_23_P25974	TTC7B	NM_001010854
A_32_P50038	SIX5	NM_175875
A_33_P33172	MECP2	NM_001110792
A_32_P19185	SETD8	NM_020382
A_23_P37841	GPM6B	NM_001001996
A_23_P48585	SALL2	NM_005407
A_23_P55688	ZNF416	NM_017879
A_23_P35649	SPINK5	NM_006846
A_23_P10559	MVK	NM_000431
A_24_P91619	GTSE1	ENST00000160874
A_23_P16736	PITX2	NM_153426
A_23_P12570	NAP1L2	NM_021963
A_24_P21379	CCRN4L	NM_012118
A_23_P13408	CDYL	NM_004824
A_24_P23777	MAN1C1	NM_020379

A_23_P13065	ZDHHC13	NM_019028
A_23_P25487	PLAG1	NM_002655
A_23_P42949	C11orf82	NM_145018
A_32_P49326	SPDYC	NM_001008778
A_32_P47062	ZFP57	NM_001109809
A_23_P11057	MAST4	NM_001164664
A_23_P21694	PDCL	NM_005388
A_23_P11411	SCML2	NM_006089
A_33_P38617	MTMR10	NM_017762
A_33_P33089	CIB2	NM_006383
A_24_P91189	ALS2CR8	AB053310
A_23_P12094	XRCC6	NM_001469
A_33_P33347	MYOD1	NM_002478
A_23_P14132	GTF2F2	NM_004128
A_32_P48644	ZNF500	AB011129
A_33_P32438	IL11	NM_000641
A_33_P32843	NRG1	NM_004495
A_23_P20389	NCOR2	NM_006312
A_23_P17522	NKX2-2	NM_002509
A_23_P73012	C9orf3	NM_032823
A_23_P71480	DEFB1	NM_005218
A_24_P15658	LYSMD3	NM_198273
A_24_P23966	ZNF37A	NM_001007094
A_23_P33147	KIAA1949	NM_133471
A_23_P5059	GNA11	AL110227
A_23_P25544	DAPP1	NM_014395
A_33_P33686	HES7	NM_001165967
A_24_P36210	SKI	NM_003036
A_32_P21861	PFKM	NM_000289
A_24_P31845	E2F4	NM_001950
A_33_P32751	ZNF705G	NM_001164457
A_33_P33412	PCTK2	NM_002595
A_33_P33678	FRAT1	NM_005479
A_23_P16442	C17orf39	NM_024052
A_33_P32654	C6orf138	NM_001013732
A_23_P39676	PGM2L1	NM_173582
A_33_P33578	HTR1D	NM_000864
A_23_P11841	ATP2B4	NM_001684
A_23_P13096	MMP10	NM_002425
A_23_P61426	MSRA	NM_012331
A_23_P32588	TBC1D8B	NM_017752
A_24_P94126	CA5B	NM_007220
A_24_P27765	GMPR	NM_006877
A_24_P12397	TREM2	NM_018965
A_23_P81006	IL8	NM_000584
A_23_P33960	MYOCD	NM_153604
A_23_P37954	CCNF	NM_001761
A_24_P29669	MAP2K3	NM_145109
A_23_P21243	CTDSPL	NM_001008392

A_23_P16022	ZNF256	NM_005773
A_23_P39813	TTC31	NM_022492
A_23_P25303[illegible]	DMD	NM_004010
A_24_P39919	ZSCAN18	NM_023926
A_32_P71632[illegible]	XKR4	NM_052898
A_23_P74547	CD53	NM_001040033
A_23_P12024[illegible]	HOXD1	NM_024501
A_23_P12125[illegible]	TNFSF10	NM_003810
A_23_P42995[illegible]	KAL1	NM_000216
A_23_P13566[illegible]	SLCO5A1	NM_030958
A_23_P13785[illegible]	MUC1	NM_002456
A_23_P40975	EDEM1	NM_014674
A_23_P11278[illegible]	ARNT2	NM_014862
A_24_P12438	NCOA7	NM_181782
A_33_P32325[illegible]	KIF18B	NM_001080443
A_23_P13264[illegible]	NCEH1	NM_020792
A_23_P20201[illegible]	ZEB1	NM_030751
A_23_P14607[illegible]	ZNF395	NM_018660
A_23_P30184[illegible]	CALCA	NM_001033952
A_23_P20435[illegible]	ZNF384	NM_001039916
A_23_P86250	PIGC	NM_002642
A_23_P59285	GCM2	NM_004752
A_23_P85414	RERE	NM_012102
A_23_P75568	CARD17	AK128199
A_32_P50283	ARF4	NM_001660
A_23_P88781	CIAPIN1	NM_020313
A_23_P12756[illegible]	LAYN	NM_178834
A_32_P45263[illegible]	FOXL2	NM_023067
A_23_P25746[illegible]	GMPR	NM_006877
A_33_P33313[illegible]	TRIM25	NM_005082
A_23_P35173[illegible]	NPHP4	NM_015102
A_24_P92302[illegible]	TAT	BC020707
A_24_P23087[illegible]	MTDH	NM_178812
A_23_P17009[illegible]	ALK	U66559
A_23_P38322[illegible]	S100A1	NM_006271
A_33_P32237[illegible]	MED13	NM_005121
A_24_P25641[illegible]	LOC10012828[illegible]	NR_024447
A_23_P65618	TGM1	NM_000359
A_23_P13947[illegible]	CD63	NM_001780
A_32_P16346[illegible]	NFE2L1	NM_003204
A_23_P41737[illegible]	ZSCAN2	NM_017894
A_24_P11314[illegible]	ATAD5	NM_024857
A_24_P94013[illegible]	CTBS	NM_004388
A_24_P33170[illegible]	KRT80	NM_182507
A_32_P16534[illegible]	SRP9	NM_003133
A_32_P96692	POLH	NM_006502
A_32_P33391[illegible]	UAP1L1	NM_207309
A_32_P36191[illegible]	TBC1D2B	NM_015079
A_23_P13108[illegible]	KANK3	NM_198471

A_33_P32437(	KLHL20	NM_014458
A_23_P12196	FAM46B	NM_052943
A_23_P25559:	ZNF268	NM_003415
A_33_P34231	WDR6	NM_018031
A_23_P32239!	ZSCAN1	NM_182572
A_23_P89665	KRT33B	NM_002279
A_23_P33907!	ZNF573	NM_152360
A_32_P15136(	SIRT5	NM_001193267
A_24_P41398!	TGOLN2	NM_006464
A_23_P23048	S100A9	NM_002965
A_23_P20087	CCDC21	NM_022778
A_32_P22020	KIF19	NM_153209
A_24_P34685!	MKI67	NM_002417
A_23_P12173	CRTC2	NM_181715
A_32_P10866(	LPAR3	ENST00000440886
A_23_P25906!	PDE8B	NM_003719
A_23_P13264(	ST6GAL1	NM_173216
A_33_P32604:	SPRR2A	NM_005988
A_23_P14495!	VCAN	NM_004385
A_23_P73397	CSF1	NM_172212
A_23_P94552	TMEM2	NM_013390
A_32_P50476	ZBTB20	NM_015642
A_23_P69877	ZFP62	NM_152283
A_23_P91390	THBD	NM_000361
A_24_P28611	SLC1A3	NM_004172
A_23_P72297	SATB1	NM_002971
A_32_P38600	MCTP2	NM_018349
A_23_P32924	ZNF44	NM_001164276
A_23_P12626(	HLX	NM_021958
A_33_P32222	FUT6	NM_000150
A_24_P13481(	BCL9L	NM_182557
A_23_P81219	PLAC8	NM_016619
A_23_P12903	ATXN3	NM_004993
A_23_P13199(	VSX1	NM_014588
A_23_P37761(	CIRBP	NR_023312
A_23_P38765(	EPB41L4B	NM_019114
A_24_P20196(	CCDC85C	NM_001144995
A_23_P65651	WARS	NM_004184
A_23_P60393	NOTCH1	NM_017617
A_23_P15456(	TOX2	NM_032883
A_33_P32482(	LTB	NM_002341
A_23_P68624	POFUT1	NM_172236
A_24_P16101!	PARP14	NM_017554
A_33_P32718	PHLPP2	NM_015020
A_23_P96853	FAF1	NM_007051
A_24_P15505!	IKBKB	NM_001556
A_23_P38577	PAOX	NM_152911
A_23_P12343	GSTM3	NM_000849
A_23_P41539!	MBD3L1	NM_145208

A_23_P32738	TP63	NM_003722
A_23_P37836	PCDH7	NM_032457
A_24_P54174	TNFRSF1B	NM_001066
A_23_P17090	NUBPL	NM_025152
A_24_P97257	RPL31	ENST00000264258
A_24_P11436	TTC22	NM_017904
A_23_P21090	ACSS2	NM_018677
A_23_P41728	IGF1R	NM_000875
A_24_P10921	APOC1	NM_001645
A_24_P26415	CCDC113	NM_014157
A_23_P27472	ATP1A3	NM_152296
A_23_P8309	UST	NM_005715
A_23_P8116	ZBTB9	NM_152735
A_23_P89236	DNAH17	NM_173628
A_23_P15823	SHMT2	NM_005412
A_32_P91381	DCAF11	NM_025230
A_23_P57784	CLDN1	NM_021101
A_23_P82176	SOX4	NM_003107
A_24_P31204	PLAGL1	NM_006718
A_23_P63807	NRBF2	NM_030759
A_23_P36802	TP53I11	AF010315
A_24_P30796	SOHLH1	NM_001012415
A_23_P74731	EYA3	NM_001990
A_23_P25178	NAA16	NM_024561
A_24_P92982	ITGB8	NM_002214
A_33_P32411	XRCC2	NM_005431
A_23_P32921	ETS1	NM_005238
A_23_P90743	REG1A	NM_002909
A_23_P40996	RAPH1	ENST00000453034
A_32_P22355	BRWD1	ENST00000333229
A_23_P32285	C9orf91	NM_153045
A_24_P25017	MLX	NM_170607
A_23_P81999	TEAD3	NM_003214
A_23_P10383	UBAP2L	NM_001127320
A_32_P10620	SKAP1	NM_003726
A_23_P21867	WFDC2	NM_006103
A_24_P76675	MFAP3L	NM_021647
A_24_P22446	TRPV3	NM_145068
A_32_P69492	POLH	NM_006502
A_24_P23542	ABCA1	NM_005502
A_23_P17739	ZNF74	NM_003426
A_24_P93765	TRIM42	AL833848
A_23_P20772	ALOXE3	NM_021628
A_24_P82358	FOXC2	NM_005251
A_23_P21752	KLF8	NM_007250
A_32_P46510	ZC3H6	NM_198581
A_23_P20031	DEPDC1	NM_017779
A_24_P12034	NIT2	NM_020202
A_23_P11138	ATG5	NM_004849

A_23_P385206	STX12	NM_177424
A_32_P429028	C20orf26	NM_015585
A_24_P155169	KCNT1	NM_020822
A_32_P857658	RPLP1	NM_001003
A_32_P430896	ZNF462	NM_021224
A_23_P27381	TSHZ1	NM_005786
A_24_P246107	KRTAP13-4	NM_181600
A_23_P318604	CYHR1	BC004544
A_23_P399201	TMSL3	NM_183049
A_33_P337042	NOTCH1	NM_017617
A_24_P82142	TCF12	NM_207038
A_23_P38446	TNFAIP1	NM_021137
A_23_P90233	ZSCAN5A	NM_024303
A_23_P400298	PRSS22	NM_022119
A_23_P68610	TPX2	NM_012112
A_24_P76267	GGT1	NM_005265
A_33_P338806	ZNF735	NM_001159524
A_23_P40025	DAZAP2	NM_014764
A_23_P71627	GLIPR2	NM_022343
A_23_P45436	EFNB1	NM_004429
A_33_P325142	KRTAP21-1	NM_181619
A_24_P508012	TAF3	ENST00000344293
A_23_P368187	NAB2	NM_005967
A_32_P353096	C6orf48	NM_001040437
A_33_P342085	KIRREL2	NM_199180
A_23_P415663	TIGD3	NM_145719
A_23_P120103	KCNS3	NM_002252
A_24_P204614	FOXD4	NM_207305
A_24_P154573	ZBTB49	NM_145291
A_33_P372019	LOC375196	NR_028386
A_23_P99891	MESDC1	NM_022566
A_23_P95130	SLC37A3	NM_207113
A_32_P485808	P2RY2	NM_176072
A_23_P61826	BCL7A	NM_020993
A_23_P79323	CDK5R2	NM_003936
A_24_P207503	MEIS3	NM_001009813
A_33_P333773	FLJ45831	XR_109367
A_23_P70660	FAM46A	NM_017633
A_23_P169576	EXOC6	NM_019053
A_23_P50815	TTYH1	NM_020659
A_33_P322112	LRRN4CL	NM_203422
A_32_P200238	UCA1	NR_015379
A_23_P865	FRRS1	NM_001013660
A_33_P324177	TUBB4Q	NM_020040
A_33_P331440	CLDN16	NM_006580
A_23_P110430	MSX1	NM_002448
A_23_P105100	IFITM2	NM_006435
A_32_P86763	TGM2	NM_004613
A_33_P327719	CHST10	NM_004854

A_24_P13915	COL8A1	ENST00000261037
A_23_P20132	PDPN	NM_006474
A_32_P16292	ARHGAP11B	NM_001039841
A_23_P50100	EFEMP1	NM_004105
A_23_P11963	UPK1A	NM_007000
A_23_P29994	RBPJ	NM_203284
A_23_P23303	EXO1	NM_003686
A_33_P32562	KRTAP10-2	NM_198693
A_23_P33173	GOLGB1	X75304
A_23_P16161	POLA2	NM_002689
A_23_P15221	E2F4	NM_001950
A_23_P20485	RB1	NM_000321
A_33_P33346	RPA2	NM_002946
A_23_P31224	PILRA	NM_178273
A_23_P80635	IRAK2	NM_001570
A_24_P35985	HDAC4	NM_006037
A_24_P23821	C2CD3	NM_015531
A_23_P91187	GMEB2	NM_012384
A_23_P78201	KRT35	NM_002280
A_33_P33361	TIGD3	NM_145719
A_23_P32082	C14orf145	NM_152446
A_32_P52126	IRX6	NM_024335
A_23_P41683	ZNF771	AF242768
A_23_P20807	IMPACT	NM_018439
A_23_P12108	GBE1	NM_000158
A_24_P28027	S100A7A	NM_176823
A_23_P11519	NGF	NM_002506
A_23_P76815	COCH	NM_004086
A_24_P22112	PA2G4	NM_006191
A_23_P11110	RNF39	NM_025236
A_24_P35594	EFNB2	NM_004093
A_24_P20112	ZNF510	NM_014930
A_23_P39067	SPIB	NM_003121
A_32_P31302	MSRA	NM_012331
A_33_P32621	APOBEC3F	NM_001006666
A_33_P33798	FGF2	NM_002006
A_33_P33590	IRGC	NM_019612
A_23_P30621	FAM84A	NM_145175
A_23_P13047	ZNF8	NM_021089
A_24_P18312	PLAC8	NM_016619
A_33_P33615	TFAP2A	NM_001032280
A_33_P32324	CLIP4	NM_024692
A_33_P32904	IMPA2	NM_014214
A_33_P33843	ADRBK1	NM_001619
A_23_P34139	CYB5D2	NM_144611
A_23_P1602	CDC42EP2	NM_006779
A_23_P17998	HES1	NM_005524
A_23_P77887	PITPNA	NM_006224
A_33_P33683	PHB2	NM_007273

A_23_P2942	NPAS3	NM_022123
A_23_P33487	TMEM217	NM_145316
A_24_P27658	TMCO1	NM_019026
A_23_P45799	ORC1L	NM_004153
A_33_P33235	CRYAA	NM_000394
A_33_P33732	CACNA2D3	NM_018398
A_23_P21674	PAPPA	NM_002581
A_24_P30279	PCCB	NM_000532
A_33_P33343	ZXDB	NM_007157
A_23_P79130	PLEKHF1	NM_024310
A_23_P92871	RXFP3	NM_016568
A_23_P16652	ZNF555	NM_152791
A_23_P51132	IL1RL1	NM_016232
A_23_P21134	TBX1	NM_080647
A_23_P88602	MEIS2	NM_170677
A_33_P32377	PDLIM4	NM_003687
A_23_P92642	ANKHD1	NM_024668
A_23_P66928	RTTN	NM_173630
A_33_P33985	BLZF1	NM_003666
A_33_P32611	GNS	NM_002076
A_32_P16339	APLF	NM_173545
A_24_P91192	RAP1GAP2	NM_015085
A_33_P33973	ZNF441	NM_152355
A_23_P75523	CD59	NM_203330
A_23_P42821	EZH1	NM_001991
A_24_P36595	THAP11	NM_020457
A_23_P32392	ZFYVE27	NM_001002261
A_24_P92235	C22orf39	NM_173793
A_24_P92018	YIPF6	NM_173834
A_23_P20596	PML	NM_033238
A_32_P19237	ENPP1	NM_006208
A_23_P12097	TTLL1	NM_012263
A_23_P14439	CSN2	NM_001891
A_24_P48177	ST3GAL2	NM_006927
A_23_P36546	HOXC10	NM_017409
A_23_P25065	TLN2	NM_015059
A_24_P10948	PLEKHA7	NM_175058
A_24_P56134	FAM150B	NM_001002919
A_33_P32646	CYP27C1	NM_001001665
A_23_P20239	SUV39H2	NM_024670
A_32_P10900	SMAD2	NM_001003652
A_24_P40808	DNMT1	NM_001379
A_33_P32103	RFX7	NM_022841
A_33_P33845	POMGNT1	NM_017739
A_23_P39491	SRCAP	NM_006662
A_23_P25145	HNF4G	NM_004133
A_23_P35006	C1orf152	NR_003242
A_32_P27020	NBPF15	NM_173638
A_23_P25450	HOPX	NM_139211

A_24_P399942	ATP11C	NM_173694
A_32_P211718	PLAUR	NM_001005376
A_33_P342340	ZNF790	NM_206894
A_23_P397285	LY6K	NM_017527
A_23_P201551	VAV3	NM_006113
A_23_P159775	GABRE	NM_004961
A_33_P330212	LCE2A	NM_178428
A_24_P55391	HIP1	ENST00000336926
A_23_P414391	CDC42EP5	NM_145057
A_23_P99036	ACVR1B	NM_004302
A_23_P102927	PWP2	NM_005049
A_33_P332061	HDAC6	NM_006044
A_23_P26916	HDAC5	NM_001015053
A_32_P27763	GTF2H5	NM_207118
A_33_P335947	SUV420H2	NM_032701
A_32_P478787	SREBF1	NM_001005291
A_33_P326730	ANKRD52	NM_173595
A_23_P117494	MTHFD1	NM_005956
A_32_P169679	LCORL	AL133031
A_33_P331179	ZNF749	NM_001023561
A_33_P340770	TMEM192	NM_001100389
A_23_P413456	CIRBP	NR_023312
A_23_P315815	NRG1	NM_004495
A_33_P360821	LOC554202	NR_027054
A_33_P339446	PROP1	NM_006261
A_23_P142849	RND3	NM_005168
A_23_P320200	RNF5	NM_006913
A_23_P32903	OCRL	NM_000276
A_23_P212617	TFRC	NM_003234
A_23_P348038	FOLR3	NM_000804
A_23_P124873	SCRIB	NM_182706
A_24_P38316	FOXP2	NM_148898
A_24_P312915	CDK11B	NM_033489
A_33_P330129	FOXB2	NM_001013735
A_23_P64489	RIC8A	NM_021932
A_23_P13679	KLHDC5	NM_020782
A_23_P118705	SDF2	NM_006923
A_24_P918147	FBXO32	ENST00000517956
A_23_P200288	S100A8	NM_002964
A_24_P167970	TBX6	NM_004608
A_23_P160881	SMPDL3B	NM_001009568
A_24_P54808	KLHL8	NM_020803
A_32_P230338	SLC35B2	NM_178148
A_32_P536917	ANKRD52	NM_173595
A_23_P205757	DLK1	NM_003836
A_32_P209960	CIITA	NM_000246
A_32_P142818	DLX1	NM_178120
A_32_P493970	ZNF621	NM_198484
A_23_P114314	CDX4	NM_005193

A_23_P16419(	DLX4	NM_138281
A_23_P12192(	SEPP1	NM_005410
A_24_P53465	CLEC4M	NM_001144904
A_23_P11866(	DDX52	NM_007010
A_33_P32497(	CYP3A5	NM_000777
A_23_P11406:	TRPM3	NM_001007471
A_23_P25398:	HOXA4	NM_002141
A_23_P13387(	MOG	NM_206814
A_23_P2383	CELA1	NM_001971
A_32_P50825(	EIF2B5	NM_003907
A_23_P15521!	CSPG5	NM_006574
A_23_P12165:	HS3ST1	NM_005114
A_23_P20049:	LBR	NM_002296
A_24_P25734(	ARL6IP5	NM_006407
A_23_P34133	WBP5	NM_016303
A_24_P11945(	STAB1	NM_015136
A_32_P13401:	XKR4	NM_052898
A_23_P95302	RFC5	NM_181578
A_32_P16271(	ZSWIM6	CB529149
A_23_P32387:	IKZF2	NM_001079526
A_23_P14443(	DCAF16	NM_017741
A_24_P35625(	CTCF	NM_006565
A_23_P63660	C10orf58	NM_032333
A_23_P21084!	PYGB	NM_002862
A_23_P11017!	CTSO	NM_001334
A_23_P41489!	TTC17	NM_018259
A_23_P50027:	IRF5	NM_001098627
A_24_P28778(	CHMP1A	NM_002768
A_23_P12832:	SCNN1A	NM_001038
A_23_P41502:	METTL7A	NM_014033
A_24_P30364:	C7orf60	NM_152556
A_32_P10003	TUBA3D	NM_080386
A_32_P37811(	KIAA1949	NM_133471
A_24_P15626:	SOX12	NM_006943
A_23_P9662	IPP	NM_005897
A_23_P50320(	PHF10	NM_018288
A_23_P17330	UCKL1	NM_017859
A_23_P96249	CELF4	NM_020180
A_23_P90155	PIH1D1	NM_017916
A_23_P39499(	SLC2A12	NM_145176
A_23_P14596!	TPST1	NM_003596
A_23_P41363(	ZNF329	NM_024620
A_23_P10361(	ANXA9	NM_003568
A_24_P35390!	MXRA8	NM_032348
A_23_P12562(	ACOT9	NM_001037171
A_33_P33545(	GPD2	NM_001083112
A_23_P3681	NETO2	NM_018092
A_24_P10209:	ZDHHC21	AF161360
A_23_P15737:	FAM3C	NM_014888

A_24_P18117	ERC1	NM_178040
A_23_P37127	FOXA1	NM_004496
A_24_P52697	H19	NR_002196
A_23_P21124	PRMT2	NM_206962
A_23_P13703	PIR	NM_003662
A_23_P27035	TMEM98	NM_015544
A_23_P21921	OGT	NM_181672
A_23_P16340	CYP1A1	NM_000499
A_24_P27721	HRH1	NM_000861
A_23_P16155	ARFGAP2	NM_032389
A_23_P25575	ATAD5	NM_024857
A_24_P11889	IRF7	NM_004029
A_24_P36923	CCDC3	NM_031455
A_23_P78144	MED31	NM_016060
A_23_P14474	ZNF454	NM_182594
A_23_P13655	PIGG	NM_017733
A_23_P21544	BAZ1B	NM_032408
A_32_P18886	IL17RD	NM_017563
A_24_P17950	WDR66	NM_144668
A_24_P25249	TRIB1	NM_025195
A_23_P21085	GINS1	NM_021067
A_23_P25182	IFRD1	NM_001007245
A_23_P15250	ABAT	NM_000663
A_33_P32240	DAPK3	NM_001348
A_23_P34877	RBM15	NM_022768
A_23_P34433	ZCCHC11	NM_001009881
A_24_P30345	TIAM2	NM_012454
A_24_P92696	MEGF6	NM_001409
A_24_P31357	VAMP2	NM_014232
A_23_P16658	LRRC15	NM_130830
A_23_P41701	ZNF311	NM_001010877
A_32_P42100	VPS8	NM_001009921
A_23_P90888	CHRNA1	NM_001039523
A_23_P20696	SEC14L1	NM_003003
A_23_P12530	IGSF1	NM_001555
A_23_P48964	VPS33B	NM_018668
A_23_P21335	PCDH1	NM_002587
A_24_P20042	PAICS	NM_001079525
A_33_P33262	ESCO2	NM_001017420
A_32_P14975	CEP152	NM_014985
A_24_P26483	NEFM	NM_005382
A_23_P65733	ZNF770	NM_014106
A_23_P55606	ZNF516	NM_014643
A_23_P14231	MKNK2	NM_017572
A_32_P16435	ESRRA	NM_004451
A_24_P89887	C9orf3	NM_032823
A_24_P37451	TMSB4X	NM_021109
A_23_P40711	PIP4K2B	NM_003559
A_23_P14176	NAPG	NM_003826

A_24_P27371	ZBTB24	NM_014797
A_23_P42619	MAST3	NM_015016
A_33_P33294	STYXL1	NM_016086
A_33_P33484	PCID2	NM_001127202
A_24_P2338	XPNPEP3	NM_022098
A_23_P37783	ANAPC5	NM_016237
A_24_P20719	IRX3	NM_024336
A_23_P13678	GINS4	NM_032336
A_23_P16636	PRAME	NM_206956
A_23_P39746	GDF7	ENST00000272224
A_23_P6087	SLC23A2	NM_203327
A_33_P32734	POLR3C	NM_006468
A_23_P20054	ZC3H12A	NM_025079
A_33_P33152	KRT77	NM_175078
A_23_P24903	P2RY2	NM_176072
A_33_P32913	INO80	NM_017553
A_23_P39176	RAPGEF1	NM_198679
A_23_P25480	PLCG1	NM_002660
A_24_P70002	LATS2	NM_014572
A_23_P13678	GINS4	NM_032336
A_23_P12857	CDK8	NM_001260
A_23_P39362	TFPI2	NM_006528
A_23_P76851	PRMT5	NM_001039619
A_23_P92719	RAI14	NM_015577
A_24_P36534	CACNG7	ENST00000391767
A_23_P10434	PIP4K2A	NM_005028
A_24_P41040	KRT83	NM_002282
A_24_P82186	SCAND2	NR_004859
A_23_P25764	RBP1	NM_002899
A_23_P86133	RPA2	NM_002946
A_33_P32247	PLA2G15	NM_012320
A_23_P87011	TAGLN	NM_001001522
A_23_P90612	MCM6	NM_005915
A_23_P16346	C15orf52	NM_207380
A_23_P64743	STK38L	NM_015000
A_23_P10818	GRIN3B	NM_138690
A_24_P41373	DNAJC11	NM_018198
A_33_P36639	THRB	NM_001128177
A_33_P32735	KRT84	NM_033045
A_33_P34007	PLIN4	NM_001080400
A_24_P97129	SDC1	NM_001006946
A_23_P13789	ARHGEF19	NM_153213
A_24_P16300	LOC151009	NR_027244
A_23_P16886	ZNF16	NM_001029976
A_24_P33644	GUK1	NM_000858
A_23_P12668	USP21	NM_001014443
A_23_P10741	P4HB	NM_000918
A_23_P17053	IL1F9	NM_019618
A_24_P16534	NFYC	NM_014223

A_23_P21882	POLQ	NM_199420
A_23_P52240	ADRB1	NM_000684
A_33_P32681	ZNF654	NM_018293
A_32_P37351	ERAP2	NM_022350
A_24_P15657	GEMIN8	NM_017856
A_33_P32975	IRX2	NM_033267
A_33_P32098	PLXDC1	NM_020405
A_24_P91202	SOX6	NM_017508
A_24_P33675	MCL1	NM_021960
A_32_P90686	NEGR1	NM_173808
A_32_P39954	ARNTL2	AF256215
A_33_P33690	BCAR1	NM_014567
A_23_P13921	H19	NR_002196
A_32_P45844	SLC8A2	NM_015063
A_23_P13968	ERP27	NM_152321
A_23_P64038	FERMT3	NM_178443
A_23_P25727	CDK5RAP1	NM_016082
A_23_P21474	SIM1	NM_005068
A_24_P28938	CHD7	NM_017780
A_24_P30258	SOX11	NM_003108
A_23_P15724	TMEM184A	NM_001097620
A_23_P4649	APOC1	NM_001645
A_23_P13754	ZNF362	NM_152493
A_23_P57306	CHAF1B	NM_005441
A_33_P34240	PEG3	NM_006210
A_23_P96461	GEMIN8	NM_017856
A_23_P33664	KRT72	NM_080747
A_23_P34045	EDA	NM_001399
A_23_P11382	NACC2	NM_144653
A_33_P34005	MED21	NM_004264
A_32_P52953	SCN4B	NM_174934
A_23_P35859	POPDC3	NM_022361
A_23_P66682	HOXB6	NM_018952
A_23_P51627	PPCS	NM_024664
A_23_P38696	DSC1	NM_004948
A_24_P38301	KRTAP13-1	NM_181599
A_23_P7033	MAGEF1	NM_022149
A_23_P21234	CCR5	NM_000579
A_32_P45102	ZSCAN23	NM_001012455
A_32_P10992	ZFAT	NM_020863
A_24_P53824	RALGPS1	NM_014636
A_23_P74926	LCE2B	NM_014357
A_23_P30187	MED27	NM_004269
A_24_P27610	RBL1	NM_183404
A_32_P20069	FAM101A	NM_181709
A_23_P31661	GLIS1	NM_147193
A_23_P40333	EXPH5	NM_015065
A_24_P94359	PHLDA1	NM_007350
A_24_P22211	N4BP2	NM_018177

A_24_P13358	MFGE8	NM_005928
A_23_P15823	APBA2	NM_005503
A_23_P13142	VAX2	NM_012476
A_23_P63371	TAL1	NM_003189
A_32_P98283	SFMBT2	NM_001029880
A_23_P37954	YARS	NM_003680
A_23_P15711	CREB5	NM_182898
A_33_P33531	CLTC	BC051800
A_23_P11247	NUDT2	NM_001161
A_24_P22813	CCL3L3	NM_001001437
A_24_P52964	H6PD	NM_004285
A_24_P25470	ZNF695	NM_020394
A_32_P49345	SPCS2	NM_014752
A_23_P10887	ZC3H8	NM_032494
A_32_P22238	HMGN2	NM_005517
A_23_P41058	PHF17	NM_024900
A_32_P20481	GUSBL1	NR_003504
A_23_P16965	WBSCR16	NM_030798
A_23_P11701	SFRS9	NM_003769
A_23_P20152	PQLC2	NM_001040125
A_23_P34733	FH	NM_000143
A_32_P73694	ATAD5	NM_024857
A_32_P13031	SATB2	NM_015265
A_32_P76115	ATP5H	NM_006356
A_33_P32713	TCF20	NM_181492
A_23_P22027	INSIG1	NM_198336
A_33_P33199	KDM4B	NM_015015
A_23_P31602	BUD31	NM_003910
A_24_P71235	CHML	NM_001821
A_23_P61009	PRIM2	NM_000947
A_23_P11248	AQP3	NM_004925
A_23_P21042	MYL9	NM_181526
A_23_P64372	TCN1	NM_001062
A_23_P6023	RAE1	ENST00000371242
A_24_P82634	ZC3H6	NM_198581
A_24_P92123	PEX14	BC017848
A_23_P13556	TNIK	ENST00000436636
A_23_P63167	CD1A	NM_001763
A_23_P16195	LRRN4CL	NM_203422
A_24_P49865	KCNA1	NM_000217
A_23_P37648	TNF	NM_000594
A_23_P10303	CRYBA4	NM_001886
A_33_P33991	PI4KA	NM_058004
A_23_P14334	OVOL2	NM_021220
A_32_P74357	LOC10013216	BC006438
A_33_P33848	POFUT1	NM_172236
A_24_P21376	PPM1F	CR625942
A_32_P92654	GUSBP1	NR_027028
A_33_P32464	MDFI	NM_005586

A_23_P17113[illegible]	EDA2R	NM_021783
A_23_P2674	KRT4	NM_002272
A_23_P65930	ZFYVE19	NM_001077268
A_24_P20058[illegible]	UBE2B	NM_003337
A_24_P32202[illegible]	CREBBP	NM_004380
A_32_P12996[illegible]	ZNF284	ENST00000328297
A_23_P2344	HOXC6	NM_153693
A_23_P75647	HYOU1	NM_006389
A_23_P21415[illegible]	SENP6	NM_015571
A_33_P32301[illegible]	SLITRK6	NM_032229
A_23_P32883[illegible]	LCOR	NM_032440
A_23_P33364	SH3D19	NM_001009555
A_23_P21846[illegible]	SERTAD1	NM_013376
A_23_P77880	PITPNA	NM_006224
A_23_P32541[illegible]	RREB1	ENST00000379938
A_33_P33527[illegible]	MC1R	NM_002386
A_32_P50272[illegible]	THAP11	NM_020457
A_23_P38029[illegible]	ProSAPiP1	NM_014731
A_23_P35127[illegible]	PAPPA	NM_002581
A_23_P97159	IPO9	NM_018085
A_23_P50262[illegible]	ZXDA	NM_007156
A_23_P14566[illegible]	EPO	NM_000799
A_24_P23100[illegible]	TIMELESS	NM_003920
A_32_P13082[illegible]	BMS1	NM_014753
A_23_P7301	WHSC1	NM_133334
A_23_P10825[illegible]	SBSN	NM_198538
A_24_P39813[illegible]	USP6NL	NM_014688
A_23_P15390[illegible]	IER2	NM_004907
A_23_P33199[illegible]	STX6	AK056657
A_23_P54900	UBN1	NM_016936
A_23_P2211	KRR1	NM_007043
A_23_P36101[illegible]	TSHZ3	NM_020856
A_24_P89872	ZNF189	NM_197977
A_32_P16826[illegible]	PPP1R15B	NM_032833
A_33_P33753[illegible]	SNAPC2	NM_003083
A_23_P78248	KRT23	NM_015515
A_23_P21117[illegible]	UBE2G2	NM_182688
A_23_P15638[illegible]	FNIP1	NM_133372
A_23_P15911[illegible]	SLC35E3	NM_018656
A_24_P35430[illegible]	WDR51A	NM_015426
A_23_P35912	CASP4	NM_033306
A_23_P13031[illegible]	PIAS2	NM_004671
A_23_P17054[illegible]	KCNH6	NM_173092
A_23_P41129[illegible]	CEBPB	NM_005194
A_24_P94184[illegible]	GMPPB	ENST00000308375
A_23_P10401	PPP2R3A	NM_002718
A_23_P15682[illegible]	C6orf105	NM_032744
A_32_P19701[illegible]	RFC3	NM_181558
A_23_P12035[illegible]	ANKRD57	NM_023016

A_32_P51150	TRIML2	NM_173553
A_33_P33010	CPOX	NM_000097
A_23_P10647	CYTL1	NM_018659
A_23_P68824	SMARCB1	NM_003073
A_32_P61684	PAG1	NM_018440
A_23_P11999	VRK2	NM_006296
A_33_P33474	RPS6KA2	NM_021135
A_23_P16271	DIAPH3	NM_030932
A_32_P19040	NCRNA00292	NR_027285
A_32_P16827	FARS2	NM_006567
A_23_P21320	WHSC1	NM_133334
A_23_P66664	TADA2A	NM_133439
A_33_P33789	ARHGEF18	NM_001130955
A_23_P12683	TNFSF4	NM_003326
A_23_P34376	TCEA3	NM_003196
A_33_P32685	PCK2	NM_001018073
A_33_P33095	SEBOX	NM_001080837
A_23_P92710	RHOBTB3	NM_014899
A_33_P33087	RXRG	NR_033824
A_23_P31922	ASS1	NM_000050
A_23_P31414	UST	NM_005715
A_23_P99044	KRT71	NM_033448
A_24_P10878	SOX10	NM_006941
A_23_P41358	FOXD2	NM_004474
A_23_P50942	RAB3GAP1	NM_012233
A_24_P34259	RERE	NM_012102
A_23_P21552	OSBPL3	NM_015550
A_23_P44942	CCDC15	NM_025004
A_33_P33165	SLC7A2	NM_001008539
A_23_P15036	REXO2	NM_015523
A_33_P34090	CNTN1	NM_001843
A_23_P93629	TRIM24	NM_015905
A_23_P12523	TP53BP2	NM_005426
A_23_P21426	GPR110	NM_153840
A_33_P32163	FTSJ1	NM_177439
A_23_P67971	GALM	NM_138801
A_24_P82954	LOC285074	NR_026846
A_24_P18531	NAPG	NM_003826
A_23_P31287	BMPER	NM_133468
A_23_P36979	MYO16	NM_015011
A_23_P14203	RFX1	NM_002918
A_23_P37727	CX3CL1	NM_002996
A_33_P33773	TCF7L2	NM_001146274
A_33_P32449	PDE8A	NM_002605
A_24_P39792	CTSB	NM_147780
A_23_P40186	CDH22	NM_021248
A_33_P32172	ZNF91	NM_003430
A_23_P20376	LGR4	NM_018490
A_33_P33680	SLC35B2	NM_178148

A_33_P33793[illegible]	MARS	NM_004990
A_23_P6771	LMCD1	NM_014583
A_23_P10009[illegible]	ZSCAN29	NM_152455
A_24_P16525[illegible]	PYCR2	NM_013328
A_33_P32150[illegible]	NSMCE1	NM_145080
A_23_P42042[illegible]	COMMD7	NM_053041
A_23_P37949	THOC6	NM_024339
A_23_P82299	C7orf47	NM_145030
A_33_P32913[illegible]	MSGN1	NM_001105569
A_33_P32583[illegible]	EDN1	NM_001955
A_24_P29296[illegible]	CDK5RAP3	NM_176096
A_33_P34004[illegible]	STIL	NM_001048166
A_23_P10692[illegible]	CHST6	NM_021615
A_24_P16340[illegible]	TRIM27	NM_006510
A_33_P34230[illegible]	NRL	NM_006177
A_23_P54144	BMP4	NM_001202
A_33_P33157[illegible]	ZNF382	NM_032825
A_24_P93527[illegible]	ZNF766	NM_001010851
A_24_P34926[illegible]	KRT80	NM_182507
A_23_P16624[illegible]	RCAN1	NM_004414
A_23_P15648[illegible]	IL17A	NM_002190
A_23_P85140	TCEAL2	NM_080390
A_23_P76914	SIX1	NM_005982
A_23_P20087[illegible]	CCDC21	NM_022778
A_33_P33511[illegible]	TYSND1	NM_173555
A_24_P14303[illegible]	CDC42EP1	NM_152243
A_24_P89718	TTC26	NM_024926
A_24_P25222[illegible]	SLC22A23	NM_021945
A_33_P33366[illegible]	CLIC3	NM_004669
A_23_P12502[illegible]	PECAM1	NM_000442
A_23_P30376[illegible]	ZNF79	NM_007135
A_23_P20446	LZTS1	NM_021020
A_32_P14434[illegible]	PARP4	NM_006437
A_23_P33911[illegible]	ACSS3	NM_024560
A_32_P22622	NOP14	NM_003703
A_23_P46760	GDF2	NM_016204
A_23_P14436[illegible]	NAP1L5	NM_153757
A_32_P83290[illegible]	ZNF574	AK131418
A_24_P46946	SOX17	NM_022454
A_23_P47507	GPR44	NM_004778
A_23_P52410	RTKN2	NM_145307
A_23_P25019	PRIM1	NM_000946
A_33_P32574[illegible]	TAF1L	NM_153809
A_23_P82641	LANCL2	NM_018697
A_33_P34248[illegible]	FAM118A	NM_001104595
A_32_P36235	IER2	NM_004907
A_24_P23836[illegible]	ZBTB34	NM_001099270
A_23_P5882	GRHL1	NM_198182
A_23_P93737	DYNC1I1	NM_004411

A_23_P38157	ZNF25	NM_145011
A_23_P25790	CENPB	NM_001810
A_24_P13367	ZNF276	NM_152287
A_23_P21625	TPD52	NM_001025252
A_23_P25424	PTPRK	NM_002844
A_23_P50008	TTC19	NM_017775
A_32_P12396	RPGRIP1L	NM_015272
A_23_P12920	IDH2	NM_002168
A_23_P40899	MBOAT1	NM_001080480
A_33_P32260	GCET2	NM_001008756
A_23_P11252	ZNF79	NM_007135
A_23_P12907	WDR76	NM_024908
A_32_P33651	ESCO2	NM_001017420
A_23_P13458	XRCC2	NM_005431
A_33_P32824	GCNT1	NM_001097634
A_23_P13683	ZBTB39	NM_014830
A_33_P32132	CLEC4G	NM_198492
A_23_P69566	MAML3	NM_018717
A_24_P51322	FLG	NM_002016
A_23_P21436	PHF19	NM_015651
A_33_P32735	INTU	NM_015693
A_23_P4294	ZNF232	NM_014519
A_24_P41778	POM121	NM_172020
A_23_P16841	ZNF425	NM_001001661
A_24_P12963	DLG5	NM_004747
A_23_P17316	NKAIN4	NM_152864
A_23_P42854	BPTF	NM_182641
A_33_P32269	CDK5R1	NM_003885
A_23_P64888	TAS2R10	NM_023921
A_23_P12989	ALDH3A2	NM_001031806
A_23_P11234	RMI1	NM_024945
A_23_P97157	IPO9	NM_018085
A_24_P85158	SIRT3	NM_012239
A_24_P37290	MVK	NM_000431
A_24_P33153	MAGEA5	NM_021049
A_23_P43006	PDPN	NM_006474
A_23_P13750	ZBTB37	NM_032522
A_32_P29118	SEMA3D	NM_152754
A_24_P34006	ELF4	NM_001421
A_33_P33886	ABLIM1	NM_001003408
A_23_P11742	DCAF11	NM_025230
A_23_P14433	CCRN4L	NM_012118
A_24_P25452	ENTPD5	BC020966
A_23_P32481	BCL6B	NM_181844
A_33_P32252	SIN3B	NM_015260
A_32_P21362	RTCD1	NM_001130841
A_32_P19626	ADAMTS9	NM_182920
A_23_P89460	AATF	NM_012138
A_32_P13256	POU5F1	NM_002701

A_33_P32391	RABEP2	NM_024816
A_23_P14704	IKZF1	NM_006060
A_32_P36126	C10orf47	NM_153256
A_24_P64233	ALDH1B1	NM_000692
A_23_P25464	FBXW2	NM_012164
A_23_P38521	ARL8B	NM_018184
A_23_P35309	TAF5L	NM_014409
A_23_P21331	ADAMTS6	NM_197941
A_24_P79040	CAPN12	NM_144691
A_23_P43820	MFSD2A	NM_032793
A_23_P32116	ZNF594	NM_032530
A_23_P42884	TMEM44	NM_138399
A_23_P14894	SPRR2E	NM_001024209
A_23_P13854	SEC24C	NM_004922
A_23_P14228	GNA11	NM_002067
A_33_P32454	N4BP2L1	NM_052818
A_33_P32939	BICC1	NM_001080512
A_23_P30442	TIGD2	NM_145715
A_23_P12803	MARS	NM_004990
A_23_P3849	TRAP1	NM_016292
A_23_P14608	GSR	NM_000637
A_23_P25473	MLF1IP	NM_024629
A_23_P11264	ABO	NM_020469
A_23_P62692	TTC22	NM_017904
A_23_P21145	PATZ1	NM_014323
A_23_P15546	LRRC2	NM_024512
A_24_P10131	SHF	AK123658
A_23_P15378	USF2	NM_003367
A_23_P25387	CDCA7L	NM_018719
A_23_P5392	TP53I3	NM_004881
A_23_P43255	FBXO36	NM_174899
A_32_P34197	ZNF687	NM_020832
A_24_P21932	TK2	NM_004614
A_23_P34691	SPATA17	NM_138796
A_23_P11107	BMP15	NM_005448
A_23_P15492	BID	NM_197966
A_23_P13280	RPN1	NM_002950
A_23_P7313	SPP1	NM_001040058
A_23_P16351	CCDC113	NM_014157
A_23_P99437	ING1	NM_198219
A_32_P50192	RIC8A	NM_021932
A_23_P96087	H1FX	NM_006026
A_33_P33402	MLL2	NM_003482
A_23_P24485	NFRKB	NM_006165
A_23_P37445	RPLP1	NM_001003
A_23_P12965	ZNF689	NM_138447
A_33_P33362	TAF4B	NM_005640
A_23_P14110	DCUN1D3	NM_173475
A_33_P33077	DENR	NM_003677

A_23_P61823	RAB24	NM_001031677
A_23_P14962	PLEKHG5	NM_198681
A_32_P51479	UNK	NM_001080419
A_33_P32098	ZNF345	NM_003419
A_23_P8133	ZNF76	NM_003427
A_24_P20938	MLXIPL	NM_032954
A_24_P18634	ANAPC1	NM_022662
A_23_P49972	CDC6	NM_001254
A_32_P21064	EGFL7	NM_201446
A_24_P7629	RPL32P3	NR_003111
A_23_P21321	WHSC2	NM_005663
A_23_P15208	SPTBN5	NM_016642
A_33_P32449	VSX2	NM_182894
A_23_P68820	SMARCB1	NM_003073
A_33_P32482	CSNK1A1P1	NR_027320
A_24_P93598	BCAT1	NM_005504
A_32_P52816	TM7SF3	NM_016551
A_33_P33421	ATG2B	NM_018036
A_23_P20546	MYH7	NM_000257
A_23_P20471	AQP6	NM_001652
A_23_P38306	SLC5A6	NM_021095
A_24_P13035	DHRS7B	NM_015510
A_23_P12881	PCK2	NM_004563
A_23_P15746	ZNF395	NM_018660
A_33_P34030	PRR11	NM_018304
A_23_P33720	FARS2	NM_006567
A_23_P20999	IL1RN	NM_173842
A_23_P82026	POU3F2	NM_005604
A_23_P52266	IFIT1	NM_001548
A_23_P63769	USP54	NM_152586
A_23_P21150	MALAT1	NR_002819
A_33_P33626	CISD2	NM_001008388
A_33_P34236	C17orf90	NM_001039842
A_23_P36091	MLH3	NM_001040108
A_23_P11244	PJA1	NM_022368
A_23_P38766	VEGFA	NM_001025370
A_23_P25567	ABLIM2	NM_032432
A_23_P11519	FCRL4	NM_031282
A_23_P21064	TH1L	NM_198976
A_23_P88119	HSPH1	NM_006644
A_23_P20169	PRRX1	NM_006902
A_24_P28794	PSMC3IP	NM_013290
A_24_P54083	PPIE	NM_006112
A_23_P34620	RAE1	NM_001015885
A_23_P67771	BARD1	NM_000465
A_24_P41910	LOC10013216	BC006438
A_24_P94394	LRRC8B	NM_015350
A_33_P32441	HAAO	NM_012205
A_23_P13456	OR2A7	NM_001005328

A_23_P25138	REPS2	NM_004726
A_32_P78379	HNF4A	AK096973
A_23_P53838	IRS2	NM_003749
A_23_P35800	DNAJC16	NM_015291
A_23_P15497	ZNF280A	NM_080740
A_23_P14880	CDC7	NM_003503
A_23_P14189	PVR	NM_006505
A_24_P23625	DLK1	NM_003836
A_23_P14678	NEB	NM_004543
A_32_P46155	ZNF708	NM_021269
A_23_P50000	FAM57A	NM_024792
A_23_P28638	PLEKHB2	NM_017958
A_23_P13626	SCRIB	NM_182706
A_33_P33543	KRTAP12-4	NM_198698
A_23_P25276	SMARCA2	NM_139045
A_23_P2671	KRT4	NM_002272
A_32_P17044	SUB1	NM_006713
A_23_P12843	METTL7A	NM_014033
A_33_P33865	SGPP2	NM_152386
A_23_P5415	NIF3L1	NM_021824
A_23_P21315	HNRPDL	NM_031372
A_24_P39710	CDC25A	NM_001789
A_24_P41085	PMS2	NM_000535
A_23_P30265	CEP72	NM_018140
A_23_P16579	HOXD13	NM_000523
A_32_P35193	PMS2L5	NR_027776
A_33_P32936	DENND4C	NM_017925
A_23_P60180	ABL1	NM_005157
A_23_P16197	CCDC106	NM_013301
A_24_P84834	DGKA	NM_201444
A_23_P12095	XRCC6	NM_001469
A_23_P20916	FSTL3	NM_005860
A_23_P16247	CDCA3	NM_031299
A_23_P75325	SGPL1	NM_003901
A_23_P92543	SCFD2	NM_152540
A_23_P22332	INPP5D	NM_001017915
A_23_P14772	TRAIP	NM_005879
A_24_P63633	CCDC84	NM_198489
A_32_P14929	KIAA1841	NM_001129993
A_24_P40403	C15orf38	NM_182616
A_24_P19474	ZNF275	NM_001080485
A_23_P12639	SETDB1	NM_012432
A_23_P33349	EEPD1	NM_030636
A_23_P50357	ARHGEF18	NM_015318
A_23_P34375	TCEA3	NM_003196
A_33_P33426	DHRS12	NM_001031719
A_23_P25875	NCAPH	NM_015341
A_23_P15663	ZSCAN12	NR_028077
A_33_P33924	BCL10	NM_003921

A_23_P36325	CCDC68	NM_025214
A_23_P10966	GABRB3	NM_000814
A_33_P32448	EIF2C3	NM_024852
A_23_P41491	GLIPR2	NM_022343
A_23_P58132	RHOH	NM_004310
A_32_P72341	TRIM59	NM_173084
A_32_P14089	FOXN2	NM_002158
A_23_P25642	ADAMDEC1	NM_014479
A_23_P35796	PPP2R5B	NM_006244
A_23_P38890	SLC22A15	NM_018420
A_32_P64096	ZKSCAN2	NM_001012981
A_23_P14507	PNRC1	NM_006813
A_23_P82503	PEG10	NM_001040152
A_23_P15318	SERPINB2	NM_001143818
A_32_P61853	KIAA1468	NM_020854
A_32_P10924	CSRNP3	NM_001172173
A_33_P32268	TNFSF10	NM_003810
A_23_P93772	HOXA5	NM_019102
A_24_P32781	STIP1	NM_006819
A_23_P63083	ASH1L	NM_018489
A_33_P32625	LIPK	NM_001080518
A_23_P16409	CAPN12	NM_144691
A_23_P3527	POLR2C	NM_032940
A_24_P35043	THAP2	NM_031435
A_23_P32593	COPA	NM_004371
A_24_P19410	CBX7	NM_175709
A_33_P33425	PITPNM3	NM_031220
A_23_P30136	ZNF572	NM_152412
A_23_P33707	S100A2	NM_005978
A_23_P44505	KLF11	NM_003597
A_32_P82462	LOC554202	NR_027054
A_23_P82738	RAD54B	NM_012415
A_24_P67538	C11orf58	NM_014267
A_23_P14391	ATP2C1	NM_014382
A_24_P69538	TLR4	NM_138554
A_23_P19691	HEBP2	NM_014320
A_23_P10930	SLC5A3	NM_006933
A_32_P35264	ZNF793	NM_001013659
A_24_P18794	BID	NM_197966
A_24_P15858	CRY2	NM_021117
A_23_P15896	SLC39A11	NM_139177
A_23_P10299	PI4KA	NM_002650
A_24_P12831	ZNF79	NM_007135
A_23_P72643	ADAM9	NM_003816
A_23_P14097	ZNF263	NM_005741
A_23_P90542	ZNF540	NM_152606
A_33_P32725	CTPS2	NM_001144002
A_32_P8221	GRM8	NM_000845
A_23_P10330	S100A7	NM_002963

A_23_P40154ɛ	PVRL3	NM_015480
A_24_P29319ɛ	FXYD3	NM_005971
A_24_P33273ɛ	GUSBP1	NR_027028
A_24_P28241ɛ	ABL1	NM_007313
A_24_P66499ɛ	CBX5	NM_001127322
A_23_P13567ɛ	KCNMB3	NM_171828
A_23_P25162ɛ	SUPT3H	NM_003599
A_32_P45351	ZNF471	AB037817
A_33_P34464ɛ	FRMD4B	NM_015123
A_23_P13767ɛ	GOLT1A	NM_198447
A_23_P17126ɛ	H2AFB1	NM_001017990
A_33_P33112ɛ	BSX	NM_001098169
A_23_P21802ɛ	KIF5A	NM_004984
A_23_P25966ɛ	ZKSCAN5	NM_014569
A_32_P23244ɛ	ESRRA	NM_004451
A_23_P21469ɛ	MAPK14	NM_001315
A_23_P21032ɛ	CEP68	NM_015147
A_23_P3261	PDE8A	NM_002605
A_24_P87579	CYB561	NM_001017916
A_33_P32523ɛ	BDH1	NM_203314
A_24_P29197ɛ	ADCK2	NM_052853
A_33_P33965ɛ	POLR3G	NM_006467
A_24_P91549ɛ	INPP4B	BC005273
A_23_P27867	PLAUR	NM_002659
A_23_P14191ɛ	TYK2	NM_003331
A_23_P12683ɛ	TNFSF4	NM_003326
A_23_P20536ɛ	PPP4R4	NM_020958
A_33_P33867ɛ	PPP1R15B	NM_032833
A_23_P8794	LOC10013283	NR_028058
A_32_P20840ɛ	GNG2	NM_053064
A_24_P15158ɛ	TEF	NM_003216
A_24_P33535ɛ	PUS1	NM_025215
A_32_P37564ɛ	TRHDE	NM_013381
A_23_P12286ɛ	GRB10	NM_001001555
A_23_P25169ɛ	NXT1	NM_013248
A_33_P32542ɛ	MAPK10	NM_138980
A_24_P13086ɛ	PHF8	NM_015107
A_23_P14014ɛ	BDKRB2	NM_000623
A_24_P36608ɛ	MICAL3	NM_015241
A_23_P21843ɛ	ZNF226	NM_001032374
A_23_P20480	BRF2	NM_018310
A_23_P47938	HOXC11	NM_014212
A_23_P75642	HYOU1	NM_006389
A_23_P30784	ABT1	NM_013375
A_23_P10618ɛ	C14orf43	NM_194278
A_23_P34274ɛ	LIX1L	NM_153713
A_23_P31333	ZNF12	NM_016265
A_24_P22814ɛ	KRT13	NM_002274
A_24_P33079ɛ	MKS1	NM_017777

A_24_P22611	NAA15	NM_057175
A_23_P11815	HS3ST2	NM_006043
A_32_P33252	PIR	NM_003662
A_24_P10368	AGPAT6	NM_178819
A_23_P36928	POLR1D	NM_015972
A_23_P15542	DUSP7	NM_001947
A_23_P11003	ZNF589	NM_016089
A_32_P21367	SMARCA5	ENST00000283131
A_23_P20269	KBTBD4	NM_016506
A_33_P33808	PAX2	NM_003990
A_23_P34879	RBM15	NM_022768
A_23_P85941	ALX3	NM_006492
A_23_P35624	LIPK	NM_001080518
A_33_P32117	CREB3L2	NM_194071
A_23_P83192	PHPT1	NM_014172
A_23_P11957	KCNN1	NM_002248
A_23_P65967	SLC38A7	NM_018231
A_23_P16094	EPS8L3	NM_024526
A_33_P32866	IRAK1	NM_001569
A_24_P67396	TTC22	ENST00000371276
A_23_P13083	BARX2	NM_003658
A_23_P65217	TGDS	NM_014305
A_23_P12082	CHI3L2	NM_001025199
A_23_P95823	NSMCE1	NM_145080
A_32_P17466	TAF7L	NM_024885
A_23_P35480	KLF12	NM_007249
A_33_P33745	SPATA7	NM_018418
A_32_P47687	SIM1	ENST00000369208
A_33_P32722	MFSD2A	NM_001136493
A_24_P25196	FGF1	NM_000800
A_32_P53464	SPRR4	NM_173080
A_32_P10083	KIF19	NM_153209
A_33_P33152	KRT79	NM_175834
A_33_P33291	DNMT1	NM_001130823
A_23_P16284	LAMP1	NM_005561
A_23_P21481	SIRT5	NM_012241
A_23_P11877	KPNB1	NM_002265
A_23_P34122	KLHL21	NM_014851
A_23_P90722	PTPRN	NM_002846
A_33_P32292	HIST2H2BF	NM_001024599
A_32_P74602	MAPK1	ENST00000215832
A_32_P40547	MEG3	NR_002766
A_23_P59179	RXRB	NM_021976
A_23_P15187	GLCE	NM_015554
A_23_P40809	KLF15	NM_014079
A_24_P36451	RCBTB1	NM_018191
A_23_P80684	PHF7	NM_173341
A_23_P37287	S100A13	NM_001024210
A_24_P64167	PTGS1	NM_000962

A_23_P15533	PLD1	NM_002662
A_33_P33383	CASZ1	NM_001079843
A_23_P34234	COX4I1	BC047869
A_23_P14118	ZNF750	NM_024702
A_24_P24244	ATN1	NM_001007026
A_23_P14111	ABAT	NM_000663
A_24_P36291	GPD1L	NM_015141
A_23_P41115	WNT1	NM_005430
A_23_P15386	LASS4	NM_024552
A_24_P94402	MYCN	NM_005378
A_23_P23044	S100A9	NM_002965
A_23_P56952	SERTAD2	NM_014755
A_23_P20318	APOC3	NM_000040
A_24_P24535	ATP5A1	NM_001001937
A_23_P42902	HKR1	NM_181786
A_32_P22456	LIN28B	NM_001004317
A_33_P36191	PMAIP1	NM_021127
A_24_P30554	TRIB3	NM_021158
A_23_P11459	SPRR2G	NM_001014291
A_32_P17797	SKA3	NM_145061
A_33_P33681	MAP3K1	NM_005921
A_23_P19712	GMNN	NM_015895
A_33_P33376	TRPC6	NM_004621
A_23_P12447	STIP1	NM_006819
A_23_P12105	PCCB	NM_000532
A_24_P1910	SUPT4H1	NM_003168
A_23_P91930	QTRTD1	NM_024638
A_24_P26239	AATF	NM_012138
A_33_P33214	ZSCAN5C	ENST00000376267
A_23_P25337	CUX1	NM_001913
A_23_P64689	PAN2	NM_014871
A_23_P82027	POU3F2	NM_005604
A_24_P53223	CREB5	NM_182898
A_23_P99260	METAP2	NM_006838
A_24_P60441	EVC	NM_153717
A_23_P67476	KIAA0355	NM_014686
A_32_P19955	C3orf57	NM_001040100
A_23_P38503	E2F3	NM_001949
A_23_P35841	SRGAP3	NM_014850
A_23_P15772	DENND4C	NM_017925
A_23_P20351	CARS	NM_001014438
A_23_P42666	MITF	NM_198159
A_23_P85780	PHGDH	NM_006623
A_23_P61057	IL16	NM_004513
A_23_P16795	PPP2R5D	NM_180976
A_23_P20511	POSTN	NM_006475
A_23_P13896	SDHD	NM_003002
A_24_P17723	CABP7	NM_182527
A_23_P22297	LOR	NM_000427

A_23_P25268	PCYT1A	NM_005017
A_33_P33822	SEC16A	NM_014866
A_23_P35901	PHF21A	NM_016621
A_23_P40827	CCDC79	NM_001136505
A_32_P37772	GIPC3	NM_133261
A_33_P32123	LZTS1	NM_021020
A_23_P51951	IL24	NM_006850
A_24_P34453	ZNF625	NM_145233
A_23_P21868	UBE2V1	NM_001032288
A_23_P21563	IGFBP3	NM_001013398
A_23_P21199	SEC22C	NM_032970
A_33_P32876	CNOT3	NM_014516
A_23_P17811	SEC14L2	NM_012429
A_23_P15415	MFSD9	NM_032718
A_24_P30476	SIN3A	NM_001145358
A_32_P47674	FST	ENST00000256759
A_23_P66487	SMARCD2	NM_003077
A_23_P89343	SNX11	NM_152244
A_23_P1885	DTX4	NM_015177
A_23_P31143	TPD52L1	NM_001003395
A_23_P49327	ZNF174	NM_003450
A_24_P36429	STX2	NM_001980
A_23_P15460	SULF2	NM_018837
A_23_P12496	FAM89B	NM_152832
A_23_P11934	TEAD2	NM_003598
A_32_P18673	ISM1	NM_080826
A_32_P14442	ZNF518B	NM_053042
A_23_P8095	RNF5	NM_006913
A_23_P88580	ARID3B	NM_006465
A_23_P29723	SGOL1	NM_001012410
A_23_P85598	CABC1	NM_020247
A_33_P32104	C1QTNF4	NM_031909
A_32_P16714	PIGW	NM_178517
A_23_P42400	POU2F1	NM_002697
A_23_P30254	PLK2	NM_006622
A_23_P2873	KLC1	NM_182923
A_33_P33468	MYBL1	NM_001144755
A_23_P10311	MAFF	NM_012323
A_32_P98502	COX5A	NM_004255
A_33_P32293	ID4	NM_001546
A_23_P48295	CDADC1	NM_030911
A_23_P12791	PAMR1	NM_015430
A_23_P80583	CLDN16	NM_006580
A_23_P19723	BMP5	NM_021073
A_23_P10891	CENPA	NM_001809
A_23_P15688	ENPP1	NM_006208
A_33_P34081	ZNF717	NM_001128223
A_23_P38864	RABAC1	NM_006423
A_23_P62948	PBX1	NM_002585

A_32_P89191(	C6orf150	NM_138441
A_24_P41592{	ESRRB	NM_004452
A_23_P11906{	ZNF71	NM_021216
A_23_P14858‹	DOCK11	NM_144658
A_23_P14670{	LMX1B	NM_002316
A_32_P20782!	EFNA5	NM_001962
A_24_P32359{	ESCO2	NM_001017420
A_23_P7843	AKIRIN2	NM_018064
A_23_P39500:	SLC2A12	NM_145176
A_24_P20604:	SLC25A4	NM_001151
A_24_P66545	KIAA0226	NM_001145642
A_23_P16768:	GDNF	NM_000514
A_23_P46118	CHML	NM_001821
A_33_P33471‹	SUPT3H	NM_181356
A_23_P41564:	ZNF48	NM_152652
A_32_P44671(	FAM123A	ENST00000357816
A_33_P32144:	ZC3HAV1L	NM_080660
A_33_P32559:	MYLIP	NM_013262
A_32_P47887:	MEGF9	NM_001080497
A_33_P32411(	DNAJC11	NM_018198
A_24_P32326!	ANKHD1	NM_017747
A_32_P48601!	RNF39	NM_025236
A_23_P41062!	ZNF367	NM_153695
A_33_P32794:	AGRP	NM_001138
A_23_P41942	POLR3G	NM_006467
A_33_P33407:	KDM6A	NM_021140
A_23_P2814	SMAD9	NM_005905
A_23_P16147‹	MCM10	NM_182751
A_23_P38451:	GYG1	NM_004130
A_33_P33269{	RAGE	NM_014226
A_23_P14019:	KIAA0125	NR_026800
A_24_P11884‹	TTC28	NM_001145418
A_24_P93764‹	CDK11A	AB209095
A_32_P18501(	BLM	NM_000057
A_32_P85978	ZNF414	NM_001146175
A_23_P29248	TST	NM_003312
A_23_P16112!	MOV10	NM_020963
A_23_P40638!	FBXL16	NM_153350
A_23_P39480!	DTNA	NM_001390
A_23_P46755	GDF2	NM_016204
A_23_P8807	CYP3A5	NM_000777
A_23_P78479	ZIM2	NM_015363
A_24_P62505	GLT25D2	NM_015101
A_32_P12533{	FAM43B	NM_207334
A_23_P21567!	C7orf44	NM_018224
A_32_P18276(	BCOR	NM_017745
A_23_P31293:	KRTAP8-1	NM_175857
A_23_P21857‹	GLB1L	NM_024506
A_24_P23325(	ZNF649	NM_023074

A_24_P21155[illegible]	PATZ1	NM_032051
A_33_P32629[illegible]	PCBP4	NM_033010
A_23_P15159[illegible]	CPNE6	NM_006032
A_32_P96775	C3orf78	NM_001124767
A_23_P21857[illegible]	GLB1L	NM_024506
A_24_P20630	LEF1	NM_016269
A_32_P69150	STEAP1	NM_012449
A_24_P30114[illegible]	LMO7	NM_005358
A_23_P40601[illegible]	IFT80	NM_020800
A_23_P16078[illegible]	PEX14	NM_004565
A_23_P16394[illegible]	SUPT4H1	NM_003168
A_33_P34241[illegible]	ARID3C	NM_001017363
A_32_P10272	PMS2L2	NR_003614
A_24_P18194[illegible]	PHF20	NM_016436
A_23_P36037[illegible]	EGLN3	NM_022073
A_23_P43009[illegible]	ZSCAN4	NM_152677
A_23_P76320	PIK3C2G	NM_004570
A_24_P13158[illegible]	CD86	NM_006889
A_23_P31338[illegible]	UGCG	NM_003358
A_24_P32701[illegible]	PPCDC	NM_021823
A_23_P26777	NUP85	NM_024844
A_23_P13878[illegible]	ACP2	NM_001610
A_33_P32683[illegible]	FAM167A	NM_053279
A_23_P16883[illegible]	PTK2B	NM_173174
A_23_P21458[illegible]	TRIM26	NM_003449
A_24_P20193[illegible]	GTF2A1	AK021641
A_23_P97423	UBE2Q1	NM_017582
A_23_P12526[illegible]	KPNA2	NM_002266
A_32_P50400[illegible]	LCOR	NM_032440
A_23_P30980[illegible]	ZNF777	NM_015694
A_24_P26812[illegible]	ST3GAL3	NM_174963
A_33_P34222[illegible]	AQP6	NM_001652
A_32_P69149	STEAP1	NM_012449
A_23_P63178	TAF12	NM_005644
A_33_P34137[illegible]	ACER2	NM_001010887
A_32_P33842[illegible]	IGFL3	NM_207393
A_24_P23960[illegible]	GADD45B	NM_015675
A_23_P83714	ZNF707	NM_173831
A_23_P97250	GON4L	NM_001037533
A_23_P64873	DCN	NM_001920
A_33_P33570[illegible]	DDX55	NM_020936
A_33_P32382[illegible]	COBLL1	NM_014900
A_24_P91232	FAM104A	NM_032837
A_23_P37659[illegible]	CLYBL	NM_206808
A_23_P30776	HIST1H2BE	NM_003523
A_23_P73540	CCDC120	NM_033626
A_23_P13882[illegible]	SYVN1	NM_172230
A_33_P32302[illegible]	GRHL1	NM_198182
A_23_P10088[illegible]	SUZ12	NM_015355

A_24_P11418	FDPS	NM_002004
A_32_P19552	ZNF594	NM_032530
A_23_P99540	ZFP36L1	NM_004926
A_23_P87902	DYRK4	NM_003845
A_23_P97871	ARID5B	NM_032199
A_33_P32555	FCHO1	NM_001161357
A_33_P32363	PVRL4	NM_030916
A_23_P34144	MNT	NM_020310
A_23_P74097	TCEB3	NM_003198
A_33_P33101	ADRB1	NM_000684
A_24_P94406	ZNF785	NM_152458
A_23_P97130	POU2F1	NM_002697
A_23_P37214	C19orf29	NM_021231
A_32_P39898	TRIM69	NM_182985
A_23_P36458	DOCK4	NM_014705
A_23_P10073	SKAP1	NM_003726
A_24_P93223	TET2	NM_017628
A_24_P29440	PLA1A	NM_015900
A_24_P59602	LOC440104	XM_001721986
A_23_P94711	IGFBPL1	AK131521
A_23_P91636	POM121L9P	NR_003714
A_24_P67378	PIP4K2A	NM_005028
A_33_P32691	STX17	NM_017919
A_32_P12997	ZNF284	ENST00000328297
A_33_P32352	CTPS	NM_001905
A_32_P71943	DSTYK	NM_015375
A_23_P16726	PHF17	NM_199320
A_24_P24510	USP7	NM_003470
A_23_P35871	E2F8	NM_024680
A_32_P23109	RNF182	NM_152737
A_23_P42037	DNMT3A	NM_175630
A_23_P33118	RASGEF1B	NM_152545
A_23_P29124	GP1BB	NM_000407
A_23_P13989	MDM2	NM_002392
A_32_P14709	ARHGAP11A	NM_199357
A_24_P20877	HYAL1	NM_153282
A_23_P68547	MCM8	NM_182802
A_24_P19364	GPT2	NM_133443
A_33_P33224	LOC654780	XR_111602
A_32_P15006	EXT2	NM_000401
A_23_P16198	MRGPRX4	NM_054032
A_23_P42414	NEDD9	NM_006403
A_23_P27285	MPPE1	NM_023075
A_32_P10017	VCY	NM_004679
A_23_P72584	ACBD7	NM_001039844
A_33_P32795	MIXL1	NM_031944
A_23_P32617	CALM2	NM_001743
A_33_P33736	EFR3B	NM_014971
A_24_P25381	FLOT2	NM_004475

A_32_P53801	TBX10	NM_005995
A_23_P50054	DMBX1	NM_147192
A_33_P32241	C15orf23	NM_001142761
A_23_P11752	OPRD1	NM_000911
A_23_P351	EPB41	NM_203342
A_23_P14829	RS1	NM_000330
A_24_P34961	UBTF	NM_001076683
A_23_P41540	KLF9	NM_001206
A_24_P19471	FAM100B	NM_182565
A_23_P16862	RBM28	NM_018077
A_23_P10150	KLK11	NM_144947
A_23_P74943	KCNH1	NM_172362
A_23_P10907	SALL4	NM_020436
A_32_P23493	TARDBP	NM_007375
A_23_P40987	MVK	M88468
A_24_P37996	SOX2	NM_003106
A_23_P43072	ATP4A	NM_000704
A_33_P33503	C10orf58	NM_032333
A_23_P14024	CHD8	NM_020920
A_23_P20027	BCL9	NM_004326
A_23_P74526	PPIE	NM_203456
A_33_P32296	FLJ39739	XR_079533
A_32_P11848	SH2D4A	ENST00000265807
A_23_P90264	EIF3K	NM_013234
A_23_P36797	CDKN1C	NM_000076
A_24_P57528	SLC39A11	NM_139177
A_23_P92213	ABHD10	NM_018394
A_33_P32582	MCM7	NM_005916
A_23_P24433	CTSF	NM_003793
A_23_P12603	RLF	NM_012421
A_24_P16604	IMPDH2	NM_000884
A_23_P15631	SKP2	NM_032637
A_32_P22093	RPE	NM_006916
A_23_P10562	COQ5	NM_032314
A_33_P34073	DBX1	NM_001029865
A_33_P33851	TOLLIP	NM_019009
A_23_P13842	USP6NL	NM_014688
A_23_P16012	ZNF132	NM_003433
A_24_P52111	RAB24	NM_001031677
A_23_P56314	UQCR	NM_006830
A_32_P13383	ACSS3	NM_024560
A_33_P34037	ZNF579	NM_152600
A_24_P90216	LGR4	NM_018490
A_33_P32489	MPP5	NM_022474
A_23_P20605	PRC1	NM_003981
A_23_P79247	PGAP1	NM_024989
A_24_P39123	CYYR1	NM_052954
A_32_P44721	ZNF462	ENST00000277225
A_23_P35906	CASP4	NM_033306

A_23_P35735	NLRP10	ENST00000328600
A_24_P12213	LIF	NM_002309
A_23_P25669	MCM3APAS	NR_002776
A_23_P4425	FLII	NM_002018
A_24_P23280	GSC	NM_173849
A_23_P28263	CTDSP1	NM_021198
A_24_P11313	BZRAP1	NM_004758
A_33_P32575	CCDC103	NM_213607
A_32_P40585	BRCA1	NM_007300
A_23_P11839	RASD1	NM_016084
A_23_P33109	KRT78	AK096419
A_33_P32573	ATG2A	NM_015104
A_23_P54100	ESR2	NM_001437
A_23_P35398	ANXA11	NM_145869
A_33_P33507	KRT7	NM_005556
A_23_P10385	DTL	NM_016448
A_23_P11685	KRT2	NM_000423
A_24_P91500	NACC1	NM_052876
A_24_P27014	CD63	NM_001040034
A_23_P40664	CSNK1E	NM_152221
A_32_P51269	IFT80	NM_020800
A_23_P42938	HOXD9	NM_014213
A_23_P20441	ITFG2	NM_018463
A_23_P39127	RCAN3	NM_013441
A_23_P15480	TRPC4AP	NM_015638
A_23_P39439	JPH2	NM_020433
A_23_P37099	MCM4	NM_005914
A_23_P15202	CSK	NM_004383
A_23_P49459	LOC81691	NM_030941
A_23_P21862	NEU4	NM_080741
A_23_P11357	PDX1	AF049893
A_23_P37044	GRTP1	NM_024719
A_23_P13284	CLCN2	NM_004366
A_33_P33569	ARFGEF1	AB209324
A_23_P46852	OBFC1	NM_024928
A_24_P37409	DUSP2	NM_004418
A_23_P10530	DGKA	NM_201444
A_23_P85703	SOX13	NM_005686
A_23_P38018	LMO4	NM_006769
A_23_P9883	NLRP3	NM_001079821
A_24_P87036	ANO1	NM_018043
A_23_P20170	S100A2	NM_005978
A_23_P36331	HOXB5	NM_002147
A_24_P25615	NKX1-2	NM_001146340
A_23_P21245	SEC61A1	NM_013336
A_23_P12796	PRCP	NM_199418
A_23_P85083	RHOXF1	NM_139282
A_23_P15640	C6orf155	NR_026807
A_23_P54029	SLC22A17	ENST00000354772

A_24_P49383	C11orf67	NM_024684
A_24_P21914	YY1	NM_003403
A_24_P41208	MCM10	NM_182751
A_32_P22238	HMGN2	NM_005517
A_24_P42681	PSMD2	NM_002808
A_23_P21100	NRIP1	NM_003489
A_23_P15690	SOBP	NM_018013
A_23_P20647	TRAF7	NM_032271
A_23_P12083	TFIP11	NM_001008697
A_32_P24612	MOG	NM_206814
A_32_P47449	KIF18B	NM_001080443
A_32_P26808	SETDB1	NM_012432
A_23_P64567	PPME1	NM_016147
A_33_P33973	ZNF461	NM_153257
A_24_P11786	ADD1	NM_001119
A_23_P37092	C3orf78	NM_001124767
A_33_P34090	TYROBP	NM_003332
A_33_P32095	SLFN5	NM_144975
A_33_P33891	STK11	NM_000455
A_23_P41428	C16orf71	NM_139170
A_33_P32306	KIF1A	NM_004321
A_23_P39594	NCOA1	NM_147223
A_23_P37098	MCM4	NM_005914
A_23_P17115	KLHL4	NM_057162
A_23_P43065	HEYL	NM_014571
A_23_P84860	FAM107A	NM_007177
A_23_P20289	ACRV1	NM_001612
A_23_P33495	FAM167A	NM_053279
A_23_P14405	PRKCD	NM_006254
A_33_P32879	RASA4	NM_006989
A_23_P20572	C14orf102	NM_199043
A_23_P12105	STAC	NM_003149
A_23_P9591	LMAN2	NM_006816
A_32_P80591	ELOVL6	ENST00000394607
A_23_P39056	KLK7	NM_005046
A_23_P10449	PAPSS2	NM_001015880
A_23_P20958	MYCN	NM_005378
A_23_P32375	FAM83D	NM_030919
A_23_P32983	UTY	NM_007125
A_23_P32026	DMKN	NM_001035516
A_32_P21384	RPL17	NM_000985
A_23_P30954	MDM2	NM_006879
A_23_P43081	HSPC159	NM_014181
A_23_P51085	SPC25	NM_020675
A_32_P39394	GLIS3	NM_001042413
A_23_P62840	YRDC	NM_024640
A_23_P20462	ELK3	NM_005230
A_23_P20273	JRKL	NM_003772
A_23_P12253	C6orf48	NM_001040437

A_24_P20155	FEZ1	NM_005103
A_32_P38324	WDR66	NM_144668
A_33_P33430	SMARCB1	NM_003073
A_23_P28238	SNX17	NM_014748
A_23_P41851	PRPS1L1	NM_175886
A_24_P35263	TSSK4	NM_174944
A_32_P54274	DRD5	NM_000798
A_23_P60210	RLN1	NM_006911
A_32_P43748	DNAH17	NM_173628
A_23_P20548	SLC7A8	NM_182728
A_23_P31141	FLCN	AL831885
A_33_P32348	CBR4	NM_032783
A_33_P33251	SOX13	NM_005686
A_23_P57089	PMEPA1	NM_020182
A_24_P21042	STARD13	NM_178006
A_24_P23348	LIF	NM_002309
A_32_P19282	PRPS1L1	NM_175886
A_33_P32284	FXYD1	NM_005031
A_23_P71889	ODF2	NM_153437
A_23_P36655	DENND5B	NM_144973
A_23_P80449	THRB	NM_000461
A_24_P38815	TPP1	NM_000391
A_23_P31134	DENND2D	AL713773
A_33_P36360	MLH3	NM_001040108
A_23_P21720	SLC35A2	NM_005660
A_23_P43167	SULF1	NM_015170
A_24_P4705	PPME1	NM_016147
A_23_P30451	ZNF397	NM_032347
A_32_P42077	C7orf28B	NM_198097
A_23_P80891	USP4	NM_003363
A_23_P12097	FAM118A	NM_017911
A_23_P15394	GTDC1	NM_001006636
A_23_P12272	PPP1R8	NM_138558
A_23_P37915	BRWD3	NM_153252
A_23_P69834	HTR1A	NM_000524
A_23_P14552	PKIB	NM_181795
A_23_P62901	BTG2	NM_006763
A_33_P32413	PLA2G4F	NM_213600
A_24_P43810	FAM83A	NM_207006
A_32_P84790	FUS	NM_004960
A_33_P33595	DNMT3B	AB208880
A_24_P11601	PSMD9	NM_002813
A_23_P52552	BAG3	NM_004281
A_24_P16594	CLDN1	NM_021101
A_33_P32492	CNOT6L	NM_144571
A_23_P14923	HAPLN2	NM_021817
A_23_P14445	CAMK2D	NM_001221
A_33_P33848	FBXO5	NM_001142522
A_23_P4190	ACSF2	NM_025149

A_24_P23727	NIPSNAP1	NM_003634
A_24_P21858	MED17	NM_004268
A_23_P14014	IFI27L2	NM_032036
A_24_P91288	SELS	NM_203472
A_24_P28704	IFITM2	NM_006435
A_32_P16225	ARHGAP18	NM_033515
A_24_P21642	DTNB	NM_033148
A_23_P31408	RNF126	NM_194460
A_23_P77818	ATP5H	NM_006356
A_33_P32721	CLIP2	NM_003388
A_24_P12403	RIPK2	NM_003821
A_23_P15141	KATNAL1	NM_032116
A_23_P25653	INTS6	NM_012141
A_23_P27273	NEDD4L	NM_015277
A_24_P38732	ZNF44	NM_016264
A_33_P32984	LMO4	NM_006769
A_33_P33569	TCEAL6	NM_001006938
A_23_P91619	MIF	NM_002415
A_24_P32394	ZRANB3	NM_032143
A_32_P15389	TAF3	NM_031923
A_23_P15411	IGFBP5	NM_000599
A_23_P99699	ZBTB1	NM_014950
A_32_P46624	C12orf60	NM_175874
A_32_P69368	ID2	NM_002166
A_23_P14977	ARHGAP12	NM_018287
A_23_P81121	EXOSC9	NM_005033
A_33_P33920	IDUA	NM_000203
A_24_P87376	BCR	NM_004327
A_32_P40487	C10orf55	NM_001001791
A_33_P33202	ADCY2	NM_020546
A_23_P21846	SERTAD1	NM_013376
A_33_P33808	STAT5B	NM_012448
A_23_P25384	HSF1	NM_005526
A_24_P80338	NEK4	NM_003157
A_23_P53541	CHD4	NM_001273
A_23_P21158	C22orf32	NM_033318
A_24_P34496	AMOT	NM_133265
A_32_P16056	OPCML	NM_001012393
A_23_P15844	BRIP1	NM_032043
A_24_P95439	CARS	NM_001014438
A_32_P23360	YPEL2	NM_001005404
A_23_P88600	MEIS2	NM_170677
A_32_P20882	PLXDC1	NM_020405
A_23_P44919	ACSM5	ENST00000331849
A_32_P19254	TCEAL6	NM_001006938
A_23_P52589	RASSF7	NM_003475
A_24_P14298	PI4KA	NM_002650
A_23_P20440	KDM5A	NM_001042603
A_33_P33568	LCE1C	NM_178351

A_23_P12739	CRY2	NM_021117
A_24_P25092	PTGS2	NM_000963
A_23_P29754	BDH1	NM_203314
A_32_P11393	LNX2	NM_153371
A_32_P44731	MLLT3	NM_004529
A_23_P30383	SCN4B	NM_174934
A_23_P32547	HIST1H4G	NM_003547
A_32_P84497	TACC2	NM_206862
A_23_P70045	H2AFY	NM_138610
A_23_P32707	ESPL1	NM_012291
A_23_P70934	DLX6	NM_005222
A_24_P41067	JAK1	NM_002227
A_24_P36354	HIP1	NM_005338
A_23_P32330	DEDD2	NM_133328
A_23_P40670	CSNK1E	NM_152221
A_23_P69468	NDUFB4	NM_004547
A_23_P39004	MAFG	NM_002359
A_32_P14704	ZNF311	NM_001010877
A_23_P25272	DLC1	NM_182643
A_33_P32303	ZNF784	NM_203374
A_24_P31713	EXOSC7	NM_015004
A_33_P35935	MARK3	NM_001128918
A_33_P34204	LGALS9	NM_002308
A_23_P16961	AFF1	NM_005935
A_23_P14661	OR1B1	NM_001004450
A_24_P61520	MLH3	NM_001040108
A_23_P71948	AKNA	NM_030767
A_23_P89919	ZNF180	NM_013256
A_23_P82642	LANCL2	NM_018697
A_23_P11370	PDGFA	NM_002607
A_23_P23765	ITGB3BP	NM_014288
A_23_P15370	MIDN	NM_177401
A_32_P41244	ZNF876P	NR_027481
A_33_P36911	IL31RA	NM_139017
A_23_P13309	RAPGEF2	NM_014247
A_23_P20044	SHC1	NM_003029
A_23_P78289	FAM104A	NM_032837
A_23_P10178	IL12RB1	NM_005535
A_23_P25253	DERA	NM_015954
A_23_P10998	CD86	NM_006889
A_23_P13150	FOXD4L1	NM_012184
A_23_P16119	VIM	NM_003380
A_23_P88630	BLM	NM_000057
A_33_P32303	TTF1	NM_007344
A_33_P32237	MED14	NM_004229
A_23_P21364	MGAT4B	NM_054013
A_24_P37424	GATA1	NM_002049
A_33_P33576	KRTAP10-12	NM_198699
A_23_P81690	COX7A2	NM_001865

A_24_P17053	UTP14A	NM_006649
A_23_P81399	SQSTM1	NM_003900
A_24_P21897	CDCA3	NM_031299
A_24_P22218	TMEM48	ENST00000371429
A_23_P32500	STAB1	NM_015136
A_23_P13204	GATA5	NM_080473
A_23_P92129	IFRD2	NM_006764
A_23_P15628	DBN1	NM_080881
A_24_P88554	PEX11B	NM_003846
A_24_P16146	C20orf199	NR_003605
A_23_P20134	DVL1	NM_004421
A_23_P61960	ATP6V0E2	NM_145230
A_33_P33160	INTS1	NM_001080453
A_32_P21911	CENPJ	NM_018451
A_23_P79429	HJURP	NM_018410
A_23_P67618	ZNF792	NM_175872
A_23_P28733	RBL1	NM_002895
A_23_P12209	ATP10B	NM_025153
A_23_P25276	SMARCA2	NM_139045
A_23_P16404	MMD	NM_012329
A_32_P33434	ZNF812	NM_001199814
A_24_P14877	RASSF1	NM_170713
A_23_P15402	SAT2	NM_133491
A_23_P77918	HS3ST3B1	NM_006041
A_24_P93828	FCGR2B	NM_004001
A_23_P14798	NEO1	NM_002499
A_23_P20350	TRIM5	NM_033034
A_23_P25736	GFI1	NM_005263
A_23_P25015	IGF2BP2	NM_006548
A_32_P52169	GMEB1	ENST00000373816
A_23_P11925	ASF1B	NM_018154
A_23_P97021	EIF2C3	NM_024852
A_23_P76159	EEA1	NM_003566
A_23_P15208	SPTBN5	NM_016642
A_23_P14227	PAF1	NM_019088
A_24_P15913	NEDD8	NM_006156
A_23_P20323	ABCG4	NM_022169
A_23_P20683	KIAA0020	NM_014878
A_23_P21082	PCMTD2	NM_018257
A_23_P42995	PRX	NM_020956
A_23_P14999	PDLIM1	NM_020992
A_23_P33181	ZNF687	NM_020832
A_32_P31240	SEMA3D	NM_152754
A_23_P21164	PPARA	NM_005036
A_32_P1533	LOC202181	NR_026921
A_23_P25638	PSIP1	NM_021144
A_24_P22411	PLA2G1B	NM_000928
A_23_P25328	TBC1D8	NM_001102426
A_23_P25541	SOX18	NM_018419

A_23_P12221(	FLJ11292	AK023417
A_32_P72219:	GABPB2	ENST00000368918
A_23_P39741:	TMEM188	NM_153261
A_32_P20582	VPS13D	NM_015378
A_24_P15649(	KCNMA1	NM_002247
A_23_P13374:	IGF2BP3	NM_006547
A_23_P42282	C4B	NM_001002029
A_24_P25205:	RBM24	NM_153020
A_23_P11206:	HGSNAT	NM_152419
A_33_P33823:	TPSD1	NM_012217
A_23_P39989:	KY	NM_178554
A_23_P10360:	MAN1C1	NM_020379
A_33_P32702!	OR1B1	NM_001004450
A_33_P32708(	CPSF3L	NM_017871
A_23_P25529:	HHATL	NM_020707
A_23_P14535:	PHF1	NM_002636
A_24_P27538(	BRI3BP	ENST00000341446
A_33_P32832:	YY2	NM_206923
A_23_P91657	EWSR1	NM_013986
A_23_P37187:	CDYL2	NM_152342
A_23_P94911	ZNF283	NM_181845
A_24_P28977	TRPC1	NM_003304
A_24_P29709!	PHF20	NM_016436
A_23_P13329:	MCTP1	NM_024717
A_24_P30269!	MRPL30	NM_145212
A_23_P25656:	TLR6	NM_006068
A_24_P40052!	TNRC18	NM_001080495
A_23_P31354:	GRK1	NM_002929
A_23_P15297(	RAPGEFL1	NM_016339
A_24_P14297:	PEX26	ENST00000329627
A_24_P12808!	RC3H2	NM_018835
A_23_P40995:	OAZ1	NM_004152
A_33_P32839(	NIP7	NM_016101
A_33_P32501:	SNX18	NM_052870
A_23_P87013	TAGLN	NM_001001522
A_33_P34331!	TRMU	NM_018006
A_23_P30986!	ZNF449	NM_152695
A_23_P15115(	FOXM1	NM_202002
A_24_P21391:	CENPB	NM_001810
A_24_P65292	SOX8	NM_014587
A_32_P41969:	ARHGEF5	NM_005435
A_33_P32562:	KRTAP10-5	NM_198694
A_33_P32945:	PRKCI	NM_002740
A_23_P55698	ZNF264	NM_003417
A_23_P41072!	ZNF491	NM_152356
A_33_P33989(	CALML4	NM_033429
A_23_P74458	KIAA0907	NM_014949
A_23_P55330	CYB5D2	NM_144611
A_23_P35973!	EPC2	NM_015630

A_33_P32284	FXYD2	NM_001680
A_32_P23331	EXOC8	NM_175876
A_33_P32173	PJA1	NM_145119
A_32_P13833	MED29	NM_017592
A_23_P89799	ACAA2	NM_006111
A_33_P34234	ZNF710	NM_198526
A_32_P32958	COX18	NM_173827
A_23_P9779	IRX5	NM_005853
A_23_P15473	ZNF133	NM_003434
A_23_P50261	HNRNPUL1	NM_007040
A_24_P22141	DYNC1I1	NM_004411
A_23_P20197	CREM	NM_183013
A_23_P12557	MID1IP1	NM_021242
A_23_P82550	STK17A	NM_004760
A_23_P20097	HYI	NM_001190880
A_32_P11745	C3orf59	NM_178496
A_33_P32292	BCAR1	NM_014567
A_23_P94683	NFX1	NM_002504
A_33_P32925	SURF1	NM_003172
A_23_P36584	EID2	NM_153232
A_24_P50934	NCKAP5	AK092189
A_23_P15601	GOLPH3	NM_022130
A_33_P33173	KRTAP5-4	NM_001012709
A_23_P20497	MBNL2	NM_144778
A_33_P33274	BACH1	NR_027655
A_32_P50965	BCDIN3D	NM_181708
A_33_P33099	HDAC2	NM_001527
A_24_P26910	NEUROG1	NM_006161
A_23_P10078	STAT5B	NM_012448
A_24_P10411	RHOF	NM_019034
A_23_P35399	ANXA11	NM_145869
A_24_P25105	EFHD2	NM_024329
A_33_P33782	MED25	NM_030973
A_33_P32194	TMEFF1	NM_003692
A_33_P32653	CEACAM4	NM_001817
A_23_P73429	HCLS1	NM_005335
A_23_P12022	LBH	NM_030915
A_23_P35964	NFAT5	NM_138714
A_23_P34744	CTSK	NM_000396
A_23_P21845	ILF3	NM_012218
A_23_P16235	HOXC5	NM_018953
A_23_P21042	MYL9	NM_181526
A_24_P14173	METAP2	NM_006838
A_23_P10717	MEOX1	NM_004527
A_32_P37657	TUBB4Q	NM_020040
A_32_P51512	ZNF837	NM_001129730
A_24_P38999	NADK	NM_023018
A_23_P40105	SOX2	NM_003106
A_24_P19473	ZSCAN22	NM_181846

A_23_P41305	NOXO1	NM_144603
A_23_P14015	IFI27L2	NM_032036
A_32_P17491	WAPAL	NM_015045
A_23_P13454	FOXP2	NM_148900
A_33_P32523	SNORD3B-1	NR_003271
A_23_P401	CENPF	NM_016343
A_23_P50050	FGFR3	NM_000142
A_23_P35507	CENPN	NM_001100624
A_24_P83615	NLRP1	NM_001033053
A_23_P10614	PDK1	NM_002610
A_23_P10943	ADORA2A	NM_000675
A_23_P78405	LIPG	NM_006033
A_32_P70736	DMRTA2	NM_032110
A_32_P95916	C6orf167	NM_198468
A_32_P52206	TYW1	NM_018264
A_23_P16565	SLC20A1	NM_005415
A_23_P14287	ATOH8	NM_032827
A_23_P11071	DUSP1	NM_004417
A_23_P14724	OSBPL10	NM_017784
A_24_P11106	ITSN1	NM_001001132
A_33_P32528	PHF12	NM_001033561
A_23_P21431	ICK	NM_016513
A_23_P40449	IL7R	NM_002185
A_32_P74615	SP5	NM_001003845
A_33_P34044	MIPEP	NM_005932
A_23_P11365	TUBB4	NM_006087
A_24_P25621	MAF	AF055376
A_24_P40945	ZNF167	NM_025169
A_23_P43490	CDKN2A	NM_058197
A_23_P38391	BTBD16	NM_144587
A_23_P44139	PRIM2	NM_000947
A_33_P32228	THBS3	CR933610
A_23_P28730	ZNF512B	NM_020713
A_24_P13991	MLX	NM_170607
A_23_P13680	ARHGAP11A	NM_014783
A_24_P28889	FAM101A	NM_181709
A_24_P32866	ARL13B	NM_182896
A_23_P24176	CCNJ	NM_019084
A_24_P21349	PTPRE	NM_006504
A_23_P50146	SIGLEC15	NM_213602
A_33_P33821	STARD13	NM_178006
A_33_P33780	TMEM18	NM_152834
A_32_P11701	ALDH1L2	NM_001034173
A_23_P87853	TMCC3	NM_020698
A_33_P36519	NEO1	NM_002499
A_24_P23857	ZNF518B	NM_053042
A_24_P83874	SERPINB6	NM_004568
A_24_P56689	ZNF205	NM_003456
A_33_P33521	LYPLAL1	NM_138794

A_32_P30693	RUVBL1	NM_003707
A_23_P10690	PPL	NM_002705
A_23_P49338	TNFRSF12A	NM_016639
A_24_P15380	SUPT16H	NM_007192
A_32_P20923	CITED4	NM_133467
A_23_P16280	HMGB1	NM_002128
A_23_P62881	SGIP1	NM_032291
A_23_P14904	PYCR2	NM_013328
A_24_P11403	EFNA3	NM_004952
A_33_P33298	CDK2AP2	NM_005851
A_23_P58188	MFAP3L	NM_001009554
A_23_P16874	NCAPG2	NM_017760
A_23_P27332	TCF4	NM_003199
A_24_P34168	MYH10	NM_005964
A_23_P35370	RP5-1022P6.2	NM_019593
A_32_P11210	SMARCC1	NM_003074
A_23_P37789	KRTAP11-1	NM_175858
A_33_P32934	FOXD4L2	NM_001099279
A_23_P15879	OPLAH	NM_017570
A_32_P35727	MSTO1	NM_018116
A_23_P25375	FAM54A	NM_138419
A_23_P37914	KRT74	NM_175053
A_23_P14749	BCORL1	NM_021946
A_23_P17085	IL1RAP	NM_002182
A_23_P13971	TNFRSF1A	NM_001065
A_33_P32894	ZNF785	NM_152458
A_33_P32273	MIER2	NM_017550
A_23_P11817	PLK1	NM_005030
A_24_P35074	KIAA0907	NM_014949
A_23_P15661	ZNF184	NM_007149
A_33_P32725	PLEKHG5	NM_198681
A_23_P25207	AHCYL2	NM_015328
A_32_P20523	TIGD2	NM_145715
A_23_P20609	LINGO1	NM_032808
A_33_P33263	SMG5	NM_015327
A_23_P31108	ZNF281	NM_012482
A_23_P15697	HUS1	NM_004507
A_24_P13836	EIF3M	NM_006360
A_23_P34813	MKL1	NM_020831
A_33_P32205	UBTD1	NM_024954
A_33_P33062	LYPD3	NM_014400
A_23_P34915	ATF3	NM_001040619
A_32_P35236	FBXL16	NM_153350
A_23_P14510	TFAP2B	NM_003221
A_23_P6746	IQSEC1	NM_014869
A_24_P45728	CGN	NM_020770
A_24_P15699	BIN1	NM_139346
A_23_P12443	ZNF718	NM_001039127
A_23_P41803	IFFO2	NM_001136265

A_23_P213772	CEP72	NM_018140
A_24_P365322	ZNF709	NM_152601
A_23_P122852	SMARCD3	NM_003078
A_23_P367676	SIN3A	NM_015477
A_23_P141208	DHRS7B	NM_015510
A_23_P60488	ODF2	NM_002540
A_23_P119578	KCNN1	NM_002248
A_23_P163373	ZNF774	NM_001004309
A_24_P62783	FABP3	NM_004102
A_23_P18372	B3GNT5	NM_032047
A_23_P116037	TM7SF2	NM_003273
A_32_P88538	FAM150B	NM_001002919
A_23_P135977	CKAP5	NM_001008938
A_24_P407717	GRB2	NM_002086
A_23_P251259	GTF2H4	NM_001517
A_23_P57534	DDX17	NM_006386
A_23_P218033	IKZF4	NM_022465
A_23_P109308	SLC5A3	NM_006933
A_23_P156842	EEF1E1	NM_004280
A_23_P388489	RPGRIP1L	NM_015272
A_23_P76488	EMP1	NM_001423
A_23_P1682	TMEM45B	NM_138788
A_23_P143147	TCFL5	NM_006602
A_33_P331622	SNAPC1	NM_003082
A_33_P341174	SIDT2	NM_001040455
A_33_P324747	KRTAP23-1	NM_181624
A_33_P340202	CCDC8	NM_032040
A_23_P423695	MXD4	NM_006454
A_23_P123073	PSMC2	ENST00000435765
A_32_P56661	IGF2	AK074614
A_33_P333727	NRARP	NM_001004354
A_23_P43164	SULF1	NM_015170
A_32_P478023	NPAS4	NM_178864
A_23_P119196	KLF2	NM_016270
A_33_P324316	MZF1	NM_198055
A_23_P93269	ZNF165	NM_003447
A_23_P3450	TUBGCP4	NM_014444
A_23_P250963	SLC1A3	NM_004172
A_32_P150891	DIAPH3	NM_001042517
A_23_P376124	RNASE8	NM_138331
A_23_P310274	PRSS2	NM_002770
A_24_P165656	PRKD3	NM_005813
A_24_P212851	SMARCA4	NM_003072
A_23_P166453	CDC42EP1	NM_152243
A_23_P33119	C8orf33	NM_023080
A_33_P336081	NFE4	NM_001085386
A_23_P159328	ANGPTL4	NM_139314
A_32_P6015	MNX1	NM_005515
A_23_P397376	MAF	NM_001031804

A_24_P30289	BAP1	NM_004656
A_23_P12765	ZNF202	NM_003455
A_24_P92053	GSTM3	NM_000849
A_33_P32506	TCF7	NM_003202
A_23_P15611	CYFIP2	NM_001037332
A_24_P91973	ZNF721	AK021514
A_23_P16418	ACSF2	NM_025149
A_24_P14367	PTBP1	NM_002819
A_23_P36204	C13orf27	NM_138779
A_23_P31253	FTSJ2	NM_013393
A_23_P37643	SLC10A4	NM_152679
A_23_P70699	ARID1B	NM_017519
A_23_P38905	ZNF317	NM_020933
A_23_P90649	IRS1	NM_005544
A_32_P38693	EPRS	NM_004446
A_23_P12740	KDM4D	NM_018039
A_32_P20221	SMARCE1	ENST00000348513
A_23_P16002	IFI16	NM_005531
A_23_P17018	OPLAH	NM_017570
A_23_P14655	MOBKL2B	NM_024761
A_23_P11640	ZBTB3	NM_024784
A_23_P26632	PYCARD	NM_013258
A_23_P2307	RHEBL1	NM_144593
A_23_P10398	HIST2H2AA4	NM_001040874
A_33_P33400	GINS4	NM_032336
A_23_P14052	FOXB1	NM_012182
A_23_P20252	ABLIM1	NM_001003408
A_24_P94389	SCUBE3	NM_152753
A_23_P50074	CBFA2T3	NM_005187
A_23_P82929	NOV	NM_002514
A_23_P97632	EPRS	NM_004446
A_23_P36327	WDR66	NM_144668
A_24_P17323	ZNF613	NM_024840
A_23_P12620	CLSPN	NM_022111
A_23_P79218	ACVR1	NM_001105
A_32_P11428	IKZF2	NM_001079526
A_32_P18336	CELF4	NM_020180
A_24_P39160	KRTAP6-3	NM_181605
A_23_P70219	TLX3	NM_021025
A_23_P63896	FAS	NM_000043
A_23_P34393	EGLN1	NM_022051
A_33_P33277	SYMPK	NM_004819
A_24_P34741	LRCH1	NM_001164213
A_23_P25189	C7orf27	NM_152743
A_23_P46539	PSRC1	NM_032636
A_23_P16554	HS1BP3	NM_022460
A_24_P7950	VAV3	NM_006113
A_23_P5221	ZNF333	NM_032433
A_23_P41604	TAF9	NM_001015892

A_23_P65481	TEP1	NM_007110
A_23_P74155	CITED4	NM_133467
A_23_P215214	LMBR1	NM_022458
A_23_P52101	CYB5R1	NM_016243
A_32_P76720	NT5DC3	NM_001031701
A_24_P192627	MLLT3	NM_004529
A_23_P327483	SPATA6	NM_019073
A_33_P324345	IGFL3	NM_207393
A_23_P21751[illegible]	CD99	NM_002414
A_32_P813273	MED27	NM_004269
A_24_P100551	SH3RF1	NM_020870
A_23_P207218	ALDH3A1	NM_000691
A_23_P64792	KCNMB4	NM_014505
A_24_P39152[illegible]	MAGED1	NM_001005333
A_23_P46982	MMS19	NM_022362
A_23_P8119	ZBTB9	NM_152735
A_33_P323685	TGFB1I1	NM_001042454
A_24_P330397	STRN3	NM_014574
A_23_P66658	TADA2A	NM_001488
A_23_P13946	SH2B3	NM_005475
A_23_P9744	PHF8	NM_001184898
A_24_P911928	MYBL1	NM_001144755
A_23_P10414[illegible]	ZMYM4	NM_005095
A_24_P7887	EP300	NM_001429
A_23_P130418	NDUFV2	NM_021074
A_33_P322683	F3	NM_001993
A_23_P154627	TSHZ2	NM_173485
A_23_P130677	LOC55908	NM_018687
A_33_P33880[illegible]	KRTAP21-2	NM_181617
A_33_P329981	NCRNA00292	NR_027286
A_23_P98995	CALCOCO1	NM_020898
A_33_P336798	ABCA12	NM_173076
A_24_P184031	PHPT1	NM_014172
A_24_P384954	QKI	NM_006775
A_32_P460039	ZNF282	NM_003575
A_23_P47728	MAP6	NM_033063
A_33_P32287[illegible]	KRTAP5-7	NM_001012503
A_23_P410859	ZSWIM6	NM_020928
A_32_P422741	CYLC1	NM_021118
A_23_P305863	MUC5B	NM_002458
A_23_P145175	ZNF318	NM_014345
A_33_P333572	INSR	NM_000208
A_32_P98291	SFMBT2	NM_001029880
A_24_P413884	CENPA	NM_001809
A_23_P33027	MLXIP	NM_014938
A_32_P65903	ZNF573	NM_152360
A_23_P333998	POLQ	AF090919
A_23_P30392	NEUROG1	NM_006161
A_23_P21776	SNAPC5	ENST00000316634

A_23_P12541[illegible]	ATP5I	NM_007100
A_23_P79145	ZNF570	NM_144694
A_23_P21868[illegible]	UBE2V1	NM_001032288
A_32_P54343[illegible]	PIK3C2G	NM_004570
A_32_P17999[illegible]	DMRTC1	NM_033053
A_33_P33642[illegible]	LBH	NM_030915
A_23_P41622[illegible]	ZNF579	NM_152600
A_23_P77223	MESP1	NM_018670
A_32_P22051[illegible]	CEP152	NM_014985
A_23_P16471[illegible]	SNRPA	NM_004596
A_23_P50003[illegible]	GLI2	NM_005270
A_23_P69329	HYAL1	NM_007312
A_32_P82034[illegible]	AMZ1	NM_133463
A_23_P57588	GTSE1	NM_016426
A_23_P58819	RANBP17	NM_022897
A_23_P14857[illegible]	MID2	NM_052817
A_23_P41785[illegible]	MYO3B	NM_138995
A_24_P11948[illegible]	SECISBP2	NM_024077
A_32_P19543	WDR63	NM_145172
A_23_P15162[illegible]	SUPT16H	NM_007192
A_23_P25346[illegible]	FAM175A	NM_139076
A_33_P33707[illegible]	CRX	NM_000554
A_23_P14109[illegible]	TFAP4	NM_003223
A_24_P27736[illegible]	CXCL5	NM_002994
A_24_P25709[illegible]	HJURP	NM_018410
A_23_P15729[illegible]	AEBP1	NM_001129
A_24_P71244	PIK3CD	NM_005026
A_23_P37967[illegible]	GNA13	NM_006572
A_23_P86382	ZNF669	NM_024804
A_23_P21628[illegible]	ARHGEF10	NM_014629
A_23_P65427	PSME2	NM_002818
A_23_P91640	ASPHD2	NM_020437
A_24_P10611[illegible]	PKD2	NM_000297
A_33_P33316[illegible]	THNSL1	NM_024838
A_32_P29806	CRADD	NM_003805
A_23_P30634	BACH2	NM_021813
A_32_P44535[illegible]	CD63	NM_001040034
A_23_P78808	SYMPK	NM_004819
A_23_P32936[illegible]	COG7	NM_153603
A_23_P20144[illegible]	PPPDE1	NM_016076
A_33_P33875[illegible]	DEPDC1	NM_001114120
A_23_P12203[illegible]	PPP2CA	NM_002715
A_23_P30121[illegible]	CRTC3	NM_022769
A_33_P34228[illegible]	GJC2	NM_020435
A_24_P67784	RIMBP3	NM_015672
A_23_P13578[illegible]	GOLGB1	NM_004487
A_23_P14056[illegible]	TTC23	NM_001040655
A_23_P15280[illegible]	RSAD1	NM_018346
A_23_P21789[illegible]	CCNL2	NM_030937

A_24_P22597	SGOL1	NM_001012409
A_24_P41343	NONO	NM_007363
A_23_P48790	UBE3A	NM_130839
A_32_P53680	TEAD1	NM_021961
A_23_P14270	LMAN2L	NM_030805
A_33_P32891	ZBTB42	NM_001137601
A_23_P33113	C8orf33	NM_023080
A_23_P13442	MICAL2	NM_014632
A_23_P20078	TGFBR3	NM_003243
A_33_P32904	ZNF668	NM_001172668
A_33_P33742	MKI67	NM_002417
A_24_P32464	ZNF544	NM_014480
A_24_P37331	NFATC3	NM_173165
A_23_P4212	HOXB13	NM_006361
A_32_P20705	EWSR1	NM_013986
A_23_P16077	IL6R	NM_000565
A_24_P39486	GOLM1	NM_016548
A_23_P16348	BUB1B	NM_001211
A_23_P37955	YARS	NM_003680
A_23_P42338	CREB3	NM_006368
A_23_P57989	TBL1XR1	NM_024665
A_23_P31123	CLASP1	NM_015282
A_23_P65584	COQ6	NM_182476
A_23_P21446	CCND3	NM_001760
A_23_P88848	DUS2L	NM_017803
A_23_P26233	PPIB	NM_000942
A_23_P41296	TBX19	NM_005149
A_24_P39998	HEPH	NM_014799
A_23_P12142	CDC25A	NM_001789
A_33_P33597	SARS	NR_034073
A_23_P33742	SERBP1	NM_001018067
A_23_P25774	SHB	NM_003028
A_24_P13574	GRTP1	NM_024719
A_23_P79251	EHD3	NM_014600
A_24_P28339	ADCK2	NM_052853
A_23_P20898	PTBP1	NM_031991
A_23_P16760	TRIP13	NM_004237
A_33_P32561	DUX4	NM_033178
A_23_P39731	MEIS1	BC036511
A_23_P14363	ASCC2	NM_032204
A_23_P20066	SERBP1	NM_001018067
A_23_P14243	MLLT1	NM_005934
A_24_P48856	CBS	NM_000071
A_23_P37549	CEBPA	NM_004364
A_23_P34488	CARNS1	NM_001166222
A_33_P33107	CTTN	NM_005231
A_23_P16722	DOCK10	NM_014689
A_23_P30411	ANKRD43	NM_175873
A_23_P37792	SFRS9	NM_003769

A_23_P13563	MAGED1	AF217963
A_23_P46375	PIGV	NM_017837
A_23_P15726	CLDN12	NM_012129
A_24_P23776	SEC14L1	NM_001143998
A_32_P51796	NANOG	NM_024865
A_23_P26094	SLTM	NM_024755
A_24_P24244	B4GALT2	NM_003780
A_32_P76576	MALT1	ENST00000348428
A_33_P32785	ZIK1	NM_001010879
A_23_P15449	DNMT3A	NM_175629
A_24_P17372	PEX10	NM_002617
A_23_P51311	LELP1	NM_001010857
A_23_P75973	RNF121	NM_018320
A_33_P33045	RNF207	NM_207396
A_23_P82554	STK17A	NM_004760
A_23_P10746	KRT31	NM_002277
A_23_P13758	GMEB1	NM_006582
A_24_P25138	NHEJ1	NM_024782
A_32_P44563	MBD5	NM_018328
A_24_P4373	C9orf91	NM_153045
A_24_P22078	RGS12	NM_198229
A_23_P12306	ITGB8	NM_002214
A_23_P21422	MARCKS	NM_002356
A_24_P36713	SCRT1	NM_031309
A_24_P5305	SPG7	NM_003119
A_32_P25146	IFFO2	NM_001136265
A_23_P25333	MNX1	NM_005515
A_23_P36177	CCND3	NM_001760
A_23_P20962	CYP1B1	NM_000104
A_33_P32220	SPHK1	NM_182965
A_23_P10638	SEMA7A	NM_003612
A_23_P11163	STYXL1	NM_016086
A_33_P32998	NCBP1	NM_002486
A_23_P20014	DCAF8	NM_015726
A_23_P31541	C20orf26	NM_015585
A_23_P25402	ZFP37	NM_003408
A_33_P33042	PLEKHG3	NM_015549
A_23_P11949	TNFAIP8L1	NM_001167942
A_23_P25274	DSCC1	NM_024094
A_24_P25533	SEC24C	NM_004922
A_23_P15080	PPFIBP2	NM_003621
A_32_P31256	KIAA1731	NM_033395
A_23_P13529	ALDH1B1	NM_000692
A_32_P42715	SHISA2	NM_001007538
A_23_P63153	DDX20	NM_007204
A_24_P31169	MAML1	NM_014757
A_23_P50288	CBFA2T2	NM_005093
A_23_P82600	NDUFB2	NM_004546
A_23_P14072	KRT8	NM_002273

A_23_P258544	TOMM20	NM_014765
A_23_P215088	ZC3HC1	NM_016478
A_24_P210675	NDE1	NM_017668
A_24_P347566	TLN2	NM_015059
A_23_P82839	AGPAT6	NM_178819
A_24_P150874	GNA13	NM_006572
A_23_P146922	GAS6	NM_000820
A_24_P6790	ATXN7L1	NM_020725
A_23_P500950	B3GNT2	NM_006577
A_23_P382602	BCL9	NM_004326
A_23_P500331	SSBP3	NM_001009955
A_23_P212830	FGFR3	NM_000142
A_23_P426809	ARHGEF11	NM_198236
A_23_P152837	CCL5	NM_002985
A_23_P202156	NFKB2	NM_001077493
A_24_P225308	ARID4B	NM_016374
A_23_P20722	SNAPC4	NM_003086
A_23_P68436	WFDC12	NM_080869
A_23_P66766	RPAIN	NM_001160246
A_23_P311616	JMJD6	NM_015167
A_33_P353934	MYO6	NM_004999
A_24_P419211	MTMR6	NM_004685
A_32_P96719	SHCBP1	NM_024745
A_23_P134755	TRPS1	NM_014112
A_23_P126939	RAB7L1	NM_003929
A_24_P367666	ASH1L	NM_018489
A_23_P421664	CAP2	NM_006366
A_33_P328178	NKX3-1	NM_006167
A_23_P303150	TMEM87B	NM_032824
A_24_P169937	NLRC3	NM_178844
A_24_P59596	ATAD2	NM_014109
A_23_P205489	SLC7A8	NM_182728
A_33_P338387	FEM1C	NM_020177
A_23_P350005	TRIML2	NM_173553
A_32_P104357	ESPL1	NM_012291
A_23_P126992	ABI1	NM_005470
A_32_P368237	CCDC74B	NM_207310
A_23_P68117	PSD4	NM_012455
A_23_P118476	SGSM2	NM_014853
A_23_P134805	NSMAF	NM_003580
A_23_P253853	KLHL8	NM_020803
A_33_P336799	TAF8	NM_138572
A_23_P51440	EPHA10	NM_001099439
A_23_P435513	TMEM106A	NM_145041
A_33_P349917	URM1	NM_001135947
A_23_P85682	NFIA	NM_005595
A_33_P327353	KRT81	NM_002281
A_23_P105453	KRT73	NM_175068
A_32_P508595	TIMELESS	NM_003920

A_23_P91221	PKIG	NM_181805
A_23_P33441	TRAF3IP3	NM_025228
A_23_P43369	PRDM15	NM_001040424
A_23_P35148	TAF13	NM_005645
A_23_P15314	MED1	NM_004774
A_23_P25189	C7orf27	NM_152743
A_33_P34973	GRIA4	NM_001077244
A_23_P20267	GDF6	NM_001001557
A_23_P37000	RIPK1	NM_003804
A_23_P20249	NOC3L	NM_022451
A_32_P35400	LHX9	NM_001014434
A_33_P32178	SMARCAL1	NM_014140
A_32_P14208	TTC3	NM_003316
A_23_P36812	AHCYL1	NM_006621
A_23_P10383	UBAP2L	NM_001127320
A_24_P53870	ZDHHC21	NM_178566
A_23_P21120	ADARB1	NM_001112
A_23_P52978	PRPF19	NM_014502
A_23_P41765	IRF1	NM_002198
A_32_P42075	C7orf28B	NM_198097
A_24_P14211	THBS1	NM_003246
A_23_P13030	TXNL4A	NM_006701
A_23_P33898	MAGED2	NM_201222
A_33_P32632	LRRC7	NM_020794
A_23_P77228	CRTC3	NM_022769
A_32_P40268	NDUFA3	ENST00000419113
A_33_P33975	KRTAP10-12	NM_198699
A_23_P73480	SCG5	NM_003020
A_33_P32520	P4HB	NM_000918
A_32_P21822	FAM109B	NM_001002034
A_23_P32558	WDR5	NM_017588
A_33_P34216	KIAA1147	NM_001080392
A_23_P12106	STAC	NM_003149
A_23_P16680	PCBP4	NM_033010
A_33_P34219	FRMPD3	ENST00000276185
A_33_P32557	NIF3L1	NM_001142356
A_23_P37514	C15orf39	NM_015492
A_23_P43020	C14orf145	NM_152446
A_23_P14084	MPHOSPH6	NM_005792
A_32_P11170	GYG2	NM_003918
A_23_P20809	MBD1	NM_015846
A_33_P32457	ZNF620	NM_175888
A_23_P38133	ZNF480	NM_144684
A_23_P36924	POLR1D	NM_015972
A_23_P13961	KRT86	NM_002284
A_23_P16373	GGA2	NM_015044
A_32_P33725	TPRG1	NM_198485
A_33_P32398	ANKDD1A	NM_182703
A_23_P20251	ASB13	NM_024701

A_23_P35646	AGPAT3	NM_020132
A_32_P39716	ZNF385C	ENST00000393873
A_23_P25969	PSAT1	NM_058179
A_23_P34556	OPRL1	NM_182647
A_24_P34884	KRTAP15-1	NM_181623
A_23_P10414	BANP	NM_079837
A_23_P13294	ELF2	NM_201999
A_23_P43557	DENND1A	NM_024820
A_32_P17753	IRX6	NM_024335
A_24_P62237	CERK	NM_022766
A_23_P42909	TMEM139	NM_153345
A_33_P32527	ZNF788	NR_027049
A_24_P16040	CDCP1	NM_178181
A_23_P32449	KIAA0355	NM_014686
A_23_P30571	RYBP	NM_012234
A_23_P41318	SFRS12IP1	NM_173829
A_23_P10665	CA7	NM_001014435
A_23_P42595	BCL2L11	NM_138621
A_33_P33453	ZFP64	NM_199427
A_23_P70445	HIST1H3E	NM_003532
A_33_P33742	MKI67	NM_002417
A_33_P32236	LHX3	NM_014564
A_23_P33930	LIN52	NM_001024674
A_23_P14136	FZD2	NM_001466
A_23_P25841	WNT7A	NM_004625
A_33_P32444	LRRC1	NM_018214
A_23_P10846	WDR35	NM_001006657
A_23_P35553	USP54	NM_152586
A_33_P33695	MANEAL	NM_001031740
A_32_P16451	KLHDC1	NM_172193
A_32_P13584	ERCC6L	NM_017669
A_32_P59777	KIAA1609	NM_020947
A_23_P93499	TFB1M	NM_016020
A_33_P32317	ZNF677	ENST00000333952
A_32_P31932	PSMB9	NM_002800
A_23_P13742	ZMPSTE24	NM_005857
A_23_P12679	SARS	NM_006513
A_24_P92840	SATB2	AK025127
A_33_P32377	PORCN	NM_203473
A_23_P16230	IRAK3	NM_007199
A_24_P85300	HEG1	NM_020733
A_23_P46245	HEYL	NM_014571
A_32_P48047	RNF187	NM_001010858
A_23_P40200	ZNF527	NM_032453
A_23_P25464	FBXW2	NM_012164
A_23_P42629	MAPK14	NM_001315
A_23_P36931	VANGL1	NM_138959
A_23_P42033	PGBD4	NM_152595
A_23_P43139	SPOCD1	NM_144569

A_23_P22190	ESRRB	X51417
A_24_P56884	TMEM199	NM_152464
A_24_P64591	EFNA5	NM_001962
A_24_P91873	ABHD1	BC028378
A_23_P15576	ANKRD40	NM_052855
A_23_P30983	STON2	NM_033104
A_24_P92260	NUP160	NM_015231
A_23_P11248	AQP3	NM_004925
A_33_P32302	NCAPG	NM_022346
A_33_P32384	ALDH3A1	NM_001135168
A_24_P37670	HDGF	NM_004494
A_23_P41496	ZNF584	NM_173548
A_23_P57347	PCNT	NM_006031
A_32_P20906	ZNF664	NM_152437
A_23_P37412	DRD5	NM_000798
A_32_P85539	HCFC2	NM_013320
A_33_P32201	SBNO2	NM_014963
A_23_P48610	TMEM55B	NM_144568
A_23_P10012	CASC5	NM_170589
A_33_P32385	DENND4B	NM_014856
A_23_P25695	KIF20A	NM_005733
A_33_P32966	KRT33A	NM_004138
A_23_P20913	LAIR1	NM_002287
A_33_P32500	EVI5L	NM_145245
A_23_P10982	TADA3	NM_006354
A_24_P17578	ARHGEF12	NM_015313
A_23_P14766	OLFML1	NM_198474
A_23_P20189	NFASC	NM_015090
A_23_P32150	DHRS2	NM_182908
A_33_P32710	KRTAP5-9	NM_005553
A_33_P32792	GRIP2	NM_001080423
A_23_P33148	LCMT1	NM_016309
A_24_P37633	CCNL2	NM_001039577
A_24_P12729	TTC7B	NM_001010854
A_23_P31732	MECOM	NM_005241
A_23_P20005	NADK	NM_023018
A_33_P32679	SLC25A23	NM_024103
A_23_P15662	PGBD1	NM_032507
A_23_P39051	TNFRSF11A	NM_003839
A_32_P35453	ZNF286A	NM_020652
A_23_P37085	RDH12	NM_152443
A_32_P45552	ZNRD1	NM_170783
A_23_P43489	CARD10	NM_014550
A_24_P93829	HES1	NM_005524
A_32_P84265	ATP5L	NM_006476
A_24_P40912	FNDC3A	AK056071
A_33_P32431	COQ5	NM_032314
A_23_P74344	TADA1	NM_053053
A_23_P42208	TMEM55A	NM_018710

A_24_P27774	CNPY3	NM_006586
A_23_P23443	EFHD2	NM_024329
A_23_P44264	EMX2	NM_004098
A_33_P33188	TBC1D8B	NM_198881
A_23_P16844	EPHB4	NM_004444
A_23_P53039	LDHC	NM_002301
A_32_P20511	FOXC1	NM_001453
A_23_P32192	CCL3L3	NM_001001437
A_23_P33247	ANAPC7	NM_016238
A_23_P62953	PBX1	NM_002585
A_23_P71270	AZGP1	NM_001185
A_33_P34116	CDKN2A	NM_058197
A_32_P87112	POM121L4P	NR_024592
A_23_P30423	RAPGEF1	NM_198679
A_33_P32724	ARFGAP2	NM_032389
A_23_P13950	BHLHE41	NM_030762
A_23_P17051	DYM	NM_017653
A_23_P36862	CHRNB2	NM_000748
A_32_P84189	FAM83D	NM_030919
A_23_P14762	FES	NM_002005
A_24_P34231	ODZ4	NM_001098816
A_23_P11764	YY1	NM_003403
A_23_P33590	STK36	NM_015690
A_23_P15545	UCN2	NM_033199
A_33_P32617	SLC35A2	NM_005660
A_23_P17570	ZNF341	NM_032819
A_23_P19902	EVX1	NM_001989
A_33_P33334	EMILIN1	NM_007046
A_24_P21880	HOXC10	NM_017409
A_24_P13790	CES2	NM_003869
A_23_P10912	EBF4	NM_001110514
A_23_P38516	METTL7B	NM_152637
A_23_P10944	HPS4	NM_022081
A_23_P39718	FEZ2	NM_001042548
A_32_P14821	ANKRD43	NM_175873
A_23_P99226	SIRT4	NM_012240
A_24_P37264	RNF144A	NM_014746
A_23_P54816	ATP6V0C	NM_001694
A_23_P36962	DNAJC3	NM_006260
A_32_P37827	FAM117B	NM_173511
A_23_P14906	PTPRU	NM_005704
A_23_P12210	SH3PXD2B	NM_001017995
A_33_P32639	MXI1	NM_005962
A_32_P11311	ZNF561	NM_152289
A_23_P32543	XPO6	NM_015171
A_24_P10170	ROR2	NM_004560
A_33_P32972	NAAA	NM_001042402
A_23_P6085	SLC23A2	NM_203327
A_23_P42086	NOD2	NM_022162

A_23_P62420	OPHN1	NM_002547
A_23_P83749	C11orf63	AK027207
A_24_P16311	CDV3	NM_017548
A_23_P37188	ARL17P1	NM_016632
A_24_P17561	SFXN2	NM_178858
A_33_P32752	NRK	NM_198465
A_23_P16378	MMP2	NM_004530
A_23_P21888	DVL3	NM_004423
A_23_P4782	CACNG7	NM_031896
A_32_P77972	UTP11L	NM_016037
A_23_P65812	SMAD6	NM_005585
A_32_P78783	ZNF778	NM_182531
A_32_P14172	COMMD7	NM_053041
A_23_P13617	CSF2RA	NM_006140
A_33_P33492	PIAS2	NM_173206
A_23_P57236	GGT7	NM_178026
A_33_P32337	LOC10012875	XR_108302
A_24_P20677	CRYAB	NM_001885
A_23_P15206	UBR1	NM_174916
A_23_P11855	HEXIM1	NM_006460
A_23_P16709	IDUA	NM_000203
A_23_P21734	TAF9	NM_001015891
A_24_P94137	ZNF473	NM_015428
A_23_P51820	RORC	NM_005060
A_23_P14193	RFC3	NM_002915
A_23_P25268	PCYT1A	NM_005017
A_33_P34047	SEL1L	NM_005065
A_33_P33733	GJC1	NM_005497
A_24_P31506	ZMIZ2	NM_031449
A_24_P25668	DRD4	NM_000797
A_23_P25977	HEATR3	ENST00000405152
A_32_P23475	TMEM55A	NM_018710
A_24_P37961	PLB1	NM_153021
A_32_P11595	TCERG1L	NM_174937
A_23_P15060	IGF2	NM_000612
A_23_P35894	PML	NM_033244
A_24_P24532	PRKAR1A	NM_212472
A_33_P32816	NLRP3	NM_004895
A_24_P29842	PRMT5	NM_001039619
A_32_P48137	KRTAP11-1	NM_175858
A_32_P75415	IPP	NM_001145349
A_23_P72697	GPIHBP1	NM_178172
A_24_P92558	ZNF674	NM_001039891
A_23_P8139	ZNF76	NM_003427
A_23_P69908	GLRX	NM_002064
A_33_P33986	TFEB	NM_007162
A_32_P82067	PRDM8	NM_020226
A_23_P36996	NCAPH2	NM_014551
A_24_P33036	RCOR1	NM_015156

A_23_P35286[illegible]	PVRL2	NM_002856
A_32_P15021	RICTOR	NM_152756
A_32_P10126[illegible]	TMEM20	ENST00000371408
A_33_P32963[illegible]	NTM	NM_001144058
A_23_P25220[illegible]	EAF2	NM_018456
A_24_P58671[illegible]	TPRG1	NM_198485
A_32_P43945[illegible]	GRAMD2	NM_001012642
A_23_P21555[illegible]	MEOX2	NM_005924
A_23_P30105[illegible]	PAIP2B	NM_020459
A_23_P90297	DYRK1B	NM_004714
A_24_P18249[illegible]	DUSP10	NM_007207
A_32_P80025[illegible]	PART1	NR_024617
A_23_P10820[illegible]	NOTCH3	NM_000435
A_24_P24805[illegible]	TOP1MT	NM_052963
A_24_P65616	PVR	NM_006505
A_23_P10047[illegible]	MYST1	NM_032188
A_24_P63453[illegible]	CPPED1	NM_018340
A_32_P49730[illegible]	RPH3AL	NM_006987
A_24_P38843[illegible]	PPP2R3A	NM_002718
A_23_P35995	ASAM	NM_024769
A_24_P28646[illegible]	PURB	NM_033224
A_33_P33356[illegible]	PPP1R14A	NM_033256
A_23_P15576[illegible]	HMGB2	NM_002129
A_23_P34990[illegible]	KCNH7	NM_033272
A_33_P32937[illegible]	KRTAP10-7	NM_198689
A_23_P40267[illegible]	CD1A	NM_001763
A_23_P46315	DENND2C	ENST00000369540
A_23_P15935[illegible]	POLE	NM_006231
A_23_P60069	VDAC3	NM_005662
A_32_P14598[illegible]	PRDM15	NM_001040424
A_23_P11696[illegible]	PARP11	NM_020367
A_23_P32438[illegible]	RPS4Y2	NM_001039567
A_24_P56467	GMPR2	NM_001002000
A_23_P38037[illegible]	IPMK	NM_152230
A_32_P30392[illegible]	DAB1	ENST00000371236
A_32_P71191[illegible]	LOC390561	XM_001716926
A_23_P20621[illegible]	THBS1	NM_003246
A_24_P84962[illegible]	RDBP	NM_002904
A_33_P32475[illegible]	ZNF805	NM_001023563
A_23_P33692[illegible]	MED29	NM_017592
A_23_P11186[illegible]	ZSCAN21	NM_145914
A_23_P21862[illegible]	NEU4	NM_080741
A_23_P50121[illegible]	ZNF398	NM_020781
A_23_P15028[illegible]	TP53I11	NM_001076787
A_24_P32708[illegible]	PRSS33	NM_152891
A_23_P53057	ZNF215	NM_013250
A_24_P19443[illegible]	KRTAP4-1	NM_033060
A_32_P47947[illegible]	KRT78	NM_173352
A_24_P18285[illegible]	LBH	NM_030915

A_23_P371824	TUFT1	NM_020127
A_24_P392632	TRPM3	NM_206948
A_23_P129925	SLFN11	NM_152270
A_23_P96497	HSFX1	NM_016153
A_23_P66815	SIRT7	NM_016538
A_23_P383819	TBX3	NM_016569
A_23_P87036	APOA4	NM_000482
A_33_P323169	ZNF667	NM_022103
A_23_P106275	FLJ10038	NR_026891
A_32_P31618	GSR	ENST00000221130
A_24_P33770	AFAP1L2	NM_032550
A_23_P144592	HSPA4L	ENST00000296464
A_23_P13147	C11orf71	CR597454
A_32_P139654	SS18L1	NM_198935
A_23_P216259	TPD52	NM_001025252
A_23_P117819	C15orf38	NM_182616
A_23_P89621	CBX4	NM_003655
A_23_P110882	TSPYL4	NM_021648
A_24_P945408	ARMC9	AB058771
A_23_P433753	PRKAR1A	NM_212472
A_32_P106117	GGA2	NM_015044
A_24_P217572	EDNRA	NM_001957
A_24_P318939	ZNF337	NM_015655
A_23_P22409	FUT10	NM_032664
A_32_P155811	CD2AP	NM_012120
A_23_P94358	MTUS1	NM_001001924
A_32_P139588	IPO7	NM_006391
A_32_P72294	PGCP	NM_016134
A_32_P441655	JPH1	NM_020647
A_23_P141302	ZNF18	NM_144680
A_23_P311875	CD6	NM_006725
A_24_P39211	ING1	NM_198219
A_24_P127828	VCP	NM_007126
A_33_P334066	KRTAP19-5	NM_181611
A_24_P159866	STK11	NM_000455
A_23_P150064	MMRN2	NM_024756
A_32_P511222	CLASP1	NM_015282
A_23_P28595	DLX2	NM_004405
A_33_P342344	ZNF720	NM_001130913
A_23_P123785	EGFL7	NM_201446
A_23_P436138	MAX	NM_145114
A_23_P409462	DCBLD1	NM_173674
A_24_P251534	CTDSPL	NM_001008392
A_23_P70278	ROS1	NM_002944
A_23_P369574	CLASP2	NM_015097
A_24_P7179	DIP2A	NM_206890
A_23_P104541	VAX1	NM_001112704
A_24_P99963	CSNK1G2	NM_001319
A_23_P96483	ZBTB33	NM_006777

A_23_P26582	GNAO1	NM_020988
A_24_P20096	ING3	ENST00000315870
A_23_P10590	ATP11A	NM_032189
A_32_P21479	IRAK1BP1	NM_001010844
A_24_P36243	MDM4	NM_002393
A_23_P76249	KRT6B	NM_005555
A_33_P32172	ATAD2	NM_014109
A_33_P33117	ZNF789	NM_213603
A_23_P39915	IL12RB1	NM_153701
A_23_P11664	SLC26A10	NM_133489
A_23_P15979	PNPLA4	NM_004650
A_24_P22675	TOX	NM_014729
A_32_P8402	SYNCRIP	NM_006372
A_23_P12093	APOBEC3C	NM_014508
A_23_P13854	AKR1C3	NM_003739
A_23_P15226	RNPS1	NM_006711
A_23_P12723	FRAT1	NM_005479
A_24_P87763	EEF2	NM_001961
A_23_P12599	E2F2	NM_004091
A_23_P7873	MCM3	NM_002388
A_33_P38317	MGC4859	BC002644
A_32_P52552	ITPRIPL1	NM_178495
A_23_P10759	ATP8B1	NM_005603
A_23_P47077	BAG3	NM_004281
A_23_P13599	ARV1	NM_022786
A_33_P33197	NR4A1	NM_002135
A_24_P17677	TLR6	NM_006068
A_23_P87199	FTH1	NM_002032
A_33_P33127	KRTAP4-3	NM_033187
A_23_P20951	DNAJB2	NM_001039550
A_23_P12140	RPL5	NM_000969
A_23_P86461	PLAC9	ENST00000372263
A_33_P32741	TP53I13	NM_138349
A_24_P38558	TMEM18	NM_152834
A_23_P77813	FN3KRP	NM_024619
A_24_P94518	RBM15B	NM_013286
A_23_P50887	LANCL1	NM_006055
A_23_P16403	PSMC5	NM_002805
A_24_P17517	PHTF2	NM_020432
A_23_P14452	ADAMTS3	NM_014243
A_23_P19455	MDC1	NM_014641
A_33_P32625	RASA3	NM_007368
A_23_P14782	EPS8L2	NM_022772
A_32_P70818	PAX9	NM_006194
A_33_P34046	MKS1	NM_017777
A_23_P16882	KLF10	NM_005655
A_23_P27734	NPAS1	NM_002517
A_33_P32456	TTC39A	NM_001080494
A_24_P31284	CEND1	NM_016564

A_33_P32934	OPN3	NM_014322
A_23_P67042	MOCOS	NM_017947
A_23_P41823	PHLPP2	NM_015020
A_23_P25501	UGGT1	NM_020120
A_23_P11716	RCBTB1	NM_018191
A_33_P33982	FOXP3	NM_014009
A_23_P25628	GOLGB1	NM_004487
A_23_P16758	UBE2B	NM_003337
A_23_P57304	TMEM50B	NM_006134
A_23_P15542	HSD17B1	NM_000413
A_33_P33380	ZNF470	NM_001001668
A_23_P39619	UBAC2	NM_177967
A_23_P11267	ZWILCH	NM_017975
A_33_P32529	SCRT2	NM_033129
A_23_P13771	POGK	NM_017542
A_23_P14369	SH3BP1	NM_018957
A_23_P887	IKBKE	NM_014002
A_33_P32510	SLC36A1	NM_078483
A_24_P15036	HSP90B1	NM_003299
A_23_P70757	FOXO3	NM_001455
A_32_P10407	PAK2	NM_002577
A_32_P14470	CTBP2	NM_022802
A_33_P33630	PRPF19	NM_014502
A_23_P9416	ACO1	NM_002197
A_24_P91701	DYNC2H1	NM_001377
A_33_P32837	ZBTB7A	NM_015898
A_32_P15502	NFIA	NM_001134673
A_23_P40506	CRABP2	NM_001878
A_23_P25011	HSPBAP1	NM_024610
A_24_P22212	TH1L	NM_198976
A_23_P72924	HMX1	NM_018942
A_23_P11793	GCSH	NM_004483
A_24_P23094	PER3	NM_016831
A_24_P27387	PRDX3	AF118073
A_23_P13543	EXOC4	NM_021807
A_32_P49654	RPS2	BC020336
A_23_P20848	CLEC4M	NM_001144904
A_23_P35035	LPAR3	NM_012152
A_23_P21556	AHR	NM_001621
A_23_P15202	TRIP4	NM_016213
A_23_P38511	ZBTB10	NM_023929
A_24_P40633	STEAP1	NM_012449
A_23_P21905	BAT1	NM_004640
A_32_P18390	SHF	NM_138356
A_23_P16743	PGM5P2	NR_002836
A_24_P91851	P4HTM	BC018731
A_23_P42967	LCOR	NM_032440
A_23_P12807	SMARCC2	NM_139067
A_33_P32997	NODAL	NM_018055

A_33_P32717[illegible]	CCDC33	NM_025055
A_32_P58280	EXOC6	NM_019053
A_23_P73543	CCDC120	NM_033626
A_23_P10119[illegible]	MYO5B	NM_001080467
A_32_P92783	STIP1	NM_006819
A_24_P28829[illegible]	KIR2DL4	NM_002255
A_23_P33774[illegible]	NRL	NM_006177
A_23_P87560	BTG1	NM_001731
A_23_P50235[illegible]	RFX2	NM_000635
A_32_P40281[illegible]	BMS1	NM_014753
A_23_P10487[illegible]	SPA17	NM_017425
A_23_P21268[illegible]	MECOM	NM_005241
A_23_P41979[illegible]	SBF2	NM_030962
A_23_P20157[illegible]	CELSR2	NM_001408
A_23_P99710	KIAA0586	NM_014749
A_23_P41127[illegible]	SLC5A2	NM_003041
A_24_P28947[illegible]	RNASET2	NM_003730
A_23_P1722	C11orf52	NM_080659
A_23_P79458	LBX2	NM_001009812
A_32_P18106	GALK2	NM_002044
A_32_P31157[illegible]	TSC22D3	NM_004089
A_23_P93321	TCF19	NM_007109
A_23_P20826[illegible]	RPS5	NM_001009
A_33_P33362[illegible]	IRX1	NM_024337
A_33_P32624[illegible]	ENST0000031[illegible]	ENST00000312015
A_23_P97610	GLT25D2	NM_015101
A_32_P11319[illegible]	ZNF592	NM_014630
A_23_P21185[illegible]	ABHD6	NM_020676
A_23_P88303	HSPA2	NM_021979
A_24_P25213[illegible]	PPARD	NM_006238
A_23_P76951	TMX1	NM_030755
A_24_P30810[illegible]	FRYL	NM_015030
A_23_P14569[illegible]	ASNS	NM_001673
A_33_P32148[illegible]	NEB	NM_001164507
A_23_P41598[illegible]	NPAS2	NM_002518
A_23_P64193	ALG9	NM_001077691
A_23_P12221[illegible]	LOX	NM_002317
A_23_P13404[illegible]	TBX18	NM_001080508
A_23_P11914[illegible]	KEAP1	NM_203500
A_32_P64497	SYNJ2	NM_003898
A_23_P10769[illegible]	ZNF586	NM_017652
A_32_P12368[illegible]	FNTA	NM_002027
A_32_P14708[illegible]	ARHGAP11B	NM_001039841
A_24_P11071[illegible]	ZNF236	NM_007345
A_23_P35783[illegible]	SDR42E1	NM_145168
A_24_P18444[illegible]	MMP19	NM_002429
A_23_P25766[illegible]	TTC26	NM_024926
A_24_P67534	LOC644538	NM_001163438
A_23_P43273	EXT1	NM_000127

A_33_P321488	KPNA3	NM_002267
A_23_P371865	CDYL2	NM_152342
A_23_P108216	FXYD1	NM_005031
A_23_P19257	SLC35B2	NM_178148
A_23_P213966	TIGD6	NM_030953
A_23_P170908	NUBPL	NM_025152
A_32_P163402	NIPAL1	NM_207330
A_23_P66753	PITPNM3	NM_031220
A_23_P138435	ZMIZ1	NM_020338
A_23_P136347	EPS8	NM_004447
A_24_P406986	SLC43A3	NM_199329
A_23_P20434	TIGD5	NM_032862
A_24_P318544	CSNK1D	AB209463
A_23_P67932	IL8RA	NM_000634
A_24_P367752	NDST1	BC012888
A_23_P403460	DLX1	NM_178120
A_23_P164623	ZBTB45	NM_032792
A_23_P204721	AQP2	NM_000486
A_23_P99853	KIAA1370	NM_019600
A_23_P1211	HTR7	NM_000872
A_23_P315843	NCOA5	NM_020967
A_32_P149936	ZNF407	NM_017757
A_23_P147245	OSBPL10	NM_017784
A_23_P64217	MLL	AK021845
A_24_P257201	MRPL30	NM_145212
A_24_P326511	SORBS1	NM_001034954
A_23_P253395	UNC13B	NM_006377
A_23_P345575	FOXO3	NM_001455
A_23_P393034	HAS3	NM_005329
A_23_P9186	ACTL7B	NM_006686
A_32_P910493	CCNT1	NM_001240
A_32_P192547	TCEAL6	NM_001006938
A_23_P120428	TP53TG5	NM_014477
A_23_P54556	MKL2	NM_014048
A_23_P38085	CARHSP1	NM_001042476
A_23_P97700	TXNIP	NM_006472
A_32_P434352	ZNF524	NM_153219
A_32_P169138	CLCN7	NM_001287
A_23_P112004	LRRC6	NM_012472
A_23_P82121	ARHGAP18	NM_033515
A_32_P840590	YRDC	AK090770
A_24_P217904	TRERF1	NM_033502
A_23_P45022	PPID	NM_005038
A_32_P149484	NBPF11	NM_183372
A_23_P379146	KRT74	NM_175053
A_23_P45087	ZNF107	NM_016220
A_24_P200420	SLC7A11	NM_014331
A_23_P250035	SDHA	NM_004168
A_23_P11237	TAF1	NM_004606

A_33_P37472(	ENST0000031	ENST00000317596
A_32_P22191	BBX	NM_020235
A_23_P3237	ELL3	NM_025165
A_33_P34031	NR2F1	NM_005654
A_23_P30389	LCE1C	NM_178351
A_33_P33425	P2RY1	NM_002563
A_23_P45717	SKI	NM_003036
A_23_P13663	FAM60A	NM_021238
A_33_P32514	NRIP1	NM_003489
A_23_P39062	PACRGL	NM_145048
A_23_P32063	RPL22	NM_000983
A_24_P23245	NDUFA6	NM_002490
A_33_P33814	ZNF331	NM_018555
A_32_P78792	LAMA3	NM_198129
A_23_P42331	HMGA1	NM_145901
A_23_P39192	LPHN1	NM_001008701
A_23_P79554	KCNH7	NM_033272
A_32_P10068	TTC28	NM_001145418
A_33_P32137	CDK5RAP1	NM_016082
A_24_P28516	POGK	NM_017542
A_23_P14902	EFNA3	NM_004952
A_23_P20026	PCNXL2	NM_014801
A_23_P42254	USP22	NM_015276
A_33_P33884	PQLC2	NM_001040125
A_32_P86242	EFHC1	NM_018100
A_23_P11006	EIF2B5	NM_003907
A_23_P16219	OVCH2	NM_198185
A_23_P91769	NDUFA6	NM_002490
A_23_P13118	GBX2	NM_001485
A_33_P34200	LOC728690	AK094642
A_32_P42409	CHRNA5	NM_000745
A_23_P13133	SMARCAL1	NM_014140
A_23_P94800	S100A4	NM_002961
A_23_P64044	FERMT3	NM_178443
A_23_P55911	ZNF440	NM_152357
A_24_P22142	ZNF784	NM_203374
A_24_P30257	MYOD1	NM_002478
A_24_P94511	ACVRL1	NM_000020
A_23_P20984	TGFA	NM_003236
A_23_P13873	MLF2	NM_005439
A_23_P16650	SREBF2	ENST00000361204
A_23_P35004	REEP5	NM_005669
A_33_P33605	ZNF444	NM_018337
A_23_P21485	PID1	NM_017933
A_23_P31713	E2F5	NM_001951
A_23_P21368	CXXC5	ENST00000302517
A_23_P36877	ZNF114	NM_153608
A_23_P41985	TAF1L	NM_153809
A_23_P30471	HES2	NM_019089

A_32_P15512	C1orf194	NM_001122961
A_24_P66161	PTPRB	NM_001109754
A_24_P63563	KIAA0141	NM_014773
A_23_P32211	TFAP2E	NM_178548
A_23_P11013	WDR19	NM_025132
A_33_P32961	CCL3L3	NM_001001437
A_33_P33404	SCLT1	NM_144643
A_33_P32928	KRT6A	NM_005554
A_23_P7388	HDAC3	NM_003883
A_23_P12621	CLSPN	NM_022111
A_24_P35926	AKT2	NM_001626
A_23_P25134	GSK3B	NM_002093
A_23_P15323	CHMP2A	NM_014453
A_33_P32590	CSNK1G2	NM_001319
A_33_P33592	HMG20B	NM_006339
A_23_P7337	EDNRA	NM_001957
A_24_P36097	RPAIN	NM_001160266
A_23_P74241	MTF1	NM_005955
A_23_P10452	NEBL	NM_006393
A_23_P21202	ZBTB38	NM_001080412
A_23_P10657	GOT2	NM_002080
A_23_P76515	LASS5	NM_147190
A_24_P65271	C16orf58	NM_022744
A_24_P82200	MEIS2	NM_170676
A_23_P26221	GOLGA8A	NM_181077
A_23_P19673	SGK1	NM_005627
A_32_P61903	WNT5A	NM_003392
A_23_P25873	WDHD1	NM_007086
A_23_P34366	HTR1D	NM_000864
A_33_P32403	PITX1	NM_002653
A_32_P74340	GLYR1	NM_032569
A_23_P29185	TTC28	NM_001145418
A_23_P36696	ASXL1	NM_015338
A_23_P46170	MED8	NM_052877
A_32_P23854	DENND1A	NM_020946
A_23_P25683	TTC15	NM_016030
A_23_P50137	MEX3C	NM_016626
A_23_P11612	CHEK1	NM_001274
A_33_P35386	HIST2H4A	CD243491
A_24_P15656	FAM127A	NM_001078171
A_23_P52939	SLC43A1	NM_003627
A_23_P45199	SLC7A11	NM_014331
A_23_P50219	IDH3B	NM_174855
A_23_P20418	PYROXD1	NM_024854
A_23_P19624	BMP6	NM_001718
A_23_P843	TOMM40L	NM_032174
A_33_P33422	CDC42EP5	NM_145057
A_23_P98850	DDIT3	NM_004083
A_23_P12014	TGFBRAP1	NM_004257

A_33_P331305	NOTCH3	NM_000435
A_23_P256526	ZNF80	NM_007136
A_23_P17786	PITPNB	NM_012399
A_23_P253650	HEG1	NM_020733
A_24_P772103	PITPNC1	AK094724
A_23_P64204	ZW10	NM_004724
A_32_P445408	UHRF1	NM_013282
A_23_P4153	ARL17P1	NM_001113738
A_23_P434442	TCEAL3	NM_001006933
A_33_P334570	CREB3L4	NM_130898
A_23_P8906	LRP12	NM_013437
A_23_P97990	HTRA1	NM_002775
A_24_P234196	RRM2	NM_001034
A_32_P9543	APOBEC3A	NM_145699
A_32_P444278	LOC729915	XM_001722873
A_23_P167324	PKD2	NM_000297
A_23_P421379	IGF2	NM_000612
A_24_P942604	SMC1A	NM_006306
A_33_P334971	ZNF766	NM_001010851
A_24_P98762	PPP2R1B	NM_002716
A_23_P381451	SP2	NM_003110
A_23_P13772	TBX3	NM_016569
A_33_P328942	ZNF775	NM_173680
A_23_P153776	USF2	NM_003367
A_33_P327667	VSX1	NM_199425
A_33_P335153	PTK2B	NM_173174
A_32_P66190	CABLES2	NM_031215
A_24_P20200	PLEKHB2	NM_017958
A_32_P333448	AR	ENST00000374690
A_23_P116712	RASSF8	NM_007211
A_23_P61463	FOXN2	NM_002158
A_24_P404822	APLN	NM_017413
A_24_P385148	ZNF197	NM_006991
A_23_P54866	PHLPP2	NM_015020
A_23_P54891	C16orf58	NM_022744
A_24_P295543	BLOC1S2	NM_001001342
A_23_P23194	PINK1	NM_032409
A_23_P360353	STX10	NM_003765
A_32_P192334	THBD	NM_000361
A_33_P328774	MYST1	NM_182958
A_23_P207614	KIAA0100	NM_014680
A_23_P45365	COL4A5	NM_033380
A_23_P101761	KISS1R	NM_032551
A_33_P341199	RABGAP1	NM_012197
A_23_P110569	TRIM36	NM_018700
A_23_P127322	NODAL	NM_018055
A_33_P332129	IQGAP3	NM_178229
A_23_P416894	PION	NM_017439
A_24_P337657	SRF	NM_003131

A_23_P25305	ASCL1	NM_004316
A_24_P23587(	RNF121	NM_018320
A_23_P20914(	ZNF91	NM_003430
A_33_P33719(	IGSF1	NM_205833
A_23_P14964(	SDHB	NM_003000
A_23_P12546:	BPTF	NM_182641
A_23_P11071	PORCN	NM_203473
A_33_P32332!	S100A7L2	NM_001045479
A_23_P13102(	ZBTB32	NM_014383
A_33_P32858(	CYGB	NM_134268
A_23_P47885	LRIG3	NM_153377
A_33_P32217:	MGAT4C	NM_013244
A_23_P10670(	RPS2	NM_002952
A_23_P10525:	GLI1	NM_005269
A_23_P34333(	FLJ31715	AK056277
A_32_P2362	DNAJB3	NM_001001394
A_23_P21570	NPAS3	NM_173159
A_23_P43425:	JPH1	NM_020647
A_33_P33308(	HUS1	NM_004507
A_23_P15843	BRIP1	NM_032043
A_32_P32097:	CRIPAK	NM_175918
A_32_P77989	NETO2	NM_018092
A_33_P32729:	ARID3A	NM_005224
A_23_P10292!	PWP2	NM_005049
A_23_P13111!	NFIC	NM_005597
A_23_P25481(	TCF15	NM_004609
A_24_P25698	MALAT1	AF001542
A_23_P92479	PPA2	NM_176869
A_32_P38686(	KIAA1467	NM_020853
A_23_P33179(	C3orf57	NM_001040100
A_33_P32990(	NR4A2	NM_006186
A_23_P3221	SQRDL	NM_021199
A_24_P57367	AHCY	NM_000687
A_23_P34200!	HFE	NM_000410
A_23_P12733	H2AFY2	NM_018649
A_23_P16584(	EMILIN1	NM_007046
A_32_P51742(	FAM120B	NM_032448
A_24_P78862	SLC43A2	NM_152346
A_24_P39248(	MYPOP	NM_001012643
A_23_P17105(	ELK1	NM_005229
A_23_P10959:	TBC1D22A	NM_014346
A_23_P20887(	AKT2	NM_001626
A_23_P62026	HCLS1	NM_005335
A_23_P78944	AMH	NM_000479
A_33_P33494:	ZBTB43	NM_014007
A_23_P93773	HOXA5	NM_019102
A_23_P42999(	FOSB	NM_006732
A_23_P43004(	SUV420H2	NM_032701
A_32_P73249	SND1	NM_014390

A_24_P16705	YME1L1	NM_139312
A_23_P11629	TMEM61	NM_182532
A_32_P98683	MLLT6	NM_005937
A_24_P13448	PIK3IP1	NM_052880
A_23_P14273	ZFYVE21	NM_024071
A_24_P31936	F11R	NM_016946
A_33_P33623	FHL2	NM_201555
A_23_P13776	PAFAH2	NM_000437
A_23_P43117	HIST1H4A	NM_003538
A_33_P32115	ERBB3	NM_001005915
A_23_P12401	IMPDH2	NM_000884
A_23_P25073	CBX7	NM_175709
A_23_P50692	RFX2	NM_000635
A_23_P34428	ZIK1	NM_001010879
A_23_P9443	C9orf78	NM_016520
A_23_P54471	ISL2	NM_145805
A_24_P36299	GRLF1	NM_004491
A_33_P32733	SH3BP4	NM_014521
A_24_P84419	VAV2	NM_003371
A_33_P33153	ARHGEF19	NM_153213
A_24_P56819	SMA4	BC035411
A_23_P90333	ZNF404	NM_001033719
A_23_P99404	ZMYM2	NM_003453
A_23_P74920	LCE2B	NM_014357
A_23_P15397	SGPP2	NM_152386
A_23_P25593	NKX3-2	NM_001189
A_24_P10831	NEDD4L	NM_015277
A_32_P10206	FOXO3	NM_001455
A_24_P29260	MGAT4B	NM_054013
A_24_P95029	TAX1BP1	NM_006024
A_23_P13261	OXTR	NM_000916
A_23_P33376	PCTK2	NM_002595
A_23_P67992	C1D	NM_006333
A_23_P14795	HGSNAT	NM_152419
A_23_P38784	SIGLEC7	NM_014385
A_23_P25931	RPS4Y1	NM_001008
A_32_P13627	NSUN7	NM_024677
A_32_P37609	SESN3	NM_144665
A_23_P10540	MAP3K12	NM_006301
A_32_P95729	FANCI	NM_018193
A_33_P32129	ZWINT	NM_032997
A_23_P50172	TSPAN32	NM_139022
A_23_P83159	KLHL9	NM_018847
A_32_P13581	RPS3A	NM_001006
A_23_P24424	DKFZp761E19	NM_138368
A_23_P10409	SYT2	NM_177402
A_23_P630	MSTO1	NM_018116
A_23_P20521	UTP14A	NM_006649
A_23_P47725	MAP6	NM_033063

A_23_P26294	TPSG1	NM_012467
A_23_P40468	LCE1A	NM_178348
A_23_P33969	LCE1E	NM_178353
A_23_P38586	CDCA2	NM_152562
A_23_P10701	APBB2	NM_173075
A_33_P33614	IFNAR2	NM_000874
A_32_P16571	SARNP	NM_033082
A_23_P21208	NFKBIZ	NM_031419
A_24_P14883	KLHDC8B	NM_173546
A_33_P33209	CTXN1	NM_206833
A_24_P29159	USP4	NM_003363
A_33_P32669	MYH7	NM_000257
A_23_P61831	BCL7A	NM_020993
A_23_P16549	GORASP2	NM_015530
A_24_P92504	CAV2	NM_001233
A_23_P72627	HGS	NM_004712
A_23_P16749	EFNA5	NM_001962
A_23_P20706	USP22	NM_015276
A_24_P25033	SNRPA	NM_004596
A_23_P6849	CCR1	NM_001295
A_24_P24137	MTA1	NM_004689
A_23_P1998	APBB1	NM_001164
A_33_P32963	DCAF16	NM_017741
A_23_P48530	INSM2	NM_032594
A_32_P30418	ZBTB22	NM_005453
A_23_P11422	RBBP7	NM_002893
A_32_P73386	MYO5B	NM_001080467
A_23_P10095	ZFP3	NM_153018
A_23_P21303	GSTCD	NM_024751
A_24_P89323	COX7B	ENST00000481445
A_23_P86054	WDTC1	NM_015023
A_23_P37552	LCE1D	NM_178352
A_23_P21785	MACF1	NM_033044
A_24_P25884	NFATC1	NM_172390
A_23_P21376	ANKH	NM_054027
A_23_P36561	NOTCH4	NM_004557
A_23_P14381	MYLK	NM_053025
A_23_P31215	EDN2	NM_001956
A_33_P32151	C1orf69	NM_001010867
A_23_P88703	TP53BP1	NM_005657
A_23_P21034	COX7A2L	NM_004718
A_23_P21342	ENC1	NM_003633
A_23_P98891	CDK2	NM_001798
A_32_P23389	CYP51A1	NM_000786
A_23_P81811	TJAP1	NM_080604
A_23_P21793	SPHAR	NM_006542
A_23_P114	EXTL2	NM_001439
A_33_P32923	KIF5B	NM_004521
A_33_P32993	ZNF550	NM_001039654

A_32_P36249	RHBDL2	NM_017821
A_23_P10247	MSH2	NM_000251
A_23_P50318	ABR	NM_021962
A_32_P39996	CTPS2	NM_175859
A_33_P32783	MSRB2	NM_012228
A_23_P12380	MURC	NM_001018116
A_24_P80915	BCLAF1	NM_014739
A_24_P40013	KRTAP7-1	NM_181606
A_23_P31520	CCBL1	NM_004059
A_23_P14684	APBA2	NM_005503
A_23_P30115	IL2	NM_000586
A_23_P36877	ZNF114	NM_153608
A_23_P32053	ZNF780A	NM_001010880
A_23_P15859	COL5A1	NM_000093
A_23_P12041	YWHAB	NM_003404
A_23_P12659	S100A11	NM_005620
A_33_P33785	AS3MT	NM_020682
A_32_P76749	NBAS	NM_015909
A_23_P21770	GYG2	NM_003918
A_23_P15132	RPL6	NM_001024662
A_23_P13167	CXCR7	NM_020311
A_23_P39814	CIR1	NM_004882
A_23_P20888	UHRF1	NM_013282
A_33_P33127	PRSS33	NM_152891
A_24_P75230	MYL12B	NM_033546
A_33_P32942	UTF1	NM_003577
A_24_P34866	ATRX	NM_000489
A_24_P19226	RALA	NM_005402
A_33_P32114	LRRC8B	NM_015350
A_24_P21223	SLC6A18	NM_182632
A_23_P10836	OTX1	NM_014562
A_32_P21136	AGAP8	NM_001077686
A_23_P90710	DES	NM_001927
A_32_P61339	PHIP	BC036479
A_23_P10288	SYNJ1	NM_203446
A_33_P32517	CMBL	NM_138809
A_24_P68094	KIF18B	BC048263
A_24_P31453	KRTAP13-2	NM_181621
A_23_P85593	CABC1	NM_020247
A_32_P32116	FAM123A	ENST00000357816
A_32_P53479	KISS1R	NM_032551
A_23_P90320	ZNF221	NM_013359
A_32_P64860	SPRY1	NM_199327
A_23_P20494	GJB2	NM_004004
A_23_P21134	DDIT3	NM_004083
A_32_P18350	PRKDC	NM_006904
A_24_P91415	USP54	NM_152586
A_23_P41286	CTBP1	NM_001012614
A_33_P32636	KRTAP6-2	NM_181604

A_32_P22456	LIN28B	NM_001004317
A_33_P33039	FAM53B	AK127323
A_23_P30331	ZXDC	NM_025112
A_32_P15267	PABPC1	NM_002568
A_23_P39362	KLF14	NM_138693
A_33_P32903	CYP1B1	NM_000104
A_23_P14676	RNF139	NM_007218
A_23_P16054	FAM63A	NM_001040217
A_23_P16607	ZNF506	ENST00000443905
A_23_P25886	PDCD4	NM_145341
A_23_P72068	GMDS	NM_001500
A_23_P74711	DEDD	NM_032998
A_23_P20805	LPIN2	NM_014646
A_23_P39931	DYSF	NM_003494
A_23_P13714	DKC1	NM_001363
A_33_P32695	PBRM1	NM_018313
A_32_P17793	GABPB2	ENST00000368918
A_32_P10887	PMP2	NM_002677
A_32_P13406	FKBP3	NM_002013
A_24_P50091	C15orf51	NR_003260
A_23_P97860	LIPA	NM_000235
A_24_P24617	MYO9B	NM_004145
A_23_P21613	WHSC1L1	NM_017778
A_24_P75725	GPR157	ENST00000377411
A_23_P15571	NEIL3	NM_018248
A_23_P18656	SCLT1	NM_144643
A_23_P70785	AIM1	NM_001624
A_33_P32213	CENPI	NM_006733
A_23_P51414	SMYD3	NM_022743
A_32_P10850	BTF3	NM_001037637
A_24_P22092	CAMTA1	NM_015215
A_32_P17109	SFRS12IP1	NM_173829
A_23_P21300	WDR1	NM_017491
A_23_P14250	GADD45B	NM_015675
A_23_P96072	GRIN1	NM_007327
A_24_P31335	NR2F2	NM_021005
A_32_P80901	RPS15A	ENST00000322989
A_23_P10129	EML2	NM_012155
A_23_P66137	SOX8	NM_014587
A_33_P32855	CLDN3	NM_001306
A_24_P30472	PPIB	NM_000942
A_24_P27081	CRK	NM_016823
A_32_P17040	TLN2	NM_015059
A_24_P37985	ACAA1	NM_001607
A_23_P10191	MYO9B	NM_004145
A_32_P48223	CDCA2	NM_152562
A_23_P52597	C1QTNF4	NM_031909
A_24_P24524	PIP4K2B	NM_003559
A_33_P33278	ZNF436	NM_001077195

A_24_P96780	CENPF	NM_016343
A_23_P15123	UBFD1	NM_019116
A_33_P33981(	SYS1	NM_033542
A_23_P6129	PAX1	NM_006192
A_23_P15566(	PPP2R2C	NM_020416
A_24_P30155:	LPIN2	NM_014646
A_33_P33194(	NCOA4	NM_001145261
A_23_P15091(	STX2	NM_001980
A_23_P35710:	APOBEC3F	NM_145298
A_23_P20636!	TMEM208	NM_014187
A_23_P15150(	PLEK2	NM_016445
A_23_P37869(	TMEM64	NM_001008495
A_24_P12205(	PSMF1	NM_006814
A_23_P15227:	RNPS1	NM_006711
A_23_P21744(	LAMP2	NM_002294
A_23_P26807	TP53	NM_000546
A_23_P21338!	BASP1	NM_006317
A_23_P3164	GMPR2	NM_001002000
A_33_P33935(	S100A7A	ENST00000329256
A_23_P20514:	KLF12	NM_007249
A_23_P12643	AS3MT	NM_020682
A_23_P17814	PLA2G3	NM_015715
A_23_P69573	GUCY1A3	NM_000856
A_23_P71774	IFNB1	NM_002176
A_23_P61702	TSPYL2	NM_022117
A_23_P37795:	KCTD12	NM_138444
A_24_P94263(	KDM6B	NM_001080424
A_33_P33762-	S100A2	NM_005978
A_23_P42558:	MED11	NM_001001683
A_32_P19477!	ZBTB34	NM_001099270
A_23_P74034	CACYBP	NM_014412
A_32_P32123(	LOC202181	NR_026921
A_23_P68752	DIP2A	NM_015151
A_23_P20269:	KBTBD4	NM_016506
A_23_P41685(	GPIHBP1	NM_178172
A_23_P9435	SLC25A25	NM_001006641
A_23_P35854!	SLC43A3	NM_199329
A_24_P41625:	GGA2	NM_015044
A_24_P30717!	SMYD4	NM_052928
A_23_P38532:	STAMBPL1	NM_020799
A_32_P42549!	SEL1L3	NM_015187
A_24_P8049	CNOT2	NM_014515
A_23_P15135	ZSCAN10	NM_032805
A_23_P83818	COL5A1	NM_000093
A_23_P56146	RAX2	NM_032753
A_23_P15069:	FJX1	NM_014344
A_32_P12785!	NANOS3	NM_001098622
A_23_P15414	SCARF1	NM_145352
A_24_P34595!	SPATA18	NM_145263

A_23_P21126	RIPK4	NM_020639
A_32_P14220	PGK1	ENST00000373316
A_23_P11103	BMP15	NM_005448
A_23_P35235	KIAA2013	NM_138346
A_23_P77151	LASS3	NM_178842
A_23_P6479	JOSD1	NM_014876
A_23_P11741	FOXG1	NM_005249
A_24_P25170	SEC62	NM_003262
A_33_P33056	TIPARP	ENST00000461166
A_32_P52461	C17orf74	NM_175734
A_33_P32939	SH2D3C	NM_170600
A_24_P34189	ACVR1B	NM_004302
A_23_P14308	MPV17	NM_002437
A_23_P59022	TRERF1	NM_033502
A_23_P72117	SMPDL3A	NM_006714
A_24_P14657	SHB	NM_003028
A_23_P78302	NFE2L1	NM_003204
A_24_P84498	PIGR	NM_002644
A_23_P33137	NEDD4	NM_198400
A_23_P32087	FAM119B	NM_206914
A_23_P35024	GLIS3	NM_001042413
A_23_P75111	CYP17A1	NM_000102
A_24_P41180	RPS5	NM_001009
A_23_P33837	NR2E1	NM_003269
A_24_P93740	PRSS23	NM_007173
A_32_P23019	AGAP7	NM_001077685
A_23_P41768	ZNF471	AB037817
A_23_P36541	ZNF783	NM_001195220
A_23_P89455	SLC35B1	NM_005827
A_23_P20473	BRF2	NM_018310
A_32_P88958	INADL	NM_176877
A_23_P80129	RRP1	NM_003683
A_23_P8558	ABHD11	NM_148912
A_23_P10728	HOXB2	NM_002145
A_24_P13506	NBPF11	NM_183372
A_23_P10052	PPP1R3B	NM_024607
A_23_P15498	GGT1	NM_005265
A_23_P14321	ACOT8	NM_005469
A_32_P17518	ZBTB44	NM_014155
A_23_P95080	CTNND1	NM_001085461
A_23_P43131	YIPF6	NM_173834
A_23_P55099	PRKCA	NM_002737
A_23_P16123	SFXN3	NM_030971
A_23_P42144	PEX6	NM_000287
A_23_P34033	LCE3E	NM_178435
A_24_P29308	ZNF579	NM_152600
A_33_P32775	ZFYVE28	NM_001172657
A_33_P32366	MAP2K7	NM_145185
A_33_P32682	KRT39	NM_213656

A_23_P92008	XPC	NM_004628
A_23_P34291	KLHL15	NM_030624
A_23_P6223	SFRS15	NM_020706
A_23_P11067	AMACR	NM_014324
A_23_P92744	F2RL1	NM_005242
A_23_P33303	PIK3CD	NM_005026
A_23_P16035	AKT3	NM_181690
A_23_P38650	GATAD2B	NM_020699
A_33_P33583	BCL11B	NM_138576
A_24_P28480	INPP5J	NM_001002837
A_23_P10077	UBTF	NM_001076683
A_24_P16803	FOXJ1	NM_001454
A_33_P34062	GDF7	NM_182828
A_24_P30757	ANKRD13A	NM_033121
A_23_P36423	MGA	AL713737
A_33_P33687	FLJ35776	NR_024101
A_23_P78628	MAP2K7	NM_145185
A_23_P40339	DKFZP586I14	NR_002186
A_32_P52669	MKL2	AK126948
A_23_P16463	ZNF419	NM_024691
A_23_P47941	HOXC11	NM_014212
A_23_P18384	ARMC8	NM_213654
A_32_P31545	HSPA12A	NM_025015
A_23_P15415	MFSD9	NM_032718
A_33_P35029	ZDHHC8P	NR_003950
A_33_P34138	TIMM13	NM_012458
A_23_P21865	ZGPAT	NM_032527
A_23_P11331	P4HTM	NM_177938
A_24_P32823	CPSF3L	NM_017871
A_33_P32409	C5orf43	NM_001048249
A_23_P10079	STAT3	NM_213662
A_24_P52597	CYP17A1	NM_000102
A_24_P38200	POLR3K	NM_016310
A_23_P20434	RFX4	NM_213594
A_24_P33445	ADAMTS6	NM_197941
A_33_P33187	FSTL3	NM_005860
A_23_P12097	FAM118A	NM_017911
A_23_P14364	ASCC2	NM_032204
A_23_P11685	PLA2G4A	NM_024420
A_23_P22129	HAP1	NM_177977
A_23_P65757	CCNB2	NM_004701
A_23_P20942	TRAK2	NM_015049
A_33_P32651	DSG1	NM_001942
A_23_P16781	RBM24	NM_153020
A_24_P27479	CDCA7L	NM_018719
A_23_P32021	FANCC	NM_000136
A_23_P15325	ZNF773	NM_198542
A_23_P37375	RPS6KA5	NM_004755
A_23_P50079	CASP6	NM_001226

A_23_P38397	SNAPC5	NM_006049
A_23_P12086	NIPSNAP1	NM_003634
A_23_P53897	SOX1	NM_005986
A_32_P11499	UBE2N	NM_003348
A_33_P32486	ASXL2	NM_018263
A_23_P31765	PKIA	NM_006823
A_32_P41460	ACER2	NM_001010887
A_23_P11786	NDNL2	NM_138704
A_23_P12088	HMOX1	NM_002133
A_24_P36436	TNFRSF1A	NM_001065
A_23_P14654	MOBKL2B	NM_024761
A_23_P37836	PCDH7	NM_032457
A_23_P43566	NDUFA8	NM_014222
A_33_P32765	ATP11C	NM_001010986
A_32_P14207	POU2F1	NM_001198783
A_24_P37739	TRIM26	NM_003449
A_24_P95070	TFPI2	ENST00000222543
A_23_P49167	AGRP	NM_001138
A_24_P16228	CEP250	NM_007186
A_23_P21722	AP3M2	NM_006803
A_24_P7965	ESRRG	NM_206594
A_33_P32169	C10orf104	NM_173473
A_23_P53736	FBXO21	NM_033624
A_32_P41407	STS	NM_000351
A_23_P46964	HIF1AN	NM_017902
A_23_P31347	FAM120B	NM_032448
A_23_P20672	MT1E	NM_175617
A_23_P8834	EPHX2	NM_001979
A_23_P31593	ABHD11	NM_148912
A_23_P20904	FXYD3	NM_005971
A_24_P14249	KRTAP1-3	NM_030966
A_24_P35190	STEAP4	NM_024636
A_23_P16058	HYI	NM_031207
A_23_P42714	SH2B1	NM_015503
A_32_P13852	GCSH	NM_004483
A_33_P33785	USP38	NM_032557
A_23_P33726	APCDD1	NM_153000
A_24_P15129	MLLT1	NM_005934
A_23_P14804	PTGER4	NM_000958
A_23_P10434	PIP4K2A	NM_005028
A_23_P41645	ELL2	NM_012081
A_23_P41221	RAP1GAP2	NM_015085
A_32_P41515	WDR27	NM_182552
A_23_P20598	IGF1R	NM_000875
A_32_P13835	MSRA	NM_012331
A_23_P37658	CLYBL	NM_206808
A_24_P94032	LAT2	ENST00000398475
A_23_P48570	DHRS2	NM_182908
A_23_P11884	KRTAP1-5	NM_031957

A_23_P21338	BASP1	NM_006317
A_32_P20438	CIAPIN1	NM_020313
A_33_P33267	FAM188B	NM_032222
A_33_P33711	ZNF365	NM_199451
A_32_P2643	HEATR5A	NM_015473
A_24_P75676	CDRT15	NM_001007530
A_23_P71410	WDYHV1	NM_018024
A_23_P16505	KDM4B	NM_015015
A_23_P11952	AP3D1	NM_003938
A_32_P35727	KDM5B	NM_006618
A_24_P15218	PRICKLE2	NM_198859
A_23_P12477	SCMH1	NM_012236
A_23_P13739	ENO1	NM_001428
A_32_P11594	GTPBP4	ENST00000360803
A_23_P42132	FAM129A	NM_052966
A_32_P18472	KPNB1	NM_002265
A_32_P56392	RBMX	NM_002139
A_23_P21733	PRKX	NM_005044
A_32_P75026	CAP1	NM_006367
A_33_P32171	RBM9	NM_001031695
A_33_P33486	KTN1	NM_004986
A_23_P43090	MORN4	NM_178832
A_24_P1949	NPTX1	NM_002522
A_23_P13561	DALRD3	NM_018114
A_33_P32270	BID	NM_197966
A_24_P11100	SAFB2	NM_014649
A_24_P82155	ANKDD1A	NM_182703
A_32_P16330	HHIPL1	NM_001127258
A_23_P18123	NLGN1	NM_014932
A_23_P29537	GRIP2	NM_001080423
A_23_P6130	PAX1	NM_006192
A_24_P17450	AMT	NM_000481
A_24_P36501	HOXB13	NM_006361
A_24_P22518	PCNXL2	NM_014801
A_23_P10310	MFNG	NM_002405
A_24_P27681	RCOR3	NM_018254
A_32_P42247	TBC1D9	NM_015130
A_33_P32720	OPRD1	NM_000911
A_23_P13079	IGFL1	NM_198541
A_23_P21465	PBX2	NM_002586
A_23_P12730	CCDC3	NM_031455
A_24_P58889	SLCO3A1	NM_013272
A_23_P93360	AGER	NM_001136
A_23_P38283	P2RY1	NM_002563
A_23_P30924	ZNF498	NM_145115
A_24_P11066	CDC42SE1	NM_001038707
A_23_P40348	NLRP10	NM_176821
A_23_P13559	ING5	NM_032329
A_24_P14318	TMSL3	NM_183049

A_23_P14293[illegible]	REV1	NM_016316
A_23_P77440	NFATC3	NM_173165
A_23_P32210[illegible]	PTPN18	NM_014369
A_33_P33859[illegible]	ZNF20	NM_021143
A_23_P13681[illegible]	KNTC1	NM_014708
A_23_P33592[illegible]	RPS6KA2	NM_021135
A_33_P35392[illegible]	C9orf100	NM_032818
A_23_P37910	MAPK3	NM_002746
A_23_P15892[illegible]	GPR125	NM_145290
A_23_P20153[illegible]	JUN	NM_002228
A_32_P33233[illegible]	FOXN4	NM_213596
A_23_P15566[illegible]	NAAA	NM_001042402
A_23_P12319[illegible]	ACTR3B	NM_020445
A_24_P36781[illegible]	LMF1	BC028099
A_24_P23814[illegible]	LRRC37A2	NM_001006607
A_23_P16122	CYP2A6	NM_000762
A_23_P11869[illegible]	FOXN1	NM_003593
A_23_P59018	TRERF1	NM_033502
A_33_P33278[illegible]	SGPL1	NM_003901
A_23_P10937[illegible]	PEX26	NM_017929
A_24_P31693[illegible]	LRRFIP1	NM_001137550
A_24_P19360[illegible]	ZNF174	NM_003450
A_32_P87613[illegible]	SKIL	Z19588
A_23_P14030[illegible]	MAPK1IP1L	NM_144578
A_23_P79807	VSTM2L	NM_080607
A_32_P86837	FAM35A	NM_019054
A_23_P48728	C14orf179	NM_052873
A_33_P33463[illegible]	UBAP2	NM_018449
A_32_P22241[illegible]	PARP15	NM_152615
A_33_P32372[illegible]	FBXO44	NM_001014765
A_24_P23017[illegible]	SPNS2	NM_001124758
A_23_P25916[illegible]	TCEAL4	NM_024863
A_23_P38750[illegible]	FOXC2	NM_005251
A_33_P32406[illegible]	BRD3	NM_007371
A_23_P37915[illegible]	BRWD3	NM_153252
A_32_P22443[illegible]	SAP130	NM_024545
A_32_P47365[illegible]	KIAA1522	NM_020888
A_23_P43547[illegible]	TMPRSS13	NM_001077263
A_23_P11069[illegible]	ZNF300	NM_052860
A_23_P23966	ZNF488	NM_153034
A_23_P71316	RBPMS	NM_001008712
A_23_P11904[illegible]	GREB1L	NM_001142966
A_23_P7154	HAND2	NM_021973
A_23_P25048[illegible]	TRIM62	NM_018207
A_33_P32212[illegible]	IPP	NM_001145349
A_23_P33527[illegible]	PART1	NR_028508
A_23_P31218[illegible]	TCEB2	NM_207013
A_33_P34175[illegible]	BRD8	NM_139199
A_33_P33232[illegible]	JUN	NM_002228

A_23_P3911	PLXDC1	NM_020405
A_23_P42284[illegible]	FERD3L	NM_152898
A_24_P94432[illegible]	ZNF517	NM_213605
A_32_P18297[illegible]	E2F7	NM_203394
A_33_P32227[illegible]	ZNF117	NM_015852
A_32_P10093[illegible]	ZNF667	NM_022103
A_33_P33028[illegible]	KLHL31	NM_001003760
A_23_P17003[illegible]	MID1	NM_033290
A_32_P36030[illegible]	RTTN	NM_173630
A_23_P66948	FAM59A	NM_022751
A_24_P8088	RIOK1	NM_153005
A_23_P25465[illegible]	CLIC3	NM_004669
A_33_P32273[illegible]	MIER3	NM_152622
A_23_P51410	SMYD3	NM_022743
A_23_P58321	CCNA2	NM_001237
A_23_P16058	ZNF296	NM_145288
A_23_P44195	MSI2	NM_138962
A_23_P38992[illegible]	HLA-DMB	NM_002118
A_23_P48339	IFT88	NM_175605
A_32_P95914	C6orf167	NM_198468
A_24_P84540	MLLT10	NM_004641
A_24_P22415[illegible]	RFX4	NM_213594
A_23_P20721[illegible]	ALDH3A1	NM_000691
A_24_P52921	BCAT1	NM_005504
A_23_P37914[illegible]	ZNF626	NM_145297
A_23_P59791	LUC7L2	NM_016019
A_23_P53162	STIM1	NM_003156
A_24_P15421[illegible]	IRF2BP2	NM_182972
A_23_P15543[illegible]	DUSP7	NM_001947
A_23_P39311[illegible]	SLC25A37	NM_016612
A_33_P33614[illegible]	SLC22A15	NM_018420
A_23_P16035[illegible]	EPB41	NM_004437
A_24_P45767	AGBL5	NM_021831
A_24_P50089[illegible]	AK2	NM_013411
A_32_P14707[illegible]	SLC8A1	NM_021097
A_23_P32876[illegible]	ZNF519	NM_145287
A_23_P20494	NDRG1	NM_006096
A_23_P15709[illegible]	ANLN	NM_018685
A_23_P42412[illegible]	ZNF354C	NM_014594
A_33_P33495[illegible]	CHEK1	NM_001114121
A_23_P15972[illegible]	GPR50	NM_004224
A_23_P32368[illegible]	HIST1H4H	NM_003543
A_23_P15040[illegible]	CREB3L1	NM_052854
A_23_P22433	RP2	NM_006915
A_23_P12896[illegible]	ALDH6A1	NM_005589
A_23_P20844[illegible]	SAFB	NM_002967
A_23_P983	PRDX6	NM_004905
A_23_P15618[illegible]	SHROOM1	NM_133456
A_23_P14515[illegible]	PDCD2	NM_002598

A_23_P14146[illegible]	CDK5R1	NM_003885
A_32_P22820[illegible]	MID1IP1	NM_021242
A_32_P42197	HNRNPA1	NM_031157
A_23_P43594[illegible]	ZNF530	AB040941
A_24_P58337	FTH1	NM_002032
A_23_P25403[illegible]	TTF1	NM_007344
A_32_P53581[illegible]	RPS6KA4	NM_003942
A_23_P24017	BTAF1	NM_003972
A_33_P32642[illegible]	MOV10	NM_020963
A_32_P94444	PRSS2	NM_002770
A_23_P82324	CARD11	NM_032415
A_24_P31339[illegible]	NUTF2	ENST00000219169
A_33_P33338[illegible]	ZNF337	NM_015655
A_23_P11204[illegible]	ZNF703	NM_025069
A_23_P14398[illegible]	ATG7	NM_006395
A_23_P37128[illegible]	LCE2D	NM_178430
A_23_P25554[illegible]	PTPRM	NM_002845
A_23_P21572[illegible]	CFTR	NM_000492
A_23_P16365[illegible]	MPG	NM_002434
A_24_P67585	VGLL3	NM_016206
A_23_P21606[illegible]	ATAD2	NM_014109
A_32_P14070[illegible]	PMS2	NM_000535
A_24_P56052	ZFP91	NM_053023
A_23_P90419	PBX4	NM_025245
A_23_P58788	CDX1	NM_001804
A_23_P41424	SLC39A8	NM_022154
A_23_P230	TTC4	NM_004623
A_23_P20032[illegible]	RABGAP1L	NM_014857
A_23_P78532	POU2F2	NM_002698
A_33_P32197[illegible]	ANKRD57	NM_023016
A_23_P43684	BNC2	NM_017637
A_23_P99582	PNN	NM_002687
A_32_P44856[illegible]	IL29	NM_172140
A_23_P34152[illegible]	DCST1	NM_152494
A_23_P20293[illegible]	APLP2	NM_001642
A_23_P74012	SPRR1A	NM_005987
A_23_P96149	KRT17	NM_000422
A_32_P10329[illegible]	SMYD3	NM_022743
A_33_P33793[illegible]	WDR78	NM_207014
A_23_P59107	MRPS18B	NM_014046
A_23_P33759	DHRS3	NM_004753
A_32_P35617[illegible]	IQSEC3	NM_015232
A_24_P22732[illegible]	RCOR2	NM_173587
A_23_P62002	CSF1	NM_172210
A_23_P25085[illegible]	SEMA3A	NM_006080
A_23_P81805	VEGFA	NM_001025366
A_23_P10265[illegible]	B4GALT5	NM_004776
A_33_P32322[illegible]	CCNG1	NM_004060
A_23_P40895[illegible]	BPIL2	NM_174932

A_32_P18324	LRIG2	NM_014813
A_23_P31945	IL33	NM_033439
A_23_P12511	KLHDC8B	NM_173546
A_23_P43613	NDUFB6	NM_182739
A_23_P21709	VPS13A	NM_033305
A_24_P10767	MAPK1IP1L	NM_144578
A_32_P40288	TMEM200A	NM_052913
A_33_P32813	SNTB2	NM_006750
A_24_P80214	IRS1	NM_005544
A_24_P17648	ZCCHC14	NM_015144
A_23_P10983	SRGAP3	NM_014850
A_23_P25130	SEC16A	NM_014866
A_23_P16216	KCTD14	NM_023930
A_33_P32460	APOA1BP	NM_144772
A_24_P48617	UQCR	NM_006830
A_33_P34031	NR2F6	NM_005234
A_23_P97161	TTF2	NM_003594
A_23_P41292	CTBP1	NM_001012614
A_33_P32875	MSH2	NM_000251
A_23_P27147	ANAPC11	NM_001002244
A_23_P25580	ZNF7	NM_003416
A_23_P92132	IFRD2	NM_006764
A_24_P19419	JPH2	NM_020433
A_32_P53204	CCNE2	NM_057749
A_23_P83469	GMDS	NM_001500
A_23_P82327	CARD11	NM_032415
A_33_P35892	SLC25A6	NM_001636
A_24_P27072	NUPR1	NM_001042483
A_33_P33519	C2orf64	NM_001008215
A_23_P35256	POLR3C	NM_006468
A_23_P20295	PDGFD	NM_025208
A_24_P37545	SSBP3	NM_001009955
A_23_P224	TTC4	NM_004623
A_23_P11908	ZNF551	BC005868
A_33_P33054	REM2	NM_173527
A_23_P30453	STK10	NM_005990
A_23_P39691	C11orf63	NM_199124
A_23_P25575	PFKFB3	NM_004566
A_23_P21017	ITGA6	NM_000210
A_23_P38867	LTA4H	NM_000895
A_23_P34752	TFCP2	NM_005653
A_23_P12689	PEX11B	NM_003846
A_23_P15464	BMP7	NM_001719
A_23_P70701	ARID1B	NM_017519
A_33_P33589	CLOCK	NM_004898
A_24_P23723	SIK1	NM_173354
A_23_P35854	SLC43A3	NM_199329
A_23_P32751	STARD4	NM_139164
A_23_P21908	ZNF3	NM_032924

A_23_P46412	SCNN1D	NM_001130413
A_23_P54573	ZFPM1	NM_153813
A_23_P24884	ST5	NM_005418
A_24_P17071[illegible]	SSC5D	AK092577
A_24_P23509[illegible]	FOXQ1	NM_033260
A_23_P25525	GTF3A	NM_002097
A_24_P36868	WDR26	NM_025160
A_23_P10581[illegible]	KDELC2	NM_153705
A_23_P20801[illegible]	ZNF407	NM_017757
A_33_P34200[illegible]	ZNF445	NM_181489
A_23_P3792	SLC7A5	NM_003486
A_33_P32516[illegible]	ABCA2	NM_001606
A_32_P68919	RNPS1	NM_006711
A_24_P57898	BHLHE23	NM_080606
A_24_P21255[illegible]	MLL2	NM_003482
A_33_P32841[illegible]	POMGNT1	NM_017739
A_24_P19753[illegible]	PDE8B	NM_003719
A_33_P33663[illegible]	KRTAP27-1	NM_001077711
A_24_P21484[illegible]	POU5F1	NM_002701
A_33_P33494[illegible]	ATAD3A	NM_018188
A_24_P62138[illegible]	RASA4P	AK025420
A_33_P33141[illegible]	PACSIN2	NM_007229
A_32_P36332[illegible]	SEL1L3	NM_015187
A_33_P32095[illegible]	AQP3	NM_004925
A_23_P15682[illegible]	HTR1B	NM_000863
A_24_P11372[illegible]	SOX3	NM_005634
A_23_P10663[illegible]	DDX28	NM_018380
A_23_P21048[illegible]	ADA	NM_000022
A_23_P16450[illegible]	DSG1	NM_001942
A_24_P18068[illegible]	LAPTM4B	NM_018407
A_23_P25030	HSD17B6	NM_003725
A_33_P32700[illegible]	RBMXL2	NM_014469
A_24_P4661	EI24	NM_004879
A_23_P20364[illegible]	CREBZF	NM_001039618
A_23_P41960[illegible]	SREBF2	NM_004599
A_33_P32482[illegible]	UBXN8	NM_005671
A_23_P52647	EHD1	NM_006795
A_23_P15259[illegible]	SLC9A3R1	NM_004252
A_24_P12749[illegible]	HHIPL1	NM_001127258
A_23_P31041	MYLIP	NM_013262
A_24_P38364[illegible]	AMZ1	NM_133463
A_23_P20481[illegible]	OSBPL8	NM_020841
A_24_P10550[illegible]	ENO1	NM_001428
A_23_P34766	YBX1	NM_004559
A_23_P15423[illegible]	NMI	NM_004688
A_24_P38062[illegible]	FAM115A	NM_014719
A_23_P14165[illegible]	NCOR1	NM_006311
A_32_P16088[illegible]	NEDD4	NM_198400
A_33_P32898[illegible]	IGFL1	NM_198541

A_32_P39505[illegible]	CARM1	NM_199141
A_23_P39193[illegible]	ADAMTSL4	AK023606
A_33_P35679[illegible]	FLJ11292	AK023417
A_23_P41775[illegible]	OPHN1	NM_002547
A_32_P18349[illegible]	METT5D1	NM_152636
A_33_P32272[illegible]	FLJ45983	NR_024256
A_32_P12989[illegible]	MEGF9	NM_001080497
A_23_P48561	EFS	NM_005864
A_23_P8241	FBXO5	NM_012177
A_24_P51118	MTAP	NM_002451
A_23_P15339[illegible]	CLEC4G	NM_198492
A_23_P22473	FTSJ1	NM_012280
A_24_P88921	XPC	NM_004628
A_23_P17033[illegible]	AP3M2	NM_006803
A_23_P41896[illegible]	CRX	NM_000554
A_23_P7101	SLBP	NM_006527
A_23_P11282[illegible]	LCMT1	NM_016309
A_23_P43193[illegible]	MR1	NM_001531
A_23_P52286	RP11-529I10.[illegible]	NM_015448
A_32_P30263[illegible]	EHD1	ENST00000320631
A_33_P32530[illegible]	MED23	NM_015979
A_33_P33543[illegible]	POM121L10P	NR_024593
A_23_P6335	SERPIND1	NM_000185
A_23_P35287[illegible]	PVRL2	NM_002856
A_23_P11103[illegible]	HIST1H3A	NM_003529
A_33_P34195[illegible]	CDADC1	NM_001193478
A_23_P20431[illegible]	LMO3	NM_018640
A_23_P20038[illegible]	KHDRBS1	NM_006559
A_23_P16486[illegible]	KLK4	NM_004917
A_23_P27107	TM4SF5	NM_003963
A_23_P21771[illegible]	ARSD	NM_009589
A_23_P30956	KIAA0776	NM_015323
A_23_P13270[illegible]	TDGF1	NM_003212
A_23_P37345	SNW1	NM_012245
A_24_P24229[illegible]	ZRANB2	NM_005455
A_23_P56654	MCEE	NM_032601
A_23_P11377[illegible]	ITGBL1	NM_004791
A_23_P12922[illegible]	FAH	NM_000137
A_23_P73604	TRMT2B	NM_024917
A_23_P10714	C17orf90	AY007126
A_32_P72822	CCNB2	AK023404
A_24_P36402[illegible]	UBE2D1	NM_003338
A_24_P35770[illegible]	VPS8	NM_001009921
A_32_P18376[illegible]	ERBB4	NM_005235
A_24_P11110[illegible]	FGF1	NM_000800
A_23_P16895[illegible]	ZHX2	NM_014943
A_32_P23414[illegible]	SHC4	NM_203349
A_23_P17345	MAFB	NM_005461
A_23_P12998[illegible]	EZH1	NM_001991

A_23_P66095	MC1R	NM_002386
A_23_P66038	ZDHHC1	NM_013304
A_23_P33463	JRK	NM_003724
A_23_P13999	UBAC2	NM_177967
A_24_P17105	TMEM64	NM_001008495
A_23_P90280	PIAS4	NM_015897
A_32_P10123	UHRF1	NM_013282
A_23_P49310	ERN2	NM_033266
A_23_P20890	SEMA6B	NM_032108
A_32_P50843	ATP1A3	NM_152296
A_23_P93988	ARHGEF5	NM_005435
A_23_P12287	TAF6	NM_005641
A_23_P11521	VPS72	NM_005997
A_24_P28913	SH3KBP1	NM_001024666
A_23_P10150	KLK11	NM_144947
A_23_P12018	NDUFA10	NM_004544
A_24_P18534	MBD1	NM_015845
A_33_P32409	DPF3	NM_012074
A_24_P10298	DNAJB2	NM_006736
A_24_P25872	DEPDC1	NM_017779
A_24_P38190	CX3CL1	NM_002996
A_32_P90623	ATP5A1	NM_001001937
A_23_P10870	DOLK	NM_014908
A_24_P93624	RAB3IP	NM_175623
A_33_P33301	DIABLO	NM_138929
A_23_P62807	SCCPDH	NM_016002
A_24_P17613	WDFY2	NM_052950
A_33_P33425	PITPNM3	NM_031220
A_24_P17931	PTPN23	NM_015466
A_24_P14881	RUVBL1	NM_003707
A_23_P33670	DTX3	NM_178502
A_33_P32839	ZNF671	NM_024833
A_23_P21174	GTF2E1	NM_005513
A_24_P41516	URM1	D83200
A_24_P93715	KLF8	NM_001159296
A_23_P11120	FKBP5	NM_004117
A_23_P10720	STAT3	NM_213662
A_23_P10608	RNASE7	NM_032572
A_23_P70719	LAMA2	NM_000426
A_23_P11506	CRABP2	NM_001878
A_23_P30855	DLX3	NM_005220
A_23_P41022	C6orf52	NM_001145020
A_33_P32321	PSPC1	ENST00000338910
A_23_P40173	CRYL1	NM_015974
A_23_P13064	GBP6	NM_198460
A_33_P32536	KCNH3	NM_012284
A_23_P34498	ICK	NM_016513
A_23_P19302	RNASET2	NM_003730
A_23_P37685	TMEM204	ENST00000253934

A_23_P117225	ERCC5	NM_000123
A_32_P149492	NBPF11	NM_183372
A_24_P280846	GOLT1A	NM_198447
A_23_P146456	CTSL2	NM_001333
A_23_P41255	G3BP2	NM_203505
A_32_P12104	ANAPC1	NM_022662
A_24_P81900	SLC2A3	NM_006931
A_23_P310540	LCE4A	NM_178356
A_23_P83328	ENG	NM_000118
A_23_P211119	SIM2	NM_005069
A_23_P8253	RAET1E	NM_139165
A_23_P98805	C11orf67	AF092136
A_24_P294931	PPP2R5D	NM_180976
A_24_P104512	EVPL	NM_001988
A_23_P308519	SLC9A3R1	BX648303
A_23_P90689	TRIB2	NM_021643
A_23_P135139	KLF9	NM_001206
A_32_P34611	ZFP82	NM_133466
A_33_P337756	PRICKLE3	NM_006150
A_23_P166823	TNNC1	NM_003280
A_23_P104617	GYLTL1B	NM_152312
A_24_P93656	CAND1	AK027783
A_23_P58102	EXOSC7	NM_015004
A_24_P353964	HADHA	NM_000182
A_23_P127374	IRF7	NM_004031
A_32_P183579	TPRXL	NR_002223
A_23_P107661	PPP2R1A	NM_014225
A_24_P212677	RNF170	NM_001160224
A_23_P13150	C11orf71	CR597454
A_32_P175584	RPS15A	NM_001019
A_23_P21777	SNAPC5	ENST00000316634
A_23_P4808	PTGER1	NM_000955
A_24_P307714	GRINL1A	AK074767
A_24_P263878	ABL2	NM_007314
A_24_P203328	TPRXL	NR_002223
A_23_P347468	FZD3	NM_017412
A_24_P941759	G2E3	NM_017769
A_23_P82941	ARFGEF1	NM_006421
A_32_P894695	TAF1L	NM_153809
A_23_P153571	IGFL2	NM_001002915
A_24_P311604	PACRGL	NM_145048
A_23_P56521	NHEJ1	NM_024782
A_23_P420782	TULP3	NM_003324
A_23_P29769	WWTR1	NM_015472
A_23_P14448	RHOJ	ENST00000316754
A_33_P332710	NLK	NM_016231
A_24_P330625	NFATC2IP	NM_032815
A_23_P25336	GLTP	NM_016433
A_23_P132793	MANF	NM_006010

A_23_P13122	TTC27	NM_017735
A_33_P33085	DUXA	NM_001012729
A_33_P33363	PAOX	NM_207127
A_33_P32589	TRMT2B	NM_024917
A_23_P22033	MAZ	NM_001042539
A_23_P13465	PTCD1	NM_015545
A_32_P30290	ZNF321	NM_203307
A_23_P25357	PHF5A	NM_032758
A_24_P33097	EIF3K	NM_013234
A_23_P20642	CHD9	NM_025134
A_23_P51361	TXLNA	NM_175852
A_23_P20417	TMPO	NM_003276
A_33_P32581	HELLS	NM_018063
A_23_P29282	NPTXR	NM_014293
A_32_P17702	SBDS	NM_016038
A_23_P40685	EP300	NM_001429
A_23_P15888	ZNF93	NM_031218
A_33_P34029	DPRX	NM_001012728
A_23_P20815	DTNA	NM_001392
A_33_P34165	AIG1	NM_016108
A_24_P15814	CCDC25	NM_018246
A_24_P34586	HPSE	ENST00000311412
A_32_P45056	ATXN7L3	NM_020218
A_23_P16502	DAPK3	NM_001348
A_23_P15602	IRX2	NM_033267
A_33_P32860	POLR3E	NM_018119
A_23_P78871	KCTD15	NM_024076
A_24_P17492	HIST1H3B	NM_003537
A_33_P33045	KRTAP5-5	NM_001001480
A_23_P11263	C4orf34	NM_174921
A_23_P43763	PLLP	NM_015993
A_23_P22994	NFYC	NM_014223
A_33_P32371	GPR137C	NM_001099652
A_33_P33754	AGAP11	NM_133447
A_32_P68411	CSNK1A1P1	NR_027320
A_23_P12557	MID1IP1	NM_021242
A_23_P66560	C17orf71	NM_018149
A_23_P34092	C1orf177	NM_152607
A_23_P13835	WNT2B	NM_004185
A_23_P11898	APCDD1	NM_153000
A_23_P16508	NDUFS7	NM_024407
A_23_P10313	SMCR7L	NM_019008
A_23_P78018	ABCA5	NM_018672
A_23_P90752	NBAS	NM_015909
A_24_P27337	CAMKK2	NM_172215
A_23_P10187	FBXO17	NM_024907
A_33_P34211	TP53INP1	NM_033285
A_23_P25564	MKL1	NM_020831
A_33_P32870	GZF1	NM_022482

A_24_P90490	BAT1	NM_004640
A_32_P38964	SCRT2	NM_033129
A_24_P11294	LINGO1	NM_032808
A_23_P41544	NCAPH	NM_015341
A_33_P33335	VPS13C	NM_020821
A_33_P32928	CALR	NM_004343
A_33_P32356	RBBP5	NM_001193272
A_23_P41234	PIGR	NM_002644
A_23_P32165	LHX2	NM_004789
A_33_P32520	JHDM1D	NM_030647
A_23_P25233	C21orf45	NM_018944
A_23_P4561	SERPINB8	NM_198833
A_23_P49878	FAM64A	NM_019013
A_23_P13121	CRYGD	NM_006891
A_23_P12424	MAGED1	NM_001005333
A_23_P75989	PAK1	NM_002576
A_23_P50187	ZFP64	NM_018197
A_23_P34518	FABP3	NM_004102
A_32_P48966	KRT28	NM_181535
A_24_P32788	TCEA3	NM_003196
A_23_P11964	ATP4A	NM_000704
A_23_P37047	POFUT1	NM_015352
A_23_P29365	RYBP	NM_012234
A_23_P10309	RBM9	NM_014309
A_23_P16679	RTP4	NM_022147
A_24_P29991	PASK	NM_015148
A_32_P15169	EXOC4	NM_021807
A_23_P13884	KAT5	NM_006388
A_23_P11182	SPAM1	NM_153189
A_32_P80597	ELOVL6	ENST00000394607
A_23_P10251	SPINK5	NM_006846
A_23_P17821	PLA2G3	NM_015715
A_23_P12562	ANXA8L2	NM_001630
A_23_P17826	SLC5A1	NM_000343
A_23_P38885	MYST4	NM_012330
A_24_P34737	ALOX5AP	NM_001629
A_32_P86173	COBL	NM_015198
A_24_P21544	PHOX2A	NM_005169
A_23_P11874	DERL2	NM_016041
A_23_P29816	PLA1A	NM_015900
A_23_P31957	NR1I3	NM_001077482
A_23_P42271	POLH	NM_006502
A_33_P32929	TCOF1	NM_001008657
A_23_P14691	GAS6	NM_000820
A_24_P14452	ARV1	NM_022786
A_32_P45984	VGLL2	NM_153453
A_23_P60579	MPI	NM_002435
A_24_P18713	FRYL	NM_015030
A_23_P20640	MON1B	ENST00000248248

A_23_P71033	HIP1	NM_005338
A_33_P32573[illegible]	DCBLD1	NM_173674
A_24_P27815[illegible]	TMEM38B	NM_018112
A_33_P33914[illegible]	SOBP	NM_018013
A_24_P17702[illegible]	CDC34	NM_004359
A_33_P32968[illegible]	TP73	NM_001126242
A_23_P21408[illegible]	EGR1	NM_001964
A_23_P41126[illegible]	ZNF707	NM_173831
A_32_P10091[illegible]	PDE4D	NM_001165899
A_23_P10696[illegible]	EVPL	NM_001988
A_23_P97372	DCLRE1B	NM_022836
A_23_P10944[illegible]	HPS4	NM_022081
A_24_P33023[illegible]	UBL3	NM_007106
A_23_P21520[illegible]	FIGNL1	NM_001042762
A_23_P16036[illegible]	THRAP3	NM_005119
A_33_P32573[illegible]	TRIM33	NM_015906
A_23_P20973[illegible]	ARMC9	NM_025139
A_24_P13715	FBXO22	NM_147188
A_24_P96749	EIF2C1	NM_012199
A_23_P47704	UCP2	NM_003355
A_24_P20599[illegible]	EPGN	NM_001013442
A_23_P11968[illegible]	MIER2	NM_017550
A_23_P15697[illegible]	MEST	NM_002402
A_33_P33678[illegible]	CHRM1	NM_000738
A_32_P39646[illegible]	POM121L4P	NR_024592
A_32_P23493[illegible]	TARDBP	NM_007375
A_23_P13991[illegible]	CHST11	NM_018413
A_23_P20979[illegible]	MYO7B	NM_001080527
A_23_P6649	PROK2	NM_021935
A_23_P89646	KRTAP9-2	NM_031961
A_23_P14719[illegible]	ZNF271	NR_024565
A_23_P25964[illegible]	EZH2	NM_004456
A_33_P32210[illegible]	ZAN	NM_173059
A_32_P39442[illegible]	HCG27	NR_026791
A_23_P86493	LBX1	NM_006562
A_23_P28550	ZNF512	NM_032434
A_23_P3242	ANP32A	NM_006305
A_23_P45524	NGFRAP1	NM_014380
A_24_P23409[illegible]	RAB7L1	NM_003929
A_23_P5325	ERCC3	NM_000122
A_24_P81947	CORO1C	NM_014325
A_24_P37020[illegible]	EBI3	NM_005755
A_24_P40630[illegible]	NDUFB2	NM_004546
A_23_P65157	COX17	NM_005694
A_23_P10813[illegible]	AP3D1	NM_003938
A_33_P33646[illegible]	RASGEF1B	NM_152545
A_32_P13845[illegible]	MBD3L1	NM_145208
A_23_P16203[illegible]	ARNTL	NM_001030273
A_23_P20452[illegible]	COL2A1	NM_001844

A_23_P97242	BEST4	NM_153274
A_24_P29052	ZFX	NM_003410
A_24_P17727	CSDC2	NM_014460
A_24_P39699	ATP6V1A	NM_001690
A_23_P12676	MTMR11	NM_001145862
A_23_P10538	THAP2	NM_031435
A_23_P64119	C11orf41	NM_012194
A_23_P11978	TMEM185B	NR_000034
A_24_P10332	FOXD1	NM_004472
A_23_P1292	ERCC6	NM_000124
A_23_P34741	ZNF593	NM_015871
A_23_P91814	SLC25A38	NM_017875
A_33_P32389	NPLOC4	NM_017921
A_33_P32235	FRY	NM_023037
A_32_P16184	XDH	NM_000379
A_23_P50376	ZNF331	NM_018555
A_23_P66813	SIRT7	NM_016538
A_32_P90210	TAF9	NM_001015892
A_24_P92640	LOC10012828	NR_024447
A_24_P36325	CNR1	NM_033181
A_23_P11374	ZNF385D	NM_024697
A_23_P10312	GTPBP1	NM_004286
A_32_P14532	DOCK1	NM_001380
A_33_P33294	MAMSTR	NM_182574
A_23_P17053	KCNH6	NM_173092
A_24_P81029	PPAPDC1A	NM_001030059
A_24_P39143	TAF9B	NM_015975
A_32_P47773	USP4	NM_003363
A_24_P34531	TAL1	NM_003189
A_23_P38451	ZNF22	NM_006963
A_24_P91292	PLK4	NM_014264
A_24_P15100	KRTAP1-1	NM_030967
A_23_P59202	TAF11	NM_005643
A_23_P10739	HES7	NM_032580
A_23_P13738	ID3	NM_002167
A_32_P33044	PARP15	NM_001113523
A_23_P16714	SIX3	NM_005413
A_32_P59302	HIVEP3	ENST00000372583
A_23_P45014	PPID	NM_005038
A_33_P33638	TUBG1	NM_001070
A_33_P32698	LRRC26	NM_001013653
A_23_P10298	TXNRD2	NM_006440
A_23_P43083	HAPLN4	NM_023002
A_23_P13084	PPP1R14A	NM_033256
A_24_P40543	TIA1	NM_022173
A_23_P21822	QPRT	NM_014298
A_23_P78873	KCTD15	NM_024076
A_24_P25992	LANCL1	NM_006055
A_23_P35127	UPP1	NM_181597

A_23_P41966	WNK1	NM_018979
A_23_P36693	KRT6C	NM_173086
A_23_P79692	ABI2	NM_005759
A_23_P32937	POU6F1	NM_002702
A_23_P12244	HIST1H1C	NM_005319
A_33_P32133	CITED2	NM_006079
A_23_P48951	MFGE8	NM_005928
A_23_P34859	SLC25A34	NM_207348
A_23_P13551	PXMP2	NM_018663
A_23_P72513	JUP	NM_021991
A_24_P96527	ATP5L	NM_006476
A_23_P16813	SCUBE3	NM_152753
A_33_P33194	AZIN1	NM_015878
A_24_P35012	RNF213	NM_020954
A_23_P11758	ENTPD5	NM_001249
A_23_P37721	HEXIM2	NM_144608
A_24_P29679	POU2F2	NM_002698
A_23_P30795	SIRT5	NM_031244
A_23_P34038	KIAA1841	NM_001129993
A_23_P21743	ATP1B4	NM_001142447
A_23_P3592	HSF4	NM_001040667
A_23_P21810	WDFY2	NM_052950
A_23_P35885	ZNF644	NM_201269
A_32_P9415	RBBP8	NM_203292
A_32_P41569	EXOC6	NM_019053
A_23_P89283	PITPNA	NM_006224
A_32_P18427	CCDC6	NM_005436
A_23_P58382	LIN54	NM_194282
A_33_P34113	SLC25A6	NM_001636
A_23_P21573	ST7	NM_018412
A_23_P10486	AB529259	AB529259
A_24_P15563	C14orf118	AB032978
A_23_P12432	NSD1	NM_022455
A_23_P13293	DGKQ	NM_001347
A_23_P78952	PIP5K1C	NM_012398
A_24_P53150	TRAF7	NM_032271
A_24_P91992	ARV1	AK092987
A_33_P33351	MURC	NM_001018116
A_23_P74022	DBT	NM_001918
A_23_P15983	NDUFA1	NM_004541
A_23_P18196	RFC4	NM_002916
A_23_P54179	ZNF410	NM_021188
A_32_P92489	PKD1L2	NM_052892
A_23_P3128	YLPM1	NM_019589
A_23_P14791	S100A16	NM_080388
A_23_P21126	RIPK4	NM_020639
A_23_P34461	SFXN2	NM_178858
A_23_P41841	OXSR1	NM_005109
A_23_P21614	TERF1	NM_017489

A_32_P21333(	RGNEF	NM_001080479
A_24_P15108	CNDP2	NM_018235
A_23_P13480	NSMAF	NM_003580
A_23_P15580	ANKRD40	NM_052855
A_32_P39331(	RAPGEF3	NM_001098531
A_33_P34003	PHC3	NM_024947
A_32_P12130	NUP50	NM_007172
A_24_P29057	RBM25	NM_021239
A_23_P14479(	PDLIM4	NM_003687
A_24_P33741	TIGD6	NM_030953
A_33_P33444(	MEF2B	NM_001145785
A_23_P10526	ETV6	NM_001987
A_24_P25033	SNRPA	NM_004596
A_23_P12498	KCNH4	NM_012285
A_23_P12673	IL10	NM_000572
A_32_P38764	FLG	NM_002016
A_23_P39955	ACTG2	NM_001615
A_23_P12933	CLCN7	NM_001287
A_23_P96325	ERCC6L	NM_017669
A_33_P32429	FAM72A	NM_001123168
A_23_P16499	CDC34	NM_004359
A_24_P28936(	LASS6	NM_203463
A_23_P1102	ACTA1	NM_001100
A_32_P47989	HIC1	NM_006497
A_23_P36947	MSI2	NM_170721
A_23_P20327	UBASH3B	NM_032873
A_23_P44144	CTDSP2	NM_005730
A_33_P33442	HS1BP3	BC057389
A_32_P60459	OTUD1	NM_001145373
A_24_P83899	GRM5	NM_000842
A_23_P12866	PSTK	NM_153336
A_32_P11786	SFRS6	NM_006275
A_23_P15310	FHOD3	NM_025135
A_33_P37771(	FLJ31715	BC022164
A_23_P25958(	TTK	NM_003318
A_23_P20158	FEZF1	NM_001024613
A_23_P21636	COL14A1	NM_021110
A_23_P11520	CRNN	NM_016190
A_23_P42908	TMEM139	NM_153345
A_23_P11332	ZBTB41	NM_194314
A_23_P21397	HSPA9	NM_004134
A_23_P10367	NES	NM_006617
A_23_P40883(	CAMKK2	NM_172215
A_23_P47034	HHEX	NM_002729
A_23_P16971	HIST1H3E	BE893548
A_23_P25772(	C9orf156	NM_016481
A_23_P20893	TLE6	NM_024760
A_23_P11094	GSTA4	NM_001512
A_23_P14201	XAB2	NM_020196

A_24_P38852	ST6GAL1	NM_173216
A_24_P19131	SLC1A4	NM_003038
A_23_P21104	BACH1	NM_206866
A_23_P12053	SPEN	NM_015001
A_23_P34213	ADAMTSL1	NM_001040272
A_23_P20484	LCP1	NM_002298
A_23_P15276	TUBG1	NM_001070
A_23_P62081	SCG5	NM_003020
A_23_P63503	RABIF	NM_002871
A_33_P33466	AP2S1	NM_004069
A_23_P65230	TMTC4	NM_032813
A_23_P41381	VKORC1L1	NM_173517
A_24_P32343	CDCA2	NM_152562
A_23_P15534	BTD	NM_000060
A_23_P19030	ARSI	NM_001012301
A_33_P33283	ILDR2	NM_199351
A_23_P25943	DCK	NM_000788
A_24_P40764	SPN	NM_001030288
A_33_P33244	ZFP41	NM_173832
A_24_P21730	ZIC4	NM_032153
A_23_P20398	RHOF	NM_019034
A_32_P9207	STON2	BX648249
A_23_P14966	TMEM183B	NM_001079809
A_23_P50153	HOXA3	NM_153631
A_23_P41065	MLLT6	NM_005937
A_33_P33202	TMEM79	NM_032323
A_32_P17083	KLHL14	NM_020805
A_23_P16973	SOX7	NM_031439
A_33_P32721	FLJ38576	XR_112683
A_23_P20063	FBXO44	NM_001014765
A_33_P33135	PSPC1	NM_001042414
A_24_P41876	SH3RF1	NM_020870
A_32_P53949	PTPN6	NM_002831
A_33_P32330	ATXN3L	NM_001135995
A_23_P75430	C11orf75	NM_020179
A_23_P40136	PITPNM2	NM_020845
A_23_P97652	GPR153	NM_207370
A_23_P10619	FOS	NM_005252
A_23_P14923	HDGF	NM_004494
A_24_P27912	MESP1	NM_018670
A_24_P22561	RRM2	NM_001034
A_23_P42828	GJC1	NM_005497
A_23_P20071	PIK3C2B	NM_002646
A_23_P73982	TMEM48	NM_018087
A_23_P48229	KCNA1	NM_000217
A_23_P35444	INA	NM_032727
A_23_P11361	CDCP1	NM_022842
A_24_P34745	MPP5	NM_022474
A_23_P25329	NLRC5	NM_032206

A_23_P50226[illegible]	POLDIP3	NM_032311
A_24_P28039[illegible]	RNF215	NM_001017981
A_23_P27133	KRT15	NM_002275
A_33_P33332[illegible]	GLIS2	NM_032575
A_23_P41128[illegible]	NR2C2AP	NM_176880
A_32_P53555	WWP1	NM_007013
A_24_P85258	KIAA1751	NM_001080484
A_23_P20224[illegible]	RET	NM_020975
A_24_P45996[illegible]	POM121L10P	BX648116
A_32_P14323[illegible]	SLC2A14	NM_153449
A_24_P21253[illegible]	GALM	NM_138801
A_23_P56150	RAX2	NM_032753
A_33_P33022[illegible]	FOXC2	NM_005251
A_23_P25479[illegible]	TAF9B	NM_015975
A_23_P33477	UVRAG	NM_003369
A_23_P31566	PHTF2	NM_020432
A_23_P15471[illegible]	ESF1	NM_016649
A_32_P10313[illegible]	SOAT1	ENST00000367619
A_23_P40700	TOB2	NM_016272
A_23_P25784[illegible]	PDGFRA	NM_006206
A_24_P34545[illegible]	CYBRD1	NM_024843
A_23_P12817[illegible]	RAB3IP	NM_175623
A_24_P23269[illegible]	SMARCD1	NM_139071
A_32_P85169[illegible]	DAXX	NM_001350
A_23_P375	CDCA8	NM_018101
A_23_P64785	ZNF641	NM_152320
A_23_P21130[illegible]	WDR4	NM_033661
A_23_P42347[illegible]	TYSND1	NM_173555
A_23_P90088	ZNF443	NM_005815
A_23_P69249	ACTL6A	NM_178042
A_23_P33117[illegible]	BHLHA15	NM_177455
A_23_P31386	TRA2A	NM_013293
A_33_P34206[illegible]	NEUROG1	NM_006161
A_24_P9671	DNAJA1	NM_001539
A_24_P36894[illegible]	EVX1	NM_001989
A_23_P34036[illegible]	MTDH	NM_178812
A_32_P13626[illegible]	NSUN7	NM_024677
A_23_P15303[illegible]	ZNF624	NM_020787
A_23_P25336[illegible]	HOXA10	NM_018951
A_23_P20698[illegible]	VEZF1	NM_007146
A_23_P76322	PIK3C2G	NM_004570
A_23_P32714[illegible]	RNF213	NM_020914
A_24_P46577	ZNRD1	NM_170783
A_23_P21246[illegible]	ENTPD3	NM_001248
A_23_P39410	OR10H3	NM_013938
A_32_P41021	ASXL1	NM_015338
A_33_P33980[illegible]	ZPLD1	NM_175056
A_23_P12749[illegible]	BBOX1	NM_003986
A_32_P98072	TCHH	NM_007113

A_23_P14739	DYNC2H1	NM_001080463
A_23_P39263	ZNF57	NM_173480
A_23_P31785	MED26	NM_004831
A_33_P32603	CARHSP1	NM_001042476
A_23_P37137	DNAJC8	NM_014280
A_24_P38226	PIAS2	NM_173206
A_33_P34057	TULP3	NM_003324
A_32_P5480	LASS6	NM_203463
A_32_P31002	ARPM1	NM_032487
A_23_P94319	KBTBD11	NM_014867
A_24_P14100	SYPL1	NM_182715
A_32_P81396	CCDC33	NM_025055
A_32_P35829	XRRA1	NM_182969
A_23_P12985	HIC1	NM_006497
A_23_P37111	DICER1	NM_177438
A_23_P30020	PLA2G12A	NM_030821
A_23_P56091	TMIGD2	NM_144615
A_33_P33989	IRF2BP1	NM_015649
A_23_P43157	MYBL1	NM_001080416
A_32_P86903	KGFLP2	NR_003670
A_23_P79133	PLEKHF1	NM_024310
A_23_P10379	VANGL1	NM_138959
A_23_P14037	NGB	NM_021257
A_24_P91985	BDKRB1	NM_000710
A_23_P15808	CDK9	NM_001261
A_33_P33643	JPH1	NM_020647
A_24_P38218	IGFBP4	NM_001552
A_24_P34583	MSX1	NM_002448
A_32_P41523	AGAP7	NM_001077685
A_24_P83455	C1orf126	NR_027136
A_23_P14324	CEBPB	NM_005194
A_33_P33700	TFE3	NM_006521
A_23_P21384	ATP6V0E1	NM_003945
A_23_P71830	ZBTB26	NM_020924
A_23_P53390	PTPRB	NM_002837
A_23_P16844	RASA4	NM_006989
A_23_P10465	CDCA5	NM_080668
A_23_P45699	FUBP1	NM_003902
A_23_P39612	SSX5	NM_021015
A_32_P23515	MSL3L2	NR_024322
A_23_P16479	ZNF580	NM_016202
A_24_P19599	ANKRD17	NM_032217
A_23_P39427	RWDD4A	NM_152682
A_23_P48246	PEX5	NM_000319
A_23_P6151	PLAGL2	NM_002657
A_24_P19359	CCNF	NM_001761
A_24_P92944	ARID5A	AY358167
A_23_P11514	WDR77	NM_024102
A_24_P92594	ZXDC	AY358102

A_23_P43595	KIF24	AK001795
A_23_P97310	NT5C1A	NM_032526
A_23_P97365	LRRC8D	NM_018103
A_32_P21383	FAM40B	NM_020704
A_33_P34150	CLCN5	NM_001127899
A_24_P40258	BCL11A	NM_018014
A_24_P37500	YBX1	NM_004559
A_24_P41118	BCL11A	NM_022893
A_23_P25952	WDR41	NM_018268
A_23_P15407	IHH	NM_002181
A_23_P15347	KLK5	NM_012427
A_32_P20276	FAM171B	NM_177454
A_33_P33807	C20orf160	NM_080625
A_23_P20814	ZNF397	NM_001135178
A_23_P8038	ZNRD1	NM_170783
A_33_P32279	SLC25A34	NM_207348
A_23_P12230	HDAC2	NM_001527
A_23_P37263	CHD9	NM_025134
A_23_P12839	RNF34	NM_194271
A_23_P41320	EIF3K	NM_013234
A_23_P78698	RAB3D	NM_004283
A_23_P10918	INSM1	NM_002196
A_23_P13120	NR4A2	NM_006186
A_24_P18469	NKX2-1	NM_003317
A_23_P26503	GDPD3	NM_024307
A_23_P23941	ZNF37A	NM_001007094
A_33_P33896	NOP14	NM_003703
A_23_P28133	SCTR	NM_002980
A_32_P94094	MYST2	NM_007067
A_23_P69437	YEATS2	NM_018023
A_24_P34864	NDUFA1	NM_004541
A_23_P27404	ZNF841	NM_001136499
A_32_P17118	NBPF10	NM_001039703
A_33_P34068	NFASC	NM_001005389
A_24_P37293	PEX5	NM_000319
A_23_P16610	TMX4	NM_021156
A_32_P53900	SLC25A4	NM_001151
A_33_P33942	C2orf67	NM_152519
A_24_P20607	CXCL11	NM_005409
A_24_P20747	DEDD2	NM_133328
A_33_P33482	BRI3	NM_015379
A_23_P34565	WWC3	NM_015691
A_24_P35057	TNIK	NM_015028
A_33_P33411	INTS1	NM_001080453
A_24_P19299	FADS1	NM_013402
A_23_P31745	DNAJB3	NM_001001394
A_33_P32553	BPIL2	NM_174932
A_23_P11015	ARHGAP24	NM_001025616
A_23_P34508	ZNF655	NM_001083956

A_24_P94222	FBLIM1	NM_017556
A_24_P40613	MAPK13	NM_002754
A_24_P25273	KLF6	NM_001300
A_24_P10789	TXNDC11	NM_015914
A_23_P20569	DLST	NM_001933
A_24_P12528	HDAC5	NM_001015053
A_23_P20963	CDC42EP3	NM_006449
A_23_P28598	DLX2	NM_004405
A_23_P15853	DEAF1	NM_021008
A_32_P21345	DMRT2	NM_006557
A_33_P33233	ZNF417	NM_152475
A_23_P14890	CAP1	NM_006367
A_23_P35331	SMURF1	NM_020429
A_23_P16076	TUFT1	NM_020127
A_33_P33366	ALDH3A2	NM_000382
A_23_P10034	ORC6L	NM_014321
A_32_P22974	DNAJB6	NM_005494
A_33_P32486	DUX4	NM_033178
A_23_P15167	PPM1A	NM_021003
A_23_P14551	IL20RA	NM_014432
A_23_P42778	LAYN	NM_178834
A_23_P11105	HIST1H2BB	NM_021062
A_24_P33590	ZNF266	NM_006631
A_23_P24028	TLX1	NM_005521
A_23_P54576	KIFC3	NM_005550
A_23_P14423	PSMD2	NM_002808
A_32_P13696	KLHL15	NM_030624
A_23_P28068	GTPBP3	NM_133644
A_23_P15848	TSPYL2	NM_022117
A_24_P23483	PCDH1	NM_032420
A_23_P14399	FANCD2	NM_001018115
A_23_P20832	SPTAN1	NM_003127
A_32_P46413	DACH2	NM_053281
A_23_P79732	RPS27A	NM_002954
A_23_P11729	ZBTB7B	NM_015872
A_24_P41524	PHF21A	NM_016621
A_32_P33793	DMRTA2	NM_032110
A_23_P38417	HOXB8	NM_024016
A_23_P10331	S100A7	NM_002963
A_24_P34794	GIT1	NM_014030
A_23_P98844	ARHGEF25	NM_182947
A_23_P25168	COMT	NM_000754
A_23_P44295	CLASP2	NM_015097
A_23_P11591	PHYH	NM_001037537
A_32_P34109	GATAD2B	NM_020699
A_23_P13075	DBP	NM_001352
A_24_P12305	RAP2B	NM_002886
A_33_P32526	CYTL1	NM_018659
A_23_P99604	G2E3	NM_017769

A_24_P36926	MMS19	NM_022362
A_24_P32844	TLX2	NM_016170
A_33_P32283	ATPIF1	NM_016311
A_32_P16968	HNF4A	AK096973
A_23_P21870	ZNF343	NM_024325
A_23_P14578	MLXIPL	NM_032951
A_23_P95002	HIPK1	NM_198268
A_24_P93174	STBD1	AK074635
A_24_P25919	SH3BP1	NM_018957
A_23_P24835	PTH	NM_000315
A_23_P30339	MYCN	NM_005378
A_23_P65068	EID3	NM_001008394
A_23_P33573	KRT3	NM_057088
A_23_P21819	BANP	NM_079837
A_32_P78319	NPAS3	NM_001164749
A_23_P16139	CHERP	NM_006387
A_23_P91151	SLC1A4	NM_003038
A_23_P15695	NCOA7	NM_181782
A_24_P10907	KIF27	NM_017576
A_23_P59418	NRF1	NM_005011
A_23_P23547	SERTAD4	NM_019605
A_33_P32721	REXO4	NM_020385
A_23_P71415	WDYHV1	NM_018024
A_23_P49481	USP7	NM_003470
A_24_P40206	SEC63	NM_007214
A_23_P33780	IL29	NM_172140
A_32_P34625	MLXIP	NM_014938
A_24_P23070	NRAP	NM_198060
A_33_P33251	CANX	NM_001746
A_24_P93142	TCF4	NM_001083962
A_23_P28614	RHOQ	NM_012249
A_33_P33412	CACNG4	NM_014405
A_32_P99330	FHDC1	NM_033393
A_24_P92237	ERG	NM_004449
A_32_P43035	DDX54	NM_024072
A_32_P45482	TNFRSF10A	NM_003844
A_23_P20490	CENPJ	NM_018451
A_32_P30649	ETV5	NM_004454
A_23_P20786	KRTAP9-4	NM_033191
A_33_P32095	EBF4	NM_001110514
A_23_P30597	GRAMD2	NM_001012642
A_33_P32298	TBPL2	NM_199047
A_23_P17808	SEC14L2	NM_012429
A_23_P15812	MTA1	NM_004689
A_24_P9285	LMAN2	NM_006816
A_23_P25738	GLRA3	NM_006529
A_24_P40563	FEZF2	NM_018008
A_23_P14901	KHDRBS1	NM_006559
A_23_P25265	STK25	NM_006374

A_23_P48663	OTX2	NM_021728
A_24_P10107:	NBPF10	NM_001039703
A_23_P15307(	BCAS3	NM_017679
A_23_P13482:	ASH2L	NM_004674
A_32_P34015!	ZCCHC12	NM_173798
A_24_P92432!	ZNF234	NM_006630
A_23_P14919-	ZP4	NM_021186
A_23_P32071:	COG4	NM_015386
A_32_P21704:	JHDM1D	NM_030647
A_33_P33431(	ETV2	NM_014209
A_23_P15302:	KRTAP2-4	NM_033184
A_23_P27265	DSEL	NM_032160
A_24_P23400	SLC6A8	NM_005629
A_23_P73043	CYP27C1	NM_001001665
A_23_P31073	MYB	NM_005375
A_24_P36360!	IRF5	NM_001098627
A_23_P56759	KRCC1	NM_016618
A_23_P42276(	KLHDC1	NM_172193
A_23_P16232:	WNT10B	NM_003394
A_33_P33650:	LCE6A	NM_001128600
A_23_P84670	GRIN1	NM_007327
A_23_P30454:	NFX1	NM_147133
A_23_P14170-	TCEB3B	NM_016427
A_33_P32206:	FAM89B	NM_001098784
A_23_P79911	PSMF1	NM_006814
A_23_P69055	ZNF197	NM_006991
A_23_P37068:	BATF2	NM_138456
A_23_P11881!	BIRC5	NM_001012271
A_23_P44335	ENTPD8	NM_001033113
A_23_P43530	SETX	NM_015046
A_23_P39456:	KIAA1467	NM_020853
A_24_P27688(	CENPO	ENST00000380834
A_23_P14253:	COL3A1	NM_000090
A_23_P15940(	SPRR1B	NM_003125
A_23_P39546-	ZNF582	NM_144690
A_32_P34394:	ANXA8L2	NM_001630
A_23_P21485(	GCNT2	NM_001491
A_23_P20590(	NTRK3	NM_001012338
A_24_P7693	KANK3	NM_198471
A_23_P11157:	HOXA3	NM_153631
A_23_P33022	POLR2L	NM_021128
A_32_P23200!	HSF5	NM_001080439
A_23_P39492(	SRCAP	NM_006662
A_32_P16854	ANAPC13	NM_015391
A_23_P97442	TMEM222	NM_032125
A_24_P26359!	RUNDC2C	NR_002939
A_33_P33359-	HN1L	NM_144570
A_24_P20426!	MLL3	NM_170606
A_23_P33234(	MORN3	NM_173855

A_23_P21768{	TSC22D3	NM_004089
A_23_P12582!	PGK1	NM_000291
A_32_P91521:	ZNF705D	NM_001039615
A_23_P41903{	CLOCK	NM_004898
A_24_P21547!	ZNF10	NM_015394
A_23_P32430	ALK	NM_004304
A_33_P33191	HTR2C	NM_000868
A_24_P11473!	CAMK2D	NM_172127
A_23_P56938	REL	NM_002908
A_23_P15689(	TCF21	NM_003206
A_23_P21754(	TMLHE	NM_018196
A_23_P12861:	KDELC1	NM_024089
A_23_P21290!	CXCL1	NM_001511
A_23_P61778	NXN	NM_022463
A_23_P12818:	PFDN5	NM_002624
A_23_P13977!	PPTC7	NM_139283
A_24_P54253	PARS2	NM_152268
A_23_P20854(	NDUFA3	NM_004542
A_24_P22706!	PITX3	NM_005029
A_23_P33703:	GMEB2	NM_012384
A_24_P35824	MON1B	NM_014940
A_23_P16944(	PTGES	NM_004878
A_24_P35017:	ACSM5	NM_017888
A_32_P50634{	POU5F2	NM_153216
A_23_P16632	ZNF257	NM_033468
A_33_P32341(	SETD1A	NM_014712
A_23_P14338!	FOXS1	NM_004118
A_33_P33532!	PHOX2A	NM_005169
A_23_P37910(	ENST0000031	ENST00000317596
A_33_P33446(	ZNF280D	NM_001002844
A_33_P32899!	USP45	NM_001080481
A_23_P16492:	SYNGR4	NM_012451
A_33_P33707:	PGC	NM_001166424
A_23_P41495{	PLXNC1	NM_005761
A_23_P21124	PRMT2	NM_206962
A_23_P20198{	MASTL	NM_032844
A_23_P25369!	GPR64	NM_001079858
A_23_P10830:	NDUFA7	NM_005001
A_32_P38060	TCF12	AK022018
A_32_P25295	NEUROD2	NM_006160
A_23_P15032!	TMEM133	NM_032021
A_23_P40714:	LUZP1	NM_033631
A_23_P20544!	CDCA4	NM_017955
A_23_P20287(	CALCA	NM_001033953
A_23_P20571:	STXBP6	NM_014178
A_23_P30867:	TAOK2	NM_004783
A_23_P15326:	ZNF773	NM_198542
A_23_P11106{	ZNF391	NM_001076781
A_33_P33608:	C10orf88	NM_024942

A_32_P20275[illegible]	FAM171B	NM_177454
A_23_P44244	SMARCA1	NM_003069
A_32_P29759	IK	NM_006083
A_23_P38303[illegible]	CC2D1B	NM_032449
A_23_P10875[illegible]	FHL2	NM_001039492
A_23_P20587[illegible]	SCAND2	NR_004859
A_23_P16404[illegible]	ERN1	NM_001433
A_23_P43441[illegible]	SOX1	NM_005986
A_33_P33189[illegible]	HAPLN2	NM_021817
A_23_P10378[illegible]	HIPK1	NM_198268
A_23_P39846[illegible]	HK2	NM_000189
A_24_P29634[illegible]	RRN3	NM_018427
A_23_P42887[illegible]	TNFAIP8L1	NM_152362
A_33_P33330[illegible]	SGSM2	AK124883
A_23_P39560[illegible]	FAM110B	NM_147189
A_24_P16706[illegible]	ZNF518A	NM_014803
A_33_P33117[illegible]	KIF23	NM_138555
A_24_P91269[illegible]	OTUD7A	ENST00000307050
A_23_P58606	CCNG1	NM_004060
A_23_P14071[illegible]	TPSD1	NM_012217
A_24_P28906[illegible]	SLC6A14	NM_007231
A_23_P10468[illegible]	RELA	NM_021975
A_23_P20980[illegible]	NAB1	NM_005966
A_23_P89589	PER1	NM_002616
A_23_P37068[illegible]	TIGD4	NM_145720
A_32_P14791[illegible]	TMEM183B	NM_001079809
A_23_P20001[illegible]	AK5	NM_174858
A_23_P69695	CISD2	NM_001008388
A_23_P13697[illegible]	SRPX2	NM_014467
A_23_P30593[illegible]	ZNF75D	NM_007131
A_23_P91562	C2CD2	NM_015500
A_24_P39454	INO80	NM_017553
A_23_P16212[illegible]	NUMA1	NM_006185
A_24_P28033[illegible]	KRT40	NM_182497
A_24_P46417	STK10	NM_005990
A_33_P33813[illegible]	PAX1	NM_006192
A_24_P16606	ZFP41	AK024438
A_32_P91661[illegible]	TSHZ3	NM_020856
A_23_P96903	SMCP	NM_030663
A_32_P37528[illegible]	ZNF660	NM_173658
A_24_P29677[illegible]	PPP1R14A	NM_033256
A_23_P10309[illegible]	RBM9	NM_001031695
A_23_P39154	ZNF345	NM_003419
A_23_P11231[illegible]	TRIM32	NM_012210
A_23_P21307[illegible]	POU4F2	NM_004575
A_33_P33430[illegible]	PAX2	NM_003988
A_24_P11680[illegible]	STAT3	NM_213662
A_33_P33689[illegible]	ANKRD30A	NM_052997
A_23_P16610[illegible]	FLRT3	NM_198391

A_23_P35890	IKZF4	NM_022465
A_33_P33364	TTLL5	NM_015072
A_24_P17776	CDADC1	NM_030911
A_32_P16222	ROR2	NM_004560
A_23_P98402	SIDT2	NM_001040455
A_23_P11528	BCL10	NM_003921
A_23_P20380	SLC26A10	ENST00000257962
A_23_P40828	PRICKLE1	NM_153026
A_23_P15161	PSME1	NM_006263
A_23_P16021	TTC39A	NM_001080494
A_33_P33535	IGBP1	NM_001551
A_24_P29711	OLIG1	NM_138983
A_23_P16194	SCGB2A2	NM_002411
A_32_P3602	USP3	NM_006537
A_33_P33977	C14orf135	AK095489
A_32_P14827	SPIC	NM_152323
A_23_P41948	CCDC99	NM_017785
A_23_P78423	ATP5A1	NM_001001937
A_23_P47565	LDHA	NM_005566
A_24_P86079	PAIP2B	NM_020459
A_23_P43476	NUP50	NM_007172
A_33_P32960	ZNF131	NM_003432
A_23_P3748	ZNF205	NM_003456
A_33_P32918	CEP55	NM_018131
A_23_P11803	NUTF2	NM_005796
A_23_P39555	ZNF226	NM_001032372
A_23_P37302	CACNA1C	NM_000719
A_23_P13516	UAP1L1	NM_207309
A_32_P2791	MSRB2	NM_012228
A_32_P15376	KIAA1324	NM_020775
A_33_P34059	ST3GAL3	NM_174963
A_24_P40666	RAD23B	NM_002874
A_23_P7361	ELOVL6	NM_024090
A_23_P16132	CHERP	NM_006387
A_24_P17922	MATN2	NM_030583
A_24_P23112	WDR13	L08237
A_24_P88870	B3GALNT1	NM_001038628
A_24_P88775	ZNF514	NM_032788
A_24_P23531	VDAC3	NM_005662
A_23_P21668	BRD3	NM_007371
A_24_P72394	RBAK	NM_021163
A_23_P56404	EN1	NM_001426
A_23_P5995	ARFGEF2	NM_006420
A_23_P11826	ZFHX3	NM_006885
A_32_P11393	LNX2	NM_153371
A_24_P17505	ATG5	NM_004849
A_23_P20699	MTMR4	NM_004687
A_23_P97770	RNPEP	NM_020216
A_24_P25443	CBX6	ENST00000407418

A_33_P32589	SIRT2	NM_001193286
A_24_P36724	KRTAP19-1	NM_181607
A_33_P32217	RUNX3	NM_001031680
A_32_P53892	DSG4	NM_177986
A_23_P31848	ATAD3C	NM_001039211
A_32_P84388	GMCL1	NM_178439
A_24_P25632	REV1	NM_001037872
A_23_P13502	CHCHD8	NM_016565
A_24_P91728	ALDH1A3	BC009245
A_24_P17961	TPR	NM_003292
A_33_P32260	GATSL3	NM_001037666
A_24_P38385	EIF4G3	NM_003760
A_32_P46321	RXRB	NM_021976
A_33_P32391	ZNF497	NM_198458
A_23_P13156	RNF144A	NM_014746
A_23_P37423	SSX7	NM_173358
A_32_P35332	ZFHX3	NM_006885
A_33_P34206	KDM4A	NM_014663
A_23_P14821	GABRB3	NM_000814
A_32_P18920	GAS2L3	NM_174942
A_24_P21507	SPAG6	NM_172242
A_24_P29753	MAMSTR	NM_182574
A_33_P32196	CEP78	NM_001098802
A_33_P32429	ZNF563	NM_145276
A_33_P33742	NHLH2	ENST00000369506
A_24_P10265	MUC5B	NM_002458
A_23_P12084	XBP1	NM_005080
A_32_P36333	KY	NM_178554
A_23_P32820	DNMBP	NM_015221
A_32_P22691	PKN2	NM_006256
A_23_P13696	RPGR	NM_000328
A_23_P18276	RBM5	NM_005778
A_32_P23051	TET2	NM_001127208
A_23_P20843	TPM4	NM_003290
A_32_P42630	GALK2	NM_001001556
A_33_P32096	WDFY2	NM_052950
A_24_P40594	HOMEZ	NM_020834
A_24_P43826	EBF2	NM_022659
A_24_P33082	HNF1B	NM_000458
A_23_P39590	XDH	NM_000379
A_24_P79632	LEUTX	NM_001143832
A_33_P33495	TMEM66	NM_016127
A_23_P73858	TGIF2LY	NM_139214
A_24_P94216	NRAP	NM_198060
A_23_P15078	PRSS23	NM_007173
A_23_P14339	PLUNC	NM_016583
A_23_P41280	PAICS	NM_001079525
A_33_P33078	LOC10012865	XR_108727
A_23_P67086	ZNF835	ENST00000342088

A_23_P14939	TXNDC11	NM_015914
A_24_P85123	OXCT2	AK024440
A_23_P78950	PIP5K1C	NM_012398
A_33_P33707	EPHB2	NM_004442
A_23_P11978	TMEM185B	NR_000034
A_23_P12671	ATPIF1	NM_178191
A_23_P37058	HOXB8	NM_024016
A_23_P35786	SEC62	NM_003262
A_24_P22094	AKR1C1	NM_001353
A_23_P30695	ZNF566	NM_032838
A_23_P14169	PARD6G	NM_032510
A_23_P43425	C9orf40	NM_017998
A_32_P10554	ANXA8L2	NM_001630
A_23_P46975	MMS19	NM_022362
A_23_P21522	DNAJB6	NM_005494
A_24_P13175	DLG1	NM_004087
A_24_P91167	PDE5A	NM_033430
A_32_P88677	KIF26A	NM_015656
A_23_P78014	TUBG2	NM_016437
A_24_P11941	HSF5	NM_001080439
A_23_P53603	ATP2A2	NM_001681
A_23_P93722	SDK1	NM_152744
A_33_P34239	GBX2	NM_001485
A_33_P32554	PIH1D1	NM_017916
A_23_P81207	ZNF141	NM_003441
A_23_P44617	COPG	NM_016128
A_32_P87703	MAT2A	NM_005911
A_23_P13459	XRCC2	NM_005431
A_23_P42882	CAMK2B	NM_172082
A_32_P20413	GABRA4	NM_000809
A_32_P47299	TCEAL8	NM_153333
A_23_P20916	FSTL3	NM_005860
A_23_P54575	ZFPM1	NM_153813
A_23_P73526	CITED1	NM_004143
A_33_P32840	FOXP4	NM_001012426
A_23_P16099	FMO4	NM_002022
A_32_P2738	TCTN3	NM_015631
A_23_P36994	FOXRED2	NM_024955
A_24_P21805	NUDCD3	NM_015332
A_33_P33166	BMP3	NM_001201
A_24_P34047	MAST4	NM_001164664
A_23_P20894	EHMT1	NM_024757
A_33_P33992	LINGO1	NM_032808
A_23_P10934	KRTAP4-1	NM_033060
A_23_P11297	SHOX2	NM_006884
A_33_P32393	NKX3-1	NM_006167
A_23_P13596	PCMTD1	NM_052937
A_23_P87500	ORMDL2	NM_014182
A_23_P37974	DENND5B	NM_144973

A_23_P64048	CDC42BPG	ENST00000342711
A_23_P15939	TOPBP1	NM_007027
A_23_P25107	MAMLD1	NM_005491
A_23_P11858	PSMC3IP	NM_013290
A_23_P37606	IKZF3	NM_012481
A_33_P34075	SUV39H1	NM_003173
A_23_P50179	MED22	NM_133640
A_33_P33275	TMEM41A	AK172748
A_24_P51683	CDK5R2	NM_003936
A_23_P42338	CREB3	NM_006368
A_23_P21188	RARB	NM_000965
A_24_P22657	KLHL11	ENST00000319121
A_33_P33879	CEBPE	NM_001805
A_23_P20882	ITGB1BP3	NM_170678
A_24_P36347	FTSJ2	NM_013393
A_23_P16216	KCTD14	NM_023930
A_23_P12742	TM7SF2	NM_003273
A_32_P14805	RUNX2	NM_001015051
A_23_P33894	MAGED2	NM_201222
A_23_P30693	PPTC7	NM_139283
A_23_P14620	RNF170	NM_030954
A_23_P13348	G3BP1	NM_005754
A_23_P15146	IL32	NM_001012631
A_23_P16495	CCDC8	NM_032040
A_23_P21362	PPP2R2B	NM_004576
A_23_P54663	CES2	NM_003869
A_33_P33961	CCDC6	NM_005436
A_32_P11457	CACYBP	NM_014412
A_23_P58671	TPPP	NM_007030
A_23_P25286	PCMT1	NM_005389
A_23_P20813	BCL2	M13995
A_23_P20842	F2RL3	NM_003950
A_24_P41913	CENPI	NM_006733
A_23_P5460	FEV	NM_017521
A_23_P41249	NHLH2	NM_005599
A_23_P11713	PTP4A2	NM_080392
A_23_P1823	PAX6	NM_000280
A_32_P42167	TYW1	NM_018264
A_23_P11050	CLPTM1L	NM_030782
A_23_P24129	DKK1	NM_012242
A_24_P4701	PPME1	NM_016147
A_24_P35718	FTSJ1	NM_177439
A_33_P32728	RAD1	NM_002853
A_23_P32073	MEF2C	NM_002397
A_23_P73801	TCEAL1	NM_001006640
A_33_P33557	GPATCH2	NM_018040
A_24_P19965	VANGL1	NM_138959
A_32_P22245	TMEM158	NM_015444
A_24_P39344	DAPK1	NM_004938

A_32_P38052	TRPS1	NM_014112
A_23_P50204	CHRD	NM_003741
A_23_P16347	C15orf52	NM_207380
A_24_P34743	FOXA1	NM_004496
A_24_P41428	NIT2	NM_020202
A_24_P92368	SIRT3	NM_012239
A_33_P34147	FSD1	NM_024333
A_32_P12179	EXOSC6	CR604283
A_33_P34002	ZNF496	NM_032752
A_24_P11772	CHRD	NM_003741
A_24_P73290	ATP2A2	NM_001681
A_32_P32173	FAM167A	NM_053279
A_23_P27373	TSHZ1	NM_005786
A_32_P48839	GLDN	AY358144
A_23_P41040	OR7D2	NM_175883
A_23_P30972	GABRD	NM_000815
A_23_P13340	CSF2	NM_000758
A_23_P87827	UNC119B	NM_001080533
A_24_P35185	LAT2	NM_032464
A_33_P34093	COX15	NM_078470
A_23_P32669	RBBP4	NM_005610
A_24_P93341	ABI2	NM_005759
A_23_P26810	TP53	NM_000546
A_23_P51397	ENAH	NM_001008493
A_23_P59613	FZD9	NM_003508
A_24_P76313	FAM43B	NM_207334
A_32_P10798	POM121L9P	NR_003714
A_23_P34992	SP100	NM_003113
A_23_P87528	BCAT1	NM_005504
A_23_P55281	HOXB7	NM_004502
A_32_P16790	ZNF681	NM_138286
A_24_P15389	MEN1	NM_130803
A_33_P32130	KRTAP1-3	NM_030966
A_24_P34151	ZNF154	NM_001085384
A_24_P85557	GPR37	NM_005302
A_24_P32342	C2orf67	NM_152519
A_23_P20069	LMO4	NM_006769
A_23_P37352	HAND2	NM_021973
A_24_P18548	ZNF507	NM_014910
A_23_P32913	GOT2	M22632
A_23_P14745	SPG21	NM_016630
A_23_P44149	CTDSP2	NM_005730
A_23_P34471	SYNJ2	NM_003898
A_24_P64714	HDAC7	NM_015401
A_23_P23850	DAB1	NM_021080
A_23_P17122	AMOT	ENST00000304758
A_24_P10205	OCLN	NM_002538
A_23_P53957	COX16	NM_016468
A_23_P14576	ARL4A	NM_005738

A_24_P65584	SMAD9	NM_001127217
A_23_P14917	FAM63A	NM_001040217
A_32_P88955	INADL	NM_176877
A_23_P21222	ARL8B	NM_018184
A_23_P52657	PYGM	NM_005609
A_24_P33294	ATG2B	NM_018036
A_23_P35066	TTC8	NM_144596
A_23_P11920	CD70	NM_001252
A_23_P11266	FOXE3	NM_012186
A_23_P33791	SSBP2	NM_012446
A_23_P11487	HOOK1	NM_015888
A_23_P13434	NEUROD6	NM_022728
A_23_P20740	BRCA1	NM_007300
A_32_P20687	ZNF135	AL157426
A_23_P21498	MICAL1	NM_022765
A_33_P33662	UGGT1	NM_020120
A_23_P12066	RPS21	NM_001024
A_24_P23391	KIAA0494	NM_014774
A_23_P16043	MYOG	NM_002479
A_23_P61123	COX16	NM_016468
A_33_P32998	UQCRB	NM_001199975
A_23_P11520	FCRL4	NM_031282
A_33_P33522	MTUS1	NM_001001925
A_24_P46212	FGFRL1	NM_001004356
A_23_P81529	ISL1	NM_002202
A_23_P9823	MLXIP	NM_014938
A_33_P34113	UNCX	NM_001080461
A_23_P49546	GRIN2C	NM_000835
A_32_P22581	PRDM16	NM_022114
A_23_P36757	HNF1A	NM_000545
A_23_P16086	LRIG2	NM_014813
A_33_P33568	MTX3	NM_001010891
A_33_P34023	ZBTB7C	NM_001039360
A_24_P76725	DCAF8	NR_028105
A_23_P15983	ZNF175	NM_007147
A_24_P95198	PRRC2B	NM_013318
A_23_P35724	ZNF343	NM_024325
A_33_P33123	CIT	NM_007174
A_24_P12844	TBX15	NM_152380
A_24_P98975	ANKRD13A	NM_033121
A_23_P15789	DMRT3	NM_021240
A_33_P32204	SMAD6	NM_005585
A_23_P25131	CTNND1	NM_001331
A_32_P13484	AP4S1	NM_007077
A_23_P78782	CA11	NM_001217
A_24_P16225	HHIPL1	AK095603
A_23_P11526	AGT	NM_000029
A_23_P4640	ZNF548	NM_152909
A_23_P13046	ZNF8	NM_021089

A_23_P95755	PFKFB3	NM_004566
A_23_P5273	SBNO2	NM_014963
A_23_P30650(	KRAS	NM_033360
A_24_P33986(	ZNF295	NM_020727
A_23_P50160(	DRD3	NM_033663
A_23_P87591	YEATS4	NM_006530
A_33_P32687(	ZKSCAN4	NM_019110
A_23_P10767(	ZFP28	NM_020828
A_23_P50455	POLD1	NM_002691
A_23_P76992	PGF	NM_002632
A_24_P38053(	CD164	NM_006016
A_23_P30453(	LCE5A	NM_178438
A_24_P10074(	ADD1	NM_014189
A_23_P11119(	SPDEF	NM_012391
A_32_P21136(	AGAP4	NM_133446
A_24_P44916	CDC42EP5	NM_145057
A_24_P16038(	PDLIM2	NM_176871
A_32_P18316(	RWDD4A	NM_152682
A_23_P32474	CDCP1	NM_022842
A_24_P29401	PIK3R1	NM_181523
A_23_P20820(	ZNF649	NM_023074
A_33_P33760(	TXLNA	NM_175852
A_23_P51926	PTAFR	NM_000952
A_23_P54669	SALL1	NM_002968
A_32_P38646(	ZNF805	NM_001023563
A_24_P94251(	TMX4	ENST00000246024
A_23_P13564(	IKZF1	NM_006060
A_23_P13087(	ZNF546	NM_178544
A_23_P39438	ZNF431	NM_133473
A_33_P33157(	TP53	NM_000546
A_23_P20784(	RARA	NM_001024809
A_33_P33369(	MRPS18B	NM_014046
A_23_P47839	DDX55	NM_020936
A_24_P97197	CTDSP1	NM_182642
A_23_P39900(	CXXC5	NM_016463
A_23_P15038(	FXYD6	NM_022003
A_23_P2502	PAH	NM_000277
A_23_P13854(	AKR1C3	NM_003739
A_23_P21830(	GLYR1	NM_032569
A_23_P761	PSMB4	NM_002796
A_24_P15048(	SPTLC2	NM_004863
A_23_P11869(	ZNF207	NM_001032293
A_32_P36490(	TNNI1	NM_003281
A_33_P32120(	ELF2	NM_201999
A_32_P80981(	LOC10013043(	AK096255
A_32_P46486(	ALX4	NM_021926
A_23_P98085	PTEN	NM_000314
A_23_P50067(	IL5RA	NM_175725
A_23_P39158(	TPM1	NM_001018004

A_33_P32330	MBTD1	NM_017643
A_24_P16607	NAP1L5	NM_153757
A_33_P32546	PDIA5	NM_006810
A_23_P14071	TPSD1	NM_012217
A_33_P32451	HRH1	NM_001098213
A_23_P76560	LRCH1	NM_001164213
A_33_P32877	MYST4	NM_012330
A_23_P27306	COLEC12	NM_130386
A_23_P71037	IL6	NM_000600
A_24_P38702	NKX2-3	NM_145285
A_33_P32580	RNPEP	NM_020216
A_24_P49349	RABGAP1L	NM_014857
A_24_P25496	HDAC8	NM_018486
A_24_P18917	LMF1	NM_022773
A_33_P32094	RELA	NM_021975
A_32_P23515	MSL3L2	NR_024322
A_24_P39049	CX3CL1	NM_002996
A_23_P43675	ZNF618	NM_133374
A_23_P65577	COQ6	NM_182476
A_23_P84587	WNK1	NM_018979
A_23_P44643	ANAPC7	NM_016238
A_23_P79599	TMEM18	NM_152834
A_33_P32400	PDE3B	NM_000922
A_24_P35045	RUNDC2B	AK023827
A_23_P91446	MMP24	NM_006690
A_23_P25656	TLR6	NM_006068
A_23_P20440	KDM5A	NM_001042603
A_23_P47843	DDX55	NM_020936
A_23_P20939	CFLAR	NM_001127184
A_32_P95313	TCEA1	NM_006756
A_23_P53663	PAWR	NM_002583
A_32_P53883	PPAPDC1A	NM_001030059
A_23_P34012	TRIM29	NM_012101
A_23_P20364	CREBZF	NM_001039618
A_24_P85009	FGF13	NM_001139498
A_23_P62764	CCDC28B	NM_024296
A_23_P70520	CDSN	NM_001264
A_23_P9513	MTA1	NM_004689
A_23_P28090	STX10	NM_003765
A_32_P32024	NEUROD4	NM_021191
A_23_P40720	ZEB2	NR_033258
A_32_P4018	ROR1	ENST00000371079
A_23_P57459	CABP7	NM_182527
A_32_P21571	RPL6	NM_001024662
A_24_P39639	EXOSC6	NM_058219
A_23_P21741	SMC1A	NM_006306
A_23_P11564	KIAA0494	NM_014774
A_23_P73451	CELF4	NM_020180
A_23_P43552	TMEM106A	NM_145041

A_24_P175909	MARS	NM_004990
A_23_P152818	MYST2	NM_007067
A_23_P161209	DLG5	NM_004747
A_33_P323692	PARP1	NM_001618
A_32_P486095	BMPER	NM_133468
A_24_P353732	SPEN	NM_015001
A_23_P30122	IL2	NM_000586
A_33_P339038	CELF3	NM_001172649
A_23_P419150	VENTX	NM_014468
A_23_P12363	ROR1	NM_005012
A_33_P340875	FOXO6	ENST00000401063
A_24_P85322	ZFYVE26	BC008927
A_23_P98369	GRIA4	NM_000829
A_24_P75056	POLD3	NM_006591
A_23_P256948	MSC	NM_005098
A_23_P398560	NR4A3	NM_006981
A_24_P312417	ZBTB26	ENST00000373656
A_32_P327190	MYT1L	NM_015025
A_24_P406939	C11orf1	ENST00000260276
A_24_P102638	RB1	NM_000321
A_23_P390700	CNTN1	NM_001843
A_23_P405320	MORN4	NM_178832
A_24_P10214	STXBP6	NM_014178
A_23_P409449	TAL2	NM_005421
A_32_P301290	FHDC1	NM_033393
A_24_P937095	SLC30A1	ENST00000367001
A_24_P67395	KRT8	NM_002273
A_23_P20566	TPM2	NM_213674
A_32_P750514	EFCAB6	Y10776
A_23_P162439	CLEC4E	NM_014358
A_24_P56833	HSD17B1	NM_000413
A_33_P322720	CDK11A	NM_024011
A_23_P12155	CRCT1	NM_019060
A_24_P8267	MED9	BC010906
A_24_P298990	CENPJ	NM_018451
A_23_P139059	QSER1	NM_001076786
A_23_P201079	PRDM2	NM_012231
A_23_P27840	SUPT5H	NM_003169
A_23_P84106	IL1RAPL1	ENST00000378993
A_32_P308022	LRRC7	NM_020794
A_23_P41487	TBC1D9	NM_015130
A_23_P433369	HEATR5A	NM_015473
A_23_P151209	CSRNP2	NM_030809
A_23_P46560	FOXD3	NM_012183
A_24_P196038	ITFG2	NM_018463
A_23_P88667	PIF1	AK026345
A_23_P154349	HIBCH	NM_014362
A_32_P229725	CDRT15	NM_001007530
A_23_P17064	CCL20	NM_004591

A_23_P86874	KCNK7	NM_033347
A_33_P33811	FAS	NM_000043
A_23_P41563	SGK494	NM_001174103
A_32_P83465	NBPF10	NM_001039703
A_23_P2537	MMAB	NM_052845
A_23_P4489	NETO1	NM_138966
A_23_P96812	POMGNT1	NM_017739
A_23_P14084	MPHOSPH6	NM_005792
A_23_P14184	THSD1	NM_018676
A_23_P25919	FOXN2	NM_002158
A_23_P25215	RECQL	NM_032941
A_33_P32897	CDRT1	NM_006382
A_23_P25202	SSX2	NM_175698
A_23_P84230	OTP	NM_032109
A_24_P69267	RUNX2	NM_004348
A_23_P11809	RPL3L	NM_005061
A_24_P92636	THRAP3	NM_005119
A_23_P16280	MRPS31	NM_005830
A_33_P33138	CELA1	NM_001971
A_23_P74716	DEDD	NM_032998
A_23_P21056	PTPN1	NM_002827
A_23_P38177	GGA3	NM_138619
A_33_P32648	RHEBL1	NM_144593
A_23_P37775	FGF10	NM_004465
A_24_P93356	PGAP1	NM_024989
A_24_P78531	CLEC4E	NM_014358
A_23_P11894	B4GALT6	NM_004775
A_23_P24987	TSPAN31	NM_005981
A_24_P91829	FEZ2	L17328
A_23_P41782	DMRTB1	NM_033067
A_32_P99432	TRAPPC5	NM_174894
A_24_P95723	KIAA0125	NR_026800
A_32_P43771	H2AFY	BC045570
A_23_P10062	MGAT4C	NM_013244
A_23_P21282	NAA15	NM_057175
A_24_P34575	POU3F3	NM_006236
A_32_P30650	ETV5	NM_004454
A_32_P20801	CKAP2L	NM_152515
A_32_P49844	RHOQ	NM_012249
A_24_P30084	CKAP5	NM_001008938
A_23_P37845	MBD3L2	NM_144614
A_23_P12816	HELLS	NM_018063
A_33_P33479	ESR2	NM_001437
A_24_P91519	C9orf91	NM_153045
A_24_P23113	ACVR2B	NM_001106
A_24_P23441	STAC	NM_003149
A_23_P13324	IK	NM_006083
A_23_P77899	CACNG4	NM_014405
A_23_P21652	RUVBL1	NM_003707

A_23_P10081	ARL13B	NM_182896
A_23_P88740	CENPN	NM_018455
A_23_P17075	POLE	NM_006231
A_23_P4536	EPB41L3	NM_012307
A_24_P83787	DISC1	NM_001012959
A_23_P89824	C18orf45	NM_032933
A_23_P14168	RAB31	NM_006868
A_23_P36934	KLK8	NM_144505
A_33_P34029	PNKP	NM_007254
A_23_P11170	GNG11	NM_004126
A_23_P41049	LZTFL1	NM_020347
A_23_P25053	IP6K1	NM_153273
A_24_P85260	HNF4G	NM_004133
A_23_P37215	C19orf29	NM_021231
A_23_P16401	SOX15	NM_006942
A_32_P13739	C14orf33	NR_027123
A_23_P77437	PRMT7	NM_019023
A_23_P32777	GATS	NM_178831
A_23_P41071	C1orf51	NM_144697
A_23_P12622	PAX7	NM_013945
A_23_P43130	FAM69B	NM_152421
A_23_P21219	SETD2	NM_014159
A_23_P10984	IL5RA	NM_000564
A_23_P12200	MSH3	NM_002439
A_23_P43178	ARF4	NM_001660
A_32_P50699	TP53INP1	NM_033285
A_23_P39729	LY6K	NM_017527
A_32_P20224	MYO16	NM_015011
A_23_P53033	MYOD1	X56677
A_32_P31647	EVPL	NM_001988
A_33_P34239	DBX2	NM_001004329
A_32_P18092	RAB3IP	NM_175623
A_23_P38181	GGA3	NM_138619
A_32_P22071	MAP1LC3B	NM_022818
A_23_P14203	RFX1	NM_002918
A_24_P56837	ASB16	NM_080863
A_23_P67101	ZIM3	NM_052882
A_24_P16782	VPS39	NM_015289
A_32_P49152	RING1	NM_002931
A_23_P13481	THAP1	NM_018105
A_24_P32361	MYO1F	NM_012335
A_33_P32879	RASA2	NM_006506
A_23_P20601	TPM1	NM_001018004
A_24_P38743	CTCFL	NM_080618
A_32_P46435	SPATS2L	NM_001100422
A_23_P24633	THYN1	NM_199297
A_23_P60354	SMARCA2	NM_139045
A_24_P22120	TRAPPC5	NM_174894
A_24_P10662	MEOX2	NM_005924

A_32_P49193	PGR	NM_000926
A_23_P30153(	ANK3	NM_020987
A_23_P11520!	CRNN	NM_016190
A_24_P14101!	ST7	NM_018412
A_32_P16114:	PRIM2	NM_000947
A_33_P33009{	KRTAP21-1	ENST00000335093
A_32_P49351:	MID2	NM_012216
A_33_P33228!	HES6	NM_018645
A_24_P38211!	MTMR4	NM_004687
A_23_P10430:	PITX3	NM_005029
A_32_P69793	SLCO1A2	NM_134431
A_32_P83935:	APOC1	AJ249921
A_23_P11727	MIPEP	NM_005932
A_23_P71727	CKS2	NM_001827
A_24_P23526(	GRB10	NM_001001555
A_33_P34137(	ERAP1	NM_001040458
A_23_P34647:	KRTAP4-3	NM_033187
A_23_P16857(	POMZP3	NM_152992
A_23_P10760	RPL17	NM_000985
A_24_P93303:	ELAVL2	ENST00000359598
A_24_P36285(	PBRM1	NM_181042
A_23_P63521	LCE2C	NM_178429
A_24_P92230(	TEF	AB051442
A_23_P14543:	PHIP	NM_017934
A_23_P21581!	ZNF655	NM_024061
A_23_P16537	ELL	NM_006532
A_32_P34599(	CRB2	NM_173689
A_23_P94762	ZNF354B	NM_058230
A_23_P14308!	MPV17	NM_002437
A_23_P14056:	TTC23	NM_001040655
A_23_P36944:	KGFLP1	NR_003674
A_32_P48705:	PAN3	NM_175854
A_23_P16595:	ACTR5	NM_024855
A_24_P18112(	PFDN5	NM_002624
A_23_P27511	OR7D2	NM_175883
A_23_P21903:	BTN3A2	NM_007047
A_23_P15016:	DRD4	NM_000797
A_23_P71073	TWIST1	NM_000474
A_23_P20990	GPC1	NM_002081
A_24_P14311{	SCML2	NM_006089
A_33_P37091!	T	NM_003181
A_32_P41065	TMCC1	NM_001017395
A_23_P10010:	VPS39	NM_015289
A_33_P32213(	CCR10	NM_016602
A_23_P14124	RASL11A	NM_206827
A_23_P31234	RBM19	NM_016196
A_33_P38511:	SCRT1	NM_031309
A_23_P92362	NDUFC1	NM_002494
A_33_P32735!	KRT83	NM_002282

A_33_P33566	ZNF192	NM_006298
A_24_P25354	TACC1	NM_006283
A_23_P25654	FAM162A	NM_014367
A_23_P65532	PELI2	NM_021255
A_33_P33688	GPC5	NM_004466
A_32_P79366	HABP4	NM_014282
A_23_P21080	CSTL1	NM_138283
A_23_P87363	ART1	NM_004314
A_33_P32860	RP11-529I10.	NM_015448
A_32_P67259	SDHA	NM_004168
A_23_P16903	SNAI2	NM_003068
A_33_P33925	LBX2	NM_001009812
A_23_P79860	KCNB1	NM_004975
A_23_P14554	FIG4	NM_014845
A_33_P34162	HOXA9	NM_152739
A_32_P11350	ATG16L1	NM_030803
A_32_P38093	ATOH8	NM_032827
A_23_P96091	NTF3	NM_002527
A_23_P16840	KCNH2	NM_000238
A_32_P77480	ARID3B	NM_006465
A_24_P17086	TGFBRAP1	ENST00000393359
A_32_P42663	DMRTA1	NM_022160
A_32_P86905	KGFLP2	NR_003670
A_24_P39436	WDR19	NM_025132
A_23_P12797	B3GNT6	NM_138706
A_32_P12930	ZWILCH	NM_017975
A_23_P14056	TLE3	NM_005078
A_23_P11973	ZFP30	NM_014898
A_23_P31838	EEF1D	NM_032378
A_23_P91479	RWDD2B	NM_016940
A_23_P11413	SSX3	NM_175711
A_23_P14951	PIGR	NM_002644
A_24_P22200	RIMS2	NM_014677
A_23_P51187	PRKCZ	NM_002744
A_24_P94225	PHF8	NM_015107
A_32_P35196	HLA-DMB	NM_002118
A_24_P93041	RBPMS	AK057533
A_23_P11199	LOXL2	NM_002318
A_24_P18262	CELSR2	NM_001408
A_33_P33252	SLC6A8	NM_005629
A_23_P17063	KCNH8	NM_144633
A_33_P33428	FAM101A	NM_181709
A_23_P25819	AIFM3	NM_001018060
A_23_P16993	RILPL1	NM_178314
A_23_P74901	ESRRG	NM_206594
A_32_P12673	NOL4	NM_003787
A_23_P20285	PDLIM2	NM_021630
A_23_P13176	HINFP	NM_015517
A_23_P86222	LYPLAL1	NM_138794

A_32_P38629	ASXL3	NM_030632
A_24_P24705	TAF1C	NM_005679
A_24_P40341	PTGES	NM_004878
A_23_P96451	CA5B	NM_007220
A_32_P16950	KIAA2022	NM_001008537
A_23_P38928	HOXA13	NM_000522
A_32_P10363	MCM2	NM_004526
A_32_P21118	PPARGC1B	NM_133263
A_23_P33424	APLF	NM_173545
A_23_P42193	ATXN7L3	NM_020218
A_23_P25391	CHRNA3	NM_000743
A_23_P11800	CAMK2N1	NM_018584
A_24_P92990	POU1F1	NM_001122757
A_24_P94384	NDNL2	AK074138
A_23_P68978	EFCAB6	NM_022785
A_23_P10407	S100A3	NM_002960
A_32_P70724	KDM5B	NM_006618
A_24_P34080	ZNF621	NM_198484
A_23_P21551	KLHL7	NM_001172428
A_23_P82336	SLC25A13	NM_014251
A_23_P12461	S100A14	NM_020672
A_33_P33467	SCAMP3	NM_052837
A_24_P20136	LDB3	NM_001171611
A_24_P41908	AVIL	NM_006576
A_23_P10695	MYO15B	NR_003587
A_32_P35594	RGSL1	NM_001137669
A_24_P90250	FANCI	NM_018193
A_32_P18692	ZNF616	BC032805
A_23_P16225	DENR	NM_003677
A_33_P32904	ZNF648	NM_001009992
A_24_P10908	USP37	NM_020935
A_33_P32694	FOXI1	NM_012188
A_33_P32819	RPE	NM_006916
A_33_P32859	NEUROD1	NM_002500
A_24_P57308	SH2D5	NM_001103161
A_24_P66350	KIAA2022	NM_001008537
A_23_P16706	UGDH	NM_003359
A_24_P21762	PRDM9	NM_020227
A_24_P37628	LRRC8A	NM_019594
A_32_P1701	POLA1	NM_016937
A_23_P16900	SH2D4A	NM_022071
A_24_P92704	PRND	NM_012409
A_33_P33348	KRTAP10-4	NM_198687
A_23_P25565	TNFRSF10A	NM_003844
A_24_P36288	IFT57	NM_018010
A_33_P33386	IHH	NM_002181
A_24_P21286	C9orf156	NM_016481
A_23_P21325	SMARCAD1	NM_020159
A_23_P21612	PPAPDC1B	NM_001102559

A_33_P33510[illegible]	KRTAP20-2	NM_181616
A_23_P11283	HNRNPH2	NM_019597
A_23_P64721	GPR109B	NM_006018
A_23_P60028	TCEB1	NM_005648
A_23_P14399[illegible]	ATG7	NM_006395
A_23_P14066[illegible]	IDH3A	NM_005530
A_33_P32313[illegible]	KISS1R	NM_032551
A_23_P14462[illegible]	GNPDA1	NM_005471
A_32_P16973[illegible]	TTC8	NM_144596
A_32_P50840[illegible]	SALL3	NM_171999
A_24_P5935	UBE2V1	NM_001032288
A_24_P38387	NDRG1	NM_006096
A_23_P3169	GMPR2	NM_001002000
A_24_P30352[illegible]	MICALL2	NM_182924
A_24_P46130	ACPP	NM_001099
A_33_P34035[illegible]	TMEM120B	NM_001080825
A_32_P96435	ZNF829	NM_001037232
A_23_P25917[illegible]	SSR4	NM_006280
A_32_P74703[illegible]	ZNF850	NM_001193552
A_23_P11036[illegible]	MAPKSP1	NM_021970
A_24_P40284[illegible]	CLCN3	NM_001829
A_23_P47967	LHX5	NM_022363
A_23_P20188[illegible]	RBBP5	NM_005057
A_23_P16263[illegible]	OLR1	NM_002543
A_33_P32127[illegible]	CALM2	NM_001743
A_33_P32779[illegible]	NFIC	NM_205843
A_23_P41853[illegible]	GRB2	NM_002086
A_33_P33182[illegible]	PPP2R5B	NM_006244
A_23_P56163	PTPRS	NM_002850
A_32_P10776	LEO1	NM_138792
A_23_P37225[illegible]	ITPKB	NM_002221
A_24_P32649[illegible]	MKX	NM_173576
A_32_P17573[illegible]	HK2	NM_000189
A_33_P32200[illegible]	ZNF391	NM_001076781
A_24_P30609[illegible]	YLPM1	L40403
A_32_P69802	SLCO1A2	NM_134431
A_23_P48175	TMEM106C	NM_024056
A_23_P12639[illegible]	SETDB1	NM_012432
A_23_P35489[illegible]	ZNF567	NM_152603
A_24_P15628[illegible]	GZF1	NM_022482
A_33_P32639[illegible]	LCN10	NM_001001712
A_23_P60002	TTC35	NM_014673
A_23_P89812	CNDP2	NM_018235
A_23_P82334	SLC25A13	NM_014251
A_32_P32681[illegible]	KRR1	NM_007043
A_32_P31238[illegible]	HKR1	NM_181786
A_23_P43500[illegible]	SRFBP1	NM_152546
A_33_P33818[illegible]	KRTAP10-10	NM_181688
A_23_P8252	RAET1E	NM_139165

A_23_P14287	TCF7L1	NM_031283
A_23_P38625	NKX3-2	NM_001189
A_23_P11885	KRT37	NM_003770
A_23_P32510	DMRT3	NM_021240
A_23_P25769	GTF2IRD1	NM_005685
A_23_P87564	BTG1	NM_001731
A_23_P24004	IFIT2	NM_001547
A_23_P96611	LUZP4	NM_016383
A_32_P21474	IRAK1BP1	NM_001010844
A_32_P39387	DERL2	ENST00000158771
A_24_P13977	PPP1R8	NM_138558
A_23_P43549	SERINC3	NM_198941
A_23_P14974	GPR88	AB042410
A_33_P33825	WNT1	NM_005430
A_23_P34037	MST1P9	NR_002729
A_23_P13826	PADI4	NM_012387
A_32_P16004	TCTEX1D1	NM_152665
A_23_P39122	MANEAL	NM_001031740
A_23_P33418	MEF2D	NM_005920
A_23_P22941	LUZP1	NM_033631
A_24_P11050	DBX2	NM_001004329
A_24_P10729	PPP2R1B	NM_181699
A_32_P14070	PMS2	NM_000535
A_33_P33112	KRTAP19-6	NM_181612
A_32_P73805	RASGRF1	NM_002891
A_33_P32434	CD70	NM_001252
A_23_P39139	EBF3	NM_001005463
A_24_P76879	TSPYL1	NM_003309
A_33_P33905	GYLTL1B	NM_152312
A_23_P14105	TGFB1I1	NM_001042454
A_23_P11297	TNFRSF4	NM_003327
A_24_P30778	RPAIN	AK098491
A_24_P21350	PTPRE	NM_006504
A_33_P36406	ZEB1	NM_001128128
A_32_P70126	MED12L	NM_053002
A_24_P22723	CADM1	NM_014333
A_33_P33162	CAB39	NM_001130849
A_23_P33420	KLF7	NM_003709
A_23_P34260	STK35	NM_080836
A_23_P10077	UBTF	NM_001076683
A_23_P15242	KIAA0182	NM_014615
A_24_P91628	YY1	ENST00000262238
A_32_P10130	USP39	NM_006590
A_23_P34147	UBE2N	NM_003348
A_33_P32389	TGIF2LY	NM_139214
A_24_P54148	HAB1	X83412
A_33_P33696	CCDC94	NM_018074
A_23_P5301	TFCP2L1	NM_014553
A_23_P20186	PCTK3	NM_212503

A_23_P49928	KRT13	NM_002274
A_23_P21796	SUV420H1	NM_016028
A_23_P35146	CMAH	NR_002174
A_23_P24883	ST5	NM_005418
A_24_P14127	GRHPR	NM_012203
A_24_P41561	CHRM1	NM_000738
A_23_P47386	GRAMD1B	AB033027
A_23_P10636	AQP9	NM_020980
A_24_P10629	AMACR	NM_014324
A_32_P22296	SPIN4	NM_001012968
A_24_P62685	H6PD	NM_004285
A_33_P33488	PBX3	NM_006195
A_32_P10756	RFC5	NM_181578
A_32_P78491	ETV1	NM_004956
A_23_P76901	PLEKHG3	NM_015549
A_33_P33751	C9orf150	NM_203403
A_23_P16572	MSTN	NM_005259
A_23_P10901	SFRS6	NM_006275
A_32_P44342	ZNF18	NM_144680
A_23_P16896	C8orf44	NM_019607
A_32_P37422	ZNF782	NM_001001662
A_23_P80503	ROBO1	NM_133631
A_24_P30906	GBX1	NM_001098834
A_33_P33901	ADAMDEC1	NM_001145271
A_23_P14932	C1orf183	NM_019099
A_23_P11711	IL22	NM_020525
A_33_P32579	GSTA4	NM_001512
A_23_P20474	SMARCD1	NM_139071
A_33_P33877	PHLDB1	NM_015157
A_23_P50108	ZNF92	NM_007139
A_32_P65805	GPR65	NM_003608
A_23_P79661	CCDC93	NM_019044
A_23_P37892	GPT2	NM_133443
A_33_P33775	HOXA1	NM_005522
A_23_P20357	RPL27A	NM_000990
A_33_P33682	ABRA	NM_139166
A_23_P23457	FBLIM1	NM_001024215
A_23_P42846	ZNF292	NM_015021
A_23_P38526	CHD5	NM_015557
A_23_P41750	SPZ1	NM_032567
A_23_P30395	GPBP1	NM_022913
A_24_P38895	H2AFX	NM_002105
A_33_P33798	SMYD1	NM_198274
A_24_P40224	COL3A1	NM_000090
A_23_P40602	PRUNE2	NM_015225
A_32_P12857	MIER1	NM_001077702
A_33_P34242	DLEU2L	NR_002771
A_23_P42969	FGL2	NM_006682
A_23_P41971	BTBD11	NM_001018072

A_23_P63459	C1orf31	NM_001012985
A_23_P15210	UBE2I	NM_194259
A_23_P21436	IRF4	NM_002460
A_23_P12138	IFT57	NM_018010
A_23_P1307	AKR1C1	NM_001353
A_23_P36464	C12orf11	NM_018164
A_23_P2143	SPCS2	NM_014752
A_23_P32746	RFX6	NM_173560
A_33_P32156	PI16	NM_153370
A_23_P1225	KAZALD1	NM_030929
A_23_P20238	SORBS1	NM_001034954
A_24_P19037	ZNF302	NM_018443
A_23_P9932	PDCD4	NM_145341
A_23_P60675	NDUFA5	NM_005000
A_23_P99292	RAD51AP1	NM_006479
A_23_P34863	FOXJ1	NM_001454
A_23_P20136	CTBS	NM_004388
A_23_P37141	PRKACB	NM_207578
A_23_P67228	SNAPC2	NM_003083
A_23_P19084	HNRNPAB	NM_004499
A_23_P25239	POU6F1	NM_002702
A_23_P94636	RC3H2	NM_018835
A_23_P16633	TMEM191A	NR_026815
A_33_P32370	INPP5F	NR_003251
A_23_P14186	ZNF134	ENST00000396161
A_23_P34380	SOS1	NM_005633
A_33_P33320	FGL2	NM_006682
A_23_P21393	C5orf4	NM_032385
A_24_P36340	HEY2	NM_012259
A_23_P56949	SERTAD2	NM_014755
A_24_P29022	HS3ST4	NM_006040
A_23_P82068	NOL7	NM_016167
A_32_P79941	LOC150786	NM_001077637
A_23_P11909	PPP1R13L	NM_006663
A_23_P11791	PLA2G4E	NM_001080490
A_33_P32285	EPB41L1	NM_177996
A_23_P85952	DENND2D	NM_024901
A_33_P32201	TRIM15	NM_033229
A_23_P36939	NUFIP1	NM_012345
A_23_P20981	PASK	NM_015148
A_24_P34192	KIAA1632	NM_020964
A_23_P34642	ZNF532	NM_018181
A_33_P33213	INSIG2	NM_016133
A_23_P40587	C12orf54	NM_152319
A_23_P44360	CASKIN2	NM_020753
A_23_P13885	DRAP1	NM_006442
A_23_P81859	HIST1H2AH	NM_080596
A_32_P95960	CLEC2D	NM_001004419
A_32_P77977	UTP11L	NM_016037

A_33_P326285	LIMS3	NR_027467
A_23_P213959	PPARGC1B	NM_133263
A_33_P336307	ITIH5L	NM_198510
A_24_P294851	TRIM38	NM_006355
A_33_P321320	MAGEF1	NM_022149
A_24_P932416	TMEM14E	NM_001123228
A_23_P66473	PITPNC1	NM_181671
A_33_P338005	C9orf30	NM_001198807
A_33_P333014	PAX6	NM_000280
A_23_P16583	KIRREL2	NM_032123
A_23_P93663	ZNF783	NM_001195220
A_24_P827	SUPT3H	NM_003599
A_32_P397426	KRTAP13-2	NM_181621
A_23_P155939	ZNF595	NM_182524
A_33_P326530	C20orf4	CR590998
A_33_P334965	SWAP70	NM_015055
A_23_P77623	ST3GAL2	NM_006927
A_23_P32715	ZNF408	NM_024741
A_24_P339059	CRISPLD2	NM_031476
A_23_P215677	C7orf44	NM_018224
A_23_P383132	HIC2	NM_015094
A_33_P326753	KCNJ15	NM_170736
A_24_P218259	FBXO32	ENST00000517956
A_24_P336577	C1orf183	NM_019099
A_23_P5875	POMC	NM_001035256
A_32_P401323	AFF3	NM_002285
A_33_P337752	HOXA4	NM_002141
A_24_P283341	MICAL1	NM_022765
A_23_P165180	RFXANK	NM_003721
A_32_P372878	SLCO5A1	AK022971
A_23_P367995	PSMA8	NM_144662
A_32_P172849	GK	NM_203391
A_23_P256455	RPA3	NM_002947
A_23_P500390	DONSON	NM_017613
A_23_P106512	PLA2G4F	NM_213600
A_24_P928038	IDE	NM_004969
A_23_P23159	AJAP1	NM_018836
A_32_P149640	EPHA5	NM_004439
A_23_P250313	KIAA1524	NM_020890
A_33_P332113	DENND3	NM_014957
A_33_P335439	KRTAP12-3	NM_198697
A_23_P38537	KRT16	NM_005557
A_24_P123245	HNRNPD	NM_031370
A_24_P398572	IGF1	NM_000618
A_33_P333442	SPRR2G	NM_001014291
A_32_P160127	EBF3	NM_001005463
A_24_P296254	ARHGAP11A	NM_014783
A_23_P159937	SLC6A8	NM_005629
A_23_P432360	PMM2	NM_000303

A_23_P21271!	CBLB	NM_170662
A_24_P18570!	EPB41L1	NM_012156
A_23_P78281	GNA13	NM_006572
A_32_P52990!	EIF3C	NM_001037808
A_23_P34644	FCGR2B	NM_004001
A_24_P27757(	TRIP13	NM_004237
A_33_P34221:	ADAP1	NM_006869
A_23_P20347!	PRKCDBP	NM_145040
A_23_P99216	POP5	NM_015918
A_33_P33453!	DRGX	NM_001080520
A_23_P14462:	GNPDA1	NM_005471
A_24_P21616!	CEBPA	NM_004364
A_33_P32462(	HOXD13	NM_000523
A_23_P11743:	IL25	NM_022789
A_32_P50247:	NT5DC4	XM_001715677
A_24_P14040!	ADAMTS3	NM_014243
A_23_P86632	DCLRE1C	NM_001033858
A_24_P93887	MED29	NM_017592
A_23_P36645:	KHDRBS2	NM_152688
A_32_P89924	CKS1B	NM_001826
A_23_P15419!	PPP1R7	NM_002712
A_23_P67312	ZNF136	NM_003437
A_23_P95527	PCTK2	NM_002595
A_32_P52109(	SNX6	ENST00000396526
A_23_P34379!	SOX6	NM_017508
A_23_P11586:	ZNF485	NM_145312
A_23_P36182(	ATG2A	NM_015104
A_33_P32310!	TCEA2	BC098585
A_33_P33941!	FPGS	NM_004957
A_23_P21831:	NARF	NM_001038618
A_23_P40724	PPARA	NM_005036
A_32_P13589(	LOC401233	AK128391
A_32_P39375:	SMURF2	CR936721
A_32_P30637!	LOC401233	NR_033884
A_32_P11900:	ESPL1	NM_012291
A_33_P32314	ITGA6	NM_000210
A_23_P15632:	TGFBI	NM_000358
A_24_P4054	TRIP6	NM_003302
A_23_P25038!	HIST1H1B	NM_005322
A_23_P42873!	ANG	NM_001145
A_23_P30568(	IGF1R	AF020763
A_23_P85201	PLP1	BC002665
A_23_P42091:	METT5D1	NM_152636
A_24_P26240:	THRA	NM_199334
A_23_P99360	TRIM13	NM_213590
A_32_P32072:	IQCK	NM_153208
A_23_P14497(	PIK3R1	NM_181523
A_24_P14660:	DDX21	NM_004728
A_24_P25329:	NKX2-8	NM_014360

A_23_P11150	SAP25	NM_001168682
A_33_P33405	ZNF385C	ENST00000393873
A_24_P34344	TRIM33	NM_033020
A_32_P44894	TSPYL1	NM_003309
A_23_P50073	POGZ	NM_015100
A_23_P10794	ZNF473	NM_015428
A_23_P39654	TADA2B	NM_152293
A_24_P13799	ZNF34	NM_030580
A_23_P42575	CALD1	NM_033138
A_23_P89801	ACAA2	NM_006111
A_23_P78795	MEIS3	NM_001009813
A_23_P92672	OCLN	NM_002538
A_23_P60259	TMEM38B	NM_018112
A_24_P20305	BCL7A	NM_020993
A_23_P31828	GPD1L	NM_015141
A_23_P11994	IGFBP2	NM_000597
A_23_P14848	RLIM	NM_016120
A_23_P25578	NCK1	NM_006153
A_23_P33977	TPRG1L	NM_182752
A_24_P89891	TRAF1	NM_005658
A_24_P21184	TBX20	NM_001166220
A_23_P11862	C1orf112	NM_018186
A_33_P34086	IQSEC3	NM_015232
A_23_P37775	FGF10	NM_004465
A_32_P64570	KANK1	NM_153186
A_23_P21902	MED20	NM_004275
A_32_P71627	ZNF705D	NM_001039615
A_24_P22571	MOBKL3	NM_015387
A_23_P16509	NDUFS7	NM_024407
A_24_P36536	TCF3	NM_003200
A_32_P47988	CAMK2D	NM_001221
A_23_P32325	TNKS	NM_003747
A_23_P59481	UBE3C	NM_014671
A_24_P38073	SDC2	NM_002998
A_23_P77000	VASH1	NM_014909
A_23_P20183	MR1	ENST00000367580
A_33_P32996	RPGR	NM_000328
A_33_P33192	ZNF705G	NM_001164457
A_23_P32647	L3MBTL4	NM_173464
A_23_P41218	ZNF252	NR_023392
A_23_P39608	AASDH	BC015096
A_23_P80662	SOX14	NM_004189
A_24_P17268	AQP2	NM_000486
A_23_P41096	KIAA1522	NM_020888
A_23_P13884	KAT5	NM_006388
A_23_P45771	NHLH1	NM_005598
A_24_P12546	LIPG	NM_006033
A_33_P33535	ZBTB8B	NM_001145720
A_23_P22200	ASB8	NM_024095

A_32_P47285	SHH	ENST00000297261
A_23_P9415	ACO1	NM_002197
A_32_P10633	TBC1D22A	NM_014346
A_24_P21995	CRTC1	NM_001098482
A_33_P34082	TGFA	NM_003236
A_24_P28295	RABGAP1L	ENST00000325594
A_33_P34052	PECAM1	NM_000442
A_23_P8663	DMTF1	NM_021145
A_23_P31545	KIRREL2	NM_199180
A_33_P33654	LHX9	NM_020204
A_33_P34076	KRTAP22-1	ENST00000334680
A_33_P34050	NFXL1	NM_152995
A_33_P37190	CHRFAM7A	NM_139320
A_23_P37503	MYO1E	NM_004998
A_23_P55706	RELB	NM_006509
A_23_P21698	RAB14	NM_016322
A_23_P14651	GOLM1	NM_016548
A_23_P7963	PGC	NM_002630
A_23_P97892	LDB1	NM_003893
A_33_P33980	TPD52L1	NM_001003397
A_24_P28562	FMO4	NM_002022
A_23_P31241	ADAMTSL1	NM_052866
A_23_P35352	IVL	NM_005547
A_23_P21200	NKTR	NM_005385
A_24_P34545	ING5	NM_032329
A_24_P20069	RING1	NM_002931
A_23_P11118	ZBTB22	NM_005453
A_24_P40998	TMEM44	NM_001011655
A_24_P37442	ZDHHC21	NM_178566
A_23_P63219	POGZ	NM_015100
A_32_P51726	SERPINB4	NM_002974
A_24_P11280	SOX1	NM_005986
A_24_P32399	TPRX1	NM_198479
A_33_P33610	TFIP11	NM_001008697
A_23_P13813	OMA1	NM_145243
A_23_P15287	RAB34	NM_031934
A_32_P90069	S100A4	NM_002961
A_23_P13412	MAP3K5	NM_005923
A_23_P42550	DONSON	NM_017613
A_32_P35319	LOC90925	XR_078810
A_23_P25202	SSX3	NM_021014
A_33_P32535	MED20	NM_004275
A_33_P33320	KHDRBS3	NM_006558
A_33_P33215	BAZ1B	NM_032408
A_24_P76952	LIN28B	NM_001004317
A_24_P21807	ZNF467	NM_207336
A_23_P34930	BCAS2	NM_005872
A_23_P31217	ALMS1	NM_015120
A_32_P37005	LHX8	NM_001001933

A_32_P41619:	OR2W5	NM_001004698
A_23_P48550	KIAA0284	NM_015005
A_32_P87940!	IKBKAP	NM_003640
A_32_P14915{	PLCL1	NM_006226
A_23_P15997.	KLHL13	NM_033495
A_24_P44845	GRLF1	NM_004491
A_23_P12101:	CSRNP1	NM_033027
A_23_P77360	CIAO1	NM_004804
A_24_P22603:	HNRNPD	NM_002138
A_23_P69970	SOX30	NM_178424
A_32_P22285:	RBMX	NM_002139
A_24_P25547:	SYT9	NM_175733
A_23_P41982{	EVI5L	NM_145245
A_24_P87341.	PLEKHB2	NM_001100623
A_23_P4490	NETO1	NM_138966
A_23_P34873:	NR2F1	NM_005654
A_33_P33471(	TBL1X	NM_005647
A_23_P41643.	PHF15	NM_015288
A_23_P11959	TGFB2	NM_003238
A_24_P28861:	DMC1	NM_007068
A_23_P32374!	PIF1	NM_025049
A_23_P59855	ZNF138	NM_006524
A_32_P20205:	YWHAQ	NM_006826
A_24_P14464	WFDC2	NM_006103
A_33_P32538(	CEBPD	NM_005195
A_23_P10918.	INSM1	NM_002196
A_24_P15114	USP37	NM_020935
A_24_P3249	RARB	NM_000965
A_24_P34975(	LOC375196	NR_028386
A_23_P42764!	TLL1	NM_012464
A_33_P32934.	KIAA1462	NM_020848
A_23_P16523	GDF15	NM_004864
A_23_P13164(	RPIA	NM_144563
A_32_P50043	RPL6	NM_001024662
A_23_P18465	RFC1	NM_002913
A_23_P43540:	GPC4	NM_001448
A_24_P30299{	ATP5I	NM_007100
A_32_P21170{	FZD3	NM_017412
A_23_P49192	CES2	NM_198061
A_32_P11319:	ZNF592	NM_014630
A_32_P19990!	RIPPLY1	NM_138382
A_23_P20289.	ACRV1	NM_001612
A_23_P12339:	KCNQ3	NM_004519
A_23_P67103	ZIM3	NM_052882
A_24_P31367{	MED31	NM_016060
A_23_P11063:	IL5	NM_000879
A_23_P11012:	CCNG2	NM_004354
A_33_P34338:	ZWILCH	NM_017975
A_23_P15825:	DUX3	NM_012148

A_23_P53397	SP1	NM_138473
A_23_P25893	ZNF623	NM_014789
A_33_P32273	FAM123B	NM_152424
A_23_P10788	ZNF563	NM_145276
A_23_P8416	GALNT11	NM_022087
A_23_P11069	ZNF300	NM_052860
A_23_P34572	HOXD4	NM_014621
A_32_P14187	TFAP2A	NM_001032280
A_24_P70480	CEACAM4	NM_001817
A_23_P10350	MDM4	NM_002393
A_32_P3705	ZNF740	NM_001004304
A_23_P33482	UVRAG	NM_003369
A_24_P39610	IP6K1	NM_153273
A_24_P97687	HTR1A	NM_000524
A_24_P85850	BNIPL	NM_138278
A_24_P151	KCNAB2	NM_003636
A_23_P37088	RDH12	NM_152443
A_24_P91272	CHRNA4	X89741
A_24_P49298	FAM149B1	ENST00000242505
A_23_P11755	FKBP3	NM_002013
A_24_P21736	ANKRD28	NM_015199
A_23_P78778	CA11	NM_001217
A_23_P40354	PITPNM2	NM_020845
A_24_P20235	PNPO	NM_018129
A_23_P14564	GPR65	NM_003608
A_23_P30446	GATA1	NM_002049
A_23_P34761	ERO1LB	NM_019891
A_23_P32807	SALL1	NM_002968
A_23_P52031	PGM1	NM_002633
A_23_P13963	GNS	NM_002076
A_23_P37547	ZADH2	NM_175907
A_23_P49862	TRPV3	NM_145068
A_23_P14114	QRICH2	NM_032134
A_24_P18803	HIF3A	AK024095
A_24_P21418	LOC51152	AF172850
A_23_P88538	NTRK3	NM_002530
A_32_P75334	FOS	NM_005252
A_24_P94034	USP22	NM_015276
A_23_P51669	MSTO1	NM_018116
A_23_P78444	ELP2	NM_018255
A_23_P88691	CHRNA5	NM_000745
A_23_P20408	OAS2	NM_016817
A_23_P35856	POU5F2	NM_153216
A_33_P32090	ZNF623	NM_001082480
A_23_P14163	KRTAP1-1	NM_030967
A_23_P38506	DNAJB6	NM_058246
A_23_P21966	NSD1	NM_022455
A_24_P24363	DCBLD1	NM_173674
A_33_P32953	ANGPTL4	NM_139314

A_32_P71447	NCAPD3	NM_015261
A_23_P16313(	FCF1	NM_015962
A_23_P45234	ZNF41	NM_153380
A_32_P91109(	EMX2	NM_004098
A_24_P31380(	ZNF587	NM_032828
A_23_P21300(	WDR1	NM_017491
A_23_P15932(	ANGPTL4	NM_139314
A_24_P17924(	LOC10012897(	XR_109205
A_33_P34093(	FZD6	NM_003506
A_23_P4626	ZNF606	NM_025027
A_32_P15738(	C20orf199	CR936805
A_24_P26466(	CHD2	NM_001271
A_32_P30551(	ZNF619	NM_001145083
A_23_P12244(	HIST1H1C	NM_005319
A_24_P78794(	YPEL2	NM_001005404
A_23_P14886(	SLC2A7	NM_207420
A_24_P10041(	EDA	NM_001005612
A_23_P118	EXTL2	NM_001439
A_24_P25323(	PSPC1	NM_001042414
A_23_P4387	KRT24	NM_019016
A_24_P13962(	USP21	NM_001014443
A_24_P39672(	PPP1CB	NM_002709
A_33_P33321(	FAS	NM_000043
A_23_P30044	SLC30A9	NM_006345
A_24_P30758(	HTATIP2	NM_001098523
A_32_P13524(	MTHFD1L	NM_015440
A_23_P41172(	PLAG1	NM_002655
A_33_P33881(	CHRNA3	NM_001166694
A_32_P30928(	WWC1	NM_015238
A_24_P91150(	MED13L	NM_015335
A_24_P31251(	PBLD	NM_001033083
A_23_P66858	KRTAP17-1	NM_031964
A_24_P22477(	LSM4	NM_012321
A_23_P39185	RDH13	NM_138412
A_23_P58675	TPPP	NM_007030
A_23_P14369(	SH3BP1	NM_018957
A_23_P10337(	ADC	NM_052998
A_23_P15902(	ZNF521	NM_015461
A_23_P65518	DACT1	NM_016651
A_24_P38409(	ZNF775	NM_173680
A_32_P19976(	ZNF630	NM_001037735
A_33_P32796(	UCN2	NM_033199
A_33_P33322(	MUC1	NM_001044392
A_33_P32758(	DES	NM_001927
A_23_P14935	TXNDC11	NM_015914
A_24_P18778(	SETD2	NM_014159
A_23_P78835	ZNF787	NM_001002836
A_32_P12615(	ST6GAL2	NM_032528
A_23_P50773	CRTC1	NM_001098482

A_23_P30576	PHF12	NM_020889
A_32_P41885	MEIS3P1	NR_002211
A_24_P36273	FOXP1	NM_032682
A_33_P32895	EFR3B	NM_014971
A_24_P12287	TRAK2	NM_015049
A_23_P39038	ZNF630	NM_001037735
A_23_P16000	UTY	NM_182660
A_23_P20520	DHRS12	NM_024705
A_32_P17436	SATB2	NM_015265
A_24_P18114	BBS10	NM_024685
A_32_P21255	SLC30A4	NM_013309
A_23_P12757	PTS	NM_000317
A_24_P41690	UPF3A	NM_023011
A_24_P93676	TRIM25	NM_005082
A_24_P40675	LOXL4	NM_032211
A_32_P59158	DMRTB1	NM_033067
A_33_P33174	NDUFV1	NM_007103
A_24_P29574	HSPA8	NM_153201
A_33_P32920	BPIL2	BC062656
A_33_P33270	ARHGEF2	NM_001162383
A_33_P33950	ACSM5	NM_017888
A_23_P40217	DOK5	NM_018431
A_24_P40721	SPTLC3	NM_018327
A_23_P11534	LGTN	NM_006893
A_23_P40192	CDH22	NM_021248
A_23_P15356	IGFL2	NM_001002915
A_23_P12489	KISS1	NM_002256
A_23_P16609	ZNF506	ENST00000443905
A_23_P2543	CUX2	NM_015267
A_24_P70937	LOC654433	NR_015377
A_23_P10036	NUPR1	NM_001042483
A_24_P39809	RAD23B	NM_002874
A_23_P54297	EIF3J	NM_003758
A_23_P13019	PYCR1	NM_006907
A_24_P88690	SLC11A1	NM_000578
A_23_P20804	ZNF24	NM_006965
A_32_P10355	STON2	BX648249
A_23_P12235	FOXQ1	NM_033260
A_24_P32887	BRD3	NM_007371
A_23_P20545	SIVA1	NM_006427
A_24_P30765	SLC6A15	NM_182767
A_24_P48403	YES1	NM_005433
A_33_P33343	CA6	NM_001215
A_24_P36786	USP36	BC038983
A_33_P34140	MEN1	NM_130803
A_32_P47591	KDM4B	NM_015015
A_24_P92314	ZC3HAV1L	AK098156
A_23_P38697	TFAP2D	NM_172238
A_23_P21217	HRH1	NM_000861

A_23_P21420	CNR1	NM_033181
A_23_P80507	ROBO1	NM_133631
A_32_P10843	ZBTB8A	NM_001040441
A_23_P13654	PIGG	NM_017733
A_32_P77954	LCE1B	NM_178349
A_24_P94419	CEP68	NM_015147
A_33_P33934	KRTAP5-5	NM_001001480
A_24_P25408	ZNF69	NM_021915
A_24_P30859	ABHD10	NM_018394
A_23_P10127	ZNF446	NM_017908
A_23_P14511	GCLC	NM_001498
A_23_P13712	SSX1	NM_005635
A_23_P37369	TCTN3	NM_015631
A_23_P15734	KRT9	NM_000226
A_24_P30305	PPARGC1A	NM_013261
A_23_P62399	TCEAL5	NM_001012979
A_33_P33009	HOXC4	NM_014620
A_24_P27379	ZNF641	NM_152320
A_33_P33199	SCML4	NM_198081
A_32_P90477	KRTAP10-11	NM_198692
A_23_P21630	RUNX1T1	NM_004349
A_23_P42087	NR1D1	NM_021724
A_33_P34047	SPN	NM_001030288
A_23_P13770	TBX19	NM_005149
A_32_P96000	ETV3L	NM_001004341
A_23_P38404	CNIH3	NM_152495
A_32_P83442	FAF1	NM_007051
A_33_P34745	ZNF90	NM_007138
A_23_P21811	SERPINA1	NM_001002236
A_24_P28367	COPA	NM_004371
A_23_P35018	TET2	NM_001127208
A_23_P14333	BMP2	NM_001200
A_23_P21323	CBR4	NM_032783
A_33_P32426	SLC22A11	NM_018484
A_23_P14170	TCEB3B	NM_016427
A_23_P25348	AADAT	NM_016228
A_23_P76538	TESC	NM_017899
A_23_P44636	ANAPC7	NM_016238
A_23_P14574	CPVL	NM_019029
A_23_P10690	PPL	NM_002705
A_24_P33966	NCLN	NM_020170
A_23_P20482	NR2C1	NM_001032287
A_23_P16835	HEY2	NM_012259
A_23_P21242	XYLB	NM_005108
A_24_P38180	UBE3A	NM_130839
A_24_P93275	UNQ353	AY358648
A_23_P16104	KLHL12	NM_021633
A_23_P16930	IFNA17	NM_021268
A_32_P83050	TRAK1	NM_001042646

A_24_P84068	MLL3	NM_170606
A_24_P22132	ZBTB38	NM_001080412
A_24_P4170	CPA6	NM_020361
A_23_P37058	HOXB8	NM_024016
A_24_P34513	LRRC8B	NM_015350
A_23_P10251	PDE6D	NM_002601
A_33_P32544	LOC10012800	NR_024445
A_33_P33598	NKX2-4	NM_033176
A_23_P9232	GCNT1	NM_001490
A_33_P32650	ST5	NM_005418
A_32_P35121	MKL2	NM_014048
A_23_P14602	FOXK1	NM_001037165
A_23_P14839	MAGEA5	NM_021049
A_23_P85777	GNPAT	NM_014236
A_23_P11996	CEBPZ	NM_005760
A_33_P32097	POU4F1	NM_006237
A_33_P33828	DCN	NM_133507
A_23_P16144	GTF2F1	NM_002096
A_24_P25407	ICAM5	NM_003259
A_24_P30319	ARL15	NM_019087
A_23_P76823	ADSSL1	NM_199165
A_23_P14011	MARK3	NM_002376
A_23_P17242	ABHD1	NM_032604
A_24_P37941	IL6R	NM_000565
A_23_P13793	MNDA	NM_002432
A_23_P33583	PRSS2	NM_002770
A_23_P21715	ZNF673	NM_017776
A_23_P31212	ZNF746	NM_152557
A_32_P17284	GK	NM_000167
A_32_P7923	BMP5	NM_021073
A_23_P4353	WSB1	NM_015626
A_32_P90113	GLRA3	ENST00000274093
A_23_P25142	CDCA7	NM_031942
A_33_P32578	FANCD2	NM_001018115
A_23_P6045	DEFB126	NM_030931
A_33_P33426	HES4	NM_021170
A_23_P41504	ARL8A	NM_138795
A_33_P33526	SP7	NM_001173467
A_32_P40648	NOTCH4	NM_004557
A_33_P33613	TMEM184A	NM_001097620
A_33_P33011	EPHA10	NM_173641
A_23_P16440	KRTAP2-4	NM_033184
A_23_P16006	ZNF600	NM_198457
A_23_P31838	ZNF683	NM_173574
A_23_P56883	GABRA4	NM_000809
A_33_P32361	ANG	NM_001145
A_23_P17382	ZNF335	NM_022095
A_23_P20014	DCAF8	NM_015726
A_24_P98047	SLC16A10	NM_018593

A_23_P25532{	PRDM8	NM_020226
A_24_P27386!	MED20	NM_004275
A_33_P33481(	TTLL7	NM_024686
A_23_P29949	EPGN	NM_001013442
A_33_P32156:	CHRNB2	NM_000748
A_23_P37002:	GGT7	AK124788
A_23_P25869{	MANBA	NM_005908
A_23_P21631(	CNOT7	NM_013354
A_23_P20248	MAP2K1	NM_002755
A_33_P32649:	NKX6-2	NM_177400
A_33_P34033!	AACS	NM_023928
A_33_P34055:	MAGEA5	NM_021049
A_33_P33171(	EPS8L3	NM_139053
A_23_P12485!	ZCCHC7	NM_032226
A_32_P18021(	FAM35A	NM_019054
A_33_P33918!	HRNR	NM_001009931
A_23_P16731:	RNF4	NM_002938
A_33_P32698(	ZNF616	NM_178523
A_24_P27670:	TARDBP	NM_007375
A_23_P50236:	EYA1	NM_000503
A_23_P17125!	IGBP1	NM_001551
A_23_P87879	CD69	NM_001781
A_23_P14834!	RNF128	NM_194463
A_24_P53976	GLUL	NM_002065
A_32_P38052‹	MYO1E	NM_004998
A_24_P11778:	SCRT2	NM_033129
A_23_P39602	NCOA1	NM_147233
A_33_P32613:	ENST0000031'	ENST00000319813
A_23_P25470:	DEK	NM_003472
A_23_P58770	HAND1	NM_004821
A_23_P25841!	TNIP2	NM_024309
A_23_P10619‹	FOS	NM_005252
A_23_P571	SLC2A1	NM_006516
A_33_P32623:	OTUD7A	NM_130901
A_24_P42501	ACOT9	NM_001037171
A_24_P11074:	ZNF844	AK027865
A_23_P82286	ZAN	NM_173059
A_24_P40873(	GALNT5	NM_014568
A_24_P41151!	TMPRSS11F	NM_207407
A_24_P13348{	CDCA4	NM_017955
A_23_P96623	OPN1MW	NM_000513
A_23_P13699‹	ZIC3	NM_003413
A_23_P57856	BCL6	NM_001130845
A_24_P23091(	MIER1	NM_001077702
A_32_P86064:	OLIG3	NM_175747
A_23_P43946	SARNP	NM_033082
A_23_P22682	ARMCX1	NM_016608
A_23_P25527:	RAD23B	NM_002874
A_23_P33619{	GLCCI1	NM_138426

A_23_P36078	SSRP1	NM_003146
A_23_P82859	OSGIN2	NM_004337
A_24_P30656	TCF25	NM_014972
A_33_P33208	CREB3L2	NM_194071
A_24_P30454	LAMP1	NM_005561
A_23_P43012	EPAS1	NM_001430
A_23_P67838	PGAP1	NM_024989
A_23_P77568	EIF3C	NM_001037808
A_23_P35607	FLT4	NM_002020
A_23_P21583	ATP5J2	NM_004889
A_23_P15163	TERF2	NM_005652
A_33_P32979	MYO1E	NM_004998
A_23_P50919	SERPINE2	NM_006216
A_24_P19357	CNOT1	NM_016284
A_33_P32302	NCAPH	NM_015341
A_23_P51699	ARHGEF2	NM_004723
A_33_P32350	TRIM47	NM_033452
A_32_P20152	TMEM97	NM_014573
A_23_P41421	PAX4	NM_006193
A_23_P10807	SLC7A10	NM_019849
A_33_P32153	NFATC2	NM_012340
A_24_P15742	NCBP2	NM_007362
A_23_P15967	TBX22	NM_016954
A_24_P45980	DLX2	NM_004405
A_33_P34165	AIG1	NM_016108
A_32_P18439	TFEC	NM_012252
A_23_P37788	KCNH2	NM_172056
A_23_P94571	ELAVL2	NM_004432
A_23_P70355	SERPINB6	NM_004568
A_23_P32491	PAX8	NM_013992
A_33_P33173	RHOG	NM_001665
A_23_P41658	GNAZ	NM_002073
A_23_P32080	ZNF354A	NM_005649
A_23_P35343	ZNF248	NM_021045
A_32_P18959	USHBP1	NM_031941
A_24_P27952	DHRS7B	NM_015510
A_23_P20668	WWP2	NM_199424
A_23_P20522	ATP7B	NM_000053
A_33_P33392	LHX8	NM_001001933
A_24_P14060	HBEGF	NM_001945
A_23_P16144	GDI2	NM_001494
A_23_P15596	PLK4	NM_014264
A_24_P74064	ZFP161	NM_003409
A_23_P37302	CACNA1C	NM_000719
A_23_P16130	CYP2A6	NM_000762
A_24_P99152	ZFP36L1	NM_004926
A_33_P37252	COBL	NM_015198
A_23_P44083	GFPT1	NM_002056
A_23_P33167	PYGB	NM_002862

A_23_P15844	SLC37A3	NM_032295
A_23_P83917	KDM2A	NM_012308
A_32_P87673	GCLC	AK094940
A_23_P59268	RANBP9	NM_005493
A_24_P15351	OSBPL8	NM_020841
A_32_P36243	SVIP	AL833119
A_23_P15728	C7orf23	NM_024315
A_32_P21383	FAM40B	NM_020704
A_23_P11533	ZBTB40	NM_014870
A_23_P13538	SP5	NM_001003845
A_24_P10695	PTGES2	NM_025072
A_24_P15650	EBF1	NM_024007
A_23_P36738	ANKRD13A	NM_033121
A_23_P27835	SUPT5H	NM_003169
A_24_P27484	TP53TG1	NR_015381
A_23_P34719	SP3	NM_003111
A_32_P37329	ZNF318	NM_014345
A_24_P39146	ENOX2	NM_182314
A_23_P82000	TEAD3	NM_003214
A_23_P15326	TRIM25	NM_005082
A_32_P13351	SUMO1	AL565221
A_23_P16099	NR5A2	NM_205860
A_23_P18046	TRPC1	NM_003304
A_23_P10162	ZNF667	NM_022103
A_32_P52916	KIAA1462	NM_020848
A_23_P78170	MYBBP1A	NM_014520
A_23_P11709	FGF23	NM_020638
A_24_P34520	DYRK3	NM_001004023
A_23_P32186	DMD	NM_004019
A_23_P8867	SDC2	NM_002998
A_23_P15536	HDAC11	NM_024827
A_23_P74278	PDE4B	NM_001037341
A_32_P83735	LGALS7	NM_002307
A_33_P34007	BNIPL	NM_001159642
A_32_P42873	ACTR3B	NM_001040135
A_23_P3793	SLC7A5	NM_003486
A_23_P21777	MSL3	NM_078629
A_23_P50003	GLI2	AB007295
A_32_P22702	PSIP1	NM_033222
A_32_P21714	ISX	NM_001008494
A_24_P12315	ZNF639	NM_016331
A_23_P3753	ZNF597	NM_152457
A_23_P13358	CDK7	NM_001799
A_23_P14804	TSPAN3	NM_005724
A_32_P18867	NPM1	NM_002520
A_24_P37676	CA6	NM_001215
A_23_P20971	SP100	NM_001080391
A_24_P94392	CACHD1	NM_020925
A_23_P21560	FAM49A	NM_030797

A_23_P40928[illegible]	HNF1B	NM_000458
A_23_P52425	NKX2-3	NM_145285
A_23_P21744[illegible]	LAMP2	NM_002294
A_23_P98052	ZNF32	NM_001005368
A_23_P66694	EVI2B	NM_006495
A_24_P30407[illegible]	IFIT2	NM_001547
A_23_P16523[illegible]	ZNF208	NM_007153
A_23_P12533[illegible]	ADK	NM_001123
A_23_P11792[illegible]	BAHD1	NM_014952
A_23_P14537[illegible]	MAPK13	NM_002754
A_23_P20938[illegible]	CASP8	NM_033355
A_23_P16320[illegible]	RIN3	AK021762
A_23_P10099[illegible]	KRT38	NM_006771
A_23_P10142[illegible]	EVI5L	NM_145245
A_24_P36552[illegible]	FOXA2	NM_021784
A_23_P15744[illegible]	POLR2K	NM_005034
A_23_P25095[illegible]	SLC26A2	NM_000112
A_23_P8297	SCML4	NM_198081
A_24_P30606[illegible]	FAM135A	NM_020819
A_23_P21635[illegible]	NFKBIL2	NM_013432
A_23_P10305	CTPS2	NM_175859
A_23_P54000	SNX6	NM_021249
A_23_P88404	TGFB3	NM_003239
A_33_P33112[illegible]	KRTAP19-4	NM_181610
A_23_P13024[illegible]	KRTAP1-3	NM_030966
A_32_P47763[illegible]	LOC388946	NM_001145051
A_24_P91939[illegible]	ATP10D	NM_020453
A_32_P18440	ARID5B	NM_032199
A_33_P32937[illegible]	KRTAP10-3	NM_198696
A_33_P36653[illegible]	PAX6	NM_000280
A_23_P56316	TIMM13	NM_012458
A_23_P71699	RORB	NM_006914
A_23_P10071[illegible]	PMP22	NM_000304
A_24_P94529[illegible]	ZNF788	NR_027049
A_23_P13876[illegible]	CLCF1	NM_013246
A_23_P13434[illegible]	CPVL	NM_019029
A_24_P92909[illegible]	TMEM209	AK027678
A_23_P34013[illegible]	PRSS16	NM_005865
A_23_P17326	UCKL1	NM_017859
A_23_P90696	TRIB2	NM_021643
A_24_P64407	HMX2	NM_005519
A_23_P36149[illegible]	ATF7	NM_006856
A_23_P42298[illegible]	FBXO36	NM_174899
A_32_P18360[illegible]	ASB1	NM_001040445
A_23_P80353	L3MBTL2	NM_031488
A_24_P29966[illegible]	ZNF238	NM_006352
A_33_P32949[illegible]	PRUNE2	NM_015225
A_33_P32204[illegible]	SMAD5	NM_001001419
A_23_P21111[illegible]	SIM2	NM_005069

A_24_P27256	ZDHHC13	NM_019028
A_32_P13365	NUP107	NM_020401
A_23_P37073	tAKR	NR_026743
A_32_P97288	KIAA1731	NM_033395
A_23_P21311	TEC	NM_003215
A_33_P33223	DDX11	NM_152438
A_23_P38337	KLF1	NM_006563
A_23_P10096	SPNS3	NM_182538
A_32_P49756	ENST0000031	ENST00000317596
A_32_P49578	WBSCR28	NM_182504
A_32_P18239	ZNF77	NM_021217
A_23_P25230	ID1	NM_002165
A_24_P17077	MED13	NM_005121
A_23_P41919	ZNF800	NM_176814
A_23_P15109	YARS2	NM_001040436
A_33_P33225	FANCL	NM_001114636
A_33_P35711	AP3D1	NM_003938
A_23_P87919	KDM2B	NM_032590
A_24_P38081	FKBP5	NM_004117
A_23_P16750	CYFIP2	NM_001037332
A_24_P39968	C20orf108	NM_080821
A_23_P13813	OMA1	NM_145243
A_23_P14599	GPR37	NM_005302
A_24_P34741	POSTN	NM_006475
A_23_P46429	CYR61	NM_001554
A_23_P16117	ZDHHC6	NM_022494
A_24_P11519	FOXK1	NM_001037165
A_23_P16731	RNF4	NM_002938
A_33_P34019	SPRED3	NM_001039616
A_23_P11465	HDAC1	NM_004964
A_33_P34019	SPRED3	NM_001039616
A_24_P20890	TRIM2	NM_015271
A_24_P10300	SLC20A1	NM_005415
A_32_P89788	GAPDH	NM_002046
A_23_P13443	EN2	NM_001427
A_33_P32839	CTNS	NM_004937
A_23_P95612	BBS10	NM_024685
A_23_P133	KDM1A	NM_015013
A_33_P33099	HDAC3	NM_003883
A_24_P25022	NR1D1	NM_021724
A_23_P13826	PADI4	NM_012387
A_33_P33418	ZNF780B	NM_001005851
A_23_P58337	FIP1L1	NM_030917
A_24_P31971	PDIA6	NM_005742
A_23_P26629	PYCARD	NM_013258
A_23_P32315	GLIS2	NM_032575
A_23_P17880	DNAL4	NM_005740
A_23_P13081	ZNF536	NM_014717
A_24_P14162	FAM111A	NM_022074

A_23_P54276	EID1	NM_014335
A_33_P33014	EXOSC4	NM_019037
A_23_P39910	C2orf64	NM_001008215
A_24_P39878	C14orf179	NM_052873
A_32_P68103	PRND	NM_012409
A_23_P11134	BCLAF1	NM_014739
A_24_P21212	ZNF33A	NM_006954
A_24_P37094	CYR61	NM_001554
A_24_P12628	BTN3A1	NM_007048
A_24_P30562	TMEM50B	NM_006134
A_23_P33159	IPO7	NM_006391
A_23_P11573	BMI1	L13689
A_24_P62615	CAP1	NM_006367
A_23_P12515	NFATC2IP	NM_032815
A_33_P33528	ZNF547	NM_173631
A_23_P14747	DNAJC6	NM_014787
A_23_P82402	GLCCI1	NM_138426
A_23_P38824	ZNF552	NM_024762
A_23_P36886	LCE1B	NM_178349
A_23_P33237	ZNF175	NM_007147
A_33_P33159	FIGLA	NM_001004311
A_32_P41801	LHFPL2	AK091868
A_23_P39123	MCM3APAS	AF426262
A_33_P33416	MEF2D	NM_005920
A_24_P16231	SCML1	NM_001037540
A_24_P37559	PHF20	AK090798
A_32_P54331	L3MBTL4	NM_173464
A_24_P55971	VEGFB	NM_003377
A_24_P38944	CCDC86	NM_024098
A_23_P31811	SMAGP	NM_001031628
A_23_P38899	ZC3H12C	NM_033390
A_24_P22761	CHURC1	NM_145165
A_33_P32876	KRT3	NM_057088
A_23_P73208	GABPB2	NM_144618
A_23_P2355	CBX5	NM_012117
A_24_P46737	SPIN4	NM_001012968
A_23_P12274	HSF2	NM_004506
A_24_P23524	PTPRZ1	NM_002851
A_23_P951	CFHR2	NM_005666
A_23_P31772	SP7	NM_152860
A_23_P12348	PRDM14	NM_024504
A_23_P50264	SEC22C	NM_032970
A_33_P34180	CTSO	NM_001334
A_24_P23148	MAP2	NM_002374
A_32_P36918	ZNF674	NM_001039891
A_23_P15535	BTD	NM_000060
A_23_P65609	GTF2A1	NM_015859
A_32_P12474	RHEB	BC009638
A_23_P32878	ZNF546	NM_178544

A_23_P20109	GUK1	NM_000858
A_23_P8820	FABP4	NM_001442
A_24_P26105	MTMR9	NM_015458
A_24_P35768	NIPBL	NM_015384
A_23_P35610	ATXN7L1	NM_152749
A_23_P69121	SIAH2	NM_005067
A_32_P31725	DLEU1	AK095311
A_23_P21304	LEF1	NM_016269
A_24_P12462	OLR1	NM_002543
A_23_P52127	ACBD6	NM_032360
A_23_P11598	ZNF684	NM_152373
A_23_P9166	TLN1	NM_006289
A_23_P79962	MKKS	NM_170784
A_23_P10683	BBS2	NM_031885
A_24_P27296	AVL9	NM_015060
A_32_P20343	ZNF30	NM_194325
A_23_P38120	KIAA0556	NM_015202
A_23_P29830	CBLB	NM_170662
A_23_P43034	ELP3	NM_018091
A_23_P13751	IVNS1ABP	NM_006469
A_23_P35838	RCE1	NM_005133
A_23_P25583	SLC7A2	NM_003046
A_23_P40156	MAT2A	NM_005911
A_23_P78428	ZSCAN30	NM_001166012
A_32_P49188	ALG9	NM_001077691
A_23_P39985	JAZF1	NM_175061
A_23_P85693	GBP2	NM_004120
A_23_P32562	SCAI	NM_173690
A_23_P42707	CTNS	NM_004937
A_23_P11228	TMOD1	NM_003275
A_23_P63432	RHBDL2	NM_017821
A_23_P12515	NFATC2IP	NM_032815
A_33_P32405	GRIP2	NM_001080423
A_23_P38968	ZNF358	NM_018083
A_23_P76658	N4BP2L1	NM_052818
A_24_P14716	PLA2G4F	NM_213600
A_33_P33783	C15orf51	NR_003260
A_32_P19716	ZNF697	NM_001080470
A_32_P32207	C10orf104	NM_173473
A_23_P16801	ZKSCAN3	NM_024493
A_23_P25949	MXD4	NM_006454
A_23_P15765	TNRC6C	NM_018996
A_23_P14218	HIF3A	NM_022462
A_33_P32816	KCNT1	NM_020822
A_23_P13365	LAMA4	NM_002290
A_24_P27234	SOX5	NM_152989
A_33_P32636	SEMA6B	NM_032108
A_32_P43303	FAM83A	NM_207006
A_32_P46036	B3GAT3	NM_012200

A_23_P57002	NNAT	NM_005386
A_23_P17190	KBTBD10	NM_006063
A_23_P34396	C1orf63	NM_020317
A_23_P40058	FNIP2	NM_020840
A_32_P12399	PDCD2	NM_002598
A_24_P39083	MPPE1	NM_023075
A_23_P34330	GFI1B	NM_004188
A_24_P6428	C20orf177	NM_022106
A_23_P43004	SUV420H2	NM_032701
A_33_P33634	SOBP	NM_018013
A_23_P20138	DDAH1	NM_012137
A_23_P15826	WBSCR16	NM_030798
A_23_P33784	CELF3	NM_007185
A_23_P15130	RAPGEF3	NM_006105
A_24_P31636	REXO2	NM_015523
A_23_P20654	SHCBP1	NM_024745
A_23_P21077	SPTLC3	NM_018327
A_33_P34059	RBM39	NM_184234
A_32_P73004	SSX2	NM_175698
A_24_P13960	PYHIN1	NM_198930
A_23_P91504	ITSN1	ENST00000381318
A_33_P32242	REXO1L1	NM_172239
A_33_P32265	SLC4A8	AK128321
A_24_P19882	CRKRS	NM_016507
A_24_P58453	NOL8	NM_017948
A_23_P21330	KLF3	NM_016531
A_24_P34373	SYT9	NM_175733
A_23_P79794	TGIF2	NM_021809
A_23_P15995	MID2	NM_012216
A_24_P75132	LRRC15	NM_130830
A_23_P31591	TCEB3C	NM_145653
A_23_P1615	FIBP	NM_004214
A_24_P70993	CD99	NM_002414
A_23_P12946	ATF7IP2	NM_024997
A_24_P79151	SYNCRIP	NM_006372
A_23_P15741	ZNF394	NM_032164
A_23_P12504	ZNF230	NM_006300
A_23_P92499	TLR2	NM_003264
A_32_P39728	ZNF354C	NM_014594
A_32_P13524	MTHFD1L	NM_015440
A_23_P15882	ARRB2	NM_004313
A_23_P16050	GLRX2	NM_016066
A_23_P65515	SIX4	NM_017420
A_23_P13326	HMGCS1	NM_002130
A_23_P63232	CREB3L4	NM_130898
A_23_P20076	TROVE2	NM_001042369
A_24_P10022	XBP1	NM_001079539
A_24_P88266	PROX1	NM_002763
A_23_P89550	NLRP1	NM_033004

A_23_P54469	ISL2	NM_145805
A_24_P49199	GLDN	NM_181789
A_23_P11440	MORF4L2	NM_012286
A_33_P33431	IRF8	NM_002163
A_24_P72518	AHCYL2	NM_015328
A_33_P32928	NEUROG3	NM_020999
A_24_P12184	ZNF132	NM_003433
A_32_P40952	UVRAG	NM_003369
A_33_P33485	OR9G1	NM_001005213
A_23_P14841	CSNK1G1	NM_022048
A_33_P34896	SPI1	NM_001080547
A_23_P11407	BPTF	NM_004459
A_23_P38770	KDSR	NM_002035
A_23_P93236	TRIM38	NM_006355
A_24_P41499	LAPTM4B	NM_018407
A_23_P71928	SH2D3C	NM_005489
A_32_P19222	DHX40	NM_024612
A_24_P34392	OAS2	NM_016817
A_33_P32543	SH2D3A	NM_005490
A_23_P77178	CCDC33	NM_182791
A_24_P37749	OSBPL3	NM_015550
A_23_P4367	TNRC6C	NM_018996
A_23_P21333	FGF1	NM_000800
A_23_P24365	ANKRD49	NM_017704
A_24_P76854	KRTAP2-1	NM_001123387
A_23_P43158	VPS8	NM_001009921
A_24_P17134	PUM1	NM_001020658
A_23_P33898	CYGB	NM_134268
A_23_P20450	PRKAB1	NM_006253
A_32_P12290	FTSJD1	NM_018348
A_23_P15527	CGGBP1	NM_001008390
A_23_P20288	FEZ1	NM_005103
A_23_P30438	HIGD2A	NM_138820
A_23_P36518	CAMTA2	NM_015099
A_23_P31163	NLRP6	NM_138329
A_23_P45940	TFB2M	NM_022366
A_23_P17084	PFKM	NM_000289
A_33_P32701	LEO1	NM_138792
A_33_P33189	UNC13B	NM_006377
A_24_P35813	ZNF776	NM_173632
A_23_P11970	RNF126	NM_194460
A_23_P14433	CCRN4L	NM_012118
A_24_P32832	GORASP2	NM_015530
A_24_P41659	ZNF575	NM_174945
A_24_P84428	CACYBP	NM_014412
A_23_P10628	FLJ10038	NR_026891
A_24_P27885	TMTC4	NM_032813
A_23_P12280	TMEM209	NM_032842
A_24_P37678	ZNF496	NM_032752

A_23_P25229:	CENPI	NM_006733
A_32_P21750(	ZBTB8A	NM_001040441
A_23_P20832!	ZNF235	NM_004234
A_33_P32664:	GLTP	NM_016433
A_23_P25247:	PECAM1	NM_000442
A_23_P39237	ZFP36	NM_003407
A_24_P83944	ZNF586	NM_017652
A_32_P71419:	ZNF619	NM_001145082
A_33_P33576!	HMGA2	NM_003484
A_23_P7965	PGC	NM_002630
A_33_P33028(	RPGRIP1L	NM_015272
A_23_P98304	ANO1	NM_018043
A_23_P31399:	PAX2	L25597
A_23_P24031	TLX1	NM_005521
A_23_P47168	FLRT1	NM_013280
A_23_P52826	BUD13	NM_032725
A_23_P46351	TDRKH	NM_006862
A_23_P39454	ZNF556	NM_024967
A_23_P21669:	MLLT3	NM_004529
A_24_P73730	CCDC113	NM_014157
A_24_P41186:	MLL5	NM_182931
A_23_P14576:	ARL4A	NM_005738
A_23_P80098	GART	NM_000819
A_24_P35564!	FLI1	NM_002017
A_23_P24623	ELP4	NM_019040
A_23_P17046:	USP3	NM_006537
A_23_P53530	MTERFD3	NM_001033050
A_23_P16741:	CNOT6	NM_015455
A_23_P15495·	MED15	NM_001003891
A_23_P16532!	PREPL	NM_006036
A_33_P32102{	FUT6	NM_000150
A_23_P14863	CHAC1	NM_024111
A_23_P12577:	HCFC1	NM_005334
A_23_P86037	UBE2Q1	NM_017582
A_32_P12865(	MID1	NM_000381
A_23_P12127:	NEK4	NM_003157
A_33_P33968{	C11orf52	NM_080659
A_23_P41630!	RPL27A	NM_000990
A_23_P11478:	PARP1	NM_001618
A_23_P16918!	IKBKAP	NM_003640
A_24_P30095:	APLP2	NM_001642
A_23_P18818	CNOT6	NM_015455
A_32_P11547!	IKZF3	ENST00000488188
A_24_P25074	ATXN7	NM_000333
A_23_P94230	LY96	NM_015364
A_23_P6474	JOSD1	NM_014876
A_23_P25644	PCID2	NM_018386
A_23_P20405:	PCBP2	NM_031989
A_24_P29807:	ANKRD10	NM_017664

A_24_P26516	FIP1L1	NM_030917
A_23_P69720	ANXA5	NM_001154
A_32_P17218	PLEKHH3	NM_024927
A_24_P38144	LMO3	NM_018640
A_33_P33278	NDEL1	NM_030808
A_23_P15163	SUPT16H	NM_007192
A_24_P37519	LZTFL1	NM_020347
A_23_P17655	KCNJ15	NM_170736
A_23_P46186	AKR1A1	NM_006066
A_24_P40968	MGA	NM_001164273
A_23_P32261	ZAN	NM_173059
A_32_P98534	KLHL12	NM_021633
A_32_P40935	ZIK1	AK128340
A_33_P33172	DPF1	NM_001135155
A_23_P78526	CEACAM19	NM_020219
A_32_P10118	C9orf5	AF086175
A_23_P39110	RUVBL2	NM_006666
A_33_P32961	CDCA7	NM_031942
A_24_P65060	MEX3B	NM_032246
A_23_P39088	PRMT1	NM_198319
A_23_P12257	DAXX	NM_001350
A_33_P33505	FTO	NM_001080432
A_23_P55448	KRT12	NM_000223
A_24_P41754	SCD5	BC004936
A_33_P32757	SEC14L1	NM_001143998
A_33_P32774	SLC26A2	NM_000112
A_23_P12900	NYNRIN	NM_025081
A_24_P55724	RNASE1	S79281
A_24_P31094	COG2	NM_007357
A_24_P30463	RPS6KA5	NM_004755
A_32_P48825	KRT72	NM_080747
A_33_P32558	MRS2	NM_020662
A_24_P28238	MYH7	NM_000257
A_23_P13993	ITM2B	NM_021999
A_33_P32563	TIMM8B	NM_012459
A_23_P11765	MYO5A	NM_000259
A_24_P11852	GIPC3	NM_133261
A_32_P31173	PLEKHG3	ENST00000247226
A_24_P20108	SLC25A37	NM_016612
A_33_P32764	CHMP1B	NM_020412
A_24_P91311	PTEN	NM_000314
A_23_P56416	LRRFIP1	NM_004735
A_23_P30071	SORBS3	NM_005775
A_24_P39383	TOMM20	NM_014765
A_33_P32678	MICAL3	NM_001122731
A_32_P18371	SP5	NM_001003845
A_33_P33534	USP37	NM_020935
A_24_P12599	HGS	NM_004712
A_33_P32483	ZFAND3	NM_021943

A_23_P10936	KRTAP4-1	NM_033060
A_23_P26583	NLRC5	NM_032206
A_23_P38136[illegible]	HOXD10	NM_002148
A_23_P76461	MYF6	NM_002469
A_23_P62042	PRR4	NM_001098538
A_23_P43940	ADAMTSL3	NM_207517
A_23_P32463	EXOSC4	NM_019037
A_32_P13922[illegible]	ZNF543	NM_213598
A_32_P20736[illegible]	AK2	NM_013411
A_23_P98458	VWA5A	NM_014622
A_24_P33112[illegible]	GNA15	NM_002068
A_24_P39832[illegible]	TRIM34	NM_001003827
A_23_P21859[illegible]	NPAS2	NM_002518
A_23_P13004[illegible]	PHB	NM_002634
A_23_P20342[illegible]	MTA2	NM_004739
A_23_P21158[illegible]	C22orf32	NM_033318
A_24_P89701	IMPDH1	NM_000883
A_33_P33881[illegible]	CHRNA3	NM_001166694
A_24_P33943[illegible]	GNAL	NM_002071
A_24_P27382[illegible]	NPAT	NM_002519
A_33_P33817[illegible]	TIAM1	NM_003253
A_24_P74981	KDM1A	NM_015013
A_24_P37840[illegible]	ZNF597	NM_152457
A_23_P14082[illegible]	ELMO3	NM_024712
A_33_P33417[illegible]	SP4	NM_003112
A_23_P65089	DDX47	NM_016355
A_24_P47988	ELL3	NM_025165
A_23_P91015	SMYD5	NM_006062
A_24_P92676[illegible]	NKTR	NM_005385
A_23_P21125[illegible]	LSS	NM_001001438
A_23_P38204[illegible]	TULP4	NM_020245
A_23_P30938[illegible]	HIST2H2AA4	NM_001040874
A_23_P13393[illegible]	DOM3Z	NM_005510
A_24_P10236[illegible]	ZNF48	ENST00000320159
A_23_P90659	LAPTM4A	NM_014713
A_23_P55802	AP1M1	NM_032493
A_23_P25263[illegible]	CSNK1G1	NM_022048
A_33_P32406[illegible]	NKX2-6	NM_001136271
A_23_P50206[illegible]	AQP6	NM_001652
A_23_P21828[illegible]	ZNF434	NM_017810
A_23_P49033	SNX1	NM_003099
A_33_P33074[illegible]	SYNRG	NM_001163547
A_23_P33278[illegible]	CHRNB4	NM_000750
A_23_P63010	LASS2	NM_181746
A_24_P17964[illegible]	TDGF3	M96956
A_33_P37375[illegible]	DAPK1	NM_004938
A_33_P33127[illegible]	BHLHA15	NM_177455
A_23_P41070[illegible]	VGLL2	NM_182645
A_23_P31217[illegible]	ALMS1	NM_015120

A_33_P34040	PGM5P2	NR_002836
A_23_P11520	FCRL4	NM_031282
A_23_P66383	PCGF2	NM_007144
A_33_P32392	CHD3	NM_001005271
A_23_P14566	EPO	NM_000799
A_24_P16581	CCNT2	ENST00000264157
A_23_P11611	PDE3B	NM_000922
A_24_P89080	DCK	NM_000788
A_32_P18911	MAP2K1	NM_002755
A_23_P34772	MLLT10	NM_001195627
A_23_P12508	IRF2	NM_002199
A_23_P21453	ZNF323	NM_030899
A_23_P60387	NOTCH1	NM_017617
A_23_P14503	KDM1B	NM_153042
A_23_P30630	OTOP1	NM_177998
A_23_P54147	NIN	NM_016350
A_23_P14324	TSHZ2	NM_173485
A_23_P61956	ATP6V0E2	NM_145230
A_23_P36698	TRHDE	NM_013381
A_33_P33393	ARHGAP11B	NM_001039841
A_24_P42589	TRIM73	BC033812
A_23_P21003	RPL31	NM_000993
A_23_P20666	NQO1	NM_000903
A_23_P1523	RHOD	NM_014578
A_23_P14687	DUX1	NM_012146
A_33_P32811	FUBP1	NM_003902
A_23_P15578	SULT1E1	NM_005420
A_24_P12117	HINFP	NM_015517
A_23_P20394	DDX11	NM_030653
A_23_P12110	HESX1	NM_003865
A_24_P25257	RABGAP1	NM_012197
A_24_P40874	CMC1	NM_182523
A_33_P38326	FAM25C	BC127791
A_23_P36568	LIMS3	NM_033514
A_32_P41897	TMEM20	NM_153226
A_23_P68423	JPH2	NM_175913
A_23_P16884	FBXO16	NM_172366
A_23_P21729	ZNF711	NM_021998
A_23_P20484	LCP1	NM_002298
A_24_P19950	RNF2	NM_007212
A_23_P16311	C14orf169	NM_024644
A_23_P21370	IL4	NM_000589
A_32_P93068	ZNF876P	NR_027481
A_23_P98057	ZNF32	NM_001005368
A_32_P18082	FMN1	NM_001103184
A_32_P50246	PSMB9	NM_002800
A_23_P37431	TAC4	NM_170685
A_23_P30078	CNOT4	NM_013316
A_23_P25177	PUS1	NM_025215

A_32_P17088	CNOT7	NM_013354
A_23_P89020	AARS	NM_001605
A_32_P22252	RNF14	ENST00000394520
A_24_P10194	SLCO4C1	NM_180991
A_23_P25959	AKAP7	NM_016377
A_23_P5027	EEF2	NM_001961
A_23_P21640	CHD3	NM_001005273
A_32_P41863	FAM110B	NM_147189
A_23_P27367	KDSR	NM_002035
A_32_P15965	KAT2B	NM_003884
A_23_P16035	TRAPPC2	NM_001011658
A_23_P12271	EPB41L2	NM_001431
A_23_P11691	AEBP2	NM_153207
A_23_P41971	BTBD11	NM_001018072
A_32_P44568	LDHA	NM_005566
A_24_P29854	ZNF257	NM_033468
A_23_P13763	PROX1	NM_002763
A_23_P30999	BCL2L11	NM_138621
A_23_P13102	GATAD2A	NM_017660
A_23_P21691	NR5A1	NM_004959
A_23_P16764	SLC36A1	NM_078483
A_24_P20327	KLF15	NM_014079
A_23_P15596	PLK4	NM_014264
A_23_P39680	NEGR1	NM_173808
A_23_P32504	TMPO	NM_003276
A_33_P33986	RBPJL	NM_014276
A_23_P11174	ZMIZ2	NM_031449
A_24_P14990	SUFU	NM_016169
A_33_P34044	MAMLD1	NM_001177465
A_23_P33850	C19orf40	NM_152266
A_23_P43192	SSX8	NR_027250
A_23_P40289	SLFN5	NM_144975
A_24_P17446	EAF1	NM_033083
A_33_P33947	DMRT2	NM_006557
A_23_P48070	ING4	NM_016162
A_33_P33392	UBN2	NM_173569
A_23_P68072	WDR54	NM_032118
A_23_P19619	HIVEP1	NM_002114
A_24_P37916	FOXO4	NM_005938
A_33_P33265	RHOT1	NM_018307
A_23_P7074	C4orf14	NM_032313
A_23_P39116	ZNF512	NM_032434
A_23_P1594	VEGFB	NM_003377
A_23_P56956	PDIA6	NM_005742
A_23_P89871	ZNF415	NM_018355
A_23_P37510	FANCI	NM_018193
A_23_P14319	MYBL2	NM_002466
A_33_P33718	NUAK1	NM_014840
A_24_P11623	C13orf1	NM_020456

A_23_P31099	HABP4	NM_014282
A_23_P32868	SIX5	NM_175875
A_23_P67088	ZNF772	NM_001024596
A_23_P16470	ZNF177	NM_003451
A_32_P10929	C15orf42	NM_152259
A_24_P11462	ADC	NM_052998
A_23_P70274	SLC16A10	NM_018593
A_32_P52742	KLHL31	NM_001003760
A_33_P33749	KLF17	NM_173484
A_24_P29950	REST	U13877
A_23_P33428	BMP2K	NM_017593
A_32_P68408	CSNK1A1P1	NR_027320
A_24_P85537	MAP3K13	NM_004721
A_24_P64781	KITLG	NM_000899
A_24_P92247	ATF7	BC042363
A_33_P32144	MESP2	NM_001039958
A_33_P32152	TTBK2	NM_173500
A_32_P11043	CYP20A1	NM_177538
A_32_P14003	FOXN4	NM_213596
A_23_P42935	BRAF	NM_004333
A_23_P38206	LSMD1	NM_032356
A_32_P88011	RNF128	NM_024539
A_23_P15348	KLK5	NM_012427
A_32_P9963	HSF2	NM_004506
A_23_P16573	KCNIP3	NM_013434
A_23_P13827	ARL8A	NM_138795
A_23_P11081	COX7C	NM_001867
A_23_P2262	MED21	NM_004264
A_32_P91663	MYT1L	NM_015025
A_32_P15063	ANXA11	ENST00000372234
A_24_P66610	LRRTM4	NM_001134745
A_24_P20715	UBE3A	NM_130839
A_32_P19364	RBMX	NM_002139
A_24_P92628	C2orf34	BC029359
A_23_P79259	SH3BP4	NM_014521
A_23_P14302	HOXD11	NM_021192
A_23_P39678	GLYR1	NM_032569
A_23_P6196	SCAND1	NM_016558
A_23_P38755	RARG	NM_000966
A_32_P42748	DUSP4	ENST00000240100
A_24_P33723	FGF5	NM_004464
A_23_P97296	NECAP2	NM_018090
A_32_P52812	FLJ38576	XR_112683
A_23_P9711	HS3ST4	NM_006040
A_23_P60225	GRHPR	NM_012203
A_33_P34182	ITGBL1	NM_004791
A_23_P13811	CAMTA1	NM_001195563
A_24_P86389	HIST1H2AM	NM_003514
A_23_P62106	TIMP1	NM_003254

A_23_P32577	DACH1	NM_080759
A_23_P1782	CD82	NM_002231
A_24_P37706	ARL8B	NM_018184
A_32_P43718	STK36	NM_015690
A_32_P22268	PRDM6	NM_001136239
A_23_P20859	LDLR	NM_000527
A_24_P82116	EEF1D	NM_032378
A_23_P32446	MIDN	NM_177401
A_23_P43597	KIF24	AK001795
A_23_P30630	OTOP1	NM_177998
A_23_P20927	TNKS	NM_003747
A_33_P32398	LSMD1	NM_032356
A_32_P48329	ZNF808	BC017109
A_23_P4856	KLK15	NM_138564
A_24_P16909	MAML2	NM_032427
A_32_P12470	ONECUT2	NM_004852
A_24_P32552	SORT1	NM_002959
A_33_P32910	THSD1	NM_199263
A_23_P71017	CLDN3	NM_001306
A_24_P41181	C7orf11	NM_138701
A_32_P66881	TLR4	NM_138554
A_23_P73420	TRAPPC9	NM_031466
A_23_P67864	ADCY3	NM_004036
A_23_P5300	CPS1	NM_001875
A_23_P16667	MFSD1	NM_022736
A_24_P16541	NBPF9	NM_001037675
A_23_P68827	CABIN1	NM_012295
A_23_P52086	RPS6KA1	NM_002953
A_23_P15220	CTCF	NM_006565
A_33_P33345	MTMR10	NM_017762
A_23_P61100	RABEP2	NM_024816
A_23_P56422	LRRFIP1	NM_004735
A_24_P39821	ZNF488	ENST00000298129
A_33_P33487	HSPH1	NM_006644
A_23_P12245	SDHC	NM_003001
A_23_P19219	ASF1A	NM_014034
A_23_P40416	HDAC9	NM_014707
A_23_P37223	CA12	NM_001218
A_33_P32294	PECAM1	NM_000442
A_24_P32935	ORAI2	NM_032831
A_23_P50273	PRRX1	NM_006902
A_33_P32773	OSM	NM_020530
A_23_P10205	HAS2	NM_005328
A_32_P18439	TFEC	NM_012252
A_23_P39511	TGFBRAP1	ENST00000393359
A_24_P34263	AK5	NM_174858
A_23_P24960	NARS2	NM_024678
A_32_P51549	ZNF316	XR_079205
A_23_P86230	MYCBP	NM_012333

A_23_P27894	SAFB2	NM_014649
A_23_P70448	HIST1H1A	NM_005325
A_23_P20392	PSD3	NM_015310
A_23_P89960	ZNF121	NM_001008727
A_24_P225679	IRS1	NM_005544
A_24_P263259	CCDC85C	NM_001144995
A_32_P863091	LOC10013085	AK097050
A_23_P97481	TTLL7	NM_024686
A_23_P146798	SEPHS2	NM_012248
A_24_P154031	IRS2	NM_003749
A_23_P155989	CENPK	NM_022145
A_24_P139941	HS1BP3	NM_022460
A_24_P204214	NCOR1	NM_006311
A_23_P33752	SLC25A6	NM_001636
A_33_P331580	CRKRS	NM_016507
A_23_P70328	CENPQ	NM_018132
A_23_P376096	TICAM1	NM_182919
A_23_P214760	HIVEP2	NM_006734
A_23_P427610	SAMD10	NM_080621
A_23_P218269	TAOK2	NM_016151
A_23_P65410	STRN3	NM_014574
A_23_P256205	ABLIM3	NM_014945
A_24_P179044	SNX9	NM_016224
A_23_P25746	C14orf128	NR_027263
A_24_P389218	ARHGEF5	NM_005435
A_33_P321385	RNASEH2B	NM_001142279
A_33_P328381	FOXS1	NM_004118
A_23_P218619	MRPL30	NM_145212
A_23_P14769	FES	NM_002005
A_23_P378526	RTEL1	NM_016434
A_33_P373669	ZNF430	NM_025189
A_32_P455122	RNF152	ENST00000312828
A_23_P133355	ZFP2	NM_030613
A_24_P367621	ZRANB3	NM_032143
A_23_P167308	RNF4	NM_002938
A_23_P23526	TRIM33	NM_033020
A_23_P93431	AIG1	NM_016108
A_24_P652700	CEP152	NM_014985
A_32_P535832	MAST3	NM_015016
A_32_P301720	TCF21	NM_198392
A_23_P139722	TNFRSF1A	NM_001065
A_23_P206776	TAT	NM_000353
A_23_P161624	FOSL1	NM_005438
A_33_P334008	POLDIP3	NM_178136
A_33_P323711	MMP2	NM_004530
A_24_P276981	STK25	NM_006374
A_24_P349965	TCF19	NM_007109
A_23_P166306	CBS	NM_000071
A_24_P35169	GATAD1	NM_021167

A_23_P14324	CEBPB	NM_005194
A_23_P77066	SNRPN	NM_022807
A_24_P10120	PDIA3	NM_005313
A_23_P21519	STK32B	NM_018401
A_24_P25805	MASTL	NM_032844
A_32_P10721	SSBP2	ENST00000320672
A_23_P40459	ZNF417	NM_152475
A_23_P78122	MYO18A	NM_078471
A_32_P74813	ZFP82	AB075828
A_23_P21449	BTN3A1	NM_007048
A_23_P43415	PITPNM2	NM_020845
A_23_P5685	TBR1	NM_006593
A_24_P68649	RNPEP	NM_020216
A_23_P41132	LCE1F	NM_178354
A_23_P50183	C5orf4	NM_032385
A_23_P16597	NFKBID	BC006273
A_23_P47199	SLC22A11	NM_018484
A_24_P37712	TEC	NM_003215
A_23_P31833	EEF1D	NM_032378
A_24_P21636	PRAME	NM_206956
A_23_P21557	HDAC9	NM_058176
A_24_P34909	LHFPL2	NM_005779
A_23_P43018	ZBTB3	NM_024784
A_23_P14550	EYA4	NM_004100
A_23_P41005	CIAO1	NM_004804
A_23_P29079	PFKL	NM_002626
A_33_P34114	ZNF837	NM_001129730
A_23_P20047	ACBD3	AB043587
A_23_P47370	H2AFX	NM_002105
A_24_P17735	THOC2	NM_001081550
A_23_P42487	KIAA1688	NM_025251
A_24_P56482	SNORD22	NR_000008
A_23_P19352	CNPY3	NM_006586
A_23_P35205	RCAN3	ENST00000374395
A_23_P12024	HOXD1	NM_024501
A_23_P56827	ASB3	NM_016115
A_23_P9170	TLN1	NM_006289
A_23_P10107	OR1E1	NM_003553
A_24_P26752	ZNF585A	NM_152655
A_23_P16780	B3GAT2	NM_080742
A_23_P46141	CTSS	NM_004079
A_23_P86171	FOXD2	NM_004474
A_23_P32722	ZNF408	NM_024741
A_23_P12754	TRPC6	NM_004621
A_23_P43504	ABCA2	NM_001606
A_23_P41646	PIF1	NM_025049
A_23_P54612	LRRC50	NM_178452
A_24_P33156	STS	NM_000351
A_23_P11311	AR	NM_000044

A_23_P52046	VAMP4	NM_003762
A_33_P32732	ZNF701	NM_001172655
A_23_P32277	BARX1	NM_021570
A_23_P30024	DMRTC2	NM_001040283
A_23_P24557	PHLDB1	NM_015157
A_23_P38603	KRTAP4-4	NM_032524
A_23_P61569	VAMP4	NM_003762
A_23_P27636	ZNF700	NM_144566
A_23_P71300	CCDC25	NM_018246
A_32_P80509	LOC10028859	AK024457
A_32_P13739	C14orf33	NR_027123
A_23_P35668	ANLN	NM_018685
A_23_P21828	ZNF434	NM_017810
A_24_P39323	HLA-DQA1	NM_002122
A_33_P32641	LCE3E	NM_178435
A_23_P15071	RHOG	NM_001665
A_23_P37654	MGA	NM_001164273
A_23_P55846	LOC147804	NR_003148
A_23_P39437	KCNRG	NM_199464
A_23_P95359	DNAJC6	NM_014787
A_32_P19108	KCTD5	NM_018992
A_23_P21228	WDR51A	NM_015426
A_23_P45011	PPP1R14C	NM_030949
A_23_P10806	LGALS7B	NM_001042507
A_32_P42298	OR2A7	BU929836
A_24_P31800	ZNF215	NM_013250
A_23_P21348	PARP8	ENST00000281631
A_23_P52198	MPP7	NM_173496
A_23_P32499	STAB1	NM_015136
A_32_P15187	ZNF398	NM_020781
A_24_P15172	NONO	NM_007363
A_23_P13006	SNF8	NM_007241
A_23_P11186	ZSCAN21	NM_145914
A_23_P73348	IRX6	NM_024335
A_24_P11410	C1orf51	NM_144697
A_23_P29414	GYG1	NM_001184720
A_24_P92325	TGM2	NM_198951
A_23_P67980	KLF7	NM_003709
A_23_P51729	GJC2	NM_020435
A_33_P33422	VSX1	NM_199425
A_33_P32750	ZBTB40	NM_001083621
A_23_P21466	RPS18	NM_022551
A_23_P16142	PLXDC2	NM_032812
A_23_P34985	NRG2	NM_004883
A_23_P58031	MAP3K13	NM_004721
A_32_P40922	ZNF628	NM_033113
A_24_P20347	MSH3	NM_002439
A_33_P32862	DLEU2L	NR_002771
A_23_P11286	HNRNPH2	NM_019597

A_24_P33176	PSG7	NM_002783
A_23_P13487	NCOA2	NM_006540
A_24_P98385	RNF20	NM_019592
A_24_P30314	ANKH	NM_054027
A_23_P17138	PHF6	NM_032335
A_23_P60599	UGT1A6	NM_001072
A_32_P38772	MBOAT1	NM_001080480
A_23_P35779	MTBP	NM_022045
A_23_P11338	USP47	NM_017944
A_24_P23385	SDHC	NM_003001
A_23_P42202	ME1	NM_002395
A_23_P39817	FAM135A	NM_020819
A_32_P17648	GALM	NM_138801
A_23_P25369	GPR64	NM_001079858
A_32_P14039	UPF3A	NM_023011
A_23_P12672	NOL9	NM_024654
A_23_P14217	FOXA3	NM_004497
A_23_P35352	IVL	NM_005547
A_24_P33000	POLR2L	NM_021128
A_24_P82703	LRRC15	NM_130830
A_33_P33628	TNNI1	NM_003281
A_23_P43604	LPAR3	NM_012152
A_33_P34212	NKX6-3	NM_152568
A_23_P13158	BMP10	NM_014482
A_23_P11947	EBI3	NM_005755
A_23_P13877	ACP2	NM_001610
A_23_P16944	SDC1	NM_001006946
A_23_P35595	SIGLEC8	NM_014442
A_24_P92418	MYO10	BC108736
A_23_P41004	BRD7	NM_013263
A_23_P12714	RAB11FIP2	NM_014904
A_23_P62270	KDM5C	NM_004187
A_23_P14005	FAM48A	NM_017569
A_24_P54131	DCLRE1B	NM_022836
A_23_P14716	CACNA2D3	AF516696
A_23_P11045	CD14	NM_000591
A_33_P32588	SLURP1	NM_020427
A_23_P78771	NUCB1	NM_006184
A_23_P12438	SHOX2	NM_006884
A_24_P82880	TPM4	NM_003290
A_23_P25909	ZSCAN16	NM_025231
A_33_P32286	LOC151009	NR_027244
A_24_P40373	ZNF385A	NM_015481
A_33_P32701	RNF126	NM_194460
A_33_P32727	ZNF217	NM_006526
A_33_P32643	FOXD3	NM_012183
A_23_P26878	NDEL1	NM_001025579
A_23_P17060	TSPYL2	NM_022117
A_24_P22714	ELF5	NM_198381

A_24_P56317	MBNL2	NM_144778
A_24_P31563{	MRGPRE	NM_001039165
A_23_P13929	NRIP2	NM_031474
A_23_P13893{	PGR	NM_000926
A_23_P10954:	SMCR7L	NM_019008
A_33_P33621:	PROX2	NM_001080408
A_24_P27715!	HLTF	NM_003071
A_23_P29081	PFKL	NM_002626
A_32_P41309-	TTTY2	NR_001536
A_23_P20239(	SUV39H2	NM_024670
A_33_P36598:	NCAPG2	NM_017760
A_33_P33090:	TBC1D8	NM_001102426
A_33_P34126:	TMPO	NM_001032283
A_23_P29422	GYG1	NM_004130
A_23_P16759!	UBE2B	NM_003337
A_33_P32683:	LIMS3	NM_033514
A_33_P32513:	HEATR5A	NM_015473
A_23_P32410:	RORC	NM_005060
A_24_P79750	AKR1A1	NM_006066
A_32_P99431	TRAPPC5	NM_174894
A_24_P72064	GHR	NM_000163
A_33_P32265-	SNORD3B-1	NR_003271
A_23_P43550:	SERINC3	NM_198941
A_24_P35979!	OTX1	NM_014562
A_23_P12528(	OR52A1	NM_012375
A_23_P16645!	LGALS1	NM_002305
A_32_P35107!	ZNF713	NM_182633
A_32_P21494:	PCBP2	NM_031989
A_23_P24926	FNTA	NM_002027
A_23_P25284!	GTF2H2D	NM_001042490
A_23_P32426	RPF1	NM_025065
A_24_P12065	CCNG2	NM_004354
A_23_P11628(	WT1	NM_024426
A_23_P42711-	GORASP1	NM_031899
A_23_P21113(	BRWD1	NM_018963
A_23_P41233!	TGFB1	NM_000660
A_24_P13771:	ZNF323	NM_030899
A_32_P1192	COQ10B	AK096309
A_23_P32389{	ZSCAN12	NR_028077
A_33_P32828-	RPS29	NM_001030001
A_23_P31762(	ARL4C	NM_005737
A_23_P16720:	SEPSECS	NM_016955
A_23_P40529!	LCE3C	NM_178434
A_23_P51479	INSL5	NM_005478
A_24_P33502!	ENTPD1	NM_001776
A_23_P84367	SSX5	NM_021015
A_23_P13090(	NCLN	NM_020170
A_23_P61498	KRT76	NM_015848
A_24_P46357	MYO10	NM_012334

A_23_P71821	PBX3	NM_006195
A_23_P10752	NFATC1	NM_172390
A_24_P41347	TP73	NM_005427
A_23_P16066	MYCL1	NM_005376
A_23_P40467	RAB3D	NM_004283
A_23_P14498	PIK3R1	NM_181523
A_24_P39922	HOXB3	NM_002146
A_23_P20507	SLC46A3	NM_181785
A_32_P11642	DHPS	NM_001930
A_23_P88141	GPHN	NM_020806
A_23_P25964	GALC	NM_000153
A_23_P35130	FAM123A	NM_152704
A_23_P16792	DLL1	NM_005618
A_23_P11214	NKRF	NM_017544
A_33_P32719	PYCR1	NM_153824
A_32_P17858	CDC5L	ENST00000371477
A_23_P15375	LSM4	NM_012321
A_23_P73345	MITF	NM_198159
A_23_P12744	DPF2	NM_006268
A_24_P16545	TTLL7	NM_024686
A_23_P11470	H6PD	NM_004285
A_23_P91979	TP63	NM_003722
A_24_P14776	FOXRED2	NM_024955
A_24_P84396	KIAA1199	NM_018689
A_23_P15325	ZNF274	NM_133502
A_23_P14575	HOXA1	NM_005522
A_24_P39814	NEBL	NM_006393
A_23_P20283	CCND1	NM_053056
A_23_P35007	C19orf46	NM_001039876
A_32_P62803	STK3	NM_006281
A_23_P52067	GRHL3	NM_198173
A_33_P32509	TP53TG1	NR_015381
A_23_P13216	CLTCL1	NM_007098
A_23_P12967	SETD1A	NM_014712
A_32_P43483	CCHCR1	NM_001105564
A_24_P30359	ACTR3B	NM_001040135
A_24_P32695	DCAF11	NM_025230
A_24_P11310	UBB	NM_018955
A_24_P39665	RPS6KA1	NM_002953
A_33_P34199	VHL	NM_000551
A_23_P39150	IVNS1ABP	NM_006469
A_32_P32475	ENOX2	NM_006375
A_24_P29070	TOM1L1	CR593166
A_24_P94148	ZNF761	NM_001008401
A_24_P22283	S100PBP	NM_001017406
A_23_P16706	UGDH	NM_003359
A_24_P57207	CREB3L3	NM_032607
A_32_P86976	NIPAL1	NM_207330
A_24_P19653	PKNOX1	NM_004571

A_23_P32701	KIAA0146	NM_001080394
A_32_P11045	CRK	NM_016823
A_23_P73097	RGS20	NM_170587
A_23_P21332	ANKHD1	NM_017747
A_33_P32714	GREB1L	NM_001142966
A_32_P17913	GUCY1A3	NM_001130683
A_23_P43476	VLDLR	NM_003383
A_23_P12578	TCEAL7	NM_152278
A_23_P82509	GATAD1	NM_021167
A_23_P3856	ZFP1	NM_153688
A_23_P33359	ZNF547	NM_173631
A_23_P50038	HTR7	NM_019859
A_24_P17329	KLF16	NM_031918
A_23_P2582	HDAC7	NM_015401
A_33_P32141	ZNF532	NM_018181
A_23_P92202	GMPPB	NM_021971
A_23_P14384	TIPARP	NM_015508
A_23_P80566	RASA2	NM_006506
A_23_P33250	NBAS	NM_015909
A_32_P18243	POLD3	NM_006591
A_23_P21597	CCNE2	NM_057749
A_23_P10350	MDM4	NM_002393
A_33_P33297	ANKLE2	NM_015114
A_24_P47547	RAN	NM_006325
A_23_P28388	FIGLA	NM_001004311
A_33_P38769	PPPDE1	NM_016076
A_23_P16686	CACNA2D2	NM_001005505
A_23_P71319	FDFT1	NM_004462
A_23_P9677	SEMA4C	NM_017789
A_32_P48697	NRK	NM_198465
A_32_P21684	SPATA18	NM_145263
A_23_P25479	BPIL1	NM_025227
A_23_P38218	STAP2	NM_001013841
A_24_P17100	MYST4	NM_012330
A_23_P34293	TLE3	NM_005078
A_24_P56484	BRMS1L	NM_032352
A_24_P23015	JRK	NM_003724
A_24_P20795	IRX4	NM_016358
A_24_P31493	RNF39	NM_025236
A_23_P39546	KIF1A	NM_004321
A_23_P21125	LSS	NM_001001438
A_32_P34108	TFEB	NM_007162
A_23_P29634	NCKIPSD	NM_184231
A_23_P15650	IRF4	NM_002460
A_23_P14155	TBX21	NM_013351
A_32_P45192	HAUS5	NM_015302
A_23_P12533	ANKRD30A	NM_052997
A_32_P20450	PTPRM	NM_002845
A_23_P11181	ING3	NM_019071

A_32_P34652[illegible]	VKORC1L1	ENST00000360768
A_33_P34012[illegible]	HSP90B3P	NR_003130
A_23_P39371[illegible]	IGHMBP2	NM_002180
A_33_P32619[illegible]	AP4B1	NM_006594
A_23_P2501	PAH	NM_000277
A_32_P79021[illegible]	FOXL2	NM_023067
A_23_P38031[illegible]	EGR4	NM_001965
A_23_P85265	SRY	NM_003140
A_23_P31961[illegible]	CHST7	NM_019886
A_23_P16096	PPAN	NM_020230
A_23_P43318[illegible]	GZF1	NM_022482
A_24_P29931[illegible]	FAM101B	NM_182705
A_24_P85200	C14orf43	NM_194278
A_23_P12766[illegible]	PRRG4	NM_024081
A_23_P38522[illegible]	DSG4	NM_177986
A_23_P14817[illegible]	FRMD4B	NM_015123
A_24_P55097[illegible]	POM121L4P	NR_024592
A_23_P50025[illegible]	YWHAB	NM_003404
A_23_P49220	TCF25	NM_014972
A_23_P74538	CD53	NM_001040033
A_23_P34825[illegible]	NUAK1	NM_014840
A_32_P67533	L3MBTL3	NM_032438
A_23_P23173	SNIP1	NM_024700
A_24_P12904	C9orf5	NM_032012
A_23_P91630	POM121L9P	NR_003714
A_23_P35538[illegible]	PPP6C	NM_002721
A_23_P41111[illegible]	CNTNAP1	NM_003632
A_23_P21178	DACH1	NM_080759
A_23_P34289[illegible]	NEFM	BC002421
A_23_P45025	MAPK10	NM_138980
A_33_P34233[illegible]	GSN	NM_001127663
A_24_P29015[illegible]	COL4A5	NM_000495
A_23_P35684	INPP5F	NM_014937
A_23_P21840[illegible]	GPS2	NM_004489
A_24_P36745	CXorf38	NM_144970
A_24_P27259[illegible]	MAPKBP1	NM_014994
A_24_P24581[illegible]	ASPHD2	NM_020437
A_23_P20059[illegible]	MUL1	NM_024544
A_33_P32947[illegible]	CYLC1	NM_021118
A_23_P16115[illegible]	PDSS1	NM_014317
A_23_P34092[illegible]	ZNF414	NM_032370
A_32_P30320[illegible]	HMBOX1	NM_024567
A_24_P28158[illegible]	GATS	NM_178831
A_23_P16483	STK11	NM_000455
A_24_P15629[illegible]	ACSS2	NM_018677
A_23_P42219[illegible]	SUV39H1	NM_003173
A_23_P50232[illegible]	AGRP	NM_001138
A_23_P24555	PHLDB1	NM_015157
A_24_P97104	DPP4	NM_001935

A_23_P10335	TNFRSF8	NM_001243
A_23_P13525	PRSS3	NM_002771
A_23_P14297	ARHGAP25	NM_001007231
A_23_P52311	TAF5	NM_006951
A_24_P25686	THRAP3	NM_005119
A_23_P34757	GPATCH2	NM_018040
A_23_P41004	KCNH7	NM_173162
A_23_P20357	SGK3	NM_013257
A_23_P14952	TACSTD2	NM_002353
A_23_P12616	HPCA	NM_002143
A_32_P18245	SMARCC1	NM_003074
A_23_P16081	GBP6	NM_198460
A_24_P32244	NUP62	NM_153719
A_23_P32927	MC1R	NM_002386
A_23_P39734	MCM9	NM_153255
A_23_P11100	PSMB9	NM_002800
A_24_P28902	TBC1D5	NM_014744
A_23_P90079	ZNF799	NM_001080821
A_23_P61236	RGS20	NM_170587
A_24_P30806	EID1	NM_014335
A_23_P25927	WSB2	NM_018639
A_33_P32596	ZNF134	NM_003435
A_23_P21880	ZC3H7B	NM_017590
A_32_P12321	TMEM117	NM_032256
A_24_P19728	C2orf85	NM_173821
A_23_P21120	MED14	ENST00000324817
A_23_P11428	MCTS1	NM_014060
A_23_P6878	SEMA3F	NM_004186
A_23_P20006	EPHB2	NM_004442
A_24_P53847	MED28	NM_025205
A_23_P12752	ETS1	NM_005238
A_23_P83916	KDM2A	NM_012308
A_23_P20146	PER3	NM_016831
A_23_P23572	PIP5K1A	NM_003557
A_24_P50062	HIPK2	NM_022740
A_24_P32906	BTN3A1	NM_007048
A_33_P32416	C2orf34	NM_024766
A_24_P27829	ASB13	NM_024701
A_32_P9597	ST6GALNAC2	BC038114
A_23_P15531	NCBP2	NM_007362
A_23_P36138	DMC1	NM_007068
A_23_P35845	NADSYN1	NM_018161
A_24_P61040	TGFBI	AI754733
A_23_P14255	GUCY1B2	NR_003923
A_32_P13360	DMRTC2	NM_001040283
A_33_P33372	NRARP	NM_001004354
A_33_P37886	ACER2	NM_001010887
A_23_P93442	SASH1	NM_015278
A_23_P35534	NEUROG3	NM_020999

A_23_P10508[illegible]	ASCL2	NM_005170
A_33_P34213[illegible]	KIF18B	NM_001080443
A_23_P15159[illegible]	CPNE6	NM_006032
A_23_P16829[illegible]	NR2E1	NM_003269
A_24_P20258[illegible]	ARHGEF1	NM_199002
A_23_P14537	TMED10	NM_006827
A_23_P20828[illegible]	ZNF304	NM_020657
A_24_P25676[illegible]	MYH10	NM_005964
A_23_P10127[illegible]	ZNF446	NM_017908
A_24_P14871[illegible]	CCR1	NM_001295
A_23_P16193[illegible]	SYT7	NM_004200
A_32_P43876[illegible]	NEUROG2	NM_024019
A_23_P10735[illegible]	SLC2A4	NM_001042
A_23_P92385	TLL1	NM_012464
A_32_P52442[illegible]	CMC1	NM_182523
A_32_P42009[illegible]	TBCK	ENST00000273980
A_32_P22195[illegible]	AGAP7	NM_001077685
A_23_P33436	MAZ	NM_002383
A_33_P32533[illegible]	LAIR1	NM_002287
A_32_P81001	TMF1	NM_007114
A_23_P25751	C14orf128	NR_027263
A_32_P32296[illegible]	MED12L	NM_053002
A_23_P11372[illegible]	MTG1	NM_138384
A_23_P40276[illegible]	NRAP	NM_198060
A_23_P64854	KRT75	NM_004693
A_23_P17082[illegible]	PUM1	NM_001020658
A_23_P94689	URM1	NM_030914
A_24_P25053[illegible]	TMX4	NM_021156
A_23_P55854	LOC147804	NR_003148
A_33_P32744[illegible]	UNC119B	NM_001080533
A_23_P40762[illegible]	MYST3	NM_006766
A_32_P25823	VPS41	ENST00000310301
A_32_P52043[illegible]	EMILIN2	NM_032048
A_32_P76035	PNPT1	NM_033109
A_23_P88589	NR2F2	NM_021005
A_23_P14296[illegible]	ZNF2	NM_021088
A_24_P39078[illegible]	SREBF1	NM_001005291
A_23_P42019[illegible]	SOCS1	NM_003745
A_24_P83678	C6orf167	NM_198468
A_32_P81149	RPL14	NM_001034996
A_23_P37043[illegible]	C1QBP	NM_001212
A_24_P10168[illegible]	FOXK1	NM_001037165
A_24_P91726[illegible]	SETX	NM_015046
A_33_P32156[illegible]	FAM113B	NM_138371
A_23_P10559	AATK	NM_001080395
A_23_P16242	ZNF20	NM_021143
A_23_P13723[illegible]	KDM5D	NM_004653
A_32_P17132[illegible]	UBE2S	NM_014501
A_23_P35017[illegible]	RNF169	NM_001098638

A_24_P28363!	ADAMTSL1	NM_001040272
A_24_P25562{	PACS2	NM_015197
A_23_P15639	C17orf75	NM_022344
A_23_P98252	ARL2	NM_001667
A_23_P10745‹	KRTAP3-1	NM_031958
A_23_P40761‹	PYDC1	NM_152901
A_23_P13424;	RHEB	NM_005614
A_23_P49041	TMEM62	NM_024956
A_23_P32029	SLC35D2	NM_007001
A_23_P21547!	CLIP2	NM_003388
A_33_P33689(	FLJ39739	AK097058
A_24_P35985!	HDAC4	NM_006037
A_23_P14064{	CYFIP1	NM_014608
A_23_P13649;	PDGFA	NM_002607
A_23_P23664	PALMD	NM_017734
A_33_P32668;	ZBTB8B	NM_001145720
A_23_P15877;	GOLIM4	NM_014498
A_32_P19263;	GPC5	NM_004466
A_23_P21180(	LRRFIP2	NM_017724
A_23_P66421	NAT9	NM_015654
A_23_P13948(	CDK2AP1	NM_004642
A_23_P15108	YPEL3	NM_031477
A_23_P21531{	VPS41	NM_014396
A_24_P16680;	TPD52	NM_001025252
A_23_P25610;	HPSE	NM_006665
A_23_P37911;	ENST0000031	ENST00000317596
A_32_P72022{	DCD	NM_053283
A_23_P33099!	MED23	NM_015979
A_24_P19810	PPCS	NM_024664
A_32_P86	GUSBP1	BC048193
A_23_P13940{	ZNF148	NM_021964
A_23_P35070‹	PRKAB2	NM_005399
A_32_P47438;	ACOX1	NM_004035
A_33_P32759!	SLC25A16	BC001407
A_32_P76156	RWDD4A	NM_152682
A_24_P94454;	PREPL	NM_006036
A_23_P14935!	SLC22A15	NM_018420
A_23_P55504	OR3A2	NM_002551
A_33_P33808;	AMZ1	NM_133463
A_23_P12127(	NEK4	NM_003157
A_33_P32705!	TPM2	NM_213674
A_23_P15857	PPP4R1	NM_001042388
A_23_P15496;	RIMBP3	NM_015672
A_24_P11702!	LDLR	NM_000527
A_24_P47182	VCL	NM_014000
A_23_P25552;	ALKBH4	NM_017621
A_24_P16026;	MAGED2	NM_201222
A_23_P33948(	HAT1	NM_003642
A_23_P15306{	BCAS3	NM_017679

A_23_P13914:	STX3	NM_004177
A_24_P30719!	AASDH	NM_181806
A_23_P10979‹	ANKRD28	NM_015199
A_23_P31890‹	SERTAD4	NM_019605
A_24_P12897:	G3BP2	NM_203505
A_23_P57323	ERG	NM_004449
A_23_P99452	BRCA2	NM_000059
A_33_P34005:	HLF	NM_002126
A_23_P16530:	RPE	NM_006916
A_33_P32301:	ATAD3C	NM_001039211
A_23_P67288	CALR	NM_004343
A_23_P50099{	HOXA9	NM_152739
A_23_P13372{	GCM1	NM_003643
A_24_P20076:	NUP43	NM_198887
A_23_P16121{	ANKRD1	NM_014391
A_32_P21057:	C1orf53	NM_001024594
A_32_P17011!	TRAK1	NM_001042646
A_23_P4400	KRTAP4-11	NM_033059
A_23_P10640!	NDN	NM_002487
A_23_P20881:	ZNF507	NM_014910
A_23_P12455:	MXD3	NM_031300
A_23_P15516	TMEM101	NM_032376
A_23_P15104	YPEL3	NM_031477
A_23_P15452:	MTA3	NM_020744
A_23_P42270:	NR2F2	NM_021005
A_33_P33153(	KRT73	NM_175068
A_23_P15035(	C11orf1	NM_022761
A_32_P16424(	FOXQ1	NM_033260
A_24_P56240	CPNE8	NM_153634
A_23_P20746:	CA10	NM_020178
A_33_P33366:	ALDH3A2	NM_001031806
A_32_P87754{	ZNF665	AK096261
A_23_P43817	MFSD2A	NM_032793
A_24_P23825(	LGALS7	NM_002307
A_23_P54846	HERPUD1	NM_014685
A_23_P32566:	ZNF134	NM_003435
A_33_P32277:	GATSL3	NM_001037666
A_23_P30815(	FAM123B	NM_152424
A_24_P60287:	SAMD5	NM_001030060
A_23_P75470	SCYL1	NM_001048218
A_24_P32219:	CALCOCO2	NM_005831
A_23_P12716:	C10orf99	NM_207373
A_24_P13423!	KHSRP	NM_003685
A_24_P68758:	TADA2B	NM_152293
A_33_P34152:	MPV17	NM_002437
A_32_P37394!	SLCO5A1	AK022971
A_23_P14520‹	HFE	NM_000410
A_23_P66924	DYM	NM_017653
A_24_P10672{	PUS7	NM_019042

A_23_P43207	KIAA0564	NM_015058
A_23_P94053	TRRAP	NM_003496
A_32_P44787	KLF17	NM_173484
A_23_P12017	TIGD1	NM_145702
A_24_P20937	ZNF107	NM_016220
A_23_P27096	PFN1	NM_005022
A_23_P10245	INSIG2	NM_016133
A_23_P37183	GATA5	NM_080473
A_33_P32905	FAM46A	NM_017633
A_33_P34241	DHX40	NM_024612
A_23_P13319	SLC25A4	NM_001151
A_32_P11424	USP47	NM_017944
A_32_P12614	ST6GAL2	NM_032528
A_33_P32557	POLE	NM_006231
A_23_P83768	USP3	NM_006537
A_24_P38792	KMO	NM_003679
A_24_P14971	ZBTB43	NM_014007
A_33_P33840	RAX	NM_013435
A_23_P23155	AJAP1	NM_018836
A_23_P61623	SLCO3A1	NM_001145044
A_23_P15942	ELAC1	NM_018696
A_32_P13686	RPS13	NM_001017
A_23_P10450	FAM53B	NM_014661
A_24_P18630	KDM2B	NM_032590
A_23_P31356	NCOA3	NM_181659
A_24_P23623	FLRT2	NM_013231
A_33_P32545	KRTAP1-5	NM_031957
A_23_P80832	TMEM41A	NM_080652
A_24_P30233	NECAP2	NM_018090
A_23_P48807	GALK2	NM_001001556
A_23_P11962	NDUFA13	NM_015965
A_23_P70233	ALDH7A1	NM_001182
A_23_P25086	TSC22D4	NM_030935
A_24_P32151	FAM101B	NM_182705
A_32_P35667	KIAA0226	NM_001145642
A_23_P39796	FOXK2	NM_004514
A_32_P15302	MPPED2	NM_001584
A_23_P15376	AKAP8L	NM_014371
A_24_P26268	LAIR1	NM_021706
A_23_P30495	HMGCR	NM_000859
A_23_P60585	MPI	NM_002435
A_23_P16584	ODC1	NM_002539
A_32_P51258	TNIK	NR_027767
A_33_P33853	NOL7	NM_016167
A_24_P30184	GART	NM_175085
A_23_P13617	CSF2RA	NM_172247
A_23_P55572	CLUL1	NM_014410
A_23_P11830	DNAJA3	NM_005147
A_24_P21423	STIL	NM_001048166

A_23_P23561	CELF3	NM_007185
A_23_P11466	CRYZ	NM_001889
A_24_P39858	UNG	NM_003362
A_24_P19983	BCL10	NM_003921
A_23_P31844	ATP6V1B2	NM_001693
A_23_P48691	ARF6	NM_001663
A_24_P38511	TAF2	NM_003184
A_23_P10774	S1PR5	NM_030760
A_33_P33683	LRRC50	NM_178452
A_24_P32663	ADRBK1	NM_001619
A_24_P14368	KIAA0146	NM_001080394
A_23_P13461	COG5	NM_006348
A_23_P28084	STX10	ENST00000242770
A_24_P48184	HLA-DMB	AB209577
A_24_P78230	NEDD4L	NM_001144967
A_23_P17356	GDAP1L1	NM_024034
A_32_P24585	SH3PXD2B	NM_001017995
A_23_P10771	MARK4	NM_031417
A_24_P37629	HNRNPUL1	NM_007040
A_32_P74955	ARID2	NM_152641
A_32_P79400	RPS18	NM_022551
A_33_P33406	KRTAP19-8	NM_001099219
A_33_P33949	P4HA2	NM_001017973
A_23_P31188	L3MBTL3	NM_032438
A_24_P93688	CALD1	AK022222
A_24_P39388	TPM3	NM_001043352
A_23_P39632	MPDZ	NM_003829
A_24_P11244	ENTPD7	NM_020354
A_24_P40562	NISCH	NM_007184
A_23_P21026	ATG16L1	NM_030803
A_24_P19670	MLL5	NM_182931
A_23_P15648	CD2AP	NM_012120
A_24_P16197	ATP11A	NM_015205
A_23_P20368	PRKRIR	ENST00000260045
A_23_P33539	ADCY2	NM_020546
A_24_P35549	LHPP	NM_022126
A_23_P16366	WFIKKN1	NM_053284
A_23_P91669	INPP5J	NM_001002837
A_23_P20275	C11orf54	NM_014039
A_23_P86635	DCLRE1C	NM_001033858
A_33_P33031	MRGPRG	NM_001164377
A_23_P15434	HIBCH	NM_014362
A_23_P56022	ZNF529	NM_020951
A_24_P67375	TPMT	NM_000367
A_23_P25246	AVPR1A	NM_000706
A_23_P32071	COG4	NM_015386
A_23_P33546	GMCL1	NM_178439
A_33_P33207	ATXN1	NM_000332
A_23_P38987	CNOT3	NM_014516

A_32_P80888	GMPPB	AK097846
A_23_P31108	ZNF281	NM_012482
A_24_P31627	KCNB1	NM_004975
A_23_P99802	FLRT2	NM_013231
A_23_P60899	TGS1	NM_024831
A_32_P31413	CSNK1G1	NM_022048
A_23_P25274	ZNF195	NM_007152
A_23_P38813	ZNF320	NM_207333
A_24_P61537	CKB	NM_001823
A_23_P12268	LPPR5	NM_001037317
A_24_P15192	TMEM97	NM_014573
A_32_P47782	ZNF233	NM_181756
A_23_P99303	SPATS2	NM_023071
A_23_P35659	SLC6A15	NM_018057
A_24_P53241	MYOCD	NM_153604
A_32_P19551	IQSEC3	NM_015232
A_24_P39879	C14orf102	AK024113
A_33_P34167	MAFG	NM_002359
A_24_P18503	FTSJD1	NM_018348
A_23_P11945	DPF1	NM_004647
A_23_P21891	FGF2	NM_002006
A_23_P1280	ASCC1	NM_015947
A_32_P37735	ARID3C	NM_001017363
A_23_P36781	SNX29	NM_001080530
A_32_P17801	DNM3	NM_015569
A_23_P16014	GALE	NM_000403
A_23_P37172	GJA5	NM_005266
A_23_P93881	SYPL1	NM_182715
A_23_P50174	PEX10	NM_153818
A_23_P845	TOMM40L	NM_032174
A_23_P11440	MORF4L2	NM_012286
A_23_P61967	RBMXL2	NM_014469
A_23_P34981	GTF2F2	X16901
A_23_P94766	ZNF354B	NM_058230
A_23_P98173	PTPRCAP	NM_005608
A_23_P37711	MCC	NM_002387
A_23_P53217	EED	NM_152991
A_23_P90150	PIH1D1	NM_017916
A_23_P23894	DSTYK	NM_015375
A_32_P42592	MCTS1	NM_014060
A_23_P42302	HLA-DQA2	NM_020056
A_23_P23669	PALMD	NM_017734
A_23_P92453	GSX2	NM_133267
A_23_P16414	PSME3	NM_176863
A_24_P92362	XPNPEP3	ENST00000357137
A_23_P97410	RFX5	NM_000449
A_23_P30187	MED27	NM_004269
A_23_P14930	HIST3H2A	NM_033445
A_23_P16276	DOCK9	NM_015296

A_24_P93006	CXXC5	NM_016463
A_23_P27048	TMEM199	NM_152464
A_24_P49514	PLEKHM1	NM_014798
A_23_P21001	PTPN18	NM_014369
A_23_P11026	C4orf31	NM_024574
A_23_P89123	CFDP1	NM_006324
A_23_P16664	ARL6IP5	NM_006407
A_33_P33065	ISYNA1	NM_016368
A_23_P7560	IL12B	NM_002187
A_23_P20165	MYCBP	NM_012333
A_23_P69537	NMU	NM_006681
A_33_P33068	ZNF806	XM_002345581
A_32_P92020	ENST0000031	ENST00000318245
A_24_P13804	CENPT	NM_025082
A_23_P67284	ZNF443	NM_005815
A_23_P68669	CHODL	NM_024944
A_24_P40624	PMS2L2	BC010535
A_23_P15328	ZNF234	NM_006630
A_23_P13913	STX3	NM_004177
A_23_P21021	EPAS1	NM_001430
A_32_P36343	SSC5D	NM_001144950
A_24_P37879	HAPLN4	NM_023002
A_23_P19226	DSE	NM_013352
A_32_P22401	MAP7D1	NM_018067
A_32_P10251	LOC541473	NR_003602
A_32_P33628	RELL1	NM_001085399
A_24_P69219	GOT2	NM_002080
A_23_P24594	HSPA8	NM_006597
A_23_P50098	PAX7	NM_013945
A_24_P42527	SCAMP1	NM_004866
A_23_P15581	NCAPG	NM_022346
A_23_P39871	MYT1L	NM_015025
A_33_P32476	ZNF835	NM_001005850
A_33_P33939	MTMR11	NM_181873
A_33_P33755	MIER3	NM_152622
A_23_P36142	B3GAT3	NM_012200
A_24_P14122	LMX1B	NM_002316
A_23_P39118	KLF16	NM_031918
A_23_P33644	OSBP	NM_002556
A_23_P16606	IDH3B	NM_174856
A_23_P20693	PMM2	NM_000303
A_24_P34205	GRAMD1B	AB033027
A_32_P32942	SMURF1	NM_020429
A_24_P20753	SRA1	NM_001035235
A_23_P86610	ZNF365	NM_014951
A_23_P13188	SOX12	U35612
A_33_P33465	OR2W5	NM_001004698
A_32_P85203	TMEM204	ENST00000253934
A_23_P14353	WDR4	NM_033661

A_23_P38998	TLX2	NM_016170
A_23_P37110	D4S234E	NM_014392
A_32_P16396	NEGR1	NM_173808
A_23_P80062	TAF4	NM_003185
A_23_P43262	SLC25A40	NM_018843
A_23_P41503	ARL8A	NM_138795
A_24_P10302	PCBP1	NM_006196
A_24_P18795	ARMC8	NM_213654
A_23_P40718	PARVB	NM_001003828
A_24_P31527	MBD3	NM_003926
A_32_P16928	MTF1	NM_005955
A_24_P20021	UPK1B	NM_006952
A_24_P56990	ELP2	ENST00000358232
A_23_P21033	HSPC159	NM_014181
A_33_P32280	SCPEP1	NM_021626
A_23_P29046	CBR1	NM_001757
A_23_P20884	SIRT6	NM_016539
A_23_P13916	CD6	NM_006725
A_23_P73661	TRAPPC2	NM_001011658
A_33_P33719	IGSF1	NM_205833
A_32_P13861	KIAA2018	NM_001009899
A_32_P24709	ZNF642	NM_198494
A_23_P25825	ENOX2	NM_182314
A_23_P30680	CITED4	NM_133467
A_23_P14301	ARID5A	NM_212481
A_23_P31989	SETD1B	NM_015048
A_23_P76882	CCNB1IP1	NM_182851
A_23_P43297	SERPINB13	NM_012397
A_32_P17192	MACC1	NM_182762
A_23_P98477	CCDC15	NM_025004
A_23_P42530	SUFU	NM_016169
A_23_P87899	DYRK4	NM_003845
A_23_P14070	C15orf23	NM_001142761
A_24_P19350	C16orf63	NM_144600
A_33_P32129	ZFHX2	NM_033400
A_23_P34868	PRICKLE3	NM_006150
A_33_P33888	LRPPRC	NM_133259
A_23_P21369	NRG2	NM_013982
A_33_P34150	ACLY	NM_001096
A_23_P51202	ZNF436	NM_001077195
A_23_P25187	KRT81	NM_002281
A_33_P32356	THOC2	NM_001081550
A_23_P64828	OAS1	NM_002534
A_23_P23869	LHX4	NM_033343
A_33_P32845	CD14	NM_001174104
A_23_P2745	GJB6	NM_006783
A_23_P10331	LCE1A	NM_178348
A_24_P10528	SFPQ	NM_005066
A_23_P12755	LOXL4	NM_032211

A_32_P341071	AGER	NM_001136
A_23_P18559	INPP4B	NM_003866
A_23_P374671	PITPNC1	NM_012417
A_33_P328615	TNFRSF4	NM_003327
A_24_P95007	GTF2I	NM_032999
A_33_P339421	ZNF133	NM_003434
A_24_P392146	KIAA1632	NM_020964
A_32_P137035	TRIM27	NM_006510
A_23_P162425	GOLT1B	NM_016072
A_23_P42335	FANCE	NM_021922
A_23_P115726	SLC16A9	NM_194298
A_23_P165331	BIN1	NM_139346
A_23_P251879	T	NM_003181
A_32_P343750	HMGB2	ENST00000296503
A_23_P81058	BMP3	NM_001201
A_32_P106376	VGLL2	NM_182645
A_23_P204958	LATS2	NM_014572
A_23_P115872	CEP55	NM_018131
A_33_P329031	PARP1	NM_001618
A_33_P333586	WDR35	NM_001006657
A_23_P65401	CFL2	NM_021914
A_23_P216291	CPA6	NM_020361
A_23_P64837	SMAGP	NM_001031628
A_33_P321364	ERN2	NM_033266
A_32_P226212	ZFHX2	NM_033400
A_24_P37319	ACSL3	NM_004457
A_33_P328366	ATP1A3	NM_152296
A_23_P133165	TUBB4Q	NM_020040
A_33_P342005	ZNF415	NM_001136038
A_24_P200854	HOXA2	NM_006735
A_23_P34033	NONO	NM_007363
A_33_P368121	PREPL	NM_006036
A_23_P30338	CCNH	NM_001239
A_23_P169599	LMAN2	NM_006816
A_32_P768527	BHLHE22	NM_152414
A_23_P160720	BATF3	NM_018664
A_23_P58036	MCCC1	NM_020166
A_32_P382525	STS	NM_000351
A_23_P89963	ZNF121	NM_001008727
A_24_P409013	WDR78	NM_024763
A_33_P340595	BCL9L	NM_182557
A_23_P29378	RNF13	NM_007282
A_23_P122464	ZNF193	NM_006299
A_23_P99883	PDIA3	NM_005313
A_23_P304016	LOC10013216	BC006438
A_32_P120084	CCDC59	NM_014167
A_23_P129786	SREBF1	NM_001005291
A_23_P30050	SLC30A9	NM_006345
A_23_P9574	ECT2	NM_018098

A_23_P75622	ATP5L	NM_006476
A_32_P80455	RORB	BX647070
A_23_P16118	ZDHHC6	NM_022494
A_23_P13899	GAPDH	NM_002046
A_23_P10798	SULT2B1	NM_004605
A_23_P17192	RAPGEF4	NM_007023
A_23_P34716	MTUS1	NM_001001924
A_23_P11331	TCEAL8	NM_153333
A_23_P9496	LHX3	NM_014564
A_24_P37728	IL31RA	NM_139017
A_32_P52825	SETD1B	NM_015048
A_23_P93464	BCKDHB	NM_000056
A_23_P41143	APEH	NM_001640
A_32_P33572	CLYBL	NM_206808
A_33_P32945	CD44	NM_000610
A_23_P43547	TMPRSS13	NM_001077263
A_23_P41655	PDE4D	NM_006203
A_33_P32650	GP1BB	NM_000407
A_32_P42453	C18orf45	NM_032933
A_23_P14827	MAGT1	NM_032121
A_23_P14783	EPHA5	NM_004439
A_24_P25564	IFT80	NM_020800
A_24_P30268	ARHGEF4	NM_015320
A_23_P10310	MFNG	NM_002405
A_24_P4816	GABARAPL1	NM_031412
A_24_P63537	ERAP1	NM_016442
A_23_P16125	SFXN2	NM_178858
A_33_P33783	GUCY1B2	CR615161
A_23_P55388	KLHL11	NM_018143
A_23_P28139	SCTR	NM_002980
A_23_P13962	GNS	NM_002076
A_23_P25771	CYP51A1	NM_000786
A_24_P37261	APBB1	NM_001164
A_23_P16613	XRN2	NM_012255
A_23_P20282	PDLIM2	NM_021630
A_23_P96080	H1FX	NM_006026
A_23_P21136	GNAZ	NM_002073
A_24_P91580	HNMT	NM_001024074
A_23_P70454	HIST1H1A	NM_005325
A_23_P50094	TNFRSF11A	NM_003839
A_23_P41675	ZNF610	NM_173530
A_23_P15347	ZNF763	NM_001012753
A_23_P40228	LNX2	NM_153371
A_24_P23966	BCKDHB	NM_183050
A_23_P74446	KDM4A	NM_014663
A_32_P23261	RUNDC2B	AK023827
A_33_P32682	GDI2	NM_001494
A_33_P32566	TTF2	NM_003594
A_23_P20986	TLX2	NM_016170

A_23_P21453[illegible]	ZNF323	NM_030899
A_23_P8473	SSBP3	NM_001009955
A_23_P38144[illegible]	SP2	NM_003110
A_23_P52885	C11orf61	NM_024631
A_24_P31989[illegible]	SMYD1	NM_198274
A_32_P19990[illegible]	RIPPLY1	NM_138382
A_33_P32137[illegible]	KCNK7	NM_005714
A_33_P32643[illegible]	DNM3	NM_001136127
A_24_P30448[illegible]	KLHDC5	NM_020782
A_23_P23630	ACAP3	NM_030649
A_24_P34618[illegible]	FAM120B	ENST00000476287
A_23_P21410[illegible]	TPMT	NM_000367
A_24_P18446[illegible]	ARL6IP4	NM_018694
A_23_P13333[illegible]	MRPS27	NM_015084
A_23_P99249	SUCLG2	NM_003848
A_23_P43611[illegible]	HMG20A	NM_018200
A_33_P33403[illegible]	ZNF768	NM_024671
A_24_P71938	SMAD1	NM_005900
A_23_P25984	SETD3	NM_032233
A_24_P91167[illegible]	SOX4	NM_003107
A_23_P20799[illegible]	PMAIP1	NM_021127
A_23_P23318	WDR3	NM_006784
A_33_P32461[illegible]	HPCA	NM_002143
A_33_P33941[illegible]	ZNF324B	NM_207395
A_23_P11842[illegible]	MAP2K3	NM_145109
A_23_P16719[illegible]	SEPSECS	NM_016955
A_23_P65823	GABARAPL3	NR_028287
A_24_P38528[illegible]	ALDH9A1	NM_000696
A_23_P30905[illegible]	FLG2	NM_001014342
A_33_P34213[illegible]	ZNF782	NM_001001662
A_23_P27571	MCOLN1	NM_020533
A_23_P43173[illegible]	SLC25A37	AK093931
A_23_P11060[illegible]	MAML1	NM_014757
A_33_P32585[illegible]	REXO1L1	NM_172239
A_23_P11600[illegible]	MAPK8IP1	NM_005456
A_23_P82674	GBAS	NM_001483
A_23_P32217	TOPORS	NM_005802
A_23_P34131[illegible]	AES	NM_198969
A_23_P82449	DFNA5	NM_004403
A_32_P54043[illegible]	ZBTB12	NM_181842
A_23_P34690	LMX1A	NM_177398
A_23_P25601[illegible]	CHIC2	NM_012110
A_23_P15597[illegible]	EGF	NM_001963
A_33_P32267[illegible]	SOX18	NM_018419
A_23_P23175	SNIP1	NM_024700
A_33_P33400[illegible]	GINS1	NM_021067
A_23_P52058	TARBP1	NM_005646
A_23_P12324[illegible]	RBAK	NM_021163
A_23_P67332	NR1H2	NM_007121

A_32_P10002	TUBA3D	NM_080386
A_23_P17318	NKAIN4	NM_152864
A_33_P32926	SNX6	NM_021249
A_24_P94014	C2CD2	NM_015500
A_23_P43494	ILF3	NM_004516
A_23_P51231	RUNX3	NM_001031680
A_33_P33920	TP53I3	NM_004881
A_23_P25086	TSC22D4	NM_030935
A_24_P18190	HSPA5	NM_005347
A_33_P33379	FIG4	NM_014845
A_23_P20243	ADD3	NM_016824
A_24_P29312	ZNF576	NM_024327
A_33_P32379	AP4M1	NM_004722
A_23_P50046	COL2A1	NM_001844
A_23_P15045	E4F1	NM_004424
A_23_P13159	BMP10	NM_014482
A_23_P13586	NOL4	NM_003787
A_32_P92281	LOC10013283	NR_028058
A_23_P38855	ZKSCAN1	NM_003439
A_24_P93595	CASZ1	NM_001079843
A_23_P40551	SCAI	NM_173690
A_23_P6624	UMPS	NM_000373
A_23_P37422	SSX7	NM_173358
A_33_P32915	PAX7	NM_001135254
A_32_P98423	C5orf13	NM_004772
A_24_P69150	RNF14	NM_004290
A_23_P37828	IKZF4	NM_022465
A_23_P15446	AGBL5	NM_001035507
A_23_P95489	STAG1	NM_005862
A_23_P15786	REXO4	NM_020385
A_23_P4551	SETBP1	NM_015559
A_23_P30270	NR1D2	NM_005126
A_33_P33383	MIDN	NM_177401
A_33_P33045	RNF207	AK128246
A_33_P32732	ZNF320	NM_207333
A_23_P14954	HIST2H2BE	NM_003528
A_33_P34138	C7orf60	NM_152556
A_24_P11098	AKT3	ENST00000366539
A_23_P21616	PSD3	NM_015310
A_32_P30588	SH3TC2	NM_024577
A_32_P64919	DIAPH3	NM_001042517
A_23_P65189	PDX1	NM_000209
A_33_P32867	SEC14L2	NM_033382
A_24_P36408	SERGEF	NM_012139
A_33_P33491	TTLL1	NM_012263
A_23_P48771	C14orf159	NM_024952
A_32_P50683	HSD17B8	NM_014234
A_23_P42395	FBXL18	NM_024963
A_23_P13069	KLK8	NM_144505

A_23_P20524:	RCOR1	NM_015156
A_23_P31477	NUDCD3	NM_015332
A_24_P74571	CBY1	NM_001002880
A_32_P75581	BHLHE22	NM_152414
A_23_P25787:	DAB2	NM_001343
A_23_P14505:	TUBE1	NM_016262
A_33_P33722:	MRS2	NM_020662
A_23_P21391(	MSX2	NM_002449
A_33_P37842!	PAK1	NM_001128620
A_23_P42055:	CIT	NM_007174
A_23_P21713!	ZNF275	NM_001080485
A_24_P20503(	ZNF676	NM_001001411
A_23_P13658!	BRDT	NM_207189
A_33_P33858:	HBCBP	XR_110362
A_24_P94319	C1orf177	NM_152607
A_23_P20890(	SEMA6B	NM_032108
A_24_P39894	ZNF516	NM_014643
A_23_P13657:	ST3GAL5	NM_003896
A_23_P41888	AGXT2L2	NM_153373
A_23_P65789	MCTP2	NM_018349
A_33_P32519:	MFSD1	NM_022736
A_32_P49436:	ATG2A	NM_015104
A_32_P14196!	NTRK3	ENST00000394480
A_23_P13906(	RNF141	NM_016422
A_23_P63751	PRDX3	NM_006793
A_33_P32937(	KRTAP10-8	NM_198695
A_23_P47863	TM7SF3	NM_016551
A_23_P30776:	CABLES2	NM_031215
A_32_P38409:	DNMBP	NM_015221
A_33_P33799:	GLS	NM_014905
A_24_P65842:	NFIB	NM_005596
A_23_P14776·	KCNH4	NM_012285
A_23_P42033:	PGBD4	NM_152595
A_23_P43910	STAP2	NM_001013841
A_24_P6903	ACTBL2	NM_001017992
A_23_P49975	KRT10	NM_000421
A_23_P16382(	ZNF629	NM_001080417
A_33_P33892!	ZNF30	NM_001099438
A_32_P83256	IRAK3	NM_007199
A_24_P28257:	SSR2	NM_003145
A_23_P31389	TRA2A	NM_013293
A_23_P75741	UBE2L6	NM_198183
A_23_P12101(	CSRNP1	NM_033027
A_23_P79199	DBI	NM_020548
A_33_P32405:	TUBE1	NM_016262
A_23_P37110(	D4S234E	NM_014392
A_24_P18536:	ZSCAN4	NM_152677
A_32_P13285	TNFAIP2	CB053075
A_23_P20307!	USP28	NM_020886

A_24_P94085:	STXBP6	AK096957
A_23_P21064!	TH1L	NM_198976
A_32_P86028	RPS13	NM_001017
A_23_P99302	SPATS2	NM_023071
A_23_P29763	WWTR1	NM_015472
A_23_P16273!	TSC22D1	NM_183422
A_23_P36079:	NTF3	NM_002527
A_24_P45087(	ZNF813	ENST00000396403
A_24_P94273(	ZSCAN29	NM_152455
A_23_P35316	ZNF695	NM_020394
A_23_P24535	TTC12	NM_017868
A_32_P19073:	FNIP2	AK057981
A_23_P38884(	RQCD1	NM_005444
A_23_P654	ZBTB17	NM_003443
A_33_P32975:	ANKRD18A	XM_001713970
A_23_P16685!	LIMD1	NM_014240
A_23_P12200:	C5orf30	NM_033211
A_23_P14243	TFDP1	NM_007111
A_23_P73750	ARMCX2	NM_014782
A_23_P33595!	GPR155	NM_001033045
A_23_P14196(	SERTAD3	NM_203344
A_33_P33612!	NOP16	NM_016391
A_23_P93372	RING1	NM_002931
A_23_P13960-	CRADD	NM_003805
A_23_P30003:	PDGFRA	NM_006206
A_24_P58549	ZNF273	NM_021148
A_23_P56894	CYP20A1	NM_177538
A_23_P49464	LOC81691	NM_030941
A_23_P12231	ACAP3	NM_030649
A_23_P83453	SMARCC1	NM_003074
A_24_P63262	RPN1	NM_002950
A_23_P20122	ZC3HAV1	NM_024625
A_23_P21681!	CDKN2B	NM_004936
A_23_P29495	CTNNB1	NM_001904
A_23_P82108	ZBTB2	NM_020861
A_23_P76829	HOMEZ	NM_020834
A_23_P31459:	NFYB	NM_006166
A_24_P50543	TRIM69	BC031266
A_23_P20442:	PPP1CC	NM_002710
A_23_P25726	NKX2-8	NM_014360
A_33_P32116:	PRKRIR	NM_004705
A_23_P21695!	RABGAP1	NM_012197
A_23_P41755	PURA	NM_005859
A_23_P32502!	MAFB	NM_005461
A_23_P26895	TUBD1	NM_016261
A_23_P34707(	PAG1	NM_018440
A_24_P19804-	ZNF483	NM_133464
A_33_P33472!	HNMT	NM_001024075
A_24_P24494-	MCTP2	NM_018349

A_23_P10103{	KRTAP9-3	NM_031962
A_23_P50837	MBD3	NM_003926
A_23_P94980	POLA1	NM_016937
A_23_P29697	ENTPD3	NM_001248
A_24_P10785!	SPRED1	NM_152594
A_32_P17655(	JMY	NM_152405
A_33_P32346(	ZKSCAN1	NM_003439
A_23_P31354:	GRK1	NM_002929
A_23_P12044:	NCOA3	NM_181659
A_24_P21588:	ARHGDIG	NM_001176
A_24_P32394‹	PHTF2	NM_020432
A_24_P16913	ABCC4	NM_005845
A_23_P94118	GTF2E2	NM_002095
A_23_P38271	MYH2	NM_017534
A_32_P25602	GCSH	NM_004483
A_23_P16911	RRAGA	NM_006570
A_24_P19243‹	TERF1	NM_017489
A_23_P59602	MIOS	NM_019005
A_24_P35738(	KRTAP13-3	NM_181622
A_33_P32312!	CREG1	NM_003851
A_24_P12456	ORMDL2	NM_014182
A_23_P64129	HTATIP2	NM_006410
A_23_P1254	GSTO1	NM_004832
A_23_P40954:	POLR1D	NM_152705
A_23_P99762	RBM25	NM_021239
A_32_P73828:	LOC728690	AK094642
A_23_P38342:	NFKBID	NM_139239
A_23_P25797:	AKR1C1	NM_001353
A_24_P33675!	MCL1	NM_021960
A_24_P24480(	NDRG2	NM_201535
A_23_P13189!	SDCBP2	NM_080489
A_24_P10038:	GK	NM_203391
A_23_P56930	RTN4	NM_020532
A_23_P90456	KRTDAP	NM_207392
A_23_P27334	ZFP161	NM_003409
A_24_P11275(	TFCP2	NM_005653
A_32_P29611	ZNF468	NM_199132
A_23_P15351‹	MED25	NM_030973
A_23_P21069(	TRIB3	NM_021158
A_23_P87917	KCNH3	NM_012284
A_23_P13450:	FZD1	NM_003505
A_33_P33471(	TBL1X	NM_005647
A_23_P11962	NDUFA13	NM_015965
A_32_P55135	SSX1	NM_005635
A_23_P67213	SH2D3A	NM_005490
A_23_P51051	ZNF142	NM_001105537
A_23_P21305(	HPGD	NM_000860
A_24_P25408{	ZNF440	NM_152357
A_23_P12357{	PRRC2B	NM_013318

A_24_P27074	ZFP1	NM_153688
A_23_P43443(	ZNF439	NM_152262
A_23_P11765	MYO5A	NM_000259
A_23_P16381(	ZNF629	NM_001080417
A_24_P48810!	ETNK1	NM_018638
A_23_P14137(	PTRF	NM_012232
A_23_P29499	CTNNB1	NM_001904
A_23_P16248(	PTPN6	NM_002831
A_32_P18322	ZNF367	NM_153695
A_33_P33507	ZNF699	NM_198535
A_23_P3186	CCNK	NM_001099402
A_23_P26890	MMP28	NM_024302
A_23_P38958	TCF7L2	NM_030756
A_23_P31721	E2F5	NM_001951
A_23_P25389(	NPNT	NM_001033047
A_24_P36756	SLC35B4	NM_032826
A_23_P25558	ATF7IP	NM_018179
A_33_P32397	WDR44	NM_019045
A_23_P82748	ENY2	NM_020189
A_23_P25106	WNT1	NM_005430
A_23_P42893	SPRR1B	NM_003125
A_24_P12218	NDUFA2	NM_002488
A_23_P59718	SRI	NM_003130
A_23_P49724	GJC1	NM_005497
A_23_P43898	EPHX4	NM_173567
A_23_P40955	PPM1A	NM_177951
A_23_P15619	PHF15	NM_015288
A_33_P32518	APBB2	NM_004307
A_23_P41827	WBSCR28	NM_182504
A_23_P98282	SPTBN2	NM_006946
A_23_P13978	IRAK4	NM_016123
A_23_P53588	WNT5B	NM_030775
A_32_P23253(	VPS13A	NM_015186
A_24_P28200(	NUP210	NM_024923
A_23_P95060	EPHB3	NM_004443
A_23_P30152	KIAA1462	NM_020848
A_23_P97932	MSRB2	NM_012228
A_23_P30622	ARX	NM_139058
A_23_P75310	ARHGAP22	NM_021226
A_23_P13757	FBXO28	NM_015176
A_23_P13555	CACHD1	NM_020925
A_23_P32374	PIF1	NM_025049
A_33_P35312(	PCOTH	NM_001014442
A_23_P21685(	RNF20	NM_019592
A_23_P16962	SHMT2	NM_005412
A_24_P39688	NDUFA10	ENST00000252711
A_23_P74688	ATAD3B	NM_031921
A_33_P33728!	DDX54	NM_001111322
A_23_P95287	NEDD4	NM_198400

A_24_P73741(	ESD	CR600585
A_33_P33384	SGK494	NM_001174103
A_23_P40962	PPFIBP2	NM_003621
A_23_P13287	C3orf26	NM_032359
A_32_P12610	E2F6	NM_198256
A_23_P30972	ASCC3	NM_022091
A_24_P25453	PGK1	NM_000291
A_24_P30124(	NEDD8	NM_006156
A_23_P20443	LZTS1	NM_021020
A_24_P13456	RP2	NM_006915
A_23_P4332	KRT14	NM_000526
A_23_P31422	LEO1	NM_138792
A_23_P10476	YAP1	NM_006106
A_24_P15890	IRAK4	NM_016123
A_23_P38174(	ASXL3	NM_030632
A_32_P23460	PFN1	NM_005022
A_23_P30689(	POLI	NM_007195
A_23_P12282	DYRK3	NM_001004023
A_23_P32083	KCNV2	NM_133497
A_24_P38800	CHRNB2	ENST00000368476
A_23_P15005	MMRN2	NM_024756
A_23_P11169	MAFK	NM_002360
A_32_P4005	TMED10	NM_006827
A_33_P36365	SUCLG2	NM_003848
A_32_P12023(	RPL17	NM_000985
A_33_P34068(	NFASC	NM_001005388
A_23_P11992	CNNM4	NM_020184
A_24_P80380	GTF2I	NM_032999
A_24_P36395	RAB11FIP2	NM_014904
A_23_P15255	BZRAP1	AF039571
A_23_P36144	SESN3	NM_144665
A_23_P12700	THNSL1	NM_024838
A_33_P33464	PHF21A	NM_001101802
A_23_P95705	MCC	NM_002387
A_23_P95838	CDH13	NM_001257
A_32_P58936	MBTD1	NM_017643
A_23_P28953	DNMT3B	NM_175850
A_24_P91649(	PRKCA	NM_002737
A_23_P23564	PIP5K1A	NM_003557
A_24_P19665	TOM1L1	NM_005486
A_23_P12945	SDR42E1	NM_145168
A_33_P32160(	SKA3	NM_145061
A_33_P33046(	ZMIZ1	NM_020338
A_23_P33141	UTF1	NM_003577
A_24_P69973	SARS	NR_034072
A_23_P85519	ZRANB2	NM_203350
A_33_P33331	RAP1GAP2	NM_015085
A_23_P12178	CRTC2	NM_181715
A_23_P10407	S100A3	NM_002960

A_24_P26513	UBE3C	NM_014671
A_23_P20437	TIGD5	NM_032862
A_23_P13460	WASL	NM_003941
A_33_P32576	PPP1R12B	NM_001167858
A_33_P33662	NTNG1	NM_014917
A_23_P20687	RRN3	NM_018427
A_23_P20173	TRAF5	NM_004619
A_24_P98914	PFKM	NM_000289
A_24_P24860	ACSL3	NM_004457
A_23_P15051	MED19	NM_153450
A_33_P33317	GDAP1L1	NM_024034
A_23_P20556	PRKCH	NM_006255
A_23_P14064	ANKDD1A	NM_182703
A_23_P27490	DNMT1	NM_001379
A_24_P28712	HSPA8	NM_006597
A_23_P21097	SCAND1	NM_016558
A_33_P33671	C1orf152	NR_003242
A_23_P25529	HHATL	NM_020707
A_32_P41924	FAM119B	NM_015433
A_33_P32978	AKNA	NM_030767
A_23_P16788	IL17F	NM_052872
A_23_P32455	ANO4	NM_178826
A_23_P39074	C16orf63	NM_144600
A_23_P38802	CRY2	NM_021117
A_23_P12626	HLX	NM_021958
A_23_P12634	BARHL2	NM_020063
A_23_P20410	TBX3	NM_016569
A_23_P36336	RXFP3	NM_016568
A_23_P13307	TBCK	NM_033115
A_33_P32849	MCM5	NM_006739
A_23_P84651	CNBP	NM_003418
A_23_P20997	VSNL1	NM_003385
A_33_P32204	TNFAIP8L1	NM_152362
A_23_P35855	LSMD1	NM_032356
A_24_P15932	GINS3	NM_022770
A_23_P20225	TFAM	NM_003201
A_23_P32068	VPS41	NM_014396
A_23_P34233	QPRT	NM_014298
A_24_P64071	ZBTB10	NM_023929
A_32_P31114	MORN4	NM_178832
A_23_P25994	LGMN	NM_001008530
A_23_P39613	NKX2-5	NM_004387
A_23_P31829	EBF2	NM_022659
A_23_P75589	CRYAB	NM_001885
A_23_P21624	TOP1MT	NM_052963
A_23_P78734	MYH14	NM_001077186
A_24_P49724	MALAT1	NR_002819
A_23_P73708	ZNF75D	NM_007131
A_23_P38197	OGT	NM_181672

A_23_P87860	MYF5	NM_005593
A_33_P339365	PLEKHA5	NM_019012
A_23_P251795	GPC2	NM_152742
A_23_P86386	ZNF669	NM_024804
A_23_P359891	JHDM1D	NM_030647
A_33_P333474	RAPGEF2	NM_014247
A_23_P16143	GTF2F1	NM_002096
A_23_P27638	ZNF700	NM_144566
A_24_P42066	GGT3P	NR_003267
A_23_P100651	ZBTB4	NM_020899
A_32_P131361	RNF13	NM_007282
A_23_P500936	FOXA2	NM_021784
A_23_P127676	CTR9	NM_014633
A_33_P334440	MEF2B	NM_001145785
A_23_P436259	ERN1	AK055561
A_24_P369154	PPP6C	NM_002721
A_23_P135315	ZBTB43	NM_014007
A_23_P202138	SFXN3	NM_030971
A_23_P56734	HNMT	NM_006895
A_23_P431769	ETV4	NM_001079675
A_32_P199301	TFDP1	NM_007111
A_23_P101875	FBXO17	NM_024907
A_23_P424	MARK1	NM_018650
A_23_P118631	SPATA20	NM_022827
A_23_P214661	RPS18	NM_022551
A_24_P283535	ARHGEF10	NM_014629
A_23_P302038	POU4F2	NM_004575
A_23_P97283	PAQR6	NM_024897
A_23_P406971	STAT5A	NM_003152
A_33_P327560	PLA2G4E	NM_001080490
A_24_P33895	ATF3	NM_001040619
A_32_P500111	LOC283398	XM_208658
A_23_P113245	RBPJ	D14041
A_23_P154330	TXNDC9	NM_005783
A_23_P363206	RBBP6	NM_006910
A_23_P107311	SDF2	NM_006923
A_24_P922919	ACP1	NM_001040649
A_24_P382461	SLC39A3	NM_144564
A_33_P332825	IL5RA	NM_175725
A_23_P96158	KRT17	NM_000422
A_23_P108641	FAM136A	NM_032822
A_23_P108244	COX6B1	NM_001863
A_23_P112874	GPC5	NM_004466
A_24_P941166	ZNF425	NM_001001661
A_24_P159312	SLC7A6	NM_001076785
A_24_P366555	BBS9	BC032715
A_23_P26037	FRMD5	NM_032892
A_23_P8293	SCML4	NM_198081
A_32_P232704	MTERF	AL832861

A_24_P94116	APOL6	NM_030641
A_23_P43133	CRIPAK	NM_175918
A_24_P34770	CTCF	NM_006565
A_23_P21253	CHMP2B	NM_014043
A_33_P33548	COQ10B	NM_025147
A_24_P24760	PCMT1	NM_005389
A_33_P33618	NAA16	NM_024561
A_23_P21874	AGPAT3	NM_020132
A_24_P34155	RUNX1	NM_001122607
A_23_P43122	TBX6	NM_004608
A_23_P11810	NDUFB10	NM_004548
A_23_P39242	ZNF396	NM_145756
A_23_P12110	HESX1	NM_003865
A_23_P55601	ZNF236	NM_007345
A_24_P19166	GOLIM4	NM_014498
A_23_P38007	ZBTB38	NM_001080412
A_23_P21646	SLC1A1	NM_004170
A_23_P11446	TBL1Y	NM_033284
A_23_P21373	PCBD2	NM_032151
A_23_P10275	BTBD3	NM_014962
A_33_P32950	PTPRCAP	NM_005608
A_24_P36603	ECT2	NM_018098
A_23_P66402	MED9	NM_018019
A_23_P40799	CRAMP1L	NM_020825
A_24_P14178	ATP2A2	NM_001681
A_23_P75083	ZMYND11	NM_212479
A_23_P16335	PIAS1	NM_016166
A_32_P23286	BCR	NM_004327
A_24_P27464	GTF2H2D	NM_001042490
A_23_P20805	LPIN2	NM_014646
A_23_P6909	CCRL1	NM_178445
A_24_P29966	TRIM32	NM_012210
A_23_P32138	RNF19B	NM_153341
A_23_P20731	MAP3K14	NM_003954
A_24_P10791	CBFB	NM_022845
A_23_P20754	SYNRG	NM_007247
A_23_P39570	RSF1	NM_016578
A_23_P16676	IL17RC	NM_153461
A_23_P14103	COX4I1	NM_001861
A_23_P14112	GALK1	NM_000154
A_23_P37711	MCC	NM_002387
A_23_P10519	CAPN5	NM_004055
A_33_P33771	MAP3K5	NM_005923
A_23_P11843	SUMO2	NM_006937
A_23_P30315	TMEM87B	NM_032824
A_23_P7020	ADPRH	NM_001125
A_32_P70593	CHI3L2	AK057181
A_32_P15232	ZNF28	NM_006969
A_23_P56736	TUBA3D	NM_080386

A_33_P32693	HAUS5	NM_015302
A_32_P41515	BAZ2B	NM_013450
A_33_P34030	LCE5A	NM_178438
A_24_P31435	ZBTB33	NM_006777
A_23_P20083	KMO	NM_003679
A_33_P32937	KRTAP10-6	NM_198688
A_23_P13145	ST6GAL2	NM_032528
A_23_P12307	CAV2	NM_001233
A_23_P6312	E2F6	NM_198256
A_23_P16414	MLX	NM_170607
A_24_P12888	TBL1XR1	NM_024665
A_23_P25101	ELL2	NM_012081
A_24_P29885	NOV	NM_002514
A_23_P635	PMF1	NM_007221
A_24_P37890	PKNOX1	NM_004571
A_23_P21786	IFI16	NM_005531
A_23_P14049	ZNF592	NM_014630
A_32_P22338	PDCD6IP	NM_013374
A_23_P566	FOXJ3	NM_014947
A_23_P25901	BAP1	NM_004656
A_23_P67271	PKN1	NM_002741
A_23_P63897	CTBP2	NM_022802
A_23_P34205	RBBP6	NM_032626
A_23_P30126	FGFBP1	NM_005130
A_23_P25173	ATP11C	NM_001010986
A_23_P16624	RCAN1	NM_004414
A_23_P15037	MPZL2	NM_144765
A_23_P89746	RAX	NM_013435
A_23_P13048	ERCC2	NM_000400
A_24_P20562	TXLNA	NM_175852
A_23_P16428	CLDN7	NM_001307
A_24_P25624	SSX2	NM_175698
A_24_P31760	SLC37A3	NM_207113
A_23_P20949	PAX3	NM_181458
A_23_P16221	MANSC1	NM_018050
A_23_P84922	HDAC8	NM_018486
A_24_P91652	PAX5	U62539
A_33_P32605	SOX30	NM_007017
A_23_P16876	PTPRZ1	NM_002851
A_23_P15240	CAPNS2	NM_032330
A_24_P36745	ZEB2	NR_033258
A_23_P82370	HOXA6	NM_024014
A_23_P51136	RHOB	NM_004040
A_23_P42958	TMEM67	NM_153704
A_33_P33998	ZNF792	NM_175872
A_24_P37009	ZNF230	NM_006300
A_24_P12301	YWHAE	NM_006761
A_23_P59691	PAX4	AF043978
A_23_P46844	TRIM8	NM_030912

A_23_P59790	LUC7L2	NM_016019
A_23_P377218	HEXIM2	NM_144608
A_23_P56833	ASB3	NM_016115
A_23_P141429	ABI3	NM_016428
A_33_P33515[illegible]	IL1RAPL1	NM_014271
A_33_P333638	PAOX	NM_207128
A_24_P24671[illegible]	RSF1	NM_016578
A_23_P14928[illegible]	EPHA2	NM_004431
A_32_P10886	C6orf52	NM_001145020
A_24_P94482[illegible]	ATG7	NM_006395
A_24_P27653[illegible]	SNIP1	NM_024700
A_24_P30405[illegible]	GSTO1	NM_004832
A_23_P13364	NUCB2	NM_005013
A_23_P357760	ARSD	NM_009589
A_24_P171549	CDCA7	NM_031942
A_23_P157620	FAM160B2	NM_022749
A_23_P5092	FUT6	NM_000150
A_23_P500000	SCEL	NM_144777
A_23_P358928	SRI	NM_003130
A_24_P96403	RUNX1	NM_001001890
A_23_P209320	PER2	NM_022817
A_23_P71513	EFR3A	NM_015137
A_23_P21460[illegible]	FLOT1	NM_005803
A_24_P303390	EHMT2	NM_025256
A_23_P136405	PDCD1	NM_005018
A_23_P16334[illegible]	PIAS1	NM_016166
A_24_P11790[illegible]	OSBPL10	NM_017784
A_23_P11171[illegible]	MTERF	NM_006980
A_23_P20328[illegible]	QSER1	NM_001076786
A_23_P6116	SEC23B	NM_032985
A_23_P6596	HES1	NM_005524
A_32_P81801	ZNF738	NR_027130
A_23_P218408	GPS2	NM_004489
A_23_P11651[illegible]	PRR5L	NM_024841
A_32_P188568	FLJ45983	NR_024256
A_23_P152136	GINS3	NM_022770
A_23_P21073[illegible]	ADAM33	NM_025220
A_23_P62890	GBP1	NM_002053
A_23_P57137	C20orf29	NM_018347
A_23_P593	GPBP1L1	NM_021639
A_24_P51790[illegible]	HNRNPA1	NM_002136
A_23_P20679[illegible]	ZNF764	NM_033410
A_23_P412515	CLDN12	NM_012129
A_23_P201478	KLHL21	NM_014851
A_23_P11715[illegible]	SUCLA2	NM_003850
A_24_P12665[illegible]	TCEA1	NM_006756
A_23_P147238	WSB2	NM_018639
A_23_P4741	KHSRP	NM_003685
A_23_P162734	RNF6	NM_005977

A_24_P43876	C9orf40	NM_017998
A_24_P23726!	MAPK1	NM_002745
A_24_P40283(	ZNF141	NM_003441
A_32_P47551:	MYO15B	NR_003587
A_23_P32452:	IQCK	NM_153208
A_23_P83414	PPP1CB	NM_002709
A_23_P15001(	DUSP5	NM_004419
A_24_P32483{	IGHD	AK128568
A_23_P13696:	RPGR	NM_000328
A_24_P36438:	MMAB	NM_052845
A_32_P12866:	TMED5	ENST00000370282
A_23_P11372{	MTG1	NM_138384
A_23_P42522{	ZC3HAV1	NM_020119
A_33_P32456!	LOC10012837{	XR_112969
A_23_P81759	QKI	NM_006775
A_23_P88880	ZNF423	NM_015069
A_23_P25827(	RAP2B	NM_002886
A_33_P33063(	KRT26	NM_181539
A_23_P10312(	GTPBP1	NM_004286
A_23_P20067(	WDR78	NM_207014
A_23_P12874{	BDKRB1	NM_000710
A_24_P26534(	KRT14	NM_000526
A_33_P32577(	APOA1BP	NM_144772
A_23_P32251!	FOSL1	NM_005438
A_23_P66319	BCAR1	NM_014567
A_24_P13572	DLST	NM_001933
A_32_P29347	ZNF260	NM_001012756
A_32_P19040{	NCRNA00292	NR_027285
A_24_P19220!	ZNF92	NM_007139
A_23_P10307(	YWHAH	NM_003405
A_23_P21171:	ZIC4	AF332509
A_32_P51482{	SMYD4	NM_052928
A_23_P25620{	ABLIM3	NM_014945
A_32_P20560:	UGGT1	NM_020120
A_23_P16704(	PDIA5	NM_006810
A_23_P78061	LHX1	NM_005568
A_23_P31858	ST18	NM_014682
A_23_P14875:	PLEKHA6	NM_014935
A_33_P32174!	SLC5A2	NM_003041
A_33_P32327!	RAB11FIP1	NM_025151
A_23_P11714(	PRDM4	NM_012406
A_23_P4764	INSR	NM_000208
A_23_P20111(	AHCYL1	NM_006621
A_23_P15702:	ZNF786	NM_152411
A_33_P33096(	GPX4	NM_001039848
A_24_P36367!	ATP6V1H	NM_015941
A_33_P32730:	ALX4	NM_021926
A_32_P18247:	ZNF625	NM_145233
A_24_P32478:	ACVRL1	NM_000020

A_23_P15864	MBD2	NM_003927
A_24_P29484:	ATXN1	NM_000332
A_24_P10066	MKKS	NM_170784
A_24_P15737(	IL17RB	NM_018725
A_32_P82580!	LOC10013283.	NR_028058
A_24_P39751!	HMGN4	NM_006353
A_23_P21242:	XYLB	NM_005108
A_23_P56010	DMWD	NM_004943
A_33_P33750:	BRPF3	NM_015695
A_33_P33168(	AHR	NM_001621
A_32_P15733!	ZNF100	NM_173531
A_33_P35298!	KDM2B	NM_032590
A_33_P33145:	RAB34	NM_001142625
A_23_P61230	HGS	NM_004712
A_24_P83211:	NPM1	NM_001037738
A_23_P13590:	EBF1	NM_024007
A_23_P21034:	COX7A2L	NM_004718
A_23_P37979	PIGW	NM_178517
A_23_P12416(	GPC4	ENST00000370828
A_23_P20411!	RBM19	NM_001146699
A_24_P22287:	UGT1A6	NM_001072
A_32_P11961(	ETFA	NM_000126
A_23_P15076!	SLCO2B1	NM_007256
A_24_P35861!	ZNF99	NM_001080409
A_24_P17490:	NOL7	AK075301
A_32_P11837:	INTU	NM_015693
A_23_P25367:	TMEM192	NM_001100389
A_24_P27016(	OS9	NM_001017956
A_32_P10737:	GBP1	NM_002053
A_23_P12412:	PXMP2	NM_018663
A_23_P18266	TUSC4	NM_006545
A_24_P16363:	OXR1	NM_181354
A_23_P20301:	NPAT	NM_002519
A_23_P25559	ABCC4	NM_005845
A_32_P16627(	STX11	NM_003764
A_23_P18292	RPL14	NM_001034996
A_23_P11764	EIF2C1	NM_012199
A_24_P91443	GSTM3	NM_000849
A_24_P28275:	SUPT7L	NM_014860
A_23_P13807:	PRKACB	NM_207578
A_23_P10620	GSTZ1	NM_145870
A_23_P16199!	HPX	NM_000613
A_32_P17365:	POLA1	NM_016937
A_24_P38865	AIP	NM_003977
A_23_P46725	EPC1	NM_025209
A_32_P12544	SMAD2	NM_001003652
A_23_P12051:	C20orf141	NM_080739
A_24_P10220:	UBR1	NM_174916
A_32_P50005	DBT	NM_001918

A_24_P35146	KIAA1524	NM_020890
A_33_P33276	FCGR2C	NM_201563
A_24_P34920	LOC729915	XM_001722873
A_23_P39247	NR3C2	NM_000901
A_23_P34278	NPAS4	NM_178864
A_23_P16357	CENPT	NM_025082
A_23_P12783	ZNF214	NM_013249
A_23_P69452	DBR1	NM_016216
A_23_P15159	CPNE6	NM_006032
A_24_P15792	TNFAIP3	NM_006290
A_24_P14328	ZNF564	NM_144976
A_23_P54421	ZNF710	NM_198526
A_24_P11900	MYH15	NM_014981
A_23_P14452	ADAMTS3	NM_014243
A_23_P13926	SIRT3	NM_012239
A_23_P31839	CUGBP1	NM_198700
A_24_P71153	PAFAH2	NM_000437
A_32_P12056	MDFIC	NM_199072
A_23_P23705	SPATA6	ENST00000371847
A_23_P14417	CPOX	NM_000097
A_32_P15154	KRT18	NM_000224
A_23_P13897	GAPDH	NM_002046
A_23_P20312	CADM1	NM_014333
A_23_P40856	LRTM1	NM_020678
A_23_P14369	SOX10	NM_006941
A_23_P32054	CAMSAP1	NM_015447
A_23_P11602	MED17	NM_004268
A_24_P91157	GNB5	BC011671
A_24_P31165	GFAP	NM_002055
A_32_P11037	SLC8A1	NM_021097
A_24_P15249	LOC643699	ENST00000327271
A_32_P11743	CDYL2	NM_152342
A_24_P28300	DVL3	NM_004423
A_23_P16824	PRPF4B	NM_003913
A_23_P16961	PRKDC	NM_006904
A_33_P33838	TREX1	NM_016381
A_24_P29733	PFTK1	NM_012395
A_24_P39843	CYB561	NM_001017916
A_24_P30498	SAP30BP	NM_013260
A_23_P35943	NBPF14	NM_015383
A_23_P13011	ASGR2	NM_080912
A_24_P15066	RUNDC2A	NM_032167
A_24_P11676	ZNF207	ENST00000394670
A_24_P69092	SEC23B	NM_032985
A_24_P19135	KCMF1	NM_020122
A_23_P4416	ZNF287	NM_020653
A_23_P3146	SPTLC2	NM_004863
A_23_P53458	CORO1C	NM_014325
A_23_P88021	GPR180	NM_180989

A_23_P30630	MCM9	NM_017696
A_24_P17981(	SLC27A3	NM_024330
A_23_P67399	STRN4	NM_001039877
A_23_P21366:	PPIP5K2	NM_015216
A_23_P21787:	ARID1A	NM_006015
A_24_P23766:	NCOR1	NM_001190438
A_23_P13402(	C6orf35	NM_018452
A_23_P67529	KCNN4	NM_002250
A_23_P21129!	PKNOX1	NM_004571
A_33_P32846(	EID2	NM_153232
A_24_P16046(	SLC39A3	NM_144564
A_33_P32449:	DBI	NM_020548
A_23_P16345{	EHD4	NM_139265
A_23_P15836(	PUF60	NM_014281
A_23_P10433	INTS1	NM_001080453
A_24_P69095	ENC1	NM_003633
A_23_P20882	ATP6V1G1	NM_004888
A_24_P40681	FAM53B	NM_014661
A_23_P15831{	ROR2	NM_004560
A_24_P10468!	SERPINB13	NM_012397
A_23_P25141	CAND1	NM_018448
A_24_P18042:	PCBD2	ENST00000512783
A_23_P15382	MLLT1	NM_005934
A_23_P20179(	PPP1R12B	NM_002481
A_23_P12792(	WEE1	NM_003390
A_23_P21108:	IFNAR2	NM_207585
A_32_P21822{	FAM109B	NM_001002034
A_23_P40044	CAD	NM_004341
A_24_P67918	ZNF396	NM_145756
A_23_P26021	COPS2	NM_004236
A_33_P33578:	HTR1D	ENST00000314113
A_23_P37016:	XRRA1	NM_182969
A_24_P14488:	RNF6	NM_005977
A_32_P11434{	RNF5	NM_006913
A_23_P33282(	IL17A	NM_002190
A_32_P47747(	PLEKHM3	NM_001080475
A_23_P39125	GLTSCR2	NM_015710
A_23_P39611!	SSX7	NM_173358
A_33_P32365(	LCE2D	NM_178430
A_24_P28845	ZNF2	NM_021088
A_23_P92349	FGFRL1	NM_001004356
A_23_P85716	FCGR2A	NM_021642
A_24_P19651!	MTAP	NM_002451
A_24_P22216!	GAB1	AK074381
A_23_P17023:	CSTA	NM_005213
A_23_P13451	PURB	NM_033224
A_23_P31658:	ZNF441	NM_152355
A_24_P92096{	GEN1	NM_182625
A_32_P80068	CLEC2D	NM_001004419

A_24_P22079	FOXO1	NM_002015
A_23_P13619	TBC1D19	NM_018317
A_23_P12455	NBPF14	NM_015383
A_24_P21535	PRKCDBP	NM_145040
A_24_P27004	ASAM	NM_024769
A_23_P34319	XPNPEP2	NM_003399
A_23_P12476	MED24	NM_014815
A_23_P11563	TRDMT1	NM_004412
A_32_P61693	CACNG8	NM_031895
A_23_P65883	SLC12A6	NM_001042496
A_32_P13458	HMGB1	NM_002128
A_23_P25563	TRIM4	NM_033017
A_33_P34090	S100A1	NM_006271
A_23_P16362	AKTIP	NM_001012398
A_23_P9715	HS3ST4	NM_006040
A_24_P1929	DDX52	NM_007010
A_23_P21495	FCGBP	NM_003890
A_23_P39040	SMARCA4	NM_003072
A_24_P21372	KLHL20	BC005253
A_33_P33669	CHST6	NM_021615
A_24_P18438	LRTOMT	NM_145309
A_23_P63681	IDE	NM_004969
A_24_P30048	C7orf25	NM_024054
A_24_P60250	AGPHD1	NM_001013619
A_32_P38136	PABPC1	NM_002568
A_23_P11487	HOOK1	NM_015888
A_24_P37644	TAF5L	NM_001025247
A_23_P32088	CDC42SE1	NM_001038707
A_23_P55873	CC2D1A	NM_017721
A_23_P34977	HAUS5	NM_015302
A_32_P56451	ATP8B1	NM_005603
A_23_P32126	ME2	NM_002396
A_23_P35576	ZNF518A	NM_014803
A_23_P11053	FST	NM_013409
A_23_P21314	TRIM2	NM_015271
A_23_P13265	TLR9	NM_017442
A_23_P16726	C4orf43	NM_018352
A_23_P25417	ADNP	NM_015339
A_23_P34762	ERO1LB	NM_019891
A_24_P15046	SMOC1	NM_001034852
A_23_P32025	SLC35D2	NM_007001
A_23_P6344	SDF2L1	NM_022044
A_32_P46162	ZNF708	NM_021269
A_24_P17822	ZNF781	NM_152605
A_32_P33821	C5orf43	NM_001048249
A_23_P20577	GNB5	NM_016194
A_32_P18151	ZNF730	ENST00000327867
A_24_P12413	TRAM2	NM_012288
A_32_P20442	ZNF555	ENST00000334241

A_23_P15369	BTBD2	NM_017797
A_24_P40683	MTMR2	NM_201278
A_23_P54322	NR2E3	NM_014249
A_32_P45409	ZNF605	NM_183238
A_23_P10034	ORC6L	NM_014321
A_24_P20401	FOXR1	NM_181721
A_23_P16861	TSPAN13	NM_014399
A_23_P85604	WDR26	NM_025160
A_23_P12197	POU4F3	NM_002700
A_23_P21413	REV3L	NM_002912
A_23_P31265	CIRBP	NR_023312
A_23_P35236	ZBTB8A	NM_001040441
A_23_P14568	ACTL6B	NM_016188
A_32_P48842	LOC644538	NM_001163438
A_23_P20237	COX15	NM_078470
A_33_P33033	LPPR5	NM_001037317
A_23_P41720	TERF2	NM_005652
A_33_P32322	C12orf11	NM_018164
A_23_P63525	LCE2C	NM_178429
A_32_P80897	HSD17B1	BC033110
A_23_P14030	PSMA3	NM_002788
A_33_P32459	SHROOM1	NM_133456
A_23_P82356	BBS9	NM_198428
A_23_P81867	HIST1H2AH	NM_080596
A_33_P33562	ZNF416	NM_017879
A_23_P10255	MALL	NM_005434
A_23_P21092	CALB2	NM_001740
A_24_P68019	ZNF551	NM_138347
A_32_P11104	C4orf26	NM_178497
A_23_P70095	CD74	NM_001025158
A_23_P35843	FCRL3	NM_052939
A_32_P77362	SC4MOL	AV701505
A_23_P58353	HNRNPD	NM_031370
A_23_P77381	DOK4	NM_018110
A_23_P46441	PVRL4	NM_030916
A_24_P17169	STK32B	NM_018401
A_23_P38432	DENND4A	NM_001144823
A_23_P93383	RGL2	NM_004761
A_23_P14230	MKNK2	NM_017572
A_23_P69513	RGS12	NM_198229
A_23_P16354	ZNF382	NM_032825
A_23_P14346	KRTAP19-1	ENST00000390689
A_23_P11078	SCAMP1	NM_004866
A_23_P13810	MED18	NM_017638
A_23_P2683	RPAP3	NM_024604
A_23_P7582	TCF7	NM_003202
A_32_P34116	NUS1	NM_138459
A_24_P93703	LOC440104	AK057179
A_23_P56127	C19orf61	NM_019108

A_24_P15132	ZNF100	NM_173531
A_23_P20399	PSMD9	NM_002813
A_23_P92107	SLC15A2	NM_021082
A_32_P54242	LCORL	NM_153686
A_23_P25091	ATP6V1C2	NM_001039362
A_24_P94326	RASA4	NM_006989
A_24_P66312	KRTAP12-1	NM_181686
A_23_P60339	C9orf64	NM_032307
A_33_P32567	KIAA1324	NM_020775
A_32_P74037	ARL13B	NM_144996
A_33_P33377	TGS1	NM_024831
A_23_P12610	CTH	NM_001902
A_23_P10518	TFDP3	NM_016521
A_23_P16381	LILRB4	NM_006847
A_23_P25229	CSDA	NM_003651
A_32_P30456	TERF2	NM_005652
A_23_P33832	ELK3	NM_005230
A_24_P19293	UBASH3B	NM_032873
A_23_P77296	CHRNB4	NM_000750
A_23_P11009	NIT2	NM_020202
A_24_P21955	NFE2L1	NM_003204
A_23_P11467	ARHGEF16	NM_014448
A_33_P32681	ZNF614	NM_025040
A_23_P40866	ZBTB20	NM_015642
A_24_P26799	TOR1AIP1	NM_015602
A_23_P20135	ATAD3A	NM_018188
A_24_P37441	PDK1	NM_002610
A_23_P38095	ZNF420	NM_144689
A_23_P20521	UTP14C	NM_021645
A_32_P18321	ZNF367	NM_153695
A_24_P29788	MTAP	NM_002451
A_32_P46154	ZNF708	NM_021269
A_23_P36625	PATZ1	NM_032051
A_23_P37481	ATM	NM_000051
A_23_P12135	BBX	NM_020235
A_33_P33374	CD248	NM_020404
A_23_P10542	HTRA3	NM_053044
A_23_P12047	TFAP2C	NM_003222
A_32_P78539	MYO16	NM_015011
A_32_P43688	C1orf69	NM_001010867
A_23_P39914	ZNF549	NM_153263
A_24_P74753	ATP6AP1	NM_001183
A_23_P21253	CHMP2B	NM_014043
A_33_P32526	CYP2W1	NM_017781
A_23_P23266	BLZF1	ENST00000367808
A_33_P32110	ZNF684	NM_152373
A_24_P11577	BIRC2	NM_001166
A_23_P12055	TASP1	NM_017714
A_23_P80966	SETD7	NM_030648

A_24_P75967	OBFC1	NM_024928
A_23_P36315	CGGBP1	NM_001008390
A_32_P40172	ZNF17	AB075827
A_23_P21192	WNT5A	NM_003392
A_23_P60471	UBAP2	NM_018449
A_24_P11687	TXNL4A	NM_006701
A_23_P98686	ATHL1	NM_025092
A_24_P71700	ZBTB47	NM_145166
A_23_P14083	AMIGO2	NM_181847
A_32_P41295	DNAJC3	NM_006260
A_23_P33089	NFKBIB	NM_001001716
A_23_P56933	RTN4	NM_020532
A_23_P48596	RNASE1	NM_198232
A_23_P21585	ZBTB10	ENST00000430430
A_24_P94193	EAF1	NM_033083
A_23_P13259	VGLL4	NM_014667
A_23_P32284	PPAPDC1B	NM_001102559
A_23_P17575	AHCY	NM_000687
A_23_P15894	ALDH4A1	NM_003748
A_32_P18560	HOXA7	NM_006896
A_33_P33575	ATHL1	NM_025092
A_23_P13073	TBC1D17	NM_024682
A_32_P38189	ZNF541	NM_001101419
A_23_P33232	ARHGEF19	NM_153213
A_32_P31048	ENST0000031	ENST00000319813
A_23_P14447	SPRY1	NM_199327
A_23_P13945	SH2B3	NM_005475
A_23_P12819	PFDN5	NM_002624
A_32_P72940	RPL35	NM_007209
A_32_P22869	PRDM10	NM_199437
A_24_P71181	GPBP1L1	NM_021639
A_23_P30897	ADAMTSL3	NM_207517
A_32_P12169	TACC2	NM_206862
A_33_P32256	ZNF526	NM_133444
A_24_P22005	MAPRE1	NM_012325
A_23_P33290	ZFC3H1	NM_144982
A_23_P36140	B3GAT3	NM_012200
A_23_P35736	STAG1	NM_005862
A_23_P69809	AGPAT9	NM_032717
A_32_P47546	CLDN16	NM_006580
A_24_P94274	ZFY	NM_003411
A_24_P34410	BRF1	NM_001519
A_23_P25761	PPP1R14C	NM_030949
A_23_P14708	ZNF280D	NM_017661
A_33_P32250	MTMR10	NM_017762
A_23_P50678	MATK	NM_139355
A_33_P33693	DNAJB5	NM_001135005
A_23_P50028	PACS1	NM_018026
A_23_P15254	SCPEP1	NM_021626

A_23_P23893	DSTYK	NM_015375
A_23_P40527	TBX1	NM_080646
A_24_P68759	LIX1L	ENST00000369308
A_23_P9894	PRMT3	NM_005788
A_24_P55496	OSR2	NM_053001
A_23_P67339	RCN3	NM_020650
A_24_P41703	PPARA	NM_005036
A_24_P12640	DNAH17	NM_173628
A_23_P54546	TPSAB1	NM_003294
A_23_P95718	TAF4B	NM_005640
A_32_P44686	NBPF15	NM_173638
A_23_P21858	BCL11A	NM_022893
A_33_P32598	DOCK9	NM_001130048
A_33_P34053	PDCL	NM_005388
A_23_P6980	ZNF717	NM_001128223
A_23_P13610	PDE4B	NM_001037341
A_23_P23646	USF1	NM_007122
A_24_P48308	TFDP1	NM_007111
A_24_P28718	TOLLIP	NM_019009
A_32_P15722	AHCTF1	NM_015446
A_33_P32923	KIF5B	NM_004521
A_23_P10566	CCDC59	NM_014167
A_32_P36104	ZDHHC1	NM_013304
A_33_P32970	FLG2	NM_001014342
A_24_P90209	ELK4	ENST00000357992
A_23_P84359	SSX5	NM_021015
A_32_P15434	SLCO4C1	NM_180991
A_33_P34156	TMIGD2	NM_144615
A_23_P14790	POU6F2	NM_007252
A_23_P16293	PAX9	NM_006194
A_23_P70968	HOXA7	NM_006896
A_23_P93727	SDK1	NM_152744
A_23_P11228	TMOD1	NM_003275
A_23_P15560	FMO3	NM_001002294
A_24_P40723	CRY1	NM_004075
A_23_P50646	LOC390940	ENST00000244321
A_23_P21245	SEC61A1	NM_013336
A_32_P43721	BLM	NM_000057
A_23_P20423	FOXJ2	NM_018416
A_23_P18294	RPL14	NM_001034996
A_23_P42117	FAM83H	NM_198488
A_33_P33511	TXNRD1	NM_003330
A_24_P22163	DICER1	NM_177438
A_23_P3402	ETFA	NM_000126
A_33_P33808	TLR9	NM_017442
A_23_P12134	XYLB	NM_005108
A_32_P35330	TRIP4	NM_016213
A_24_P54835	RPS29	NM_001032
A_32_P98313	NDUFA4	NM_002489

A_23_P39851[illegible]	PKMYT1	NM_004203
A_33_P33804[illegible]	GPR88	NM_022049
A_33_P32461[illegible]	RPL5	NM_000969
A_24_P22492[illegible]	MFNG	NM_002405
A_23_P25010[illegible]	CAND2	NM_012298
A_24_P53080	ETFA	NM_000126
A_23_P10513[illegible]	CAT	NM_001752
A_33_P33031[illegible]	MYNN	AK092022
A_32_P70216[illegible]	FBXL16	NM_153350
A_23_P34034[illegible]	LCE3E	NM_178435
A_33_P33266[illegible]	ZNF782	NM_001001662
A_23_P32933[illegible]	IQGAP3	NM_178229
A_23_P13966[illegible]	SLC2A3	NM_006931
A_33_P33439[illegible]	LMBR1	NM_022458
A_24_P94306[illegible]	KIAA0182	NM_014615
A_24_P18816[illegible]	SCAND3	NM_052923
A_23_P14971	HAS3	NM_005329
A_23_P10104[illegible]	KRTAP3-2	NM_031959
A_23_P25792[illegible]	ETS2	NM_005239
A_33_P33891[illegible]	TFAM	NM_003201
A_23_P37849[illegible]	ZNF776	AK095607
A_23_P26434	DBNDD1	NM_001042610
A_23_P15723[illegible]	IMPDH1	NM_000883
A_33_P34112[illegible]	PURA	NM_005859
A_23_P38075[illegible]	PRSS1	NM_002769
A_33_P34003[illegible]	SPATS2L	NM_001100422
A_33_P33030[illegible]	BTBD2	NM_017797
A_32_P36664[illegible]	MANBA	NM_005908
A_23_P13743[illegible]	RNF11	NM_014372
A_23_P2271	PTHLH	NM_198965
A_23_P4223	CALCOCO2	NM_005831
A_23_P15938[illegible]	SEC24D	NM_014822
A_23_P72859	PPFIA2	AB210009
A_23_P2793	ALOX5AP	NM_001629
A_23_P63009	LASS2	NM_181746
A_23_P55400	KRT36	NM_003771
A_33_P32521[illegible]	HMX3	NM_001105574
A_32_P87346[illegible]	BACH2	NM_021813
A_23_P20595[illegible]	ALDH1A3	NM_000693
A_24_P29096[illegible]	DNAJC16	NM_015291
A_24_P92868[illegible]	HIC2	ENST00000443632
A_23_P65877	SLC12A6	NM_001042496
A_23_P32445[illegible]	ZNF254	NM_203282
A_32_P97192	PMS2L2	BC010535
A_24_P24256	ENST0000031[illegible]	ENST00000317596
A_24_P41471[illegible]	BRPF3	NM_015695
A_32_P17366[illegible]	CRISP2	NM_003296
A_33_P32319[illegible]	POU3F3	NM_006236
A_23_P10652[illegible]	INO80	NM_017553

A_23_P31333(	ZNF483	NM_133464
A_32_P38003	EIF2AK2	ENST00000233057
A_24_P27367!	YAP1	NM_006106
A_23_P43492!	CEP120	NM_153223
A_23_P87973	TRIM13	NM_213590
A_23_P13995(	KPNA3	NM_002267
A_24_P47245!	ARF6	NM_001663
A_23_P31901:	ZNF383	NM_152604
A_23_P12858:	SAP18	NM_005870
A_23_P84836	NPEPPS	NM_006310
A_23_P89073	ZNF23	NM_145911
A_24_P91590!	TUSC3	AK026149
A_23_P14591(	AEBP1	NM_001129
A_24_P31218!	MEPCE	NM_019606
A_23_P62267	KDM5C	NM_004187
A_24_P93855	FOXP4	NM_001012426
A_32_P13282:	OPHN1	NM_002547
A_33_P34125:	PEX26	NM_001127649
A_24_P38960!	C10orf47	NM_153256
A_23_P14008!	RNASEH2B	NM_024570
A_24_P1054	NFKBIL2	NM_013432
A_32_P63182	PDIA3	NM_005313
A_32_P50727:	ZNF324	NM_014347
A_23_P96940	TNFRSF1B	NM_001066
A_23_P16454(	MBD1	NM_015846
A_23_P19517	ITPR3	NM_002224
A_23_P11507	ZZZ3	NM_015534
A_23_P97274	SCAMP3	NM_052837
A_23_P95736	ZNF429	NM_001001415
A_24_P40117	KIAA0556	NM_015202
A_23_P25845(	FOXC1	NM_001453
A_23_P40040(	ANKRD43	NM_175873
A_32_P45620!	RNF152	ENST00000312828
A_24_P19141:	NAB1	NM_005966
A_23_P66774	GGT6	NM_153338
A_23_P60472	UBAP2	NM_018449
A_33_P32572:	TAF10	NM_006284
A_23_P32376:	TRAF3IP3	NM_025228
A_32_P41461	WASF2	NM_006990
A_24_P15984!	ZNF146	NM_007145
A_23_P5240	ZNF43	NM_003423
A_23_P41639!	STC2	NM_003714
A_33_P33110:	CYB5A	NM_148923
A_32_P37893:	YEATS4	NM_006530
A_24_P13682	TLE3	NM_005078
A_24_P10001(	ZNF486	NM_052852
A_23_P33231(	ARRDC1	NM_152285
A_24_P11352:	DGCR8	NM_022720
A_24_P27154:	C22orf32	NM_033318

A_23_P66260	ZNF267	NM_003414
A_23_P21860[illegible]	EIF5B	NM_015904
A_23_P16157	KHSRP	NM_003685
A_23_P769	PSMB4	NM_002796
A_23_P550	ARNT	NM_001668
A_23_P68927	CBY1	NM_001002880
A_23_P21713	SPAG16	NM_024532
A_23_P25849	KCNH5	NM_139318
A_24_P78789[illegible]	XYLT1	NM_022166
A_24_P76740	CC2D1B	NM_032449
A_23_P66827	FAM106A	NR_026809
A_23_P41978[illegible]	ZNF781	NM_152605
A_23_P73660	TRAPPC2	NM_001011658
A_23_P16550[illegible]	TNP1	NM_003284
A_32_P36228[illegible]	TPRX1	NM_198479
A_23_P88381	NUMB	NM_001005743
A_23_P32148[illegible]	COX18	NM_173827
A_23_P19028	ARSI	NM_001012301
A_23_P30614[illegible]	PML	NM_002675
A_23_P25768[illegible]	TRAPPC9	NM_031466
A_23_P8412	GALNT11	NM_022087
A_23_P69810	AGPAT9	NM_032717
A_23_P13034[illegible]	KCTD1	NM_198991
A_33_P32165[illegible]	EPHB4	NM_004444
A_23_P17024[illegible]	OCRL	NM_000276
A_23_P20822[illegible]	ZNF480	NM_144684
A_23_P25770[illegible]	HSPB1	NM_001540
A_33_P38131[illegible]	SEMA3F	NM_004186
A_23_P14646[illegible]	VAV2	NM_003371
A_23_P6381	MN1	NM_002430
A_23_P15678[illegible]	STX11	NM_003764
A_23_P19408	HIST1H2BM	NM_003521
A_23_P82478	PUS7	NM_019042
A_32_P15767[illegible]	TRIM16L	NM_001037330
A_23_P40581[illegible]	PYGM	NM_005609
A_33_P33080[illegible]	EIF4E2	NM_004846
A_33_P32697[illegible]	ZNF3	NM_017715
A_23_P77731	CRYM	NM_001888
A_24_P10683[illegible]	WHSC1L1	NM_023034
A_23_P80739	PLCD1	NM_006225
A_23_P35820	CFL1	NM_005507
A_23_P85521	ZRANB2	NM_203350
A_24_P28409[illegible]	DACT1	NM_016651
A_23_P99536	MPP5	NM_022474
A_24_P75344	SMYD4	NM_052928
A_33_P32803[illegible]	LOC10013085[illegible]	AK097050
A_23_P31822[illegible]	SF1	NM_004630
A_24_P30377[illegible]	CTSB	NM_147780
A_32_P33561	LOC10000967[illegible]	NR_024407

A_24_P76620[illegible]	MAP3K1	NM_005921
A_23_P42292[illegible]	TAF3	NM_031923
A_23_P75398	NDUFS3	NM_004551
A_23_P10513	TFDP3	NM_016521
A_23_P42279	C4B	NM_001002029
A_24_P25435[illegible]	AIRE	NM_000658
A_23_P25779[illegible]	NDUFA2	NM_002488
A_24_P92775[illegible]	KRT77	NM_175078
A_32_P49423	NPM1	NM_001037738
A_32_P17974[illegible]	ATXN7L1	NM_020725
A_24_P24646[illegible]	ATF2	NM_001880
A_33_P33114[illegible]	GCH1	NM_001024071
A_23_P11136[illegible]	HSF2	NM_004506
A_32_P56754	PARP14	NM_017554
A_24_P62833	ADAMTSL4	AK023606
A_23_P11028[illegible]	GSTCD	NM_024751
A_33_P36474[illegible]	ZNF29P	X52357
A_23_P11741[illegible]	BRF1	NM_145685
A_23_P16274[illegible]	CRYL1	NM_015974
A_23_P11084[illegible]	CNOT8	NM_004779
A_32_P51640[illegible]	AACS	AK022740
A_23_P20573[illegible]	BCL11B	NM_138576
A_23_P91379	RIN2	NM_018993
A_23_P8664	DMTF1	NM_021145
A_23_P41910[illegible]	TCP11L2	NM_152772
A_23_P88095	TBC1D4	NM_014832
A_23_P15553[illegible]	POU1F1	NM_000306
A_32_P39841[illegible]	ZNF17	NM_006959
A_23_P4443	TBX4	NM_018488
A_23_P35916	ATM	NM_000051
A_23_P92300	ZNF501	NM_145044
A_24_P26303[illegible]	ALDH4A1	NM_003748
A_23_P42258[illegible]	MYC	NM_002467
A_24_P39007[illegible]	SFRS9	NM_003769
A_23_P25935[illegible]	SLC35E4	NM_001001479
A_24_P36116[illegible]	CHD8	NM_020920
A_23_P87279	TRPM5	NM_014555
A_33_P33025[illegible]	GPR153	NM_207370
A_23_P42829	SND1	NM_014390
A_24_P41795[illegible]	C14orf43	NM_194278
A_23_P30698[illegible]	SOX7	NM_031439
A_23_P36642[illegible]	L3MBTL4	BC039316
A_24_P32390[illegible]	PEAR1	NM_001080471
A_33_P32675[illegible]	SEMG1	NM_003007
A_23_P44684	ECT2	NM_018098
A_23_P77833	PGS1	NM_024419
A_24_P21088[illegible]	SMURF2	AK002019
A_24_P21040[illegible]	KLF5	NM_001730
A_23_P12812[illegible]	RAN	ENST00000448750

A_32_P22484(	AKTIP	NM_001012398
A_23_P83403	LIMCH1	NM_014988
A_23_P35349	SVIL	NM_021738
A_23_P16080(	NR0B2	NM_021969
A_33_P33788:	SLC9A3R1	NM_004252
A_33_P32517!	PDSS1	NM_014317
A_23_P89882	TRIM28	NM_005762
A_24_P40722	RASSF8	NM_001164746
A_23_P56356	PLB1	NM_153021
A_24_P12497:	NDNL2	NM_138704
A_23_P47806	MIP	NM_012064
A_23_P50288:	CBFA2T2	NM_005093
A_23_P41430	FLCN	NM_144606
A_24_P40934(	NPEPPS	NM_006310
A_23_P81973	HSD17B8	NM_014234
A_33_P33707(	SARM1	NM_015077
A_23_P55256	ZNF652	NM_014897
A_23_P35885	PRDM10	NM_199437
A_24_P19324	SOHLH2	NM_017826
A_23_P68031	STAT4	NM_003151
A_23_P54460	SPRED1	NM_152594
A_33_P32700(	NCOA1	NM_147223
A_32_P18493:	UBE2S	NM_014501
A_24_P96325	ZGPAT	NM_032527
A_23_P17124!	IGBP1	NM_001551
A_24_P92361:	PTHLH	ENST00000354417
A_23_P30501(	TADA3	NM_006354
A_23_P47116	RASSF7	NM_003475
A_32_P39050:	LOC10012828	NR_024447
A_24_P15385:	TRIM37	NM_001005207
A_33_P32261	CYFIP2	NM_001037332
A_23_P15218:	POLR3E	NM_018119
A_24_P8474	PTPRZ1	NM_002851
A_23_P11173	RAMP3	NM_005856
A_23_P14162	KRTAP1-1	NM_030967
A_24_P22299	ZRANB3	NM_032143
A_23_P10612	KIAA0586	NM_014749
A_23_P25909	ZSCAN16	NM_025231
A_23_P30977!	N4BP2	NM_018177
A_24_P12804:	ATRX	BT007188
A_23_P202	EPS15	NM_001981
A_32_P33605!	ZBTB47	NM_145166
A_24_P33860:	SKI	NM_003036
A_23_P16903(	TNFRSF10B	NM_003842
A_24_P23775	ZMYM2	AL136621
A_24_P6135	L3MBTL2	NM_031488
A_23_P16821:	VOPP1	NM_030796
A_23_P14314:	ID2	NM_002166
A_32_P4199	RNF152	NM_173557

A_23_P67437	KIR3DL1	NM_013289
A_32_P51221	CAB39	NM_016289
A_24_P12434	PDGFD	NM_025208
A_23_P91254	ZNF334	NM_199441
A_33_P34040	HIST3H2A	NM_033445
A_23_P14577	NDUFA4	NM_002489
A_23_P14567	ACTL6B	NM_016188
A_32_P95223	FDPSL2A	NR_003262
A_23_P92623	PPP3CA	NM_000944
A_33_P34122	POMZP3	NM_152992
A_32_P43551	NCCRP1	NM_001001414
A_23_P88434	FOXN3	NM_005197
A_23_P15736	WDR60	NM_018051
A_23_P75903	TOLLIP	NM_019009
A_24_P92359	ZNF605	BC020877
A_33_P33802	MTG1	NM_138384
A_23_P45875	TPR	NM_003292
A_23_P89601	KRT32	NM_002278
A_23_P33121	B4GALNT2	NM_153446
A_24_P27037	NUFIP1	NM_012345
A_23_P35222	MAPKBP1	NM_014994
A_24_P36313	TRIM36	NM_018700
A_23_P15786	KRT25	NM_181534
A_32_P34128	THSD1P1	NR_002816
A_23_P13410	EPB41L2	NM_001431
A_33_P32353	UBA5	NM_024818
A_23_P11030	CNOT6L	NM_144571
A_23_P13725	SLC6A15	NM_182767
A_23_P13227	MCM5	NM_006739
A_24_P20630	MICALL2	NM_182924
A_23_P50270	PAX3	NM_013942
A_24_P37291	HNF1A	NM_000545
A_33_P32255	TRMT2B	NM_024917
A_23_P20127	UBE4B	NM_006048
A_23_P15746	UBXN8	NM_005671
A_32_P43640	TBC1D19	NM_018317
A_23_P17003	CEND1	NM_016564
A_23_P16028	KLHL20	NM_014458
A_23_P50331	FAM32A	NM_014077
A_23_P10775	S1PR5	NM_030760
A_23_P30519	STAT4	NM_003151
A_32_P72447	UBE2S	NM_014501
A_23_P20444	ANAPC5	NM_016237
A_23_P34820	SPRR1A	NM_005987
A_33_P32789	REC8	NM_001048205
A_23_P91757	TEF	NM_003216
A_23_P25315	EP400	NM_015409
A_24_P50248	FAM110C	NM_001077710
A_24_P41770	MXD3	NM_001142935

A_23_P50007[illegible]	TGIF1	NM_170695
A_23_P36994[illegible]	FOXRED2	NM_024955
A_23_P84929	SLC38A5	NM_033518
A_23_P90004	SIN3B	NM_015260
A_23_P41716	GNB2L1	NM_006098
A_23_P24332	MUC15	NM_145650
A_23_P28869	PTPRA	NM_002836
A_23_P16612[illegible]	POLR3F	NM_006466
A_33_P32937[illegible]	KRTAP10-4	NM_198687
A_24_P15339[illegible]	SGSM3	NM_015705
A_33_P33346[illegible]	PLP1	NM_001128834
A_23_P16048[illegible]	DCAF6	NM_018442
A_24_P7594	APOL6	NM_030641
A_23_P5686	TBR1	U49250
A_32_P69120	TMEM67	NM_153704
A_24_P87413	ZNF771	NM_016643
A_23_P36234	TEAD1	NM_021961
A_23_P12738[illegible]	MYBPC3	NM_000256
A_23_P20949[illegible]	PAX3	NM_181458
A_23_P21035[illegible]	LIMS1	NM_004987
A_24_P13532[illegible]	NRP1	NM_001024629
A_23_P57059	STAU1	NM_017453
A_32_P48044[illegible]	ESX1	NM_153448
A_23_P24763	RPS13	NM_001017
A_23_P25176	TBX5	NM_080718
A_23_P14413[illegible]	C3orf58	NM_173552
A_23_P14998[illegible]	PDLIM1	NM_020992
A_23_P16455	GNA15	NM_002068
A_33_P34074[illegible]	ZSCAN10	NM_032805
A_23_P34743[illegible]	DVL1	NM_004421
A_23_P34588[illegible]	GFPT1	NM_002056
A_23_P38824[illegible]	C22orf39	NM_173793
A_23_P79086	NFKBIB	NM_002503
A_32_P70420	RNF34	BC046174
A_23_P15745[illegible]	POLR2K	NM_005034
A_24_P41935[illegible]	PDE4D	NM_006203
A_23_P46426	CYR61	NM_001554
A_23_P31651[illegible]	HOXB3	NM_002146
A_23_P20334[illegible]	ZFP91	NM_053023
A_23_P68165	SMYD1	NM_198274
A_23_P41392[illegible]	DMRTA1	NM_022160
A_23_P5813	SOCS5	NM_144949
A_23_P52793	PPP2R1B	NM_002716
A_23_P90023	ZNF557	NM_024341
A_32_P12572[illegible]	RNF13	NM_007282
A_23_P20800[illegible]	SEC11C	NM_033280
A_23_P16541[illegible]	BZW1	NM_014670
A_24_P13032	UBE2D1	NM_003338
A_33_P33564[illegible]	SOS1	NM_005633

A_23_P12237	ZFAND3	NM_021943
A_24_P24689	NEU4	NM_080741
A_32_P87980	TRIM16L	NM_001037330
A_23_P20585	ARIH1	NM_005744
A_24_P34809	ME2	NM_002396
A_33_P32937	KRTAP10-9	NM_198690
A_23_P21644	NFIB	NM_005596
A_32_P19833	SFRS12IP1	NM_173829
A_33_P33476	MED18	NM_017638
A_23_P31657	RPL8	NM_000973
A_23_P24308	ALDH3B2	NM_000695
A_23_P16107	CD2	NM_001767
A_24_P39960	NOTCH3	NM_000435
A_23_P11060	MAML1	NM_014757
A_24_P64358	ASB8	NM_024095
A_24_P93512	ZNF23	AF024709
A_23_P2990	CEBPE	NM_001805
A_24_P25166	KCNMB3	NM_171828
A_32_P89837	TRAF3	NM_145725
A_23_P15074	C2CD3	AL080220
A_24_P38240	ZNF791	NM_153358
A_32_P54057	NFE2L3	BC008645
A_24_P27052	GTF2A2	NM_004492
A_23_P15911	CRKRS	NM_016507
A_32_P54105	MYPOP	NM_001012643
A_24_P38941	PNMA2	NM_007257
A_23_P21623	NEFM	NM_005382
A_32_P23512	RPL37	NM_000997
A_23_P14123	POLR2A	NM_000937
A_23_P12291	BRI3	NM_015379
A_32_P42775	ZNF718	BC020836
A_24_P31771	WHSC1L1	NM_017778
A_24_P15062	ZNF490	NM_020714
A_23_P65129	SPRYD3	NM_032840
A_24_P28929	ARHGEF25	NM_182947
A_33_P37487	FUT10	NM_032664
A_23_P50095	B3GNT2	NM_006577
A_23_P50146	CDK11B	NM_033489
A_23_P14185	ZNF544	NM_014480
A_23_P30189	PRDM2	NM_015866
A_33_P32647	NOTO	NM_001134462
A_33_P32556	HNRNPH2	NM_019597
A_23_P13242	MYLK	NM_053031
A_23_P25396	NR1H4	NM_005123
A_33_P33806	TBPL1	NM_004865
A_32_P78295	ATOH7	NM_145178
A_33_P32567	LOC404266	NR_033201
A_23_P91795	GRAMD4	NM_015124
A_24_P28634	ZBTB2	NM_020861

A_33_P32948.	OTOP1	NM_177998
A_23_P21135!	DGCR8	NM_022720
A_32_P52609	LPIN1	NM_145693
A_32_P40665.	MAP2K1	NM_002755
A_23_P32507!	RNGTT	NM_003800
A_24_P89843	CYHR1	NM_032687
A_23_P15394:	C2orf34	NM_024766
A_24_P78540	NR2C1	NM_003297
A_23_P20617.	SNAP23	NM_003825
A_32_P91173.	POU6F2	NM_007252
A_23_P51291	PARS2	NM_152268
A_23_P15484!	OLIG1	NM_138983
A_32_P53617(	DOCK10	NM_014689
A_23_P26107	RFX7	NM_022841
A_33_P33891!	STK10	NM_005990
A_23_P50188.	DHPS	NM_013407
A_32_P47554	HINT1	NM_005340
A_33_P36641(	LOC388946	NM_001145051
A_24_P41716.	TBL1X	NM_005647
A_23_P3532	LITAF	NM_004862
A_24_P24700(	DENND2C	NM_198459
A_32_P21058!	ASXL3	NM_030632
A_23_P8754	AASS	NM_005763
A_23_P76529	ITGB7	NM_000889
A_23_P41315(	KLF13	NM_015995
A_33_P32917!	CAMKV	NM_024046
A_23_P12493.	IKZF1	AF116605
A_32_P35375	DEAF1	NM_021008
A_24_P27276:	DENND1A	NM_020946
A_23_P20446!	PLA2G1B	NM_000928
A_33_P33289.	BMP2K	NM_017593
A_24_P11690!	MALT1	NM_006785
A_33_P33338(	NOXO1	NM_172168
A_32_P46031.	LRBA	NM_006726
A_23_P20464(	NANOG	NM_024865
A_32_P19054(	FAM69B	NM_152421
A_32_P10171	PCBD1	BM550965
A_23_P24397	RCOR2	NM_173587
A_23_P21161!	SCUBE1	NM_173050
A_32_P22063.	LRRC8C	NM_032270
A_33_P36724!	USP28	NM_020886
A_32_P36980!	ZNF169	NM_194320
A_23_P15218	ZNF768	NM_024671
A_24_P49731	NFYB	NM_006166
A_23_P3627	SPG7	NM_003119
A_24_P20032	RFX5	NM_000449
A_32_P18693!	SUCLG2	NM_003848
A_23_P50839	MBD3	NM_003926
A_23_P15133.	DLEU1	NR_002605

A_23_P22672	ALG13	NM_001099922
A_24_P26878	MYNN	NM_018657
A_23_P75453	MEN1	NM_130803
A_33_P33200	SCML2	NM_006089
A_24_P66528	SRP9	NM_003133
A_32_P74473	FLJ38576	XR_112683
A_33_P32877	MYST3	NM_001099412
A_24_P20587	SEC22C	NM_004206
A_24_P38276	FZD1	NM_003505
A_24_P85099	HMGA2	NM_003483
A_24_P39688	NDUFA10	ENST00000252711
A_23_P33598	HOXC12	NM_173860
A_23_P32706	KIAA0232	NM_014743
A_23_P71591	NOL8	NM_017948
A_23_P80694	ACTR8	NM_022899
A_23_P1513	PITPNM1	X98654
A_24_P31643	PLA2G4C	NM_003706
A_32_P61029	CSDA	NM_003651
A_24_P15380	RHOJ	NM_020663
A_23_P31034	MYLIP	NM_013262
A_33_P33664	HIBCH	NM_198047
A_23_P34946	C1orf107	NM_014388
A_33_P33669	GPR125	NM_145290
A_24_P12723	BCR	NM_004327
A_33_P33020	UGT1A8	NM_019076
A_23_P14718	CANX	NM_001746
A_33_P34135	NDUFB3	NM_002491
A_23_P92230	TSC22D2	NM_014779
A_23_P62709	SPRR3	NM_005416
A_23_P25237	RBBP8	NM_002894
A_32_P22929	PHF6	NM_032458
A_24_P34788	ALOXE3	NM_021628
A_32_P72861	TLE1	NM_005077
A_23_P13687	ZBTB39	NM_014830
A_23_P11136	SYTL4	NM_080737
A_33_P33587	GRINL1A	NR_027390
A_23_P41674	GRPEL2	NM_152407
A_23_P35121	SKIL	NM_005414
A_24_P27460	SRA1	NM_001035235
A_23_P38837	ATRNL1	NM_207303
A_23_P29257	H1F0	NM_005318
A_24_P33706	TSC22D2	NM_014779
A_23_P25055	AGGF1	NM_018046
A_23_P25503	FNDC3A	NM_001079673
A_23_P39207	STRN3	NM_014574
A_23_P27346	SMAD4	NM_005359
A_32_P12224	ASCL5	XM_940966
A_32_P15980	TPRG1L	NM_182752
A_23_P3413	OTUD7A	NM_130901

A_23_P45059	DOCK1	NM_001380
A_24_P297539	UBE2C	NM_181803
A_24_P360601	NOL8	NM_017948
A_32_P70771	ATP6V0E1	NM_003945
A_24_P20524	TMEM192	NM_001100389
A_23_P336479	G3BP1	NM_005754
A_33_P385151	LIN54	NM_194282
A_23_P89139	ZNF83	NM_018300
A_23_P103771	LRRC8C	NM_032270
A_32_P193080	FMNL2	NM_052905
A_33_P325697	ACTL7B	NM_006686
A_23_P33643	IL7R	ENST00000303115
A_23_P300728	LMBR1	NM_022458
A_23_P257895	MAPK1	NM_138957
A_23_P132923	DGKQ	NM_001347
A_23_P22761	SHOX	NM_006883
A_23_P168218	RPL15	NM_002948
A_24_P735072	IKZF3	ENST00000488188
A_23_P32233	KLF4	NM_004235
A_23_P251252	GTF2H4	NM_001517
A_24_P64344	BLNK	NM_013314
A_24_P925186	SOX5	NM_152989
A_24_P243044	GSK3B	ENST00000264235
A_24_P58283	SP3	NM_003111
A_23_P353085	C19orf39	NM_175871
A_23_P77661	ZNF720	NM_001130913
A_23_P200461	ITPKB	NM_002221
A_23_P77669	ZNF821	NM_017530
A_23_P84620	DTL	NM_016448
A_23_P108785	ACTR3	NM_005721
A_23_P215669	POLR2J2	NM_032959
A_23_P415558	ZNF212	NM_012256
A_23_P138811	CHORDC1	NM_012124
A_23_P142671	ZNF513	NM_144631
A_23_P93389	NUDT3	NM_006703
A_23_P4850	ZNF625	NM_145233
A_23_P210300	ASXL2	NM_018263
A_23_P96568	FLNA	NM_001456
A_23_P116829	UBE2N	NM_003348
A_23_P62920	KIFAP3	NM_014970
A_23_P101615	ZNF565	NM_001042474
A_23_P340149	ZNF711	NM_021998
A_33_P322610	LCE4A	NM_178356
A_23_P67785	SPAG16	NM_001025436
A_33_P324838	HCG2P7	NR_001318
A_23_P31996	SLC46A2	NM_033051
A_32_P39963	EXOSC6	CR604283
A_33_P323099	SCUBE1	NM_173050
A_23_P211785	ZNF35	NM_003420

A_23_P73677	RHOXF2B	NM_001099685
A_23_P250948	BRPF1	NM_001003694
A_24_P4426	INPP5F	NM_014937
A_33_P337662	MLLT6	NM_005937
A_32_P23838	CYP4V2	NM_207352
A_23_P82351	BBS9	NM_198428
A_23_P201789	KDM5B	NM_006618
A_23_P392501	TNRC6C	NM_001142640
A_23_P97795	ACBD5	NM_145698
A_23_P202978	CASP1	NM_033292
A_23_P47867	PPFIBP1	NM_003622
A_32_P212195	PAIP1	NM_006451
A_23_P251881	NCR3	NM_147130
A_32_P21579	TRDMT1	NM_004412
A_23_P141779	CXXC1	NM_014593
A_23_P141738	SS18	NM_001007559
A_23_P202053	ITIH2	NM_002216
A_23_P98382	TIMM8B	NM_012459
A_23_P378514	ATOH7	NM_145178
A_24_P280706	SUGT1	NM_006704
A_32_P148710	CFL1	NM_005507
A_23_P216845	GFI1B	NM_004188
A_23_P120572	RIN2	NM_018993
A_23_P13711	MLL2	NM_003482
A_33_P328780	LBX2	NM_001009812
A_33_P322947	KRTAP5-10	NM_001012710
A_23_P149390	CGN	NM_020770
A_23_P146186	RRS1	NM_015169
A_23_P114607	LCE1C	NM_178351
A_23_P209879	ATF2	ENST00000264110
A_33_P339831	MMP24	NM_006690
A_23_P368101	SAP30L	NM_024632
A_23_P45074	TUFM	S75463
A_23_P29680	CAMKV	NM_024046
A_23_P140646	CYFIP1	NM_014608
A_24_P109351	KIAA0564	NM_001009814
A_23_P143520	C21orf122	NR_027292
A_23_P97394	BCAR3	NM_003567
A_24_P26496	NOTCH4	NM_004557
A_32_P152437	AKAP12	NM_005100
A_32_P81806	ZNF738	NR_027130
A_24_P391625	KRT79	NM_175834
A_24_P393711	ABL1	NM_005157
A_23_P125042	ZNF222	NM_013360
A_23_P156739	C6orf125	NM_032340
A_33_P337561	SGK3	NM_013257
A_23_P309207	ZNF577	NM_032679
A_24_P293358	C2CD2	NM_015500
A_32_P223859	SMPDL3A	NM_006714

A_23_P21596	STK3	NM_006281
A_23_P20648	SURF1	NM_003172
A_32_P23269	EXPH5	NM_015065
A_24_P10638	SENP6	AK001406
A_24_P39895	UNC45A	NM_001039675
A_24_P12771	MAFA	NM_201589
A_32_P52378	DTNA	NM_001392
A_33_P34158	NRBF2	NM_030759
A_23_P25189	C7orf27	NM_152743
A_23_P10377	LRRC8C	NM_032270
A_23_P13013	BCL6B	NM_181844
A_24_P33710	OXTR	NM_000916
A_32_P13137	REEP5	NM_005669
A_24_P13129	POU3F1	NM_002699
A_23_P40293	PPFIA2	NM_003625
A_23_P48130	IFT81	CR608549
A_23_P20391	GTF2H3	ENST00000228955
A_23_P56304	ZNF430	NM_025189
A_33_P33424	RASA4	NM_006989
A_23_P56971	C20orf11	NM_017896
A_23_P53856	N4BP2L2	NM_014887
A_23_P15202	CSK	NM_004383
A_23_P30307	MED7	NM_004270
A_33_P34113	KRTAP3-3	NM_033185
A_23_P15893	GPR125	NM_145290
A_23_P42776	LCE2C	NM_178429
A_32_P61857	KIAA1468	NM_020854
A_33_P33060	MYCL1	NM_001033081
A_33_P33229	C10orf108	NR_027151
A_23_P88731	RAD51	NM_002875
A_32_P37442	SOX30	AK302162
A_23_P71741	KIF27	NM_017576
A_23_P35796	PCMTD1	NM_052937
A_32_P16353	ZNF322B	NM_199005
A_23_P25710	NCBP1	NM_002486
A_23_P20967	PLEK	NM_002664
A_23_P20489	NUPL1	NM_014089
A_24_P69691	ZNF25	NM_145011
A_32_P14846	LCE5A	NM_178438
A_24_P34800	RASD1	NM_016084
A_33_P33389	DAB2	NM_001343
A_23_P32696	C3orf59	NM_178496
A_24_P75245	TNRC18	NM_001080495
A_33_P33705	GALC	NM_001037525
A_23_P16126	CSGALNACT2	NM_018590
A_23_P66289	ZNF668	NM_024706
A_32_P22377	IL6ST	NM_002184
A_23_P21781	GABRD	NM_000815
A_32_P21025	RPL22	NM_000983

A_32_P10882	ZBTB41	NM_194314
A_23_P21176	COL8A1	ENST00000261037
A_33_P37144	LRP5L	NM_001135772
A_32_P16160	C19orf40	NM_152266
A_23_P65870	FBXO22	NM_012170
A_24_P33757	FAM8A1	NM_016255
A_33_P33917	SIRT7	NM_016538
A_33_P33525	ACOT8	NM_005469
A_23_P25941	CDV3	NM_017548
A_23_P63584	AHCTF1	NM_015446
A_33_P32928	TMEM191A	NR_026815
A_23_P37794	CBFB	NM_001755
A_23_P40528	SEMA3D	NM_152754
A_32_P20401	RPL29	NM_000992
A_33_P32348	PAX8	NM_003466
A_23_P68198	SH3YL1	NM_015677
A_23_P16142	PLXDC2	NM_032812
A_24_P16839	ZNF177	NM_003451
A_23_P14967	PIAS3	NM_006099
A_23_P20313	UBE4A	NM_004788
A_24_P65658	ZNF432	AB018341
A_24_P37976	MOBKL1B	NM_018221
A_23_P25581	TMEM66	NM_016127
A_24_P37004	FAM59A	NM_022751
A_23_P14351	C21orf122	NR_027292
A_24_P20187	UTP14C	NM_021645
A_23_P17624	TIAM1	NM_003253
A_23_P16211	NUMA1	NM_006185
A_23_P15912	SLC16A5	NM_004695
A_24_P35022	SLC22A23	NM_021945
A_23_P12094	XRCC6	NM_001469
A_23_P55682	ZSCAN18	NM_023926
A_23_P76598	EFHA1	NM_152726
A_23_P20262	GAL	NM_015973
A_23_P25658	PRDM13	NM_021620
A_23_P24215	TBC1D12	NM_015188
A_23_P25110	SGCB	NM_000232
A_23_P87556	CPNE8	NM_153634
A_33_P34188	FLRT3	NM_198391
A_23_P33813	CSPG4P5	AB067507
A_23_P14830	RBM3	NM_006743
A_23_P15069	PSMA8	NM_144662
A_23_P75380	AIP	NM_003977
A_32_P1919	ZNF321	NM_203307
A_33_P33857	ZNF763	NM_001012753
A_32_P2442	CYP20A1	NM_177538
A_23_P15135	WASF3	NM_006646
A_33_P32212	DFNA5	NM_004403
A_33_P38867	PPA2	NM_176869

A_23_P78372	THOC1	NM_005131
A_23_P15319	TGIF1	NM_170695
A_24_P36665	SH3D19	NM_001009555
A_23_P25588	GSN	NM_198252
A_24_P94290	BMPER	ENST00000297161
A_24_P30593	TMCC3	NM_020698
A_23_P33499	SUDS3	NM_022491
A_23_P25407	STBD1	NM_003943
A_32_P79338	XYLT1	NM_022166
A_32_P84772	ZNF765	NM_001040185
A_23_P12129	UPK1B	NM_006952
A_23_P43779	COPS4	NM_016129
A_23_P28324	MEIS1	NM_002398
A_24_P20002	IL1R1	NM_000877
A_23_P50061	TNFRSF8	NM_001243
A_23_P46440	PVRL4	NM_030916
A_23_P32730	PAK2	NM_002577
A_23_P16890	ZFPM2	NM_012082
A_24_P93578	ZNF121	NM_001008727
A_32_P18318	PCOTH	NM_001014442
A_23_P25178	NAA16	NM_024561
A_23_P15025	RBM14	NM_006328
A_32_P35475	CNOT6L	NM_144571
A_24_P39756	MAPK14	NM_139013
A_23_P58521	ERCC8	NM_000082
A_32_P78813	LOC728690	AK094642
A_32_P33105	C20orf151	NM_080833
A_23_P16172	HSPB2	NM_001541
A_32_P34408	RPSAP52	NR_026825
A_23_P12356	RPS6	NM_001010
A_24_P17546	CAMSAP1	NM_015447
A_23_P79471	HK2	NM_000189
A_24_P11856	POM121L1P	NR_024591
A_24_P70675	PLA2G12A	NM_030821
A_23_P12336	PRMT6	NM_018137
A_23_P21807	SLC38A2	NM_018976
A_24_P97849	DBN1	NM_080881
A_23_P32025	MYO19	NM_025109
A_23_P92954	HSD17B4	NM_000414
A_33_P34120	ZNF582	NM_144690
A_32_P82222	CABLES2	NM_031215
A_33_P33282	PLAGL2	NM_002657
A_33_P33645	KRTAP6-1	NM_181602
A_23_P59375	ID4	NM_001546
A_23_P10994	ACVR2B	NM_001106
A_32_P10694	ZNF429	NM_001001415
A_23_P51181	MEGF6	NM_001409
A_23_P35895	CAMKK2	NM_006549
A_23_P20414	KRT85	NM_002283

A_33_P36127	USP48	NM_001032730
A_33_P32707	HTRA3	NM_053044
A_23_P37347	SNW1	NM_012245
A_32_P24223	SUZ12	NM_015355
A_23_P20931	CCNT2	NM_058241
A_23_P27664	SPIB	NM_003121
A_23_P13120	HES6	NM_018645
A_23_P63999	CUGBP1	AB210019
A_24_P41433	AFF1	NM_005935
A_24_P16359	C7orf60	NM_152556
A_24_P68631	HIST2H2AB	NM_175065
A_23_P88831	SLC7A6	NM_001076785
A_24_P21170	XPNPEP2	NM_003399
A_23_P32565	TRIM42	NM_152616
A_24_P28435	TMEM107	NM_032354
A_23_P43124	KBTBD8	NM_032505
A_32_P99174	CHST11	NM_018413
A_24_P34199	FLG	NM_002016
A_23_P20875	ZNF566	NM_001145345
A_23_P17097	ZNF692	NM_017865
A_24_P12905	FOXI1	NM_012188
A_23_P83558	KCNH6	NM_173092
A_32_P21898	YBX1	NM_004559
A_23_P28948	REM1	NM_014012
A_23_P33384	CIITA	NM_000246
A_23_P16333	CA12	ENST00000178638
A_23_P16148	TIAL1	NM_001033925
A_23_P20334	ZFP91	NM_053023
A_23_P10099	KRT38	NM_006771
A_23_P12980	RAB40B	NM_006822
A_23_P36275	PRDM5	NM_018699
A_23_P41697	FEM1C	NM_020177
A_23_P11111	VARS2	NM_020442
A_32_P37547	PIK3C3	ENST00000262039
A_33_P33068	ZNF846	NM_001077624
A_24_P26138	TAF1D	NM_024116
A_23_P50175	SIGLEC7	NM_014385
A_24_P23470	APBB2	NM_004307
A_23_P39131	GLTSCR2	NM_015710
A_24_P12432	IKZF5	ENST00000368886
A_32_P85275	SH3TC2	NM_024577
A_23_P54781	RBBP6	BC051317
A_24_P10130	ZNF568	NM_198539
A_32_P19261	TAF1	NM_004606
A_23_P93994	UBN2	NM_173569
A_23_P14501	BRD8	NM_006696
A_33_P34044	MAP3K6	NM_004672
A_24_P37615	YEATS2	AK090720
A_33_P33332	HOXD9	NM_014213

A_33_P32878	DDX21	NM_004728
A_33_P34100	LTA4H	NM_000895
A_23_P42647	ZNF45	NM_003425
A_23_P64247	TTC17	BC033000
A_24_P20371	FUBP3	NM_003934
A_23_P36220	PIP4K2B	NM_003559
A_23_P8196	ME1	NM_002395
A_23_P20315	TMPRSS13	CR613669
A_23_P25216	DAPK1	NM_004938
A_24_P91535	ZNF445	NM_181489
A_24_P33077	HOXB2	NM_002145
A_24_P96709	CDK11A	CR606630
A_32_P19887	METT5D1	NM_152636
A_24_P38677	PPP1CC	NM_002710
A_23_P41676	NOL4	NM_003787
A_24_P14826	TGFB2	NM_001135599
A_23_P87150	LPXN	NM_004811
A_32_P35551	TMPRSS4	NM_019894
A_23_P11830	DNAJA3	NM_005147
A_23_P2705	LPAR6	NM_005767
A_32_P30569	ZNF652	NM_001145365
A_23_P30631	MCM9	NM_017696
A_23_P44734	USP38	NM_032557
A_24_P80145	EHMT2	NM_006709
A_24_P18627	IGFBPL1	NM_001007563
A_23_P10539	CCT2	NM_006431
A_23_P75494	NPAS4	NM_178864
A_24_P32463	SBF2	AL832942
A_24_P31676	INSM1	NM_002196
A_32_P80157	C12orf54	NM_152319
A_32_P17967	TOB2	NM_016272
A_23_P21007	ZNF514	NM_032788
A_23_P16160	POLA2	NM_002689
A_23_P70818	SMO	NM_005631
A_33_P32815	SLC27A3	ENST00000271857
A_32_P14610	PDLIM5	NM_006457
A_23_P81408	MAT2B	NM_182796
A_32_P30196	ATN1	NM_001007026
A_32_P49456	HIVEP3	NM_024503
A_23_P13796	NHLH2	NM_005599
A_32_P22251	UBXN7	ENST00000296328
A_33_P33963	IL1R1	NM_000877
A_23_P27094	PFN1	NM_005022
A_24_P39386	PHTF1	NM_006608
A_33_P33737	DRD4	NM_000797
A_24_P10715	ZNF333	AF372702
A_23_P77572	EIF3C	NM_001037808
A_32_P23046	ZNF714	NM_182515
A_23_P40867	BTAF1	NM_003972

A_23_P11220	KDM4C	NM_015061
A_23_P18751	TMPRSS11E	NM_014058
A_24_P38803	HIAT1	NM_033055
A_33_P32814	TRAF3IP3	NM_025228
A_23_P15697	HUS1	NM_004507
A_23_P32992	GPR109A	NM_177551
A_23_P12990	TRIM16L	NM_001037330
A_23_P42319	RXRA	NM_002957
A_32_P20420	SIX4	NM_017420
A_23_P84063	NTM	NM_016522
A_24_P65858	SASH1	NM_015278
A_23_P13089	NCLN	NM_020170
A_33_P32600	PIAS4	ENST00000262971
A_24_P32236	VPS4B	NM_004869
A_33_P33221	DAZAP2	NM_001136269
A_23_P10220	MSH6	NM_000179
A_23_P12052	ProSAPiP1	NM_014731
A_23_P36920	CLCN5	NM_000084
A_23_P40448	S1PR1	NM_001400
A_32_P77558	ZNF670	AK027325
A_23_P52714	FGF19	NM_005117
A_24_P62538	CSDA	NM_003651
A_24_P41925	RASAL2	NM_170692
A_23_P31335	ZNF12	NM_016265
A_33_P33977	SYT2	NM_001136504
A_23_P13245	CCNL1	NM_020307
A_23_P78831	ZNF787	NM_001002836
A_23_P49219	TCF25	NM_014972
A_23_P21280	FGF5	NM_004464
A_23_P5464	RQCD1	ENST00000273064
A_33_P32934	GATA4	NM_002052
A_23_P19778	SLC13A4	NM_012450
A_23_P14422	SEC62	NM_003262
A_24_P43391	TMEM165	NM_018475
A_23_P54929	LYRM1	NM_020424
A_24_P53985	ZMYM1	NM_024772
A_23_P10320	PNRC2	NM_017761
A_24_P10289	WDTC1	NM_015023
A_23_P39662	SYNRG	NM_007247
A_33_P32875	BCAP29	NM_018844
A_33_P33727	NBPF15	NM_001170755
A_32_P50448	LY6G6C	NM_025261
A_24_P20501	ZNF250	NM_021061
A_24_P41526	DDX21	ENST00000354185
A_33_P34039	SAMD10	NM_080621
A_23_P11951	ZNF284	NM_001037813
A_24_P37589	ACPP	NM_001099
A_23_P13362	CDC23	NM_004661
A_23_P15829	TOP3A	NM_004618

A_32_P35229	KIAA2018	NM_001009899
A_32_P32552	ZNF470	NM_001001668
A_23_P14062	NUP107	NM_020401
A_23_P25475	CD164	NM_006016
A_23_P25619	SOHLH2	NM_017826
A_24_P15349	MECOM	NM_001164000
A_23_P56256	POLR2I	NM_006233
A_23_P35965	ZNF664	NM_152437
A_23_P13336	TCERG1	NM_006706
A_32_P15143	CRAMP1L	NM_020825
A_33_P32933	GFRA1	NM_005264
A_32_P32644	MED24	NM_014815
A_32_P14672	SS18	NM_005637
A_23_P16080	COG2	NM_007357
A_23_P30429	NDST1	NM_001543
A_23_P16408	MYH10	NM_005964
A_23_P14103	CHST4	NM_005769
A_23_P15129	IFNG	NM_000619
A_23_P21501	CMAH	NR_002174
A_23_P13554	DPYD	NM_000110
A_23_P90436	COMP	NM_000095
A_23_P68068	WDR54	NM_032118
A_23_P43539	PTGER4	NM_000958
A_24_P19394	PIGN	NM_176787
A_23_P15138	RAP2A	NM_021033
A_23_P14533	CCHCR1	NM_019052
A_23_P34014	ZNF711	NM_021998
A_23_P58180	CYP4V2	NM_207352
A_32_P57797	RHOT1	NM_018307
A_23_P11394	ADK	NM_001123
A_23_P40645	C11orf36	NR_027138
A_32_P11372	KGFLP1	NR_003674
A_32_P16725	ANO4	NM_178826
A_23_P16705	EVC	ENST00000382674
A_23_P11599	SPI1	NM_003120
A_23_P11653	SWAP70	NM_015055
A_23_P37855	PARP15	NM_152615
A_23_P93690	MCM7	NM_182776
A_23_P18887	MCCC2	NM_022132
A_23_P64051	CDC42BPG	ENST00000342711
A_24_P34212	KLHDC10	NM_014997
A_23_P50344	TUBB4	NM_006087
A_32_P43746	PRUNE2	NM_015225
A_24_P24925	LMOD2	NM_207163
A_23_P58937	TRAM2	NM_012288
A_33_P32167	DNAJC6	NM_014787
A_23_P16905	MRPS28	NM_014018
A_23_P13345	IRX1	NM_024337
A_24_P22338	HIST1H2AB	NM_003513

A_24_P93588!	ACOX1	NM_004035
A_23_P11953!	EFNA2	NM_001405
A_23_P10540!	MAP3K12	NM_006301
A_24_P12829:	ZNF138	NM_006524
A_23_P76291	PRR4	NM_007244
A_33_P32597:	AMACR	NM_014324
A_33_P32297(	WFIKKN1	NM_053284
A_33_P32276!	SLC9A5	NM_004594
A_23_P11544	TNFSF18	NM_005092
A_24_P21110(	TNFRSF11A	ENST00000269485
A_23_P31873	RAB11FIP1	NM_001002233
A_32_P13719	XPOT	NM_007235
A_23_P71598	MPDZ	NM_003829
A_23_P29384	ZNF502	NM_033210
A_23_P14020:	PCK2	NM_004563
A_24_P21752(	EREG	NM_001432
A_23_P60640	NPLOC4	NM_017921
A_23_P38569!	WNT3A	NM_033131
A_24_P21985	FOXJ2	NM_018416
A_32_P21385	ZC3H12C	NM_033390
A_23_P48628	ZBTB25	NM_006977
A_24_P38253:	NNAT	NM_005386
A_32_P49687:	PPFIA2	NM_003625
A_32_P3742	RFX3	ENST00000382004
A_32_P11358	ZNF292	NM_015021
A_23_P42802	PDIA4	NM_004911
A_23_P21817(	UNC45A	NM_001039675
A_33_P32648	SAMD9L	NM_152703
A_23_P41363:	ZNF329	NM_024620
A_23_P37554:	LCORL	NM_001166139
A_32_P78569:	ZNF517	NM_213605
A_24_P31075(	FBXO28	NM_015176
A_23_P14330:	VPS16	NM_022575
A_23_P43095	ZFHX4	NM_024721
A_23_P20956	CYBRD1	NM_024843
A_23_P15328:	FOSB	NM_006732
A_33_P33588!	SSC5D	NM_001144950
A_23_P16374:	ZNF213	NM_004220
A_24_P85026	ZCCHC7	NM_032226
A_33_P32377:	NR1H3	NM_005693
A_23_P21016	HOXD8	NM_019558
A_23_P20458:	TXNRD1	NM_003330
A_24_P15688(	DDX59	NM_001031725
A_24_P18146	PSD3	NM_015310
A_23_P16107	TOR1AIP1	NM_015602
A_33_P33914:	CRYZ	NM_001130042
A_32_P71514!	NLRC3	NM_178844
A_32_P10590	C19orf39	NM_175871
A_23_P74697	ATAD3B	NM_031921

A_23_P13211	SIK1	NM_173354
A_23_P25157	KLHL24	NM_017644
A_24_P13756	SCLT1	NM_144643
A_23_P47426	ACAD8	NM_014384
A_32_P41518	MDC1	NM_014641
A_23_P16011	S1PR1	NM_001400
A_23_P717	TMEM206	NM_018252
A_24_P18641	ZFAND3	NM_021943
A_24_P10572	CREB1	NM_134442
A_23_P36133	ADARB1	NM_015833
A_33_P33033	NCAPD2	NM_014865
A_23_P40037	C2orf67	NM_152519
A_24_P17455	RHOA	NM_001664
A_23_P13291	RBM47	NM_019027
A_23_P8408	ZNF862	NM_001099220
A_32_P16897	KRT16	NM_005557
A_23_P20817	ZNF613	AK023652
A_23_P38584	KRT27	NM_181537
A_23_P74467	KIAA0907	NM_014949
A_23_P39791	CBLC	NM_012116
A_23_P13399	PPIL1	NM_016059
A_23_P25297	SH3RF1	NM_020870
A_23_P12735	NDUFV1	NM_007103
A_24_P13486	ITIH5L	NM_198510
A_23_P18413	C3orf1	NM_016589
A_23_P39724	CLCA2	NM_006536
A_23_P15712	SKAP2	NM_003930
A_23_P10808	CREB3L3	NM_032607
A_23_P71492	NEFL	NM_006158
A_23_P86195	SLC44A3	NM_152369
A_23_P16008	ZNF600	NM_198457
A_23_P10666	CMTM1	NM_052999
A_24_P38002	EIF5A2	NM_020390
A_33_P34108	HIST1H4D	NM_003539
A_24_P9090	HNRPDL	NM_031372
A_23_P56298	ZNF430	NM_025189
A_23_P11941	C19orf2	NM_003796
A_24_P29423	GLS	ENST00000320717
A_23_P15573	NR3C2	NM_000901
A_23_P11522	HAX1	NM_006118
A_23_P21762	TAF7L	NM_024885
A_23_P39981	HMGCS1	NM_002130
A_33_P33628	ZFP30	NM_014898
A_23_P50456	POLD1	NM_002691
A_23_P36214	DNER	NM_139072
A_23_P18505	GAB1	NM_207123
A_23_P18748	TMPRSS11E	NM_014058
A_23_P14469	RAD1	NM_002853
A_23_P26883	MMP28	NM_024302

A_23_P10473	STT3A	NM_152713
A_24_P35757	KIAA1370	NM_019600
A_32_P43728	FAM110C	ENST00000327669
A_23_P17914	PNPLA3	NM_025225
A_23_P14104	ZNF688	NM_145271
A_33_P33998	LASS6	NM_203463
A_33_P33798	HES3	NM_001024598
A_33_P32875	KIAA1949	NM_133471
A_24_P34208	WWP2	NM_199423
A_23_P93082	NUS1	NM_138459
A_23_P39406	PTRF	NM_012232
A_33_P33409	AKTIP	NM_001012398
A_23_P72680	IFT81	NM_014055
A_24_P21831	ZNF26	NM_019591
A_23_P16935	STX17	NM_017919
A_24_P92256	SGSM2	NM_014853
A_24_P38532	PEAR1	NM_001080471
A_24_P26801	MEAF6	NM_022756
A_23_P40333	EXPH5	NM_015065
A_23_P36476	C20orf94	NM_001009608
A_23_P36383	FBXO3	NM_033406
A_33_P33810	CSTL1	NM_138283
A_32_P14459	MACC1	NM_182762
A_23_P20828	ZNF304	NM_020657
A_23_P19987	IGF2BP3	NM_006547
A_23_P68408	ZHX3	NM_015035
A_32_P18602	ANP32A	NM_006305
A_23_P90223	ZNF585B	NM_152279
A_24_P14403	FLJ25328	NR_024335
A_32_P64475	C1orf55	NM_152608
A_23_P79816	EMILIN3	NM_052846
A_23_P35492	CHD9	NM_025134
A_23_P11522	HAX1	NM_006118
A_23_P16877	CCDC146	NM_020879
A_23_P37237	CHURC1	NM_145165
A_23_P25212	TRIP6	NM_003302
A_23_P39635	NIN	NM_020921
A_23_P35167	TK2	NM_004614
A_24_P71366	BZW1	NM_014670
A_23_P20668	WWP2	NM_199424
A_23_P14508	TTC9	NM_015351
A_23_P53668	NFYB	NM_006166
A_23_P7201	MED28	ENST00000237380
A_23_P10988	ITIH4	NM_002218
A_24_P39090	PRX	NM_181882
A_24_P4110	ELP3	NM_018091
A_23_P12364	UGCG	ENST00000374279
A_23_P97005	JAK1	NM_002227
A_23_P30582	SLC9A3	NM_004174

A_23_P83234	ZBTB6	NM_006626
A_23_P12576[illegible]	HCFC1	NM_005334
A_23_P15421[illegible]	ITGB6	NM_000888
A_23_P41723[illegible]	FEM1C	NM_020177
A_23_P14171[illegible]	C18orf10	NM_015476
A_32_P11924[illegible]	FOXD4	NM_207305
A_23_P91891	COPB2	NM_004766
A_23_P21004[illegible]	HDAC4	NM_006037
A_32_P22251[illegible]	UBXN7	ENST00000296328
A_23_P88435	FOXN3	NM_005197
A_24_P93754	KDM4C	NM_001146694
A_23_P21851[illegible]	ZNF222	NM_013360
A_33_P35312[illegible]	PCOTH	NM_001135816
A_23_P20760[illegible]	PSMD11	NM_002815
A_23_P11545[illegible]	RPL22	NM_000983
A_33_P33722[illegible]	ZNF81	NM_007137
A_32_P13105[illegible]	ZNF148	NM_021964
A_23_P10120[illegible]	CYB5A	NM_001914
A_33_P32611[illegible]	NCBP1	NM_002486
A_23_P86283	LAPTM5	NM_006762
A_24_P40243[illegible]	TGFB2	NM_003238
A_24_P35348[illegible]	KLHL4	NM_057162
A_24_P17551[illegible]	TXN	NM_003329
A_32_P58163	C15orf29	NM_024713
A_23_P16233[illegible]	CNOT2	NM_014515
A_33_P32718[illegible]	SIRT2	NM_012237
A_23_P95553	WDR6	NM_018031
A_32_P46214	SLC9A9	NM_173653
A_33_P32243[illegible]	DLG1	NM_001098424
A_23_P98898	CDK2	NM_001798
A_32_P14471[illegible]	NR2C2AP	NM_176880
A_23_P98254	ARL2	NM_001667
A_23_P3767	NFAT5	NM_138714
A_23_P22134	BNC1	NM_001717
A_33_P33312[illegible]	G3BP1	ENST00000394123
A_23_P14658[illegible]	C9orf30	NM_080655
A_24_P26108[illegible]	OSGIN2	NM_004337
A_23_P14782[illegible]	RAP2C	NM_021183
A_23_P11083[illegible]	IRX4	NM_016358
A_23_P31578[illegible]	RFXAP	NM_000538
A_23_P3841	TRAP1	NM_016292
A_33_P33135[illegible]	CHRFAM7A	NM_139320
A_24_P57700	ZHX3	NM_015035
A_33_P33776[illegible]	SPAM1	NM_001174044
A_32_P34827[illegible]	ZNF699	NM_198535
A_24_P32307[illegible]	GNRH2	NM_178332
A_23_P16589[illegible]	TAF1B	NM_005680
A_23_P33981[illegible]	ARRDC4	NM_183376
A_23_P21341[illegible]	SEMA5A	NM_003966

A_24_P70303	CACNG4	NM_014405
A_24_P25252	ANKRD12	NM_015208
A_23_P13291!	FAM114A1	NM_138389
A_23_P13907	IGF1	NM_000618
A_23_P14002!	UBL3	NM_007106
A_33_P32990	C1orf159	ENST00000379319
A_33_P34066	ZFP62	NM_152283
A_32_P77369	SC4MOL	AV701505
A_23_P86550	EIF3A	NM_003750
A_32_P61657	ZNF33B	NM_006955
A_24_P35796	GRAMD2	NM_001012642
A_23_P15051	E4F1	NM_004424
A_23_P7992	HIST1H1D	NM_005320
A_23_P10574	APPL2	NM_018171
A_32_P72417	TSHZ3	NM_020856
A_23_P12010!	KCNS3	NM_002252
A_23_P36376!	KRT86	NM_002284
A_32_P2368	DNAJB3	NM_001001394
A_23_P34170	ZNF484	NM_001007101
A_23_P81401	SQSTM1	NM_003900
A_24_P48983	CCDC120	NM_033626
A_23_P21497	SEC63	NM_007214
A_23_P16445	TBX2	NM_005994
A_33_P33388!	LOC401233	NR_033884
A_32_P4262	C20orf26	NM_015585
A_24_P12281	AP4S1	NM_007077
A_33_P33393!	SH3YL1	NM_015677
A_24_P92189	HOOK1	NM_015888
A_24_P32354!	MYH14	NM_001077186
A_24_P19682	HLA-DQA1	NM_002122
A_23_P16894	ZHX2	NM_014943
A_23_P27827	ZBTB7A	NM_015898
A_33_P33798	C14orf104	NM_001083908
A_23_P46378	PIGV	NM_017837
A_33_P32563	SELPLG	NM_003006
A_23_P42758	FGF19	NM_005117
A_24_P34533	RABIF	NM_002871
A_24_P17464	PDLIM5	NM_006457
A_23_P57052	EYA2	NM_005244
A_23_P39386	HCST	NM_014266
A_24_P34217	ERAP2	NM_022350
A_23_P41489!	TTC17	NM_018259
A_33_P33907!	TRIM36	NM_001017397
A_23_P21681	CDKN2B	NM_004936
A_23_P50158	NFYA	NM_002505
A_24_P36724	KRTAP19-1	NM_181607
A_24_P12618	NR2C2AP	NM_176880
A_23_P15760	DDHD2	NM_015214
A_23_P10817	PSG6	NM_002782

A_33_P33829!	CASP8AP2	NM_012115
A_23_P32177	LHX6	NM_014368
A_23_P50418	ZNF791	NM_153358
A_24_P24282(	TSN	NM_004622
A_23_P89884	TRIM28	NM_005762
A_24_P13871:	HEATR3	NM_182922
A_23_P88347	FERMT2	NM_006832
A_23_P32756:	LHX9	NM_020204
A_23_P16107(	TOR1AIP1	NM_015602
A_24_P20772:	MED15	NM_001003891
A_23_P30687	SERPINB9	NM_004155
A_23_P25674	CKB	NM_001823
A_23_P15475i	ZNF337	NM_015655
A_33_P32556:	ZNF780A	NM_001142579
A_23_P56553	METTL8	NM_024770
A_23_P28075	GPX4	NM_001039848
A_24_P24260!	KLHL12	NM_021633
A_23_P46337	C1orf107	NM_014388
A_23_P13740	NAV3	NM_014903
A_24_P36518(	DSEL	NM_032160
A_32_P24165	FANCD2	NM_001018115
A_33_P32667:	SYTL4	NM_080737
A_32_P44869(	C5orf41	NM_153607
A_32_P80624	NR1D2	NM_005126
A_24_P32864(	RBM5	NM_005778
A_23_P20689!	TMEM159	NM_020422
A_23_P80831	TMEM41A	NM_080652
A_33_P33313:	EPHB2	NM_004442
A_24_P15708:	CASP8	NM_033355
A_24_P67096	ABCA5	NM_018672
A_23_P16150:	UBTD1	NM_024954
A_23_P30030:	JMJD1C	NM_004241
A_24_P19685:	TLN1	NM_006289
A_23_P21806i	PLEKHA5	NM_019012
A_23_P10171(	ZNF155	NM_003445
A_23_P12402·	MED10	NM_032286
A_23_P35909:	SMAD3	U68019
A_23_P21575:	NDUFA5	NM_005000
A_24_P26235!	PHB	NM_002634
A_32_P16749:	SAP18	NM_005870
A_23_P62320	KAL1	NM_000216
A_23_P50338	TUBB4	NM_006087
A_24_P21783·	HIST1H3D	NM_003530
A_23_P64954	USP5	NM_003481
A_23_P8913	CA2	NM_000067
A_23_P31372i	FAM98A	NM_015475
A_23_P17028(	ATP10D	NM_020453
A_23_P13183	EXT2	NM_000401
A_23_P20824(	ZNF160	BC000807

A_23_P21230	TRIM37	NM_015294
A_23_P97584	DNTTIP2	NM_014597
A_23_P11763	ATXN3	NM_004993
A_23_P42348	TYSND1	NM_173555
A_23_P35966	PLAC2	NR_027064
A_23_P34710	EGLN1	NM_022051
A_23_P17354	GDAP1L1	NM_024034
A_24_P41871	BMPR1A	NM_004329
A_32_P56759	PARP14	NM_017554
A_23_P30489	BDKRB2	NM_000623
A_23_P12009	USP39	NM_006590
A_23_P30243	ERAP2	NM_022350
A_32_P22109	TSHZ2	NM_173485
A_23_P31393	PPIF	AY358854
A_23_P11501	ADAMTSL4	NM_019032
A_23_P34452	LOR	NM_000427
A_23_P96899	PGLYRP3	NM_052891
A_23_P66992	ONECUT2	NM_004852
A_23_P15673	PHF1	NM_024165
A_23_P35961	RPF1	NM_025065
A_23_P15505	APOL6	NM_030641
A_23_P85862	GON4L	NM_032292
A_23_P45902	RBBP4	NM_005610
A_32_P14189	SUMO2	NM_006937
A_23_P21031	DTNB	NM_183360
A_24_P92499	AHDC1	NM_001029882
A_23_P13034	KCTD1	NM_198991
A_23_P11436	MECP2	NM_004992
A_24_P79054	TGFB1	NM_000660
A_24_P23564	CUEDC2	NM_024040
A_23_P96285	REEP1	NM_022912
A_23_P25325	GCET2	NM_001008756
A_24_P34003	RNF128	NM_194463
A_23_P75921	TRAF6	NM_145803
A_23_P42451	RANBP9	NM_005493
A_23_P31921	ASS1	NM_000050
A_24_P34719	DENR	NM_003677
A_23_P34072	XPOT	NM_007235
A_24_P95154	TUSC3	NM_178234
A_24_P27373	TTC9	NM_015351
A_32_P39969	ZNF81	NM_007137
A_24_P11460	TBL1XR1	NM_024665
A_23_P15511	DDX5	NM_004396
A_23_P13249	IL17RD	NM_017563
A_33_P33544	KRTAP12-2	NM_181684
A_23_P14006	FBXL3	NM_012158
A_32_P67256	SDHA	NM_004168
A_23_P15322	ZNF616	BC032805
A_24_P31790	SORBS1	NM_001034954

A_32_P197011	RFC3	NM_181558
A_32_P73830	CSDE1	NM_001007553
A_23_P386346	ATAD3B	NM_031921
A_32_P162547	LIMCH1	NM_014988
A_24_P306214	KIAA1609	NM_020947
A_23_P380724	FASTKD5	NM_021826
A_23_P12874	GTPBP4	NM_012341
A_24_P175187	SAMD9	NM_017654
A_33_P327001	NCOA2	NM_006540
A_32_P301559	NDUFA11	NM_175614
A_23_P320705	CRYGD	BC117338
A_23_P32896	FCGBP	NM_003890
A_23_P86390	NRP1	NM_003873
A_33_P327464	CAMK2A	NM_015981
A_23_P115842	CCAR1	NM_018237
A_23_P134078	CDYL	NM_004824
A_23_P1145	MTPAP	NM_018109
A_33_P334375	ZNF567	NM_152603
A_23_P146943	ATP1B1	NM_001677
A_23_P31437	CYP2W1	NM_017781
A_24_P74160	SNRPD2	NM_177542
A_32_P426233	CSPG4P5	AB067507
A_23_P304991	HLCS	NM_000411
A_23_P14698	GTF2A2	NM_004492
A_24_P311036	MYOG	NM_002479
A_33_P321631	ZCCHC14	NM_015144
A_33_P354876	C1orf46	CU449054
A_24_P128402	USP48	NM_032236
A_23_P147115	ZNF136	NM_003437
A_23_P355455	TBC1D5	NM_014744
A_23_P395582	ZFP42	NM_174900
A_32_P331618	ZKSCAN1	NM_003439
A_33_P325148	MAPKSP1	NM_021970
A_23_P145247	ZNF187	NM_001023560
A_23_P64019	MTMR2	NM_201278
A_32_P55939	RPL27A	NM_000990
A_23_P23428	FDPS	NM_002004
A_23_P24709	OSBP	NM_002556
A_23_P133868	ZKSCAN4	NM_019110
A_32_P88791	GTF2I	NM_032999
A_23_P201547	NTNG1	NM_014917
A_24_P22943	GIPC3	NM_133261
A_23_P328511	HSBP1	NM_001537
A_24_P76158	DOCK11	NM_144658
A_32_P181020	DOM3Z	NM_005510
A_24_P179277	TSPYL1	AK096882
A_24_P278552	MPZL2	NM_005797
A_23_P24114	PLA2G12B	NM_032562
A_23_P17620	CYYR1	NM_052954

A_24_P23225[illegible]	SLC1A1	NM_004170
A_23_P14494[illegible]	SMAD5	NM_001001419
A_32_P22782[illegible]	PID1	NM_017933
A_32_P64016	RUNDC2C	NR_002939
A_24_P18634[illegible]	ZFP14	NM_020917
A_33_P32906[illegible]	HELT	NM_001029887
A_23_P32670[illegible]	OVOL1	NM_004561
A_33_P32097[illegible]	ARGFX	NM_001012659
A_24_P41732[illegible]	ZNF530	NM_020880
A_24_P40732[illegible]	PPM1A	NM_177951
A_32_P63174	PDIA3P	NR_002305
A_23_P26084	SELS	NM_018445
A_24_P30398[illegible]	BMI1	NM_005180
A_33_P32214[illegible]	NTNG1	ENST00000370068
A_33_P36414[illegible]	MYEF2	NM_016132
A_23_P16258[illegible]	VDR	NM_001017535
A_23_P39925	DYSF	NM_003494
A_24_P92136[illegible]	CALD1	NM_033138
A_23_P42431[illegible]	TCF20	NM_005650
A_32_P22394	MAP7D1	NM_018067
A_23_P32758	TEAD4	NM_003213
A_23_P40460[illegible]	C5orf41	NM_153607
A_32_P34183[illegible]	GHR	NM_000163
A_32_P39542[illegible]	VANGL2	NM_020335
A_24_P22697[illegible]	ZNF365	NM_014951
A_23_P25950[illegible]	ZFP42	NM_174900
A_32_P44694[illegible]	TRIM24	ENST00000343526
A_24_P62102[illegible]	GMEB1	ENST00000373816
A_32_P34812[illegible]	KY	NM_178554
A_23_P32009[illegible]	CSDC2	NM_014460
A_23_P12897[illegible]	BATF	NM_006399
A_23_P12189[illegible]	PARP8	NM_024615
A_23_P57733	DRD3	NM_033663
A_33_P32292[illegible]	HIST2H2BF	NM_001024599
A_23_P10042[illegible]	ZCCHC14	NM_015144
A_23_P62139	PQBP1	NM_001167989
A_23_P51936	TNFRSF9	NM_001561
A_23_P64808	HOXC13	NM_017410
A_23_P32536	GNG2	NM_053064
A_23_P42392[illegible]	SS18L1	NM_198935
A_32_P34444	FHOD3	NM_025135
A_32_P22818[illegible]	NRK	NM_198465
A_23_P25155[illegible]	ARL4C	NM_005737
A_23_P20622[illegible]	VPS13C	NM_020821
A_24_P33344[illegible]	MORF4L2	NM_012286
A_24_P11088[illegible]	KIAA2018	NM_001009899
A_33_P32784[illegible]	CHCHD8	NM_016565
A_23_P90523	OCEL1	NM_024578
A_23_P98321	EI24	AF010313

A_23_P20694!	ACOX1	NM_004035
A_23_P10806!	LGALS7B	NM_001042507
A_32_P19652(	LSM11	ENST00000286307
A_23_P21777!	MSL3	NM_078629
A_33_P32719!	ARFGEF2	NM_006420
A_23_P11352!	GTPBP6	NM_012227
A_23_P75147	SPAG6	NM_012443
A_23_P15786!	TNC	NM_002160
A_23_P73699	MED12	NM_005120
A_32_P48455!	ZNF154	NM_001085384
A_24_P30037!	PI16	NM_153370
A_24_P18832!	UCHL5	NM_015984
A_24_P30462!	VASH1	NM_014909
A_23_P99473	UPF3A	NM_023011
A_23_P20540!	MIA2	NM_054024
A_32_P80678	ADAM9	NM_003816
A_33_P32527!	PLAC9	NM_001012973
A_23_P21907!	SAMD9	NM_017654
A_23_P16621!	GABPA	NM_002040
A_23_P10527(	ZNF84	NM_003428
A_23_P20574(	EML1	NM_001008707
A_24_P23973!	B4GALT5	NM_004776
A_23_P14907!	MAP7D1	NM_018067
A_23_P15374!	IFI30	NM_006332
A_23_P23815	SLC30A1	NM_021194
A_24_P34611	SIX3	NM_005413
A_23_P10776!	ZNF266	NM_006631
A_23_P38820(	KDELC2	NM_153705
A_23_P16385(	CREBBP	NM_004380
A_24_P93534!	ENST0000037!	ENST00000378432
A_23_P40184	CDH22	NM_021248
A_23_P13029!	ANKRD12	NM_015208
A_24_P25299(	FOLR3	NM_000804
A_24_P54845!	CRYZ	NM_001130043
A_23_P17911	NFAM1	NM_145912
A_23_P10489!	EIF4G2	NM_001418
A_23_P67521	IRGC	NM_019612
A_23_P16558!	PLCL1	NM_006226
A_24_P20197!	TEP1	NM_007110
A_23_P12394!	TGFBR1	NM_004612
A_23_P2541	CUX2	NM_015267
A_33_P32624!	ZNF503	NM_032772
A_32_P31019!	GREB1L	NM_001142966
A_32_P20580	VPS13D	NM_015378
A_23_P34176	WWC3	NM_015691
A_23_P43016!	EXOC8	NM_175876
A_24_P23840!	ZNF644	NM_201269
A_23_P89902	RTN2	NM_005619
A_23_P15970!	FOXP3	NM_014009

A_23_P15235(	ZNF200	NM_003454
A_24_P14357(	SIRPB1	NM_006065
A_23_P10182(	LPAR2	NM_004720
A_24_P33301(	RNF24	NM_007219
A_24_P19912(	TCF15	NM_004609
A_23_P33245(	HDAC9	NM_058177
A_23_P11981(	GPD2	NM_000408
A_24_P90097	ADD3	NM_016824
A_23_P11543	FUCA1	NM_000147
A_23_P58796	RGMB	NM_001012761
A_23_P10802(	ZNF146	NM_007145
A_23_P13045(	MZF1	NM_198055
A_32_P19142(	FOXF2	NM_001452
A_32_P43465	ZNF182	NM_006962
A_32_P31829(	PROX2	NM_001080408
A_23_P15586(	PGRMC2	NM_006320
A_23_P23421	ARHGEF11	NM_198236
A_23_P36454(	C12orf60	NM_175874
A_33_P33420(	ZHX3	NM_015035
A_23_P13690(	HMGN5	NM_030763
A_23_P88420	NEDD8	NM_006156
A_23_P15798	KRTAP4-12	NM_031854
A_23_P32825(	FHDC1	NM_033393
A_23_P60101	ZNF696	NM_030895
A_32_P16905(	METTL8	NM_024770
A_32_P16182(	WWC1	NM_015238
A_24_P16954(	ZNF17	NM_006959
A_23_P11534(	LGTN	NM_006893
A_23_P38393(	SPIC	NM_152323
A_32_P82807	SETD8	NM_020382
A_23_P36740(	PCBD1	NM_000281
A_33_P34016(	GIT1	NM_014030
A_23_P897	C1orf116	NM_023938
A_24_P18038(	NIPBL	NM_015384
A_24_P38307(	AASDH	NM_181806
A_24_P92654(	C11orf82	AK058145
A_23_P10977(	ZBTB11	NM_014415
A_23_P50013(	KANK1	NM_153186
A_33_P32535(	HIST2H2BF	NM_001161334
A_23_P20365(	PICALM	NM_007166
A_32_P31311(	WWP1	NM_007013
A_23_P21419(	RNGTT	NM_003800
A_33_P32329(	F2RL3	NM_003950
A_23_P94493	TLE4	NM_007005
A_32_P12424(	OLIG3	NM_175747
A_23_P16640(	OSM	NM_020530
A_24_P17425(	CCDC93	NM_019044
A_24_P31216(	SLC25A40	ENST00000341119
A_23_P48936	SMAD3	NM_005902

A_24_P22610[illegible]	RBM47	NM_019027
A_23_P36077[illegible]	NRG1	NM_013960
A_23_P10812[illegible]	ZNF223	NM_013361
A_32_P35790[illegible]	FAM149B1	ENST00000242505
A_23_P77079	MYEF2	NM_016132
A_23_P63369	TAL1	NM_003189
A_24_P31350[illegible]	PLK1	NM_005030
A_23_P31315	CBX3	NM_016587
A_23_P55926	NR1H2	NM_007121
A_23_P16100[illegible]	NR5A2	NM_205860
A_33_P32854[illegible]	C1orf68	NM_001024679
A_23_P11137[illegible]	MRS2	NM_020662
A_23_P33662[illegible]	TCHH	NM_007113
A_33_P34198[illegible]	RPS29	NM_001032
A_23_P33895[illegible]	S100PBP	NM_022753
A_23_P10785[illegible]	FIZ1	NM_032836
A_24_P20424[illegible]	ANXA2P1	NR_001562
A_33_P34144[illegible]	GPHN	NM_020806
A_23_P77401	CPPED1	NM_018340
A_32_P20420[illegible]	SIX4	NM_017420
A_23_P25319[illegible]	RPL15	NM_002948
A_24_P25521[illegible]	MYO5A	NM_000259
A_23_P11129[illegible]	RPP40	NM_006638
A_24_P29676[illegible]	ZNF211	NM_198855
A_23_P43061	ZNF34	NM_030580
A_23_P14466[illegible]	CMBL	NM_138809
A_23_P73747	ARMCX2	NM_014782
A_23_P11493[illegible]	MAEL	NM_032858
A_23_P25069[illegible]	TSGA13	NM_052933
A_23_P2216	KRR1	NM_007043
A_23_P54165	KLHDC2	NM_014315
A_23_P13950[illegible]	ERGIC2	NM_016570
A_23_P48581	C14orf93	NM_021944
A_33_P33031[illegible]	MYNN	NM_001185119
A_24_P25204[illegible]	PTP4A1	NM_003463
A_23_P14918[illegible]	ZP4	NM_021186
A_23_P10687[illegible]	PMFBP1	NM_031293
A_23_P21173[illegible]	UBP1	NM_014517
A_24_P23130[illegible]	PGLYRP4	NM_020393
A_23_P73135	USHBP1	NM_031941
A_24_P24264[illegible]	CTSS	NM_004079
A_23_P26697	TRIM47	NM_033452
A_23_P11295[illegible]	ATF7IP	NM_018179
A_23_P88083	CDC16	NM_003903
A_23_P38466[illegible]	MPP7	NM_173496
A_24_P14902[illegible]	SCAMP1	NM_004866
A_24_P37903	LOX	NM_002317
A_24_P29563[illegible]	PRR5L	NM_024841
A_33_P34158[illegible]	THBS1	NM_003246

A_24_P93315[illegible]	HOXB6	S69023
A_24_P93031[illegible]	ZKSCAN4	AK056698
A_24_P12455[illegible]	CCND1	NM_053056
A_32_P49435	AHRR	NM_020731
A_23_P29036	IFNGR2	NM_005534
A_23_P10203[illegible]	COQ10B	NM_025147
A_24_P11570[illegible]	COX15	NM_078470
A_23_P12718[illegible]	TACC2	NM_206862
A_23_P18772	SFRS12	NM_139168
A_33_P32567[illegible]	CARM1	NM_199141
A_23_P13436[illegible]	ETV1	NM_004956
A_23_P15498[illegible]	ZNF70	NM_021916
A_23_P30575[illegible]	ABHD3	NM_138340
A_23_P10065[illegible]	ZBTB4	NM_020899
A_24_P35310[illegible]	ZNF599	NM_001007248
A_23_P13137[illegible]	PQLC3	NM_152391
A_23_P21605[illegible]	FAM83A	NM_032899
A_33_P33479[illegible]	HSF1	NM_005526
A_33_P33279[illegible]	ZNF615	NM_001199324
A_23_P19313	TBP	NM_003194
A_23_P43988	DPYD	NM_000110
A_23_P75402	NDUFS3	NM_004551
A_32_P10484[illegible]	MED12L	NM_053002
A_33_P33129[illegible]	CLASP1	NM_015282
A_33_P33165[illegible]	RPS6KA5	NM_182398
A_23_P11668[illegible]	SMARCC2	NM_139067
A_33_P33656[illegible]	UBA5	ENST00000356232
A_33_P32089[illegible]	ZNF683	NM_001114759
A_23_P21162[illegible]	NUP50	NM_007172
A_23_P16020[illegible]	ZNF643	NM_023070
A_24_P28578	EPS15	NM_001981
A_23_P42899[illegible]	NCOA2	NM_006540
A_23_P56170	MED16	NM_005481
A_33_P33084[illegible]	RHOB	NM_004040
A_23_P20034[illegible]	PTGS2	NM_000963
A_23_P24784	TNNI2	NM_003282
A_23_P59921	SUB1	NM_006713
A_24_P10877[illegible]	KCTD17	NM_024681
A_24_P11134[illegible]	CASP9	NM_001229
A_23_P21346	PCGF3	NM_006315
A_24_P17066[illegible]	ARID5B	NM_032199
A_32_P52070[illegible]	KRT74	NM_175053
A_32_P22535[illegible]	CPEB2	NM_182485
A_23_P60000	TTC35	NM_014673
A_23_P98022	SIRT1	NM_012238
A_23_P15448[illegible]	PNPT1	NM_033109
A_23_P22647	FAM155B	NM_015686
A_23_P21150[illegible]	KDELR3	NM_016657
A_24_P19452[illegible]	CD99L2	NM_031462

A_23_P34392	HIST2H2AB	NM_175065
A_23_P53891	KLF5	NM_001730
A_23_P43496	LIMD1	NM_014240
A_24_P15354	RPS6KL1	NM_031464
A_24_P99679	POLR2A	NM_000937
A_23_P21060	ZNF217	NM_006526
A_24_P21057	SLTM	NM_024755
A_23_P46947	CNNM1	NM_020348
A_23_P42432	RIOK1	NM_153005
A_23_P20341	FADS1	NM_013402
A_23_P33526	PART1	NR_028508
A_24_P45431	HNRNPA1	NM_031157
A_23_P79221	ACVR1	NM_001105
A_33_P33649	ZNF662	NM_207404
A_23_P7194	LGI2	NM_018176
A_33_P32381	MYO18A	NM_078471
A_23_P10993	HEMK1	NM_016173
A_33_P33823	KRTAP3-1	NM_031958
A_24_P17793	RGNEF	NM_001080479
A_23_P11995	SLC8A1	NM_021097
A_23_P36578	GRHL1	NM_198182
A_23_P33444	SNTB2	NM_006750
A_32_P48658	FLJ45983	NR_024256
A_23_P31661	GLIS1	NM_147193
A_23_P35431	GPR161	NM_153832
A_23_P83751	C11orf63	NM_024806
A_23_P12673	IL10	NM_000572
A_23_P20294	ZBTB44	NM_014155
A_23_P11153	GLI3	NM_000168
A_24_P2584	C1orf63	BC065040
A_23_P38030	PYDC1	NM_152901
A_23_P39172	DR1	NM_001938
A_23_P20439	AACS	NM_023928
A_24_P16841	PRDX2	NM_181738
A_33_P33777	LOC10012911	AK123839
A_23_P16467	ZNF225	NM_013362
A_24_P21248	MCTP1	NM_024717
A_23_P14891	CPT2	NM_000098
A_24_P93074	EPHA10	NM_001099439
A_33_P32148	KDELC2	NM_153705
A_23_P94412	PDCD1LG2	NM_025239
A_33_P33936	GLTSCR2	NM_015710
A_33_P32389	ONECUT3	NM_001080488
A_23_P33258	KIAA1107	NM_015237
A_33_P33190	ZZZ3	NM_015534
A_23_P99700	MNAT1	NM_002431
A_33_P32668	DSC3	NM_001941
A_24_P89708	IMPDH1	NM_000883
A_23_P13347	GPX3	NM_002084

A_24_P16599	GATA2	NM_032638
A_23_P92727	RAI14	NM_015577
A_24_P23067	SOCS2	NM_003877
A_23_P58835	F2RL1	NM_005242
A_23_P13075	DBP	NM_001352
A_23_P5064	CADM4	NM_145296
A_23_P30328	RPTN	NM_001122965
A_23_P4096	CA4	NM_000717
A_23_P38636	HDX	NM_144657
A_23_P16354	TAF1C	NM_005679
A_23_P39456	ZNF556	NM_024967
A_23_P43163	USP32	NM_032582
A_33_P33022	NR2C1	NM_001032287
A_32_P51648	UBE3C	NM_014671
A_33_P32900	ZNF394	NM_032164
A_23_P11165	SHH	NM_000193
A_33_P33159	CCDC103	NM_213607
A_33_P33177	KRTAP4-7	NM_033061
A_24_P40606	RNF144B	NM_182757
A_23_P12659	S100A11	NM_005620
A_33_P32995	SCXA	NM_001008271
A_23_P40807	ADPRH	NM_001125
A_23_P12077	SLC25A1	NM_005984
A_23_P40828	CCDC79	NM_001136505
A_32_P39813	KRBA2	NM_213597
A_24_P13680	RFC1	NM_002913
A_23_P32860	SLC24A6	NM_024959
A_23_P38305	APPBP2	NM_006380
A_24_P17801	MYC	M13930
A_33_P32969	RUFY4	NM_198483
A_23_P33484	ARL15	NM_019087
A_23_P75002	MKX	NM_173576
A_23_P66844	KRTAP4-7	NM_033061
A_33_P35020	CHORDC1	NM_012124
A_23_P15641	CAP2	NM_006366
A_33_P33875	FEZF2	NM_018008
A_23_P10902	KCNK15	NM_022358
A_23_P25210	RIPK2	NM_003821
A_33_P33153	SLFN11	NM_001104587
A_23_P37296	A2ML1	NM_144670
A_23_P13317	TMEM165	NM_018475
A_23_P21374	CXCL14	NM_004887
A_23_P42874	FAM84B	NM_174911
A_23_P73702	MED12	NM_005120
A_23_P748	IRF6	NM_006147
A_23_P86196	SLC44A3	NM_152369
A_23_P14408	B4GALT4	NM_212543
A_23_P20394	DDX11	NM_030653
A_23_P20385	ERBB3	NM_001005915

A_23_P20504	ANKRD10	NM_017664
A_23_P20459	LTA4H	NM_000895
A_23_P16375	KCTD5	NM_018992
A_23_P86623	ENTPD7	NM_020354
A_23_P20802	DSC3	NM_024423
A_33_P33688	NBPF1	NM_017940
A_33_P32713	THAP3	NM_138350
A_24_P11937	YLPM1	NM_019589
A_23_P33465	CRELD2	NM_024324
A_23_P38101	WBSCR27	NM_152559
A_33_P32465	MAT2B	NM_182796
A_23_P21783	FAM129A	NM_052966
A_32_P2333	SUB1	NM_006713
A_23_P35595	C10orf10	NM_007021
A_23_P83923	ZNF672	NM_024836
A_23_P10731	SDF2	NM_006923
A_33_P32586	SCD5	NM_024906
A_23_P84872	SECISBP2	NM_024077
A_24_P29822	NCKAP5	NM_207481
A_23_P36275	PRDM5	NM_018699
A_23_P42257	IER3	NM_003897
A_23_P16743	MGAT5	NM_002410
A_23_P24260	ENTPD1	NM_001776
A_23_P14339	PLUNC	NM_016583
A_23_P78750	SLC17A7	NM_020309
A_24_P40445	ITGB1BP3	NM_170678
A_23_P85742	CREG1	NM_003851
A_23_P37574	MORF4L1	NM_206839
A_23_P98941	MORN3	NM_173855
A_24_P13472	TFAM	NM_003201
A_23_P12758	NNMT	NM_006169
A_32_P14746	RPS15A	NM_001019
A_33_P33374	ZNF705A	NM_001004328
A_23_P36989	TMEM158	NM_015444
A_23_P10031	MID1	NM_000381
A_23_P83659	XPNPEP2	NM_003399
A_32_P11859	B3GAT2	ENST00000230053
A_33_P36765	C21orf122	NR_027292
A_23_P41471	ZNF528	NM_032423
A_23_P4793	ZNF581	NM_016535
A_32_P40499	ZNF549	NM_153263
A_23_P35652	TRIM5	NM_033092
A_32_P23076	C20orf94	NM_001009608
A_23_P36393	HSPA4L	NM_014278
A_23_P13789	OXCT2	NM_022120
A_23_P10473	STT3A	NM_152713
A_23_P46202	PEAR1	NM_001080471
A_23_P60479	DNAJA1	NM_001539
A_24_P39704	ST6GAL1	NM_173216

A_24_P98411	HSPA5	NM_005347
A_24_P13590:	RPS2	NM_002952
A_23_P14318	MYBL2	NM_002466
A_32_P38911	HEATR5B	NM_019024
A_23_P9056	RB1CC1	NM_014781
A_32_P83845	HEY1	NM_001040708
A_23_P9465	FPGS	NM_004957
A_23_P21886	PBRM1	NM_018313
A_24_P25207	BTN3A2	NM_007047
A_23_P47800	DIABLO	NM_019887
A_24_P33268	ZNF761	NM_001008401
A_23_P39122	MANEAL	NM_152496
A_23_P42706	HOXA11	NM_005523
A_32_P10777	MBD2	ENST00000256429
A_32_P12991	ZNF252	BC065734
A_23_P14019	KIAA0125	NR_026800
A_24_P41936	C14orf145	BC036939
A_32_P52490	C11orf86	NM_001136485
A_24_P27101	PPP4R1	NM_001042388
A_33_P33201	FAM150B	NM_001002919
A_23_P11849	TOM1L1	NM_005486
A_32_P23418	HES5	NM_001010926
A_33_P32261	NR2E3	NM_016346
A_23_P47527	C11orf10	NM_014206
A_23_P56135	C19orf61	NM_019108
A_23_P20002	ANKMY2	NM_020319
A_23_P20774	THRA	NM_003250
A_23_P72025	SLC25A20	NM_000387
A_23_P40909	ANO4	NM_178826
A_24_P10577	ACTR3	NM_005721
A_24_P34129	ZNF665	NM_024733
A_24_P30442	IGF1	NM_000618
A_23_P31181	GHRH	NM_021081
A_24_P15576	AKAP13	NM_006738
A_24_P11714	KIR3DL1	NM_013289
A_23_P27247	MED13	NM_005121
A_23_P16139	MXI1	NM_130439
A_33_P34078	ANKRD22	NM_144590
A_23_P11153	GLI3	NM_000168
A_33_P32353	MTMR3	NM_021090
A_23_P17909	NFAM1	NM_145912
A_24_P13200	MSX2	NM_002449
A_24_P17917	ICK	NM_016513
A_23_P10585	SLC46A3	NM_181785
A_24_P14462	C6orf35	NM_018452
A_23_P59772	CLCN1	NM_000083
A_23_P21215	NUP210	NM_024923
A_23_P21595	MYC	NM_002467
A_23_P15525	FOXP1	NM_032682

A_23_P11148(	STAG3L4	NM_022906
A_24_P20463(	ZNF716	NM_001159279
A_23_P14791(	S100A16	NM_080388
A_23_P64098	RIN1	NM_004292
A_33_P34101(	KIAA1751	NM_001080484
A_23_P31055	NUP43	NM_198887
A_33_P33059(	SNAP23	NM_003825
A_33_P32924(	WDR5	NM_017588
A_23_P75468	SCYL1	NM_001048218
A_23_P10953(	APOBEC3B	NM_004900
A_23_P36567(	ZNF385A	NM_015481
A_23_P21496(	CITED2	NM_006079
A_23_P47004	DHX32	NM_018180
A_24_P30786(	LLGL2	NM_001015002
A_32_P74254	TMEM61	NM_182532
A_23_P40634(	AFAP1L2	NM_001001936
A_23_P5761	NFE2L2	NM_006164
A_24_P33647(	NHLH1	NM_005598
A_23_P82758	ZNF596	NM_001042416
A_32_P10311	MAPKSP1	NM_021970
A_23_P66027	ZFP90	NM_133458
A_23_P80032	E2F1	NM_005225
A_23_P30291(	ZFYVE28	NM_020972
A_23_P50090(	ZNF19	NM_006961
A_24_P25530	GRIN2C	NM_000835
A_23_P47535	TAF6L	NM_006473
A_23_P38221(	OLIG3	NM_175747
A_24_P11925(	BLZF1	NM_003666
A_23_P34193(	NOG	NM_005450
A_23_P10654(	C16orf61	NM_020188
A_24_P63944(	CD59	NM_203330
A_24_P84924(	ETV3	ENST00000368192
A_24_P63950(	STX6	NM_005819
A_23_P13622(	RRM2	NM_001034
A_32_P56525	FAM115A	NM_014719
A_32_P12659(	EEPD1	NM_030636
A_23_P66988	ONECUT2	NM_004852
A_32_P16669(	HEG1	NM_020733
A_23_P25163(	HIST1H2AB	NM_003513
A_33_P33606(	ACVR1	NM_001105
A_23_P39553(	RSC1A1	NM_006511
A_23_P33655(	IL1RAP	NM_134470
A_23_P25443(	RFPL2	NM_006605
A_23_P80136	RRP1	NM_003683
A_23_P13193(	FERMT1	NM_017671
A_23_P88339	FERMT2	NM_006832
A_23_P21232(	PTPN23	NM_015466
A_23_P12624(	EIF4G3	NM_003760
A_24_P85478	ARIH1	NM_005744

A_24_P31191	BTN3A3	NM_006994
A_23_P27040	TMEM98	NM_015544
A_33_P33356	TMEM208	NM_014187
A_32_P49628	SP6	NM_199262
A_24_P17817	GGT1	NM_005265
A_23_P7451	NR2F1	ENST00000327111
A_32_P93355	SAP25	NM_001168682
A_32_P56639	ZEB2	NM_014795
A_24_P20513	FNBP1	NM_015033
A_23_P15391	MAP2	NM_002374
A_23_P72187	MED14	NM_004229
A_23_P94795	TEAD4	NM_003213
A_23_P91111	PREB	NM_013388
A_23_P53126	LMO2	NM_005574
A_24_P3783	HIST1H2BM	NM_003521
A_23_P33833	CHST7	NM_019886
A_32_P45623	SNORD31	NR_002560
A_33_P32167	EYA1	NM_000503
A_23_P33479	LRRC2	NM_024512
A_23_P16612	POLR3F	NM_006466
A_23_P45389	RAB9A	NM_004251
A_32_P83949	PRMT3	NM_005788
A_23_P50645	LOC390940	ENST00000244321
A_23_P53450	KRT82	NM_033033
A_24_P59934	OSBPL3	NM_015550
A_24_P21608	PAF1	NM_019088
A_24_P15816	SPAG1	NM_003114
A_23_P16066	FBXO42	NM_018994
A_23_P10480	ZBTB16	NM_006006
A_23_P14588	PFTK1	NM_012395
A_23_P16264	GABARAPL1	NM_031412
A_23_P86731	ZNF239	NM_005674
A_33_P32645	DCTN1	NM_004082
A_23_P10917	BFSP1	NM_001195
A_33_P32166	TMPRSS4	NM_019894
A_23_P12973	LLGL2	NM_004524
A_32_P33138	KIAA2022	NM_001008537
A_23_P10925	RBM39	NM_184234
A_24_P13435	BTBD3	NM_014962
A_24_P38031	CAMK2A	NM_015981
A_32_P51848	ZBTB46	NM_025224
A_23_P10650	LCMT2	NM_014793
A_24_P92957	RAPH1	NM_213589
A_23_P20570	RPS6KL1	AK131532
A_23_P21525	POLR2J2	NM_032959
A_23_P77073	SPPL2A	NM_032802
A_23_P10463	NDUFS5	NM_004552
A_23_P25733	KHDRBS3	NM_006558
A_32_P74485	ZNF699	ENST00000308650

A_23_P20616	TTBK2	NM_173500
A_23_P21095	FER1L4	NR_024377
A_23_P21010	CYP26B1	NM_019885
A_24_P30506	HOXB4	NM_024015
A_23_P16516	ARID3A	NM_005224
A_23_P25945	ADCK1	NM_020421
A_23_P59988	AMAC1L2	NM_054028
A_24_P16652	TNFAIP3	NM_006290
A_23_P12358	IPPK	NM_022755
A_32_P44792	ARX	NM_139058
A_23_P31338	UGCG	NM_003358
A_23_P36888	FAM113B	NM_138371
A_24_P86084	NR6A1	ENST00000487099
A_23_P53456	CORO1C	NM_014325
A_23_P96205	MBNL3	NM_018388
A_23_P38343	ZNHIT3	NM_004773
A_24_P15604	SLC39A6	NM_012319
A_23_P19192	LYPLA1	NM_006330
A_23_P13008	IFT20	NM_174887
A_33_P33259	PQBP1	NM_144495
A_23_P16425	PPY2	NR_002181
A_23_P31896	ST3GAL1	NM_003033
A_23_P15023	C11orf68	NM_031450
A_23_P35249	RHOXF2	NM_032498
A_32_P38208	ZNF805	NM_001023563
A_24_P2093	XAB2	NM_020196
A_32_P10010	REPS2	NM_004726
A_23_P12875	BDKRB1	NM_000710
A_33_P33026	HIST1H2BE	NM_003523
A_23_P15601	GOLPH3	NM_022130
A_23_P14147	SUPT6H	NM_003170
A_23_P42533	PPP4R4	NM_058237
A_23_P29047	CBR1	NM_001757
A_24_P12240	TCEB3	NM_003198
A_33_P35075	SNORD22	BM548627
A_23_P11095	FOXF2	NM_001452
A_24_P26103	PMP2	NM_002677
A_23_P36521	GPR110	NM_025048
A_23_P56969	C20orf11	NM_017896
A_23_P32463	C9orf72	NM_018325
A_23_P20653	PHKB	NM_001031835
A_24_P10251	CABIN1	NM_012295
A_23_P76460	MYF6	NM_002469
A_23_P16725	PHF17	NM_199320
A_24_P32833	ROCK2	NM_004850
A_24_P19629	MLL	NM_005933
A_24_P10829	IMPACT	NM_018439
A_23_P25841	TNIP2	NM_024309
A_24_P39156	ARNT	NM_001668

A_23_P87576	CCNT1	NM_001240
A_23_P50241	SERPINB4	NM_002974
A_23_P44586	COX5A	NM_004255
A_23_P72912	SLC4A8	NM_004858
A_24_P65121	TTC23	NM_001040655
A_24_P23391	MED8	NM_201542
A_23_P43248	TAF2	NM_003184
A_23_P16119	VIM	NM_003380
A_23_P40190	PHF19	NM_001009936
A_33_P33514	SMARCAD1	NM_001128429
A_23_P16163	RBM14	NM_006328
A_23_P28697	HAAO	NM_012205
A_24_P22953	OBFC2A	NM_001031716
A_23_P22378	SOX11	NM_003108
A_23_P11937	CYTH2	NM_004228
A_23_P31356	NCOA3	NM_181659
A_23_P55319	FLOT2	NM_004475
A_23_P41127	KAT2B	NM_003884
A_33_P33296	ZFP92	NM_001136273
A_23_P44366	PRKAG2	NM_016203
A_24_P68696	SH2D5	NM_001103161
A_24_P66027	APOBEC3B	NM_004900
A_32_P39461	SHC4	NM_203349
A_23_P16027	LRRC7	NM_020794
A_33_P33248	TRAV20	AK127936
A_32_P48988	SS18	NM_001007559
A_23_P42832	PTF1A	NM_178161
A_23_P43444	NACC2	ENST00000371753
A_32_P19237	ENPP1	NM_006208
A_23_P54205	C14orf118	NM_017926
A_23_P80817	TAGLN3	NM_013259
A_23_P76145	ARNTL2	AF256215
A_24_P26672	SF1	NM_004630
A_24_P15293	TNRC18	NM_001080495
A_23_P14114	FBXL20	NM_032875
A_23_P4637	ZNF548	NM_152909
A_23_P15024	CCDC85B	NM_006848
A_23_P67121	CBLC	NM_012116
A_23_P29897	AFF1	NM_005935
A_24_P49718	IRF2BP2	NM_182972
A_23_P99424	TPP2	NM_003291
A_23_P13614	MED24	NM_014815
A_23_P15608	GHR	NM_000163
A_23_P16363	ZNF276	NM_152287
A_32_P14388	MEMO1	NM_015955
A_24_P30546	GATAD2A	NM_017660
A_23_P34688	RBPJL	NM_014276
A_23_P10505	TPP1	NM_000391
A_23_P34832	PCGF2	NM_007144

A_33_P32588:	NOTCH2	NM_001200001
A_23_P32567(	ZNF653	NM_138783
A_24_P28552:	MAP4K3	NM_003618
A_23_P38862!	ATAD2B	NM_017552
A_23_P45345	HTATSF1	NM_014500
A_32_P47462(	IGSF22	NM_173588
A_23_P25613:	HR	NM_005144
A_32_P19332:	RICTOR	NM_152756
A_32_P43056!	ZC3H6	NM_198581
A_33_P32830-	KIAA0802	NM_015210
A_33_P32562!	KRTAP4-7	NM_033061
A_23_P3247	ANP32A	NM_006305
A_24_P40379!	NRIP2	NM_031474
A_23_P91250	ZNF334	NM_199441
A_23_P10193:	ZNF527	NM_032453
A_24_P92980:	TRA2A	AB052759
A_23_P11576:	ECD	NM_007265
A_23_P31190:	ATP10B	NM_025153
A_23_P12764:	ARHGEF12	NM_015313
A_33_P34098!	LHX1	NM_005568
A_23_P16068!	C1orf103	NM_018372
A_23_P24361	GRM5	NM_000842
A_23_P16257!	HSPB8	NM_014365
A_23_P53345	ARNTL2	NM_020183
A_23_P36184:	RIMKLB	NM_020734
A_23_P40850	CHST2	NM_004267
A_23_P12784(	ZNF214	NM_013249
A_33_P32179!	AKR7L	NM_201252
A_23_P21825:	ARHGDIG	NM_001176
A_24_P20435{	PYCR1	NM_153824
A_32_P93568{	LOC654780	XR_111602
A_33_P33813!	VAX1	NM_001112704
A_32_P18151:	ZNF730	ENST00000327867
A_23_P16305!	C14orf135	NM_022495
A_23_P11020-	CXCL5	NM_002994
A_24_P34584(	ANTXR2	NM_058172
A_24_P18036:	SNX18	NM_001145427
A_23_P12806(	ZNF26	NM_019591
A_23_P16047!	DCAF6	NM_018442
A_23_P25488:	ZYX	NM_003461
A_24_P21282(	ZNF525	NR_003699
A_32_P46159:	SVIL	NM_021738
A_23_P10715-	USP32	NM_032582
A_32_P15832:	LMO7	NM_005358
A_32_P11627	RPS27A	NM_002954
A_24_P68991	NKX6-1	NM_006168
A_23_P68505	C20orf177	NM_001190826
A_23_P20353(	TSPAN32	NM_139022
A_33_P33506!	LMX1A	NM_177398

A_23_P133475	GPX3	NM_002084
A_23_P149887	CSGALNACT2	NM_018590
A_23_P371005	ZNF227	NM_182490
A_23_P48626	ZBTB25	NM_006977
A_23_P48826	TRIM69	NM_182985
A_23_P9006	TUSC3	NM_178234
A_23_P54170	MED6	NM_005466
A_23_P151624	REM2	NM_173527
A_23_P216199	PPP1R3B	NM_024607
A_24_P256526	SP2	BC005914
A_24_P751074	ETS1	NM_005238
A_23_P127608	TMPRSS4	NM_019894
A_23_P40470	HIST1H2BE	NM_003523
A_32_P491270	CCRL1	NM_178445
A_24_P184385	LRTOMT	NM_145309
A_32_P133521	SUMO1	AL565221
A_24_P873263	SUZ12	NM_015355
A_23_P139704	DUSP6	NM_001946
A_32_P221799	HIST1H2AM	NM_003514
A_24_P28524	DBT	NM_001918
A_23_P212482	SLC9A9	NM_173653
A_23_P390343	DHX40	NM_024612
A_23_P212552	TBL1XR1	NM_024665
A_23_P209101	DMKN	NM_001126056
A_33_P335456	GPD2	NM_001083112
A_23_P161368	CYP2C8	NM_000770
A_23_P203790	OS9	NM_006812
A_23_P71067	TWIST1	NM_000474
A_24_P305678	PITPNB	NM_012399
A_23_P170873	GTPBP6	NM_012227
A_33_P322560	NFE2L2	NM_006164
A_33_P324524	TERC	NR_001566
A_23_P90839	TIA1	NM_022173
A_23_P250455	HDAC6	NM_006044
A_23_P218096	RBM26	NM_022118
A_23_P59293	RREB1	NM_001003699
A_33_P338230	PRDM16	NM_199454
A_33_P338058	EIF2C1	NM_012199
A_23_P100754	SMURF2	NM_022739
A_23_P129433	SLC9A5	NM_004594
A_23_P53817	SOX21	NM_007084
A_23_P433183	FLJ25328	NR_024335
A_33_P321643	LOC147804	NR_003148
A_23_P212545	RAB5A	NM_004162
A_23_P104372	DNAJC9	NM_015190
A_23_P201273	UBE4B	NM_006048
A_33_P330469	KRTAP5-1	NM_001005922
A_23_P35848	NADSYN1	NM_018161
A_23_P94411	PDCD1LG2	NM_025239

A_33_P34227	YEATS2	AK090720
A_23_P21578	HBP1	NM_012257
A_23_P89003	PRSS33	NM_152891
A_33_P33186	CCBL2	NM_001008661
A_23_P83007	C9orf150	NM_203403
A_24_P33945	ZNF24	NM_006965
A_23_P20284	FBXO3	NM_012175
A_24_P36667	AFF3	NM_002285
A_23_P21580	ZNF117	NM_015852
A_33_P33697	MED4	NM_014166
A_32_P10394	B4GALT4	NM_212543
A_24_P27745	UBE2K	NM_005339
A_33_P33065	EIF4G2	NM_001418
A_24_P35703	UBE2G2	NM_182688
A_23_P91850	IL20RB	NM_144717
A_23_P14938	CGN	NM_020770
A_23_P15563	CD80	NM_005191
A_23_P38830	ZNF552	NM_024762
A_23_P20051	ZNF238	NM_006352
A_23_P25019	MTDH	NM_178812
A_32_P73482	ZNF732	NM_001137608
A_23_P41681	ZFP82	NM_133466
A_33_P33568	LCE1B	NM_178349
A_24_P49775	ZNF493	NM_001076678
A_23_P38815	PLA2G12B	NM_032562
A_23_P12350	TRIB1	NM_025195
A_23_P36950	PADI4	NM_012387
A_23_P13982	YAF2	NM_001190979
A_23_P36944	KGFLP1	NR_003674
A_24_P17127	LOR	NM_000427
A_23_P35055	C12orf57	NM_138425
A_32_P38069	NIPAL1	ENST00000295461
A_23_P31654	RPL8	NM_000973
A_33_P33160	INTS6	NM_012141
A_23_P31759	SEMA3A	NM_006080
A_33_P34176	KLK10	NM_002776
A_23_P82959	FOXH1	NM_003923
A_23_P21345	BTF3	NM_001037637
A_32_P15017	RICTOR	NM_152756
A_23_P36665	CRY1	NM_004075
A_24_P12340	ABLIM3	NM_014945
A_23_P17103	SLC6A14	NM_007231
A_24_P92685	SEC24D	AK000709
A_23_P12292	C1orf116	NM_023938
A_23_P79043	TMEM147	NM_032635
A_32_P36455	TYK2	NM_003331
A_23_P6041	DEFB126	NM_030931
A_23_P18443	PPARGC1A	NM_013261
A_24_P31205	NUS1	NM_138459

A_32_P27774	ZNF81	NM_007137
A_23_P50167	SLC39A6	NM_012319
A_32_P29784	TPM3	NM_001043352
A_23_P66563	C17orf71	NM_018149
A_23_P85082	RHOXF1	NM_139282
A_23_P42813	EFNB2	NM_004093
A_23_P16307	GCH1	NM_000161
A_23_P25989	SETD3	NM_032233
A_32_P21683	NWD1	NM_001007525
A_23_P16978	TFCP2	NM_005653
A_24_P31831	MED6	NM_005466
A_24_P59494	PARP11	NM_020367
A_23_P42342	FAM171B	NM_177454
A_24_P94470	TCF12	AK022018
A_23_P36945	SYS1	NM_033542
A_23_P21452	ZNF192	NM_006298
A_32_P50941	HIST1H1B	NM_005322
A_23_P29922	TLR3	NM_003265
A_23_P72044	NPLOC4	NM_017921
A_23_P38101	WBSCR27	NM_152559
A_33_P33498	RBM3	NM_006743
A_23_P31826	DLX5	NM_005221
A_23_P61927	DOCK1	NM_001380
A_23_P20129	CASZ1	NM_017766
A_23_P11929	ECSIT	NM_016581
A_23_P30621	FAM84A	NM_145175
A_23_P50306	KL	NM_004795
A_24_P14984	ABI1	NM_005470
A_23_P55318	FLOT2	NM_004475
A_23_P39177	MACF1	NM_033044
A_23_P16878	BCAP29	NM_018844
A_23_P20728	SNAPC4	NM_003086
A_23_P86489	LBX1	X90828
A_23_P27627	ASNA1	NM_004317
A_33_P32402	RP11-529I10.	NM_015448
A_23_P10051	TMEM170A	NM_145254
A_23_P41604	MAPK8IP3	AK024437
A_23_P28073	GPX4	NM_001039848
A_24_P40282	CACNA2D3	NM_018398
A_32_P47577	LIX1L	ENST00000369308
A_24_P11328	ZNF229	NM_014518
A_23_P20996	SMC6	NM_024624
A_33_P32930	NKAIN4	NM_152864
A_23_P4679	ERF	NM_006494
A_23_P33791	PPFIBP1	NM_177444
A_23_P10271	SIRPB1	NM_001135844
A_23_P21809	RBM26	NM_022118
A_23_P35675	CEBPG	NM_001806
A_33_P32955	KRTAP26-1	NM_203405

A_23_P14848	RLIM	NM_016120
A_23_P13414	ZBTB24	NM_014797
A_32_P20742	ZNF845	NM_138374
A_23_P76659	N4BP2L1	NM_052818
A_23_P60499	ZNF462	NM_021224
A_23_P73632	NR0B1	NM_000475
A_24_P27427	STAT1	NM_139266
A_23_P25098	ISOC1	NM_016048
A_24_P99046	STK38L	NM_015000
A_23_P33600	RNF207	NM_207396
A_23_P17035	MRPL12	NM_002949
A_23_P91487	RWDD2B	NM_016940
A_33_P34001	TLE6	NM_001143986
A_33_P33891	MINPP1	NM_001178117
A_24_P31763	ZKSCAN5	NM_014569
A_32_P21962	EYA4	NM_004100
A_24_P24962	CDC42BPG	NM_017525
A_23_P76090	STAT2	NM_005419
A_23_P33503	ZNF721	NM_133474
A_24_P32069	IGFBP3	NM_001013398
A_23_P66593	GFAP	NM_002055
A_23_P15190	PCSK6	NM_002570
A_32_P18470	TCEAL5	NM_001012979
A_23_P70867	SBDS	NM_016038
A_23_P40810	MTERF	NM_006980
A_23_P11533	AHDC1	NM_001029882
A_24_P13278	RAB18	NM_021252
A_24_P11624	KLHDC2	NM_014315
A_23_P13116	GTDC1	AF090899
A_23_P39852	PKMYT1	NM_182687
A_32_P42776	ZNF836	NM_001102657
A_23_P25945	LSM6	NM_007080
A_23_P38094	ZNF420	NM_144689
A_33_P32838	SLC39A8	NM_001135147
A_23_P30468	EMP2	NM_001424
A_23_P13546	HARBI1	NM_173811
A_24_P18294	GCET2	NM_001008756
A_32_P22301	MAP3K6	NM_004672
A_23_P16784	KCNQ5	NM_019842
A_23_P13201	ITCH	NM_031483
A_23_P48897	CCPG1	AF011794
A_23_P10087	HOXB3	NM_002146
A_23_P25206	PPARG	NM_138711
A_23_P11810	NDUFB10	NM_004548
A_32_P12056	MDFIC	NM_199072
A_23_P51565	TNNI1	NM_003281
A_32_P1173	C6orf150	NM_138441
A_23_P52176	ZNF124	NM_003431
A_23_P15429	RAB6C	AK055504

A_32_P11620(	RELL1	NM_001085400
A_23_P74042	LPHN2	NM_012302
A_24_P94248:	GPR180	NM_180989
A_23_P91212	CHD6	NM_032221
A_24_P94445{	INSIG2	NM_016133
A_32_P76630	KRT14	NM_000526
A_23_P11973!	ZFP30	NM_014898
A_23_P50274(	RASAL2	NM_170692
A_23_P68121	PSD4	NM_012455
A_24_P18127!	SUCLA2	NM_003850
A_23_P1331	COL13A1	NM_080801
A_23_P43138:	C14orf80	NM_001134875
A_23_P13546:	HARBI1	NM_173811
A_23_P86587	NKX6-2	NM_177400
A_23_P36947:	LCE3A	NM_178431
A_23_P47857	TM7SF3	NM_016551
A_32_P13598!	TDGF1	NM_003212
A_32_P77217:	FAM43B	NM_207334
A_23_P31593	BUD31	NM_003910
A_23_P31532	ZC3HAV1L	NM_080660
A_23_P61945	MITF	NM_198159
A_23_P5903	SLCO4A1	NM_016354
A_32_P48784	ARHGAP21	NM_020824
A_23_P77076	SPPL2A	NM_032802
A_23_P82762	ZNF596	NM_001042416
A_23_P20847:	ELAVL1	NM_001419
A_32_P18251:	KIAA1609	NM_020947
A_23_P10938:	GSC2	NM_005315
A_33_P33816{	TCHH	NM_007113
A_33_P32292{	ACE	NM_000789
A_23_P5070	CADM4	NM_145296
A_33_P33857!	ZNF793	NM_001013659
A_33_P32189:	ENTPD1	NM_001776
A_23_P62620	CLCA2	NM_006536
A_24_P27922(	ATP6V0C	NM_001694
A_23_P4628	ZNF606	NM_025027
A_23_P21039!	CHRNA4	NM_000744
A_24_P40978	CYHR1	NM_138496
A_24_P24339(	TCOF1	NM_001008657
A_23_P1519	PITPNM1	NM_004910
A_23_P35106!	SOCS3	NM_003955
A_24_P37139!	C3orf58	NM_173552
A_32_P46191	ZNF727	NM_001159522
A_23_P10405⁄	C1orf9	NM_016227
A_32_P36684!	SUDS3	NM_022491
A_23_P4208	ZNHIT3	NM_004773
A_23_P34953	C1orf107	NM_014388
A_23_P39543{	HTRA3	NM_053044
A_32_P38623	PPP1R9A	NM_017650

A_23_P250002	HACE1	NM_020771
A_23_P153311	TYK2	NM_003331
A_23_P385529	POLR3E	AB040885
A_23_P7596	MYO10	NM_012334
A_23_P218040	KRT5	NM_000424
A_24_P401990	ERCC2	NM_000400
A_24_P348118	LGALS7B	NM_001042507
A_23_P152297	POLR3K	NM_016310
A_32_P59549	GFRA1	NM_145793
A_32_P200165	ELAVL1	NM_001419
A_23_P28105	TSN	NM_004622
A_33_P329557	TCEAL8	NM_153333
A_23_P142325	FZR1	NM_001136198
A_32_P48244	ZNF100	BC035579
A_24_P936145	GNAO1	NM_138736
A_23_P135826	ZNF93	NM_031218
A_33_P321227	F2R	BG026194
A_23_P38689	GNAL	NM_002071
A_23_P204423	PPP1CC	NM_002710
A_23_P102351	CHST10	NM_004854
A_33_P331126	KRTAP19-2	NM_181608
A_23_P203564	ZNF143	NM_003442
A_23_P435183	LRRFIP1	NM_001137550
A_24_P16417	SDK1	NM_152744
A_24_P42316	BDP1	NM_018429
A_23_P381594	CASKIN2	NM_020753
A_24_P55719	MED22	NM_133640
A_23_P138805	CHORDC1	NM_012124
A_32_P448547	CC2D1B	NM_032449
A_23_P170570	SNAI3	NM_178310
A_23_P52888	MPPED2	NM_001584
A_23_P35791	RPS6KA4	NM_003942
A_33_P339353	PTAFR	NM_001164722
A_23_P43964	LHPP	NM_022126
A_23_P30024	NFKB1	NM_003998
A_23_P145865	ADAP1	NM_006869
A_23_P160704	C1orf103	NM_018372
A_23_P97865	LIPA	NM_000235
A_23_P391264	BTN3A2	NM_007047
A_23_P49230	PRDM7	NM_052996
A_23_P156596	BTN3A3	NM_006994
A_33_P337751	HOXA6	NM_024014
A_23_P329152	ILF3	NM_012218
A_23_P346357	ZFP41	NM_173832
A_24_P220454	CUX1	NM_181552
A_23_P422204	PPPDE1	NM_016076
A_33_P326656	KRTAP5-6	NM_001012416
A_24_P35109	CLDN3	NM_001306
A_23_P74115	RAD54L	NM_003579

A_33_P322135	KIAA0564	NM_001009814
A_32_P433170	IP6K1	NM_153273
A_23_P205046	ANKRD10	NM_017664
A_23_P309711	TMX1	NM_030755
A_24_P482017	C7orf44	BC001743
A_24_P800604	FAM83H	NM_198488
A_23_P168490	HERPUD2	NM_022373
A_23_P210081	OSGEPL1	NM_022353
A_32_P182156	ZNF511	NM_145806
A_23_P67432	ZNF583	NM_152478
A_23_P88209	SCFD1	NM_016106
A_24_P912799	SEMA5A	NM_003966
A_23_P46470	ERRFI1	NM_018948
A_23_P93584	PRDM1	NM_001198
A_23_P135748	TM4SF1	NM_014220
A_32_P59486	SDHD	NM_003002
A_24_P49517	ZNF251	NM_138367
A_23_P419239	ETNK1	NM_018638
A_32_P69520	SLC39A11	NM_139177
A_23_P24870	CD44	NM_000610
A_23_P41166	B3GALNT1	NM_001038628
A_33_P338302	MXI1	NM_130439
A_23_P111155	ZBTB12	NM_181842
A_24_P212129	ZNF33A	NM_006974
A_23_P92342	NKX6-1	NM_006168
A_23_P115885	MINPP1	NM_004897
A_23_P62583	MEGF6	NM_001409
A_24_P261125	TMEM2	NM_013390
A_23_P11884	ALDH9A1	NM_000696
A_23_P126448	APOA1BP	NM_144772
A_23_P71661	TAL2	NM_005421
A_23_P394154	PDE8A	NM_002605
A_23_P116557	LGALS9	NM_009587
A_32_P419552	IER3	NM_003897
A_23_P343250	ZNF554	NM_001102651
A_33_P330725	AK5	NM_174858
A_23_P104145	ZMYM4	NM_005095
A_32_P526472	FLRT2	NM_013231
A_23_P129144	GCOM1	NM_001018100
A_24_P207828	LCE2B	NM_014357
A_24_P219808	PTOV1	NM_017432
A_32_P204518	WDR60	NM_018051
A_32_P366879	SETD1B	NM_015048
A_23_P166196	C20orf4	NM_015511
A_23_P111020	SRF	NM_003131
A_24_P48014	SOCS1	NM_003745
A_23_P126349	BARHL2	NM_020063
A_23_P104493	PAPSS2	NM_001015880
A_23_P363344	TPM1	NM_000366

A_23_P48747	DHRS1	NM_138452
A_32_P22943[illegible]	DKFZP586I14[illegible]	NR_002186
A_32_P19391[illegible]	CYP4V2	NM_207352
A_23_P89931	ZNF574	NM_022752
A_33_P33602[illegible]	HIST1H2AI	NM_003509
A_23_P47340	DSCAML1	NM_020693
A_24_P41918	C14orf135	ENST00000404681
A_23_P1431	BMPR1A	NM_004329
A_23_P40709[illegible]	ZNF366	NM_152625
A_23_P18267	TUSC4	NM_006545
A_23_P11714[illegible]	PRDM4	NM_012406
A_23_P16476	KLF16	NM_031918
A_33_P34000[illegible]	TCEANC	NM_152634
A_23_P40354	MAPRE1	NM_012325
A_23_P21468[illegible]	PPARD	NM_006238
A_24_P78788[illegible]	TMEM191A	NR_026815
A_23_P79231	CREB1	NM_134442
A_23_P20541[illegible]	BRMS1L	NM_032352
A_23_P33433	MAZ	NM_001042539
A_23_P75839	TSG101	NM_006292
A_23_P30345[illegible]	GPR161	NM_153832
A_23_P43447[illegible]	STT3B	NM_178862
A_24_P30162[illegible]	ZNF155	NM_003445
A_23_P10283[illegible]	CEP250	NM_007186
A_23_P15110[illegible]	PHB2	NM_007273
A_23_P37484[illegible]	GAL	NM_015973
A_23_P8801	CYP3A5	NM_000777
A_23_P14763[illegible]	TCEA2	NM_003195
A_23_P30675[illegible]	CRYAA	NM_000394
A_23_P40129	SAMD10	NM_080621
A_23_P14539[illegible]	CCNC	NM_005190
A_23_P12178[illegible]	SRD5A3	NM_024592
A_23_P38410[illegible]	LOC10012800[illegible]	AK093927
A_23_P20469[illegible]	CDKN1B	NM_004064
A_23_P10952[illegible]	CARD10	NM_014550
A_24_P24751[illegible]	ZNF283	NM_181845
A_24_P31973[illegible]	MEIS1	NM_002398
A_32_P11746[illegible]	C3orf59	NM_178496
A_32_P49624[illegible]	SPNS3	CR606228
A_32_P11311[illegible]	ZNF561	NM_152289
A_32_P87417	IFI16	NM_005531
A_23_P37623	GOLGA8A	NM_181077
A_23_P38817[illegible]	SLC39A3	NM_213568
A_24_P27693[illegible]	ATP6V1C2	NM_001039362
A_24_P12319[illegible]	PLD1	NM_002662
A_23_P41197[illegible]	C10orf104	NM_173473
A_32_P58937	MBTD1	NM_017643
A_23_P1981	INS	NM_000207
A_23_P19663	CTGF	NM_001901

A_24_P15130!	KLF2	NM_016270
A_23_P78265	KRT33A	NM_004138
A_33_P33743!	UNC45A	NM_001039675
A_23_P16450	YES1	NM_005433
A_23_P77847	GRB2	NM_002086
A_23_P12967	SETD1A	NM_014712
A_23_P10125	ZNF614	NM_025040
A_23_P13125!	SPATS2L	NM_015535
A_32_P34914!	SLC4A4	NM_003759
A_24_P25895!	ZNF317	NM_020933
A_23_P34988	PDCL	NM_005388
A_23_P15910	SNRNP35	NM_022717
A_24_P94429	FZR1	ENST00000395095
A_23_P59877	FABP5	NM_001444
A_23_P15145!	MYCBP2	NM_015057
A_24_P34633!	SESN1	NM_014454
A_23_P34001!	NLRC3	NM_178844
A_23_P15044(	EIF3M	NM_006360
A_23_P10086{	MYO19	NM_001033580
A_24_P86537	HSPB1	NM_001540
A_23_P21502	LRRC1	NM_018214
A_24_P32151	GOLT1B	NM_016072
A_33_P33755	MIER3	NM_152622
A_33_P32760!	LIMD1	XM_001717607
A_23_P39665	SLC11A1	NM_000578
A_32_P20019	FAM40B	NM_020704
A_23_P11413	SSX3	NM_175711
A_23_P25240	COMMD10	NM_016144
A_23_P89708	PNPO	NM_018129
A_23_P50156	VPS4B	NM_004869
A_23_P4678	ERF	NM_006494
A_23_P20821(	ZNF432	NM_014650
A_23_P14134(	MPP3	NM_001932
A_23_P31476(	PRKAG2	NM_016203
A_23_P46017	CCBL2	NM_001008661
A_23_P42570	MED1	NM_004774
A_23_P42389	SLC25A16	NM_152707
A_24_P85317	CHD2	NM_001042572
A_23_P45851	HIAT1	NM_033055
A_32_P12275	C9orf30	NM_080655
A_23_P11493	MAEL	NM_032858
A_23_P20195	ARID4B	NM_016374
A_23_P37244	SNAPC1	NM_003082
A_32_P16453	FOXE3	NM_012186
A_23_P38194!	KRT7	NM_005556
A_32_P25156	STT3B	ENST00000295770
A_23_P10232	BANK1	NM_017935
A_24_P34100(	ENST0000031	ENST00000318245
A_23_P13048	ZNF211	NM_198855

A_23_P32983!	UTY	NM_007125
A_24_P11637{	GCOM1	NM_001018090
A_23_P12701:	TCF7L2	NM_030756
A_24_P81841	CDKN1B	NM_004064
A_32_P20669{	CKS1B	NM_001826
A_32_P55804	CDC5L	ENST00000371477
A_23_P25647:	SEMA3C	NM_006379
A_23_P20228(	VCL	NM_014000
A_24_P27537:	PYROXD1	NM_024854
A_23_P12341:	TOX	NM_014729
A_23_P12535:	CD99L2	NM_031462
A_23_P21463{	EHMT2	NM_006709
A_23_P35787	RPS6KA4	NM_003942
A_24_P25184:	SETD7	NM_030648
A_24_P45160	C17orf90	NM_001039842
A_23_P35597	C10orf10	NM_007021
A_23_P40272	GNRH2	NM_001501
A_33_P32705(	SIAH1	NM_003031
A_23_P15001{	DUSP5	NM_004419
A_33_P34191!	AREG	NM_001657
A_24_P25639(	RIN3	AK090451
A_24_P20497:	HBP1	NM_012257
A_23_P9756	PUF60	NM_014281
A_23_P13112{	ZNF569	NM_152484
A_23_P43016!	EXOC8	NM_175876
A_23_P12686!	PADI3	NM_016233
A_23_P15161(	PSME1	NM_006263
A_23_P43201:	ZPLD1	NM_175056
A_32_P39041!	ZPLD1	NM_175056
A_33_P33864!	ARHGAP5	NM_001030055
A_24_P24782(	ELF5	AK074633
A_23_P59876	FABP5	NM_001444
A_32_P33433	ZNF812	NM_001199814
A_24_P80810(	MCTS1	NM_014060
A_24_P13494:	VHL	NM_000551
A_23_P22350	GRAMD3	NM_023927
A_23_P14298(	EGR4	NM_001965
A_24_P63522	HMGCS1	NM_002130
A_33_P33141(	YAF2	NM_001190979
A_23_P54953	SAP30BP	NM_013260
A_23_P25939:	SFMBT1	NM_001005159
A_33_P32713!	IKBKAP	NM_003640
A_23_P50279:	WDFY1	NM_020830
A_23_P65890	CIB2	NM_006383
A_23_P66900	CDK5RAP3	NM_176096
A_24_P10516·	MAGT1	NM_032121
A_23_P10883!	YPEL5	NM_016061
A_32_P46177(	DYNC2H1	NM_001377
A_33_P32594(	PAN3	NM_175854

A_23_P48717	NPC2	NM_006432
A_23_P61171	KCNG2	NM_012283
A_23_P84259	COX5A	NM_004255
A_32_P20629	ZNF322B	NM_199005
A_23_P20658	PRKCB	NM_002738
A_23_P42575	ARMC6	NM_033415
A_33_P32687	SNX1	NM_003099
A_33_P33627	POM121L10P	NR_024593
A_23_P32872	KLHL8	NM_020803
A_23_P14252	COL3A1	NM_000090
A_23_P62335	TMLHE	NM_018196
A_23_P12204	PPP2CA	NM_002715
A_24_P21910	RPS6KL1	NM_031464
A_32_P30399	ZNF702P	NR_003578
A_23_P10420	YME1L1	NM_139312
A_24_P34430	PSME3	NM_176863
A_23_P16425	PIPOX	NM_016518
A_23_P16866	CROT	NM_021151
A_23_P14586	S100A11	NM_005620
A_23_P36921	CLCN5	ENST00000376108
A_23_P35600	KCNIP3	NM_013434
A_23_P12663	MAP3K6	NM_004672
A_23_P25253	MRPS26	NM_030811
A_32_P84982	FOXI3	NM_001135649
A_33_P33061	PLAU	NM_001145031
A_24_P98210	TFEC	NM_012252
A_24_P94216	FAM63A	AB037811
A_24_P16761	INTS6	NM_012141
A_23_P36825	GPRC5A	NM_003979
A_24_P26952	SLURP1	NM_020427
A_33_P33769	CHAC1	NM_024111
A_32_P17348	IK	NM_006083
A_23_P2083	ASCL3	NM_020646
A_24_P64653	METTL7B	NM_152637
A_32_P51080	STT3A	NM_152713
A_23_P11854	CLTC	NM_004859
A_32_P12482	OXCT2	NM_022120
A_24_P17066	SPEN	NM_015001
A_33_P32898	CDX1	NM_001804
A_24_P32847	PLEKHA3	NM_019091
A_32_P41071	OBSCN	NM_052843
A_23_P60047	TACC1	NM_006283
A_23_P10885	ZNF224	AK292500
A_23_P31116	ACOT13	NM_018473
A_23_P14251	NACC1	NM_052876
A_23_P11178	FAM115A	NM_014719
A_23_P26413	RBL2	NM_005611
A_23_P37551	ZNF681	NM_138286
A_23_P20226	ANK3	NM_020987

A_23_P41232	CCR5	NM_000579
A_23_P16787	MEMO1	NM_015955
A_33_P33966	KDM4B	NM_015015
A_23_P993	NR5A2	NM_205860
A_33_P32407	CNKSR2	NM_014927
A_23_P5389	DPY30	NM_032574
A_23_P21878	DDX17	NM_006386
A_23_P15857	ZNF271	NR_024565
A_33_P33552	CROT	NR_026585
A_33_P32394	GTF2IRD2	NM_173537
A_33_P33342	TMEM184A	NM_001097620
A_23_P12109	TOP2B	NM_001068
A_32_P59712	SRXN1	NM_080725
A_23_P83278	CHMP5	NM_016410
A_23_P11879	TMEM107	NM_032354
A_23_P25960	RNF144B	NM_182757
A_33_P33335	SENP7	NM_020654
A_23_P13180	SGK2	NM_170693
A_32_P84778	ZNF765	NM_001040185
A_24_P65722	LILRB4	ENST00000391736
A_24_P18337	PPP2CA	NM_002715
A_23_P36007	NCKAP5	NM_207363
A_23_P38300	IGFBP5	NM_000599
A_23_P20690	NDE1	NM_017668
A_33_P33361	TIGD5	NM_032862
A_23_P92794	C5orf13	NM_004772
A_23_P9340	BARHL1	NM_020064
A_32_P61197	SRFBP1	NM_152546
A_23_P25081	WRN	NM_000553
A_23_P38032	SLC39A9	NM_018375
A_24_P29570	SUV420H1	ENST00000304363
A_33_P33152	KRT78	NM_173352
A_23_P86002	TMED5	NM_016040
A_23_P40171	CCDC74B	NM_207310
A_33_P34187	GNRH2	ENST00000380347
A_32_P45696	ZNF280C	NM_017666
A_23_P67299	DOCK6	NM_020812
A_33_P32234	FRY	NM_023037
A_23_P50735	ZNF181	NM_001029997
A_23_P31577	KCND1	NM_004979
A_23_P21405	NR3C1	NM_001018077
A_33_P32148	PRDM13	NM_021620
A_23_P32972	MAPK8IP3	NM_015133
A_24_P24874	ZNF501	NM_145044
A_24_P20777	RPL37	NM_000997
A_33_P33222	TNFRSF1B	NM_001066
A_23_P24311	ALDH3B2	NM_000695
A_23_P16799	HIST1H2BG	NM_003518
A_32_P22754	SLC33A1	NM_004733

A_33_P37392(	CAP2	NM_006366
A_33_P33175(	GTF2IRD2	NM_173537
A_32_P99311	ZNF233	NM_181756
A_23_P12669(	NR1I3	NM_001077482
A_23_P10367(	NES	NM_006617
A_23_P21655(	EPB41L4B	NM_018424
A_32_P34622(	OAZ1	NM_004152
A_23_P64121	C11orf41	NM_012194
A_23_P82669	GBAS	NM_001483
A_23_P91114	PREB	NM_013388
A_23_P35365(	MEF2A	NM_005587
A_24_P93668(	SNRK	NM_017719
A_23_P55551	CHMP1B	NM_020412
A_23_P66081	N4BP1	NM_153029
A_24_P19884	ZBTB7B	NM_015872
A_24_P24334(	MAT2B	NM_182796
A_23_P14136(	CCR10	NM_016602
A_32_P16917(	MSX2	NM_002449
A_23_P20346(	TAF10	NM_006284
A_23_P13102(	ZBTB32	NM_014383
A_23_P66599	VPS25	NM_032353
A_23_P70480	HIST1H4L	NM_003546
A_23_P38271(	LPIN1	NM_145693
A_24_P11208(	ASF1A	NM_014034
A_23_P43690	BNC2	NM_017637
A_23_P11779(	CLN6	NM_017882
A_23_P51355	KIAA2013	ENST00000376572
A_23_P86424	NCOA4	NM_005437
A_33_P33185(	ZNF345	NM_003419
A_23_P98232	ZFPL1	NM_006782
A_23_P21559(	SRPK2	NM_182691
A_24_P53519	CHAF1A	NM_005483
A_32_P46852(	NCR3	NM_001145467
A_23_P14245(	SIRT2	NM_012237
A_23_P16800(	HIST1H2AI	NM_003509
A_23_P25305(	CD99L2	NM_031462
A_23_P16295(	EGLN3	NM_022073
A_23_P32393(	TSPAN5	NM_005723
A_23_P5550	PUM2	NM_015317
A_32_P13194(	POM121	NM_172020
A_23_P10666(	CMTM1	NM_052999
A_24_P41418(	IQSEC1	NM_014869
A_23_P16827(	TBPL1	NM_004865
A_24_P85275(	HLA-DQA2	NM_020056
A_23_P94204	OXR1	NM_181354
A_23_P21032(	CEP68	NM_015147
A_33_P36748(	VPS36	NM_016075
A_33_P38416(	MYO5B	NM_001080467
A_23_P34227(	ADAMTS1	NM_006988

A_32_P16056!	OPCML	NM_001012393
A_32_P75689{	ARL4A	BC001111
A_23_P37359{	MAFK	NM_002360
A_23_P21904!	HIST1H3D	NM_003530
A_23_P13011(	ASGR2	NM_080912
A_23_P15232;	COQ7	NM_016138
A_23_P30268:	FIGNL1	NM_001042762
A_32_P23161;	TM4SF1	NM_014220
A_24_P11770!	MAML3	NM_018717
A_33_P32240;	CSRNP1	NM_033027
A_24_P21082!	NME4	NM_005009
A_23_P68040	PCBP1	NM_006196
A_24_P15239{	TP53AIP1	NM_001195195
A_23_P27215	UBB	NM_018955
A_23_P12361(	JAK2	NM_004972
A_24_P7121	NSUN7	NM_024677
A_23_P25039;	NKX2-5	NM_001166175
A_23_P43726	NUP160	NM_015231
A_23_P25434(	RPN1	NM_002950
A_32_P32948{	ZNF251	NM_138367
A_23_P13540;	COBRA1	NM_015456
A_33_P34046!	PSTK	NM_153336
A_24_P33051{	CA12	NM_001218
A_24_P26272{	SH2D3A	NM_005490
A_24_P93850	LOC404266	NR_033201
A_32_P18824	BRD7	NM_013263
A_23_P43198:	HMGXB4	NM_001003681
A_33_P33306(	PRAM1	NM_032152
A_23_P21721{	KCND1	NM_004979
A_23_P14650(	GOLM1	NM_016548
A_23_P83736	ING2	NM_001564
A_24_P30007(	SEMA3F	NM_004186
A_23_P25795(	ILF2	NM_004515
A_33_P33304	FAM110B	NM_147189
A_24_P14964!	PUF60	NM_014281
A_23_P12563!	ZFX	NM_003410
A_23_P54019	ZBTB42	NM_001137601
A_24_P1731	MEF2A	NM_005587
A_23_P71328	MATN2	NM_030583
A_32_P18238{	ZNF77	NM_021217
A_24_P24092	ZNF181	NM_001029997
A_32_P72300:	AMAC1L2	NM_054028
A_24_P42803	WDR26	NM_025160
A_32_P45960;	TCF20	NM_181492
A_24_P38662;	ARRB1	NM_004041
A_24_P23455	ZNF385D	ENST00000281523
A_23_P15599;	PCDH12	ENST00000231484
A_23_P21776:	TMSB4X	ENST00000380635
A_23_P10978!	NR1I2	NM_003889

A_24_P17958!	MARK1	NM_018650
A_33_P33068·	ZNF836	NM_001102657
A_24_P71439	PUM2	NM_015317
A_23_P50123:	KCNAB2	NM_003636
A_23_P17123:	AMOT	ENST00000304758
A_23_P21436:	IRF4	NM_002460
A_23_P14140!	NME2	NM_002512
A_32_P20964:	KIAA0232	NM_014743
A_23_P32318(	HOXD3	NM_006898
A_23_P33958:	CXorf38	NM_144970
A_33_P33811·	KRTAP5-3	NM_001012708
A_33_P32120!	PDCD4	NM_145341
A_33_P32660:	TAF6L	NM_006473
A_33_P32349:	IFT81	NM_031473
A_23_P14180:	SERPINB7	NM_001040147
A_23_P51307	LELP1	NM_001010857
A_23_P39304	PSG9	NM_002784
A_23_P11084:	CNOT8	NM_004779
A_33_P32869!	ADAMTS6	NM_197941
A_24_P11635:	SIVA1	NM_006427
A_23_P9640	CNIH3	NM_152495
A_33_P33986(	ZSCAN30	NM_001166012
A_23_P11633	PRKAB2	NM_005399
A_24_P22981	ZNF253	NM_021047
A_33_P33653(	CHRNA1	NM_001039523
A_24_P58857	ZNF813	NM_001004301
A_23_P37649:	IGFBP5	NM_000599
A_23_P25205:	FILIP1L	NM_182909
A_23_P14764:	TCEA2	NM_003195
A_23_P17023:	CSTA	NM_005213
A_24_P10388(	IDI1	NM_004508
A_32_P82863	ZNF829	NM_001037232
A_24_P35660:	HEXIM1	NM_006460
A_23_P18304	UBA5	NM_198329
A_33_P32979:	COL8A2	NM_005202
A_23_P14031(	GPR137C	NM_001099652
A_23_P42800	PDIA4	NM_004911
A_23_P14797(	BRDT	AF019085
A_23_P79978	SLC24A3	NM_020689
A_23_P27450	APOC4	NM_001646
A_24_P29142(	REV1	NM_016316
A_23_P21887!	TREX1	NM_016381
A_24_P33366:	MAPK6	NM_002748
A_32_P83049	EFR3B	NM_014971
A_24_P40529	TMLHE	NM_018196
A_23_P47541	TAF6L	NM_006473
A_23_P16691(	DNAJC13	NM_015268
A_24_P11239!	PBLD	NM_022129
A_23_P21349!	ERAP1	NM_016442

A_23_P10553!	WDR51B	NM_172240
A_24_P20904:	IL5	NM_000879
A_24_P36067	CDKN2B	NM_078487
A_23_P93258	HIST1H3B	NM_003537
A_23_P50042	EYA2	NM_005244
A_23_P7976	HIST1H1E	NM_005321
A_23_P17480	SIGLEC1	NM_023068
A_24_P38561	SP100	NM_003113
A_23_P60565	ZNF354A	NM_005649
A_23_P32255(	REC8	NM_001048205
A_24_P14912	C5orf13	NM_004772
A_32_P46526	LSM11	NM_173491
A_23_P16521	ZNF253	NM_021047
A_24_P19497	ZNF492	NM_020855
A_33_P32214	KIRREL	NM_018240
A_23_P13902	POU2F3	NM_014352
A_23_P36877	ZNF560	NM_152476
A_23_P14395	CEP97	NM_024548
A_23_P11062	CTNND2	NM_001332
A_32_P30729	ZNF479	NM_033273
A_24_P21008	C11orf71	NM_019021
A_23_P3371	RASGRF1	NM_002891
A_32_P19151!	HTR2C	NM_000868
A_24_P36007	LRBA	NM_006726
A_33_P34239	CBX2	NM_005189
A_23_P32499	KLHL7	NM_018846
A_23_P71268	AZGP1	NM_001185
A_24_P10556	PRKAB2	NM_005399
A_23_P11740	MIXL1	NM_031944
A_23_P12869	SPRY2	NM_005842
A_24_P39790	FAM164A	NM_016010
A_23_P10670	RPS2	NM_002952
A_33_P33580(	LOC440934	XR_108436
A_23_P11864	EIF4E	NM_001968
A_23_P20920(	CCNE1	NM_001238
A_24_P12649	ZNF99	NM_001080409
A_23_P25760	RPL29	NM_000992
A_23_P17456	SIRPB1	NM_006065
A_33_P32200	ZNF341	NM_032819
A_23_P50030(	TRIM15	NM_033229
A_23_P25715!	ATXN7	NM_000333
A_23_P56555	METTL8	NM_024770
A_23_P13872	MLF2	NM_005439
A_32_P18734(	FNTA	NM_002027
A_23_P87603	TARBP2	NM_134323
A_23_P25922(	EPHB3	NM_004443
A_32_P43978	ZNF846	NM_001077624
A_23_P42934(	TEAD1	NM_021961
A_32_P18390(	SHF	NM_138356

A_23_P14317:	SLA2	NM_032214
A_23_P21686!	ZNF189	NM_197977
A_32_P52122:	NLGN1	ENST00000457714
A_23_P13044	ZNF701	NM_018260
A_23_P57379	CDC45L	NM_003504
A_24_P12920!	FBXL4	NM_012160
A_23_P46455	DNAJC8	NM_014280
A_33_P32223!	FILIP1L	NM_182909
A_23_P76109	RILPL2	NM_145058
A_23_P14989:	CSGALNACT2	NM_018590
A_23_P13050!	ZNF229	NM_014518
A_24_P27498:	TMEFF1	NM_003692
A_23_P79652	HOXD3	NM_006898
A_23_P75247	OPALIN	NM_001040103
A_32_P21246	HCG27	NR_026791
A_23_P43358(	HTR2C	NM_000868
A_23_P67702	ZNF85	NM_003429
A_23_P14874{	PLEKHA6	NM_014935
A_23_P16741!	CNOT6	NM_015455
A_24_P10411!	RHOF	NM_019034
A_23_P50182:	JUP	NM_002230
A_23_P34600(	CCPG1	NM_020739
A_32_P19748!	KLF13	NM_015995
A_24_P14746:	SERPINB8	NM_001031848
A_23_P5654	IL1F7	NM_014439
A_23_P48088	CD27	NM_001242
A_32_P60133	TRIM37	NM_015294
A_23_P50179!	MED22	NM_133640
A_32_P38918:	MLXIPL	AF056184
A_23_P57676	HLTF	NM_003071
A_33_P33248{	MICAL1	NM_022765
A_23_P17197	RAPGEF4	NM_007023
A_23_P56390	WDFY1	NM_020830
A_32_P40449(	MESP2	NM_001039958
A_32_P49077:	SLC10A4	NM_152679
A_33_P33643:	KRTAP5-2	NM_001004325
A_23_P13244	TCEA1	NM_006756
A_33_P32456(	BHLHA9	NM_001164405
A_23_P36689!	B3GNT6	NM_138706
A_33_P32615(	KIAA1632	NM_020964
A_23_P16841!	MLL3	NM_170606
A_24_P13230	RAB6A	NM_002869
A_24_P16640:	HIST1H4B	NM_003544
A_23_P84320	HMX1	NM_018942
A_23_P21409:	LYPLA1	NM_006330
A_23_P42626	MEPCE	NM_019606
A_23_P38574	IGFBP4	NM_001552
A_23_P48535	ARHGAP5	NM_001030055
A_23_P22205	SLC4A4	NM_003759

A_24_P13430[illegible]	SPINLW1	NM_020398
A_24_P13663	TCF12	ENST00000267811
A_24_P37309[illegible]	TGFB3	NM_003239
A_23_P75056	GATA3	NM_001002295
A_24_P26172[illegible]	RNF10	NM_014868
A_23_P14179[illegible]	SERPINB7	NM_001040147
A_33_P33237[illegible]	PNRC2	NM_017761
A_23_P11954[illegible]	HMG20B	NM_006339
A_24_P19509[illegible]	SOHLH1	NM_001012415
A_23_P14119[illegible]	ZNF750	NM_024702
A_33_P34238[illegible]	C20orf200	NR_033263
A_24_P10985[illegible]	ZNF468	NM_199132
A_23_P14226[illegible]	PAF1	NM_019088
A_24_P86288[illegible]	DRD5	NM_000798
A_23_P13291[illegible]	FAM114A1	NM_138389
A_23_P90296	DYRK1B	NM_004714
A_23_P17049[illegible]	TRAIP	NM_005879
A_23_P88411	SEL1L	NM_005065
A_23_P91217	CHD6	NM_032221
A_23_P13048[illegible]	ERCC2	NM_000400
A_33_P32379[illegible]	TCHHL1	NM_001008536
A_23_P20089[illegible]	GOLPH3L	NM_018178
A_23_P25312[illegible]	VGLL1	NM_016267
A_23_P70874	SBDS	NM_016038
A_24_P27260[illegible]	ZNF808	NM_001039886
A_23_P14742[illegible]	ADAMTS9	NM_182920
A_23_P14285[illegible]	AQP12A	NM_198998
A_23_P20275	PLEKHF2	NM_024613
A_23_P58898	CASP8AP2	NM_012115
A_32_P20302[illegible]	FAM9B	NM_205849
A_32_P79667[illegible]	S1PR3	NM_005226
A_33_P32233[illegible]	KRTAP8-1	NM_175857
A_23_P31993	SLC46A2	NM_033051
A_23_P42783	ZNF277	NM_021994
A_24_P50154[illegible]	YPEL2	NM_001005404
A_23_P15264[illegible]	SLC43A2	NM_152346
A_33_P33047[illegible]	TPP2	BC024905
A_23_P23584	CTNNBIP1	NM_020248
A_23_P22765	NDUFB11	NM_019056
A_23_P14799[illegible]	PICALM	NM_007166
A_23_P20542[illegible]	FOXG1	NM_005249
A_23_P55421	CBX8	NM_020649
A_33_P33239[illegible]	LOC150786	NM_001077637
A_23_P82065	ELOVL4	NM_022726
A_32_P13834[illegible]	LY6K	NM_017527
A_23_P39916[illegible]	HIC1	NM_006497
A_32_P17115[illegible]	PLEKHM3	NM_001080475
A_23_P31481[illegible]	SLC10A4	NM_152679
A_23_P25488[illegible]	ZYX	NM_003461

A_23_P10660	CRISPLD2	NM_031476
A_24_P42603	TRIO	NM_007118
A_33_P32395	ZNF3	NM_017715
A_23_P10884	DUSP2	NM_004418
A_23_P50517	ZNF541	NM_001101419
A_33_P33112	KRTAP19-7	NM_181614
A_24_P13737	ATP2C1	NM_001001487
A_23_P25822	MICALL2	NM_182924
A_23_P35494	ZNF493	NM_001076678
A_32_P13555	TMEM188	NM_153261
A_33_P32348	PRELP	NM_002725
A_23_P15992	IKBKG	NM_003639
A_23_P25510	LHFPL2	NM_005779
A_23_P33418	MEF2D	NM_005920
A_33_P32849	VLDLR	NM_001018056
A_33_P33726	BHLHA9	NM_001164405
A_23_P14925	TMEM79	NM_032323
A_23_P16237	DDX54	NM_024072
A_23_P43085	ZNF33A	AK027057
A_23_P25194	DCTN4	NM_016221
A_32_P58190	PNRC2	NM_017761
A_23_P43126	PLEKHA6	NM_014935
A_23_P23960	BLOC1S2	NM_001001342
A_23_P70180	JMY	NM_152405
A_33_P34215	RAPH1	NM_213589
A_24_P20297	MTMR3	NM_021090
A_24_P32639	CRB2	NM_173689
A_24_P12467	DYNLL1	NM_001037494
A_23_P19389	HMGN4	NM_006353
A_23_P12963	IRF8	NM_002163
A_24_P18837	CD55	NM_000574
A_32_P19564	KLHL15	NM_030624
A_23_P36872	TFEB	NM_007162
A_24_P32501	TCFL5	BC065520
A_23_P12727	FAM35A	NM_019054
A_32_P17106	ASCL2	NM_005170
A_23_P10768	ZNF324	NM_014347
A_23_P20826	RPS5	NM_001009
A_23_P53257	AVIL	BX647344
A_24_P26866	ABHD10	NM_018394
A_23_P15791	MAMDC2	NM_153267
A_23_P29855	USO1	NM_003715
A_33_P33828	NYNRIN	NM_025081
A_23_P14815	COBRA1	NM_015456
A_23_P53276	TIMELESS	NM_003920
A_23_P12171	ANXA3	NM_005139
A_23_P15916	AATK	NM_001080395
A_23_P21442	NFYA	NM_002505
A_33_P32419	PLAC8	NM_001130715

A_24_P56194	CREBL2	NM_001310
A_23_P26223	ASL	NM_001024943
A_24_P27375[illegible]	TP63	NM_003722
A_24_P405205	ATP2B4	NM_001001396
A_32_P174908	WAPAL	NM_015045
A_32_P91250	UBE2L3	NM_003347
A_23_P368794	TCERG1L	NM_174937
A_24_P407259	SOX21	NM_007084
A_23_P127224	FRAT1	NM_005479
A_24_P183544	GPR110	ENST00000371253
A_23_P96008	MALT1	NM_006785
A_24_P352608	GALC	NM_000153
A_23_P409988	PHC3	NM_024947
A_24_P185205	TP53I13	NM_138349
A_23_P107714	MARK4	NM_031417
A_24_P157388	CDV3	NM_017548
A_23_P395075	KDM3A	NM_018433
A_23_P21832[illegible]	MNT	NM_020310
A_32_P37753[illegible]	DENND4C	NM_017925
A_23_P500844	PDE5A	NM_001083
A_23_P80389	TRMU	NM_018006
A_23_P43367[illegible]	ZNF529	NM_020951
A_23_P65174	PHF11	NM_001040443
A_23_P82775	SOX17	NM_022454
A_23_P121074	EOMES	NM_005442
A_24_P919279	ZNF790	NM_206894
A_23_P55549	CHMP1B	NM_020412
A_23_P108574	PRKD3	NM_005813
A_23_P14638[illegible]	AK3	NM_016282
A_24_P77904	HOXA10	NM_018951
A_23_P209689	ROCK2	NM_004850
A_24_P350622	KIR2DL4	NM_002255
A_24_P24678[illegible]	REST	NM_005612
A_23_P117759	GCOM1	NM_001018090
A_24_P319635	MCL1	NM_021960
A_23_P88163	ATG2B	NM_018036
A_23_P81850	HIST1H4B	NM_003544
A_24_P22140[illegible]	PYROXD1	NM_024854
A_23_P24984	TSPAN31	NM_005981
A_24_P941572	FAM114A1	NM_138389
A_24_P319354	SUMO1	NM_001005781
A_24_P73599	IL16	NM_172217
A_33_P338578	ZNF713	NM_182633
A_23_P134334	NEUROD6	NM_022728
A_24_P35022[illegible]	SHC4	NM_203349
A_33_P32357[illegible]	QSER1	NM_001076786
A_33_P330874	LAMA4	NM_001105207
A_33_P33443[illegible]	KCNH5	NM_172376
A_24_P343255	KLHDC3	NM_057161

A_24_P94458	ZNF682	NM_033196
A_23_P16453	PIK3C3	NM_002647
A_23_P11399	KIRREL	NM_018240
A_23_P40215	KCTD17	NM_024681
A_24_P22166	ZBTB41	NM_194314
A_33_P33666	EYA1	NM_000503
A_23_P28015	ZNF558	NM_144693
A_23_P74993	FLJ39739	AK096104
A_23_P62981	TBX15	NM_152380
A_23_P21692	NEK6	NM_014397
A_23_P11021	ACSL1	NM_001995
A_32_P53512	PHB2	NM_007273
A_24_P15691	HIST2H2BE	NM_003528
A_24_P28747	SAV1	NM_021818
A_23_P84219	LIPH	NM_139248
A_33_P33726	PDGFA	NM_033023
A_24_P17248	TRIM22	NM_006074
A_24_P9883	DKFZp761E19	NM_138368
A_23_P11318	FTO	NM_001080432
A_23_P36384	SH3PXD2B	NM_001017995
A_23_P12957	TIGD7	NM_033208
A_24_P92732	C2CD3	NM_015531
A_23_P21650	TPM2	NM_213674
A_23_P10982	TADA3	NM_006354
A_33_P32278	EPB41	NM_001166006
A_23_P14487	ATOX1	NM_004045
A_33_P33096	C11orf58	NM_014267
A_33_P32575	AGPHD1	NM_001083612
A_32_P37788	GDNF	NM_001190468
A_23_P86403	KIF5B	NM_004521
A_32_P22314	RASGEF1A	NM_145313
A_24_P40603	SLC35A1	NM_006416
A_23_P55518	SMAD7	NM_005904
A_23_P11757	ENTPD5	NM_001249
A_23_P13147	KCMF1	NM_020122
A_24_P28149	ACAP3	NM_030649
A_33_P37757	RPL32P3	NR_003111
A_23_P1641	RCE1	NM_005133
A_23_P20158	SORT1	NM_002959
A_24_P21462	PPA2	NM_176869
A_23_P80398	NCAPH2	NM_152299
A_23_P6651	C3orf63	NM_015224
A_23_P36174	ATXN3L	NM_001135995
A_23_P63997	CUGBP1	AB210019
A_23_P31035	SHPK	NM_013276
A_32_P5251	RARA	NM_001024809
A_24_P15114	ZNF235	BC002663
A_23_P34455	NEDD9	NM_006403
A_23_P10039	TCEB2	NM_007108

A_33_P33116[illegible]	SCFD1	NM_016106
A_23_P98455	VWA5A	NM_014622
A_23_P20382[illegible]	ZNF10	NM_015394
A_23_P20705[illegible]	SOCS3	NM_003955
A_24_P39748[illegible]	GCNT2	NM_001491
A_24_P35064[illegible]	PRRC2B	NM_013318
A_23_P69680	SEC24B	NM_006323
A_23_P25046[illegible]	ATP6AP1	NM_001183
A_23_P30438[illegible]	HIGD2A	NM_138820
A_23_P5318	LRPPRC	NM_133259
A_23_P10047[illegible]	MYST1	NM_032188
A_23_P39367[illegible]	SALL3	NM_171999
A_24_P32085	MOBKL2B	NM_024761
A_33_P38787[illegible]	JAK2	NM_004972
A_24_P26352[illegible]	TXNDC9	NM_005783
A_32_P34099[illegible]	SYT2	NM_001136504
A_23_P15747[illegible]	ATP6V1H	NM_015941
A_32_P45929[illegible]	B4GALNT2	NM_153446
A_33_P32937[illegible]	KRTAP10-1	NM_198691
A_23_P43177[illegible]	ETV4	NM_001079675
A_23_P16506[illegible]	AES	NM_198969
A_23_P30196[illegible]	SIGLEC1	NM_023068
A_33_P33553[illegible]	ZNF676	NM_001001411
A_33_P33238[illegible]	GATAD2B	NM_020699
A_23_P58721	RPL37	NM_000997
A_33_P33884[illegible]	TMEM120B	NM_001080825
A_24_P27068[illegible]	SLC38A7	NM_018231
A_23_P15097[illegible]	HOXC4	NM_014620
A_23_P20889[illegible]	CHAF1A	NM_005483
A_23_P20464[illegible]	NANOG	NM_024865
A_33_P32450[illegible]	DAK	NM_015533
A_23_P40916[illegible]	NBEAL2	NM_015175
A_33_P34029[illegible]	FEZF1	NM_001024613
A_32_P83254	IRAK3	NM_007199
A_33_P33426[illegible]	ADAM33	NM_153202
A_24_P85869[illegible]	NFIX	NM_002501
A_23_P15676[illegible]	BRPF3	NM_015695
A_24_P4678	ACAD8	NM_014384
A_23_P21748[illegible]	FOXO4	NM_005938
A_33_P32437[illegible]	ZNF248	NM_021045
A_33_P32091[illegible]	APOC4	NM_001646
A_23_P27767	ZNF444	NM_018337
A_23_P25322[illegible]	ARHGEF4	NM_032995
A_23_P41071[illegible]	C1orf51	NM_144697
A_23_P72568	SNX4	NM_003794
A_24_P19087[illegible]	C1orf25	NM_030934
A_32_P36793[illegible]	CEP120	NM_153223
A_33_P32891[illegible]	ZBTB32	NM_014383
A_24_P12455[illegible]	HOXC8	NM_022658

A_23_P15944	ELAC1	NM_018696
A_23_P102419	PLEKHA3	NM_019091
A_23_P9392	C9orf114	NM_016390
A_23_P117172	PARP4	NM_006437
A_23_P7191	LGI2	NM_018176
A_23_P65555	SAV1	NM_021818
A_23_P72657	OPCML	NM_001012393
A_23_P325093	GGT7	NM_178026
A_32_P151263	SLC2A4	NM_001042
A_24_P199905	YWHAQ	NM_006826
A_24_P222684	TROVE2	NM_001042369
A_23_P77430	PRMT7	NM_019023
A_24_P943815	PNPLA4	NM_004650
A_23_P164028	PSMC5	NM_002805
A_23_P62932	ATP1B1	NM_001677
A_23_P203267	TRIM29	NM_012101
A_23_P204164	ETNK1	NM_001039481
A_24_P228027	CYB5D2	NM_144611
A_23_P211840	UBA3	NM_003968
A_33_P335862	TAF4B	NM_005640
A_23_P133650	FAM8A1	NM_016255
A_23_P211762	COL8A1	ENST00000261037
A_23_P26124	RORA	NM_134260
A_23_P144188	SLC33A1	NM_004733
A_23_P356585	HLF	NM_002126
A_23_P166775	IL17RC	NM_153461
A_23_P126159	HPCA	NM_002143
A_23_P29638	NCKIPSD	NM_184231
A_33_P328161	KCNT1	AK123276
A_24_P102821	PTAFR	NM_000952
A_24_P133475	DHRS1	NM_138452
A_32_P191311	PTPN11	NM_002834
A_23_P9621	ELF4	NM_001421
A_24_P156757	MYCL1	NM_005376
A_33_P323514	DLX5	NM_005221
A_23_P23468	WNT3A	NM_033131
A_23_P165968	SEMG1	NM_003007
A_23_P170986	TMCC1	NM_001017395
A_23_P36831	GPRC5A	NM_003979
A_23_P398254	SMURF1	NM_020429
A_23_P78429	ZSCAN30	NM_001166012
A_23_P108157	TJP3	NM_014428
A_24_P208703	FAM107A	NM_007177
A_24_P937965	RAB3GAP1	BC030992
A_23_P94736	ST6GALNAC4	NM_175039
A_33_P325184	DSEL	NM_032160
A_32_P77098	TMEM200B	NM_001003682
A_23_P154130	MAP4K3	NM_003618
A_23_P67424	ZNF461	NM_153257

A_23_P32018	NDUFA11	NM_175614
A_23_P10761	RAB27B	NM_004163
A_33_P34179	HDAC11	NM_024827
A_24_P11412	LAPTM5	NM_006762
A_23_P16342	PPCDC	NM_021823
A_24_P29085	PTPRS	NM_130853
A_23_P52298	NPM3	NM_006993
A_23_P38836	ZNF543	NM_213598
A_24_P59765	RHOT1	NM_018307
A_24_P20094	TSC22D4	NM_030935
A_23_P42163	ZBTB47	NM_145166
A_32_P74793	GLCE	NM_015554
A_23_P12707	PPRC1	NM_015062
A_23_P83094	TLE4	NM_007005
A_23_P30441	C1orf55	NM_152608
A_24_P48162	MPG	NM_002434
A_24_P32414	MUC5B	NM_002458
A_24_P10605	PCYT1A	ENST00000292823
A_24_P92697	FLT4	NM_182925
A_24_P20132	ZMYND11	NM_006624
A_23_P15958	PHF12	NM_001033561
A_23_P33968	ZNF675	NM_138330
A_23_P35612	KIAA1468	NM_020854
A_23_P37729	TGFA	NM_003236
A_23_P10206	HAS2	NM_005328
A_24_P22822	B4GALT6	NM_004775
A_33_P34141	ZNF260	NM_001012756
A_23_P61341	ACSS3	AK074267
A_23_P16524	ZNF208	NM_007153
A_23_P11652	USP1	NM_003368
A_23_P32736	DMXL2	NM_015263
A_32_P23139	LDHA	NM_005566
A_23_P20948	NEUROD1	NM_002500
A_24_P32381	MYCBP2	NM_015057
A_23_P23748	WDR47	NM_014969
A_23_P16976	ANXA4	NM_001153
A_24_P28318	CD14	NM_000591
A_23_P10834	ZNF571	NM_016536
A_32_P93403	CTSC	BX379032
A_23_P10464	C11orf2	NM_013265
A_32_P85266	LOC202181	NR_026921
A_23_P21795	HK1	NM_033500
A_23_P40936	NR2C2	NM_003298
A_33_P32511	SLC33A1	NM_004733
A_23_P13819	NCF2	NM_000433
A_33_P33119	ZFPM1	NM_153813
A_23_P17076	PDLIM5	NM_006457
A_23_P20644	FANCA	NM_000135
A_23_P11785	KIAA0101	NM_014736

A_23_P15479(	TRPC4AP	NM_015638
A_32_P47936(	WEE1	NM_003390
A_23_P16582(	ALS2CR8	NM_024744
A_23_P27936	ELL	NM_006532
A_33_P33055:	TNFRSF6B	NM_032945
A_23_P77279	C15orf29	NM_024713
A_33_P33174:	REXO1L1	NM_172239
A_23_P73780	IRAK1	NM_001569
A_23_P11758:	JDP2	NM_130469
A_23_P54198	C14orf118	NM_017926
A_23_P21108(	IFNAR2	NM_207585
A_32_P33054	CTPS	NM_001905
A_23_P9831	ETV5	NM_004454
A_24_P20673(	ZNF143	NM_003442
A_23_P25673!	PGCP	NM_016134
A_33_P33125(	PSD4	NM_012455
A_24_P10886:	SCML1	NM_001037540
A_23_P40123(	DDHD2	NM_015214
A_23_P42881(	AKIRIN2	NM_018064
A_24_P35448(	NAAA	NM_014435
A_23_P5234	ZNF43	NM_003423
A_32_P16846(	CASK	NM_003688
A_23_P69342	ACAA1	NM_001607
A_23_P59528	ACN9	NM_020186
A_33_P33516(	FOXR2	NM_198451
A_23_P4710	ZNF559	NM_032497
A_23_P10457(	CDK2AP2	NM_005851
A_23_P31791(	ZNF273	NM_021148
A_23_P10948(	PIK3IP1	NM_052880
A_23_P35737(	PTH2	NM_178449
A_23_P21917(	RXRA	NM_002957
A_24_P17118:	ACBD3	NM_022735
A_23_P32029(	ZNF827	NM_178835
A_32_P49547!	CRTC1	NM_001098482
A_23_P22224	EIF4EBP1	NM_004095
A_24_P46953	SGK3	NM_013257
A_33_P32140(	ATF3	NM_001040619
A_23_P2573	TMEM117	NM_032256
A_24_P12252(	WDR3	NM_006784
A_23_P87616	ATP5G2	NM_001002031
A_23_P60727	KCNMA1	NM_002247
A_23_P14814:	GRM8	NM_000845
A_24_P16882:	ZNF673	NM_017776
A_23_P67072	ZNF615	NM_198480
A_23_P25970:	RP11-35N6.1	NM_207299
A_33_P35902!	CXCL14	NM_004887
A_24_P20889:	COX18	NM_173827
A_23_P15223:	IRX3	NM_024336
A_33_P34180:	NUP62	NM_016553

A_23_P16029	ZNF256	NM_005773
A_23_P14775!	APLN	NM_017413
A_24_P94066(	MED1	NM_004774
A_32_P10882!	ZBTB41	NM_194314
A_23_P14394	CEP97	ENST00000341893
A_23_P74981	ZNF670	NM_033213
A_23_P145	HMGCL	NM_000191
A_32_P14037(	NFXL1	NM_152995
A_23_P78084	HOXB5	NM_002147
A_33_P33890:	SRD5A3	NM_024592
A_23_P10978	NR1I2	NM_003889
A_23_P37083(	KLHL14	NM_020805
A_23_P79803	VSTM2L	NM_080607
A_23_P18493	PTPN13	NM_080685
A_23_P41979!	SBF2	NM_030962
A_23_P12000:	SP110	NM_004510
A_23_P42630	MEPCE	NM_019606
A_24_P78556	RASSF8	NM_007211
A_23_P42101	KAZALD1	NM_030929
A_23_P10346	MRPL12	NM_002949
A_23_P28761	HNF4A	NM_001030004
A_33_P36426	ZNF460	NM_006635
A_23_P25602	LAS1L	NM_031206
A_24_P82749	PA2G4	NM_006191
A_24_P64918	ZBTB1	NM_014950
A_23_P37604	IL17RB	NM_018725
A_23_P12854	MED4	NM_014166
A_23_P30721	HLA-DQA1	NM_002122
A_33_P34204(	HACE1	NM_020771
A_32_P17730(	ZADH2	ENST00000322342
A_23_P586	DMAP1	NM_019100
A_24_P38378	DKFZp761E19	NM_138368
A_23_P14493	C14orf104	NM_018139
A_23_P99320	KRT18	NM_000224
A_32_P14692	POM121L1P	NR_024591
A_33_P34241	TCHHL1	NM_001008536
A_23_P21519	TYW1	NM_018264
A_23_P52442	BLNK	NM_013314
A_33_P32780(	ELF1	NM_001145353
A_23_P14026	CREBL2	NM_001310
A_24_P46111	PRKCI	NM_002740
A_23_P15656	KLHDC3	NM_057161
A_23_P12692!	SFPQ	NM_005066
A_23_P18966	P4HA2	NM_001017973
A_33_P33904!	YAF2	NM_001190979
A_23_P21864(	TNFRSF6B	NM_032945
A_23_P71989	UPP1	NM_181597
A_23_P85969	ZNF326	NM_182976
A_23_P19592	PGM3	NM_015599

A_23_P21690(	NR6A1	ENST00000487099
A_23_P21287(	SMAD1	NM_005900
A_23_P7612	PRDM9	NM_020227
A_33_P32645(	FCF1	NM_015962
A_23_P20099!	ST3GAL3	NM_174963
A_24_P41371	CC2D1A	NM_017721
A_23_P70231	ALDH7A1	NM_001182
A_32_P19268:	NPHP4	NM_015102
A_23_P20171:	S100A6	NM_014624
A_23_P49579	NEUROD2	NM_006160
A_23_P15618(	SLC22A4	NM_003059
A_23_P20406·	ATF7	NM_006856
A_23_P3766	NFAT5	NM_138714
A_24_P16249	ZNF479	NM_033273
A_24_P63003!	PHIP	NM_017934
A_23_P6714	SENP7	NM_020654
A_24_P33897:	NIPA2	NM_030922
A_33_P33610:	MYO5C	NM_018728
A_24_P23995	RNF187	NM_001010858
A_33_P32100(	NET1	NM_001047160
A_23_P64762	EEA1	NM_003566
A_23_P40635(	MFSD3	NM_138431
A_24_P15391	FOXI3	NM_001135649
A_33_P33275:	TBC1D9	NM_015130
A_23_P11363·	CBFB	NM_001755
A_23_P10787!	KLF1	NM_006563
A_23_P65262	N4BP2L2	NM_033111
A_24_P35286·	PSME3	NM_005789
A_33_P32312:	HIF1A	NM_181054
A_23_P50274:	RASAL2	NM_170692
A_23_P12254!	RDBP	NM_002904
A_24_P87368(	CENPN	NM_001100624
A_33_P32362(	REXO1L1	NM_172239
A_24_P34198:	ATF7IP2	NM_024997
A_23_P25283:	HINT1	NM_005340
A_23_P55632	SERPINB3	NM_006919
A_23_P92154	MBD4	NM_003925
A_23_P9289	RFX3	NM_134428
A_32_P45953:	FCHO1	NM_015122
A_23_P40805	KLF15	NM_014079
A_23_P13442(	GPNMB	NM_001005340
A_23_P13493!	DUSP4	NM_001394
A_23_P71908	SNAPC3	NM_001039697
A_32_P31377:	ZNF280B	NM_080764
A_32_P18984!	ZNF680	NM_178558
A_23_P12034!	PELI1	NM_020651
A_23_P56369	ABCA12	NM_173076
A_23_P14204!	PRDX2	NM_005809
A_23_P21308!	SLC10A7	NM_001029998

A_23_P40709	NFXL1	NM_152995
A_23_P14244	MYO1F	NM_012335
A_24_P34627	SRF	NM_003131
A_23_P71624	PAX5	NM_016734
A_23_P11600	MAPK8IP1	NM_005456
A_23_P20819	ZNF577	NR_024181
A_23_P19652	LRP11	NM_032832
A_33_P33574	REPS2	NM_004726
A_23_P14618	RRS1	NM_015169
A_24_P33659	SMCP	NM_030663
A_23_P59163	ATF6B	NM_004381
A_23_P21344	UTP15	NM_032175
A_23_P76799	BAZ1A	NM_013448
A_24_P36099	CLEC1A	NM_016511
A_23_P25994	STK35	NM_080836
A_23_P13018	FIBP	NM_198897
A_33_P33461	RUFY4	NM_198483
A_23_P8432	COPS6	NM_006833
A_23_P15680	TULP4	NM_020245
A_33_P32770	GABRB3	NM_000814
A_32_P14255	ZNF562	NM_017656
A_32_P34387	SPATA7	NM_018418
A_24_P3415	PPP2R2C	NM_020416
A_23_P12970	TERF2IP	NM_018975
A_23_P10656	GALNS	NM_000512
A_24_P93263	MED28	ENST00000237380
A_23_P39119	RAB11FIP1	NM_001002233
A_23_P43317	PRDM12	NM_021619
A_23_P61487	LRRC20	NM_018205
A_23_P32030	AKR7L	NM_201252
A_23_P90220	ZNF585A	NM_152655
A_24_P10286	TMEM61	NM_182532
A_23_P56785	ALPI	NM_001631
A_33_P32850	LASS5	NM_147190
A_23_P36569	DMRT2	NM_006557
A_23_P25894	DNAJB9	NM_012328
A_23_P21559	SRPK2	NM_182691
A_24_P28204	ZNF28	NM_006969
A_23_P19565	ASCC3	NM_006828
A_23_P60793	ASMTL-AS1	NR_026710
A_33_P33998	USP39	NM_006590
A_32_P78990	HEATR5B	NM_019024
A_23_P2801	ELF1	NM_172373
A_33_P32791	NBPF14	XM_001726946
A_23_P31697	SYNJ2	NM_003898
A_33_P32695	GABPA	NM_002040
A_23_P39885	DPP4	NM_001935
A_23_P25472	PIM1	NM_002648
A_23_P94494	TLE4	NM_007005

A_32_P15342[illegible]	DDX52	ENST00000349699
A_32_P32728[illegible]	ZNF469	NM_001127464
A_23_P39682	ZAP70	NM_001079
A_24_P27152[illegible]	JOSD1	NM_014876
A_24_P83183	WHSC2	NM_005663
A_24_P26073	LRPPRC	NM_133259
A_23_P11929[illegible]	ECSIT	NM_016581
A_23_P86421	NCOA4	NM_005437
A_24_P92752	C1orf194	NM_001122961
A_23_P31306	HOXA13	NM_000522
A_23_P38891[illegible]	CCDC101	NM_138414
A_24_P29596[illegible]	SLC38A2	NM_018976
A_32_P60223	ING5	NM_032329
A_23_P44342	ENTPD8	NM_001033113
A_23_P15647	NLK	NM_016231
A_23_P36805	NT5DC3	NM_001031701
A_24_P60268	LRRTM4	NM_024993
A_23_P45185	FIGF	NM_004469
A_23_P14641[illegible]	C9orf5	NM_032012
A_24_P13647[illegible]	BTN2A3	NR_027795
A_23_P91350	RP5-1022P6.2	NM_019593
A_32_P44886[illegible]	ATF6B	NM_004381
A_23_P58443	ANKHD1-EIF4[illegible]	NM_020690
A_23_P47135	NR1H3	NM_005693
A_23_P6140	OPRL1	NM_182647
A_23_P36637[illegible]	TDGF1	NM_003212
A_23_P25757[illegible]	SRA1	NM_001035235
A_33_P32304[illegible]	ZNF774	NM_001004309
A_23_P11147[illegible]	STAG3L4	NM_022906
A_23_P12327[illegible]	FZD6	NM_003506
A_23_P16129[illegible]	HSPA12A	AK023936
A_23_P52697	CD248	NM_020404
A_24_P21475[illegible]	NR3C1	NM_001018077
A_32_P14170	PDCD6IP	NM_013374
A_23_P59349	HECA	NM_016217
A_24_P30862[illegible]	HEMK1	NM_016173
A_23_P14355[illegible]	CLTCL1	NM_007098
A_23_P20903[illegible]	ZNF302	NM_018443
A_23_P3584	PLA2G15	NM_012320
A_23_P97725	STX6	NM_005819
A_23_P50090[illegible]	ZNF19	NM_006961
A_32_P6862	WWTR1	NM_015472
A_23_P13465	ELF5	NM_198381
A_24_P21115[illegible]	EXOSC5	NM_020158
A_23_P12951[illegible]	MRPL28	NM_006428
A_23_P41344	EREG	NM_001432
A_32_P33386[illegible]	ZBTB20	NM_015642
A_24_P22621[illegible]	CEP120	NM_153223
A_23_P21165[illegible]	CERK	NM_022766

A_23_P68665	ADRM1	NM_007002
A_23_P14232	FZR1	NM_001136198
A_23_P20836	ITPKC	NM_025194
A_24_P19280	CARD17	NM_001007232
A_33_P32488	KRBA2	NM_213597
A_23_P63926	PCBD1	NM_000281
A_23_P31362	COBL	NM_015198
A_23_P37908	SOX21	NM_007084
A_23_P71827	ZBTB26	NM_020924
A_33_P33506	LMX1B	NM_001174146
A_23_P21203	DLG1	NM_004087
A_23_P66854	KRT20	NM_019010
A_23_P57413	PPM1F	NM_014634
A_33_P32186	PNN	NM_002687
A_32_P89073	SAMD1	NM_138352
A_33_P32154	LYL1	NM_005583
A_23_P35045	PRDM1	NM_001198
A_23_P11751	ARID4A	NM_002892
A_23_P80048	FER1L4	NR_024377
A_24_P11396	NT5C1A	NM_032526
A_24_P93711	ZNF292	NM_015021
A_23_P10999	MYH15	NM_014981
A_23_P73093	INSIG1	NM_198336
A_33_P32363	NOBOX	NM_001080413
A_23_P82693	PABPC1	NM_002568
A_32_P18294	RPS3	NM_001005
A_23_P16072	AP4B1	NM_006594
A_24_P39101	KCTD10	NM_031954
A_23_P10812	ZNF223	NM_013361
A_23_P20868	NUP62	NM_153719
A_23_P31943	H2AFX	NM_002105
A_33_P34086	CD2	AK310676
A_23_P13573	ZNF627	NM_145295
A_33_P32597	TMEM222	NM_032125
A_23_P99211	POP5	NM_015918
A_24_P32231	ERN1	AK055561
A_24_P28576	EDEM1	NM_014674
A_33_P32500	SNX17	NM_014748
A_23_P31718	LRRFIP2	NM_006309
A_24_P11886	ZNF678	NM_178549
A_24_P51485	CSDE1	NM_001007553
A_23_P37291	RORC	NM_005060
A_23_P13471	HRSP12	NM_005836
A_23_P74663	TAF1A	NM_005681
A_23_P45140	KRAS	NM_033360
A_23_P42588	TRIO	NM_007118
A_24_P54955	TTC3	NM_003316
A_24_P18520	TP53I13	NM_138349
A_23_P55731	CIC	NM_015125

A_32_P122972	MSI2	NM_138962
A_24_P254789	COL14A1	NM_021110
A_23_P106425	C15orf38	NM_182616
A_33_P331174	ZNF774	NM_001004309
A_24_P256513	AGGF1	BC002828
A_24_P16892	TAF2	NM_003184
A_24_P134789	KRTAP10-10	NM_181688
A_23_P200637	FBXO44	NM_001014765
A_23_P79289	COBLL1	NM_014900
A_23_P153646	FSD1	NM_024333
A_33_P338093	ZNF800	NM_176814
A_32_P330267	ITGBL1	NM_004791
A_23_P49988	KRTAP4-2	NM_033062
A_33_P330359	PRAMEF11	NM_001146344
A_23_P335143	SNRPN	U81001
A_33_P336815	SLA2	NM_032214
A_23_P314115	BMI1	NM_005180
A_23_P343411	AGRN	NM_198576
A_23_P14609	DMXL2	NM_015263
A_33_P329151	VCY	NM_004679
A_23_P136874	ATRX	NM_000489
A_24_P343695	RET	NM_020630
A_24_P231513	ACTR3	NM_005721
A_23_P161091	ZMYM1	NM_024772
A_24_P236799	RAB31	NM_006868
A_24_P79199	PRIC285	NM_033405
A_24_P345540	ZC3H8	NM_032494
A_24_P268676	BHLHE40	NM_003670
A_33_P330772	KRTAP5-11	NM_001005405
A_23_P119533	EFNA2	NM_001405
A_24_P804992	NFIB	NM_005596
A_32_P353993	SAMD5	NM_001030060
A_24_P128145	ATF2	NM_001880
A_24_P203284	KRTAP12-2	NM_181684
A_32_P444224	MED16	NM_005481
A_23_P61886	TSPAN5	ENST00000305798
A_23_P110345	CHIC2	NM_012110
A_33_P326501	PEX6	NM_000287
A_24_P281801	ZNF732	NM_001137608
A_24_P916797	AKAP9	AK000270
A_33_P324950	NFIC	NM_205843
A_32_P794056	TSC22D1	NM_183422
A_32_P120940	ASL	NM_001024943
A_32_P196115	ZNF498	NM_145115
A_33_P325686	ZNF83	NM_001105549
A_32_P395163	INPP4B	NM_003866
A_23_P257164	AMT	NM_000481
A_23_P60296	OSTF1	NM_012383
A_32_P100428	UBE2L3	NM_003347

A_23_P31464	ZNF280C	NM_017666
A_33_P33931	CAPN5	NM_004055
A_23_P43594	SAMD1	NM_138352
A_23_P38878	DMAP1	NM_019100
A_24_P89858	TRIM26	NM_003449
A_32_P19511	ENST0000031	ENST00000317596
A_23_P39003	TMEM20	NM_153226
A_23_P95672	IGF2BP2	NM_006548
A_23_P12224	SLC6A18	NM_182632
A_23_P16858	CCDC126	NM_138771
A_23_P25515	MTMR6	NM_004685
A_24_P11593	GPR44	NM_004778
A_23_P11031	CWH43	NM_025087
A_23_P74391	OPN3	NM_014322
A_23_P12581	RBM10	NM_005676
A_24_P37286	ETV6	NM_001987
A_23_P33960	ZNF578	NM_001099694
A_32_P11919	TPM3	NM_001043352
A_24_P22144	OXR1	NM_181354
A_23_P21045	KCNK15	BC082981
A_23_P10941	THAP7	NM_030573
A_33_P32933	LRRC1	AU119761
A_23_P48358	PCCA	NM_000282
A_24_P11900	FAM98A	NM_015475
A_33_P33548	C9orf171	NM_207417
A_23_P31359	KRT7	NM_005556
A_23_P33219	IRF8	NM_002163
A_24_P76521	GSG2	AK056691
A_23_P15705	AP4M1	NM_004722
A_23_P71117	SLC25A40	NM_018843
A_32_P20817	RPS3A	NM_001006
A_23_P89480	HOXB4	NM_024015
A_24_P40124	ZNF160	NM_198893
A_23_P34218	WAPAL	NM_015045
A_23_P40339	DKFZP586I14	NR_002186
A_23_P35815	CFL1	NM_005507
A_32_P15503	NFIA	NM_001134673
A_23_P21473	FBXL4	NM_012160
A_23_P30204	HMHB1	NM_021182
A_23_P13937	RPS3	NM_001005
A_23_P36631	SP1	NM_138473
A_23_P61466	CD163L1	NM_174941
A_23_P13891	FLI1	NM_002017
A_32_P24711	ZNF642	NM_198494
A_23_P25523	STEAP4	NM_024636
A_32_P68614	GPBP1	NM_022913
A_32_P50804	ONECUT1	NM_004498
A_23_P5281	LYL1	NM_005583
A_32_P14515	RPL31	NM_000993

A_33_P32181	POLR2A	NM_000937
A_23_P62465	ZFY	NM_003411
A_23_P15765	HEY1	NM_001040708
A_23_P11130	RBM16	NM_014892
A_24_P32118	ZFYVE26	NM_015346
A_32_P21836	ZNF542	NR_003127
A_33_P35090	YBX1P2	BF683093
A_33_P33233	ZNF497	NM_198458
A_23_P76388	UNG	NM_003362
A_24_P81170	PPFIBP1	NM_003622
A_23_P20995	S100A13	ENST00000339556
A_23_P12194	SNCB	NM_001001502
A_23_P41128	KAT2B	NM_003884
A_32_P15425	YWHAQ	NM_006826
A_33_P32909	SMC1A	NM_006306
A_24_P20634	ZNF746	NM_152557
A_23_P64102	RIN1	NM_004292
A_23_P60130	MAL2	NM_052886
A_24_P15110	ZFP28	NM_020828
A_23_P13523	TLE1	NM_005077
A_24_P24704	ZNF492	NM_020855
A_23_P4798	ZNF581	NM_016535
A_33_P33179	KRTAP20-1	NM_181615
A_23_P33765	ALPI	NM_001631
A_24_P11329	AP2S1	NM_004069
A_23_P10276	ERBB4	NM_005235
A_23_P12821	SOCS2	NM_003877
A_23_P15642	MAN1A1	NM_005907
A_33_P32292	HIST2H2BE	NM_003528
A_23_P14437	ANKRD17	NM_032217
A_23_P89998	EXOSC5	NM_020158
A_24_P21676	TOMM20	NM_014765
A_23_P10611	SIX6	NM_007374
A_23_P32673	SUV420H1	NM_017635
A_23_P20282	CTTN	NM_005231
A_33_P32137	ZNF585B	NM_152279
A_24_P46577	RPSAP52	NR_026825
A_32_P20078	TIGD1	NM_145702
A_23_P83671	WDR6	NM_018031
A_23_P68006	IL1R1	NM_000877
A_23_P12920	IDH2	NM_002168
A_23_P12733	PI4K2A	NM_018425
A_23_P60376	EDF1	NM_153200
A_32_P26092	TNFRSF4	NM_003327
A_23_P20925	TNKS	NM_003747
A_23_P15501	LIF	NM_002309
A_23_P13120	NR4A2	NM_006186
A_32_P34020	THSD1P1	NR_002816
A_23_P10504	MRPL23	NM_021134

A_23_P13691(	WDR13	NM_017883
A_24_P34039(	CPEB2	NM_182485
A_23_P71644	FANCG	NM_004629
A_23_P42029:	C11orf45	NM_145013
A_24_P11445!	RYBP	NM_012234
A_23_P32480:	VARS2	NM_020442
A_23_P64860	SELPLG	NM_003006
A_23_P31031:	TCOF1	NM_001008656
A_23_P38636(	HDX	NM_144657
A_24_P33611:	CABIN1	NM_012295
A_23_P94533	CTSL1	NM_001912
A_23_P11421(	POU3F4	NM_000307
A_23_P11779:	CLN6	NM_017882
A_23_P67737	ZNF568	AL832834
A_23_P84344	SIGIRR	NM_021805
A_33_P34161:	LOC10013087.	NR_024569
A_23_P14165	GPR18	NM_005292
A_23_P68486	C20orf108	NM_080821
A_23_P21897:	AFF4	NM_014423
A_32_P20756:	NUCB2	NM_005013
A_23_P34383:	PARP11	NM_020367
A_32_P44305	MTX3	NM_001010891
A_33_P32698:	FAM160B2	NM_022749
A_23_P80456	THRB	NM_000461
A_23_P99405	ZMYM2	NM_003453
A_24_P91314(	HOPX	NM_139211
A_23_P12140!	RBM15B	NM_013286
A_23_P37101:	ZNF227	NM_182490
A_23_P12794!	ADM	NM_001124
A_23_P9145	UHRF2	NM_152896
A_23_P14950!	ZBTB48	NM_005341
A_24_P13919:	ITCH	NM_031483
A_32_P85351!	PRMT10	ENST00000322396
A_23_P20586:	NR2E3	NM_014249
A_23_P20578!	GABPB1	NM_002041
A_23_P15965(	COX7B	NM_001866
A_24_P28191:	MLL	AF272382
A_33_P33434	ZNF316	XR_079205
A_23_P13117(	GTDC1	AF090899
A_24_P91592(	COBLL1	BC006264
A_23_P20042!	S100PBP	NM_022753
A_23_P12077:	SLC25A1	AK292313
A_23_P34941(	ERBB3	NM_001982
A_23_P13138:	FANCL	NM_018062
A_24_P42136	KRT18	NM_000224
A_24_P85419!	PHB	NM_002634
A_24_P12687:	SUPT6H	NM_003170
A_23_P13364!	FAM8A1	NM_016255
A_24_P37483	OTUD1	NM_001145373

A_32_P76905[illegible]	AKT3	NM_181690
A_23_P14548[illegible]	ULBP2	NM_025217
A_23_P39014[illegible]	RALGPS1	NM_014636
A_32_P82077[illegible]	FLJ35776	NR_024101
A_33_P32384[illegible]	SLC2A7	NM_207420
A_23_P13380[illegible]	TAF8	NM_138572
A_23_P12435	PPP1R12B	NM_032103
A_24_P41637[illegible]	HOXB4	NM_024015
A_23_P16044[illegible]	PPP1R15B	NM_032833
A_23_P31452[illegible]	FLOT1	NM_005803
A_24_P91405	ZNF709	NM_152601
A_23_P20333[illegible]	FAM111A	NM_022074
A_23_P21787[illegible]	ARID1A	NM_006015
A_33_P33636[illegible]	BLNK	NM_013314
A_23_P12708[illegible]	CUEDC2	NM_024040
A_24_P41437[illegible]	PPP3CA	NM_000944
A_23_P16889[illegible]	SORBS3	NM_005775
A_23_P40146[illegible]	IL20	NM_018724
A_24_P54191[illegible]	DENND5B	NM_144973
A_23_P94623	PPP6C	NM_001123355
A_23_P20147[illegible]	KLHL21	NM_014851
A_32_P47960[illegible]	FAM98A	NM_015475
A_23_P13693[illegible]	SYP	NM_003179
A_23_P50060[illegible]	TRIM4	NM_033017
A_23_P10118[illegible]	NARS	NM_004539
A_23_P17135[illegible]	WDR44	NM_019045
A_32_P11983[illegible]	ZNF584	NM_173548
A_24_P35152[illegible]	CBR4	AK027337
A_23_P90508	ZNF486	NM_052852
A_24_P23625	HS3ST3B1	ENST00000360954
A_32_P98927	STAG3L4	NM_022906
A_24_P13281[illegible]	OPALIN	NM_001040103
A_32_P78740[illegible]	DCST1	NM_152494
A_33_P33625[illegible]	MED31	NM_016060
A_23_P15559[illegible]	FMO3	NM_001002294
A_23_P82814	FBXO32	NM_058229
A_23_P85218	SOX3	NM_005634
A_23_P15442[illegible]	HOXD12	NM_021193
A_23_P20372[illegible]	RAB6A	NM_002869
A_23_P12488[illegible]	CXCL10	NM_001565
A_24_P28458[illegible]	ZNF559	NM_032497
A_23_P16859[illegible]	CCDC126	NM_138771
A_24_P33705[illegible]	CCNL1	NM_020307
A_23_P29263	CSNK1E	NM_152221
A_32_P47643	FAM110C	ENST00000327669
A_32_P14422[illegible]	MYL12B	NM_033546
A_23_P31615	PMP2	NM_002677
A_24_P34599[illegible]	CANX	NM_001746
A_33_P34213[illegible]	ZNF169	NM_194320

A_23_P50786	CLIP3	NM_015526
A_33_P33658	MRPL12	NM_002949
A_23_P774	DENND4B	NM_014856
A_24_P41975	TDRKH	NM_001083965
A_23_P12509	CRELD2	NM_024324
A_23_P12581	RBM10	NM_005676
A_23_P90172	PPP1R15A	NM_014330
A_23_P14780	ZNF654	NM_018293
A_24_P41058	VGLL4	NM_014667
A_24_P41615	RBL2	NM_005611
A_23_P20612	FMN1	NM_001103184
A_23_P20842	F2RL3	NM_003950
A_32_P22799	MED11	NM_001001683
A_23_P75162	VENTX	NM_014468
A_24_P30559	ADRM1	NM_007002
A_23_P25615	SOHLH2	NM_017826
A_33_P32873	ASCL4	NM_203436
A_23_P13601	FBXO8	NM_012180
A_24_P25932	MIB2	NM_080875
A_23_P21227	RPL29	NM_000992
A_24_P79985	LIPH	ENST00000296252
A_23_P13066	DNM2	NM_001005360
A_23_P11509	RAB25	NM_020387
A_23_P10679	SIAH1	NM_003031
A_23_P50530	AP2S1	NM_004069
A_23_P14772	SLC35E3	NM_018656
A_23_P40328	OTX1	NM_014562
A_23_P33093	ST6GALNAC5	NM_030965
A_23_P66608	KAT2A	NM_021078
A_24_P10576	KDM3A	NM_018433
A_23_P11879	TMEM107	NM_032354
A_33_P32177	TLE1	NM_005077
A_24_P67350	SLC24A3	NM_020689
A_33_P32520	DYSFIP1	NM_001007533
A_33_P34035	TMEM188	NM_153261
A_23_P15480	EPB41L1	NM_012156
A_24_P68247	TRIM4	NM_033017
A_24_P18686	CHMP5	NM_016410
A_23_P27677	IRF3	NM_001571
A_32_P19130	SPATA6	ENST00000371847
A_24_P26494	COMP	NM_000095
A_23_P50508	PLA2G4C	NM_003706
A_33_P33596	IL16	NM_001172128
A_24_P83437	ZNF326	NM_182975
A_32_P627	TAF9B	AK074449
A_23_P65031	DYNLL1	NM_001037494
A_23_P34142	WBP5	NM_016303
A_23_P35354	DLX6	NM_005222
A_23_P20515	LMO7	NM_005358

A_23_P20363	FAM164A	NM_016010
A_33_P33156	NKX1-1	XM_937659
A_23_P14606	SPAG1	NM_003114
A_23_P34747	FZD3	NM_017412
A_24_P25741	CXCL2	NM_002089
A_32_P10277	ZNF626	NM_001076675
A_23_P57925	FEZF2	NM_018008
A_24_P10111	CNOT1	NM_206999
A_23_P2601	HSP90B1	NM_003299
A_23_P34597	ZBTB7C	NM_001039360
A_32_P45410	HAUS5	NM_015302
A_23_P32444	MXRA8	NM_032348
A_23_P91964	RASA2	NM_006506
A_23_P31893	ST3GAL1	NM_003033
A_33_P33602	EVX2	NM_001080458
A_23_P36819	LSM11	NM_173491
A_32_P82635	FUCA1	NM_000147
A_23_P27197	KRTAP4-5	NM_033188
A_33_P33309	NXN	BC104634
A_33_P32361	UNG	NM_003362
A_23_P25282	SCAP	NM_012235
A_24_P20954	TFAP2B	NM_003221
A_23_P32620	SGMS2	NM_152621
A_33_P33260	FUBP3	NM_003934
A_23_P33581	TOB2	NM_016272
A_23_P20833	PDE4A	NM_006202
A_23_P21833	CYB561	NM_001017916
A_23_P97038	POU3F1	NM_002699
A_32_P41834	ZBTB7B	NM_015872
A_23_P26696	TRIM47	NM_033452
A_23_P16346	SNRPD2	NM_177542
A_23_P16661	AGTR1	NM_031850
A_23_P16191	CCDC86	NM_024098
A_23_P7783	KDM3B	NM_016604
A_23_P89649	KRTAP3-3	NM_033185
A_23_P14582	PPP1R9A	NM_017650
A_23_P16446	ADNP2	NM_014913
A_24_P69784	PACS1	NM_018026
A_23_P75529	PKNOX2	NM_022062
A_23_P47879	STAT6	NM_003153
A_23_P20781	PAIP1	NM_006451
A_33_P32590	MYOCD	NM_001146313
A_23_P38626	C4orf26	NM_178497
A_23_P35612	ZNF451	NM_001031623
A_23_P40213	ZNF280B	NM_080764
A_23_P27186	KRT26	NM_181539
A_32_P19929	FAM60A	NM_001135812
A_23_P50389	NAT14	NM_020378
A_23_P88840	DUS2L	NM_017803

A_32_P22787(	SLC30A4	ENST00000261867
A_24_P45028!	CCDC153	NM_001145018
A_24_P77676	HSPA9	NM_004134
A_33_P33582:	NES	NM_006617
A_24_P94162!	ZNF70	NM_021916
A_32_P92456!	TBL1Y	AK056903
A_23_P3602	NUDT7	NM_001105663
A_23_P56140	CSNK1G2	NM_001319
A_23_P62731	MRPS14	NM_022100
A_23_P21743:	ATP1B4	NM_012069
A_32_P18036!	SLC25A41	NM_173637
A_23_P14240:	ZNF101	NM_033204
A_24_P37037:	CBX6	NM_014292
A_23_P34154:	NKX2-8	NM_014360
A_32_P53280:	OBSCN	NM_001098623
A_23_P32175	LHX6	NM_014368
A_23_P30926:	AKAP9	NM_005751
A_23_P83798	ALX1	NM_006982
A_23_P21354:	SNX18	NM_052870
A_23_P53247	DTX3	NM_178502
A_24_P28607!	AFF4	NM_014423
A_23_P15418!	SAP130	NM_024545
A_23_P10257!	SLC2A4RG	NM_020062
A_33_P36668:	C10orf55	NM_001001791
A_33_P33735(	SPTAN1	NM_001130438
A_23_P37702	TPSAB1	NM_003294
A_24_P71904	HPGD	NM_000860
A_23_P25671(	SMARCA5	NM_003601
A_24_P3704	GTF2H2B	NR_033417
A_24_P94169!	PCGF5	NM_032373
A_23_P56798	ACP1	NM_004300
A_23_P87964	ESD	NM_001984
A_24_P63642	T	NM_003181
A_23_P16358(	CENPT	NM_025082
A_33_P32884(	PSMA8	NM_144662
A_23_P40108:	ZNF575	NM_174945
A_23_P70775	E2F3	NM_001949
A_23_P11130:	RBM16	NM_014892
A_24_P93245:	BAZ2B	BX641037
A_23_P31951:	KLF11	NM_003597
A_33_P35252(	A2ML1	NM_144670
A_33_P33293:	FASN	NM_004104
A_23_P34536:	C18orf25	NM_145055
A_24_P17160:	NKTR	NM_005385
A_23_P11093!	CRISP2	NM_003296
A_24_P37718:	SEPSECS	NM_016955
A_23_P21356:	F2R	NM_001992
A_23_P16632:	MICAL3	NM_015241
A_24_P39215:	C11orf86	NM_001136485

A_32_P43812	DCUN1D4	NM_001040402
A_23_P43617	PTGES2	NM_025072
A_23_P14699(	WWP1	NM_007013
A_23_P13654(	PIGG	NM_017733
A_24_P41863(	MACF1	NM_012090
A_23_P13245(	CCNL1	NM_020307
A_24_P39949(	KLK10	NM_002776
A_23_P21198(	SNRK	NM_017719
A_24_P21997(	C20orf11	NM_017896
A_23_P87653	KRT6A	NM_005554
A_32_P22761(	LOC10028859(	AK024457
A_33_P34149(	PTPRS	BC029496
A_23_P20728(	APPBP2	NM_006380
A_32_P82086(	TERC	NR_001566
A_23_P11324(	RBPJ	ENST00000342320
A_23_P20663(	PKMYT1	NM_182687
A_32_P39373	ALX1	NM_006982
A_33_P32842(	ZNF778	NM_182531
A_33_P32851(	ZNF599	NM_001007248
A_24_P19016(	TMEM97	NM_014573
A_32_P95998	ETV3L	NM_001004341
A_24_P72184(	KLHL9	AK022805
A_23_P11738(	MIA2	NM_054024
A_24_P33077(	CALCOCO2	NM_005831
A_23_P39019(	NUPL1	NM_014089
A_23_P11947(	EBI3	NM_005755
A_24_P35495(	CCDC126	NM_138771
A_23_P13335(	ZFP2	NM_030613
A_32_P10927(	HSF5	NM_001080439
A_32_P7791	AHCTF1	NM_015446
A_32_P17709(	ZNF557	NM_024341
A_23_P13887(	PACS1	NM_018026
A_23_P43610(	MIB2	NM_080875
A_23_P56590	C1D	NM_006333
A_23_P42087	BPHL	NM_004332
A_23_P16536(	ASB1	NM_001040445
A_32_P84846	ZNF780B	NM_001005851
A_24_P17308(	STAT5A	NM_003152
A_24_P93043(	RASGEF1B	BX648337
A_23_P50217	ZNF671	NM_024833
A_32_P85273	GOLGA8A	NM_181077
A_33_P32174(	ZNF705A	NM_001004328
A_23_P41820(	ZNF251	NM_138367
A_33_P33539(	PIK3CD	NM_005026
A_23_P66085	N4BP1	NM_153029
A_23_P21583(	ATP5J2	NM_004889
A_24_P22452(	C17orf75	NM_022344
A_24_P14931(	ULBP2	NM_025217
A_32_P95757	TMEM217	NM_145316

A_23_P16572	EIF4E2	NM_004846
A_23_P58497	UTP15	NM_032175
A_23_P9582	TUFM	NM_003321
A_23_P21008	OSGEPL1	NM_022353
A_24_P37130	C3orf63	NM_015224
A_23_P15736	FAM3C	NM_014888
A_23_P97712	C1orf68	NM_001024679
A_33_P34069	KIF24	NM_194313
A_23_P39137	GRLF1	NM_004491
A_33_P33764	CYP17A1	NM_000102
A_33_P32180	AKR7L	NM_201252
A_23_P13086	CCDC94	NM_018074
A_23_P37610	TICAM1	NM_182919
A_23_P13447	C7orf50	NM_032350
A_23_P11338	GRIP1	NM_021150
A_23_P21460	FLOT1	NM_005803
A_23_P51499	PRELP	NM_002725
A_23_P55880	ZNF564	NM_144976
A_23_P11712	RARG	NM_000966
A_33_P32864	FANCA	NM_001018112
A_23_P20789	CSNK1D	NM_001893
A_24_P92292	MYO9B	NM_004145
A_24_P33172	TMED7	NM_181836
A_24_P17901	FAM136B	NM_001012983
A_24_P11124	SVIP	NM_148893
A_23_P33891	PHLDA1	NM_007350
A_32_P20229	NDUFB6	NM_002493
A_23_P15453	PRPF6	NM_012469
A_23_P15305	PRPSAP1	NM_002766
A_24_P36685	USHBP1	NM_031941
A_23_P15039	FXYD6	NM_022003
A_23_P87952	SOX5	NM_152989
A_23_P31769	UIMC1	NM_016290
A_23_P33960	ZNF578	NM_001099694
A_23_P10418	ELF3	NM_004433
A_23_P11570	PCGF6	NM_001011663
A_23_P3819	ZNF747	NM_023931
A_23_P13995	CDK8	NM_001260
A_32_P69296	ZBTB44	NM_014155
A_23_P21278	MTBP	AK022122
A_23_P2489	C12orf57	BU533173
A_23_P15743	AZIN1	NM_015878
A_23_P42532	GMCL1L	NR_003281
A_23_P40837	HSPA12A	NM_025015
A_23_P14950	ZBTB48	NM_005341
A_23_P11321	TMEM45A	NM_018004
A_23_P12678	F3	NM_001993
A_33_P32953	CLEC1A	NM_016511
A_24_P14513	OSMR	NM_001168355

A_24_P16378	HGSNAT	NM_152419
A_23_P74081	TP73	NM_005427
A_23_P3204	MAPK6	NM_002748
A_33_P32391[illegible]	SYT7	NM_004200
A_23_P63319	SOAT1	NM_003101
A_23_P14550[illegible]	MED23	NM_004830
A_24_P15135[illegible]	SGK2	NM_170693
A_23_P12460[illegible]	TMEM45A	NM_018004
A_23_P11560[illegible]	ARHGAP21	NM_020824
A_23_P40078	XPO1	NM_003400
A_24_P27042[illegible]	DPF3	AK124946
A_32_P64162	SNX17	NM_014748
A_23_P42751[illegible]	IL22	NM_020525
A_32_P11735[illegible]	LIMCH1	NM_014988
A_24_P24060	RHOXF2	NM_032498
A_23_P20313[illegible]	UBE4A	NM_004788
A_33_P34239[illegible]	CBX2	NM_032647
A_23_P30631[illegible]	SYT9	NM_175733
A_24_P36484[illegible]	ZNF213	NM_004220
A_33_P33035[illegible]	EDA	NM_001005610
A_24_P81965	RAP2A	NM_021033
A_24_P13431[illegible]	ADNP	ENST00000349014
A_23_P21082[illegible]	PCMTD2	NM_018257
A_24_P20060[illegible]	PCBD2	NM_032151
A_32_P40121[illegible]	C15orf42	NM_152259
A_24_P64442	EHF	NM_012153
A_23_P87625	NEUROD4	NM_021191
A_23_P33776[illegible]	ENAH	NM_018212
A_23_P10091[illegible]	GIT1	NM_014030
A_23_P87251	MRPL17	NM_022061
A_33_P32712[illegible]	HOXB2	NM_002145
A_32_P30877[illegible]	ZNF620	NM_175888
A_23_P11331[illegible]	P4HTM	NM_177938
A_23_P21490[illegible]	MTHFD1L	NM_015440
A_23_P35564	SEC31B	NM_015490
A_23_P34440[illegible]	AHRR	NM_020731
A_23_P12958[illegible]	ZNF75A	NM_153028
A_23_P64585	PHOX2A	NM_005169
A_32_P77886[illegible]	DACH2	NM_053281
A_23_P20009[illegible]	SPSB1	NM_025106
A_23_P39925[illegible]	RNF182	NM_152737
A_23_P20785[illegible]	TNS4	NM_032865
A_23_P16099[illegible]	FMO4	NM_002022
A_33_P34062[illegible]	TAF1A	NM_005681
A_23_P25694[illegible]	MSC	NM_005098
A_24_P56407	FCF1	NM_015962
A_33_P33302[illegible]	CXCL1	NM_001511
A_23_P38593[illegible]	PAN3	NM_175854
A_23_P20859[illegible]	LDLR	NM_000527

A_23_P11893:	ZNF532	NM_018181
A_23_P16082{	C1orf159	NM_017891
A_23_P20554{	MAX	NM_145113
A_23_P20384:	BAZ2A	NM_013449
A_24_P36544:	SLC2A4RG	NM_020062
A_33_P33034:	MAN1A1	NM_005907
A_23_P47746	ODZ4	NM_001098816
A_33_P32831:	TMEM209	NM_032842
A_23_P20946.	CSRNP3	NM_024969
A_23_P34566(	ZGPAT	NM_181485
A_32_P72525{	NWD1	NM_001007525
A_24_P33038!	SLC22A17	NM_016609
A_23_P16823(	PRPF4B	NM_003913
A_24_P11597:	RIC8A	NM_021932
A_23_P14322	NKX2-1	NM_003317
A_32_P17999{	DMRTC1	NM_033053
A_23_P62868	EXOSC10	NM_001001998
A_23_P46482	IL20	NM_018724
A_33_P32317(	ZNF677	NM_182609
A_23_P59441	SLC35B4	NM_032826
A_24_P31022.	ZNF347	NM_032584
A_23_P37486:	CD55	NM_000574
A_23_P21467{	CUTA	NM_015921
A_24_P50458	TERF1	NM_017489
A_23_P31671	UQCRB	NM_006294
A_23_P13068{	ELOF1	NM_032377
A_23_P20739{	NBR1	NM_031858
A_24_P16780(	IDH3A	NM_005530
A_32_P27972	SEC63	NM_007214
A_23_P11338:	GRIP1	NM_021150
A_24_P22204:	HMGA1	NM_002131
A_24_P81149	ENY2	NM_020189
A_33_P33636:	NFYC	NM_001142590
A_23_P31355(	SLC25A41	NM_173637
A_23_P487	UCK2	NM_012474
A_23_P31373.	KIAA1731	NM_033395
A_23_P36183	GTF2H1	NM_005316
A_24_P17434:	CCNT2	NM_058241
A_23_P13743	HOXC8	NM_022658
A_23_P15643:	MAN1A1	NM_005907
A_23_P11546:	S100A5	NM_002962
A_23_P25410:	PI16	NM_153370
A_24_P25081!	POF1B	NM_024921
A_23_P32011:	SRXN1	NM_080725
A_23_P82002	TEAD3	NM_003214
A_23_P21478{	SNX9	NM_016224
A_33_P32954:	ZBTB3	NM_024784
A_32_P16464{	SAFB	NM_002967
A_23_P70915	ORAI2	NM_032831

A_23_P13519[illegible]	TMEFF1	NM_003692
A_23_P21622[illegible]	EGR3	NM_004430
A_33_P32163[illegible]	KDM5C	NM_001146702
A_24_P11014[illegible]	C15orf51	NR_003260
A_33_P38050[illegible]	FNIP2	NM_020840
A_23_P13293[illegible]	SPCS3	NM_021928
A_33_P33064[illegible]	QRICH2	NM_032134
A_33_P34122[illegible]	ZSCAN2	NM_181877
A_23_P27846	PLIN4	NM_001080400
A_23_P20983[illegible]	MXD1	NM_002357
A_24_P33385[illegible]	SGIP1	NM_032291
A_24_P10727[illegible]	DRAP1	NM_006442
A_32_P23418[illegible]	HES5	NM_001010926
A_23_P91278	NFATC2	NM_173091
A_32_P89755	SSR1	NM_003144
A_24_P22434[illegible]	TTBK2	NM_173500
A_23_P10668[illegible]	EMP2	NM_001424
A_32_P50196[illegible]	RASA1	ENST00000274376
A_24_P2995	PUM2	NM_015317
A_33_P34109[illegible]	KLF1	NM_006563
A_24_P12642[illegible]	FAM188B	NM_032222
A_24_P20880[illegible]	RAB5A	NM_004162
A_32_P12418[illegible]	GRPEL2	NM_152407
A_32_P16627[illegible]	STX11	NM_003764
A_23_P39015[illegible]	AVIL	NM_006576
A_23_P78664	DDX39	NM_005804
A_24_P22274[illegible]	IL24	NM_006850
A_24_P92287[illegible]	KLC1	NM_182923
A_23_P27493	PPAN	NM_020230
A_24_P14837[illegible]	WASF2	ENST00000430629
A_24_P94113[illegible]	MIER2	AB033019
A_33_P33944[illegible]	TSPAN32	NM_139022
A_23_P20751[illegible]	PDK2	NM_002611
A_23_P33793[illegible]	FBLIM1	NM_017556
A_24_P17088[illegible]	C8orf33	NM_023080
A_32_P14166[illegible]	PBX2	NM_002586
A_24_P24377[illegible]	ZNF277	NM_021994
A_24_P19183[illegible]	SFRS12	NM_139168
A_33_P32872[illegible]	DPP4	NM_001935
A_33_P32403[illegible]	PITX3	NM_005029
A_23_P94633	RC3H2	NM_018835
A_23_P41796	MAP3K1	NM_005921
A_23_P13618[illegible]	UCN3	NM_053049
A_23_P15974[illegible]	BCOR	NM_017745
A_23_P16142[illegible]	ANKRD22	NM_144590
A_24_P68783	IL1F5	NM_012275
A_23_P23296	PKP1	NM_000299
A_33_P38623[illegible]	USP45	NM_001080481
A_32_P11934[illegible]	BICC1	ENST00000373886

A_23_P422018	SPRR4	NM_173080
A_23_P216188	IKBKB	NM_001556
A_23_P84289	LRRC20	NM_018205
A_23_P115608	ARHGAP21	NM_020824
A_33_P33825[illegible]	JRKL	NM_003772
A_23_P254254	SGSH	NM_000199
A_23_P133315	ZNF131	NM_003432
A_23_P13053[illegible]	CDC37	NM_007065
A_23_P138376	RAB18	NM_021252
A_24_P8350	PCNT	NM_006031
A_33_P335072	PPARG	NM_138711
A_24_P252705	TRDMT1	NM_004412
A_23_P203466	TAF10	NM_006284
A_23_P20463	RAD21	NM_006265
A_24_P374319	RAP2C	NM_021183
A_33_P33450[illegible]	POMC	NM_001035256
A_24_P409265	DENND4A	NM_005848
A_33_P32832[illegible]	YY2	NM_206923
A_23_P27994	TYROBP	NM_003332
A_23_P259135	NCOA6	NM_014071
A_23_P47559	C11orf58	NM_014267
A_24_P7157	RIMKLB	NM_020734
A_23_P23346	MLLT11	NM_006818
A_23_P312924	POU2AF1	NM_006235
A_24_P39299[illegible]	KRT16	NM_005557
A_32_P467914	FBXL20	AK024690
A_23_P322076	ZNF697	NM_001080470
A_23_P35030	CPSF3L	NM_017871
A_23_P13885	ATN1	NM_001007026
A_23_P56630	STAT1	NM_007315
A_23_P311358	ZNF282	NM_003575
A_23_P15567[illegible]	UBE2K	NM_005339
A_23_P20873[illegible]	FOXA3	NM_004497
A_24_P22799[illegible]	UBE2I	ENST00000397515
A_24_P74070	PARD6G	NM_032510
A_23_P202484	ZNF503	NM_032772
A_32_P80245	ZFP57	NM_001109809
A_24_P62708	PRKACB	NM_002731
A_23_P10172[illegible]	ZNF45	NM_003425
A_32_P130365	SCAND3	NM_052923
A_33_P340454	OBSCN	NM_001098623
A_33_P33371[illegible]	ABLIM2	NM_001130083
A_24_P26627[illegible]	POM121	NM_172020
A_33_P327306	NUP43	NM_198887
A_23_P165295	NFIX	NM_002501
A_23_P213592	RNF44	NM_014901
A_23_P140394	NFATC4	NM_004554
A_23_P94819	RPH3AL	NM_006987
A_23_P434968	KLF17	NM_173484

A_23_P21005	LRRTM4	NM_024993
A_32_P92528	MBD3L2	NM_144614
A_24_P61490	NKX2-1	NM_003317
A_23_P16929	DMRT1	NM_021951
A_24_P28811	PIK3C3	ENST00000262039
A_23_P15493	HIRA	NM_003325
A_32_P13400	XKR4	NM_052898
A_23_P30686	NR4A3	NM_173199
A_24_P23258	GRAMD4	NM_015124
A_24_P91625	KIAA1107	NM_015237
A_32_P98227	LDB3	NM_001171610
A_23_P40634	MFSD3	NM_138431
A_23_P13002	EPN3	NM_017957
A_33_P33327	ALMS1	NM_015120
A_33_P32566	CCDC101	NM_138414
A_23_P21706	CTPS	NM_001905
A_33_P35508	GATA2	NM_001145661
A_33_P32532	ZBTB48	NM_005341
A_23_P20465	KITLG	NM_000899
A_24_P37604	KLK4	NM_004917
A_32_P21179	USF2	AY007087
A_33_P34220	TBR1	NM_006593
A_32_P33191	tAKR	NR_026743
A_23_P36693	KRT6C	NM_173086
A_23_P15662	ZNF184	NM_007149
A_23_P12754	TRPC6	NM_004621
A_23_P12526	TP53BP2	NM_005426
A_23_P8175	PLAGL1	NM_006718
A_23_P31653	GPR25	NM_005298
A_33_P32945	PRKCB	NM_002738
A_23_P11864	EIF4E	NM_001968
A_23_P11417	TBC1D8B	NM_017752
A_23_P21479	SYNCRIP	NM_006372
A_23_P43079	INTS8	NM_017864
A_24_P26063	HIST1H1D	NM_005320
A_23_P26439	DBNDD1	NM_001042610
A_23_P8083	LY6G6C	NM_025261
A_23_P34932	XYLT1	NM_022166
A_23_P16735	C4orf29	NM_001039717
A_33_P33456	FLJ25328	NR_024335
A_23_P75299	LHPP	NM_022126
A_24_P23793	TCF23	NM_175769
A_33_P32795	UNQ353	AY358648
A_32_P45124	RIMBP3	NM_015672
A_23_P28012	ZNF682	NM_033196
A_23_P23221	GADD45A	NM_001924
A_23_P90453	KRTDAP	NM_207392
A_23_P4494	DSC2	NM_024422
A_23_P25080	MCM2	NM_004526

A_23_P42330	PCDH12	NM_016580
A_23_P15889	CBLN2	NM_182511
A_24_P28872	CASK	NM_003688
A_23_P63205	DR1	NM_001938
A_24_P15965	ZNF254	NM_203282
A_23_P43137	C14orf80	NM_001134875
A_23_P10020	HSBP1	NM_001537
A_23_P12555	PRICKLE3	ENST00000376317
A_23_P21043	GHRH	NM_021081
A_23_P32453	LCE3B	NM_178433
A_23_P41414	CASP6	NM_001226
A_23_P14275	EIF2AK2	NM_002759
A_33_P33813	VAX1	NM_199131
A_24_P19656	FXYD2	NM_021603
A_23_P38642	GTF2H3	NM_001516
A_24_P6494	TBC1D12	NM_015188
A_23_P20468	CLEC2D	NM_001004419
A_23_P21429	EFHC1	NM_018100
A_32_P39393	GLIS3	NM_001042413
A_23_P68059	PCGF1	NM_032673
A_24_P12639	TTC19	NM_017775
A_23_P13048	KRTAP5-9	NM_005553
A_24_P17625	GABPB1	NM_005254
A_33_P33551	BCL2	NM_000657
A_32_P82790	PRMT3	NM_005788
A_23_P50081	IMPA2	NM_014214
A_23_P16488	PNKP	NM_007254
A_23_P32791	ZIC3	NM_003413
A_24_P11783	PLEKHA5	AB051473
A_33_P33761	NT5DC4	BC041437
A_23_P32138	RNF19B	NM_153341
A_24_P30785	FBXL18	NM_024963
A_23_P35485	RS1	NM_000330
A_23_P20349	TRIM22	NM_006074
A_23_P76774	GSC	NM_173849
A_23_P13759	COL8A2	NM_005202
A_23_P15351	MED25	NM_030973
A_33_P33027	ZSCAN30	NM_001166012
A_23_P23586	CTNNBIP1	NM_020248
A_23_P42168	MDFI	NM_005586
A_23_P25705	C8orf55	NM_016647
A_23_P7473	SH3TC2	NM_024577
A_32_P14137	GATS	NM_178831
A_23_P39677	PCGF3	NM_006315
A_32_P22318	SUMO1P3	NR_002190
A_23_P56117	ZNF576	NM_024327
A_23_P16482	RNASEH2A	NM_006397
A_23_P67330	NR1H2	NM_007121
A_23_P11570	PCGF6	NM_001011663

A_23_P10413[illegible]	ZNF678	NM_178549
A_23_P34045[illegible]	COPS4	NM_016129
A_23_P40482[illegible]	KIAA1147	NM_001080392
A_23_P11339[illegible]	APLN	NM_017413
A_24_P77432	ROBO1	NM_133631
A_23_P10394[illegible]	DNAJC11	NM_018198
A_23_P70201	CHD1	NM_001270
A_32_P10882	C6orf52	NM_001145020
A_32_P43417[illegible]	SPNS2	NM_001124758
A_33_P33906[illegible]	ACSL6	NM_001009185
A_32_P20027[illegible]	CRYM	NM_001888
A_23_P21698[illegible]	RAB14	NM_016322
A_23_P20736[illegible]	STAT5A	NM_003152
A_23_P10183[illegible]	ETV2	NM_014209
A_23_P13693[illegible]	SYP	NM_003179
A_24_P19175	ZNF358	NM_018083
A_23_P23292	RXRG	NM_006917
A_23_P21051[illegible]	NCOA5	NM_020967
A_32_P48662[illegible]	IGSF22	NM_173588
A_23_P15951[illegible]	HADHA	NM_000182
A_33_P34202[illegible]	ENTPD8	NM_001033113
A_32_P78906[illegible]	FAM100B	NM_182565
A_23_P78825	ZNF790	NM_206894
A_23_P11390	VCY	NM_004679
A_24_P92367	YDJC	NM_001017964
A_32_P63257	LOC10013328[illegible]	XM_002342513
A_23_P22526	HEPH	NM_014799
A_32_P46139[illegible]	KDM6B	NM_001080424
A_24_P16764[illegible]	GCH1	NM_000161
A_23_P15663[illegible]	PGBD1	NM_032507
A_32_P36862	GPR155	NM_001033045
A_23_P11717[illegible]	PARP4	NM_006437
A_23_P15754[illegible]	NKX3-1	NM_006167
A_32_P19540[illegible]	FAM117B	NM_173511
A_23_P16689[illegible]	DNAJB11	NM_016306
A_23_P92320	NUP54	NM_017426
A_24_P39345[illegible]	CHRNA1	BC006314
A_24_P33066[illegible]	UBFD1	NM_019116
A_23_P58912	SLC35A1	NM_006416
A_23_P97309	CASP9	NM_001229
A_32_P20930	SDHC	NM_003001
A_23_P11068[illegible]	STC2	NM_003714
A_23_P85543	RNF2	NM_007212
A_33_P32220[illegible]	CBX3	NM_016587
A_24_P12818[illegible]	ZNF738	NR_027130
A_23_P37877	NARFL	NM_022493
A_24_P48057	IRX5	NM_005853
A_24_P27356[illegible]	C12orf35	NM_018169
A_24_P40671[illegible]	MLLT10	NM_004641

A_23_P21769[illegible]	SHOX	U89331
A_23_P96182	MYST3	NM_006766
A_33_P32982[illegible]	PPP4R4	NM_020958
A_33_P32493[illegible]	CHRD	NM_003741
A_23_P34071[illegible]	ONECUT1	NM_004498
A_24_P18329[illegible]	CSNK1A1	NM_001025105
A_32_P57140	JMJD1C	NM_032776
A_23_P16858[illegible]	POMZP3	NM_152992
A_23_P15084[illegible]	ZNF140	NM_003440
A_23_P37579	MORF4L1	NM_206839
A_33_P32874[illegible]	DYSFIP1	NM_001007533
A_23_P5131	ISYNA1	NM_016368
A_23_P17033[illegible]	ALDH4A1	NM_003748
A_23_P29592	ZNF639	NM_016331
A_23_P33589[illegible]	STK36	BC026158
A_33_P33265[illegible]	LRP5L	NM_182492
A_23_P67008	GATA6	ENST00000269216
A_23_P36076	SSRP1	NM_003146
A_23_P31537[illegible]	ATG16L1	AK000897
A_23_P25280[illegible]	WBP1	NM_012477
A_23_P20245[illegible]	ZNF22	NM_006963
A_32_P20629[illegible]	ZNF322A	NM_024639
A_24_P38019[illegible]	CTNND2	NM_001332
A_23_P55568	CLUL1	NM_014410
A_33_P33568[illegible]	LCE1E	NM_178353
A_23_P18392	PRKCI	NM_002740
A_24_P25293[illegible]	APOA4	NM_000482
A_32_P53928[illegible]	ZFC3H1	NM_144982
A_33_P34179[illegible]	VRK2	NM_006296
A_23_P16923[illegible]	ZNF510	NM_014930
A_23_P30428[illegible]	PSMC2	NM_002803
A_33_P32606[illegible]	STK3	NM_006281
A_23_P41061[illegible]	C12orf23	NM_152261
A_33_P32798[illegible]	PNRC1	NM_006813
A_23_P33695[illegible]	SLC8A2	NM_015063
A_33_P34047[illegible]	FMN1	NM_001103184
A_24_P24819	AGRN	NM_198576
A_23_P33523[illegible]	GAB1	NM_207123
A_23_P70371	PHF3	NM_015153
A_23_P25471[illegible]	UIMC1	NM_016290
A_23_P16015[illegible]	GALE	NM_000403
A_23_P41111[illegible]	CNTNAP1	U87223
A_23_P40049	CAD	NM_004341
A_23_P21737[illegible]	COL4A6	NM_033641
A_24_P34294[illegible]	CSPG5	NM_006574
A_24_P36550[illegible]	FERMT1	NM_017671
A_24_P41829[illegible]	CEP97	NM_024548
A_23_P16284[illegible]	SCEL	NM_144777
A_32_P53653[illegible]	ZFHX3	NM_006885

A_32_P15501	CTXN1	NM_206833
A_24_P12587	RIPK4	NM_020639
A_23_P51009	NDUFB3	NM_002491
A_32_P80254	EID2B	NM_152361
A_24_P13665	NFE2L3	NM_004289
A_23_P37251	SNAPC1	NM_003082
A_23_P12612	C1orf25	NM_030934
A_23_P14386	TMF1	NM_007114
A_23_P33889	PTPN1	NM_002827
A_24_P40001	SIGLEC8	NM_014442
A_33_P32645	HOXA11	NM_005523
A_23_P28042	ZNF737	NM_001159293
A_24_P25307	FAM119B	NM_206914
A_32_P37325	ZNF525	NR_003699
A_23_P76731	RAGE	NM_014226
A_23_P60306	TLR4	NM_138554
A_23_P40707	DNM2	NM_001005360
A_23_P35123	ZNF775	NM_173680
A_24_P92459	VEZF1	NM_007146
A_23_P40643	SRPK2	NM_182691
A_24_P20263	KLK15	NM_017509
A_23_P15916	AATK	NM_001080395
A_23_P23227	PKN2	NM_006256
A_23_P12884	GRK5	NM_005308
A_23_P75769	MS4A4A	NM_024021
A_23_P12160	SAP30	NM_003864
A_23_P20593	MEF2A	NM_001171894
A_23_P15193	TCF12	NM_207038
A_32_P98732	GCM1	NM_003643
A_24_P22229	ABCC4	NM_005845
A_23_P14253	MBD5	BC014534
A_23_P95924	ZNF562	NM_017656
A_23_P10132	ARHGEF1	NM_199002
A_23_P27668	SPIB	NM_003121
A_23_P14043	MYO5C	NM_018728
A_23_P94111	GTF2E2	NM_002095
A_24_P22230	FNIP1	NM_133372
A_23_P41137	APEH	NM_001640
A_24_P41471	NFYA	NM_002505
A_23_P11602	MED17	NM_004268
A_24_P17640	ZFP90	NM_133458
A_24_P12011	CFLAR	NM_003879
A_32_P53018	ZNF180	NM_013256
A_23_P41116	PER2	NM_022817
A_23_P11018	SC4MOL	NM_006745
A_32_P25357	CDH15	NM_004933
A_24_P71073	LOC10017093	NR_024054
A_23_P49810	HOXB7	NM_004502
A_23_P21388	NIPBL	NM_133433

A_33_P32820(	VANGL2	NM_020335
A_23_P35226(	BCL2	NM_000633
A_23_P77630	MAP1LC3B	NM_022818
A_23_P68740	AIRE	NM_000658
A_23_P12447(	CLCN3	NM_173872
A_23_P12705‹	PANK1	NM_148977
A_33_P32428:	PKN2	NM_006256
A_24_P40959!	MAPK8IP1	NM_005456
A_23_P15365:	FSD1	NM_024333
A_23_P12360{	JAK2	NM_004972
A_32_P19100‹	ATAD2B	NM_017552
A_33_P32193!	ZNF521	NM_015461
A_23_P10772‹	ZFP112	NM_013380
A_23_P14778(	RIMS2	NM_014677
A_23_P69683	SEC24B	NM_006323
A_24_P33779(	STK17A	NM_004760
A_23_P28120	SIX2	NM_016932
A_23_P35667	IKZF5	ENST00000368886
A_23_P1552	CTSC	NM_001814
A_23_P16434:	VAMP2	NM_014232
A_23_P37848{	ZNF511	NM_145806
A_33_P33749‹	KLF14	NM_138693
A_32_P32413	SETBP1	NM_015559
A_24_P41823!	RPS14	NM_001025071
A_24_P21186!	ARMC8	NM_015396
A_24_P97342	PROK2	NM_021935
A_23_P33072	MAF1	NM_032272
A_24_P94081!	SGPL1	NM_003901
A_23_P14134!	MPP3	NM_001932
A_33_P33797:	CCDC106	NM_013301
A_24_P28601:	UBE2K	NM_005339
A_23_P25720:	RNF146	NM_030963
A_32_P40889:	LOC150786	NM_001077637
A_24_P50759	TNF	NM_000594
A_23_P43150	ZHX1	NM_001017926
A_23_P11215!	EIF2C2	NM_012154
A_32_P15740:	SLC25A13	NM_014251
A_23_P11503(	IPO13	NM_014652
A_32_P53782!	SFMBT2	NM_001029880
A_32_P35337!	EIF2C2	BC054491
A_23_P33224(	ATOH1	NM_005172
A_32_P34175{	STIM1	NM_003156
A_24_P20025(	APEH	ENST00000296456
A_23_P21489:	AKAP12	NM_144497
A_33_P32674(	RFX3	NM_134428
A_23_P15550‹	OXSR1	NM_005109
A_23_P50259	ZNF526	NM_133444
A_24_P17107!	CREM	NM_183013
A_23_P39666(	TBC1D2B	NM_015079

A_32_P42925	MYL12B	NM_033546
A_23_P77286	C15orf29	NM_024713
A_23_P38110	CCDC74B	NM_207310
A_24_P10234	EPN3	NM_017957
A_32_P20743	ZNF845	NM_138374
A_23_P29034	IFNGR2	NM_005534
A_23_P21355	CSNK1A1	NM_001025105
A_23_P79426	CAB39	NM_016289
A_23_P31601	RHOJ	NM_020663
A_33_P33089	MIB2	NM_080875
A_23_P14999	PBLD	NM_022129
A_32_P11913	SEPP1	NM_005410
A_24_P31415	APP	NM_000484
A_23_P10344	MEAF6	NM_022756
A_33_P33321	RBP3	NM_002900
A_23_P30572	MIER1	NM_020948
A_23_P14879	UCHL5	NM_015984
A_23_P12580	ZCCHC12	NM_173798
A_33_P32500	NFATC4	NM_001136022
A_24_P37939	RPS14	ENST00000401695
A_24_P24710	AK3	NM_016282
A_32_P16750	DDAH1	NM_012137
A_23_P36129	DAK	NM_015533
A_23_P88947	LMF1	NM_022773
A_23_P33840	KDM1B	NM_153042
A_23_P40847	CHST2	NM_004267
A_23_P59616	GTF2IRD2	NM_173537
A_33_P34055	TMF1	NM_007114
A_32_P55241	SHISA2	NM_001007538
A_23_P91414	C20orf160	NM_080625
A_23_P33306	SMARCE1	NM_003079
A_23_P14672	ALG2	NM_033087
A_24_P40187	C9orf139	NM_207511
A_23_P93562	SESN1	NM_014454
A_24_P73766	MEG3	AK098378
A_23_P39160	ARRDC1	NM_152285
A_32_P39606	ZNF610	NM_173530
A_23_P32471	SYNJ1	NM_203446
A_24_P31417	ETS2	NM_005239
A_32_P42662	LHX1	NM_005568
A_23_P10066	SERPINF1	NM_002615
A_23_P40758	PEG3	NM_006210
A_24_P94301	LASS3	NM_178842
A_33_P33171	SEC24C	NM_004922
A_33_P33108	KRT40	NM_182497
A_32_P14255	ZNF562	NM_017656
A_23_P50349	TRIP10	NM_004240
A_23_P11372	HPRT1	NM_000194
A_23_P54834	NIP7	NM_016101

A_23_P31161	JMJD6	NM_001081461
A_33_P33667	MGAT4B	ENST00000292591
A_23_P37478	SH3KBP1	NM_001024666
A_23_P34628	GTF2B	NM_001514
A_23_P52417	NKX2-3	NM_145285
A_23_P57401	LZTR1	NM_006767
A_23_P67089	ZNF772	NM_001024596
A_33_P33442	HIST1H4A	NM_003538
A_23_P40406	VEZF1	NM_007146
A_33_P34286	DMWD	NM_004943
A_23_P63816	NRBF2	NM_030759
A_24_P92469	CDC42EP3	ENST00000295324
A_23_P25120	TUFM	NM_003321
A_23_P42146	EP400	NM_015409
A_24_P8098	NSD1	NM_022455
A_24_P19611	GTF2H5	NM_207118
A_32_P46651	C14orf4	NM_024496
A_23_P35673	ZDHHC8P	NR_003950
A_23_P25803	KDM3A	NM_018433
A_24_P33030	ARID4A	NM_002892
A_23_P64661	ARHGAP9	NM_032496
A_23_P21691	NR5A1	NM_004959
A_33_P32373	ZNF140	NM_003440
A_23_P10669	CHMP1A	NM_002768
A_23_P20775	MAFG	NM_032711
A_24_P12233	SYTL4	NM_080737
A_23_P16521	PRAM1	NM_032152
A_23_P21641	RAPGEF1	NM_198679
A_23_P42721	JMJD1C	NM_032776
A_24_P17758	GEN1	NM_182625
A_24_P32849	SOCS5	NM_144949
A_23_P10048	VKORC1	NM_206824
A_23_P43679	ZNF618	NM_133374
A_23_P16649	SGSM3	NM_015705
A_23_P15976	POF1B	NM_024921
A_33_P32997	KRT23	NM_015515
A_24_P41455	ARRDC3	NM_020801
A_24_P34408	REC8	NM_001048205
A_24_P13041	RTKN2	NM_145307
A_24_P20526	ESF1	NM_016649
A_23_P12083	TFIP11	NM_001008697
A_23_P10270	SNPH	NM_014723
A_23_P21432	RIPK1	NM_003804
A_23_P62115	TIMP1	NM_003254
A_24_P91559	COL4A6	BC005305
A_23_P39537	HIST1H4D	NM_003539
A_23_P21687	NR4A3	NM_006981
A_23_P70398	VEGFA	NM_001025370
A_23_P88184	BTBD7	NM_018167

A_23_P24269	C10orf88	NM_024942
A_24_P64618!	C5orf43	NM_001048249
A_23_P99027	PTPN11	NM_002834
A_24_P17073:	C1orf126	AK095631
A_23_P33722:	TFAP2A	NM_001032280
A_23_P953	CFHR2	NM_005666
A_33_P33661:	FLNA	NM_001110556
A_33_P33953:	ZNF420	NM_144689
A_33_P33254:	USP16	NM_001001992
A_32_P32920(	TDGF3	NR_002718
A_23_P13631:	RCOR3	NM_018254
A_23_P16176!	FXYD2	NM_021603
A_23_P21712(	EHMT1	NM_024757
A_23_P11300!	EFNA1	NM_004428
A_24_P26114	OXSR1	NM_005109
A_32_P87133:	C1orf126	NR_027136
A_33_P33640(	HR	NM_005144
A_23_P27414	ZNF497	NM_198458
A_24_P58620	GRAMD1B	NM_020716
A_24_P91705:	ZFP28	BC099903
A_33_P32521!	EZH2	NM_004456
A_23_P25443:	RFPL2	NM_006605
A_23_P43084:	HAPLN4	NM_023002
A_33_P32619:	ZNF789	NM_213603
A_23_P25313	BRI3BP	NM_080626
A_24_P34337:	ATP5J2	NM_001003714
A_33_P33009(	HOXC6	NM_153693
A_33_P32256!	ZNF516	NM_014643
A_24_P39361:	GNAO1	NM_138736
A_23_P14446!	PAPSS1	NM_005443
A_24_P12589:	PPM1F	NM_014634
A_24_P66493!	CNBP	NM_001127192
A_23_P21196!	ATP6V1A	NM_001690
A_23_P13485:	DOK2	NM_003974
A_23_P95172	RNF213	NM_020914
A_23_P27424	ZNF418	NM_133460
A_23_P20855:	LPHN1	NM_001008701
A_33_P32514(	C20orf141	NM_080739
A_33_P32106:	ASB13	NM_024701
A_23_P69491	RHOA	NM_001664
A_32_P74307:	ZFY	NM_001145276
A_32_P33823:	ZNF662	NM_207404
A_33_P34100:	PBXIP1	NM_020524
A_24_P70953(	MGC4859	BC002644
A_23_P13849!	PTPRE	NM_006504
A_24_P29942!	KLHDC10	NM_014997
A_33_P33672(	MMAB	NM_052845
A_23_P86252	PIGC	NM_002642
A_33_P33518!	HIST1H4L	NM_003546

A_32_P470728	GSX1	NM_145657
A_23_P26511	GDPD3	NM_024307
A_23_P408955	E2F2	NM_004091
A_24_P942030	VAMP4	NM_003762
A_23_P50834	ZNF714	NM_182515
A_24_P136619	CRISPLD2	BC007689
A_24_P131622	FAM107A	NM_007177
A_23_P76218	DCD	NM_053283
A_23_P36127	DAK	NM_015533
A_32_P210202	E2F7	NM_203394
A_23_P140830	ELMO3	NM_024712
A_23_P385254	CHD5	NM_015557
A_24_P320572	ZNF451	NM_015555
A_24_P49142	ZNF490	NM_020714
A_23_P26847	SOX9	NM_000346
A_24_P63019	IL1R2	NM_004633
A_23_P211039	ADAMTS1	NM_006988
A_23_P216649	ABCA1	NM_005502
A_24_P36285	ZNF224	NM_013398
A_23_P208259	ZNF347	NM_032584
A_24_P687302	LAMA3	NM_198129
A_33_P351925	NWD1	NM_001007525
A_23_P358714	KIAA1324	NM_020775
A_23_P76151	NAB2	NM_005967
A_23_P112429	RPL35	NM_007209
A_23_P27013	HOXB9	NM_024017
A_23_P121447	ARPM1	NM_032487
A_23_P404091	GRPEL2	NM_152407
A_23_P57404	LZTR1	NM_006767
A_24_P940524	ZFX	NM_003410
A_24_P110967	PCMTD1	NM_052937
A_23_P47848	ARL6IP4	NM_018694
A_32_P497073	CREB1	NM_134442
A_23_P384600	IPO13	NM_014652
A_23_P165479	FAHD2B	NM_199336
A_23_P136473	IRF2	NM_002199
A_23_P315122	EMX1	NM_004097
A_23_P8859	ZNF250	NM_021061
A_23_P147121	ZNF136	NM_003437
A_24_P348925	CCNK	NM_001099402
A_23_P204947	GJB2	NM_004004
A_23_P89145	ZNF83	NM_018300
A_33_P327218	MFSD9	NM_032718
A_23_P218047	KRT5	NM_000424
A_23_P71389	MTMR9	NM_015458
A_32_P132317	GPR155	NM_001033045
A_24_P303091	CXCL10	NM_001565
A_33_P332294	SPDYC	NM_001008778
A_32_P192437	CKS1B	NM_001826

A_23_P14766	OLFML1	NM_198474
A_23_P12046	ZFP64	NM_199427
A_24_P57058	ZNF542	NR_003127
A_23_P36874	HDAC10	NM_032019
A_24_P24537	SERPINB2	NM_002575
A_24_P25141	TMEM87B	NM_032824
A_23_P21083	MYT1	NM_004535
A_23_P37068	TRAF3	NM_145725
A_23_P32508	PTOV1	NM_017432
A_32_P48023	PTPRCAP	ENST00000326294
A_23_P39441	ZNF431	NM_133473
A_23_P12933	PKD1L2	NM_001076780
A_23_P16629	ABCG1	NM_207627
A_24_P13203	RNF14	NM_004290
A_23_P87257	MRPL17	NM_022061
A_23_P37907	MAPK3	NM_001040056
A_23_P5757	TPRKB	NM_016058
A_23_P33026	PIP5K1C	NM_012398
A_23_P60248	TXN	NM_003329
A_23_P65307	SLITRK6	NM_032229
A_33_P32247	IKZF5	NM_022466
A_24_P19197	SAP30L	NM_024632
A_23_P65442	IRF9	NM_006084
A_23_P88919	NME4	NM_005009
A_33_P33273	IRF3	NM_001197123
A_23_P14920	B4GALT2	NM_003780
A_24_P31061	SLC35A2	NM_001042498
A_33_P33506	HOPX	NM_001145460
A_32_P69158	RIMS2	NM_001100117
A_32_P31322	C3orf78	ENST00000477703
A_23_P9144	UHRF2	NM_152896
A_24_P18621	AEBP2	NM_153207
A_32_P10394	B4GALT4	NM_212543
A_23_P14371	APOBEC3G	NM_021822
A_24_P18151	ZNF646	NM_014699
A_23_P20079	NOTCH2	NM_024408
A_23_P34272	STARD13	NM_178006
A_23_P10832	NR2F6	NM_005234
A_23_P12921	FAH	NM_000137
A_33_P33220	ZNF554	NM_001102651
A_32_P32187	MAP3K13	ENST00000265026
A_23_P19657	LRP11	NM_032832
A_23_P15058	SCGB1A1	NM_003357
A_33_P33374	GNAL	NM_002071
A_23_P11025	ZNF185	NM_007150
A_24_P37945	NOL9	NM_024654
A_23_P82722	GATA4	NM_002052
A_32_P87959	GSK3B	NM_001146156
A_23_P32204	SMG5	NM_015327

A_23_P41541	PRMT10	NM_138364
A_23_P39050	ZNF823	NM_001080493
A_33_P33693	GPX3	NM_002084
A_23_P39309	PSG9	NM_002784
A_24_P31221	ZNF277	NM_021994
A_23_P14713	CDH15	NM_004933
A_23_P48384	VPS36	NM_016075
A_33_P32586	NYNRIN	NM_025081
A_24_P13251	IKBKB	NM_001556
A_33_P33939	PKP1	NM_000299
A_23_P38752	ZBTB40	NM_001083621
A_23_P67928	IL8RA	NM_000634
A_23_P21647	ZBTB5	NM_014872
A_23_P84910	ZNF157	NM_003446
A_23_P10761	RAB27B	NM_004163
A_23_P36508	FOXL1	NM_005250
A_23_P15685	PECI	NM_206836
A_23_P13212	SIK1	NM_173354
A_23_P14509	PLA2G7	NM_005084
A_23_P13619	TBC1D19	NM_018317
A_24_P17640	ZFP90	NM_133458
A_23_P43399	SPG7	NM_003119
A_32_P36587	LAMA2	NM_001079823
A_23_P42021	CACNG8	NM_031895
A_23_P42290	ZNF678	NM_178549
A_32_P38884	KLF8	NM_007250
A_23_P21322	PHOX2B	NM_003924
A_32_P14570	NDUFV2	NM_021074
A_23_P12004	BAZ2B	NM_013450
A_23_P49865	YBX2	NM_015982
A_33_P38355	POU2F2	NM_002698
A_23_P21107	OLIG2	NM_005806
A_23_P40587	C9orf72	NM_145005
A_24_P48069	DOK4	NM_018110
A_24_P39897	COQ7	NM_016138
A_23_P66767	GGT6	NM_153338
A_23_P31866	MED30	NM_080651
A_24_P12630	ZNF500	NM_021646
A_24_P12800	ZNF395	NM_018660
A_23_P89327	ZNF286A	NM_020652
A_23_P36701	TCTEX1D1	NM_152665
A_23_P41255	C18orf25	NM_145055
A_23_P10147	ZNF442	NM_030824
A_23_P4604	ZNF611	NM_030972
A_23_P84952	TFE3	NM_006521
A_23_P32279	BARX1	NM_021570
A_23_P79015	SCN1B	NM_199037
A_23_P10858	ANKLE2	NM_015114
A_23_P42009	DENND4A	NM_001144823

A_23_P15824	TOP3A	NM_004618
A_23_P12455	MXD3	NM_031300
A_24_P20065	C6orf62	NM_030939
A_23_P21136	GNAZ	NM_002073
A_32_P16431	MORF4L1	NM_206839
A_23_P14283	DCTN1	NM_004082
A_23_P18939	RASA1	NM_002890
A_23_P13820	SLCO1A2	NM_134431
A_23_P33015	POLR2L	NM_021128
A_23_P27810	ZNF607	NM_032689
A_24_P76459	PTP4A2	NM_080392
A_23_P25966	ZKSCAN5	NM_014569
A_24_P15871	DTX4	NM_015177
A_24_P27492	ZNF703	NM_025069
A_24_P24416	PI4K2A	NM_018425
A_23_P34027	LMX1A	NM_177398
A_24_P41654	MEX3C	NM_016626
A_23_P11739	MIXL1	NM_031944
A_23_P35243	RGS12	NM_002926
A_23_P53630	RNF10	NM_014868
A_23_P90845	TIA1	NM_022173
A_23_P97623	RRAGC	NM_022157
A_33_P32896	SERPINA1	BX248257
A_24_P49106	TCEAL7	NM_152278
A_23_P16884	FBXO16	NM_172366
A_24_P39248	MYPOP	NM_001012643
A_23_P34690	CACNA2D2	NM_001005505
A_23_P16115	ZNF438	NM_182755
A_23_P81392	WWC1	NM_015238
A_33_P32534	PQLC3	NM_152391
A_33_P33074	KRTAP9-8	NM_031963
A_23_P13052	CDC37	NM_007065
A_33_P32568	C14orf80	NM_001134875
A_23_P41066	RASSF1	NM_170713
A_32_P10168	FAM3C	NM_014888
