# Supplementary material for: GRHL3 binding and enhancers rearrange as epidermal keratinocytes transition between functional states
Source: PLoS Genet. 2017 Apr 26;13(4):e1006745. doi: 10.1371/journal.pgen.1006745 (PMC5425218; doi:10.1371/journal.pgen.1006745)
Supplement: S2 Table — (PDF) [file pgen.1006745.s015.pdf]

TF with SE	TF without SE
ATF5	TCF4
RUNX1	TCF7L2
STAT6*	XBP1
PBX2	RUNX2
RARG	SMAD4
RBL1	SOX11
RELB	SP1
TP63	SP3
GRHL3	STAT1
NR3C1	STAT3
NOTCH3	PRDM1
LRRFIP1	OVOL1
JUND*	OVOL2
GRHL1	PBX1
GRHL2	KLF4
GABPB2	JUN
FOXP1	FOXN1
FOSL2*	FOXD1
FOSL1	E2F1
FOS	CREB5
ETS1*	BRIP1
CEBPG	ATF7
	ATF4
	ATF3
	ATF2
	SOX6
	RORA
	FOXN2
* in NHEK-M	JUNB

Table S2. List of transcription factors selected for siRNA and their overlap with SE in NHEK-D or NHEK-M (indicated by "*")
