# Supplementary material for: GRHL3 binding and enhancers rearrange as epidermal keratinocytes transition between functional states
Source: PLoS Genet. 2017 Apr 26;13(4):e1006745. doi: 10.1371/journal.pgen.1006745 (PMC5425218; doi:10.1371/journal.pgen.1006745)
Supplement: S3 Table — (PDF) [file pgen.1006745.s016.pdf]

Table S3. List of all transcriptional regulators that overlap NHEK-D SE.

SE chromosome	SE start	SE end	SE number	Transcription Factor	strand
chr1	839621	1027669	region_37	NM_021170	+
chr1	839621	1027669	region_37	NM_00114246	+
chr1	2304339	2528305	region_14	NM_00101092	+
chr1	3585621	3673180	region_281	NM_00120418	+
chr1	3585621	3673180	region_281	NM_00120418	+
chr1	3585621	3673180	region_281	NM_00120418	+
chr1	3585621	3673180	region_281	NM_00120418	+
chr1	3585621	3673180	region_281	NM_005427	+
chr1	3585621	3673180	region_281	NM_00120418	+
chr1	3585621	3673180	region_281	NM_00120418	+
chr1	3585621	3673180	region_281	NM_00112624	+
chr1	3585621	3673180	region_281	NM_00112624	+
chr1	3585621	3673180	region_281	NM_00112624	+
chr1	3585621	3673180	region_281	NM_00120419	+
chr1	3585621	3673180	region_281	NM_00120419	+
chr1	3585621	3673180	region_281	NM_00120419	+
chr1	6258950	6456872	region_128	NM_00102459	+
chr1	6473099	6580270	region_80	NM_019089	+
chr1	6639398	6694371	region_259	NM_00119575	+
chr1	6639398	6694371	region_259	NM_00127864	+
chr1	6639398	6694371	region_259	NM_00127864	+
chr1	6639398	6694371	region_259	NM_005341	+
chr1	6639398	6694371	region_259	NM_00119575	+
chr1	6639398	6694371	region_259	NM_138350	+
chr1	16211385	16302707	region_326	NM_00124288	+
chr1	16211385	16302707	region_326	NM_003443	+
chr1	16211385	16302707	region_326	NM_00128760	+
chr1	16211385	16302707	region_326	NM_00128760	+
chr1	24641874	24720611	region_617	NM_198173	+
chr1	24641874	24720611	region_617	NM_00119501	+
chr1	24641874	24720611	region_617	NM_198174	+
chr1	24641874	24720611	region_617	NM_021180	+
chr1	27172274	27248946	region_90	NM_021969	+
chr1	38454654	38515318	region_437	NM_002699	+
chr1	41917562	42003223	region_687	NM_024503	+
chr1	41917562	42003223	region_687	NM_00112771	+
chr1	153913764	153963861	region_438	NM_130898	+
chr1	153913764	153963861	region_438	NR_045658	+
chr1	153913764	153963861	region_438	NM_00125598	+
chr1	153913764	153963861	region_438	NM_00125597	+
chr1	153913764	153963861	region_438	NM_00125597	+
chr1	153913764	153963861	region_438	NM_00125598	+
chr1	154883217	155066863	region_33	NR_045515	+
chr1	154883217	155066863	region_33	NR_049765	+
chr1	154883217	155066863	region_33	NM_00125240	+
chr1	154883217	155066863	region_33	NM_00125645	+
chr1	154883217	155066863	region_33	NR_046206	+

chr1	156459061	156480683	region_695	NM_00127162	+
chr1	156459061	156480683	region_695	NM_005920	+
chr1	249105947	249168002	region_536	NM_00119332	+
chr1	249105947	249168002	region_536	NM_00113603	+
chr1	249105947	249168002	region_536	NM_024836	+
chr1	249105947	249168002	region_536	NM_017865	+
chr10	3761312	3829233	region_421	NM_001300	+
chr10	3761312	3829233	region_421	NR_027653	+
chr10	3761312	3829233	region_421	NM_00116012	+
chr10	3761312	3829233	region_421	NM_00116012	+
chr10	80807067	81111457	region_36	NM_020338	+
chr10	104128149	104195680	region_517	NM_00128872	+
chr10	104128149	104195680	region_517	NM_002502	+
chr10	104128149	104195680	region_517	NM_00107749	+
chr10	104128149	104195680	region_517	NM_00126140	+
chr10	135009090	135171955	region_120	NM_014468	+
chr11	350070	658866	region_13	NM_004029	+
chr11	350070	658866	region_13	NM_00129363	+
chr11	350070	658866	region_13	NM_004031	+
chr11	350070	658866	region_13	NM_001572	+
chr11	350070	658866	region_13	NM_021008	+
chr11	692151	911757	region_12	NM_00129363	+
chr11	692151	911757	region_12	NM_021008	+
chr11	47398466	47448904	region_538	NM_00108054	+
chr11	47398466	47448904	region_538	NM_003120	+
chr11	57042986	57137855	region_351	NM_003146	+
chr11	62303236	62400156	region_61	NM_004739	+
chr11	62469934	62575083	region_310	NM_024784	+
chr11	63949268	64160649	region_6	NM_00128245	+
chr11	63949268	64160649	region_6	NM_00128245	+
chr11	63949268	64160649	region_6	NM_004451	+
chr11	65291942	65431449	region_78	NM_021975	+
chr11	65291942	65431449	region_78	NM_00124398	+
chr11	65291942	65431449	region_78	NM_00114513	+
chr11	65291942	65431449	region_78	NM_00124398	+
chr11	65617741	65692778	region_59	NM_00130085	+
chr11	65617741	65692778	region_59	NM_005438	+
chr11	65617741	65692778	region_59	NM_00130085	+
chr11	65617741	65692778	region_59	NR_125339	+
chr11	65617741	65692778	region_59	NM_00130085	+
chr11	65617741	65692778	region_59	NM_00130084	+
chr11	118955117	118992799	region_753	NM_00124325	+
chr11	118955117	118992799	region_753	NM_015517	+
chr11	118955117	118992799	region_753	NM_198971	+
chr12	48099229	48277110	region_81	NM_000376	+
chr12	48099229	48277110	region_81	NM_00101753	+
chr12	48099229	48277110	region_81	NM_00101753	+
chr12	53606900	53647355	region_525	NM_00104272	+
chr12	53606900	53647355	region_525	NM_000966	+

chr12	53606900	53647355	region_525	NM_00124373	+
chr12	53606900	53647355	region_525	NM_00124373	+
chr12	53606900	53647355	region_525	NM_00124373	+
chr12	54321475	54432522	region_123	NM_014212	+
chr12	54321475	54432522	region_123	NM_173860	+
chr12	54321475	54432522	region_123	NM_017410	+
chr12	54321475	54432522	region_123	NM_018953	+
chr12	54321475	54432522	region_123	NM_006897	+
chr12	54321475	54432522	region_123	NM_017409	+
chr12	54321475	54432522	region_123	NM_004503	+
chr12	54321475	54432522	region_123	NR_003084	+
chr12	54321475	54432522	region_123	NM_022658	+
chr12	54321475	54432522	region_123	NM_153693	+
chr12	54321475	54432522	region_123	NM_014620	+
chr12	57022890	57083355	region_646	NM_013449	+
chr12	57022890	57083355	region_646	NM_00130090	+
chr12	57466986	57638402	region_122	NM_00117808	+
chr12	57466986	57638402	region_122	NM_00117808	+
chr12	57466986	57638402	region_122	NR_033659	+
chr12	57466986	57638402	region_122	NM_00117807	+
chr12	57466986	57638402	region_122	NM_00117807	+
chr12	57466986	57638402	region_122	NM_003153	+
chr12	96587074	96658569	region_676	NM_00130351	+
chr12	96587074	96658569	region_676	NM_005230	+
chr12	122425091	122525516	region_406	NM_014938	+
chr12	124779420	125078079	region_54	NM_00107726	+
chr12	124779420	125078079	region_54	NM_00120665	+
chr12	124779420	125078079	region_54	NM_006312	+
chr14	23754104	23791784	region_674	NM_020834	+
chr14	24601973	24809200	region_25	NM_006084	+
chr14	99640923	99741571	region_313	NM_00128223	+
chr14	99640923	99741571	region_313	NM_00128223	+
chr14	99640923	99741571	region_313	NM_022898	+
chr14	99640923	99741571	region_313	NM_138576	+
chr14	102948134	103071461	region_175	NM_015156	+
chr14	105235079	105293945	region_596	NM_00113760	+
chr14	105760450	105978473	region_135	NM_004689	+
chr14	105760450	105978473	region_135	NM_00120325	+
chr15	31617614	31668378	region_400	NM_015995	+
chr15	31617614	31668378	region_400	NM_00130246	+
chr15	67356612	67443591	region_283	NM_00114510	+
chr15	67356612	67443591	region_283	NM_005902	+
chr15	67356612	67443591	region_283	NM_00114510	+
chr15	96864803	96911655	region_723	NM_021005	+
chr15	96864803	96911655	region_723	NM_00114515	+
chr15	96864803	96911655	region_723	NM_00114515	+
chr15	96864803	96911655	region_723	NM_00114515	+
chr16	1015110	1275376	region_424	NM_014587	+
chr16	2171885	2336327	region_62	NM_00128877	+

chr16	2171885	2336327	region_62	NM_004424	+
chr16	2171885	2336327	region_62	NM_00128877	+
chr16	3139327	3241965	region_287	NM_032805	+
chr16	3139327	3241965	region_287	NM_00128241	+
chr16	3139327	3241965	region_287	NM_00128241	+
chr16	3139327	3241965	region_287	NM_003456	+
chr16	3139327	3241965	region_287	NM_00104242	+
chr16	3139327	3241965	region_287	NM_00113465	+
chr16	3139327	3241965	region_287	NR_104432	+
chr16	3139327	3241965	region_287	NM_004220	+
chr16	3139327	3241965	region_287	NM_00127815	+
chr16	4338292	4478843	region_149	NM_00131891	+
chr16	4338292	4478843	region_149	NM_032575	+
chr16	29815965	29837112	region_558	NM_00127627	+
chr16	29815965	29837112	region_558	NM_00127627	+
chr16	29815965	29837112	region_558	NR_074080	+
chr16	29815965	29837112	region_558	NM_002383	+
chr16	29815965	29837112	region_558	NM_00104253	+
chr16	30064139	30134972	region_232	NM_004608	+
chr16	30345855	30390920	region_621	NM_00121490	+
chr16	30345855	30390920	region_621	NM_00121490	+
chr16	30725923	30835436	region_428	NM_00108041	+
chr16	31043926	31160089	region_162	NM_00117266	+
chr16	31043926	31160089	region_162	NM_024706	+
chr16	31043926	31160089	region_162	NM_014699	+
chr16	31043926	31160089	region_162	NM_00117267	+
chr16	31043926	31160089	region_162	NM_00117266	+
chr16	67175437	67283379	region_133	NM_001538	+
chr16	67175437	67283379	region_133	NM_001950	+
chr16	67175437	67283379	region_133	NM_00104066	+
chr16	67555159	67598039	region_581	NM_00119102	+
chr16	67555159	67598039	region_581	NM_006565	+
chr16	67854838	68066748	region_83	NM_020457	+
chr16	73053238	73116580	region_719	NM_006885	+
chr16	73053238	73116580	region_719	NM_00116476	+
chr16	88479377	88604152	region_339	NM_153813	+
chr16	88479377	88604152	region_339	NM_00112746	+
chr16	88696950	89081667	region_24	NM_178310	+
chr16	89752698	89812615	region_387	NR_110129	+
chr16	89752698	89812615	region_387	NM_152287	+
chr16	89752698	89812615	region_387	NR_110128	+
chr16	89752698	89812615	region_387	NR_110126	+
chr16	89752698	89812615	region_387	NR_110122	+
chr16	89752698	89812615	region_387	NM_00111352	+
chr17	1880532	1996951	region_413	NM_00109820	+
chr17	1880532	1996951	region_413	NM_006497	+
chr17	2256311	2337694	region_239	NM_020310	+
chr17	4686029	4873821	region_34	NM_015099	+
chr17	4686029	4873821	region_34	NM_00117116	+

chr17	4686029	4873821	region_34	NM_00117116	+
chr17	4686029	4873821	region_34	NM_00117116	+
chr17	7184107	7234139	region_417	NM_015982	+
chr17	7283862	7400842	region_119	NM_00112883	+
chr17	7283862	7400842	region_119	NM_020899	+
chr17	7452034	7496887	region_154	NM_006942	+
chr17	8006555	8135971	region_111	NM_032580	+
chr17	8006555	8135971	region_111	NM_00116596	+
chr17	17564394	17781000	region_29	NM_004176	+
chr17	17564394	17781000	region_29	NM_00100529	+
chr17	37752773	37912147	region_190	NM_006160	+
chr17	38244424	38279951	region_552	NM_00119091	+
chr17	38244424	38279951	region_552	NM_199334	+
chr17	38244424	38279951	region_552	NM_00119091	+
chr17	38244424	38279951	region_552	NM_003250	+
chr17	38244424	38279951	region_552	NM_021724	+
chr17	38463455	38532356	region_411	NM_000964	+
chr17	38463455	38532356	region_411	NM_00114530	+
chr17	38463455	38532356	region_411	NM_00114530	+
chr17	38463455	38532356	region_411	NM_00102480	+
chr17	40670200	40740671	region_330	NM_198204	+
chr17	40670200	40740671	region_330	NM_198205	+
chr17	40670200	40740671	region_330	NM_170607	+
chr17	42247773	42300108	region_205	NM_014233	+
chr17	42247773	42300108	region_205	NR_045058	+
chr17	42247773	42300108	region_205	NM_00107668	+
chr17	42247773	42300108	region_205	NM_00107668	+
chr17	45882114	45993569	region_653	NM_003110	+
chr17	45882114	45993569	region_653	NM_00125824	+
chr17	45882114	45993569	region_653	NM_199262	+
chr17	46079843	46157648	region_294	NM_003204	+
chr17	79848232	80115977	region_22	NM_032711	+
chr17	79848232	80115977	region_22	NM_002359	+
chr18	3446998	3457661	region_521	NM_173207	+
chr18	3446998	3457661	region_521	NM_00127868	+
chr18	3446998	3457661	region_521	NM_174886	+
chr18	3446998	3457661	region_521	NM_003244	+
chr18	3446998	3457661	region_521	NM_00127868	+
chr18	3446998	3457661	region_521	NM_00127868	+
chr18	3446998	3457661	region_521	NM_173209	+
chr18	3446998	3457661	region_521	NM_173208	+
chr18	3446998	3457661	region_521	NM_173210	+
chr18	3446998	3457661	region_521	NM_170695	+
chr18	3446998	3457661	region_521	NM_173211	+
chr18	46441350	46486983	region_511	NM_005904	+
chr18	46441350	46486983	region_511	NM_00119082	+
chr18	46441350	46486983	region_511	NM_00119082	+
chr18	46441350	46486983	region_511	NM_00119082	+
chr19	571081	959937	region_10	NM_005224	+

chr19	1379295	1993229	region_2	NM_00108048	+
chr19	1379295	1993229	region_2	NM_00113613	+
chr19	1379295	1993229	region_2	NM_003200	+
chr19	1379295	1993229	region_2	NM_00128145	+
chr19	1379295	1993229	region_2	NM_00128145	+
chr19	1379295	1993229	region_2	NM_031918	+
chr19	3336709	3405369	region_457	NM_005597	+
chr19	3336709	3405369	region_457	NM_00124500	+
chr19	3336709	3405369	region_457	NM_205843	+
chr19	3336709	3405369	region_457	NM_00124500	+
chr19	3336709	3405369	region_457	NM_00124500	+
chr19	3433691	3558518	region_297	NM_005597	+
chr19	3433691	3558518	region_297	NM_00124500	+
chr19	3433691	3558518	region_297	NM_205843	+
chr19	3433691	3558518	region_297	NM_00124500	+
chr19	3433691	3558518	region_297	NM_00124500	+
chr19	3572060	3819062	region_262	NM_00131907	+
chr19	3572060	3819062	region_262	NM_006339	+
chr19	3572060	3819062	region_262	NM_032753	+
chr19	3950416	4131976	region_58	NM_00131799	+
chr19	3950416	4131976	region_58	NM_015897	+
chr19	3950416	4131976	region_58	NM_015898	+
chr19	6048500	6111066	region_564	NM_000635	+
chr19	6048500	6111066	region_564	NM_134433	+
chr19	7440727	7635065	region_372	NM_018083	+
chr19	10361916	10445999	region_632	NM_00110316	+
chr19	12883260	12918151	region_113	NM_002229	+
chr19	13094237	13172343	region_529	NM_00127104	+
chr19	13094237	13172343	region_529	NM_002501	+
chr19	13094237	13172343	region_529	NM_00127104	+
chr19	13189649	13284124	region_121	NM_005583	+
chr19	13189649	13284124	region_121	NM_00127104	+
chr19	13189649	13284124	region_121	NM_002501	+
chr19	13189649	13284124	region_121	NM_00127104	+
chr19	17294251	17379824	region_498	NM_005234	+
chr19	18384996	18455589	region_430	NM_005354	+
chr19	18384996	18455589	region_430	NM_00128696	+
chr19	19690640	19780666	region_635	NM_033204	+
chr19	19690640	19780666	region_635	NM_025245	+
chr19	19690640	19780666	region_635	NR_038198	+
chr19	19690640	19780666	region_635	NM_00130094	+
chr19	35738446	35768852	region_691	NM_207291	+
chr19	35738446	35768852	region_691	NM_003367	+
chr19	36206743	36282987	region_471	NM_00131690	+
chr19	36206743	36282987	region_471	NM_00131690	+
chr19	36206743	36282987	region_471	NM_014383	+
chr19	36591549	36652025	region_608	NM_00130275	+
chr19	42554871	42639236	region_212	NM_00120702	+
chr19	42554871	42639236	region_212	NM_022752	+

chr19	42554871	42639236	region_212	NM_00120702	+
chr19	42554871	42639236	region_212	NM_00124799	+
chr19	42554871	42639236	region_212	NM_002698	+
chr19	42695724	42729938	region_554	NM_00131403	+
chr19	42695724	42729938	region_554	NM_133444	+
chr19	42744114	42830703	region_65	NM_015125	+
chr19	42744114	42830703	region_65	NM_006494	+
chr19	42744114	42830703	region_65	NM_00130103	+
chr19	42744114	42830703	region_65	NM_00130840	+
chr19	42744114	42830703	region_65	NM_00131265	+
chr19	42744114	42830703	region_65	NM_00130481	+
chr19	45535817	45695737	region_193	NM_006509	+
chr19	45535817	45695737	region_193	NM_145288	+
chr19	45872390	46033240	region_47	NM_006732	+
chr19	45872390	46033240	region_47	NM_00111417	+
chr19	46252478	46330558	region_480	NM_175875	+
chr19	49092048	49184167	region_664	NM_001352	+
chr19	49362091	49405003	region_730	NM_003323	+
chr19	50135300	50204919	region_374	NM_00119712	+
chr19	50135300	50204919	region_374	NM_00119712	+
chr19	50135300	50204919	region_374	NM_00119712	+
chr19	50135300	50204919	region_374	NM_001571	+
chr19	50135300	50204919	region_374	NR_045568	+
chr19	50135300	50204919	region_374	NM_00119712	+
chr19	50135300	50204919	region_374	NM_00119712	+
chr19	50135300	50204919	region_374	NM_00119712	+
chr19	50135300	50204919	region_374	NM_00119712	+
chr19	50345442	50441526	region_200	NM_012068	+
chr19	50345442	50441526	region_200	NM_00129074	+
chr19	50345442	50441526	region_200	NM_00119364	+
chr19	55847977	56015431	region_155	NM_033113	+
chr19	56089842	56203705	region_118	NM_00116342	+
chr19	56089842	56203705	region_118	NM_207115	+
chr19	56089842	56203705	region_118	NM_203374	+
chr19	56089842	56203705	region_118	NM_152600	+
chr19	56089842	56203705	region_118	NM_153219	+
chr19	56089842	56203705	region_118	NM_032836	+
chr19	56089842	56203705	region_118	NM_00119560	+
chr19	56089842	56203705	region_118	NM_016202	+
chr19	56089842	56203705	region_118	NM_016535	+
chr19	58858446	58921053	region_278	NM_00131800	+
chr19	58858446	58921053	region_278	NM_198458	+
chr19	58858446	58921053	region_278	NM_00120700	+
chr19	58858446	58921053	region_278	NM_173548	+
chr19	58858446	58921053	region_278	NR_049780	+
chr19	58858446	58921053	region_278	NM_138466	+
chr19	58858446	58921053	region_278	NR_134461	+
chr19	59049600	59094512	region_317	NM_198055	+
chr19	59049600	59094512	region_317	NM_00131698	+

chr19	59049600	59094512	region_317	NM_00131698	+
chr19	59049600	59094512	region_317	NM_00126703	+
chr19	59049600	59094512	region_317	NM_003422	+
chr2	27543768	27608001	region_639	NM_144631	+
chr2	27543768	27608001	region_639	NM_00120145	+
chr2	28542445	28643085	region_227	NM_005253	+
chr2	74634241	74758165	region_69	NM_00100981	+
chr2	74634241	74758165	region_69	NM_016170	+
chr2	74634241	74758165	region_69	NM_00128243	+
chr2	85464367	85555712	region_547	NM_031283	+
chr2	172947919	172986856	region_528	NM_00103849	+
chr2	172947919	172986856	region_528	NM_004405	+
chr2	172947919	172986856	region_528	NM_178120	+
chr2	178099933	178133519	region_612	NM_00131390	+
chr2	178099933	178133519	region_612	NM_00114541	+
chr2	178099933	178133519	region_612	NM_00131390	+
chr2	178099933	178133519	region_612	NM_00131390	+
chr2	178099933	178133519	region_612	NM_00131390	+
chr2	178099933	178133519	region_612	NM_00114541	+
chr2	178099933	178133519	region_612	NM_00131390	+
chr2	178099933	178133519	region_612	NM_006164	+
chr2	242549192	242642860	region_388	NM_015963	+
chr2	242549192	242642860	region_388	NM_00116435	+
chr20	30124132	30200744	region_491	NM_181353	+
chr20	30124132	30200744	region_491	NM_002165	+
chr20	44509780	44601845	region_370	NM_022095	+
chr20	48719960	48811217	region_172	NM_005194	+
chr20	48719960	48811217	region_172	NM_00128587	+
chr20	48719960	48811217	region_172	NM_00128587	+
chr20	60860694	61091163	region_28	NM_080473	+
chr20	61318109	61493692	region_221	NM_006602	+
chr20	61318109	61493692	region_221	NM_00130172	+
chr20	62149121	62225987	region_234	NM_012384	+
chr20	62248881	62298798	region_609	NM_012384	+
chr20	62449371	62612977	region_88	NM_020713	+
chr20	62653239	62751953	region_628	NM_018419	+
chr21	36164106	36263941	region_187	NM_001754	+
chr21	36164106	36263941	region_187	NM_00100189	+
chr21	36164106	36263941	region_187	NM_00112260	+
chr21	40123662	40229180	region_463	NM_00125629	+
chr21	40123662	40229180	region_463	NM_005239	+
chr21	45681682	45771251	region_513	NM_000383	+
chr22	19667977	19812623	region_355	NM_005992	+
chr22	19667977	19812623	region_355	NM_080647	+
chr22	19667977	19812623	region_355	NM_080646	+
chr22	21299884	21410171	region_225	NM_030573	+
chr22	21299884	21410171	region_225	NM_00100869	+
chr22	38597274	38643132	region_258	NM_00116157	+
chr22	38597274	38643132	region_258	NM_00116157	+

chr22	38597274	38643132	region_258	NM_00116157	+
chr22	38597274	38643132	region_258	NM_012323	+
chr22	41592878	41779030	region_160	NM_00114539	+
chr22	41592878	41779030	region_160	NM_003216	+
chr22	46390948	46547805	region_7	NM_00100192	+
chr22	46390948	46547805	region_7	NM_005036	+
chr22	50147608	50253264	region_444	NM_014838	+
chr3	5019077	5068851	region_345	NM_003670	+
chr3	141085122	141160821	region_689	NM_00108041	+
chr3	150125352	150130666	region_699	NM_00130326	+
chr3	150125352	150130666	region_699	NR_130136	+
chr3	150125352	150130666	region_699	NM_014779	+
chr4	1279680	1410429	region_103	NM_00129007	+
chr4	6987992	7078132	region_248	NM_152293	+
chr5	415737	547579	region_484	NM_020731	+
chr5	415737	547579	region_484	NM_00124241	+
chr6	10398371	10466652	region_116	NM_00103228	+
chr6	10398371	10466652	region_116	NM_00104242	+
chr6	10398371	10466652	region_116	NM_003220	+
chr6	32116077	32190416	region_600	NM_002586	+
chr6	33159907	33185989	region_506	NM_00127040	+
chr6	33159907	33185989	region_506	NM_021976	+
chr6	33159907	33185989	region_506	NM_00129198	+
chr6	34191063	34217780	region_638	NM_00131908	+
chr6	34191063	34217780	region_638	NM_145901	+
chr6	34191063	34217780	region_638	NM_00131907	+
chr6	34191063	34217780	region_638	NM_00131907	+
chr6	34191063	34217780	region_638	NM_00131908	+
chr6	34191063	34217780	region_638	NM_00131908	+
chr6	34191063	34217780	region_638	NM_002131	+
chr6	34191063	34217780	region_638	NM_145899	+
chr6	34191063	34217780	region_638	NM_145903	+
chr6	34191063	34217780	region_638	NM_00131907	+
chr6	34191063	34217780	region_638	NM_145902	+
chr6	34191063	34217780	region_638	NM_145905	+
chr6	35410460	35480065	region_218	NM_00128939	+
chr6	35410460	35480065	region_218	NM_003214	+
chr6	35410460	35480065	region_218	NM_003322	+
chr6	43137907	43197614	region_431	NM_003131	+
chr6	43137907	43197614	region_431	NM_00129200	+
chr7	1459519	1639496	region_76	NM_002360	+
chr7	27119984	27260463	region_32	NM_152739	+
chr7	27119984	27260463	region_32	NM_153631	+
chr7	27119984	27260463	region_32	NM_153620	+
chr7	27119984	27260463	region_32	NM_005522	+
chr7	27119984	27260463	region_32	NM_006735	+
chr7	27119984	27260463	region_32	NM_030661	+
chr7	27119984	27260463	region_32	NM_006896	+
chr7	27119984	27260463	region_32	NM_024014	+

chr7	27119984	27260463	region_32	NM_002141	+
chr7	27119984	27260463	region_32	NM_019102	+
chr7	27119984	27260463	region_32	NR_037939	+
chr7	27119984	27260463	region_32	NM_005523	+
chr7	27119984	27260463	region_32	NM_018951	+
chr7	27119984	27260463	region_32	NM_000522	+
chr7	73865675	74005453	region_493	NM_016328	+
chr7	73865675	74005453	region_493	NM_005685	+
chr7	73865675	74005453	region_493	NM_00119920	+
chr7	98963469	99083290	region_295	NM_00101325	+
chr7	98963469	99083290	region_295	NM_213603	+
chr7	99677928	99728749	region_696	NM_017715	+
chr7	99677928	99728749	region_696	NM_00131813	+
chr7	99677928	99728749	region_696	NM_032924	+
chr7	99677928	99728749	region_696	NM_00131813	+
chr7	99677928	99728749	region_696	NM_00131813	+
chr7	99677928	99728749	region_696	NM_00127828	+
chr7	99677928	99728749	region_696	NM_00127829	+
chr7	99677928	99728749	region_696	NM_00127829	+
chr7	99677928	99728749	region_696	NM_00127829	+
chr7	99677928	99728749	region_696	NM_00127828	+
chr7	148841350	148960187	region_246	NM_020781	+
chr7	148841350	148960187	region_246	NM_003575	+
chr7	148841350	148960187	region_246	NM_170686	+
chr7	148841350	148960187	region_246	NM_012256	+
chr7	148841350	148960187	region_246	NM_00130348	+
chr7	148841350	148960187	region_246	NM_00119522	+
chr8	10528780	10594175	region_584	NM_031439	+
chr8	144288476	144379444	region_341	NM_138465	+
chr8	144288476	144379444	region_341	NM_00127115	+
chr8	144288476	144379444	region_341	NM_173832	+
chr8	144288476	144379444	region_341	NM_030895	+
chr8	144399062	144700788	region_17	NM_201589	+
chr8	144766237	144823208	region_195	NM_00128880	+
chr8	144766237	144823208	region_195	NR_110193	+
chr8	144766237	144823208	region_195	NR_110191	+
chr8	144766237	144823208	region_195	NR_110190	+
chr8	144766237	144823208	region_195	NM_00128880	+
chr8	144766237	144823208	region_195	NM_00128880	+
chr8	144766237	144823208	region_195	NR_110192	+
chr8	144766237	144823208	region_195	NM_00128880	+
chr8	144766237	144823208	region_195	NM_173831	+
chr8	144766237	144823208	region_195	NM_00110059	+
chr8	144766237	144823208	region_195	NM_00110059	+
chr8	144766237	144823208	region_195	NM_00128880	+
chr8	145502460	145562298	region_492	NM_005526	+
chr8	145502460	145562298	region_492	NM_031309	+
chr8	145577287	145704986	region_50	NM_003923	+
chr8	145979591	146079057	region_180	NR_134285	+

chr8	145979591	146079057	region_180	NM_030580	+
chr8	145979591	146079057	region_180	NM_138367	+
chr8	145979591	146079057	region_180	NM_00128676	+
chr8	145979591	146079057	region_180	NM_00128677	+
chr8	145979591	146079057	region_180	NR_134284	+
chr8	145979591	146079057	region_180	NM_003416	+
chr8	145979591	146079057	region_180	NM_213605	+
chr8	145979591	146079057	region_180	NM_00131793	+
chr8	145979591	146079057	region_180	NM_00128279	+
chr8	145979591	146079057	region_180	NM_00128279	+
chr8	145979591	146079057	region_180	NM_00128279	+
chr9	34545590	34672685	region_598	NM_00101736	+
chr9	35711659	35757771	region_729	NM_006368	+
chr9	130150172	130214797	region_673	NM_00128669	+
chr9	130150172	130214797	region_673	NM_00128669	+
chr9	130150172	130214797	region_673	NM_007135	+
chr9	130150172	130214797	region_673	NM_00128669	+
chr9	137105434	137353011	region_26	NM_002957	+
chr9	137105434	137353011	region_26	NM_00129192	+
chr9	137105434	137353011	region_26	NM_00129192	+
chr9	138948168	139141359	region_166	NM_178138	+
chr9	138948168	139141359	region_166	NM_014564	+
chrX	153166567	153295210	region_363	NM_004992	+
