# Supplementary material for: GRHL3 binding and enhancers rearrange as epidermal keratinocytes transition between functional states
Source: PLoS Genet. 2017 Apr 26;13(4):e1006745. doi: 10.1371/journal.pgen.1006745 (PMC5425218; doi:10.1371/journal.pgen.1006745)
Supplement: S4 Table — (PDF) [file pgen.1006745.s017.pdf]

Table S4. Motif enrichment in NHEK-D or NHEK-M SE for transcription factors differentially expressed in keratinocyte differentiation or migration, respectively.

**NHEK-D:**	**Motif ID**	**Name**	***p*-value**	**Adjusted *p*-value**
	MA0481.1	**FOXP1**	2.10E-156	2.01E-154
	MA0162.2	EGR1	3.22E-146	3.09E-144
	MA0039.2	Klf4	2.04E-141	1.96E-139
	MA0773.1	MEF2D	1.47E-121	1.41E-119
	PH0100.1	LMX1A	6.14E-108	5.89E-106
	MA0079.2	SP1	4.86E-93	4.67E-91
	MA0073.1	RREB1	8.84E-91	8.49E-89
	MA0508.1	**PRDM1**	3.87E-74	3.71E-72
	MA0146.1	Zfx	2.83E-70	2.71E-68
	MA0120.1	ID1	1.66E-65	1.60E-63
	MA0602.1	Arid5a	7.39E-58	7.09E-56
	PH0120.1	NKX6-3	4.74E-49	4.55E-47
	MA1099.1	Hes1	1.33E-48	1.27E-46
	MA0507.1	POU2F2	8.18E-47	7.86E-45
	MA0050.1	IRF1	6.33E-45	6.08E-43
	MA0830.1	TCF4	7.88E-38	7.56E-36
	MA0104.2	Mycn	7.46E-36	7.16E-34
	MA0908.1	HOXD11	1.19E-35	1.15E-33
	MA0511.1	RUNX2	2.92E-35	2.80E-33
	MA0024.2	E2F1	2.60E-32	2.50E-30
	PH0082.1	IRX2	5.86E-32	5.63E-30
	MA0098.3	**ETS1**	1.20E-31	1.16E-29

MA0650.1	HOXA13	2.15E-31	2.06E-29
MA0664.1	MLXIPL	2.75E-28	2.64E-26
MA0824.1	ID4	1.66E-27	1.59E-25
MA0476.1	**FOS**	1.90E-26	1.82E-24
MA0062.1	**GABPA**	4.94E-25	4.74E-23
MA0031.1	FOXD1	8.45E-25	8.11E-23
MA0655.1	JDP2	2.44E-23	2.35E-21
MA0470.1	E2F4	4.99E-23	4.79E-21
MA0825.1	MNT	2.62E-22	2.52E-20
**Lrrfip**	**Lrrfip**	2.49E-19	2.39E-17
MA0603.1	Arntl	2.20E-18	2.11E-16
MA0591.1	Bach1::Mafk	1.54E-17	1.48E-15
MA0515.1	**Sox6**	1.58E-17	1.52E-15
MA0840.1	CREB5	2.35E-15	2.26E-13
MA0834.1	ATF7	8.22E-14	7.89E-12
MA0144.2	STAT3	9.53E-14	9.15E-12
MA0513.1	SMAD2::SMAD3::SMAD4	4.25E-13	4.08E-11
MA0137.3	STAT1	7.41E-13	7.11E-11
MA0692.1	TFEB	1.96E-12	1.88E-10
MA0745.1	SNAI2	3.05E-11	2.92E-09
MA0626.1	NPAS2	5.07E-10	4.87E-08
MA0605.1	Atf3	1.87E-09	1.79E-07
MA0483.1	Gfi1b	3.40E-09	3.26E-07
MA0520.1	STAT6	7.39E-09	7.10E-07

MA0620.1	Mitf	1.02E-08	9.75E-07
MA0903.1	HOXB3	2.36E-08	2.27E-06
--	PBX2	5.19E-08	4.99E-06
--	OVOL1	1.58E-07	1.51E-05
MA0510.1	RFX5	1.87E-07	1.80E-05
MA0863.1	MTF1	3.57E-06	3.42E-04
MA0844.1	XBP1	1.34E-05	1.29E-03
MA0075.2	PRRX2	4.68E-05	4.48E-03
MA0869.1	SOX11	5.07E-04	4.76E-02

NHEK-M SE:

ID	Name	*p*-value	Adjusted *p*-value
MA0481.1	FOXP1	3.24E-265	1.36E-263
MA0162.2	EGR1	7.58E-220	3.18E-218
MA0508.1	PRDM1	3.15E-124	1.32E-122
MA1099.1	HES1	5.84E-102	2.45E-100
MA0905.1	HOXC10	2.22E-93	9.32E-92
MA0830.1	TCF4	1.25E-90	5.23E-89
MA0650.1	HOXA13	4.70E-81	1.97E-79
MA0120.1	ID1	4.66E-64	1.96E-62
MA0156.1	FEV	5.73E-64	2.41E-62
MA0137.3	STAT1	5.90E-54	2.48E-52
MA0816.1	ASCL2	7.00E-45	2.94E-43
MA0603.1	Arntl	4.66E-39	1.96E-37
MA0098.3	ETS1	9.90E-31	4.16E-29
MA0515.1	Sox6	1.34E-29	5.62E-28
MA0880.1	DLX3	9.10E-20	3.82E-18
MA0626.1	NPAS2	2.81E-18	1.18E-16
MA0840.1	CREB5	3.32E-15	1.40E-13
MA0512.1	Rxra	3.33E-10	1.40E-08
MA0764.1	ETV4	4.31E-10	1.81E-08
MA0759.1	ELK3	1.16E-06	4.87E-05
