# Supplementary material for: GRHL3 binding and enhancers rearrange as epidermal keratinocytes transition between functional states
Source: PLoS Genet. 2017 Apr 26;13(4):e1006745. doi: 10.1371/journal.pgen.1006745 (PMC5425218; doi:10.1371/journal.pgen.1006745)
Supplement: S5 Table — (PDF) [file pgen.1006745.s018.pdf]

Rank	SE number	GRHL3 peaks	Linked gene	Reference(s)	chr	start	stop
1	region_2	78	--	--	chr19	1379295	1993229
2	region_24	54	IL17C	PMC3577967	chr16	88696950	89081667
3	region_10	52	ELANE, PTRN3	PMC4527112,	chr19	571081	959937
4	region_1	49	SBNO2	PMC3123121	chr19	974463	1336105
5	region_30	46	SUN1	19169254	chr7	841717	1221443

Table S5. Top SE with greatest number of GRHL3 binding events and the psoriasis related genes they overlap
