# Supplementary material for: GRHL3 binding and enhancers rearrange as epidermal keratinocytes transition between functional states
Source: PLoS Genet. 2017 Apr 26;13(4):e1006745. doi: 10.1371/journal.pgen.1006745 (PMC5425218; doi:10.1371/journal.pgen.1006745)
Supplement: S6 Table — (PDF) [file pgen.1006745.s019.pdf]

Table S6. Nearest gene to SE gained when GRHL3 is knocked-down in NHEK-D.
FBLN1
CABIN1
ISLR
HPCAL1
ZBTB21
CPNE5
DENND3
AGXT
SHANK2
ZBTB40
ANKRD11
GRK5
ankrd39
SORCS2
CELF4
ACTRT2
RBFOX3
KCTD5
KIF1A
KRBA1
NTNG2
WNT11
TSPAN18
ZFYVE28
MAP1S
UNC5A
NKD1
PIGG
ARHGEF18
MUC5B
JPH3
ADAMTS2
MGAT5B
GNB1
IRX4
NUP210
ALPPL2
HDAC4
NOTCH3
LDOC1L
pitpna
KIAA2013
AJAP1
PTP4A3
MAN1C1
FLNC
TMEM161A
PDGFA

TRAPPC9
AGPAT3
POLG
MMAB
MATN1
HMG20A
ECE1
CNGB1
JAKMIP3
SPNS3
