# Supplementary material for: GRHL3 binding and enhancers rearrange as epidermal keratinocytes transition between functional states
Source: PLoS Genet. 2017 Apr 26;13(4):e1006745. doi: 10.1371/journal.pgen.1006745 (PMC5425218; doi:10.1371/journal.pgen.1006745)
Supplement: S7 Table — (PDF) [file pgen.1006745.s020.pdf]

Table S7. Dharmacon catalog numbers for siRNA used in screen.

Gene Symbol	Catalog number
ATF2	M-009871-00
ATF3	M-008663-01
ATF4	M-005125-02
ATF5	M-008822-01
ATF7	M-008865-00
BRIP1	M-010587-00
CEBPG	M-011608-01
CREB5	M-008436-02
E2F1	M-003259-01
ETS1	M-003887-00
FOS	M-003265-01
FOSL1	M-004341-04
FOSL2	M-004110-00
FOXD1	M-011862-01
FOXN1	M-009733-00
FOXP1	M-004256-01
GABPB2	M-016074-00
GRHL1	M-020375-00
GRHL2	M-014515-01
GRHL3	M-014017-00
JUN	M-003268-03
JUND	M-003900-05
KLF4	M-005089-03
LRRFIP1	M-019842-01
NOTCH3	M-011093-01
NR3C1	M-003424-03
OVOL1	M-006543-00
OVOL2	M-013793-00
PBX1	M-019680-00
PBX2	M-011746-01
PRDM1	M-009322-02
RARG	M-003439-01
RBL1	M-003298-02
RELB	M-004767-02
RORA	M-003440-01
RUNX1	M-003926-02
RUNX2	M-012665-01
SMAD4	M-003902-01
SOX11	M-017377-02
SOX6	M-015101-01
SP1	M-026959-00
SP3	M-023096-02
STAT1	M-003543-01
STAT3	M-003544-02
STAT6	M-006690-01
TCF4	M-004594-01
TCF7L2	M-003816-01
TP63	M-003330-01
XBP1	M-009552-02
