# Supplementary material for: GRHL3 binding and enhancers rearrange as epidermal keratinocytes transition between functional states
Source: PLoS Genet. 2017 Apr 26;13(4):e1006745. doi: 10.1371/journal.pgen.1006745 (PMC5425218; doi:10.1371/journal.pgen.1006745)
Supplement: S8 Table — (PDF) [file pgen.1006745.s021.pdf]

Table S8. Primer sequences for REST and MED1 ChIPs

ChIP Primers	Sequence
Smap1 F	CTCACTTTCCTCCCCTGTGT
Smap1 R	TTCGCCTGCAGTTTAGAAGC
Metrnl F	ATCTTGGCTGGAAAAGGGGA
Metrnl R	CCCTAGAGACTCAGGCCAGA
Dner F	AAGCTTAGAGAGGGCTGGTC
Dner R	GCGAGGTGTTCAATGTCCAG
Negative (IL5) F	GGGGTAAAATCATCGAGCAA
Negative (IL5) R	CACTGCCTGATTCAATGTCAG
EDC-1 F	GAACCTACTTCAGCCCTGGT
EDC-1 R	AATGGCCACTGGAGGAATGA
EDC-2 F	TGGGAGTGGCTCTAGTCTCT
EDC-2 R	CCTGCCCCAGTTTACAGAGA
TP63-1 F	AGTCTCCTCACTGAAGGCAC
TP63-1 R	CTTCCCTGTCTCTTGGCTGA
TP63-2 F	TTTTCTGCCCTGTCCTCCAT
TP63-2 R	AAGTTAGGGGTATGCGCTGT
MIR205-1 F	CTACTCCCAGACCTTGCACA
MIR205-1 R	ATTCAAGGTGGGAGGAAGGG
MIR205-2 F	TCCACATTCTGAACCCTCCC
MIR205-2 R	GACCACCTACACTTCTGGCT
EGFR-1 F	TGTTGGTCAGGCAAGTCTCA
EGFR1-R	ACAAAAGATGGTTGGCTGGG
EGFR-2 F	TGAGAGAGCGTGAACAGAGG
EGFR-2 R	TCTCCCCTGTAGGTCTCTCC
TE-1 F	CTTGACTTGCCCGAGAGAGA
TE-1 R	GGTAGAGAACATGGCCCTGT
TE-2 F	AACTTAGCCGGGTACAGTGG
TE-2 R	GGGGTCTCGAAATGTTGTCC
Neg control F	TAATGTGAGCCCAGGTGTGT
Neg control R	AGTGAAATGGGCAGGGTGTA
TE-3 F	GGGACACTAGGAAGGCAGTT
TE-3 R	CTGGTGACTCAGCCTCTCAA
